# Multimodal Precise Control Over Multiselective Carbonylation of 1,3-Enynes

**DOI:** 10.1021/jacs.5c00032

**Published:** 2025-02-19

**Authors:** Chang-Sheng Kuai, Yuanrui Wang, Ting Yang, Xiao-Feng Wu

**Affiliations:** † Dalian National Laboratory for Clean Energy, Dalian Institute of Chemical Physics, Chinese Academy of Sciences, Dalian, Liaoning 116023, China; ‡ University of Chinese Academy of Sciences, Beijing 100049, China; § Leibniz-Institut für Katalyse e. V., Albert-Einstein-Straβe 29a, Rostock 18059, Germany

## Abstract

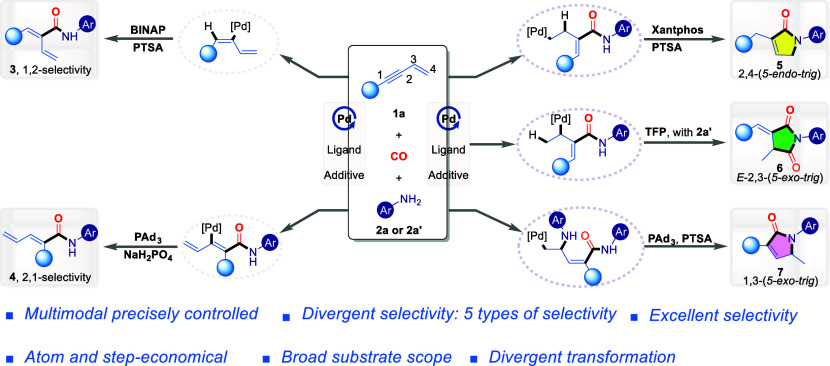

Efficiently constructing structurally diverse and complex organic molecules through selective catalytic functionalization is a central goal in synthetic chemistry, yet achieving precise control over multiple reactive centers in multisite substrates remains a formidable challenge. Building on foundational advances in single- and dual-selective transformations, we report a multimodal strategy for the selective carbonylation of 1,3-enynes, a versatile class of multisite substrates. Through meticulous fine-tuning of the catalytic conditions, our approach enables five distinct regio- and stereoselective carbonylative transformations, including direct functionalization (1,2- and 2,1-hydroaminocarbonylation) and tandem cyclization pathways (2,4-, 1,3-, and 2,3-carbonylation). Furthermore, mechanistic studies suggested that multidimensional precise regulation enables the seamless relay of up to three tandem reactions (hydroaminocarbonylation-hydroamination–transamination) with exceptional accuracy. This unified platform not only establishes a robust framework for tackling the enduring challenges of selectivity control in multisite substrates but also broadens the chemical space accessible through 1,3-enyne transformations, exemplifying atom- and step-economic principles and paving the way for transformative advancements in drug discovery, materials science, and beyond.

## Introduction

**Figure 1:**
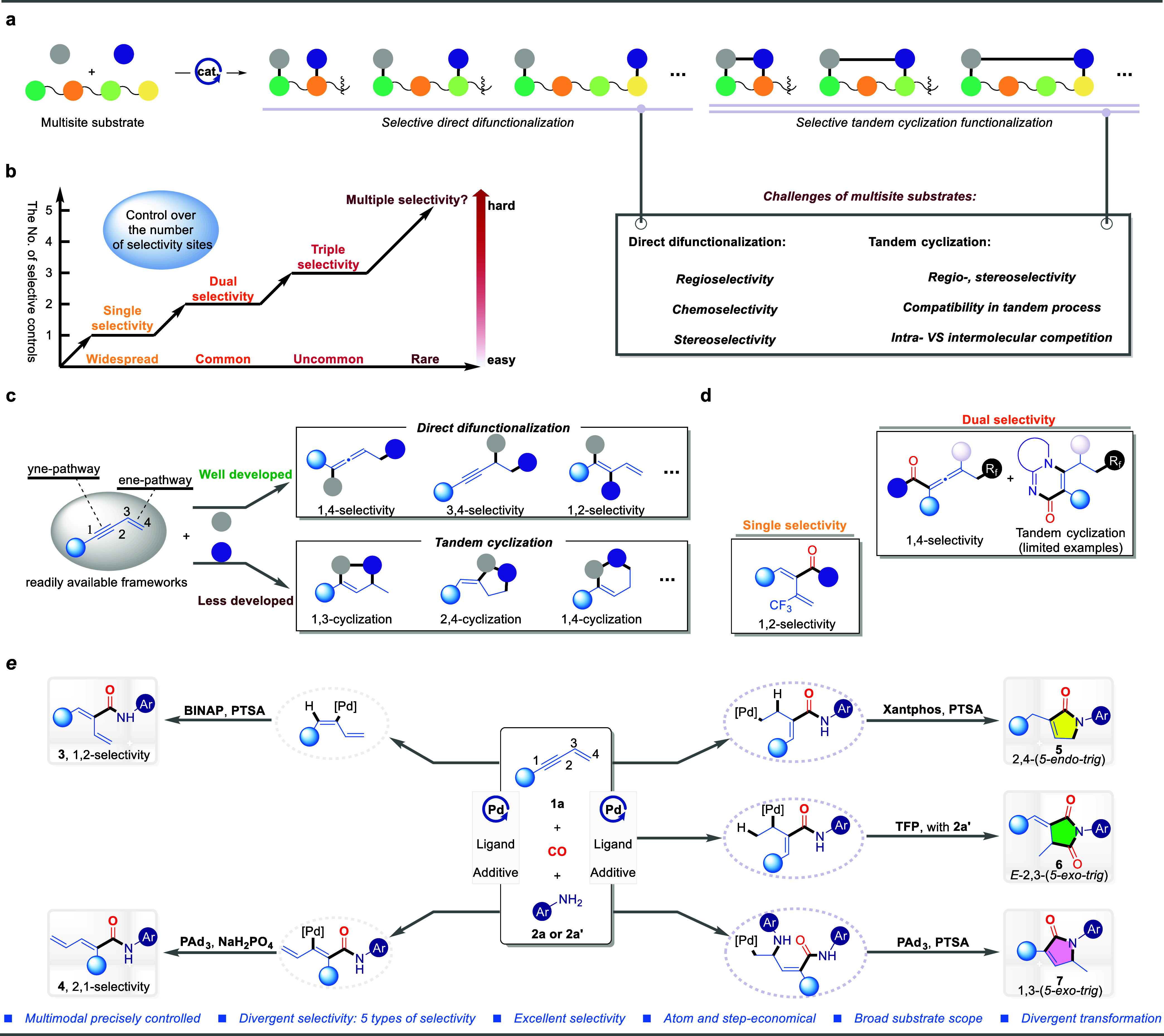
Challenge of selectivity control in substrates with multiple reactive sites. **a**, Selectivity diversity and challenges in reactions involving substrates with multiple reactive sites. **b**, Classification of selective transformations by increasing complexity. **c**, State of art on 1,3-enynes difunctionalization. **d**, Our previous works in 1,3-enynes carbonylation. **e**, This study presents a method for multimodal, precisely controlled multiselective carbonylation of 1,3-enynes.

Designing efficient and straightforward strategies for rapidly constructing structurally diverse organic molecules is a cornerstone of organic synthesis. Achieving molecular diversity through selective catalytic functionalization of the same substrate is undoubtedly more cost- and effort-efficient than conventional methods, which rely on transformations using different reagents and catalysts under individually optimized reaction conditions.^1−7^ However, controlling selectivity becomes considerably more challenging when dealing with multisite substrates containing multiple reactive centers. Those substrates, as shown in Figure 1a, offer immense potential for constructing complex molecular frameworks but also present significant obstacles due to their inherent reactivity and steric biases. Achieving high selectivity in divergent catalysis requires innovative strategies and a deep understanding of the factors governing chemo-, regio-, and stereoselectivity. Furthermore, this challenge is heightened by the necessity to precisely orchestrate direct difunctionalization or tandem cyclization processes, especially when confronted with complex reactivity patterns and competing intra- or intermolecular interactions.^7−10^ Addressing these challenges holds the potential for transformative advancements in drug discovery, materials science, and beyond, paving new frontiers in synthetic chemistry.^11−13^

To better contextualize this challenge, a literature survey was conducted to classify the development and difficulty of selective transformations originating from the same substrate, based on the number of controlled selectivities,^14−21^ as shown in Figure 1b. Over the past few decades, single-selectivity transformations have emerged as the cornerstone of modern synthetic methodologies, finding widespread application in both academic research and industrial practices. Building on this foundational work, recent advances have facilitated the development of dual-selective transformations from the same substrate by merely fine-tuning reaction parameters such as ligands, catalysts, and solvents, achieving remarkable maturity in this field.^22−31^ In stark contrast, while a handful of groundbreaking methods have been reported, transformations involving triple selectivity remain relatively uncommon, primarily due to the heightened demand for meticulous control over each selectivity.^32−39^ Even more challenging are multiselectivity transformations, which have been scarcely explored. The primary obstacles stem from an exponential increase in complexity: additional reactive centers drastically escalate competition between reaction pathways, amplify the likelihood of undesired side reactions, and necessitate multidimensional optimization (e.g., catalysts, ligands, solvents, and additives) to address the unique requirements of multiple reactive sites. Consequently, advancing research in this area not only requires a deeper understanding of catalytic system design and modulation but also holds immense potential to provide transformative strategies for diversity-oriented synthesis in drug discovery and beyond.^40−42^

Functionalization of 1,3-enynes, a readily available class of multisite substrates featuring conjugated double and triple carbon–carbon bonds, has garnered significant attention in recent years, particularly for their direct functionalization reactions (Figure 1c). As substrates that exhibit remarkable versatility, they can undergo a variety of regioselective coupling transformations, such as 1,2-, 3,4-, and 1,4-functionalizations, among others.^43−55^ However, despite the remarkable progress in this field, the efficient one-step tandem cyclization for the construction of more complex frameworks remains largely underexplored, with the majority of studies predominantly focusing on single-selectivity transformations.^56−61^ Building on our long-standing interest in carbonylation chemistry^62−65^ and prior research on enyne carbonylation, we successfully established single-selective and substrate-controlled dual-selective enyne carbonylation reactions (Figure 1d).^66,67^ Inspired by these advancements, we envisioned the development of a more challenging unified strategy capable of achieving multimodal selectivity control while ensuring broad applicability and operational simplicity.

Herein, we introduce a multimodal and precisely controlled strategy for the multiselective carbonylation of 1,3-enynes (Figure 1e). By the fine-tuning of multidimensional catalytic conditions, this platform facilitates five distinct transformations, encompassing direct functionalization (1,2- and 2,1-hydroaminocarbonylation) and tandem cyclization (2,4-, 1,3-tandem carbonylation, and 2,3-tandem dicarbonylation) pathways. Remarkably, this strategy achieves exceptional control over regio- and stereoselectivity while adhering to principles of atom and step economy.

## Results and Discussion

**Figure 2:**
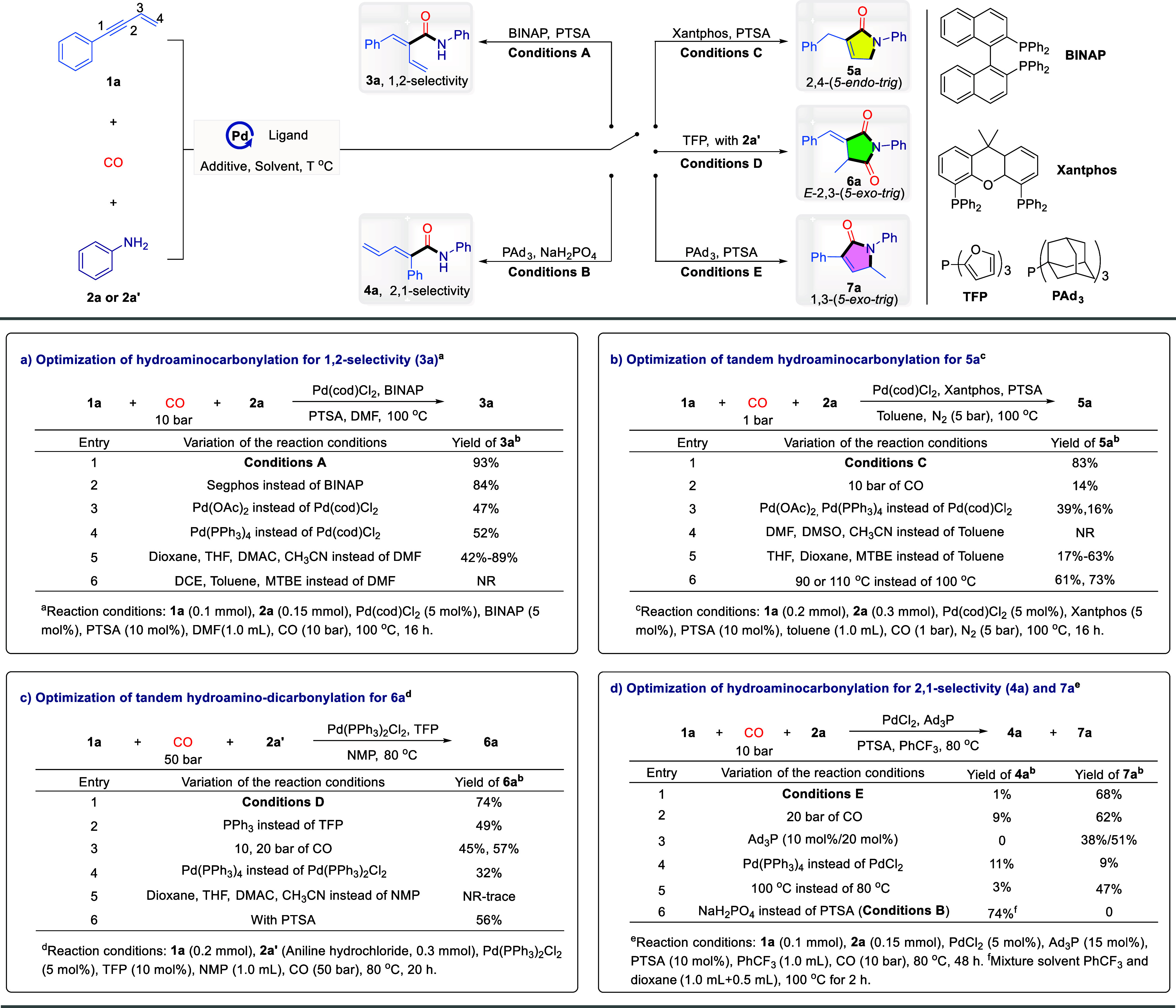
Optimization studies of multiselective carbonylation of 1,3-enynes. ^b^Yield was determined by GC using ^n^hexadecane as the internal standard.

To achieve this multiselectivity control, we embarked on exploratory studies focused on the selective carbonylation of 1,3-enynes. Using Pd(cod)Cl_2_ and PTSA as the catalytic system, 1,3-enynes **2a** and aniline **1a** was employed as model substrates under a CO atmosphere (Figure 2a). Fortunately, through systematic experimentation, α,β-unsaturated amide **3a** was obtained with excellent 2,1-selectivity and 93% yield under the conditions of the bisphosphine BINAP ligand (entry 1). When Segphos was used in place of BINAP exhibited slightly lower catalytic efficiency (entry 2), and the same issue was observed with the different palladium catalysts (entries 3–4). Through solvent screening, DMF was the most effective solvent, while other polar solvents (dioxane, DMAC) exhibited lower yields, and nonpolar solvents (DCE, Toluene) showed no activity in this catalytic system (entries 5–6).

Subsequently, efforts were shifted to the more challenging tandem cyclocarbonylation reactions (Figure 2b). Surprisingly, a 5-endo-trig lactam, which does not conform to Baldwin’s rules, was obtained from a 2,4-tandem cyclocarbonylation. Upon switching to the Xantphos ligand under 1 atm of carbon monoxide in a nonpolar solvent delivered the lactam **5a** in 83% yield (entry 1). When the carbon monoxide pressure was increased to 10 bar, the yield sharply dropped to 14% (entry 2), causing significant disruption in the catalytic system. Furthermore, other catalytic precursors (Pd^II^ or Pd^0^) exhibited even lower activity (entry 3). Polar solvents (such as DMF, DMSO, CH_3_CN) inhibited the formation of product **5a**, while other nonpolar solvents (THF, MTBE) did not enhance catalytic performance (entries 4–5). Similarly, adjusting the reaction temperature, either higher or lower, proved unfavorable for the formation of **5a** (entry 6).

After achieving the tandem cyclocarbonylation reaction, we further explored the possibility of realizing the tandem cyclo-dicarbonylation of enynes to synthesize succinimides (Figure 2c). To investigate the feasibility of tandem cyclo-dicarbonylation, we replaced the diphosphine ligand with a monophosphine ligand to enhance the coordination ability of carbon monoxide with the catalyst. Following the conversion of the nucleophile aniline to its hydrochloride salt **2a’**, we validated our hypothesis, achieving the selective 2,3-tandem cyclo-dicarbonylation to form *E*-5-exo-trig succinimide **6a** under TFP ligand conditions, with a yield of 74%, which is superior to that obtained with PPh_3_ (entries 1–2). This catalytic process requires high CO pressure, as lowering it results in a decreased yield of **6a** (entry 3). A similar trend is observed when changing the Pd^0^ catalytic precursor (entry 4). Additionally, other solvents either inhibit the reaction or diminish catalytic activity within the system (entry 5). Lastly, in this catalytic system, PTSA do not enhance the yield (entry 6), which may indicate that the **2a’** plays a significant role in influencing reaction selectivity. Notably, the use of aniline hydrochloride here instead of free aniline is for its slow release which might affect the reaction basicity. Additionally, the presence of HCl can favor the generation of palladium hydride. However, no desired product could be detected if we separately add free aniline and free HCl (even catalytic amount) which might due to the interaction between HCl and phosphine ligand.

We have demonstrated that regioselective control in tandem cyclocarbonylation (**5a** vs **6a**) can be achieved through various modalities. Building on this, we aimed to alter the regioselectivity of the first hydroaminocarbonylation step (Figure 2d). Surprisingly, when employing the bulky electron-donating ligand PAd_3_, the 1,3-selective tandem cyclocarbonylation 5-endo-trig lactam **7a** was obtained in 68% yield, with only 1% 2,1-selectivity of the α,β-unsaturated amide **4a** (entry 1). Increasing the CO pressure to 20 bar diminished the selectivity of the reaction system (entry 2). Neither decreasing nor increasing the ligand loading improved the reaction yield (entry 3). The use of the catalyst precursor Pd(PPh_3_)_4_ reduced both catalytic activity and selectivity, highlighting the critical role of the PAd_3_ ligand (entry 4). Additionally, increasing the reaction temperature led to lower selectivity (entry 5). Finally, substituting PTSA with a weakly acidic additive NaH_2_PO_4_ restricted the reaction to the 2,1-selective hydroaminocarbonylation product, the α,β-unsaturated amide **4a**, yielding 74% (entry 6).

Having established the optimal conditions, the generality of substrates toward 2,4-tandem cyclocarbonylation was first explored with the assistance of ligand Xantphos and PTSA in toluene. As shown in Table 1 (left), applying unsubstituted aniline **2a** to the standard conditions yielded the lactam **5a** in 76% isolated yield. Whether the aniline’s benzene ring is substituted with electron-withdrawing or electron-donating groups at the *para*-position, the expected products (**5b**–**5l**) were obtained in good yields under standard conditions. For instance, electron-donating groups such as Me, ^t^Bu, OMe, OPh, OCF_3_, and even SMe and NMe_2_, as well as the electron-withdrawing group CF_3_, were well tolerated. The structure of **5h** has been further confirmed by single crystal X-ray crystallography (CCDC: 2383423). Notably, even the Br-substituted aniline, typically sensitive to transition metal catalysts, successfully afforded the desired product in 51% yield. This outcome provides opportunities for further diversification of these substituents in subsequent transformations. The broad applicability of *meta*-substituents further demonstrates the reaction system’s compatibility with varying electronic effects (**5m**-**5p**). Due to steric hindrance, the presence of *ortho*-methyl substituents on aniline inhibits the occurrence of the reaction (**5q**). When aminoindan and naphthylamine were subjected to the standard condition, the corresponding products **5r**, **5s** were obtained in 67%, 57% yields. Moreover, heteroarylamines also demonstrated compatibility with this catalytic system (**5t**).

**Table 1:**
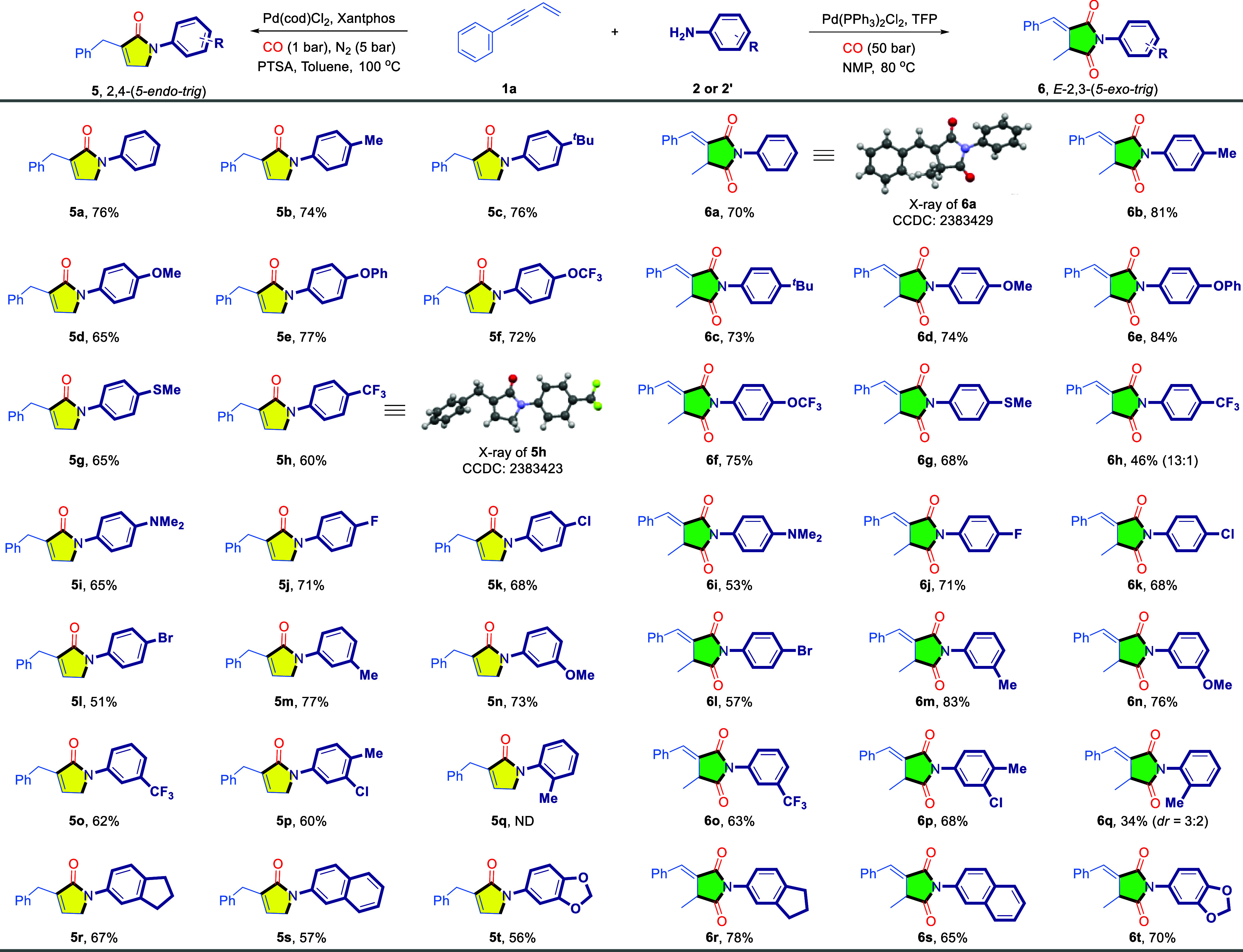
Scope of 2,4- and 2,3-Tandem Cyclocarbonylation of 1,3-Enyne with Respect to the Aniline^a^^,^^b^

a General condition: Conditions C: **1a** (0.2 mmol), **2** (0.3 mmol), Pd(cod)Cl_2_ (5 mol %), Xantphos (5 mol %), PTSA (10 mol %), toluene (1.0 mL), CO (1 bar), N_2_ (5 bar), 100 °C, 16 h.

b Conditions D: **1a** (0.2 mmol), **2’** (ArNH_2_·HCl, 0.3 mmol), Pd(PPh_3_)_2_Cl_2_ (5 mol %), TFP (10 mol %), NMP (1.0 mL), CO (50 bar), 80 °C, 20 h. All yields were isolated yields. The ratio was determined by ^1^H NMR.

The substrate tolerance for the 2,3-tandem cyclo-dicarbonylation was subsequently investigated (Table 1, right). By employing the TFP ligand and using aniline hydrochloride **2’** as the substrate, simple aniline was efficiently converted to succinimide **6a** with a 70% yield. Furthermore, the structure of **6a** was further confirmed by single-crystal X-ray crystallography (CCDC: 2383429). Substrates bearing electron-donating groups at the *para*-position of aniline hydrochloride all underwent the reaction smoothly, affording the corresponding products with moderate to good yields (**6b**–**6g**, **6i**). However, the introduction of a CF_3_ electron-withdrawing group at the para position markedly suppressed the reaction, yielding only 46% of the product **6h**. Additionally, a slight degree of olefin isomerization was observed, leading to the formation of maleimide **6h’**, with a product ratio of 13:1. Halides such as fluoro, chloro and bromide substituents, exhibited excellent compatibility, facilitating reactions that yielded the corresponding products in favorable yields (**6j**–**6l**). Substrates bearing either electron-withdrawing or electron-donating groups at the *meta*-position also demonstrated commendable reactivity, resulting the corresponding products in good yields (**6n**–**6p**). Furthermore, no isomerization to the maleimide product was detected with the CF_3_ substitution (**6o**). Despite the steric hindrance caused by *ortho*-methyl substituent, which typically inhibits the reaction in 2,4-tandem cyclo-dicarbonylation, the desired product can be achieved under TFP ligand conditions, yielding 34% (**6q**). Finally, aminoindan, naphthylamine, and heteroarylamines all demonstrated excellent compatibility within this reaction system, yielding the desired products (**6r**–**6t**) with yields ranging from 65% to 78%. However, the reactions failed when alkyl amines were tested instead of aniline.

Next, we directed our focus to explore the substrate scope of 1,3-enyne derivatives under the current regiodivergent strategy (Table 2). A range of aryl-substituted 1,3-enynes was employed in the investigation. Under Xantphos conditions, substrates bearing either electron-donating groups (Me, OMe, OCF_3_, F, Cl, Br) or electron-withdrawing groups (CF_3_, NO_2_, CN, COOMe) on the phenyl ring were all well-tolerated, affording the corresponding products with yields ranging from 41% to 75% (**5u**–**5aj**). When switching to the TFP/**2a’** conditions, substrates with electron-donating groups were smoothly converted into the corresponding products, with yields ranging from 45% to 70% (**6u**–**6z**, **6af and 6ag**). However, due to steric hindrance, the *ortho*-methyl-substituted enyne significantly suppressed the reaction, resulting in a yield of only 17% (**6ae**). When the substrates with electron-withdrawing groups, olefin isomerization is promoted, leading to maleimide products. For example, the CF_3_-substituted substrate resulted in a 2.3:1 mixture of two isomers (**6aa**:**6aa’**), whereas the CN- and COOMe-substituted substrate exclusively produced the maleimide product **6ac’** and **6ad’**. However, the nitro substituent directly suppressed the reaction (**6ab**), likely due to the incompatibility of the nitro group with the catalytic system. This inhibition suggests that the nitro group interferes with the reaction pathway, rendering the catalytic conditions ineffective. Furthermore, naphthyl, heterocyclic, and alkyl-substituted 1,3-enynes were also evaluated under the 2,4-tandem cyclo-carbonylation conditions. With the exception of substrates containing reactive amide protons, which were incompatible with this catalytic system (**5al**), all other substrates successfully afforded the desired lactam products (**5ah**–**5ak**). Then shifting to the 2,3-tandem cyclo-dicarbonylation conditions, all substrates successfully yielded the desired products, except for the pyridine-substituted enyne, which led to the isomerized maleimide **6ai’**. Notably, substrates with reactive amide protons, which were previously incompatible, performed well under this condition, delivering the target product with a yield of 76% (**6al**). However, reaction with enyne with substation at the alkene position failed.

**Table 2:**
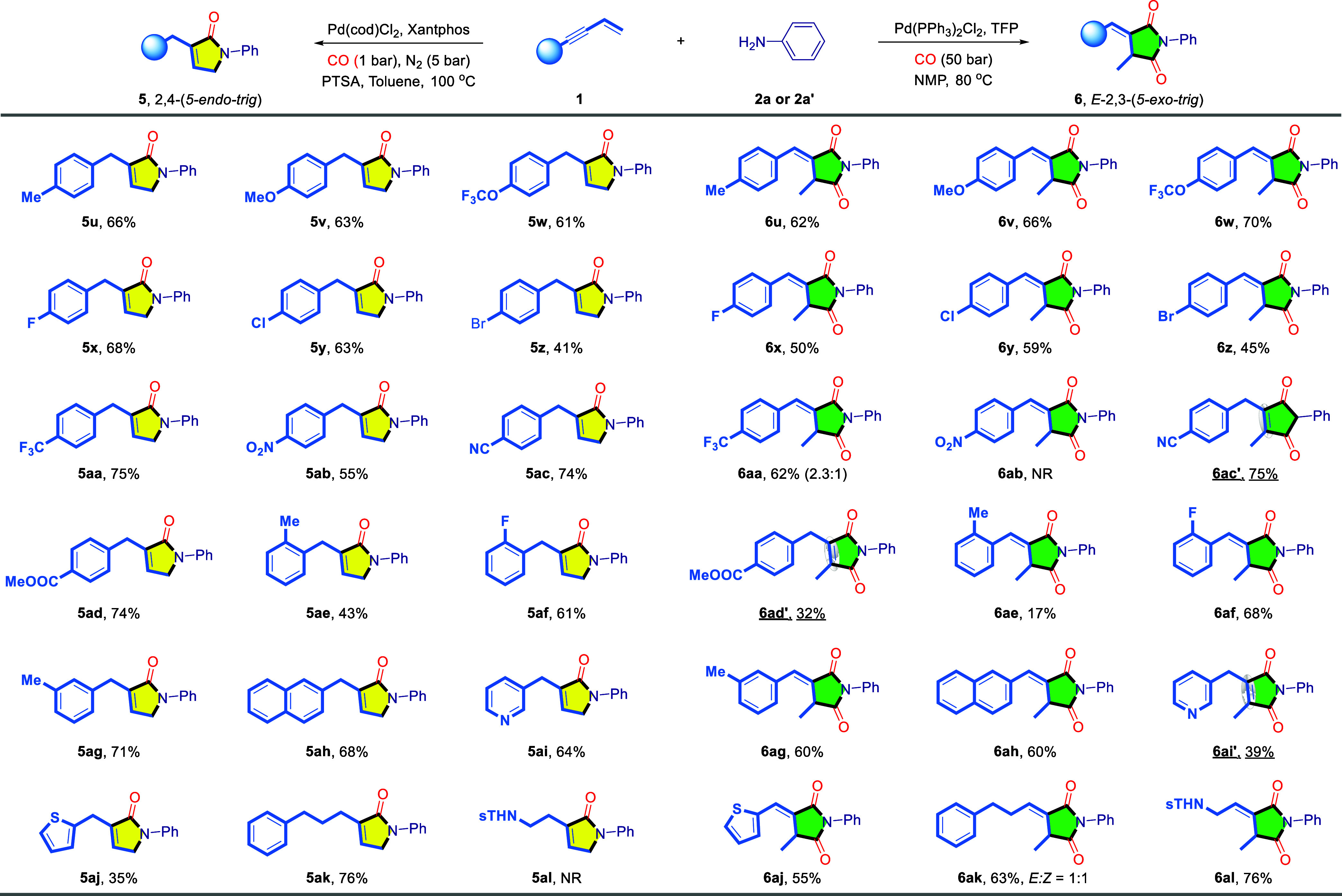
Scope of the Reaction with Respect to the 1,3-Enyne Substrate^a^^b^

a General condition:Conditions C: **1** (0.2 mmol), **2a** (0.3 mmol), Pd(cod)Cl_2_ (5 mol %), Xantphos (5 mol %), PTSA (10 mol %), toluene (1.0 mL), CO (1 bar), N_2_ (5 bar), 100 °C, 16 h.

b Conditions D: **1a** (0.2 mmol), **2a’** (PhNH_2_·HCl, 0.3 mmol), Pd(PPh_3_)_2_Cl_2_ (5 mol %), TFP (5 mol %), NMP (1.0 mL), CO (50 bar), 80 °C, 20 h. All yields were isolated yields. The ratio was determined by ^1^H NMR.

Subsequently, the 1,3-tandem cyclocarbonylation reactions were investigated under optimized conditions (Table 3). The initial investigation, using aniline as the substrate, afforded the corresponding *5-exo-trig* lactam product **7a**, with an isolated yield of 65%. A variety of substituents, including ^t^Bu, OCF_3_, SMe, halides (F, Cl, Br), and CF_3_, at both the *para-* and *meta-* positions, were well-tolerated, affording lactam products (**7b**–**7k**) in reasonable yields. The structure of compound **7g** was confirmed through X-ray crystallography (CCDC: 2383540). The *meta*-position CF_3_ substituent may have reduced the regioselectivity of the reaction, likely due to electronic effects, resulting in a minor formation of the 2,4-regioselective lactam product, in a **7k**:**5o** = 13:1 selectivity. Naphthylamine and disubstituted anilines also proved to be well-suited for this reaction (**7l**–**7m**). However, the steric hindrance from *ortho*-methyl-substituted aniline inhibited the reaction’s progress (**7n**). Next, the substrate scope of 1,3-enynes was evaluated. Both electron-donating substituents (Me, OMe, OCF_3_, F and etc.) and electron-withdrawing (CF_3_, COOMe) on the enyne were well-tolerated, leading to the desired products with excellent selectivity (**7o**–**7w**). However, steric hindrance from *ortho*-methyl substituents on the phenyl ring of the 1,3-enyne impeded the reaction. Additionally, heteroaryl- and alkyl-substituted 1,3-enynes also proved compatible with this catalytic system (**7z**–**7ac**). Finally, after successfully achieving divergent (2,3-; 2,4- and 1,3-) tandem cyclocarbonylation of 1,3-enynes, we proceeded to explore strategies aimed at halting the reaction at the first step: regioselective hydroaminocarbonylation. Subsequently, the substrate scope for this divergent approach was investigated (Table 4). In the evaluation of aniline substrates, different electronic properties (OMe and CF_3_) exhibited compatibility with both catalytic systems that display excellent regioselectivity (**3a**–**3c**; **4a**–**4c**). Notably, the sterically hindered *ortho*-methyl substituent showed excellent reactivity (**3d**; **4d**), without the inhibition observed in the previous tandem carbonylation reactions. Thus, it can be inferred that the steric effects of the *ortho*-methyl group hinder the progression of the tandem cyclocarbonylation reaction by impeding the second cyclization step. In the investigation of the applicability of 1,3-enyne substrates, both electron-donating (OMe) and electron-withdrawing groups (CF_3_) could yield the desired products (**3e**, **3f**; **4e**, **4f**), although electron-withdrawing groups suppressed the reaction, resulting in moderate yields. Subsequently, substrates featuring sterically hindered *ortho*-methyl and naphthyl substitutions were examined, both of which exhibited good reactivity and yielded the corresponding products with good yields (**3g**, **3h**; **4g**, **4h**).

**Table 3:**
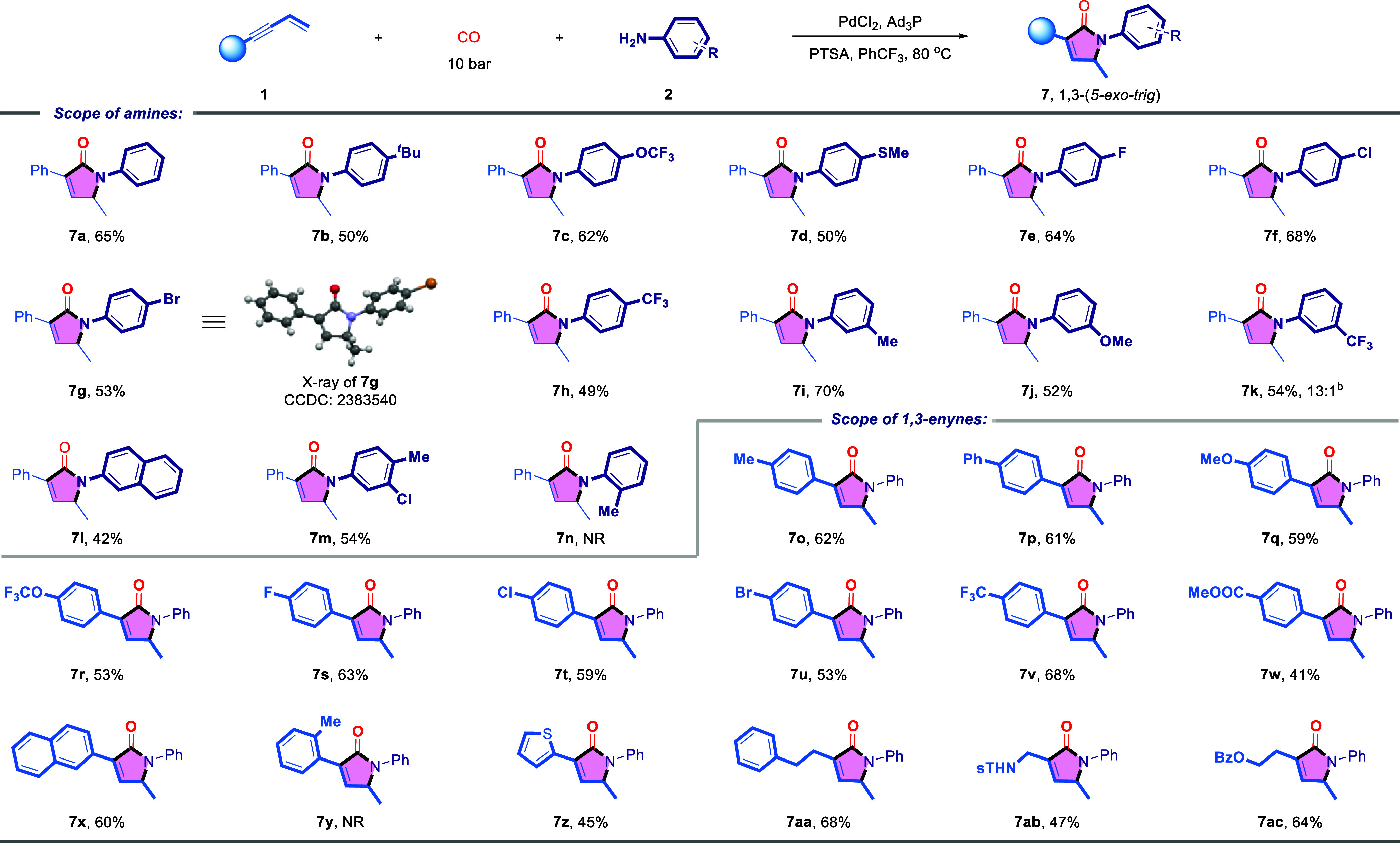
Scope of 1,3-Tandem Cyclocarbonylation^a^

a General condition: Conditions E: **1** (0.1 mmol), **2** (0.15 mmol), PdCl_2_ (5 mol %), Ad_3_P (15 mol %), PTSA (10 mol %), PhCF_3_ (1.0 mL), CO (10 bar), 80 °C, 48 h. All yields were isolated yields.

b The ratio of **7k**:**5o** = 13:1 was determined by ^1^H NMR.

**Table 4:**
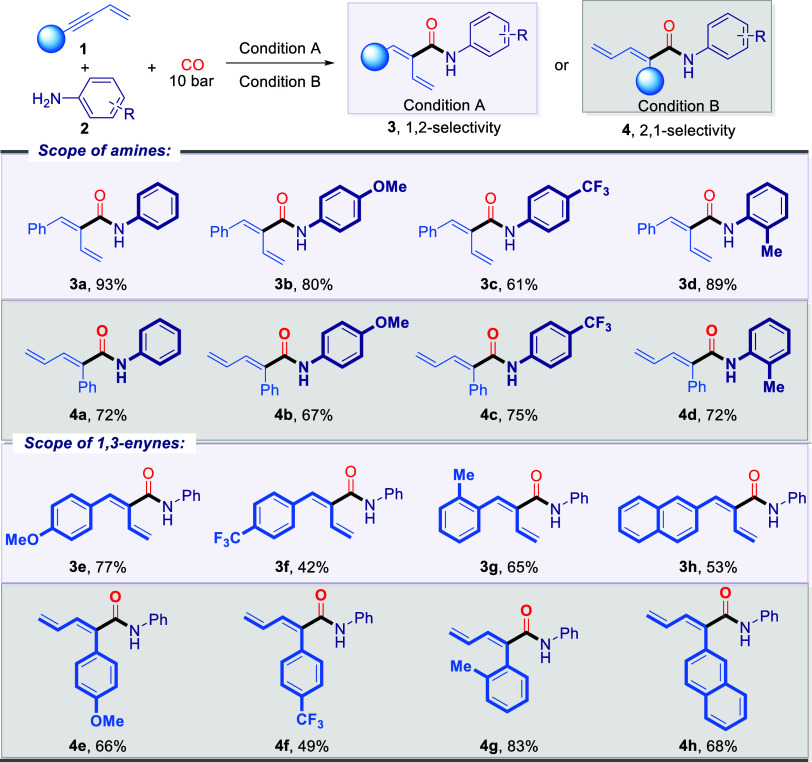
Scope of 1,2- and 2,1-Hydroaminocarbonylation^a^^b^

a General condition: Conditions A: **1** (0.1 mmol), **2** (0.15 mmol), Pd(cod)Cl_2_ (5 mol %), BINAP (5 mol %), PTSA (10 mol %), DMF (1.0 mL), CO (10 bar), 100 °C, 16 h.

b Conditions B: **1** (0.1 mmol), **2** (0.15 mmol), PdCl_2_ (5 mol %), Ad_3_P (15 mol %), NaH_2_PO_4_ (10 mol %), PhCF_3_ (1.0 mL), dioxane (0.5 mL), CO (10 bar), 100 °C for 2 h. All yields were isolated yields.

**Figure 3:**
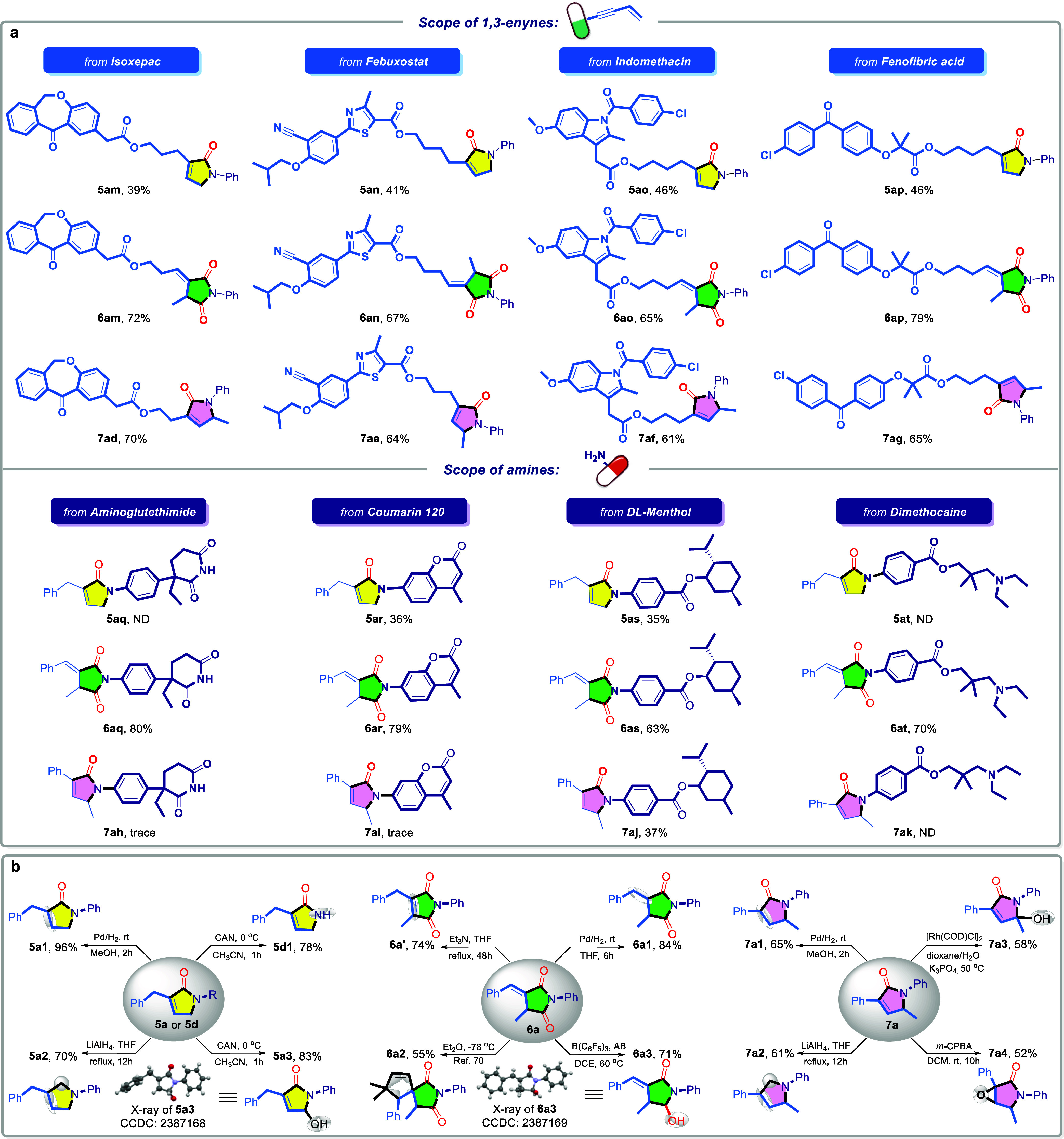
Bioactive molecules modification and derivatizations. **a**, Late-stage functionalization of bioactive natural and synthetic molecules. **b**, Synthetic transformations.

To further demonstrate the synthetic utility of this multimodal-controlled strategy, several key transformations were performed, as illustrated in Figure 3. Given that late-stage functionalization has emerged as an increasingly valuable approach for identifying bioactive compounds, it underscores the need to expand the capabilities of modern synthetic methodologies, particularly in constructing and tolerating molecular complexity.^68,69^ Therefore, various late-stage modifications of complex natural products or drug derivatives of 1,3-enynes and amines were examined to validate the practicality of this multimodal control strategy, focusing on the three selective tandem cyclocarbonylation reactions (Figure 3a). Gratifyingly, the late-stage modification of drug derivatives featuring 1,3-enyne handles, such as Isoxepac, Febuxostat, Indomethacin, and Fenofibric acid, were achieved efficiently, resulting in the formation of three corresponding modified drug molecules with yields ranging from 39% to 79% (**5am**–**5ap**; **6am**–**6ap**; **7ad**–**7ag**), thereby confirming the generality of our method.

Subsequently, we began evaluating the modifications of bioactive natural or drug molecules containing amino groups, such as Aminoglutethimide, Coumarin 120, DL-menthol, and Dimethocaine. However, we found that among these three transformations, the 2,3-tandem cyclo-dicarbonylation exhibited the best functional group tolerance, yielding the corresponding products in good yields (**6aq**-**6at**). In the 2,4-cyclocarbonylation, only Coumarin 120 and DL-menthol yielded the corresponding products, with isolated yields of 36% (**5ar**) and 35% (**5as**), respectively. The catalytic system for the 1,3-tandem cyclocarbonylation was particularly sensitive to substrates with complex amines, yielding the target product exclusively from DL-menthol (**7aj**).

Next, we further explored the synthetic utility of these three tandem cyclocarbonylations by conducting a series of site-selective postmodifications (Figure 3b). Hydrogenation of three template products (**5a**, **6a** and **7a**) using Pd/C as catalyst successfully yielding the corresponding product **5a1**, **6a1** and **7a1** in yields ranging from 65 to 96% yield. The pyrrolidines **5a2** and **7a2** were successfully obtained in good yields by reducing lactams **5a** and **7a** with LiAlH_4_. Then, in the presence of cerium ammonium nitrate (CAN), deprotection of the N-PMP substituted lactam **5d** was achieved, yielding the free lactam **5d1** in good yield. Additionally, when the N-substituent was a phenyl group, the corresponding allylic hydroxylation product **5a3** was obtained, and its structure was confirmed via X-ray analysis. Under Et_3_N conditions, succinimide **6a** underwent double bond isomerization, converting into maleimide **6a’**. Subsequently, the 1,3-dipolar cycloaddition of 2-diazopropane with succinimide **6a** afforded the spiro-pyrazoline product **6a2**.^70^ Moreover, under the catalysis of B(C_6_F_5_), the methyl-side carbonyl group of succinimide **6a** was selectively reduced to the hydroxyl product **6a3** in 71% yields, with its structure was confirmed by single-crystal X-ray analysis. In addition, the rhodium-mediated oxidative C–H functionalization of lactam **7a** produced **7a3** in 58% yield. Finally, oxidation of **7a** with *m*-CPBA yielded the epoxide **7a4** in 52%.

**Figure 4:**
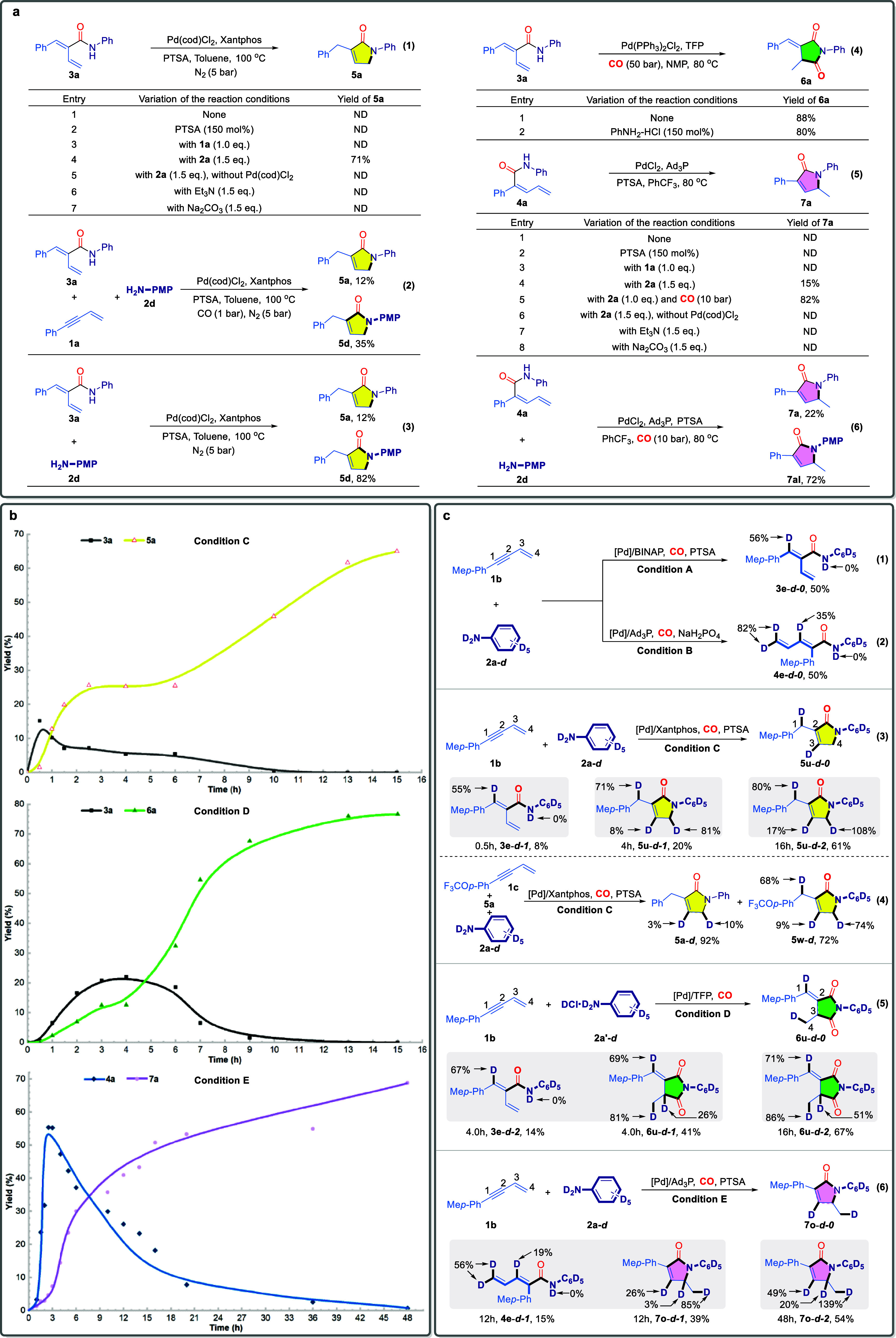
Mechanistic studies.: **a**, Control experiment studies. **b**, Kinetic Studies. **c**, Isotopic labeling studies.

To shed light on the reaction mechanism, several control experiments were conducted (Figure 4a). First, to demonstrate that enamide **3a** is the key intermediate in the first step of the tandem 2,4-cyclocarbonylation reaction, initiated by the selective addition of Pd–H to the enyne, the reaction was conducted under standard conditions in the absence of aniline **2a** and carbon monoxide. However, no further cyclized product **5a** was detected (Figure 4a, eq 1, entry 1). We hypothesized that the stagnation of the tandem reaction might be attributed to insufficient acidity in the system, which could be preventing the in situ formation of the active Pd–H catalyst necessary to initiate the process. To test this assumption, we increased the acidity by adding 150 mol % PTSA to the reaction mixture. However, no formation of the desired product **5a** was observed (eq 1, entry 2). We then shifted our focus to investigate whether the enyne **1a** and aniline **2a** participated in the subsequent tandem reaction. Upon introducing either enyne **1a** or aniline **2a** separately into the reaction, the cyclized product **5a** was indeed obtained in a 71% yield in the presence of **2a**, confirming the role of **2a** in promoting or participating in the cyclization process (eq 1, entries 3–4). Further control experiment demonstrated that the Pd catalyst is essential for the remaining cyclization steps (eq 1, entry 5). To investigate the role of aniline in the subsequent cyclization, aniline was replaced with Et_3_N and Na_2_CO_3_ to evaluate whether the basicity of aniline contributes to the reaction, thereby suggesting that the final cyclization proceeds via hydroamidation.^71,72^ However, the target product was not obtained (eq 1, entries 6–7). In addition, to further confirm the role of **2a**, a cross-reaction experiment was conducted by mixing **1a**, **2d**, and **3a** under standard conditions (Figure 4a, eq 2). The experiment not only yielded the tandem cyclization product **5d** but also the single-step cyclization product **5a**. In a further cross-experiment, **3a** and **2d** were mixed under standard conditions. Surprisingly, we observed the occurrence of a transamination reaction, resulting in the formation of both **5a** and the transamination product **5d**, with yields of 12% and 82%, respectively (Figure 4a, eq 3). Based on the above experimental results, we speculate that enamide **3a** is a key intermediate in the tandem 2,4-cyclocarbonylation reaction. Furthermore, **2a** is essential in the tandem cyclization step. We hypothesize that it may further undergo hydroamination with the terminal alkene of **3a**, followed by an intramolecular transamination to yield the cyclization product **5a**.^73,74^

Subsequently, we turned our attention to the investigation of the intermediates in the 2,3-tandem dicarbonylation reaction. We found that enamide **3a** could be efficiently converted into the target product **6a** under standard conditions, regardless of the presence of aniline hydrochloride **2a’** (Figure 4a, eq 4, entries 1–2). This suggests that **3a** is also a crucial intermediate in this tandem reaction, indicating that the cyclization process does not require the facilitative role of **2a’**. These observations collectively underscore the significance of **3a** in both transformations.

Finally, through retrosynthetic analysis of the 1,3-tandem carbonylation product **7a**, we identified **4a** as the key intermediate preceding the final cyclization. To validate this hypothesis, **4a** was subjected to the standard conditions for the synthesis of **7a**, but the target product was not obtained (Figure 4a, eq 5, entry 1). Subsequently, the addition of 150 mol % PTSA to increase the acidity of the reaction system, or the introduction of **1a**, failed to promote further cyclization (entries 2–3). The introduction of **2a** resulted in a low yield of the target product (15%); however, upon further introduction of CO into the catalytic system, the yield of **7a** dramatically increased to 82% (entries 4–5), indicating that the cyclization step requires CO participation in its catalytic cycle.

Subsequent Pd control and base substitution experiments did not yield the cyclization product **7a** (entries 6–8). Finally, in the cross-reaction experiment (Figure 4a, eq 6), the same transamination phenomenon was observed, yielding both **7a** and **7al**. The above experimental data suggest the following conclusions: 1) **4a** is a crucial intermediate in the formation of the cyclization product **7a**. 2) CO facilitates the smooth progression of the cyclization step. 3) The cyclization mechanism is similar to that of the formation of the cyclization product **5a**, involving hydroamination at the terminal alkene branch, followed by a rapid intramolecular transamination.

To further verify the existence of the two intermediates (**3a** and **4a**) and to elucidate the mechanism within the catalytic system, the kinetic studies of the selective tandem cyclocarbonylation reaction was conducted under conditions C, D, and E. The results are presented in Figure 4b. Under condition C, intermediate **3a** was rapidly generated, reaching its maximum concentration within the first 0.5 h; subsequently, it underwent gradual consumption. In parallel, product **5a** exhibited a sigmoidal kinetic curve that correlated with the formation of **3a**. In contrast, under condition D, involving TFP and **2a’**, the growth of **3a** was more gradual, peaking after 4 h, followed by a symmetrical slow consumption. Similarly, product **6a** displayed an S-shaped growth pattern, occurring concurrently with the generation of **3a**. Finally, under condition D, intermediate **4a** experienced a rapid, volcano-like increase in concentration during the first 3 h, after which it was gradually consumed. Concurrently, product **7a** was produced alongside the formation of **4a**, initially increasing slowly before accelerating rapidly. These findings further substantiate that the hydroaminocarbonyl amides **3a** and **4a** serve as intermediates in those reactions and can be converted into the corresponding products (**5a**, **6a** and **7a**) via subsequent selective cyclization.

Finally, deuterium-labeling experiments for the five selective products (**3a**–**7a**) were conducted under five standard reaction conditions (Condition A–E) to further elucidate the mechanism, as illustrated in Figure 4b. At the outset, employing deuterated aniline **2a-***d* and enyne **1b** under reaction condition A, the corresponding 2,1-selective hydroaminocarbonylation product **3e-***d-***0** was obtained without significant deuterium scrambling. The limited deuterium incorporation, 56% on the alkene and 0% on the amide, suggests that H–D exchange likely occurred with other hydrogen sources present in the catalytic system. However, under the 1,2-selective hydroaminocarbonylation reaction conditions (Condition B), a divergent deuterated product **4e-***d***–0** was obtained, with 35% and 82% deuterium incorporation at the C2 and C4 positions, respectively (Figure 4c, eq 2). This result suggests that the catalytic cycle likely involves a migratory insertion of the acyl-palladium species into the enyne, resulting in the formation of an allylpalladium intermediate, leading to the divergent deuterium incorporation observed at C2 and C4. Subsequently, we conducted the 2,3-tandem cyclocarbonylation reaction under Condition C. Based on our hypothesis that this reaction involves twice selective insertions of Pd–H into the unsaturated bond, we anticipated obtaining the deuterated product **5u-***d***-0,** with deuterium incorporation at the C1 and C3 positions. However, the experimental results indicated the formation of a divergent deuterated product **5u-***d***-2** under standard conditions, with deuterium incorporation rates of 80%, 17%, and 108% observed at the C1, C3, and C4 positions, respectively (Figure 4c, eq 3). We speculate that the deuteration divergence at the C3 and C4 position due occurs to deuterium exchange in the product under the given catalytic conditions. To investigate this, we shortened the reaction time to monitor the deuteration rates at these positions. After 0.5 h, only the hydroaminocarbonylation product **3e-***d***-1** was detected, without any deuterium scrambling. However, after 4 h, the deuteration rate at the C4 position had decreased to only 80% (Figure 4c, eq 3). To further confirm our hypothesis, a cross-reaction was conducted involving the nondeuterated 2,4-tandem cyclocarbonylation product **5a**, alongside trifluoromethoxy-substituted enyne **1c** and deuterated aniline **2a-***d* in a single reaction system. The results revealed that **5a** underwent deuterium exchange at the C3 and C4 positions, with deuteration rates of 3% and 10%, respectively (Figure 4c, eq 4). These observations further substantiate our hypothesis and clarify the underlying reason for the anomalous deuteration at the C3 and C4 positions, demonstrating that deuterium exchange occurred at these sites within the product under the given catalytic conditions. In the 2,3-tandem dicarbonylation transformation, deuterated aniline hydrochloride **2a-***d***’** was employed to conduct the corresponding deuterium-labeling experiment in conditions D (Figure 4c, eq 5). Similarly, deuterium scrambling occurred in product **6u-***d***-2**, with a 51% deuteration rate at the C3 position. Employing the same strategy as before, we reduced the reaction time to 4 h, leading to the detection of two products: **3e-***d***-2** and **6u-***d***-1**. In **3e-***d***-2**, deuterium incorporation occurred as expected at the C1 position, with a rate of 67%. However, in **6u-***d***-1**, compared to **6u-***d***-2**, the C3 position exhibited a lower deuteration rate (26% vs 51%). Finally, under conditions E, we performed a deuterium-labeling experiment on the 1,3-tandem cyclocarbonylation reaction (Figure 4c, eq 6). The expected product **7o-***d***-0** was not detected; instead, we observed the formation of **7o-***d***-2**, which exhibited deuterium scrambling at the C3 position with a deuteration rate of 20%. When the reaction time was reduced to 12h, two products, **4e-***d***-1** and **7o-***d***-1**, were detected. The deuterium incorporation in **4e-***d***-1** was consistent with that in **4e-***d***-0** (Figure 4c, eq 2), indicating that the insertion into the enyne in the first step follows a similar process. However, in the case of **7o-***d***-1**, the deuteration at the C3 position significantly decreased (3%) compared to that of 7o-d-2 (C3, 20%). These results indicate that deuterium exchange occurs within the catalytic systems of all three selective tandem cyclocarbonylation reactions.

**Figure 5:**
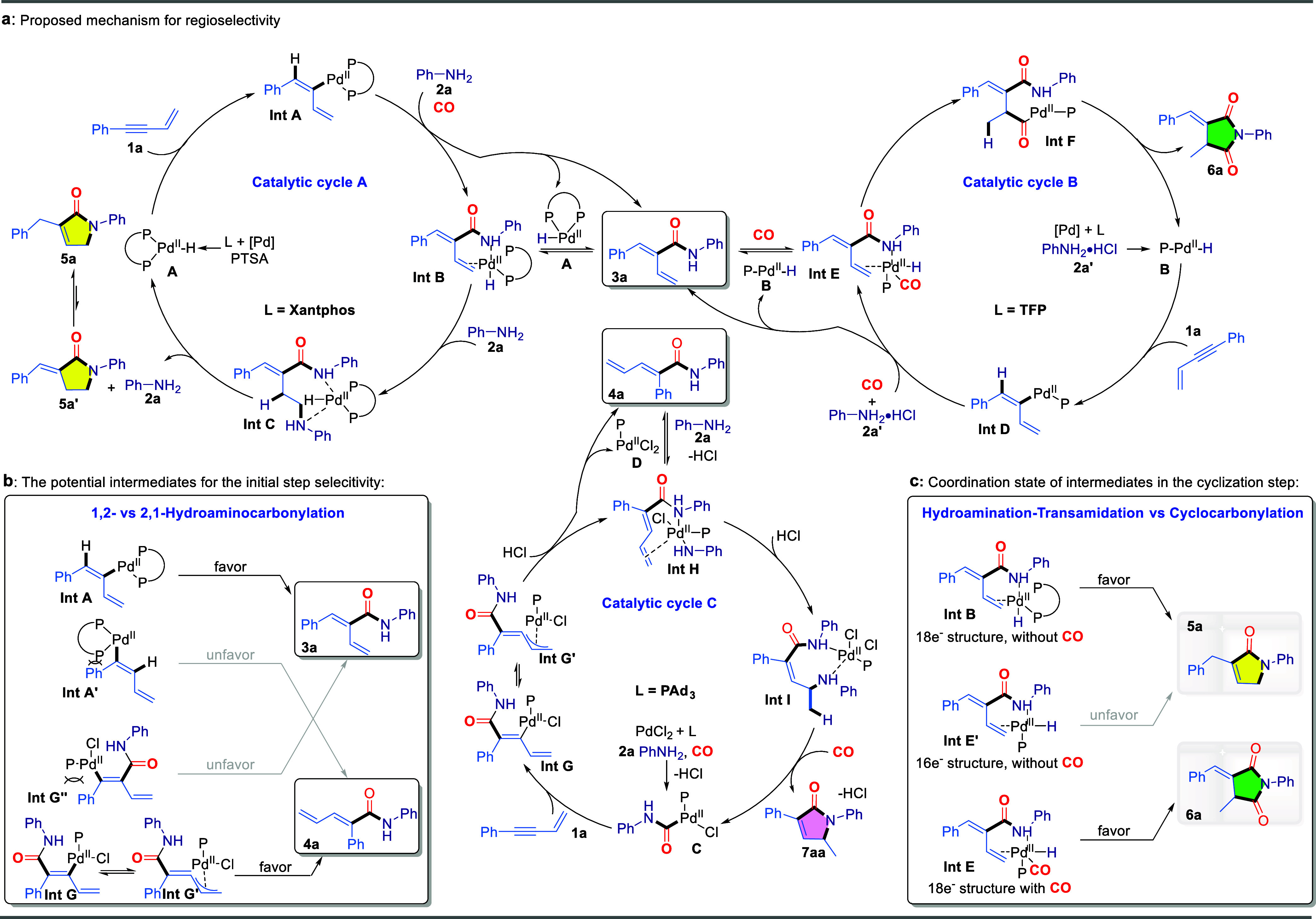
Proposed mechanism and Potential intermediates. a, Proposed mechanism for regioselectivity. b, The potential intermediates for the initial step selectivity. c, The coordination state of intermediates in the cyclization step.

Based on experimental observations and prior studies,^75−79^ three plausible mechanisms have been proposed to elucidate the tandem multimodal-controlled divergent selectivity in the carbonylation of 1,3-enynes (Figure 5a). In the catalytic cycle A. Initially, with the assistance of ligand and PTSA, the active Pd–H species A is generated in situ, which then coordinates to enyne **1a** and undergoes regioselective insertion, forming the alkenyl-palladium intermediate Int A. Subsequently, upon the addition of CO and aniline, carbonylation proceeds, yielding intermediate **3a** and regenerating the active Pd–H species A. Subsequently, the active Pd–H species A inserts into the terminal olefin of **3a** in the presence of aniline, yielding the hydroamination intermediate Int C, as supported by the previous control experiments. Int C then rapidly undergoes intramolecular transamination to form **5a’**, releasing another molecule of aniline. Finally, olefin isomerization occurs, resulting in the formation of the final tandem cyclization product **5a**. For the tandem cyclo-dicarbonylation catalytic mechanism, as illustrated in catalytic cycle B, the active Pd–H species B is generated in situ in the presence of aniline hydrochloride **2a’** and the ligand TFP. This species similarly undergoes selective insertion into the enyne **1a**, followed by carbonylation, leading to the formation of intermediate **3a**, alongside the regeneration of Pd–H B. Subsequently, Pd–H B undergoes a second selective insertion into the terminal olefin of **3a**, followed by a further carbonylation step to yield the acylpalladium species Int F. Finally, nucleophilic attack by the amide on the acylpalladium completes the cyclization, affording the tandem cyclo-dicarbonylation product **6a** and regenerating Pd–H B for the continuation of the catalytic cycle. Finally, the proposed mechanism for the 1,3-tandem cyclocarbonylation is illustrated in catalytic cycle C. The palladium acyl complex C was initially generated from PdCl_2_ in the presence of ligand PAd_3_, amine, and CO. This complex subsequently coordinated with and inserted into enyne **1a**, leading to the formation of the palladium complex Int G. Through isomerization, a more stable allylic palladium intermediate, Int *G*′, was obtained, which upon protonation with hydrochloric acid yielded intermediate **4a** and regenerated the palladium catalyst. This process aligns with the deuterium distribution results observed in isotopic labeling studies for **4a**-*d***-1**. Next, the amino-palladium complex selectively inserted into the olefin of intermediate **4a**, forming the hydroamination intermediate Int I. Finally, with the assistance of palladium catalyst and CO, an intramolecular transamination occurred, producing the final product **7a** and regenerating the active acyl-palladium catalyst C.

By analyzing the steric environment of potential insertion intermediates between the initial active catalytic species and the enyne, we aim to elucidate the underlying factors responsible for the opposing regioselectivities observed in 1,2- and 2,1-hydroaminocarbonylation during the initial step (Figure 5b). Under the bidentate phosphine ligand Xantphos, the in situ generated active Pd–H catalyst coordinates with the enyne and undergoes selective insertion, potentially leading to two insertion pathways: 1,2-addition to form intermediate **Int A**, and 2,1-addition to form **Int A’**. Due to steric hindrance and insights from deuterium labeling studies, the 1,2-addition pathway is favored, resulting in the formation of product **3a**. Given the deuterium labeling study results, switching to the bulky monophosphine ligand PAd_3_ promotes the *in situ* formation of an acyl-palladium species, which then undergoes migratory insertion into the enyne with two possible regioselectivity pathways (1,2- or 2,1-addition). Due to steric hindrance from the phenyl group and the bulky ligand PAd_3_ in intermediate **Int***G***′’**, along with resonance stabilization (**Int G** and **Int***G***′**), the 1,2-addition pathway is favored, ultimately yielding the 2,1-hydroaminocarbonylation product **4a** with distinct apparent regioselectivity.

Finally, based on the analysis of the coordination geometry of the palladium complex, we proposed potential intermediates to elucidate the mechanism of mono- and dicarbonylation control (Figure 5c). In the presence of the bidentate phosphine ligand Xantphos, the coordinatively saturated (18-electron) Pd–H species **Int B** favors further hydroamination-transamination, yielding the tandem cyclized monocarbonylation product **5a**. Switching to the monophosphine ligand TFP provides an additional coordination site, allowing CO to coordinate with palladium in a chelated form. Consequently, the coordinatively saturated (18-electron) species **Int E** is more inclined than the unsaturated (16-electron) intermediate **Int E’** to undergo intramolecular cyclocarbonylation, ultimately yielding the dicarbonylation product **6a**.

## Conclusions

In summary, we developed a multimodal strategy for the selective carbonylation of 1,3-enynes. By meticulously fine-tuning the catalytic conditions, a wide range of structurally diverse scaffolds can be synthesized from the same starting substrate, enabling five distinct regio- and stereoselective transformations, including direct functionalization (1,2- and 2,1-hydroaminocarbonylation) and tandem cyclization pathways (2,4-, 1,3-, and 2,3-carbonylation). The practicality of this approach lies in its use of readily available materials, high selectivity, atom and step economy, broad substrate scope, and its applicability in modifying complex drug-like molecules and other synthetic transformations. Furthermore, mechanistic studies suggested that multidimensional precise regulation enables the seamless relay of up to three tandem reactions (hydroaminocarbonylation-hydroamination-transamination) with exceptional accuracy. This method not only expands the accessible chemical space for 1,3-enyne transformations but also demonstrates its broad applicability and potential to inspire future advancements in drug discovery, materials science, and synthetic methodology.

## Supporting Information Available

General comments, general procedures, analytic data, and NMR spectrum (PDF)

## References

[ref1] Lee Y.-C.; Patil S.; Golz C.; Strohmann C.; Ziegler S.; Kumar K.; Waldmann H. A ligand-directed divergent catalytic approach to establish structural and functional scaffold diversity. Nat. Commun. 2017, 8 (1), 14043 10.1038/ncomms14043. 28195128 PMC5316858

[ref2] Ping L.; Chung D. S.; Bouffard J.; Lee S.-G. Transition metal-catalyzed site- and regio-divergent C–H bond functionalization. Chem. Soc. Rev. 2017, 46, 4299–4328. 10.1039/C7CS00064B. 28537608

[ref3] Zhan G.; Du W.; Chen Y.-C. Switchable divergent asymmetric synthesis via organocatalysis. Chem. Soc. Rev. 2017, 46, 1675–1692. 10.1039/C6CS00247A. 28221384

[ref4] Lee Y.-C.; Kumar K.; Waldmann H. Ligand-Directed Divergent Synthesis of Carbo- and Heterocyclic Ring Systems. Angew. Chem., Int. Ed. 2018, 57, 5212–5226. 10.1002/anie.201710247. 29105306

[ref5] Li L.; Chen Z.; Zhang X.; Jia Y. Divergent Strategy in Natural Product Total Synthesis. Chem. Rev. 2018, 118, 3752–3832. 10.1021/acs.chemrev.7b00653. 29516724

[ref6] Nájera C.; Beletskaya I. P.; Yus M. Metal-catalyzed regiodivergent organic reactions. Chem. Soc. Rev. 2019, 48, 4515–4618. 10.1039/C8CS00872H. 31282495

[ref7] Dorn S. K.; Brown M. K. Cooperative Pd/Cu Catalysis for Alkene Arylboration: Opportunities for Divergent Reactivity. ACS Catal. 2022, 12, 2058–2063. 10.1021/acscatal.1c05696. 36212545 PMC9540610

[ref8] Dhungana R. K.; Kc S.; Basnet P.; Giri R. Transition Metal-Catalyzed Dicarbofunctionalization of Unactivated Olefins. Chem. Rec. 2018, 18, 1314–1340. 10.1002/tcr.201700098. 29517841

[ref9] Li J.; Luo Y.; Cheo H. W.; Lan Y.; Wu J. Photoredox-Catalysis-Modulated Nickel-Catalyzed Divergent Difunctionalization of Ethylene. Chem 2019, 5, 192–203. 10.1016/j.chempr.2018.10.006.

[ref10] Derosa J.; Apolinar O.; Kang T.; Tran V. T.; Engle K. M. Recent developments in nickel-catalyzed intermolecular dicarbofunctionalization of alkenes. Chem. Sci. 2020, 11, 4287–4296. 10.1039/C9SC06006E. 34122886 PMC8152638

[ref11] Macarron R.; Banks M. N.; Bojanic D.; Burns D. J.; Cirovic D. A.; Garyantes T.; Green D. V. S.; Hertzberg R. P.; Janzen W. P.; Paslay J. W.; Schopfer U.; Sittampalam G. S. Impact of high-throughput screening in biomedical research. Nat. Rev. Drug Discovery 2011, 10, 188–195. 10.1038/nrd3368. 21358738

[ref12] O’Connor C. J.; Beckmann H. S. G.; Spring D. R. Diversity-oriented synthesis: producing chemical tools for dissecting biology. Chem. Soc. Rev. 2012, 41, 4444–4456. 10.1039/c2cs35023h. 22491328

[ref13] Kim J.; Kim H.; Park S. B. Privileged Structures: Efficient Chemical “Navigators” toward Unexplored Biologically Relevant Chemical Spaces. J. Am. Chem. Soc. 2014, 136, 14629–14638. 10.1021/ja508343a. 25310802

[ref14] Beller M.; Seayad J.; Tillack A.; Jiao H. Catalytic Markovnikov and anti-Markovnikov Functionalization of Alkenes and Alkynes: Recent Developments and Trends. Angew. Chem., Int. Ed. 2004, 43, 3368–3398. 10.1002/anie.200300616. 15221826

[ref15] Mahatthananchai J.; Dumas A. M.; Bode J. W. Catalytic Selective Synthesis. Angew. Chem., Int. Ed. 2012, 51, 10954–10990. 10.1002/anie.201201787. 23011639

[ref16] Uraguchi D.; Shibazaki R.; Tanaka N.; Yamada K.; Yoshioka K.; Ooi T. Catalyst-Enabled Site-Divergent Stereoselective Michael Reactions: Overriding Intrinsic Reactivity of Enynyl Carbonyl Acceptors. Angew. Chem., Int. Ed. 2018, 57, 4732–4736. 10.1002/anie.201800057. 29436085

[ref17] Beletskaya I. P.; Nájera C.; Yus M. Chemodivergent reactions. Chem. Soc. Rev. 2020, 49, 7101–7166. 10.1039/D0CS00125B. 32926052

[ref18] Chintawar C. C.; Yadav A. K.; Kumar A.; Sancheti S. P.; Patil N. T. Divergent Gold Catalysis: Unlocking Molecular Diversity through Catalyst Control. Chem. Rev. 2021, 121, 8478–8558. 10.1021/acs.chemrev.0c00903. 33555193

[ref19] Ke Y.; Li W.; Liu W.; Kong W. Ni-catalyzed ligand-controlled divergent and selective synthesis. Sci. China: Chem. 2023, 66, 2951–2976. 10.1007/s11426-023-1533-y.

[ref20] Wang L.-T.; Zhou B.; Liu F.-L.; Wei W.-T.; Ye L.-W. Radical strategies for chemodivergent cyclization reactions. Trends Chem. 2023, 5, 906–919. 10.1016/j.trechm.2023.10.002.

[ref21] Wang Y.; Feng J.; Li E.-Q.; Jia Z.; Loh T.-P. Recent advances in ligand-enabled palladium-catalyzed divergent synthesis. Org. Biomol. Chem. 2023, 22, 37–54. 10.1039/D3OB01679J. 38050418

[ref22] Xu T.; Sha F.; Alper H. Highly Ligand-Controlled Regioselective Pd-Catalyzed Aminocarbonylation of Styrenes with Aminophenols. J. Am. Chem. Soc. 2016, 138, 6629–6635. 10.1021/jacs.6b03161. 27159663

[ref23] Hu Y.-C.; Ji D.-W.; Zhao C.-Y.; Zheng H.; Chen Q.-A. Catalytic Prenylation and Reverse Prenylation of Indoles with Isoprene: Regioselectivity Manipulation through Choice of Metal Hydride. Angew. Chem., Int. Ed. 2019, 58, 5438–5442. 10.1002/anie.201901025. 30748079

[ref24] Kuai C.-S.; Ji D.-W.; Zhao C.-Y.; Liu H.; Hu Y.-C.; Chen Q.-A. Ligand-Regulated Regiodivergent Hydrosilylation of Isoprene under Iron Catalysis. Angew. Chem., Int. Ed. 2020, 59, 19115–19120. 10.1002/anie.202007930. 32619282

[ref25] Chen Y.; Zhu K.; Huang Q.; Lu Y. Regiodivergent sulfonylarylation of 1,3-enynes via nickel/photoredox dual catalysis. Chem. Sci. 2021, 12, 13564–13571. 10.1039/D1SC04320J. 34777776 PMC8528021

[ref26] Wu F.-P.; Wu X.-F. Ligand-Controlled Copper-Catalyzed Regiodivergent Carbonylative Synthesis of α-Amino Ketones and α-Boryl Amides from Imines and Alkyl Iodides. Angew. Chem., Int. Ed. 2021, 60, 695–700. 10.1002/anie.202012251. 32991025

[ref27] Kuai C.-S.; Teng B.-H.; Zhao Y.; Wu X.-F. Activator-regulated chemodivergent deoxygenative- and alkoxy-carbonylation of alcohols with boronic acids. J. Catal. 2023, 425, 196–202. 10.1016/j.jcat.2023.06.012.

[ref28] Wu F.; Wang B.; Li N.-Q.; Yang H.-Y.; Ren Z.-H.; Guan Z.-H. Palladium-catalyzed regiodivergent hydrochlorocarbonylation of alkenes for formation of acid chlorides. Nat. Commun. 2023, 14 (1), 3167 10.1038/s41467-023-38748-3. 37258529 PMC10232548

[ref29] Zhang W.-S.; Ji D.-W.; Li Y.; Zhang X.-X.; Mei Y.-K.; Chen B.-Z.; Chen Q.-A. Nickel-catalyzed divergent Mizoroki–Heck reaction of 1,3-dienes. Nat. Commun. 2023, 14, 651 10.1038/s41467-023-36237-1. 36746964 PMC9902549

[ref30] Gu X.-W.; Zhao Y.-H.; Wu X.-F. Ligand-controlled regiodivergent aminocarbonylation of cyclobutanols toward 1,1- and 1,2-substituted cyclobutanecarboxamides. Nat. Commun. 2024, 15 (1), 9412 10.1038/s41467-024-53571-0. 39482305 PMC11528034

[ref31] Jeong J.; Cao S.; Kang H.-J.; Yoon H.; Lee J.; Shin S.; Kim D.; Hong S. Divergent Enantioselective Access to Diverse Chiral Compounds from Bicyclo[1.1.0]butanes and α,β-Unsaturated Ketones under Catalyst Control. J. Am. Chem. Soc. 2024, 146, 27830–27842. 10.1021/jacs.4c10153. 39348293

[ref32] Wang F.; Wang D.; Zhou Y.; Liang L.; Lu R.; Chen P.; Lin Z.; Liu G. Divergent Synthesis of CF3-Substituted Allenyl Nitriles by Ligand-Controlled Radical 1,2- and 1,4-Addition to 1,3-Enynes. Angew. Chem. Int. Ed. 2018, 57, 7140–7145. 10.1002/anie.201803668. 29667331

[ref33] Kuang Z.; Chen H.; Qiu J.; Ou Z.; Lan Y.; Song Q. Cu-Catalyzed Regio- and Stereodivergent Chemoselective sp2/sp3 1,3- and 1,4-Diborylations of CF3-Containing 1,3-Enynes. Chem 2020, 6, 2347–2363. 10.1016/j.chempr.2020.06.034.

[ref34] Jiang W.-S.; Ji D.-W.; Zhang W.-S.; Zhang G.; Min X.-T.; Hu Y.-C.; Jiang X.-L.; Chen Q.-A. Orthogonal Regulation of Nucleophilic and Electrophilic Sites in Pd-Catalyzed Regiodivergent Couplings between Indazoles and Isoprene. Angew. Chem. Int. Ed. 2021, 60, 8321–8328. 10.1002/anie.202100137. 33463001

[ref35] Jin S.; Li S.-J.; Ma X.; Su J.; Chen H.; Lan Y.; Song Q. Elemental-Sulfur-Enabled Divergent Synthesis of Disulfides, Diselenides, and Polythiophenes from β-CF3–1,3-Enynes. Angew. Chem. Int. Ed. 2021, 60, 881–888. 10.1002/anie.202009194. 32985082

[ref36] Zhao C.-Y.; Ji D.-W.; Zheng H.; He G.-C.; Liu H.; Hu Y.-C.; Chen Q.-A. Pd-Catalyzed Redox Divergent Coupling of Ketones with Terpenols. ACS Catal. 2021, 11, 6825–6834. 10.1021/acscatal.1c01488.

[ref37] Wang X.-C.; Li B.; Ju C.-W.; Zhao D. Nickel(0)-catalyzed divergent reactions of silacyclobutanes with internal alkynes. Nat. Commun. 2022, 13 (1), 3392 10.1038/s41467-022-31006-y. 35697690 PMC9192776

[ref38] Wang Z.-C.; Zhang J.-W.; Koh M. J.; Shi S.-L. Divergent and Selective Light Alkene Cross-Coupling. Angew. Chem. Int. Ed. 2023, 62 (45), e202310203 10.1002/anie.202310203. 37786301

[ref39] Wang H.; Jie X.; Chong Q.; Meng F. Pathway-divergent coupling of 1,3-enynes with acrylates through cascade cobalt catalysis. Nat. Commun. 2024, 15 (1), 3427 10.1038/s41467-024-47719-1. 38654019 PMC11039462

[ref40] Schreiber S. L. Target-Oriented and Diversity-Oriented Organic Synthesis in Drug Discovery. Science 2000, 287, 1964–1969. 10.1126/science.287.5460.1964. 10720315

[ref41] Burke M. D.; Schreiber S. L. A Planning Strategy for Diversity-Oriented Synthesis. Angew. Chem. Int. Ed. 2004, 43, 46–58. 10.1002/anie.200300626. 14694470

[ref42] Galloway W. R. J. D.; Isidro-Llobet A.; Spring D. R. Diversity-oriented synthesis as a tool for the discovery of novel biologically active small molecules. Nat. Commun. 2010, 1, 80 10.1038/ncomms1081. 20865796

[ref43] Komanduri V.; Krische M. J. Enantioselective Reductive Coupling of 1,3-Enynes to Heterocyclic Aromatic Aldehydes and Ketones via Rhodium-Catalyzed Asymmetric Hydrogenation: Mechanistic Insight into the Role of Brønsted Acid Additives. J. Am. Chem. Soc. 2006, 128, 16448–16449. 10.1021/ja0673027. 17177363

[ref44] Cheng J.-K.; Loh T.-P. Copper- and Cobalt-Catalyzed Direct Coupling of sp3 α-Carbon of Alcohols with Alkenes and Hydroperoxides. J. Am. Chem. Soc. 2015, 137, 42–45. 10.1021/ja510635k. 25541811

[ref45] Yang Y.; Perry I. B.; Lu G.; Liu P.; Buchwald S. L. Copper-catalyzed asymmetric addition of olefin-derived nucleophiles to ketones. Science 2016, 353, 144–150. 10.1126/science.aaf7720. 27284169 PMC5062742

[ref46] Adamson N. J.; Jeddi H.; Malcolmson S. J. Preparation of Chiral Allenes through Pd-Catalyzed Intermolecular Hydroamination of Conjugated Enynes: Enantioselective Synthesis Enabled by Catalyst Design. J. Am. Chem. Soc. 2019, 141, 8574–8583. 10.1021/jacs.9b02637. 31070902 PMC6568270

[ref47] Zhang K.-F.; Bian K.-J.; Li C.; Sheng J.; Li Y.; Wang X.-S. Nickel-Catalyzed Carbofluoroalkylation of 1,3-Enynes to Access Structurally Diverse Fluoroalkylated Allenes. Angew. Chem. Int. Ed. 2019, 58, 5069–5074. 10.1002/anie.201813184. 30773793

[ref48] Dherbassy Q.; Manna S.; Talbot F. J. T.; Prasitwatcharakorn W.; Perry G. J. P.; Procter D. J. Copper-catalyzed functionalization of enynes. Chem. Sci. 2020, 11, 11380–11393. 10.1039/D0SC04012F. 34094380 PMC8163025

[ref49] Manna S.; Dherbassy Q.; Perry G. J. P.; Procter D. J. Enantio- and Diastereoselective Synthesis of Homopropargyl Amines by Copper-Catalyzed Coupling of Imines, 1,3-Enynes, and Diborons. Angew. Chem. Int. Ed. 2020, 59, 4879–4882. 10.1002/anie.201915191. PMC7383811 31917893

[ref50] Liu W.; Liu C.; Wang M.; Kong W. Modular Synthesis of Multifunctionalized CF3-Allenes through Selective Activation of Saturated Hydrocarbons. ACS Catal. 2022, 12, 10207–10221. 10.1021/acscatal.2c01521.

[ref51] Zhang F.-H.; Guo X.; Zeng X.; Wang Z. Asymmetric 1,4-functionalization of 1,3-enynes via dual photoredox and chromium catalysis. Nat. Commun. 2022, 13 (1), 5036 10.1038/s41467-022-32614-4. 36028488 PMC9418150

[ref52] Chen H.; Zhu C.; Yue H.; Rueping M. Group 14 Elements Hetero-Difunctionalizations via Nickel-Catalyzed Electroreductive Cross-Coupling. Angew. Chem. Int. Ed. 2023, 62 (33), e202306498 10.1002/anie.202306498. 37309588

[ref53] Wang Z.-L.; Li Q.; Yang M.-W.; Song Z.-X.; Xiao Z.-Y.; Ma W.-W.; Zhao J.-B.; Xu Y.-H. Regio- and enantioselective CuH-catalyzed 1,2- and 1,4-hydrosilylation of 1,3-enynes. Nat. Commun. 2023, 14 (1), 5048 10.1038/s41467-023-40703-1. 37598226 PMC10439940

[ref54] Hossain A.; Anderson R. L.; Zhang C. S.; Chen P.-J.; Fu G. C. Nickel-Catalyzed Enantioconvergent and Diastereoselective Allenylation of Alkyl Electrophiles: Simultaneous Control of Central and Axial Chirality. J. Am. Chem. Soc. 2024, 146, 7173–7177. 10.1021/jacs.4c00593. 38447585 PMC11003353

[ref55] Shan Q.-C.; Zhao Y.; Wang S.-T.; Liu H.-F.; Duan X.-H.; Guo L.-N. Nickel-Catalyzed Modular Four-Component 1,4-Alkylcarbonylation of 1,3-Enynes to Tetra-Substituted CF 3 −Allenyl Ketones. ACS Catal. 2024, 14, 2144–2150. 10.1021/acscatal.3c05776.

[ref56] Pagar V. V.; RajanBabu T. V. Tandem catalysis for asymmetric coupling of ethylene and enynes to functionalized cyclobutanes. Science 2018, 361, 68–72. 10.1126/science.aat6205. 29976822 PMC6055924

[ref57] Zhou Y.; Zhou L.; Jesikiewicz L. T.; Liu P.; Buchwald S. L. Synthesis of Pyrroles through the CuH-Catalyzed Coupling of Enynes and Nitriles. J. Am. Chem. Soc. 2020, 142, 9908–9914. 10.1021/jacs.0c03859. 32395998 PMC8009196

[ref58] Li L.; Wang S.; Luo P.; Wang R.; Wang Z.; Li X.; Deng Y.; Peng F.; Shao Z. Direct access to spirocycles by Pd/WingPhos-catalyzed enantioselective cycloaddition of 1,3-enynes. Nat. Commun. 2021, 12 (1), 5667 10.1038/s41467-021-25981-x. 34580311 PMC8476582

[ref59] Li Q.; Fang X.; Pan R.; Yao H.; Lin A. Palladium-Catalyzed Asymmetric Sequential Hydroamination of 1,3-Enynes: Enantioselective Syntheses of Chiral Imidazolidinones. J. Am. Chem. Soc. 2022, 144, 11364–11376. 10.1021/jacs.2c03620. 35687857

[ref60] Lu W.; Zhao Y.; Meng F. Cobalt-Catalyzed Sequential Site- and Stereoselective Hydrosilylation of 1,3- and 1,4-Enynes. J. Am. Chem. Soc. 2022, 144, 5233–5240. 10.1021/jacs.2c00288. 35298144

[ref61] Xie B.-Y.; He Z.-T. Chemodivergent Tandem Hydroalkylation and Hydroalkenoxylation of Conjugated Enynes. ACS Catal. 2024, 14, 9742–9751. 10.1021/acscatal.4c02377.

[ref62] aPeng J.-B.; Geng H.-Q.; Wu X.-F. The Chemistry of CO: Carbonylation. Chem 2019, 5, 526–552. 10.1016/j.chempr.2018.11.006.

[ref63] Carbon Monoxide in Organic Synthesis-Carbonylation Chemistry, Gabriele B., Eds.; Wiley-VCH, 2022.

[ref64] Zhang S.; Neumann H.; Beller M. Synthesis of α,β-unsaturated carbonyl compounds by carbonylation reactions. Chem. Soc. Rev. 2020, 49, 3187–3210. 10.1039/C9CS00615J. 32255444

[ref65] Liu Y.; Chen Y.-H.; Yi H.; Lei A. An Update on Oxidative C–H Carbonylation with CO. ACS Catal. 2022, 12, 7470–7485. 10.1021/acscatal.2c01639.

[ref66] aKuai C.-S.; Teng B.-H.; An D.-L.; Zhao Y.; Wu X.-F. Palladium-Catalyzed Regioselective Carbonylation of 2-Trifluoromethyl-1,3-enynes to Multisubstituted Conjugated Dienes. Org. Lett. 2023, 25, 682–687. 10.1021/acs.orglett.2c04331. 36656103

[ref67] aHussain S. M. S.; Suleiman R.; Ali B. E. New conjugated dienamides via palladium-catalyzed selective aminocarbonylation of enynes. Tetrahedron Lett. 2012, 53, 6535–6539. 10.1016/j.tetlet.2012.09.084.

[ref68] Cernak T.; Dykstra K. D.; Tyagarajan S.; Vachal P.; Krska S. W. The medicinal chemist’s toolbox for late stage functionalization of drug-like molecules. Chem. Soc. Rev. 2016, 45, 546–576. 10.1039/C5CS00628G. 26507237

[ref69] Hong B.; Luo T.; Lei X. Late-Stage Diversification of Natural Products. ACS Cent. Sci. 2020, 6, 622–635. 10.1021/acscentsci.9b00916. 32490181 PMC7256965

[ref70] Hamadi N. B.; Msaddek M. Regio- and Stereoselectivity in 1,3-dipolar Cycloaddition Reaction of 2-diazopropane with Benzylidene-N-arylsuccinimide and Benzylidene-N-arylmethylsuccinimide Derivatives: Synthesis of Gem-dimethylcyclopropane. J. Chem. Res. 2007, 2007, 121–123. 10.3184/030823407X191868.

[ref71] Banerjee D.; Junge K.; Beller M. A General Catalytic Hydroamidation of 1,3-Dienes: Atom-Efficient Synthesis of N-Allyl Heterocycles, Amides, and Sulfonamides. Angew. Chem. Int. Ed. 2014, 53, 1630–1635. 10.1002/anie.201308874. 24452993

[ref72] Huang L.; Arndt M.; Gooßen K.; Heydt H.; Gooßen L. J. Late Transition Metal-Catalyzed Hydroamination and Hydroamidation. Chem. Rev. 2015, 115, 2596–2697. 10.1021/cr300389u. 25721762

[ref73] Eldred S. E.; Stone D. A.; Gellman S. H.; Stahl S. S. Catalytic Transamidation under Moderate Conditions. J. Am. Chem. Soc. 2003, 125, 3422–3423. 10.1021/ja028242h. 12643691

[ref74] Dander J. E.; Garg N. K. Breaking Amides using Nickel Catalysis. ACS Catal. 2017, 7, 1413–1423. 10.1021/acscatal.6b03277. 28626599 PMC5473294

[ref75] Wu X.-F.; Fang X.; Wu L.; Jackstell R.; Neumann H.; Beller M. Transition-Metal-Catalyzed Carbonylation Reactions of Olefins and Alkynes: A Personal Account. Acc. Chem. Res. 2014, 47, 1041–1053. 10.1021/ar400222k. 24564478

[ref76] Xu J.-X.; Wu X.-F. Palladium-Catalyzed Carbonylative Cyclization of Terminal Alkynes and Anilines to 3-Substituted Maleimides. Adv. Synth. Catal. 2018, 360, 3376–3380. 10.1002/adsc.201800672.

[ref77] Yang J.; Liu J.; Jackstell R.; Beller M. Palladium-catalyzed aerobic oxidative carbonylation of alkynes with amines: a general access to substituted maleimides. Chem. Commun. 2018, 54, 10710–10713. 10.1039/C8CC05802D. 30182114

[ref78] Ai H.-J.; Lu W.; Wu X.-F. Ligand-Controlled Regiodivergent Thiocarbonylation of Alkynes toward Linear and Branched α,β-Unsaturated Thioesters. Angew. Chem. Int. Ed. 2021, 60, 17178–17184. 10.1002/anie.202106079. 34058046

[ref79] Cai S.; Zhang H.; Huang H. Transition-Metal-Catalyzed Hydroaminocarbonylations of Alkenes and Alkynes. Trends Chem. 2021, 3, 218–230. 10.1016/j.trechm.2020.11.006.

