# Supplementary material for: Multimodal Precise Control Over Multiselective Carbonylation of 1,3-Enynes
Source: J Am Chem Soc. 2025 Feb 19;147(9):7950–64. doi: 10.1021/jacs.5c00032 (PMC11887062; doi:10.1021/jacs.5c00032)
Supplement: Supplementary file 1 — ja5c00032_si_001.pdf [file ja5c00032_si_001.pdf]

Supporting Information

Multimodal Precise Control over Multi-Selective Carbonylation of 1,3-Enynes

Chang-Sheng Kuai,[1,2] Yuanrui Wang,[1,2] Ting Yang,[1] Xiao-Feng Wu*[1,2,3]

1. Dalian National Laboratory for Clean Energy, Dalian Institute of Chemical Physics, Chinese Academy of Sciences, 116023 Dalian, Liaoning, China. *E-mail: xwu2020@dicp.ac.cn
2. University of Chinese Academy of Sciences, Beijing 100049, China.
3 Leibniz-Institut für Katalyse e. V., Albert-Einstein-Straβe 29a, 18059 Rostock, Germany

Table of Contents

1. General experimental details S2
2. General procedure for Pd-catalyzed divergent carbonylation of 1,3-enynes S3
3. Synthetic applications S5
4. Kinetic studies. S8
5. Isotopic labeling studies S11
6. Spectroscopic Data of Products S21
7. NMR Spectra of the Products S64
8. Mercury plot, crystal data and refinement results S231

1. General experimental details

Unless otherwise noted, all reactions were carried out under a carbon monoxide or nitrogen atmosphere. The amines and reagents were ordered from Adamas-beta®, Energy Chemical Sigma-Aldrich, Bidepharm and used without purification. All solvents were dried by standard techniques and distilled prior to use. Column chromatography was performed on silica gel (200-300 meshes). All NMR spectra were recorded at ambient temperature using Bruker Avance III 400 MHz NMR (^{1}H, 400 MHz; ^{13}C {^{1}H}, 101 MHz, ^{19}F 376 MHz), Bruker AVANCE III HD 700MHz NMR spectrometers (^{1}H, 700 MHz; ^{13}C{^{1}H}, 100 MHz). ^{1}H NMR chemical shifts are reported relative to TMS and were referenced via residual proton resonances of the corresponding deuterated solvent ($CDCl_3$: 7.26 ppm) whereas ^{13}C{^{1}H} NMR spectra are reported relative to TMS via the carbon signals of the deuterated solvent ($CDCl_3$: 77.0 ppm). Data for ^{1}H are reported as follows: chemical shift (δ ppm), multiplicity (s = singlet, d = doublet, t = triplet, q = quartet, quint = quintet, m = multiplet, br = broad), coupling constant (Hz), and integration. All ^{13}C NMR spectra were broad-band ^{1}H decoupled. All reactions were monitored by GC-FID or NMR analysis. HRMS data was obtained with Micromass HPLC-Q-TOF mass spectrometer (ESI) or Agilent 6540 Accurate-MS spectrometer (Q-TOF).

Because of the high toxicity of carbon monoxide, all the reactions should be performed in an autoclave. The laboratory should be well-equipped with a CO detector and alarm system.

2. General procedure for Pd-catalyzed divergent carbonylation of 1,3-enynes

2.1 General procedure for Pd-catalyzed 2,4-tandem cyclocarbonylation.

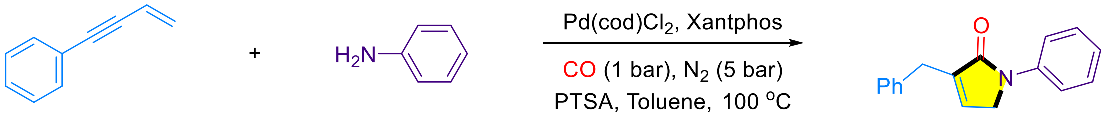

1a **2a** **5a**

A 4 mL screw-cap vial was charged with Pd(cod)Cl_2 (5 mol%), xantphos (5 mol%), PTSA (10 mol%) and an oven-dried stir bar. The vial was closed with a Teflon septum and cap and connected to the atmosphere via a needle. After toluene (1.0 mL), 1,3-enyne (**1a**, 0.2 mmol), aniline (**2a**, 0.3 mmol), were added with a syringe under argon atmosphere, the vial was moved to an alloy plate and put into a Parr 4560 series autoclave (300 mL) under an argon atmosphere. At room temperature, the autoclave was flushed two times with nitrogen (~ 5 bar), and charged with 1 bar of CO and 5 bar of N_2. The autoclave was placed on a heating plate equipped with a magnetic stirrer and an aluminum block. The reaction mixture was heated to 100 °C for 16 h. After the reaction was complete, the autoclave was cooled down with ice water to room temperature and the pressure was released carefully. The reaction mixture concentration under reduced pressure, the crude product was purified by column chromatography on silica gel to afford the corresponding product **5a**.

2.2 General procedure for Pd-catalyzed 2,3-tandem cyclo-dicarbonylation.

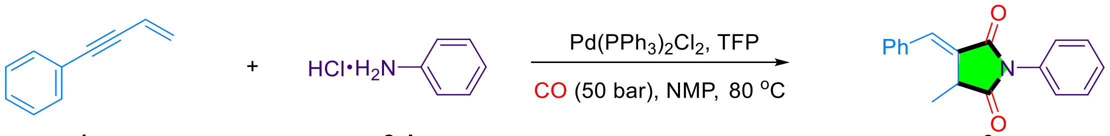

1a **2a'** **6a**

A 4 mL screw-cap vial was charged with $Pd(PPh_3)_2Cl_2$ (5 mol%), TFP (10 mol%) and an oven-dried stir bar. The vial was closed with a Teflon septum and cap and connected to the atmosphere via a needle. After NMP (1.0 mL), 1,3-enyne (**1a**, 0.2 mmol), aniline hydrochloride (**2a'**, 0.3 mmol), were added with a syringe under argon atmosphere, the vial was moved to an alloy plate and put into a Parr 4560 series autoclave (300 mL) under an argon atmosphere. At room temperature, the autoclave was flushed two times with nitrogen (~ 5 bar), and charged with 50 bar of CO. The autoclave was placed on a heating plate equipped with a magnetic stirrer and an aluminum block. The reaction mixture was heated to 80 °C for 20 h. After the reaction was complete, the autoclave was cooled down with ice water to room temperature and the pressure was released carefully. The reaction mixture concentration under reduced pressure, the crude product was purified by column chromatography on silica gel to afford the corresponding product **6a**.

2.3 General procedure for Pd-catalyzed 1,3-tandem cyclocarbonylation.

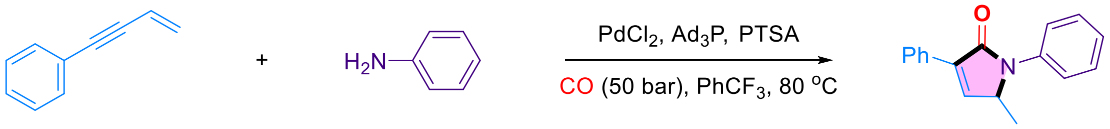

1a **2a** **7a**

A 4 mL screw-cap vial was charged with $PdCl_2$ (5 mol%), PAd_3 (15 mol%), PTSA (10 mol%) and an oven-dried stir bar. The vial was closed with a Teflon septum and cap and connected to the atmosphere via a needle. After $PhCF_3$ (1.0 mL), 1,3-enyne (**1a**, 0.10 mmol), aniline (**2a**, 0.15 mmol),

were added with a syringe under argon atmosphere, the vial was moved to an alloy plate and put into a Parr 4560 series autoclave (300 mL) under an argon atmosphere. At room temperature, the autoclave was flushed two times with nitrogen (~ 5 bar), and charged with 10 bar of CO. The autoclave was placed on a heating plate equipped with a magnetic stirrer and an aluminum block. The reaction mixture was heated to 80 °C for 48 h. After the reaction was complete, the autoclave was cooled down with ice water to room temperature and the pressure was released carefully. The reaction mixture concentration under reduced pressure, the crude product was purified by column chromatography on silica gel to afford the corresponding product **7a**.

2.4 General procedure for Pd-catalyzed 1,2-hydroaminocarbonylation.

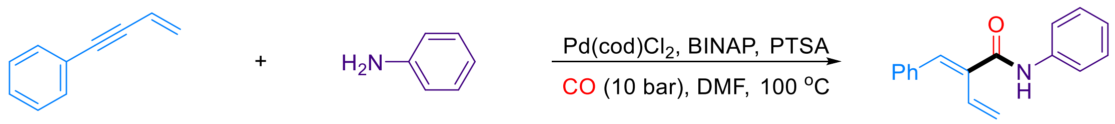

1a **2a** **3a**

A 4 mL screw-cap vial was charged with Pd(cod)Cl_2 (5 mol%), BINAP (5 mol%), PTSA (10 mol%) and an oven-dried stir bar. The vial was closed with a Teflon septum and cap and connected to the atmosphere via a needle. After DMF (1.0 mL), 1,3-enyne (**1a**, 0.10 mmol), aniline (**2a**, 0.15 mmol), were added with a syringe under argon atmosphere, the vial was moved to an alloy plate and put into a Parr 4560 series autoclave (300 mL) under an argon atmosphere. At room temperature, the autoclave was flushed two times with nitrogen (~ 5 bar), and charged with 10 bar of CO. The autoclave was placed on a heating plate equipped with a magnetic stirrer and an aluminum block. The reaction mixture was heated to 100 °C for 16 h. After the reaction was complete, the autoclave was cooled down with ice water to room temperature and the pressure was released carefully. The reaction mixture concentration under reduced pressure, the crude product was purified by column chromatography on silica gel to afford the corresponding product **3a**.

2.4 General procedure for Pd-catalyzed 2,1-hydroaminocarbonylation.

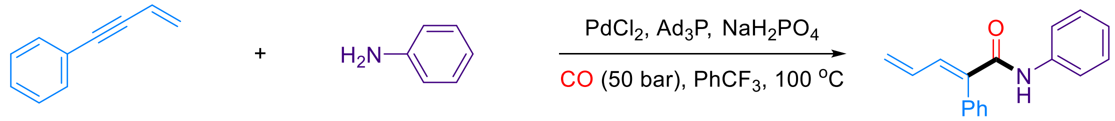

1a **2a** **4a**

A 4 mL screw-cap vial was charged with $PdCl_2$ (5 mol%), PAd_3 (15 mol%), NaH_2PO_4 (10 mol%) and an oven-dried stir bar. The vial was closed with a Teflon septum and cap and connected to the atmosphere via a needle. After $PhCF_3$ (1.0 mL) and dioxane (0.5 mL), 1,3-enyne (**1a**, 0.10 mmol), aniline (**2a**, 0.15 mmol), were added with a syringe under argon atmosphere, the vial was moved to an alloy plate and put into a Parr 4560 series autoclave (300 mL) under an argon atmosphere. At room temperature, the autoclave was flushed two times with nitrogen (~ 5 bar), and charged with 10 bar of CO. The autoclave was placed on a heating plate equipped with a magnetic stirrer and an aluminum block. The reaction mixture was heated to 100 °C for 2 h. After the reaction was complete, the autoclave was cooled down with ice water to room temperature and the pressure was released carefully. The reaction mixture concentration under reduced pressure, the crude product was purified by column chromatography on silica gel to afford the corresponding product **4a**.

3. Synthetic applications.

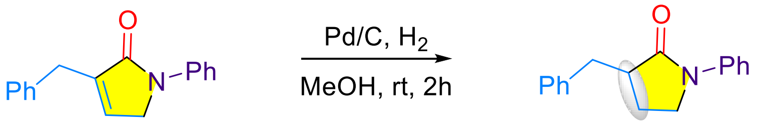

5a **5a1**

A Schlenk tube was charged with lactam **5a** (24.9 mg, 0.1 mmol, 1.0 equiv) and Pd/C catalyst (2.5 mg, 10.0 wt% palladium on carbon), and then the reactor was evacuated/filled with H_2 (balloon) for three times. After that, MeOH (0.5 mL) was added and the reaction mixture was stirred under H_2 atmosphere (balloon) for 2 h at room temperature, and monitored by NMR. The reaction mixture was filtered through Celite. The solvent in the filtrate was evaporated under reduced pressure. The residue was purified by flash chromatography to afford the pure saturated lactam **5a1** (24.0 mg, 96%) as a white solid, R_f=0.2 (PE/EtOAc 5/1).

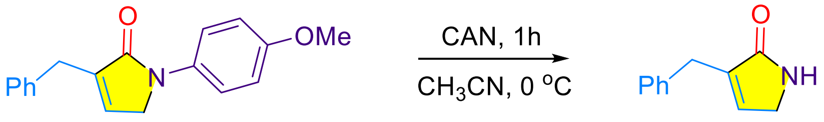

5d **5d1**

The lactam **5d** (27.9 mg, 0.1 mmol, 1.0 equiv) was dissolved into CH_3CN (0.8 mL), and a solution of CAN (140.4 mg, 0.3 mmol) in H_2O (1.2 mL) was added dropwise at 0 °C. The resultant solution was stirred for 1 h at 0 °C. Hereafter, saturated aqueous $NaHCO_3$ was added and the organic components were extracted with CH_2Cl_2 (3 × 15 mL). Upon removal of the organic solvent, the crude product was purified by flash chromatography to afford the deprotected lactam **5d1** (13.5 mg, 78%) as a yellow solid, R_f=0.2 (PE/EtOAc 15/1).

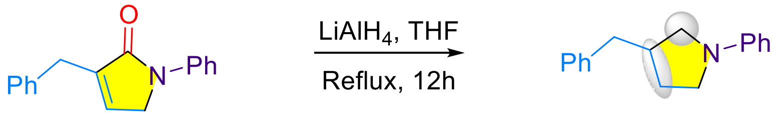

5a **5a2**

To an overdried Schlenk tube was added lactam **5a** (24.9 mg, 0.1 mmol, 1.0 equiv), and the reactor was evacuated/filled with N_2 for three times. After that, $LiAlH_4$ in THF (1.0 M, 0.6 mL, 6.0 equiv) was added and the reaction mixture was heated to reflux for 12 h. The reaction mixture was quenched with 10% NaOH (aq) and heated at reflux for another 1 h. The organic components were extracted with CH_2Cl_2 (3 × 15 mL), the combined organic layers were washed with brine, dried over Na_2SO_4, filtered, and then concentrated under reduced pressure. The residue was purified by flash chromatography to afford the pure, reduced product **5a2** (16.7 mg, 70%) as a brown oil, R_f=0.2 (PE/EtOAc 100/1).

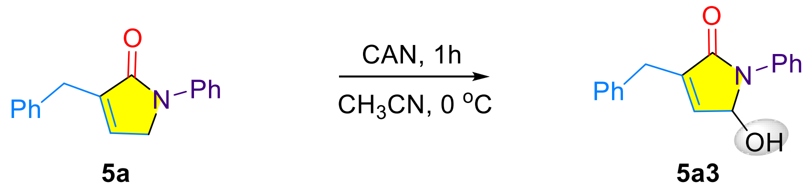

The lactam **5a** (24.9 mg, 0.1 mmol, 1.0 equiv) was dissolved into CH_3CN (0.8 mL), and a solution of CAN (140.4 mg, 0.3 mmol) in H_2O (1.2 mL) was added dropwise at 0 °C. The resultant solution was stirred for 1 h at 0 °C. Hereafter, saturated aqueous $NaHCO_3$ was added and the organic components were extracted with CH_2Cl_2 (3 × 15 mL). Upon removal of the organic solvent, the crude product was purified by flash chromatography to afford the deprotected lactam **5d3** (22.1 mg, 83%) as a white solid, R_f=0.2 (PE/EtOAc 10/1).

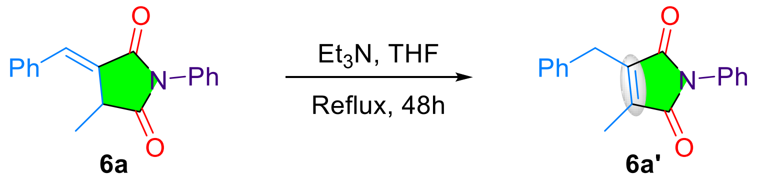

To a stirred solution of **6a** (0.10 mmol, 27.7 mg) in THF (1.0 mL) was added triethylamine (1.0 mL) and the reaction mixture was refluxed for 48 h and then concentrated in vacuo. The residue was dissolved in ethyl acetate and the organic layer was washed with water and brine and dried over Na_2SO_4. Concentration of the organic layer in vacuo followed by silica gel column chromatographic purification of the residue with petroleum ether and ethyl acetate furnished **6a'** in 20.4mg, 74% yields, R_f=0.2 (PE/EtOAc 5/1).

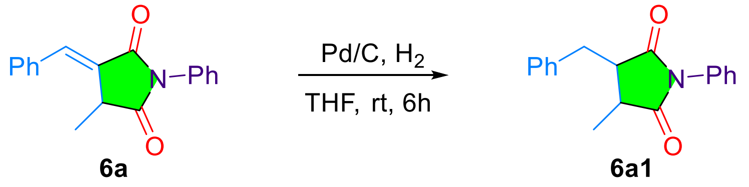

A Schlenk tube was charged with lactam **6a** (27.7 mg, 0.1 mmol, 1.0 equiv) and Pd/C catalyst (2.5 mg, 10.0 wt% palladium on carbon), and then the reactor was evacuated/filled with H_2 (balloon) for three times. After that, THF (0.5 mL) was added and the reaction mixture was stirred under H_2 atmosphere (balloon) for 6 h at room temperature, and monitored by NMR. The reaction mixture was filtered through Celite. The solvent in the filtrate was evaporated under reduced pressure. The residue was purified by flash chromatography to afford the pure saturated lactam **5a1** (23.3 mg, 84%) as a white solid, R_f=0.2 (PE/EtOAc 5/1).

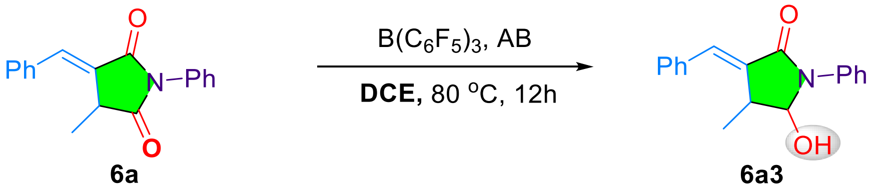

To a pressure tube were sequentially added **6a** (27.7 mg, 0.1 mmol), $B(C_6F_5)_3$ (1.0 mg, 2.0 mol %), NH_3•BH_3 (22.4 mg, 0.8 mmol), and DCE (1.0 mL). Then the reaction mixture was stirred

at 60 °C for 12 h. After cooling to ambient temperature, the mixture was diluted with EtOAc (2.0 mL). Then aqueous NaOH (5.0 mL, 4.0 M) was added to the reaction mixture, which was extracted with EtOAc three times (10.0 mL each). The combined organic phases were dried over Na_2SO_4, then filtered and evaporated under reduced pressure. After the removal of volatile materials by rotary evaporation, the resultant mixture was purified by silica gel column chromatography using a mixture of EtOAc and hexane (2:1) to give the corresponding pure product **6a3** (19.8 mg, 71%).

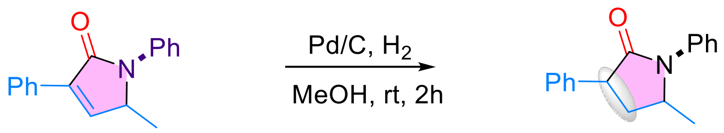

7a **7a1**

A Schlenk tube was charged with lactam **6a** (27.7 mg, 0.1 mmol, 1.0 equiv) and Pd/C catalyst (2.5 mg, 10.0 wt% palladium on carbon), and then the reactor was evacuated/filled with H_2 (balloon) for three times. After that, THF (0.5 mL) was added and the reaction mixture was stirred under H_2 atmosphere (balloon) for 2 h at room temperature, and monitored by NMR. The reaction mixture was filtered through Celite. The solvent in the filtrate was evaporated under reduced pressure. The residue was purified by flash chromatography to afford the pure saturated lactam **5a1** (16.3 mg, 65%) as a white solid, R_f=0.2 (PE/EtOAc 5/1).

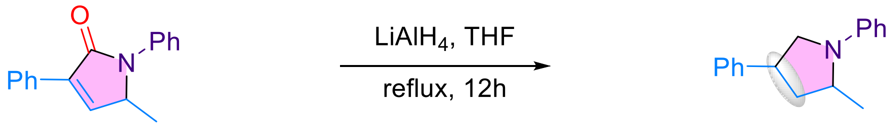

7a **7a2**

To an overdried Schlenk tube was added lactam 7a (24.9 mg, 0.1 mmol, 1.0 equiv), and the reactor was evacuated/filled with N_2 for three times. After that, $LiAlH_4$ in THF (1.0 M, 0.6 mL, 6.0 equiv) was added and the reaction mixture was heated to reflux for 12 h. The reaction mixture was quenched with 10% NaOH (aq) and heated at reflux for another 1 h. The organic components were extracted with CH_2Cl_2 (3 × 15 mL), the combined organic layers were washed with brine, dried over Na_2SO_4, filtered, and then concentrated under reduced pressure. The residue was purified by flash chromatography to afford the pure, reduced product **5a2** (14.5 mg, 61%) as a red solid, R_f=0.2 (PE/EtOAc 100/1).

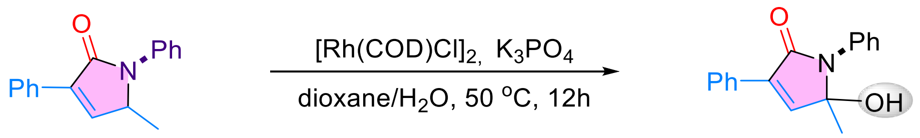

7a **7a3**

The lactam **7a** (24.9 mg, 0.1 mmol, 1.0 equiv), $[Rh(COD)Cl]_2$ (2.5 mg, 2.5 mol%) and K_3PO_4 (31.8 mg, 1.5 equiv) were dissolved into 1,4-dioxane/H_2O (5/1, v/v, 1.0 mL), and then the resultant mixture was stirred for 12 h at 50 °C under air, after which the pure product **7a2** was isolated by flash chromatography R_f=0.2 (PE/EtOAc 5/1), in 15.5 mg, 58%, as a brown oil.

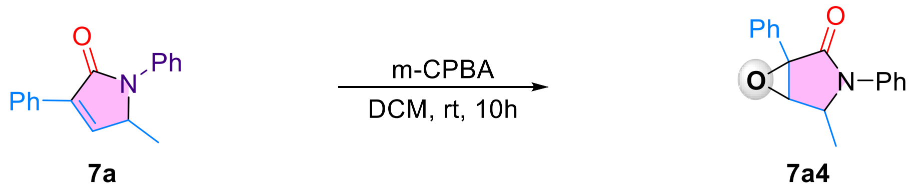

The mixture of *m*-CPBA (3.0 equiv) and **7a** (24.9 mg, 0.1 mmol, 1.0 eq) in DCM (2.0 mL) was stirred at room temperature for 10 h under N_2 atmosphere. The resulting mixture was then quenched with saturated Na_2SO_3 (aq.) and extracted with Et_2O, the combined organic phases were concentrated under vacuum, and the crude was purified by flash column chromatography on silica gel, eluting with ethyl acetate/petroleum ether 1:10 (v/v) to give compound **7a4** (13.9 mg, 52%) as a light-yellow oil.

4. Kinetic studies.

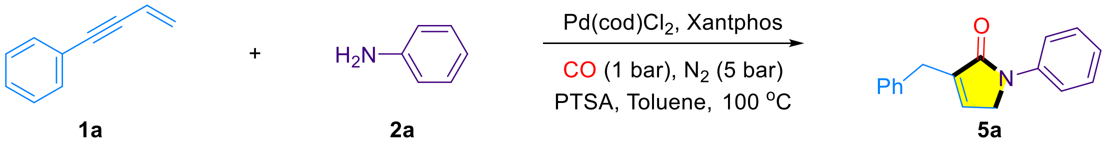

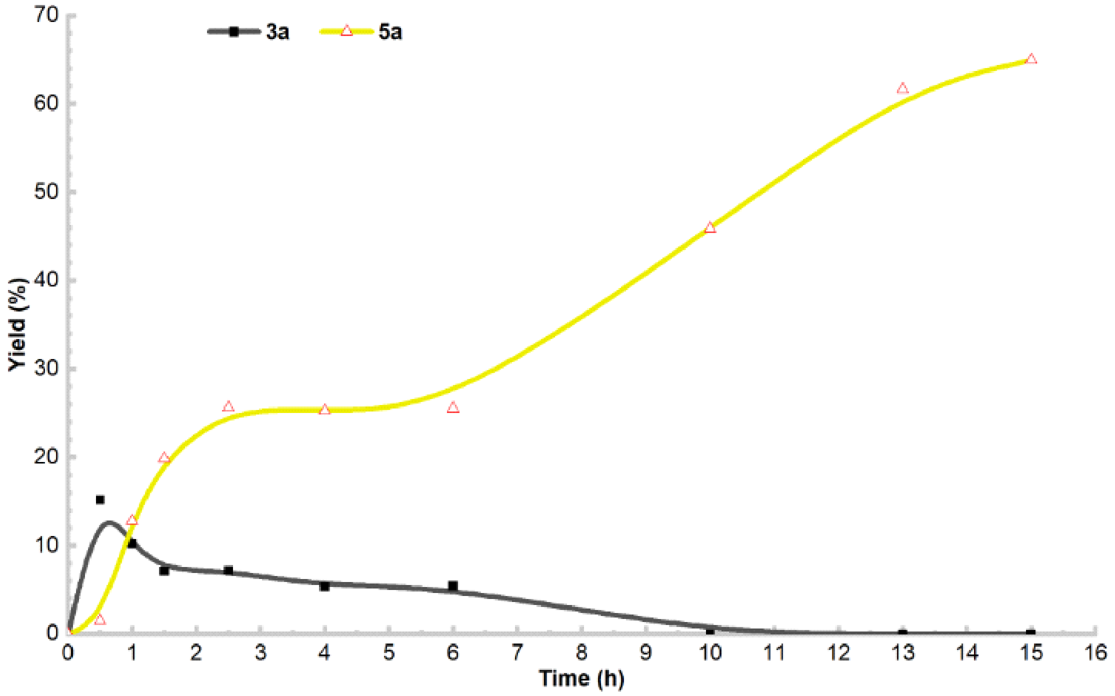

A 4 mL screw-cap vial was charged with Pd(cod)Cl_2 (5 mol%), xantphos (5 mol%), PTSA (10 mol%) and an oven-dried stir bar. The vial was closed with a Teflon septum and cap and connected to the atmosphere via a needle. After toluene (1.0 mL), 1,3-enyne (**1a**, 0.10 mmol), aniline (**2a**, 0.15 mmol), were added with a syringe under argon atmosphere, the vial was moved to an alloy plate and put into a Parr 4560 series autoclave (300 mL) under an argon atmosphere. At room temperature, the autoclave was flushed two times with nitrogen (~ 5 bar), and charged with 1 bar of CO and 5 bar of N_2. The autoclave was placed on a heating plate equipped with a magnetic stirrer and an aluminum block. The reaction mixture was heated to 100 °C. After the reaction was complete, the autoclave was cooled down with ice water to room temperature and the pressure was released carefully. Subsequently, 20 μL of ^{n}hexadecane was added to the reaction mixture as an internal

standard and mixed thoroughly. The yields were determined using GC-FID analysis. The experiments were conducted independently at various reaction times: 0.5 h, 1.5 h, 2.5 h, 4.0 h, 6.0 h, 10.0 h, 13.0 h, and 15.0 h.

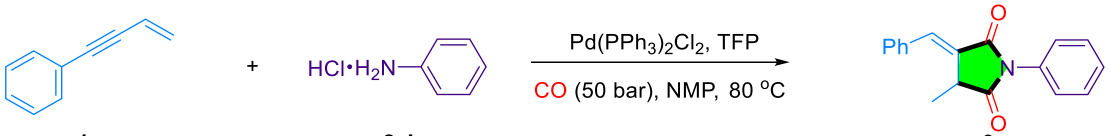

1a **2a'** **6a**

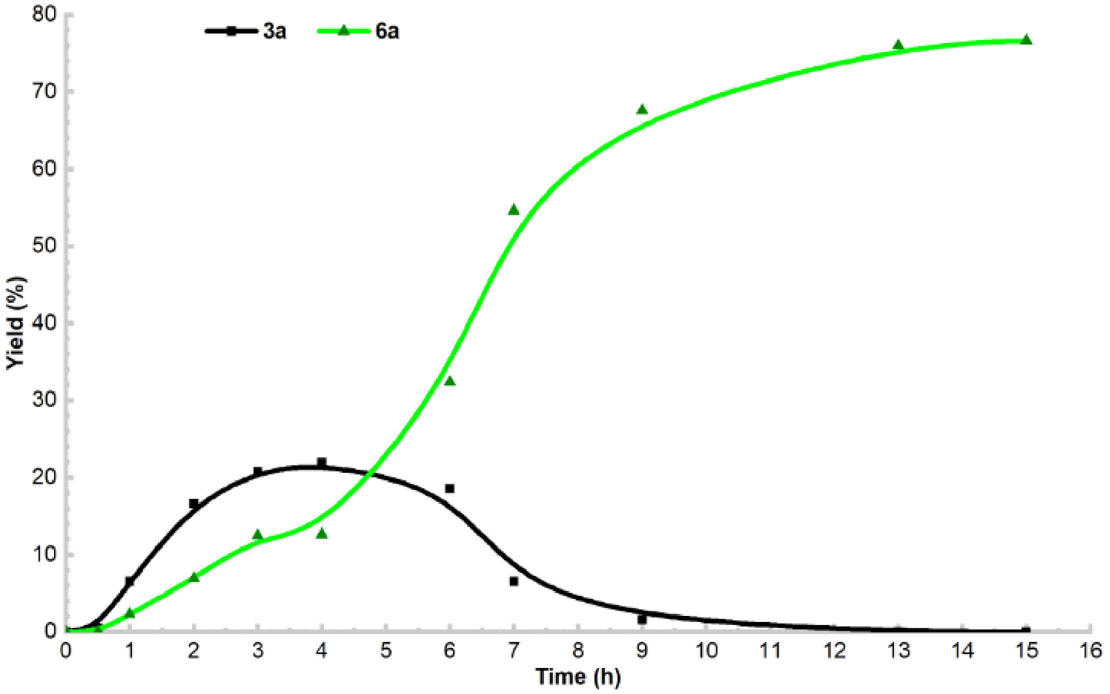

A 4 mL screw-cap vial was charged with $Pd(PPh_3)_2Cl_2$ (5 mol%), TFP (10 mol%) and an oven-dried stir bar. The vial was closed with a Teflon septum and cap and connected to the atmosphere via a needle. After NMP (1.0 mL), 1,3-enyne (**1a**, 0.10 mmol), aniline hydrochloride (**2a'**, 0.15 mmol), were added with a syringe under argon atmosphere, the vial was moved to an alloy plate and put into a Parr 4560 series autoclave (300 mL) under an argon atmosphere. At room temperature, the autoclave was flushed two times with nitrogen (~ 5 bar), and charged with 50 bar of CO. The autoclave was placed on a heating plate equipped with a magnetic stirrer and an aluminum block. The reaction mixture was heated to 80 °C. After the reaction was complete, the autoclave was cooled down with ice water to room temperature and the pressure was released carefully. Subsequently, 20 μL of ^{n}hexadecane was added to the reaction mixture as an internal standard and mixed thoroughly. The yields were determined using GC-FID analysis. The experiments were conducted independently at various reaction times: 0.5 h, 1.0 h, 2.0 h, 3.0 h, 4.0 h, 6.0 h, 7.0 h, 9.0 h, 13.0 h and 15.0 h.

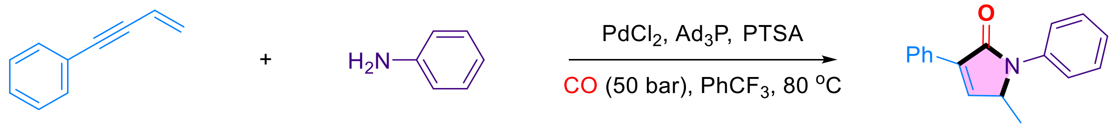

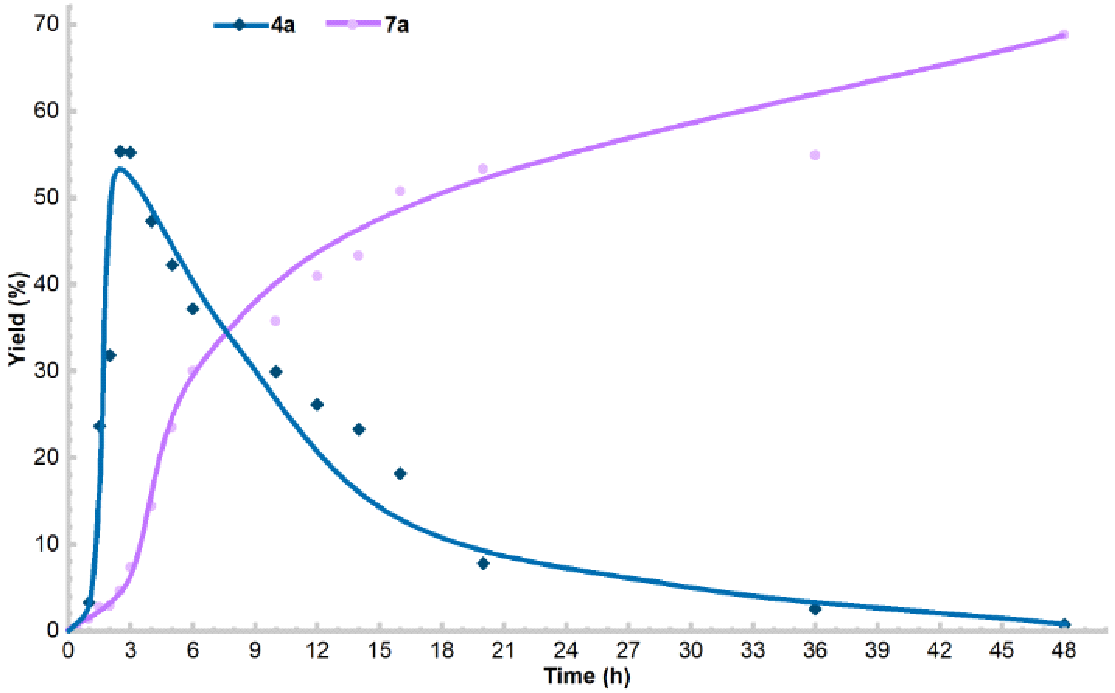

A 4 mL screw-cap vial was charged with $PdCl_2$ (5 mol%), PAd_3 (15 mol%), PTSA (10 mol%) and an oven-dried stir bar. The vial was closed with a Teflon septum and cap and connected to the atmosphere via a needle. After $PhCF_3$ (1.0 mL), 1,3-enyne (**1a**, 0.10 mmol), aniline (**2a**, 0.15 mmol), were added with a syringe under argon atmosphere, the vial was moved to an alloy plate and put into a Parr 4560 series autoclave (300 mL) under an argon atmosphere. At room temperature, the autoclave was flushed two times with nitrogen (~ 5 bar), and charged with 10 bar of CO. The autoclave was placed on a heating plate equipped with a magnetic stirrer and an aluminum block. The reaction mixture was heated to 80 °C. Subsequently, 20 μL of ^{n}hexadecane was added to the reaction mixture as an internal standard and mixed thoroughly. The yields were determined using GC-FID analysis. The experiments were conducted independently at various reaction times: 1.0 h, 1.5, 2.0 h, 2.5, 3.0 h, 4.0 h, 5.0 h, 6.0 h, 10.0 h, 12.0 h, 14.0 h, 16.0 h, 20.0 h, 36.0 h and 48.0 h.

5. Isotopic labeling studies.

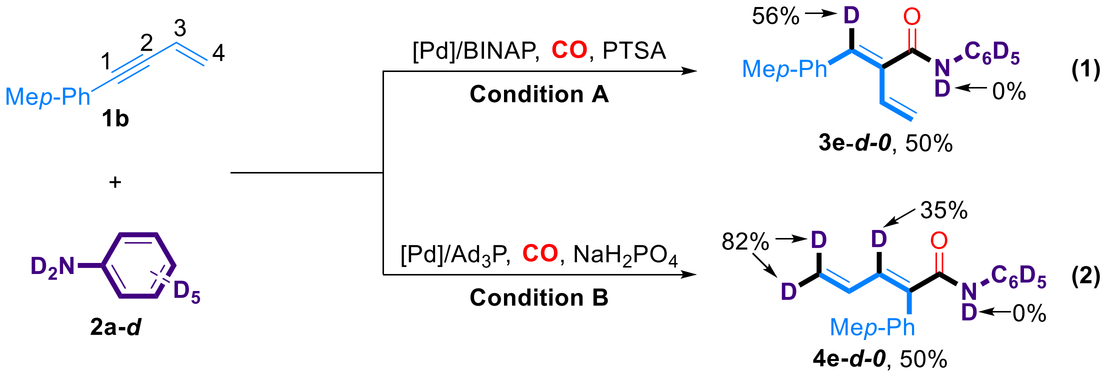

Condition A: A 4 mL screw-cap vial was charged with Pd(cod)Cl_2 (5 mol%), BINAP (5 mol%), PTSA (10 mol%) and an oven-dried stir bar. The vial was closed with a Teflon septum and cap and connected to the atmosphere via a needle. After DMF (1.0 mL), 1,3-enyne (**1b**, 0.10 mmol), aniline-d7 (**2a-*d***, 0.15 mmol), were added with a syringe under argon atmosphere, the vial was moved to an alloy plate and put into a Parr 4560 series autoclave (300 mL) under an argon atmosphere. At room temperature, the autoclave was flushed two times with nitrogen (~ 5 bar), and charged with 10 bar of CO. The autoclave was placed on a heating plate equipped with a magnetic stirrer and an aluminum block. The reaction mixture was heated to 100 °C for 16h. After the reaction was complete, the autoclave was cooled down with ice water to room temperature and the pressure was released carefully. The reaction mixture concentration under reduced pressure, the crude product was purified by column chromatography on silica gel to afford the corresponding product **3e-*d-0***.

Condition A: A 4 mL screw-cap vial was charged with $PdCl_2$ (5 mol%), PAd_3 (15 mol%), NaH_2PO_4 (10 mol%) and an oven-dried stir bar. The vial was closed with a Teflon septum and cap and connected to the atmosphere via a needle. After $PhCF_3$ (1.0 mL), 1,3-enyne (**1a**, 0.10 mmol), aniline-d7 (**2a-*d***, 0.15 mmol), were added with a syringe under argon atmosphere, the vial was moved to an alloy plate and put into a Parr 4560 series autoclave (300 mL) under an argon atmosphere. At room temperature, the autoclave was flushed two times with nitrogen (~ 5 bar), and charged with 10 bar of CO. The autoclave was placed on a heating plate equipped with a magnetic stirrer and an aluminum block. The reaction mixture was heated to 100 °C for 2h. After the reaction was complete, the autoclave was cooled down with ice water to room temperature and the pressure was released carefully. The reaction mixture concentration under reduced pressure, the crude product was purified by column chromatography on silica gel to afford the corresponding product **4e-*d-0***.

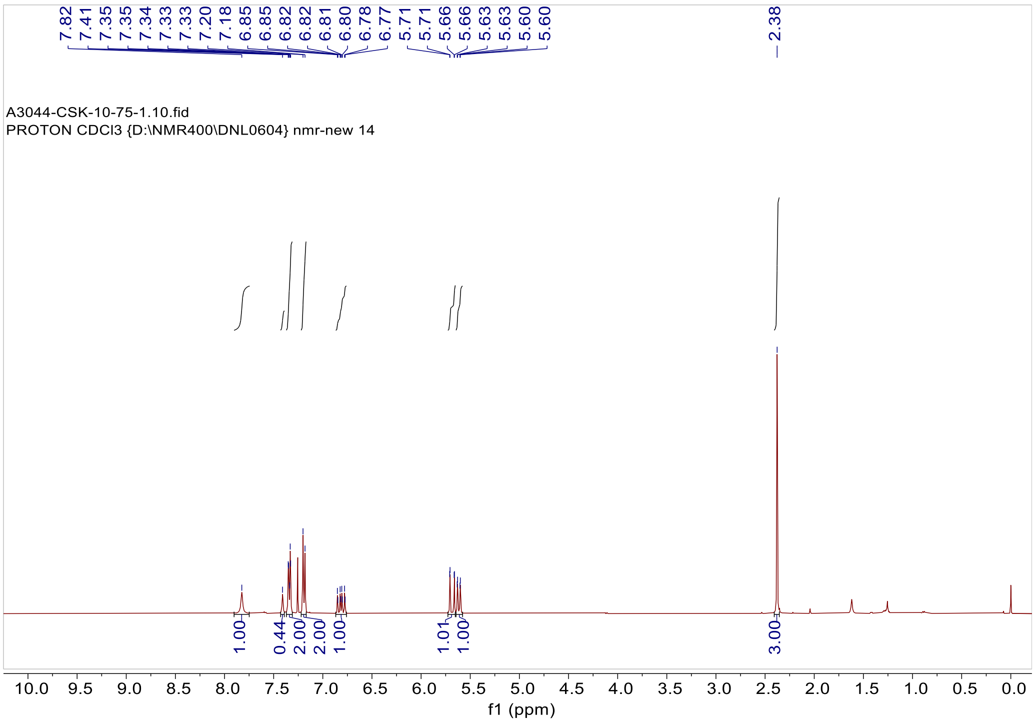

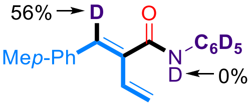

3e-*d-0* ^{1}H NMR (700 MHz, $CDCl_3$)

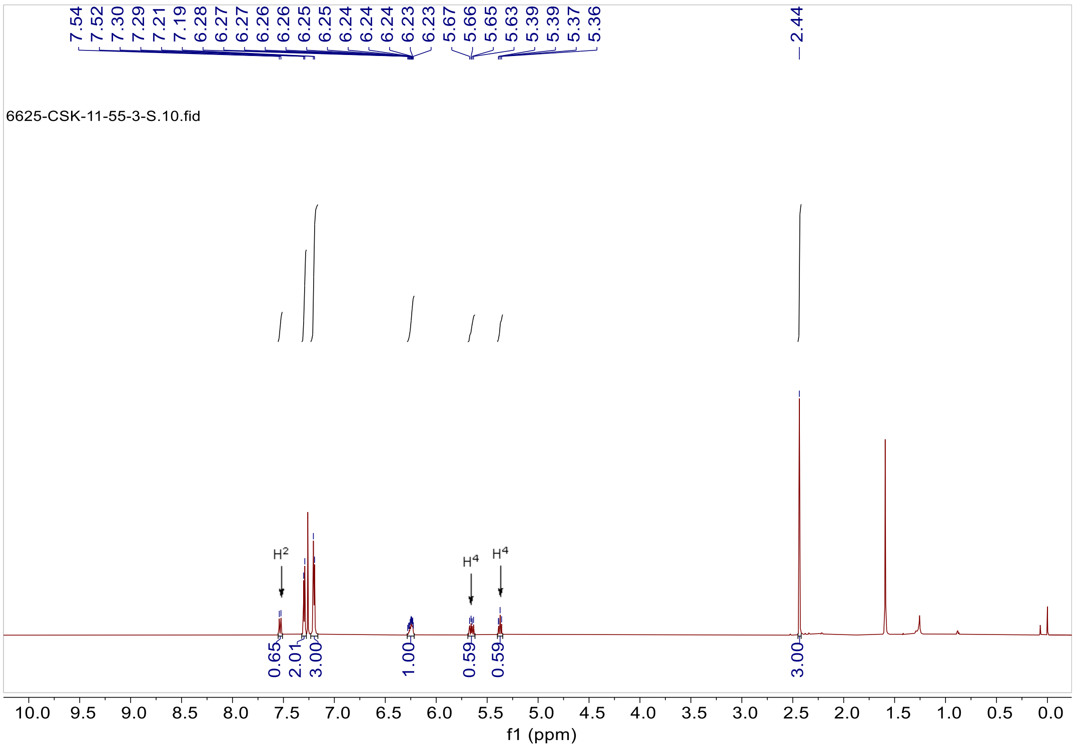

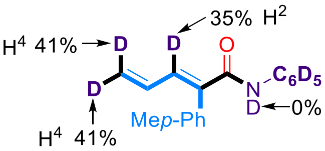

4e-*d-0* ^{1}H NMR (700 MHz, $CDCl_3$)

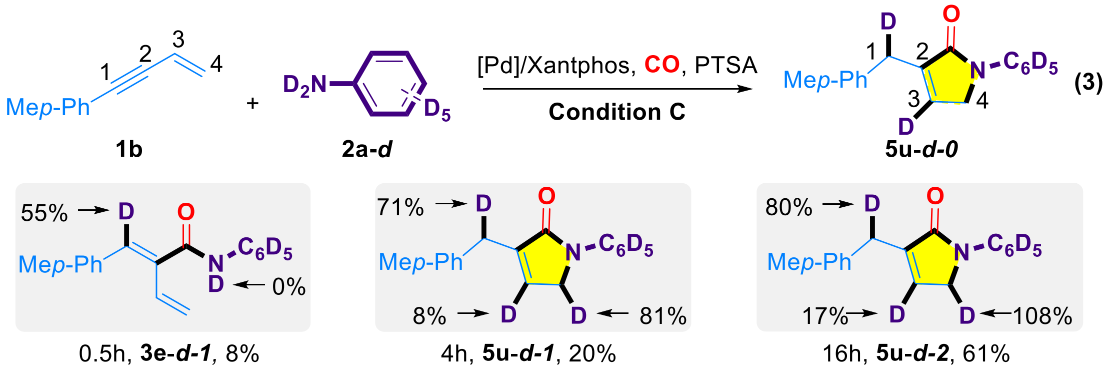

Condition C: A 4 mL screw-cap vial was charged with Pd(cod)Cl_2 (5 mol%), xantphos (5 mol%), PTSA (10 mol%) and an oven-dried stir bar. The vial was closed with a Teflon septum and cap and connected to the atmosphere via a needle. After toluene (1.0 mL), 1,3-enyne (**1b**, 0.10 mmol), aniline-d7 (**2a-*d***, 0.15 mmol), were added with a syringe under argon atmosphere, the vial was moved to an alloy plate and put into a Parr 4560 series autoclave (300 mL) under an argon atmosphere. At room temperature, the autoclave was flushed two times with nitrogen (~ 5 bar), and charged with 1 bar of CO and 5 bar of N_2. The autoclave was placed on a heating plate equipped with a magnetic stirrer and an aluminum block. The reaction mixture was heated to 100 °C for 0.5 h, 4.0 h, and 16.0 h, respectively. After completion, the autoclave was cooled to room temperature using ice water, and the pressure was carefully released. The reaction mixture was concentrated under reduced pressure, and the crude product was purified by column chromatography on silica gel to yield the corresponding products **3e-*d-1***, **5u-*d-1***, and **5u-*d-2***.

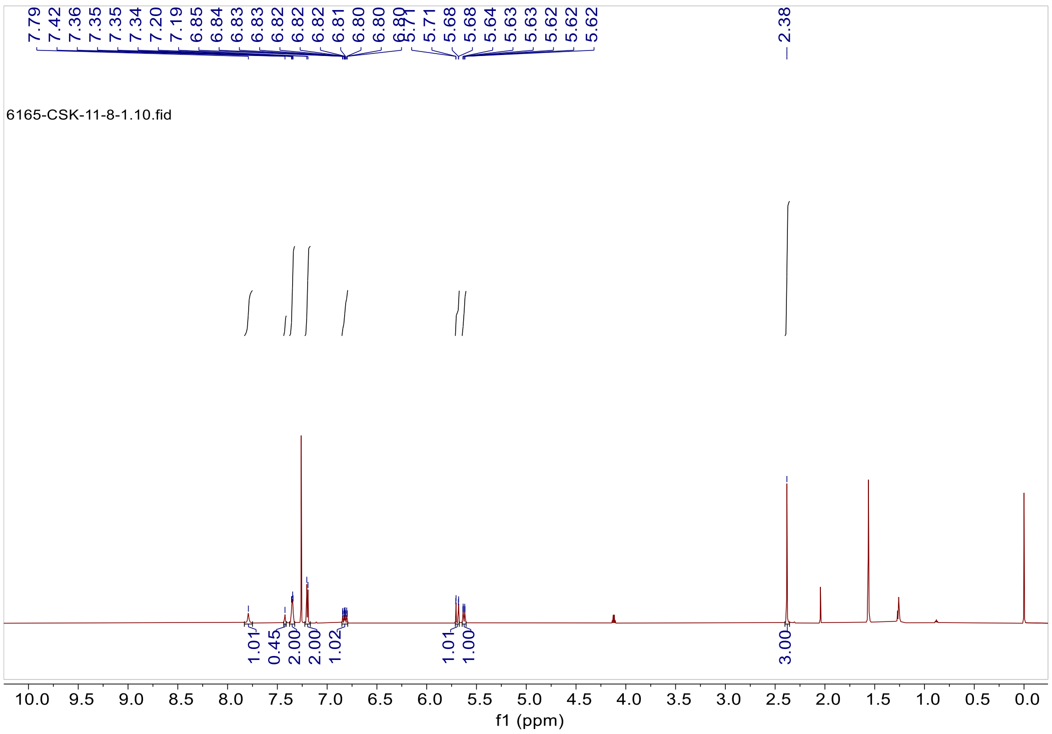

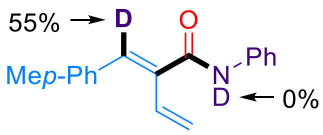

3e-*d-1* ^{1}H NMR (700 MHz, $CDCl_3$)

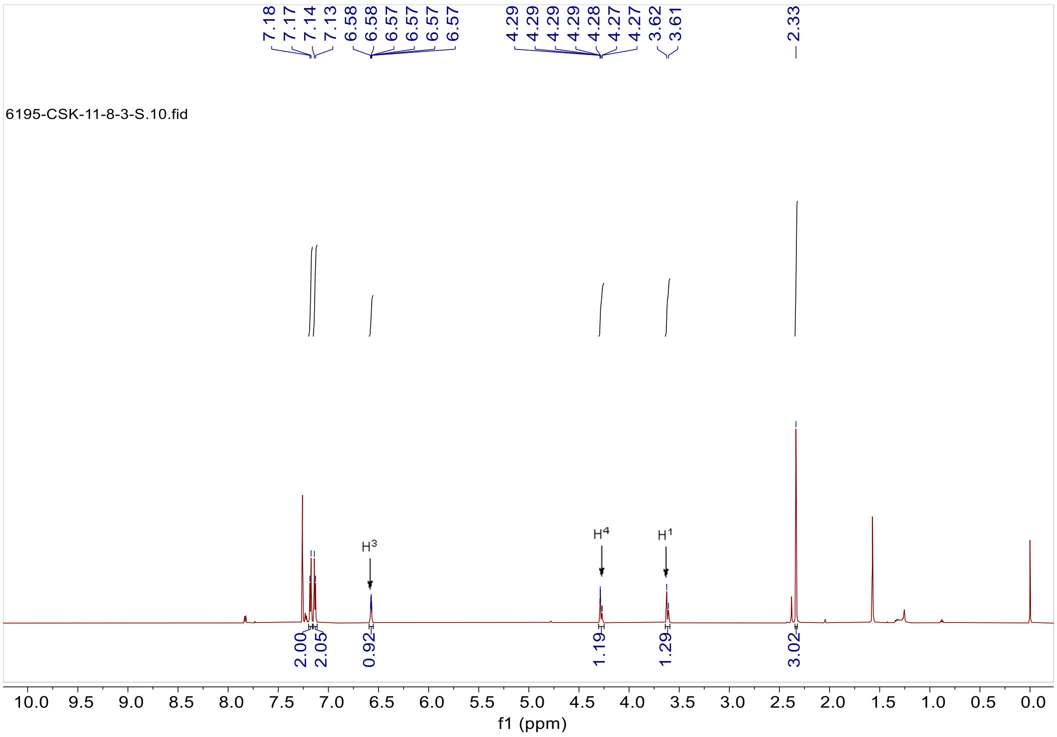

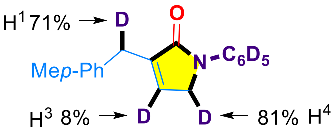

5u-*d-1* ^{1}H NMR (700 MHz, $CDCl_3$)

4h, **5ba-*d-1***

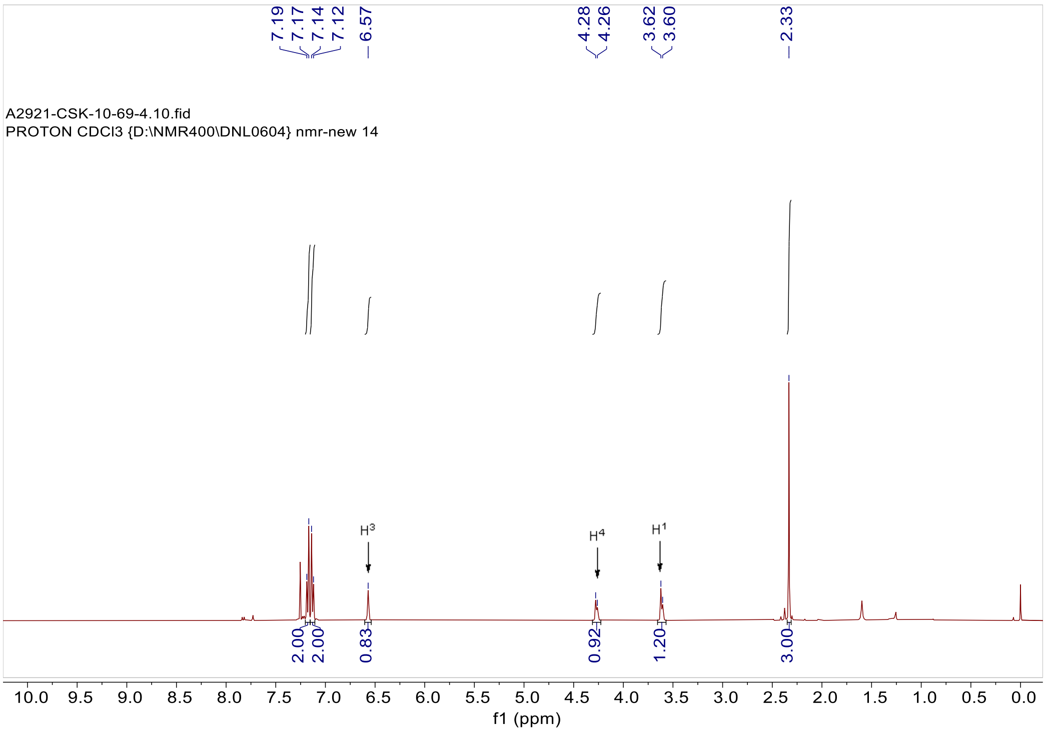

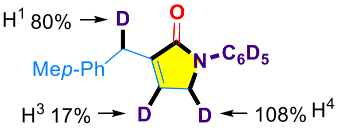

5u-*d-2* ^{1}H NMR (400 MHz, $CDCl_3$)

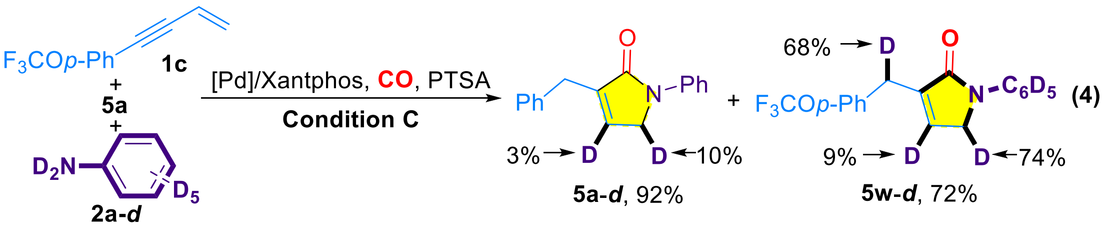

Condition C: A 4 mL screw-cap vial was charged with Pd(cod)Cl_2 (5 mol%), xantphos (5 mol%), PTSA (10 mol%) and an oven-dried stir bar. The vial was closed with a Teflon septum and cap and connected to the atmosphere via a needle. After toluene (1.0 mL), 1,3-enyne (**1c**, 0.10 mmol), aniline-d7 (**2a-*d***, 0.15 mmol) and **5a**(0.10 mmol), were added with a syringe under argon atmosphere, the vial was moved to an alloy plate and put into a Parr 4560 series autoclave (300 mL) under an argon atmosphere. At room temperature, the autoclave was flushed two times with nitrogen (~ 5 bar), and charged with 1 bar of CO and 5 bar of N_2. The autoclave was placed on a heating plate equipped with a magnetic stirrer and an aluminum block. The reaction mixture was heated to 100 °C for 16.0 h. After completion, the autoclave was cooled to room temperature using ice water, and the pressure was carefully released. The reaction mixture was concentrated under reduced pressure, and the crude product was purified by column chromatography on silica gel to yield the corresponding products **5a-*d***, and **5w-*d***.

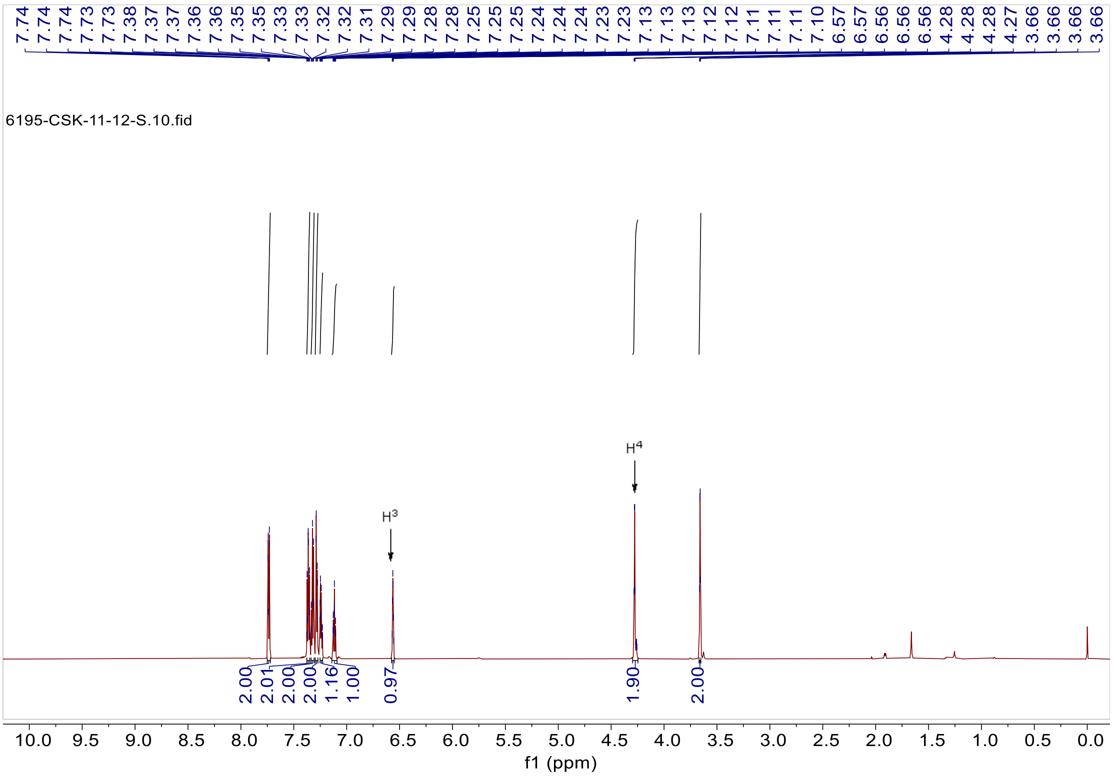

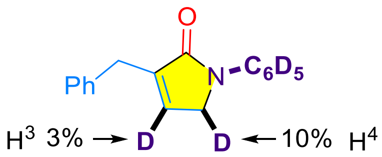

5a-*d* ^{1}H NMR (700 MHz, $CDCl_3$)

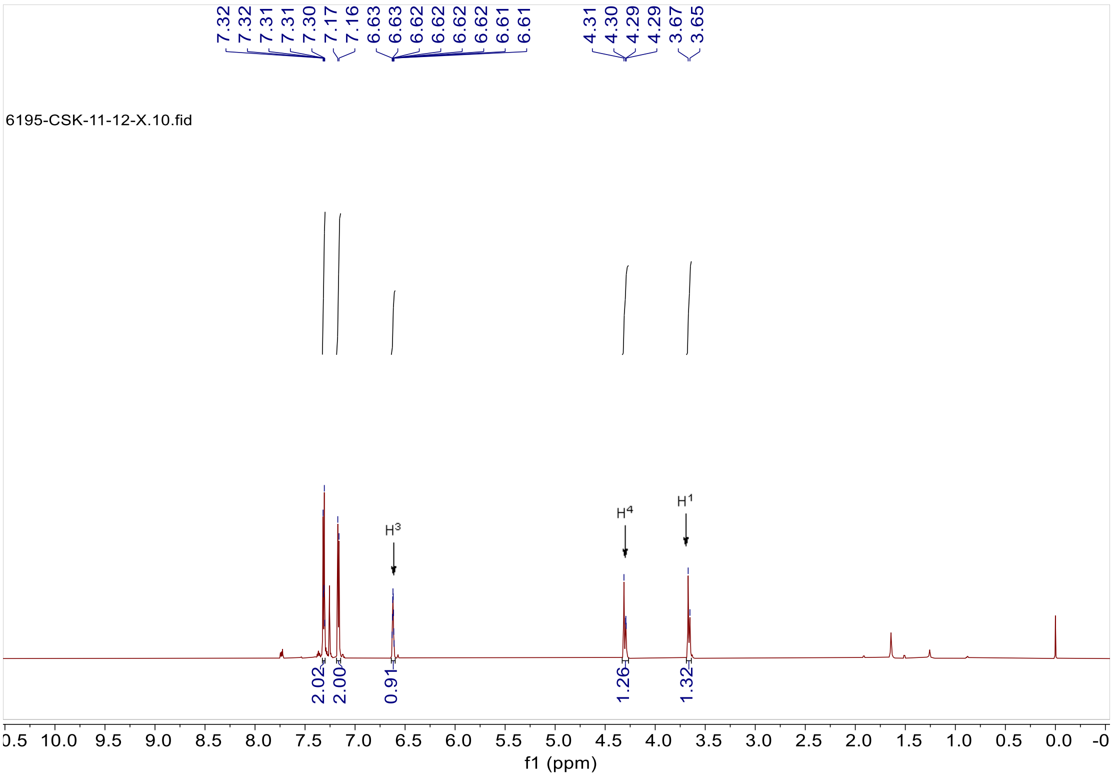

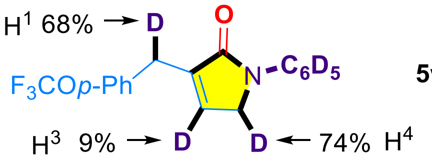

5w-*d* ^{1}H NMR (700 MHz, $CDCl_3$)

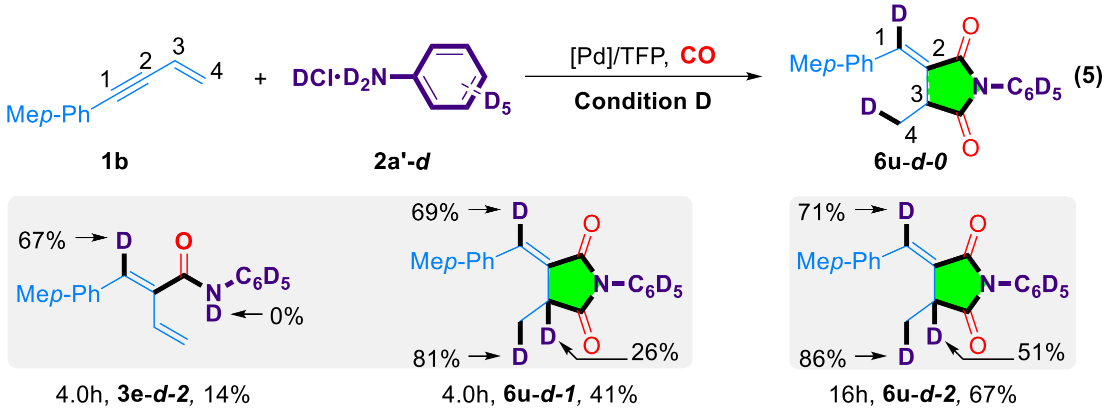

Condition D: A 4 mL screw-cap vial was charged with $Pd(PPh_3)_2Cl_2$ (5 mol%), TFP (10 mol%) and an oven-dried stir bar. The vial was closed with a Teflon septum and cap and connected to the atmosphere via a needle. After NMP (1.0 mL), 1,3-enyne (**1b**, 0.10 mmol), aniline hydrochloride-d7 (**2a'-d**, 0.15 mmol), were added with a syringe under argon atmosphere, the vial was moved to an alloy plate and put into a Parr 4560 series autoclave (300 mL) under an argon atmosphere. At room temperature, the autoclave was flushed two times with nitrogen (~ 5 bar), and charged with 50 bar of CO. The autoclave was placed on a heating plate equipped with a magnetic stirrer and an aluminum block. The reaction mixture was heated to 80 ºC for 4.0 h and 16.0 h, respectively. After completion, the autoclave was cooled to room temperature using ice water, and the pressure was carefully released. The reaction mixture was concentrated under reduced pressure, and the crude product was purified by column chromatography on silica gel to yield the corresponding products **3e-*d-2***, **6u-*d-1***, and **6u-*d-2***.

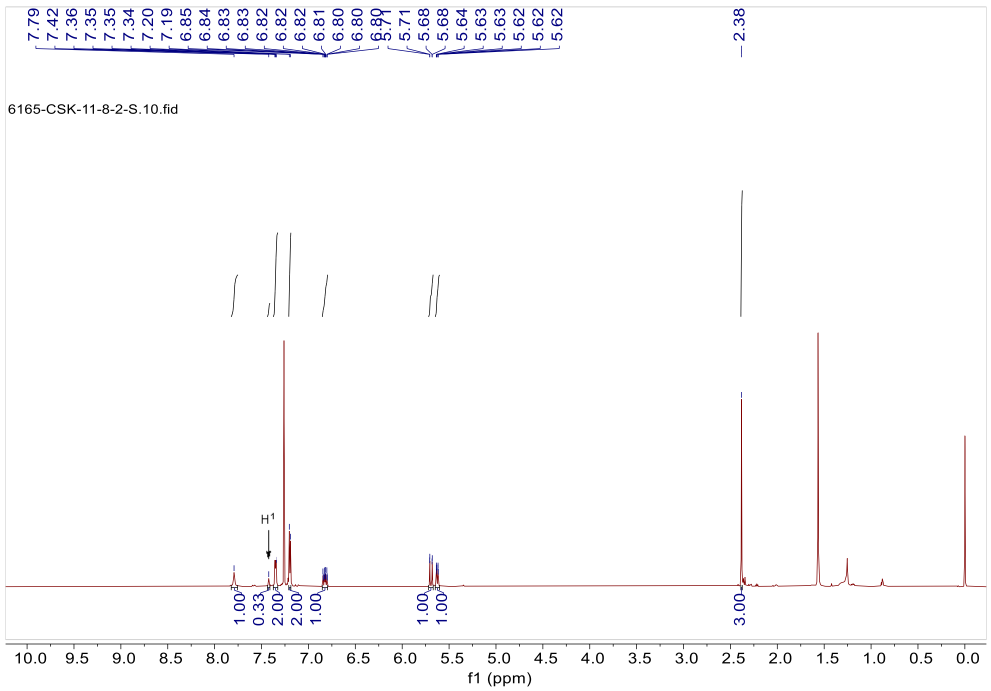

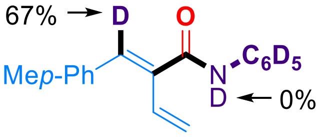

3e-*d-2* ^{1}H NMR (700 MHz, $CDCl_3$)

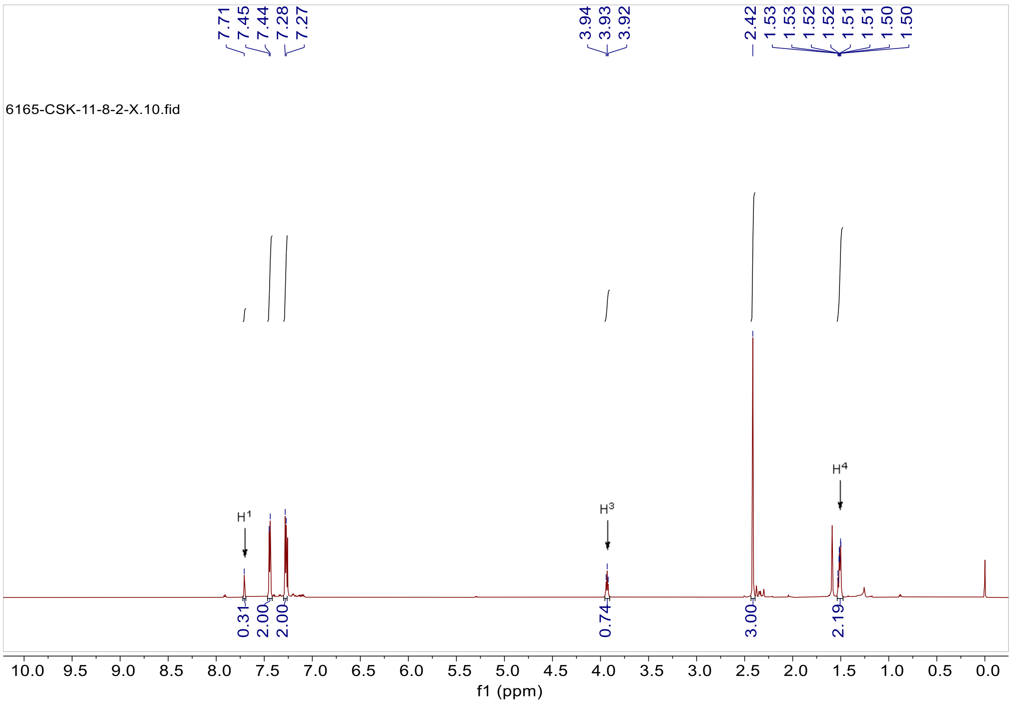

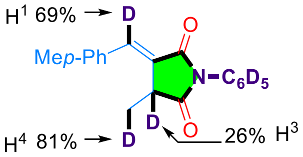

6u-*d-1* ^{1}H NMR (700 MHz, $CDCl_3$)

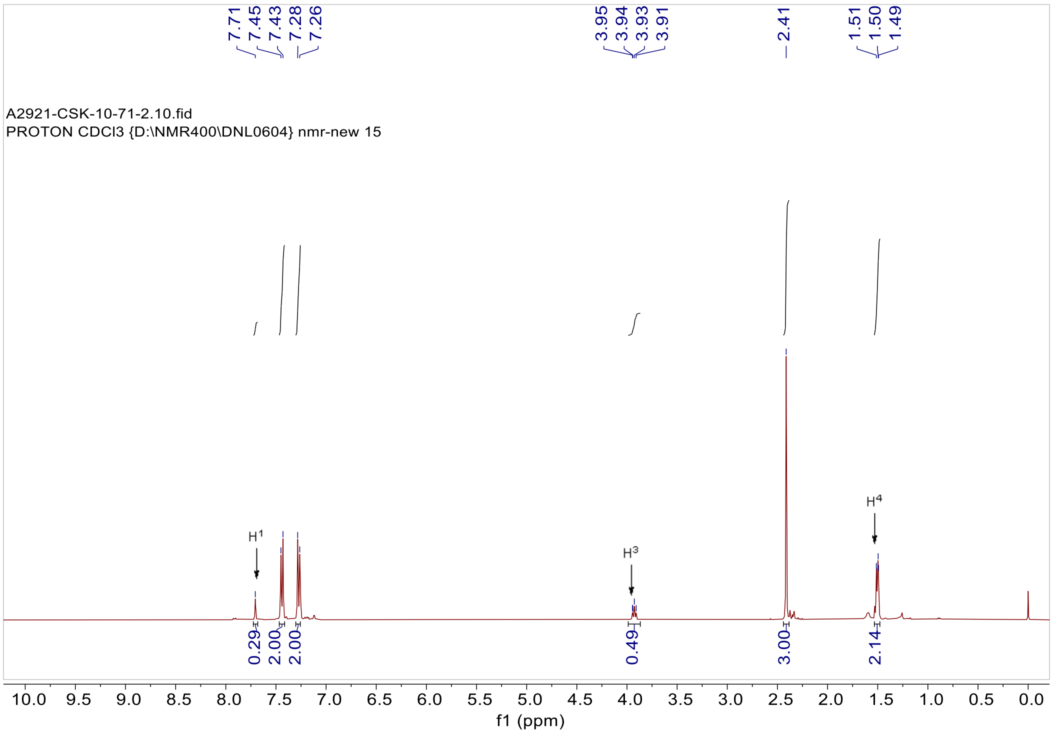

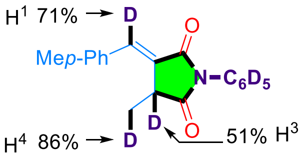

6u-*d-2* ^{1}H NMR (700 MHz, $CDCl_3$)

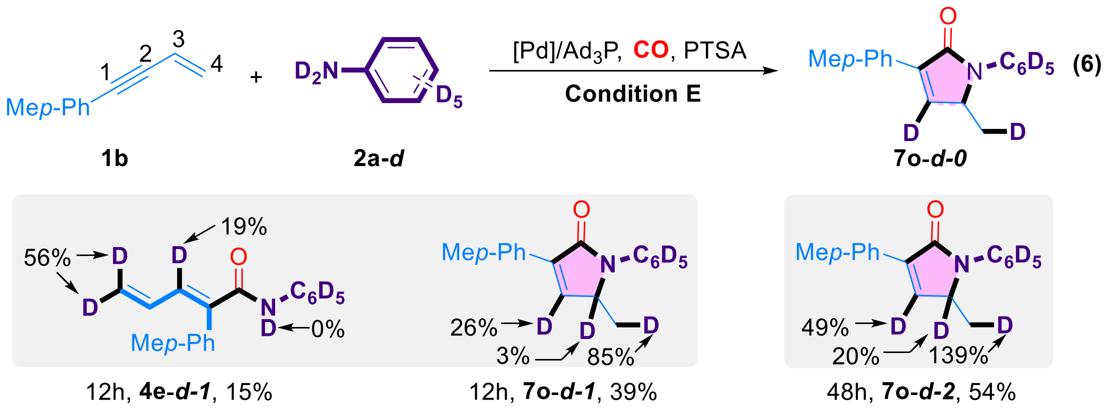

Condition E: A 4 mL screw-cap vial was charged with $PdCl_2$ (5 mol%), PAd_3 (15 mol%), PTSA (10 mol%) and an oven-dried stir bar. The vial was closed with a Teflon septum and cap and connected to the atmosphere via a needle. After $PhCF_3$ (1.0 mL), 1,3-enyne (**1b**, 0.10 mmol), aniline-d7 (**2a-*d***, 0.15 mmol), were added with a syringe under argon atmosphere, the vial was moved to an alloy plate and put into a Parr 4560 series autoclave (300 mL) under an argon atmosphere. At room temperature, the autoclave was flushed two times with nitrogen (~ 5 bar), and charged with 10 bar of CO. The autoclave was placed on a heating plate equipped with a magnetic stirrer and an aluminum block. The reaction mixture was heated to 80 ºC for 12 h and 48.0 h, respectively. After completion, the autoclave was cooled to room temperature using ice water, and the pressure was carefully released. The reaction mixture was concentrated under reduced pressure, and the crude product was purified by column chromatography on silica gel to yield the corresponding products **4e-*d-1***, **7o-*d-1***, and **7o-*d-2***.

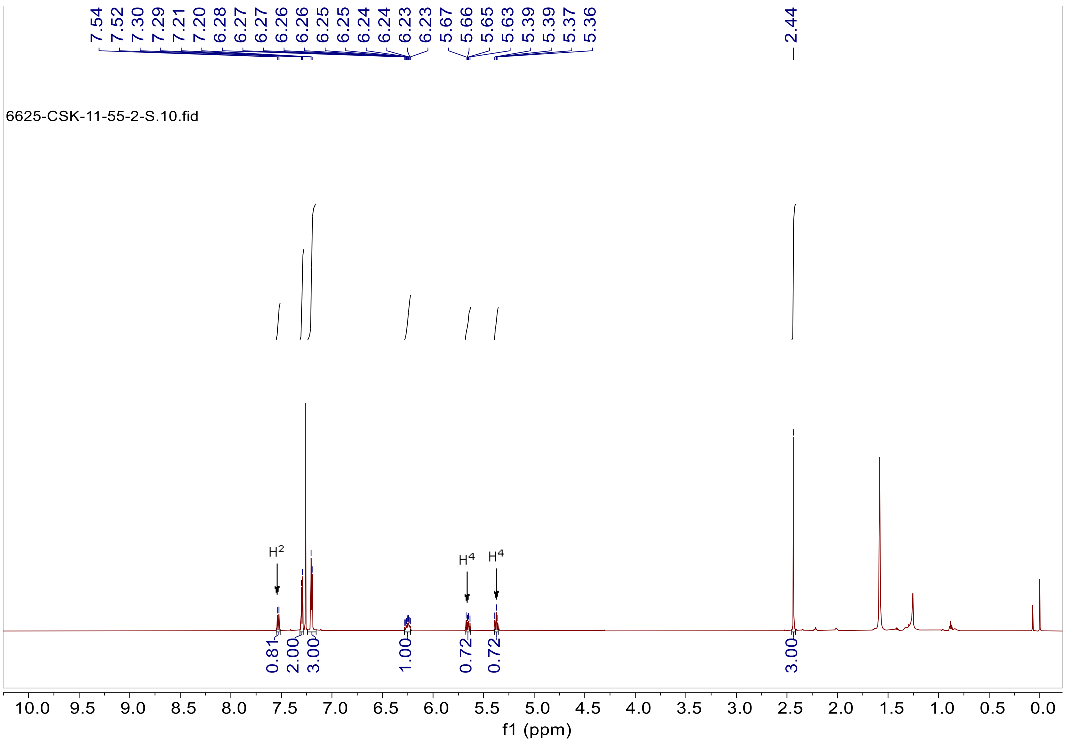

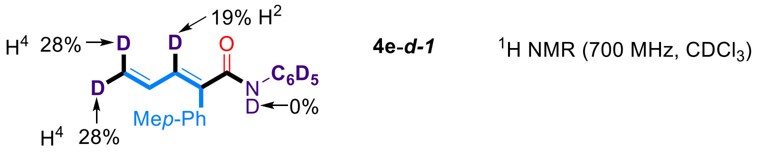

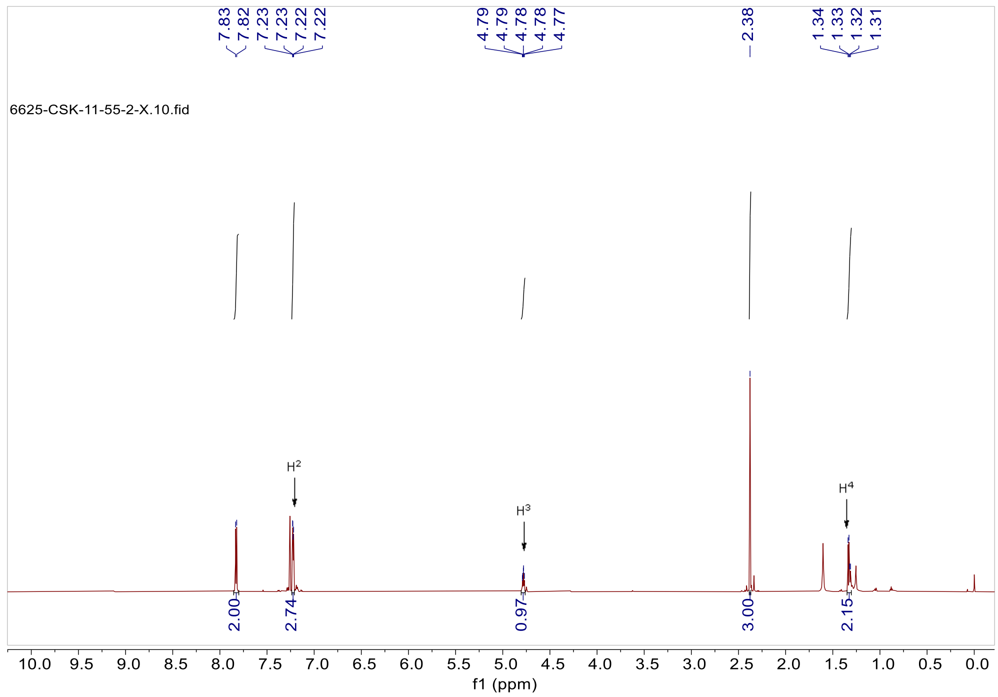

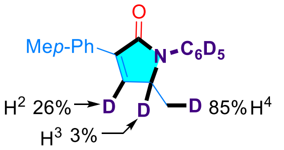

7o-*d*-1 ^{1}H NMR (700 MHz, $CDCl_3$)

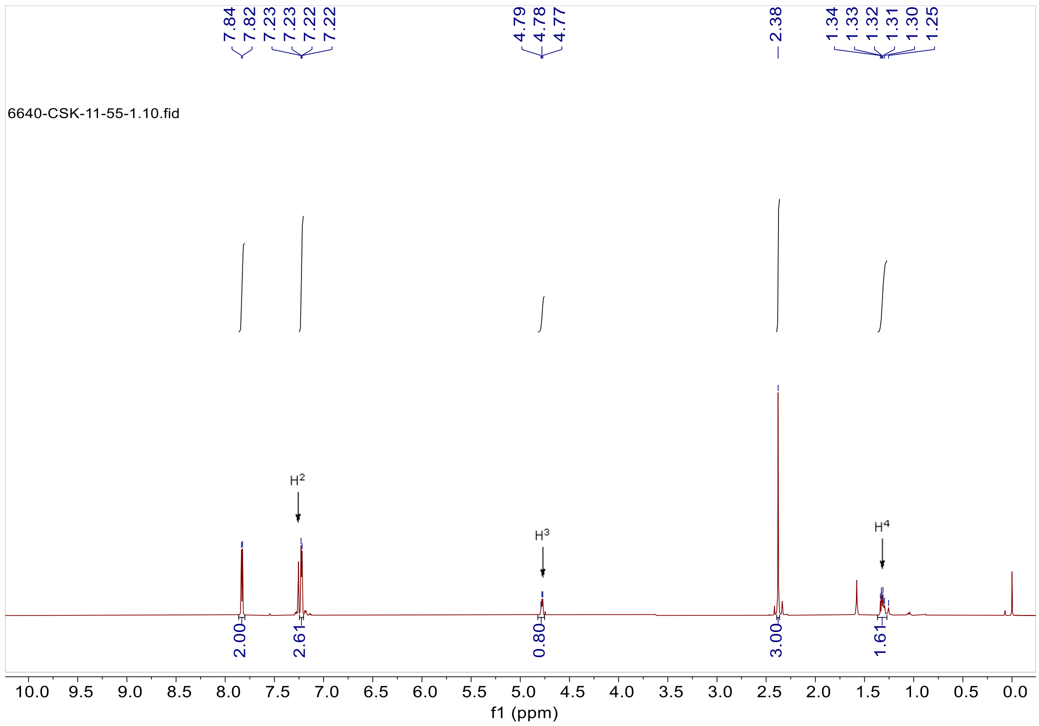

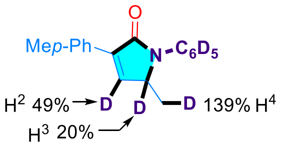

7o-*d*-2 ^{1}H NMR (700 MHz, $CDCl_3$)

6. Spectroscopic Data of Products

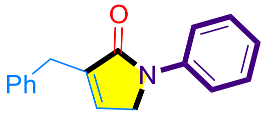

3-Benzyl-1-phenyl-1,5-dihydro-2H-pyrrol-2-one (5a):

White solid, 37.8mg, 76% yield, R_f=0.2 (PE/EtOAc 15/1).

^{1}H NMR (400 MHz, $CDCl_3$) δ 7.77 – 7.70 (m, 2H), 7.40 – 7.30 (m, 4H), 7.30 – 7.21 (m, 3H), 7.15 – 7.08 (m, 1H), 6.57 (p, J = 1.9 Hz, 1H), 4.28 (q, J = 2.1 Hz, 2H), 3.66 (q, J = 2.1 Hz, 2H).

^{13}C NMR (100 MHz, $CDCl_3$) δ 170.0, 141.1, 139.4, 138.3, 135.3, 129.1, 129.1, 128.6, 126.5, 124.0, 118.5, 51.1, 32.4.

HRMS (ESI-TOF) m/z: $[M + H]^+$ calculated for $C_{17}H_{16}NO$ 250.1226; Found 250.1228.

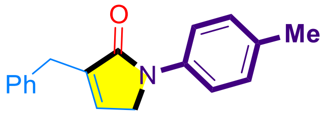

3-Benzyl-1-(*p*-tolyl)-1,5-dihydro-2H-pyrrol-2-one (5b):

White solid, 38.8mg, 74% yield, R_f=0.2 (PE/EtOAc 15/1).

^{1}H NMR (400 MHz, $CDCl_3$) δ 7.63 – 7.57 (m, 2H), 7.34 – 7.26 (m, 4H), 7.25 – 7.21 (m, 1H), 7.16 (d, J = 8.6 Hz, 2H), 6.53 (p, J = 1.9 Hz, 1H), 4.23 (q, J = 2.1 Hz, 2H), 3.65 (q, J = 2.1 Hz, 2H), 2.31 (s, 3H).

^{13}C NMR (100 MHz, $CDCl_3$) δ 169.9, 141.0, 138.3, 136.9, 135.1, 133.7, 129.6, 129.1, 128.6, 126.5, 118.7, 51.2, 32.4, 20.8.

HRMS (ESI-TOF) m/z: $[M + H]^+$ calculated for $C_{18}H_{18}NO$ 264.1383; Found 264.1386.

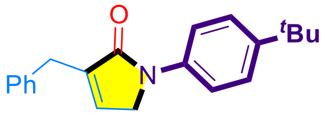

3-Benzyl-1-(4-(tert-butyl)phenyl)-1,5-dihydro-2H-pyrrol-2-one (5c):

White solid, 46.3mg, 76% yield, R_f=0.2 (PE/EtOAc 15/1).

^{1}H NMR (400 MHz, $CDCl_3$) δ 7.68 – 7.61 (m, 2H), 7.40 – 7.36 (m, 2H), 7.34 – 7.28 (m, 4H), 7.26 – 7.22 (m, 1H), 6.54 (t, J = 1.9 Hz, 1H), 4.26 (q, J = 2.1 Hz, 2H), 3.66 (q, J = 2.1 Hz, 2H), 1.31 (s, 9H).

^{13}C NMR (100 MHz, $CDCl_3$) δ 169.9, 147.0, 141.0, 138.3, 136.8, 135.2, 129.1, 128.6, 126.5, 125.9, 118.5, 51.2, 34.4, 32.4, 31.4.

HRMS (ESI-TOF) m/z: $[M + H]^+$ calculated for $C_{21}H_{24}NO$ 306.1852; Found 306.1860.

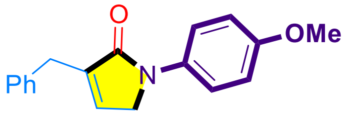

3-Benzyl-1-(4-methoxyphenyl)-1,5-dihydro-2H-pyrrol-2-one (5d):

White solid, 36.5mg, 65% yield, R_f=0.2 (PE/EtOAc 10/1).

^{1}H NMR (400 MHz, $CDCl_3$) δ 7.61 (d, J = 9.0 Hz, 2H), 7.36 – 7.26 (m, 4H), 7.26 – 7.20 (m, 1H), 6.90 (d, J = 9.2 Hz, 2H), 6.53 (p, J = 1.8 Hz, 1H), 4.24 (q, J = 2.1 Hz, 2H), 3.79 (s, 3H), 3.66 (q, J = 2.1 Hz, 2H).

^{13}C NMR (100 MHz, $CDCl_3$) δ 169.8, 156.3, 141.0, 138.4, 135.0, 132.7, 129.1, 128.6, 126.5, 120.6, 114.3, 55.5, 51.5, 32.4.

HRMS (ESI-TOF) m/z: $[M + H]^+$ calculated for $C_{18}H_{18}NO_2$ 280.1332; Found 280.1339.

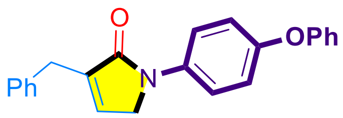

3-Benzyl-1-(4-phenoxyphenyl)-1,5-dihydro-2H-pyrrol-2-one (5e):

White solid, 52.5mg, 77% yield, R_f=0.2 (PE/EtOAc 10/1).

^{1}**H NMR** (400 MHz, $CDCl_3$) δ 7.72 – 7.65 (m, 2H), 7.37 – 7.26 (m, 6H), 7.26 – 7.21 (m, 1H), 7.08 (t, J = 7.4 Hz, 1H), 7.05 – 7.01 (m, 2H), 7.01 – 6.96 (m, 2H), 6.56 (p, J = 1.8 Hz, 1H), 4.27 (q, J = 2.1 Hz, 2H), 3.66 (q, J = 2.1 Hz, 2H).

^{13}**C NMR** (100 MHz, $CDCl_3$) δ 169.9, 157.6, 153.3, 141.0, 138.3, 135.2, 135.1, 129.8, 129.1, 128.7, 126.5, 123.1, 120.3, 119.8, 118.4, 51.3, 32.4.

HRMS (ESI-TOF) m/z: $[M + H]^+$ calculated for $C_{23}H_{20}NO_2$ 342.1489; Found 342.1495.

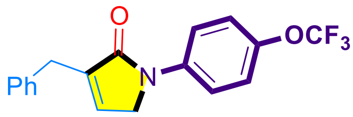

3-Benzyl-1-(4-(trifluoromethoxy)phenyl)-1,5-dihydro-2H-pyrrol-2-one (5f):

White solid, 48.2mg, 72% yield, R_f=0.2 (PE/EtOAc 10/1).

^{1}**H NMR** (400 MHz, $CDCl_3$) δ 7.84 – 7.73 (m, 2H), 7.36 – 7.29 (m, 3H), 7.28 – 7.18 (m, 4H), 6.60 (p, J = 1.8 Hz, 1H), 4.28 (q, J = 2.1 Hz, 2H), 3.66 (q, J = 2.1 Hz, 2H).

^{13}**C NMR** (100 MHz, $CDCl_3$) δ 170.0, 145.1 (q, J = 1.8 Hz), 141.0, 138.1, 138.0, 135.5, 129.1, 128.7, 126.6, 121.8, 120.5 (q, J = 256.8 Hz), 119.5, 51.0, 32.4.

^{19}**F NMR** (376 MHz, $CDCl_3$) δ -58.10.

HRMS (ESI-TOF) m/z: $[M + H]^+$ calculated for $C_{18}H_{15}F_3NO_2$ 334.1049; Found 334.1047.

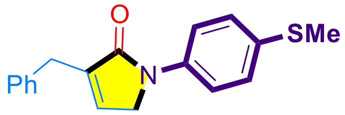

3-Benzyl-1-(4-(methylthio)phenyl)-1,5-dihydro-2H-pyrrol-2-one (5g):

White solid, 38.3mg, 65% yield, R_f=0.2 (PE/EtOAc 5/1).

^{1}**H NMR** (400 MHz, $CDCl_3$) δ 7.71 – 7.65 (m, 2H), 7.35 – 7.27 (m, 5H), 7.27 – 7.22 (m, 2H), 6.56 (p, J = 1.8 Hz, 1H), 4.25 (q, J = 2.1 Hz, 2H), 3.65 (q, J = 2.1 Hz, 2H), 2.47 (s, 3H).

^{13}**C NMR** (100 MHz, $CDCl_3$) δ 169.9, 141.0, 138.2, 137.1, 135.2, 133.3, 129.1, 128.6, 128.1, 126.5, 119.1, 51.0, 32.4, 16.7.

HRMS (ESI-TOF) m/z: $[M + H]^+$ calculated for $C_{18}H_{18}NOS$ 296.1104; Found 296.1102.

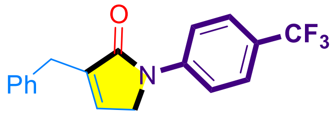

3-Benzyl-1-(4-(trifluoromethyl)phenyl)-1,5-dihydro-2H-pyrrol-2-one (5h):

White solid, 38.2mg, 60% yield, R_f=0.2 (PE/EtOAc 15/1).

^{1}**H NMR** (400 MHz, $CDCl_3$) δ 7.88 (d, J = 8.9 Hz, 2H), 7.61 (d, J = 9.0 Hz, 2H), 7.36 – 7.30 (m, 2H), 7.30 – 7.22 (m, 3H), 6.63 (p, J = 2.0 Hz, 1H), 4.30 (q, J = 2.1 Hz, 2H), 3.66 (q, J = 2.1 Hz, 2H).

^{13}**C NMR** (100 MHz, $CDCl_3$) δ 170.3, 142.3, 141.0, 137.9, 135.9, 129.1, 128.7, 126.7, 126.3 (q, J = 3.7 Hz), 125.5 (q, J = 33.0 Hz), 124.2 (q, J = 271.4 Hz), 117.7, 50.8, 32.3.

^{19}**F NMR** (376 MHz, $CDCl_3$) δ -62.06.

HRMS (ESI-TOF) m/z: $[M + H]^+$ calculated for $C_{18}H_{15}F_3NO$ 318.1100; Found 318.1097.

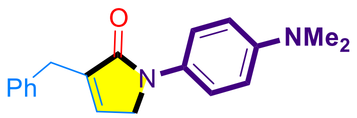

3-Benzyl-1-(4-(dimethylamino)phenyl)-1,5-dihydro-2H-pyrrol-2-one (5i):

White solid, 37.7mg, 65% yield, R_f=0.2 (PE/EtOAc 3/1).

^{1}H NMR (400 MHz, $CDCl_3$) δ 7.57 – 7.50 (m, 2H), 7.35 – 7.27 (m, 4H), 7.26 – 7.21 (m, 1H), 6.77 – 6.71 (m, 2H), 6.49 (p, J = 1.9 Hz, 1H), 4.21 (q, J = 2.1 Hz, 2H), 3.65 (q, J = 2.1 Hz, 2H), 2.92 (s, 6H).

^{13}C NMR (100 MHz, $CDCl_3$) δ 169.7, 147.9, 141.0, 138.5, 134.7, 129.4, 129.2, 128.6, 126.4, 120.8, 113.1, 51.7, 40.9, 32.5.

HRMS (ESI-TOF) m/z: $[M + H]^+$ calculated for $C_{19}H_{21}N_2O$ 293.1648; Found 193.1647.

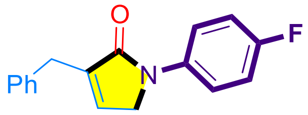

3-Benzyl-1-(4-fluorophenyl)-1,5-dihydro-2H-pyrrol-2-one (5j):

White solid, 38.1mg, 71% yield, R_f=0.2 (PE/EtOAc 15/1).

^{1}H NMR (400 MHz, $CDCl_3$) δ 7.76 – 7.63 (m, 2H), 7.37 – 7.22 (m, 5H), 7.05 (t, J = 8.7 Hz, 2H), 6.57 (p, J = 2.1 Hz, 1H), 4.25 (q, J = 2.2 Hz, 2H), 3.65 (q, J = 2.1 Hz, 2H).

^{13}C NMR (100 MHz, $CDCl_3$) δ 169.9, 159.2 (d, J = 243.6 Hz), 140.9, 138.2, 135.6 (d, J = 2.9 Hz), 135.3, 129.1, 128.7, 126.6, 120.3 (d, J = 7.7 Hz), 115.7 (d, J = 22.0 Hz), 51.3, 32.4.

^{19}F NMR (376 MHz, $CDCl_3$) δ -118.47.

HRMS (ESI-TOF) m/z: $[M + H]^+$ calculated for $C_{17}H_{15}FNO$ 268.1132; Found 268.1130.

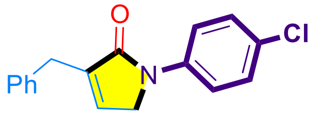

3-Benzyl-1-(4-chlorophenyl)-1,5-dihydro-2H-pyrrol-2-one (5k):

White solid, 38.5mg, 68% yield, R_f=0.2 (PE/EtOAc 15/1).

^{1}H NMR (400 MHz, $CDCl_3$) δ 7.73 – 7.64 (m, 2H), 7.38 – 7.22 (m, 7H), 6.58 (p, J = 2.0 Hz, 1H), 4.24 (q, J = 2.3 Hz, 2H), 3.65 (q, J = 2.2 Hz, 2H).

^{13}C NMR (100 MHz, $CDCl_3$) δ 167.0, 141.0, 138.1, 138.0, 135.4, 129.1, 129.1, 129.0, 128.7, 126.6, 119.5, 51.0, 32.4.

HRMS (ESI-TOF) m/z: $[M + H]^+$ calculated for $C_{17}H_{15}ClNO$ 284.0837; Found 284.0846.

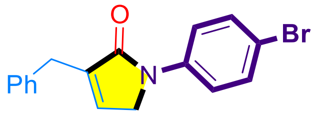

3-Benzyl-1-(4-bromophenyl)-1,5-dihydro-2H-pyrrol-2-one (5l):

White solid, 33.1mg, 51% yield, R_f=0.2 (PE/EtOAc 20/1).

^{1}H NMR (400 MHz, $CDCl_3$) δ 7.68 – 7.61 (m, 2H), 7.50 – 7.43 (m, 2H), 7.36 – 7.30 (m, 2H), 7.29 – 7.22 (m, 3H), 6.58 (p, J = 2.0 Hz, 1H), 4.25 (q, J = 2.1 Hz, 2H), 3.65 (q, J = 2.1 Hz, 2H).

^{13}C NMR (100 MHz, $CDCl_3$) δ 170.0, 141.0, 138.5, 138.0, 135.4, 132.0, 129.1, 128.7, 126.6, 119.8, 116.7, 50.9, 32.4.

HRMS (ESI-TOF) m/z: $[M + H]^+$ calculated for $C_{17}H_{15}BrNO$ 328.0332; Found 328.0341.

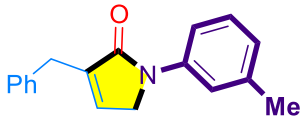

3-Benzyl-1-(m-tolyl)-1,5-dihydro-2H-pyrrol-2-one (5m):
White solid, 40.7mg, 77% yield, R_f=0.2 (PE/EtOAc 10/1).
^{1}H NMR (400 MHz, $CDCl_3$) δ 7.61 (t, J = 2.1 Hz, 1H), 7.48 (dd, J = 8.2, 2.7 Hz, 1H), 7.35 – 7.27 (m, 4H), 7.26 – 7.20 (m, 2H), 6.94 (d, J = 7.6 Hz, 1H), 6.55 (p, J = 1.9 Hz, 1H), 4.26 (q, J = 2.1 Hz, 2H), 3.65 (q, J = 2.1 Hz, 2H), 2.36 (s, 3H).
^{13}C NMR (100 MHz, $CDCl_3$) δ 170.0, 141.0, 139.4, 139.0, 138.3, 135.3, 129.1, 128.9, 128.6, 126.5, 124.9, 119.4, 115.7, 51.2, 32.4, 21.7.
HRMS (ESI-TOF) m/z: $[M + H]^+$ calculated for $C_{18}H_{18}NO$ 264.1383; Found 264.1391.

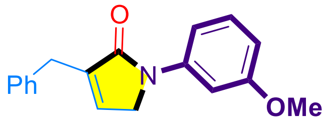

3-Benzyl-1-(3-methoxyphenyl)-1,5-dihydro-2H-pyrrol-2-one (5n):
White solid, 40.7mg, 73% yield, R_f=0.2 (PE/EtOAc 5/1).
^{1}H NMR (400 MHz, $CDCl_3$) δ 7.56 (t, J = 2.3 Hz, 1H), 7.36 – 7.21 (m, 6H), 7.20 – 7.15 (m, 1H), 6.70 – 6.63 (m, 1H), 6.55 (t, J = 1.9 Hz, 1H), 4.25 (d, J = 2.2 Hz, 2H), 3.82 (s, 3H), 3.65 (d, J = 2.1 Hz, 2H).
^{13}C NMR (100 MHz, $CDCl_3$) δ 170.1, 160.3, 141.0, 140.7, 138.2, 135.4, 129.7, 129.1, 128.6, 126.5, 110.4, 109.8, 104.4, 55.3, 51.2, 32.3.
HRMS (ESI-TOF) m/z: $[M + H]^+$ calculated for $C_{18}H_{18}NO_2$ 280.1332; Found 280.1339.

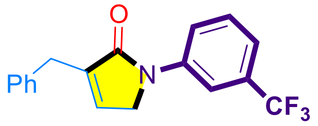

3-Benzyl-1-(3-(trifluoromethyl)phenyl)-1,5-dihydro-2H-pyrrol-2-one (5o):
White solid, 39.1mg, 62% yield, R_f=0.2 (PE/EtOAc 10/1).
^{1}H NMR (400 MHz, $CDCl_3$) δ 8.04 – 7.96 (m, 2H), 7.48 (t, J = 7.9 Hz, 1H), 7.40 – 7.31 (m, 3H), 7.30 – 7.23 (m, 3H), 6.64 (p, J = 2.0 Hz, 1H), 4.32 (q, J = 2.1 Hz, 2H), 3.67 (q, J = 2.1 Hz, 2H).
^{13}C NMR (100 MHz, $CDCl_3$) δ 170.2, 141.0, 139.9, 137.9, 135.7, 131.5 (q, J = 32.3 Hz), 129.7, 129.1, 128.7, 126.6, 124.0 (q, J = 272.9 Hz), 121.3, 120.4 (q, J = 4.0 Hz), 114.8 (q, J = 4.0 Hz), 50.9, 32.3.
^{19}F NMR (376 MHz, $CDCl_3$) δ -62.68.
HRMS (ESI-TOF) m/z: $[M + H]^+$ calculated for $C_{18}H_{15}F_3NO$ 318.1100; Found 318.1107.

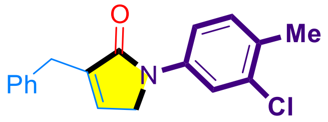

3-Benzyl-1-(3-chloro-4-methylphenyl)-1,5-dihydro-2H-pyrrol-2-one (5p):
Yellow solid, 35.7mg, 60% yield, R_f=0.2 (PE/EtOAc 15/1).
^{1}H NMR (400 MHz, $CDCl_3$) δ 7.76 (d, J = 2.4 Hz, 1H), 7.56 (dd, J = 8.4, 2.4 Hz, 1H), 7.35 – 7.26 (m, 4H), 7.26 – 7.21 (m, 1H), 7.19 (d, J = 8.6 Hz, 1H), 6.57 (p, J = 1.9 Hz, 1H), 4.23 (q, J = 2.1 Hz, 2H), 3.64 (q, J = 2.1 Hz, 2H), 2.33 (s, 3H).

^{13}C NMR (100 MHz, $CDCl_3$) δ 169.9, 141.0, 138.2, 138.1, 135.4, 134.7, 131.5, 131.1, 129.1, 128.7, 126.6, 118.9, 116.6, 51.0, 32.4, 19.4.

HRMS (ESI-TOF) m/z: $[M + H]^+$ calculated for $C_{18}H_{17}ClNO$ 298.0993; Found 298.1001.

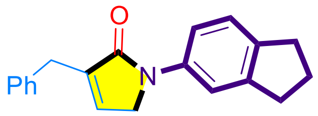

3-Benzyl-1-(2,3-dihydro-1H-inden-5-yl)-1,5-dihydro-2H-pyrrol-2-one (5r):

White solid, 38.6mg, 67% yield, R_f=0.2 (PE/EtOAc 15/1).

^{1}H NMR (400 MHz, $CDCl_3$) δ 7.66 – 7.60 (m, 1H), 7.39 (dd, J = 8.1, 2.3 Hz, 1H), 7.34 – 7.26 (m, 4H), 7.25 – 7.21 (m, 1H), 7.19 (d, J = 8.1 Hz, 1H), 6.53 (p, J = 1.8 Hz, 1H), 4.24 (q, J = 2.1 Hz, 2H), 3.65 (q, J = 2.1 Hz, 2H), 2.89 (dt, J = 15.4, 7.5 Hz, 4H), 2.07 (p, J = 7.5 Hz, 2H).

^{13}C NMR (100 MHz, $CDCl_3$) δ 169.9, 145.3, 141.0, 140.2, 138.4, 137.6, 135.1, 129.2, 128.6, 126.5, 124.6, 117.0, 115.5, 51.6, 33.2, 32.5, 32.4, 25.7.

HRMS (ESI-TOF) m/z: $[M + H]^+$ calculated for $C_{20}H_{20}NO$ 290.1539; Found 290.1545.

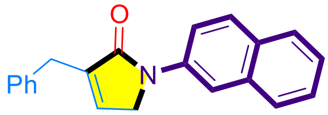

3-Benzyl-1-(naphthalen-2-yl)-1,5-dihydro-2H-pyrrol-2-one (5s):

White solid, 34.0mg, 57% yield, R_f=0.2 (PE/EtOAc 15/1).

^{1}H NMR (400 MHz, $CDCl_3$) δ 8.08 (d, J = 2.4 Hz, 1H), 8.02 (dd, J = 8.9, 2.3 Hz, 1H), 7.83 (d, J = 9.0 Hz, 1H), 7.79 (dd, J = 8.3, 3.4 Hz, 2H), 7.47 – 7.43 (m, 1H), 7.42 – 7.37 (m, 1H), 7.36 – 7.28 (m, 4H), 7.27 – 7.23 (m, 1H), 6.60 (q, J = 1.9 Hz, 1H), 4.38 (q, J = 2.2 Hz, 2H), 3.69 (q, J = 2.2 Hz, 2H).

^{13}C NMR (100 MHz, $CDCl_3$) δ 170.2, 141.1, 138.2, 137.1, 135.5, 133.8, 130.4, 129.2, 128.9, 128.7, 127.7, 127.6, 126.6, 126.5, 125.0, 118.6, 115.2, 51.3, 32.4.

HRMS (ESI-TOF) m/z: $[M + H]^+$ calculated for $C_{21}H_{18}NO$ 300.1383; Found 300.1395.

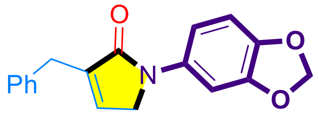

1-(Benzo[d][1,3]dioxol-5-yl)-3-benzyl-1,5-dihydro-2H-pyrrol-2-one (5t):

White solid, 32.8 mg, 56% yield, R_f=0.2 (PE/DCM/EtOAc 10/4/1).

^{1}H NMR (400 MHz, $CDCl_3$) δ 7.45 (d, J = 2.2 Hz, 1H), 7.35 – 7.22 (m, 5H), 6.98 (dd, J = 8.4, 2.2 Hz, 1H), 6.78 (d, J = 8.4 Hz, 1H), 6.53 (p, J = 1.8 Hz, 1H), 5.95 (s, 2H), 4.22 (q, J = 2.1 Hz, 2H), 3.65 (q, J = 2.1 Hz, 2H).

^{13}C NMR (100 MHz, $CDCl_3$) δ 169.8, 148.0, 144.2, 140.9, 138.3, 135.0, 133.9, 129.1, 128.6, 126.5, 111.9, 108.1, 101.8, 101.3, 51.8, 32.4.

HRMS (ESI-TOF) m/z: $[M + H]^+$ calculated for $C_{18}H_{16}NO_3$ 294.1125; Found 295.1135.

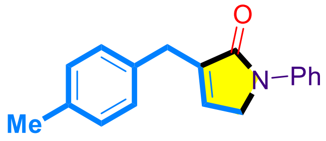

3-(4-Methylbenzyl)-1-phenyl-1,5-dihydro-2H-pyrrol-2-one (5u):

White solid, 34.6 mg, 66% yield, R_f=0.2 (PE/EtOAc 10/1).

^{1}H NMR (400 MHz, $CDCl_3$) δ 7.76 – 7.70 (m, 2H), 7.36 (dd, J = 8.7, 7.4 Hz, 2H), 7.19 – 7.08 (m, 5H), 6.56 (p, J = 1.8 Hz, 1H), 4.26 (q, J = 2.1 Hz, 2H), 3.61 (q, J = 2.1 Hz, 2H), 2.33 (s, 3H).

^{13}C NMR (100 MHz, $CDCl_3$) δ 170.1, 141.3, 139.5, 136.0, 135.20, 135.18, 129.3, 129.1, 129.0, 124.0, 118.5, 51.1, 32.0, 21.1.

HRMS (ESI-TOF) m/z: $[M + H]^+$ calculated for $C_{18}H_{18}NO$ 264.1383; Found 264.1394.

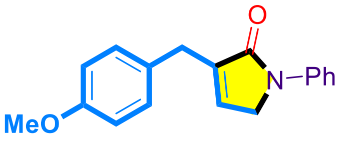

3-(4-Methoxybenzyl)-1-phenyl-1,5-dihydro-2H-pyrrol-2-one (5v):

Light yellow solid, 35.2 mg, 63% yield, R_f=0.2 (PE/EtOAc 5/1).

^{1}H NMR (400 MHz, $CDCl_3$) δ 7.77 – 7.68 (m, 2H), 7.39 – 7.34 (m, 2H), 7.20 (d, J = 8.7 Hz, 2H), 7.12 (t, J = 7.5 Hz, 1H), 6.86 (d, J = 8.7 Hz, 2H), 6.56 (p, J = 2.0 Hz, 1H), 4.28 (q, J = 2.1 Hz, 2H), 3.79 (s, 3H), 3.60 (q, J = 2.2 Hz, 2H).

^{13}C NMR (100 MHz, $CDCl_3$) δ 170.1, 158.3, 141.4, 139.4, 135.1, 130.3, 130.1, 129.1, 124.0, 118.5, 114.0, 55.3, 51.0, 31.5.

HRMS (ESI-TOF) m/z: $[M + H]^+$ calculated for $C_{18}H_{18}NO_2$ 280.1332; Found 280.1336.

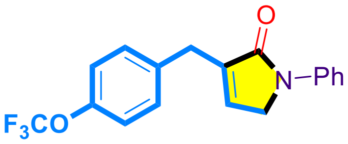

1-Phenyl-3-(4-(trifluoromethoxy)benzyl)-1,5-dihydro-2H-pyrrol-2-one (5w):

White solid, 40.9 mg, 61% yield, R_f=0.2 (PE/EtOAc 10/1).

^{1}H NMR (400 MHz, $CDCl_3$) δ 7.77 – 7.69 (m, 2H), 7.40 – 7.34 (m, 2H), 7.33 – 7.29 (m, 2H), 7.20 – 7.14 (m, 2H), 7.14 – 7.10 (m, 1H), 6.62 (p, J = 1.8 Hz, 1H), 4.30 (q, J = 2.0 Hz, 2H), 3.66 (q, J = 2.0 Hz, 2H).

^{13}C NMR (100 MHz, $CDCl_3$) δ 169.8, 147.9 (q, J = 1.8 Hz), 140.4, 139.3, 137.0, 135.6, 130.4, 129.1, 124.2, 121.2, 120.5 (q, J = 256.8 Hz), 118.6, 51.1, 31.7.

^{19}F NMR (376 MHz, $CDCl_3$) δ -57.88.

HRMS (ESI-TOF) m/z: $[M + H]^+$ calculated for $C_{18}H_{15}F_3NO_2$ 334.1049; Found 334.1057.

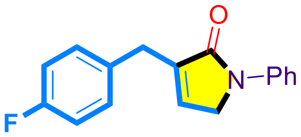

3-(4-Fluorobenzyl)-1-phenyl-1,5-dihydro-2H-pyrrol-2-one (5x):

White solid, 36.3 mg, 68% yield, R_f=0.2 (PE/EtOAc 10/1).

^{1}H NMR (400 MHz, $CDCl_3$) δ 7.76 – 7.70 (m, 2H), 7.37 (dd, J = 8.7, 7.3 Hz, 2H), 7.28 – 7.21 (m, 2H), 7.16 – 7.09 (m, 1H), 7.00 (t, J = 8.7 Hz, 2H), 6.58 (p, J = 2.0 Hz, 1H), 4.30 (q, J = 2.1 Hz, 2H), 3.64 (t, J = 2.1 Hz, 2H).

^{13}C NMR (100 MHz, $CDCl_3$) δ 169.9, 161.7 (d, J = 244.3 Hz), 140.9, 139.3, 135.3, 133.9 (d, J = 3.3 Hz), 130.5 (d, J = 8.1 Hz), 129.1, 124.1, 118.6, 115.4 (d, J = 21.3 Hz), 51.1, 31.6.

^{19}F NMR (376 MHz, $CDCl_3$) δ -116.66.

HRMS (ESI-TOF) m/z: $[M + H]^+$ calculated for $C_{17}H_{15}FNO$ 268.1132; Found 268.1138.

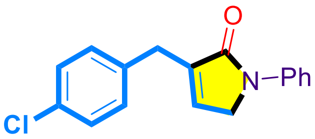

3-(4-Chlorobenzyl)-1-phenyl-1,5-dihydro-2H-pyrrol-2-one (5y):
Slight yellow solid, 35.4 mg, 63% yield, R_f=0.2 ((PE/EtOAc 10/1).
^{1}H NMR (400 MHz, $CDCl_3$) δ 7.77 – 7.70 (m, 2H), 7.37 (dd, J = 8.7, 7.4 Hz, 2H), 7.30 – 7.27 (m, 2H), 7.24 – 7.19 (m, 2H), 7.15 – 7.09 (m, 1H), 6.59 (p, J = 1.8 Hz, 1H), 4.30 (q, J = 2.0 Hz, 2H), 3.63 (q, J = 2.0 Hz, 2H).
^{13}C NMR (100 MHz, $CDCl_3$) δ 169.8, 140.6, 139.3, 136.7, 135.5, 132.4, 130.5, 129.1, 128.8, 124.2, 118.6, 51.1, 31.8.
HRMS (ESI-TOF) m/z: $[M + H]^+$ calculated for $C_{17}H_{15}ClNO$ 284.0837; Found 284.0842.

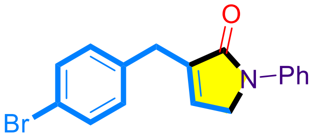

3-(4-Bromobenzyl)-1-phenyl-1,5-dihydro-2H-pyrrol-2-one (5z):
White solid, 26.5 mg, 41% yield, R_f=0.2 ((PE/EtOAc 10/1).
^{1}H NMR (400 MHz, $CDCl_3$) δ 7.77 – 7.70 (m, 2H), 7.47 – 7.42 (m, 2H), 7.40 – 7.34 (m, 2H), 7.20 – 7.15 (m, 2H), 7.15 – 7.11 (m, 1H), 6.60 (p, J = 1.8 Hz, 1H), 4.31 (q, J = 2.0 Hz, 2H), 3.62 (q, J = 2.0 Hz, 2H).
^{13}C NMR (100 MHz, $CDCl_3$) δ 169.8, 140.5, 139.3, 137.2, 135.5, 131.7, 130.9, 129.1, 124.2, 120.4, 118.6, 51.1, 31.9.
HRMS (ESI-TOF) m/z: $[M + H]^+$ calculated for $C_{17}H_{15}BrNO$ 328.0332; Found 328.0327.

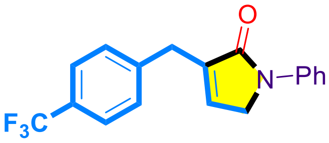

1-Phenyl-3-(4-(trifluoromethyl)benzyl)-1,5-dihydro-2H-pyrrol-2-one (5aa):
White solid, 47.3 mg, 75% yield, R_f=0.2 (PE/EtOAc 10/1).
^{1}H NMR (400 MHz, $CDCl_3$) δ 7.72 (dd, J = 8.7, 1.2 Hz, 2H), 7.57 (d, J = 8.2 Hz, 2H), 7.43 – 7.33 (m, 4H), 7.16 – 7.09 (m, 1H), 6.62 (p, J = 1.8 Hz, 1H), 4.30 (q, J = 2.0 Hz, 2H), 3.72 (s, 2H).
^{13}C NMR (100 MHz, $CDCl_3$) δ 169.7, 142.4 (q, J = 1.21 Hz), 140.0, 139.3, 135.8, 129.4, 129.1, 128.9 (q, J = 32.3 Hz), 125.6 (q, J = 3.9 Hz), 124.2, 121.6 (q, J = 271.8 Hz), 118.6, 51.1, 32.2.
^{19}F NMR (376 MHz, $CDCl_3$) δ -62.37.
HRMS (ESI-TOF) m/z: $[M + H]^+$ calculated for $C_{18}H_{15}F_3NO$ 318.1100; Found 318.1099.

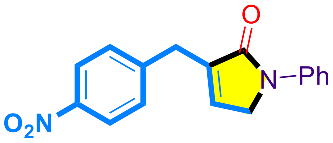

3-(4-Nitrobenzyl)-1-phenyl-1,5-dihydro-2H-pyrrol-2-one (5ab):
Brown solid, 32.3 mg, 55% yield, R_f=0.2 (PE/DCM/EtOAc 10/2/1).
^{1}H NMR (400 MHz, $CDCl_3$) δ 8.17 (d, J = 8.8 Hz, 2H), 7.72 (dd, J = 8.9, 1.2 Hz, 2H), 7.47 (d, J = 8.7 Hz, 2H), 7.37 (dd, J = 8.7, 7.4 Hz, 2H), 7.17 – 7.11 (m, 1H), 6.71 (p, J = 1.8 Hz, 1H), 4.35 (q, J = 1.9 Hz,

2H), 3.78 (q, J = 2.0 Hz, 2H).

^{13}C NMR (100 MHz, $CDCl_3$) δ 169.5, 146.8, 146.0, 139.3, 139.1, 136.2, 129.9, 129.2, 124.4, 123.9, 118.7, 51.2, 32.3.

HRMS (ESI-TOF) m/z: $[M + H]^+$ calculated for $C_{17}H_{15}N_2O_3$ 295.1077; Found 295.1075.

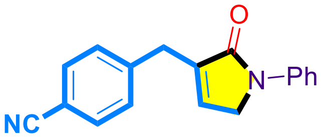

4-((2-Oxo-1-phenyl-2,5-dihydro-1H-pyrrol-3-yl)methyl)benzonitrile (5ac):

White solid, 40.5 mg, 74% yield, R_f=0.2 ((PE/DCM/EtOAc 10/2/1).

^{1}H NMR (400 MHz, $CDCl_3$) δ 7.72 (d, J = 7.6 Hz, 2H), 7.60 (d, J = 8.3 Hz, 2H), 7.41 (d, J = 8.2 Hz, 2H), 7.37 (dd, J = 8.7, 7.4 Hz, 2H), 7.16 – 7.09 (m, 1H), 6.68 (p, J = 1.8 Hz, 1H), 4.33 (q, J = 1.9 Hz, 2H), 3.72 (q, J = 2.0 Hz, 2H).

^{13}C NMR (100 MHz, $CDCl_3$) δ 169.5, 143.9, 139.4, 139.1, 136.1, 132.4, 129.9, 129.2, 124.3, 118.9, 118.6, 110.5, 51.2, 32.5.

HRMS (ESI-TOF) m/z: $[M + H]^+$ calculated for $C_{18}H_{15}N_2O$ 275.1179; Found 275.1176.

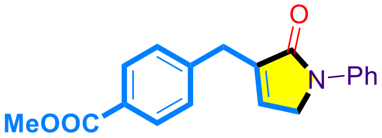

Methyl 4-((2-oxo-1-phenyl-2,5-dihydro-1H-pyrrol-3-yl)methyl)benzoate (5ad):

White solid, 45.3 mg, 74% yield, R_f=0.2 (PE/EtOAc 5/1).

^{1}H NMR (400 MHz, $CDCl_3$) δ 7.99 (d, J = 8.4 Hz, 2H), 7.72 (dd, J = 8.9, 1.2 Hz, 2H), 7.40 – 7.33 (m, 4H), 7.16 – 7.08 (m, 1H), 6.59 (p, J = 1.8 Hz, 1H), 4.29 (q, J = 2.0 Hz, 2H), 3.90 (s, 3H), 3.71 (q, J = 2.1 Hz, 2H).

^{13}C NMR (100 MHz, $CDCl_3$) δ 169.7, 167.0, 143.7, 140.1, 139.3, 135.8, 130.0, 129.12, 129.14, 128.5, 124.2, 118.6, 52.1, 51.1, 32.4.

HRMS (ESI-TOF) m/z: $[M + H]^+$ calculated for $C_{19}H_{18}NO_3$ 308.1281; Found 308.1280.

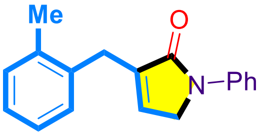

3-(2-Methylbenzyl)-1-phenyl-1,5-dihydro-2H-pyrrol-2-one (5ae):

Light yellow solid, 22.6 mg, 43% yield, R_f=0.2 (PE/EtOAc 10/1).

^{1}H NMR (400 MHz, $CDCl_3$) δ 7.78 – 7.72 (m, 2H), 7.37 (dd, J = 8.8, 7.5 Hz, 2H), 7.23 – 7.15 (m, 4H), 7.15 – 7.09 (m, 1H), 6.38 (p, J = 2.0 Hz, 1H), 4.27 (q, J = 2.3 Hz, 2H), 3.65 (q, J = 2.3 Hz, 2H), 2.29 (s, 3H).

^{13}C NMR (100 MHz, $CDCl_3$) δ 170.1, 140.4, 139.4, 136.48, 136.46, 135.2, 130.4, 130.0, 129.1, 126.8, 126.2, 124.1, 118.5, 51.1, 30.1, 19.4.

HRMS (ESI-TOF) m/z: $[M + H]^+$ calculated for $C_{18}H_{18}NO$ 264.1383; Found 264.1388.

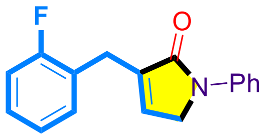

3-(2-Fluorobenzyl)-1-phenyl-1,5-dihydro-2H-pyrrol-2-one (5af):
White solid, 32.4 mg, 61% yield, R_f=0.2 (PE/EtOAc 10/1).
^{1}H NMR (400 MHz, $CDCl_3$) δ 7.80 – 7.68 (m, 2H), 7.34 (dtd, J = 15.7, 7.5, 2.1 Hz, 3H), 7.26 – 7.20 (m, 1H), 7.14 – 7.03 (m, 3H), 6.64 – 6.57 (m, 1H), 4.28 (q, J = 2.1 Hz, 2H), 3.69 (p, J = 2.0 Hz, 2H).
^{13}C NMR (100 MHz, $CDCl_3$) δ 169.8, 161.1 (d, J = 245.8 Hz), 139.37, 139.35, 135.5, 131.5 (d, J = 4.4 Hz), 129.1, 128.5 (d, J = 8.1 Hz), 125.2 (d, J = 15.8 Hz), 124.3 (d, J = 3.7 Hz), 124.1, 118.6, 115.4 (d, J = 22.0 Hz), 51.1, 25.8 (d, J = 3.3 Hz).
^{19}F NMR (376 MHz, $CDCl_3$) δ -117.84.
HRMS (ESI-TOF) m/z: $[M + H]^+$ calculated for $C_{17}H_{15}FNO$ 268.1132; Found 268.1135.

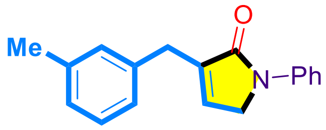

3-(3-Methylbenzyl)-1-phenyl-1,5-dihydro-2H-pyrrol-2-one (5ag):
White solid, 37.1 mg, 71% yield, R_f=0.2 ((PE/EtOAc 10/1).
^{1}H NMR (400 MHz, $CDCl_3$) δ 7.77 – 7.71 (m, 2H), 7.40 – 7.33 (m, 2H), 7.24 – 7.18 (m, 1H), 7.14 – 7.03 (m, 4H), 6.57 (p, J = 1.8 Hz, 1H), 4.27 (q, J = 2.1 Hz, 2H), 3.62 (q, J = 2.1 Hz, 2H), 2.34 (s, 3H).
^{13}C NMR (100 MHz, $CDCl_3$) δ 170.1, 141.1, 139.5, 138.20, 138.24, 135.3, 129.9, 129.1, 128.5, 127.3, 126.1, 124.0, 118.5, 51.1, 32.3, 21.4.
HRMS (ESI-TOF) m/z: $[M + H]^+$ calculated for $C_{18}H_{18}NO$ 264.1383; Found 264.1387.

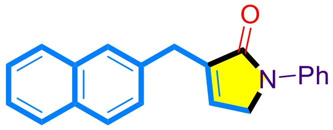

3-(Naphthalen-2-ylmethyl)-1-phenyl-1,5-dihydro-2H-pyrrol-2-one (5ah):
White solid, 40.9 mg, 68% yield, R_f=0.2 (PE/EtOAc 10/1).
^{1}H NMR (400 MHz, $CDCl_3$) δ 7.80 (dt, J = 9.3, 4.0 Hz, 3H), 7.76 – 7.70 (m, 3H), 7.44 (ddd, J = 6.8, 4.2, 1.8 Hz, 2H), 7.42 – 7.38 (m, 1H), 7.38 – 7.32 (m, 2H), 7.11 (dd, J = 8.0, 6.8 Hz, 1H), 6.54 (p, J = 2.0 Hz, 1H), 4.24 (q, J = 2.1 Hz, 2H), 3.81 (q, J = 2.2 Hz, 2H).
^{13}C NMR (100 MHz, $CDCl_3$) δ 170.0, 141.0, 139.4, 135.8, 135.6, 133.7, 132.3, 129.1, 128.3, 127.7, 127.6, 127.5, 126.1, 125.6, 124.1, 118.6, 51.1, 32.5.
HRMS (ESI-TOF) m/z: $[M + H]^+$ calculated for $C_{21}H_{18}NO$ 300.1383; Found 300.1386.

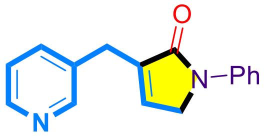

1-Phenyl-3-(pyridin-3-ylmethyl)-1,5-dihydro-2H-pyrrol-2-one (5ai):
White solid, 32.2 mg, 64% yield, R_f=0.2 (PE/EtOAc 1/1).
^{1}H NMR (400 MHz, $CDCl_3$) δ 8.55 (d, J = 2.6 Hz, 1H), 8.50 (dd, J = 4.8, 1.8 Hz, 1H), 7.76 – 7.69 (m, 2H), 7.65 (dt, J = 7.8, 2.1 Hz, 1H), 7.41 – 7.34 (m, 2H), 7.28 – 7.22 (m, 1H), 7.17 – 7.09 (m, 1H), 6.64

(p, J = 1.8 Hz, 1H), 4.32 (q, J = 2.1 Hz, 2H), 3.67 (q, J = 2.1 Hz, 2H).

^{13}C NMR (100 MHz, $CDCl_3$) δ169.6, 150.2, 148.0, 140.0, 139.2, 136.7, 135.8, 133.8, 129.1, 124.2, 123.5, 118.6, 51.1, 29.6.

HRMS (ESI-TOF) m/z: $[M + H]^+$ calculated for $C_{16}H_{15}N_2O$ 251.1179; Found 251.1182.

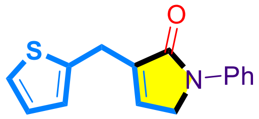

1-Phenyl-3-(thiophen-2-ylmethyl)-1,5-dihydro-2H-pyrrol-2-one (5aj):

White solid, 17.6 mg, 35% yield, R_f=0.2 ((PE/EtOAc 10/1).

^{1}H NMR (400 MHz, $CDCl_3$) δ 7.77 – 7.70 (m, 2H), 7.37 (dd, J = 8.7, 7.4 Hz, 2H), 7.18 (dd, J = 5.1, 1.3 Hz, 1H), 7.16 – 7.09 (m, 1H), 7.00 – 6.91 (m, 2H), 6.76 (p, J = 1.8 Hz, 1H), 4.33 (q, J = 2.1 Hz, 2H), 3.88 (q, J = 2.1 Hz, 2H).

^{13}C NMR (100 MHz, $CDCl_3$) δ 169.6, 140.3, 140.0, 139.3, 135.6, 129.1, 127.0, 126.1, 124.2, 124.1, 118.6, 51.1, 26.4.

HRMS (ESI-TOF) m/z: $[M + H]^+$ calculated for $C_{15}H_{14}NOS$ 256.0791; Found 256.0798.

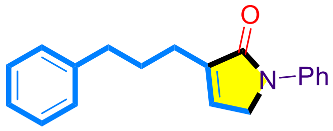

1-Phenyl-3-(3-phenylpropyl)-1,5-dihydro-2H-pyrrol-2-one (5ak):

Light yellow solid, 41.9 mg, 76% yield, R_f=0.2 (PE/DCM 1/2).

^{1}H NMR (700 MHz, $CDCl_3$) δ 7.77 – 7.71 (m, 2H), 7.37 (dd, J = 8.7, 7.3 Hz, 2H), 7.28 (t, J = 7.6 Hz, 2H), 7.23 – 7.20 (m, 2H), 7.20 – 7.16 (m, 1H), 7.14 – 7.10 (m, 1H), 6.75 (q, J = 1.9 Hz, 1H), 4.30 (q, J = 2.0 Hz, 2H), 2.71 (t, J = 7.7 Hz, 2H), 2.42 – 2.38 (m, 2H), 1.94 (p, J = 7.7 Hz, 2H).

^{13}C NMR (176 MHz, $CDCl_3$) δ 170.4, 142.0, 141.1, 139.5, 134.0, 129.1, 128.5, 128.4, 125.9, 124.0, 118.5, 51.0, 35.6, 29.2, 25.5.

HRMS (ESI-TOF) m/z: $[M + H]^+$ calculated for $C_{19}H_{20}NO$ 278.1539; Found 278.1548.

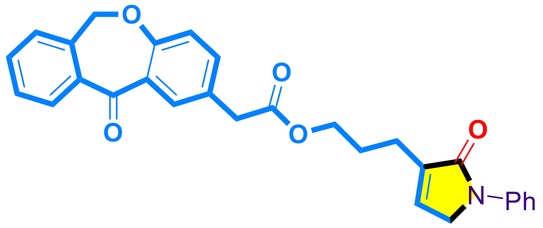

3-(2-Oxo-1-phenyl-2,5-dihydro-1H-pyrrol-3-yl)propyl 2-(11-oxo-6,11-dihydrodibenzo[b,e]oxepin-2-yl)acetate (5am):

Brown oil, 18.1 mg, 39% yield, R_f=0.2 (PE/EtOAc 2/1).

^{1}H NMR (700 MHz, $CDCl_3$) δ 8.13 (d, J = 2.5 Hz, 1H), 7.88 (dd, J = 7.7, 1.5 Hz, 1H), 7.74 – 7.71 (m, 2H), 7.56 (td, J = 7.4, 1.4 Hz, 1H), 7.46 (td, J = 7.5, 1.3 Hz, 1H), 7.44 (dd, J = 8.4, 2.5 Hz, 1H), 7.37 (tt, J = 7.3, 2.0 Hz, 3H), 7.12 (tt, J = 7.4, 1.2 Hz, 1H), 7.04 (d, J = 8.4 Hz, 1H), 6.78 (p, J = 1.8 Hz, 1H), 5.19 (s, 2H), 4.31 (q, J = 2.0 Hz, 2H), 4.17 (t, J = 6.5 Hz, 2H), 3.66 (s, 2H), 2.41 (tq, J = 8.6, 1.8 Hz, 2H), 1.99 – 1.94 (m, 2H).

^{13}C NMR (176 MHz, $CDCl_3$) δ 190.9, 171.5, 170.2, 160.5, 140.4, 139.9, 139.4, 136.4, 135.6, 134.7, 132.8, 132.4, 129.5, 129.3, 129.1, 127.9, 127.8, 125.2, 124.1, 121.1, 118.6, 73.6, 64.2, 51.1, 40.3, 26.3, 22.4.

HRMS (ESI-TOF) m/z: $[M + H]^+$ calculated for $C_{29}H_{26}NO_5$ 468.1805; Found 468.1806.

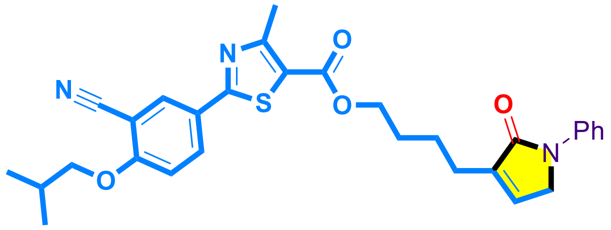

4-(2-Oxo-1-phenyl-2,5-dihydro-1H-pyrrol-3-yl)butyl 2-(3-cyano-4-isobutoxyphenyl)-4-methylthiazole-5-carboxylate (5an):

White solid, 21.6 mg, 41% yield, R_f=0.2 (PE/ EtOAc 2/1).

^{1}H NMR (700 MHz, $CDCl_3$) δ 8.17 (d, J = 2.4 Hz, 1H), 8.09 (dd, J = 8.8, 2.3 Hz, 1H), 7.79 – 7.68 (m, 2H), 7.37 (dd, J = 8.7, 7.4 Hz, 2H), 7.12 (tt, J = 7.3, 1.2 Hz, 1H), 7.00 (d, J = 8.8 Hz, 1H), 6.81 (p, J = 1.8 Hz, 1H), 4.36 – 4.32 (m, 4H), 3.90 (d, J = 6.6 Hz, 2H), 2.76 (s, 3H), 2.47 – 2.41 (m, 2H), 2.20 (dt, J = 13.3, 6.7 Hz, 1H), 1.87 – 1.83 (m, 2H), 1.79 – 1.74 (m, 2H), 1.09 (d, J = 6.8 Hz, 6H).

^{13}C NMR (176 MHz, $CDCl_3$) δ 170.3, 167.2, 162.5, 162.1, 161.2, 140.7, 139.4, 134.3, 132.6, 132.1, 129.1, 126.0, 124.0, 121.8, 118.5, 115.4, 112.6, 102.9, 75.7, 65.1, 51.1, 28.4, 28.2, 25.5, 24.1, 19.1, 17.5.

HRMS (ESI-TOF) m/z: $[M + H]^+$ calculated for $C_{30}H_{32}N_3O_4S$ 530.2108; Found 530.2106.

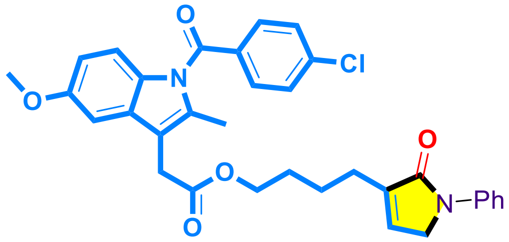

4-(2-Oxo-1-phenyl-2,5-dihydro-1H-pyrrol-3-yl)butyl 2-(1-(4-chlorobenzoyl)-5-methoxy-2-methyl-1H-indol-3-yl)acetate (5ao):

Yellow solid, 26.3 mg, 46% yield, R_f=0.2 (PE/ EtOAc 2/1).

^{1}H NMR (700 MHz, $CDCl_3$) δ 7.76 – 7.69 (m, 2H), 7.64 (d, J = 8.5 Hz, 2H), 7.45 (d, J = 8.5 Hz, 2H), 7.37 (dd, J = 8.8, 7.4 Hz, 2H), 7.12 (tt, J = 7.4, 1.2 Hz, 1H), 6.97 (d, J = 2.6 Hz, 1H), 6.88 (d, J = 9.0 Hz, 1H), 6.66 (dd, J = 9.0, 2.6 Hz, 1H), 6.62 (p, J = 1.8 Hz, 1H), 4.28 (q, J = 2.0 Hz, 2H), 4.15 (t, J = 6.5 Hz, 2H), 3.82 (s, 3H), 3.67 (s, 2H), 2.38 (s, 3H), 2.31 (tt, J = 6.8, 1.8 Hz, 2H), 1.72 – 1.68 (m, 2H), 1.61 – 1.56 (m, 2H).

^{13}C NMR (176 MHz, $CDCl_3$) δ 170.9, 170.3, 168.3, 156.0, 140.6, 139.4, 139.3, 135.9, 134.2, 133.9, 131.1, 130.8, 130.7, 129.1, 129.1, 124.0, 118.5, 114.9, 112.7, 111.7, 101.3, 64.7, 55.7, 51.0, 30.4, 28.3, 25.3, 23.9, 13.4.

HRMS (ESI-TOF) m/z: $[M + H]^+$ calculated for $C_{33}H_{32}ClN_2O_5$ 571.1994; Found 571.2012.

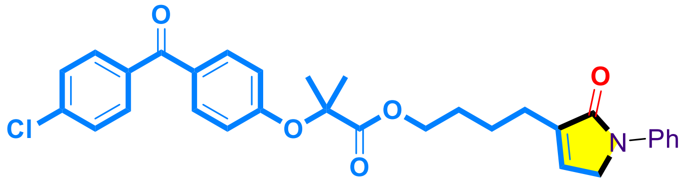

4-(2-Oxo-1-phenyl-2,5-dihydro-1H-pyrrol-3-yl)butyl 2-(4-(4-chlorobenzoyl)phenoxy)-2-methylpropanoate (5ap):

Brown solid, 24.3 mg, 46% yield, R_f=0.2 (PE/ EtOAc 2/1).

^{1}H NMR (700 MHz, $CDCl_3$) δ 7.75 – 7.68 (m, 4H), 7.66 (d, J = 8.5 Hz, 2H), 7.41 (d, J = 8.5 Hz, 2H),

7.36 (t, J = 8.0 Hz, 2H), 7.12 (t, J = 7.4 Hz, 1H), 6.86 (d, J = 8.8 Hz, 2H), 6.62 (s, 1H), 4.30 – 4.23 (m, 2H), 4.19 (t, J = 6.4 Hz, 2H), 2.30 – 2.23 (m, 2H), 1.69 (s, 8H), 1.48 (p, J = 7.8 Hz, 2H).
^{13}C NMR (176 MHz, $CDCl_3$) δ 194.2, 173.8, 170.2, 159.8, 140.5, 139.4, 138.5, 136.2, 134.3, 132.0, 131.1, 130.2, 129.1, 128.6, 124.0, 118.5, 117.0, 79.4, 65.5, 51.0, 28.1, 25.5, 25.3, 23.8.
HRMS (ESI-TOF) m/z: $[M + H]^+$ calculated for $C_{31}H_{31}ClNO_5$ 532.1885; Found 532.1882.

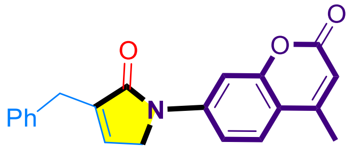

3-Benzyl-1-(4-methyl-2-oxo-2H-chromen-7-yl)-1,5-dihydro-2H-pyrrol-2-one (5ar):
Yellow solid, 11.8 mg, 36% yield, R_f=0.2 (PE/DCM/EtOAc 2/2/1).
^{1}H NMR (700 MHz, $CDCl_3$) δ 8.03 (dd, J = 8.8, 2.3 Hz, 1H), 7.58 (d, J = 8.8 Hz, 1H), 7.53 (d, J = 2.3 Hz, 1H), 7.34 (t, J = 7.5 Hz, 2H), 7.30 – 7.26 (m, 3H), 6.66 (t, J = 1.9 Hz, 1H), 6.21 (t, J = 1.3 Hz, 1H), 4.33 (q, J = 2.2 Hz, 2H), 3.68 (q, J = 2.1 Hz, 2H), 2.42 (d, J = 1.4 Hz, 3H).
^{13}C NMR (176 MHz, $CDCl_3$) δ 170.3, 161.0, 154.4, 152.1, 142.5, 141.1, 137.8, 136.0, 129.1, 128.7, 126.7, 125.4, 115.8, 114.1, 113.4, 105.1, 50.9, 32.3, 18.6.
HRMS (ESI-TOF) m/z: $[M + H]^+$ calculated for $C_{21}H_{18}NO_3$ 332.1281; Found 332.1282.

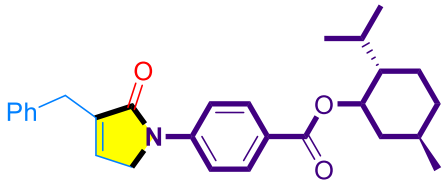

(1R,5R)-2-isopropyl-5-methylcyclohexyl 4-(3-benzyl-2-oxo-2,5-dihydro-1H-pyrrol-1-yl)benzoate (5as):
White solid, 15.3 mg, 35% yield, R_f=0.2 (PE/EtOAc 10/1).
^{1}H NMR (700 MHz, $CDCl_3$) δ 8.05 (d, J = 8.8 Hz, 2H), 7.84 (d, J = 8.9 Hz, 2H), 7.33 (t, J = 7.6 Hz, 2H), 7.30 – 7.26 (m, 2H), 7.27 – 7.23 (m, 1H), 6.63 (t, J = 1.9 Hz, 1H), 4.92 (dt, J = 10.9, 5.4 Hz, 1H), 4.32 (t, J = 2.3 Hz, 2H), 3.66 (d, J = 2.0 Hz, 2H), 2.12 (dt, J = 12.2, 2.6 Hz, 1H), 1.98 – 1.91 (m, 2H), 1.73 (dt, J = 11.6, 3.0 Hz, 2H), 1.59 – 1.53 (m, 2H), 1.14 – 1.08 (m, 2H), 0.92 (d, J = 7.3 Hz, 6H), 0.79 (d, J = 7.0 Hz, 3H).
^{13}C NMR (176 MHz, $CDCl_3$) δ 170.3, 165.7, 143.1, 141.1, 138.0, 135.9, 130.8, 129.1, 128.7, 126.6, 125.9, 117.2, 74.7, 50.9, 47.3, 41.0, 34.3, 32.3, 31.5, 26.5, 23.7, 22.1, 20.8, 16.6.
HRMS (ESI-TOF) m/z: $[M + H]^+$ calculated for $C_{28}H_{34}NO_3$ 432.2533; Found 432.2541.

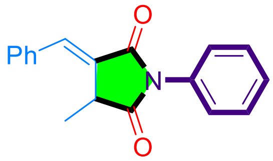

***E*-3-Benzylidene-4-methyl-1-phenylpyrrolidine-2,5-dione (6a)**:
White solid, 38.8 mg, 70% yield, R_f=0.2 (PE/EtOAc 10/1).
^{1}H NMR (400 MHz, $CDCl_3$) δ 7.74 (d, J = 1.8 Hz, 1H), 7.56 – 7.45 (m, 6H), 7.44 – 7.37 (m, 4H), 3.95 (qd, J = 7.3, 2.0 Hz, 1H), 1.51 (d, J = 7.3 Hz, 3H).
^{13}C NMR (100 MHz, $CDCl_3$) δ 177.3, 169.6, 135.8, 133.5, 132.0, 130.2, 130.2, 129.4, 129.2, 129.1, 128.6, 126.5, 38.4, 14.6.
HRMS (ESI-TOF) m/z: $[M + H]^+$ calculated for $C_{18}H_{16}NO_2$ 278.1176; Found 278.1182.

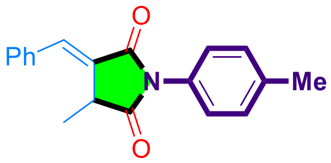

***E*-3-benzylidene-4-methyl-1-(p-tolyl)pyrrolidine-2,5-dione (6b)**:
Light yellow solid, 47.2 mg, 81% yield, R_f=0.2 (PE/DCM/EtOAc 10/2/1).
^{1}H NMR (400 MHz, $CDCl_3$) δ 7.72 (d, J = 2.3 Hz, 1H), 7.56 – 7.50 (m, 2H), 7.49 – 7.39 (m, 3H), 7.31 – 7.24 (m, 4H), 3.93 (qd, J = 7.3, 2.3 Hz, 1H), 2.39 (s, 3H), 1.49 (d, J = 7.3 Hz, 3H).
^{13}C NMR (100 MHz, $CDCl_3$) δ 177.5, 169.8, 138.6, 135.6, 133.6, 130.2, 130.1, 129.8, 129.5, 129.4, 129.1, 126.3, 38.4, 21.3, 14.6.
HRMS (ESI-TOF) m/z: $[M + H]^+$ calculated for $C_{19}H_{18}NO_2$ 292.1332; Found 292.1331.

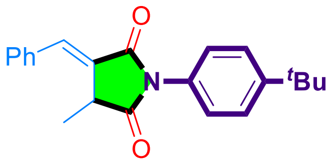

***E*-3-benzylidene-1-(4-(tert-butyl)phenyl)-4-methylpyrrolidine-2,5-dione (6c)**:
White solid, 48.9 mg, 73% yield, R_f=0.2 (PE/EtOAc 9/1).
^{1}H NMR (400 MHz, $CDCl_3$) δ 7.73 (d, J = 2.3 Hz, 1H), 7.56 – 7.49 (m, 4H), 7.48 – 7.41 (m, 3H), 7.34 – 7.29 (m, 2H), 3.94 (qd, J = 7.3, 2.2 Hz, 1H), 1.50 (d, J = 7.3 Hz, 3H), 1.34 (s, 9H).
^{13}C NMR (100 MHz, $CDCl_3$) δ 177.5, 169.8, 151.5, 135.6, 133.6, 130.2, 130.1, 129.5, 129.3, 129.1, 126.2, 125.9, 38.4, 34.8, 31.3, 14.6.
HRMS (ESI-TOF) m/z: $[M + H]^+$ calculated for $C_{22}H_{24}NO_2$ 334.1802; Found 334.1815.

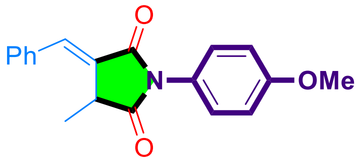

***E*-3-benzylidene-1-(4-methoxyphenyl)-4-methylpyrrolidine-2,5-dione (6d)**:
Yellow solid, 45.4 mg, 74% yield, R_f=0.2 (PE/EtOAc 5/1).
^{1}H NMR (400 MHz, $CDCl_3$) δ 7.72 (d, J = 2.2 Hz, 1H), 7.56 – 7.50 (m, 2H), 7.50 – 7.40 (m, 3H), 7.32 – 7.28 (m, 2H), 7.03 – 6.98 (m, 2H), 3.94 (td, J = 7.3, 2.3 Hz, 1H), 3.83 (s, 3H), 1.49 (d, J = 7.3 Hz, 3H).
^{13}C NMR (100 MHz, $CDCl_3$) δ 177.6, 169.9, 159.5, 135.6, 133.6, 130.2, 130.1, 129.5, 129.1, 127.7, 124.7, 114.5, 55.5, 38.3, 14.6.
HRMS (ESI-TOF) m/z: $[M + H]^+$ calculated for $C_{19}H_{18}NO_3$ 308.1281; Found 308.1290.

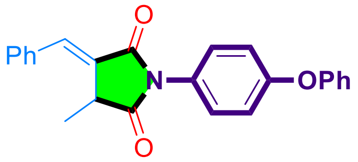

***E*-3-benzylidene-4-methyl-1-(4-phenoxyphenyl)pyrrolidine-2,5-dione (6e)**:
White solid, 62.0 mg, 84% yield, R_f=0.2 (PE/EtOAc 5/1).
^{1}H NMR (400 MHz, $CDCl_3$) δ 7.73 (d, J = 2.3 Hz, 1H), 7.57 – 7.50 (m, 2H), 7.49 – 7.40 (m, 3H), 7.39 – 7.32 (m, 4H), 7.18 – 7.12 (m, 1H), 7.08 (td, J = 8.4, 7.8, 1.6 Hz, 4H), 3.94 (qd, J = 7.3, 2.2 Hz, 1H), 1.50 (d, J = 7.3 Hz, 3H).
^{13}C NMR (100 MHz, $CDCl_3$) δ 177.4, 169.7, 157.5, 156.4, 135.8, 133.5, 130.2, 130.2, 130.0, 129.3, 129.1, 127.9, 126.7, 124.0, 119.6, 118.8, 38.4, 14.6.

HRMS (ESI-TOF) m/z: $[M + H]^+$ calculated for $C_{24}H_{20}NO_3$ 370.1438; Found 370.1445.

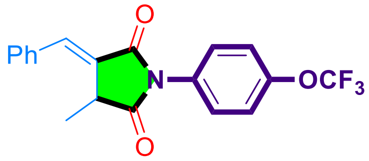

***E*-3-benzylidene-4-methyl-1-(4-(trifluoromethoxy)phenyl)pyrrolidine-2,5-dione (6f)**:
White solid, 54.2 mg, 75% yield, (PE/EtOAc 5/1).
^{1}H NMR (400 MHz, $CDCl_3$) δ 7.75 (d, J = 2.3 Hz, 1H), 7.59 – 7.51 (m, 2H), 7.46 (td, J = 8.6, 8.2, 5.6 Hz, 5H), 7.34 (d, J = 8.8 Hz, 2H), 3.97 (qd, J = 7.3, 2.3 Hz, 1H), 1.51 (d, J = 7.3 Hz, 3H).
^{13}C NMR (100 MHz, $CDCl_3$) δ 177.1, 169.3, 148.7 (q, J = 1.8 Hz), 136.3, 133.4, 130.5, 130.3, 130.3, 129.1, 128.9, 127.9, 121.6, 120.4 (q, J = 257.9 Hz), 38.4, 14.6.
^{19}F NMR (376 MHz, $CDCl_3$) δ -57.82.
HRMS (ESI-TOF) m/z: $[M + H]^+$ calculated for $C_{19}H_{15}F_3NO_3$ 362.0999; Found 362.1013.

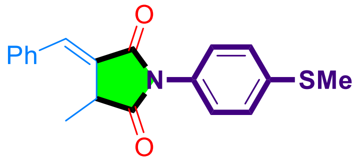

***E*-3-benzylidene-4-methyl-1-(4-(trimethyl-l4-sulfaneyl)phenyl)pyrrolidine-2,5-dione (6g)**:
Yellow solid, 43.9 mg, 68% yield, R_f=0.2 (PE/EtOAc 5/1).
^{1}H NMR (700 MHz, $CDCl_3$) δ 7.73 (d, J = 2.3 Hz, 1H), 7.56 – 7.51 (m, 2H), 7.50 – 7.45 (m, 2H), 7.45 – 7.42 (m, 1H), 7.38 – 7.34 (m, 2H), 7.34 – 7.30 (m, 2H), 3.94 (qd, J = 7.4, 2.3 Hz, 1H), 2.51 (s, 3H), 1.50 (d, J = 7.4 Hz, 3H).
^{13}C NMR (176 MHz, $CDCl_3$) δ 177.3, 169.6, 139.5, 135.9, 133.5, 130.2, 130.2, 129.3, 129.1, 128.9, 126.8, 126.7, 38.4, 15.8, 14.6.
HRMS (ESI-TOF) m/z: $[M + H]^+$ calculated for $C_{19}H_{18}NO_2S$ 324.1053; Found 324.1063.

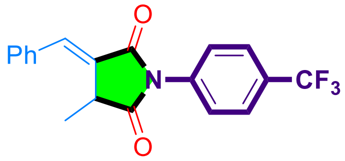

***E*-3-benzylidene-4-methyl-1-(4-(trifluoromethyl)phenyl)pyrrolidine-2,5-dione (6h)**:
White solid, 15.9 mg, 46% yield, R_f=0.2 (PE/EtOAc 10/1). **6h**:**6h'** = 13:1
^{1}H NMR (400 MHz, $CDCl_3$) δ 7.81 – 7.71 (m, 3H), 7.59 (d, J = 8.6 Hz, 2H), 7.57 – 7.52 (m, 2H), 7.52 – 7.41 (m, 3H), 3.98 (qd, J = 7.4, 2.3 Hz, 1H), 1.52 (d, J = 7.3 Hz, 3H).
^{13}C NMR (100 MHz, $CDCl_3$) δ 176.9, 169.1, 136.6, 135.2 (q, J = 1.20 Hz), 133.3, 130.4, 130.4 (q, J = 33.0 Hz), 130.3, 129.2, 128.8, 126.6, 126.2 (q, J = 3.7 Hz), 123.8 (q, J = 272.2 Hz), 38.5, 14.6.
^{19}F NMR (376 MHz, $CDCl_3$) δ -62.67.
HRMS (ESI-TOF) m/z: $[M + H]^+$ calculated for $C_{19}H_{15}F_3NO_2$ 346.1049; Found 346.1055.

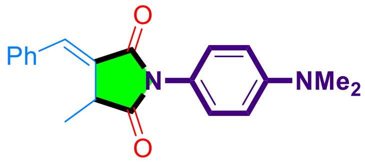

***E*-3-benzylidene-1-(4-(dimethylamino)phenyl)-4-methylpyrrolidine-2,5-dione (6i)**:
Brown solid, 33.9 mg, 53% yield, R_f=0.2 (PE/EtOAc 5/1).
^{1}H NMR (700 MHz, $CDCl_3$) δ 7.71 (d, J = 2.4 Hz, 1H), 7.56 – 7.50 (m, 2H), 7.45 (t, J = 7.6 Hz, 2H),

7.41 (t, J = 7.3 Hz, 1H), 7.24 – 7.19 (m, 2H), 6.80 – 6.76 (m, 2H), 3.91 (qd, J = 7.4, 2.4 Hz, 1H), 2.98 (s, 6H), 1.48 (d, J = 7.4 Hz, 3H).

^{13}C NMR (176 MHz, $CDCl_3$) δ 177.9, 170.2, 150.4, 135.1, 133.7, 130.2, 130.0, 129.8, 129.0, 127.1, 120.5, 112.4, 40.5, 38.3, 14.6.

HRMS (ESI-TOF) m/z: $[M + H]^+$ calculated for $C_{21}H_{25}N_2O_2$ 337.1911; Found 337.1916.

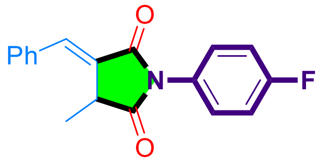

***E*-3-benzylidene-1-(4-fluorophenyl)-4-methylpyrrolidine-2,5-dione (6j)**:

Yellow solid, 42.1 mg, 71% yield, R_f=0.2 (PE/EtOAc 10/1).

^{1}H NMR (400 MHz, $CDCl_3$) δ 7.74 (d, J = 2.2 Hz, 1H), 7.57 – 7.51 (m, 2H), 7.51 – 7.42 (m, 3H), 7.41 – 7.36 (m, 2H), 7.22 – 7.14 (m, 2H), 3.95 (qd, J = 7.3, 2.2 Hz, 1H), 1.50 (d, J = 7.3 Hz, 3H).

^{13}C NMR (100 MHz, $CDCl_3$) δ 177.3, 169.5, 162.1 (d, J = 248.7 Hz), 136.0, 133.4, 130.26, 130.24, 129.1, 128.3 (d, J = 8.8 Hz), 127.9 (d, J = 3.3 Hz), 116.2 (d, J = 23.1 Hz), 38.4, 14.6.

HRMS (ESI-TOF) m/z: $[M + H]^+$ calculated for $C_{18}H_{15}FNO_2$ 296.1081; Found 296.1099.

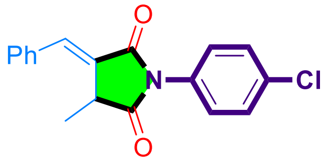

***E*-3-benzylidene-1-(4-chlorophenyl)-4-methylpyrrolidine-2,5-dione (6k)**:

Yellow solid, 42.5 mg, 68% yield, R_f=0.2 (PE/EtOAc 10/1).

^{1}H NMR (700 MHz, $CDCl_3$) δ 7.73 (d, J = 2.4 Hz, 1H), 7.55 – 7.51 (m, 2H), 7.49 – 7.41 (m, 5H), 7.39 – 7.35 (m, 2H), 3.94 (qd, J = 7.4, 2.3 Hz, 1H), 1.50 (d, J = 7.4 Hz, 3H).

^{13}C NMR (176 MHz, $CDCl_3$) δ 177.0, 169.3, 136.2, 134.3, 133.4, 130.5, 130.3, 130.3, 129.3, 129.1, 129.0, 127.6, 38.4, 14.6.

HRMS (ESI-TOF) m/z: $[M + H]^+$ calculated for $C_{18}H_{15}ClNO_2$ 312.0786; Found 312.0792.

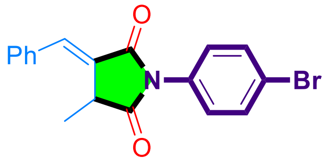

***E*-3-benzylidene-1-(4-bromophenyl)-4-methylpyrrolidine-2,5-dione (6l)**:

White solid, 40.2 mg,57% yield, R_f=0.2 (PE/EtOAc 10/1).

^{1}H NMR (700 MHz, $CDCl_3$) δ 7.73 (d, J = 2.4 Hz, 1H), 7.65 – 7.60 (m, 2H), 7.56 – 7.51 (m, 2H), 7.50 – 7.45 (m, 2H), 7.45 – 7.40 (m, 1H), 7.33 – 7.28 (m, 2H), 3.94 (qd, J = 7.4, 2.3 Hz, 1H), 1.49 (d, J = 7.4 Hz, 3H).

^{13}C NMR (176 MHz, $CDCl_3$) δ 177.0, 169.3, 136.2, 133.4, 132.3, 131.1, 130.3, 130.3, 129.1, 129.0, 127.9, 122.3, 38.4, 14.6.

HRMS (ESI-TOF) m/z: $[M + H]^+$ calculated for $C_{18}H_{15}BrNO_2$ 356.0281; Found 356.0273.

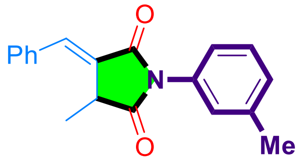

***E*-3-benzylidene-4-methyl-1-(m-tolyl)pyrrolidine-2,5-dione (6m)**:

Yellow solid, 48.3 mg, 83% yield, R_f=0.2 (PE/EtOAc 10/1).

^{1}H NMR (700 MHz, $CDCl_3$) δ 7.73 (d, J = 2.3 Hz, 1H), 7.56 – 7.50 (m, 2H), 7.49 – 7.44 (m, 2H), 7.44 – 7.41 (m, 1H), 7.38 (t, J = 7.7 Hz, 1H), 7.22 (d, J = 7.7 Hz, 1H), 7.19 – 7.14 (m, 2H), 3.94 (qd, J = 7.4, 2.3 Hz, 1H), 2.40 (s, 3H), 1.50 (d, J = 7.4 Hz, 3H).

^{13}C NMR (176 MHz, $CDCl_3$) δ 177.4, 169.7, 139.2, 135.7, 133.6, 131.9, 130.2, 130.1, 129.5, 129.5, 129.1, 129.0, 127.1, 123.6, 38.4, 21.4, 14.6.

HRMS (ESI-TOF) m/z: $[M + H]^+$ calculated for $C_{19}H_{18}NO_2$ 292.1332; Found 292.1338.

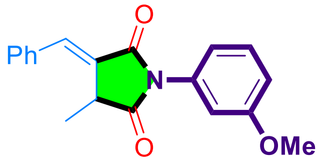

***E*-3-benzylidene-1-(3-methoxyphenyl)-4-methylpyrrolidine-2,5-dione (6n)**:

White solid, 23.3 mg (0.1 mmol), 76% yield, R_f=0.2 (PE/EtOAc 5/1).

^{1}H NMR (400 MHz, $CDCl_3$) δ 7.74 (d, J = 2.2 Hz, 1H), 7.57 – 7.51 (m, 2H), 7.50 – 7.37 (m, 4H), 7.02 – 6.89 (m, 3H), 3.95 (qd, J = 7.3, 2.3 Hz, 1H), 3.83 (s, 3H), 1.51 (d, J = 7.3 Hz, 3H).

^{13}C NMR (100 MHz, $CDCl_3$) δ 177.2, 169.6, 160.1, 135.8, 133.5, 133.0, 130.2, 130.2, 129.9, 129.4, 129.1, 118.7, 114.6, 112.2, 55.5, 38.4, 14.6.

HRMS (ESI-TOF) m/z: $[M + H]^+$ calculated for $C_{19}H_{18}NO_3$ 308.1281; Found 308.1283.

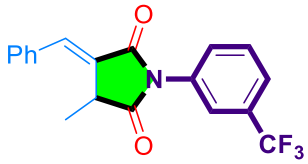

***E*-3-benzylidene-4-methyl-1-(3-(trifluoromethyl)phenyl)pyrrolidine-2,5-dione (6o)**:

White solid, 43.5 mg, 63% yield, R_f=0.2 (PE/EtOAc 10/1).

^{1}H NMR (700 MHz, $CDCl_3$) δ 7.76 (d, J = 2.4 Hz, 1H), 7.73 (d, J = 2.2 Hz, 1H), 7.69 – 7.61 (m, 3H), 7.57 – 7.52 (m, 2H), 7.48 (dd, J = 8.3, 6.5 Hz, 2H), 7.46 – 7.42 (m, 1H), 3.98 (qd, J = 7.4, 2.4 Hz, 1H), 1.52 (d, J = 7.4 Hz, 3H).

^{13}C NMR (176 MHz, $CDCl_3$) δ 176.9, 169.2, 136.5, 133.3, 132.6, 131.6 (q, J = 33.5 Hz), 130.4, 130.3, 129.7, 129.7, 129.2, 128.8, 125.2 (q, J = 3.9 Hz), 123.6 (q, J = 273.1 Hz), 123.5 (q, J = 4.2 Hz), 38.4, 14.6.

HRMS (ESI-TOF) m/z: $[M + H]^+$ calculated for $C_{19}H_{15}F_3NO_2$ 346.1049; Found 346.1049.

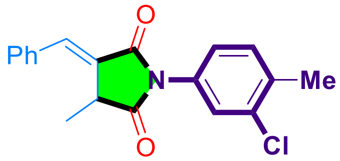

***E*-3-benzylidene-1-(3-chloro-4-methylphenyl)-4-methylpyrrolidine-2,5-dione (6p)**:

Yellow solid, 44.5 mg, 68% yield, R_f=0.2 (PE/EtOAc 10/1).

^{1}H NMR (700 MHz, $CDCl_3$) δ 7.73 (d, J = 2.5 Hz, 1H), 7.53 (d, J = 7.3 Hz, 2H), 7.46 (t, J = 7.4 Hz, 2H), 7.44 – 7.39 (m, 2H), 7.34 (d, J = 8.2 Hz, 1H), 7.21 (dd, J = 8.1, 2.2 Hz, 1H), 3.93 (qd, J = 7.3, 2.3 Hz, 1H), 2.40 (s, 3H), 1.49 (d, J = 7.4 Hz, 3H).

^{13}C NMR (176 MHz, $CDCl_3$) δ 177.1, 169.4, 136.7, 136.1, 134.6, 133.4, 131.2, 130.6, 130.2, 129.1, 127.0, 124.6, 38.4, 19.9, 14.6.

HRMS (ESI-TOF) m/z: $[M + H]^+$ calculated for $C_{19}H_{17}ClNO_2$ 326.0942; Found 326.0939.

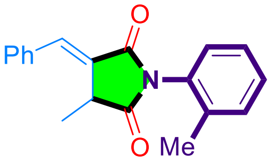

***E*-3-benzylidene-4-methyl-1-(m-tolyl)pyrrolidine-2,5-dione (6q)**:

Brown solid, 19.8 mg, 34% yield, R_f=0.2 (PE/EtOAc 10/1).

^{1}H NMR (700 MHz, $CDCl_3$) δ 7.73 (t, J = 1.9 Hz, 1H), 7.55 (d, J = 7.4 Hz, 2H), 7.47 (dd, J = 8.3, 6.5 Hz, 2H), 7.44 – 7.41 (m, 1H), 7.36 – 7.31 (m, 3H), 7.19 – 7.11 (m, 1H), 3.98 (dqd, J = 14.7, 7.4, 2.3 Hz, 1H), 2.19 (d, J = 15.5 Hz, 3H), 1.53 (dd, J = 7.4, 5.1 Hz, 3H).

^{13}C NMR (176 MHz, $CDCl_3$) δ 177.3, 177.3, 169.7, 169.6, 135.8, 135.7, 135.7, 135.6, 133.5, 133.5, 131.2, 131.2, 131.2, 131.1, 130.2, 130.2, 130.2, 129.7, 129.6, 129.1, 129.1, 128.1, 128.0, 127.0, 127.0, 38.7, 38.6, 17.9, 17.7, 15.0, 14.6.

HRMS (ESI-TOF) m/z: $[M + H]^+$ calculated for $C_{19}H_{18}NO_2$ 292.1332; Found 292.1339.

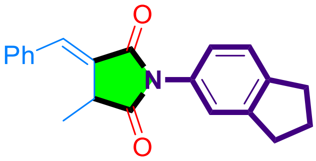

***E*-3-benzylidene-1-(2,3-dihydro-1H-inden-5-yl)-4-methylpyrrolidine-2,5-dione (6r)**:

Brown solid, 49.5 mg, 78% yield, R_f=0.2 (PE/EtOAc 10/1).

^{1}H NMR (700 MHz, $CDCl_3$) δ 7.72 (d, J = 2.4 Hz, 1H), 7.57 – 7.51 (m, 2H), 7.48 – 7.44 (m, 2H), 7.43 – 7.40 (m, 1H), 7.32 (d, J = 7.9 Hz, 1H), 7.19 (d, J = 1.8 Hz, 1H), 7.09 (dd, J = 7.9, 2.2 Hz, 1H), 3.93 (qd, J = 7.3, 2.3 Hz, 1H), 2.94 (dt, J = 15.5, 7.5 Hz, 4H), 2.11 (p, J = 7.5 Hz, 2H), 1.49 (d, J = 7.4 Hz, 3H).

^{13}C NMR (176 MHz, $CDCl_3$) δ 177.6, 170.0, 145.5, 145.1, 135.5, 133.6, 130.2, 130.1, 129.9, 129.6, 129.1, 124.9, 124.4, 122.6, 38.4, 32.9, 32.7, 25.6, 14.6.

HRMS (ESI-TOF) m/z: $[M + H]^+$ calculated for $C_{21}H_{20}NO_2$ 318.1489; Found 318.1490.

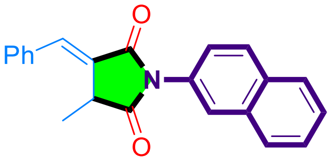

***E*-3-benzylidene-4-methyl-1-(naphthalen-2-yl)pyrrolidine-2,5-dione (6s)**:

Brown solid, 21.3 mg (0.1 mmol), 65% yield, R_f=0.2 (PE/EtOAc 10/1).

^{1}H NMR (400 MHz, $CDCl_3$) δ 7.96 (d, J = 8.7 Hz, 1H), 7.93 – 7.85 (m, 3H), 7.77 (d, J = 2.2 Hz, 1H), 7.59 – 7.51 (m, 4H), 7.51 – 7.42 (m, 4H), 4.00 (qd, J = 7.3, 2.3 Hz, 1H), 1.55 (d, J = 7.3 Hz, 3H).

^{13}C NMR (100 MHz, $CDCl_3$) δ 177.5, 169.8, 135.9, 133.5, 133.2, 132.9, 130.2, 130.2, 129.5, 129.4, 129.1, 129.0, 128.3, 127.8, 126.9, 126.6, 125.6, 123.9, 38.5, 14.6.

HRMS (ESI-TOF) m/z: $[M + H]^+$ calculated for $C_{22}H_{18}NO_2$ 328.1332; Found 328.1329.

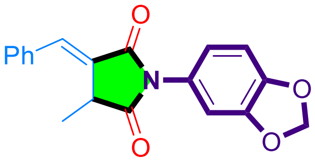

***E*-1-(benzo[d][1,3]dioxol-5-yl)-3-benzylidene-4-methylpyrrolidine-2,5-dione (6t)**:

Yellow solid, 44.9 mg, 70% yield, R_f=0.2 (PE/EtOAc 5/1).

^{1}H NMR (700 MHz, $CDCl_3$) δ 7.72 (d, J = 2.4 Hz, 1H), 7.56 – 7.50 (m, 2H), 7.49 – 7.44 (m, 2H), 7.44

– 7.40 (m, 1H), 6.92 – 6.88 (m, 1H), 6.86 – 6.81 (m, 2H), 6.02 (s, 2H), 3.92 (qd, J = 7.4, 2.3 Hz, 1H), 1.49 (d, J = 7.3 Hz, 3H).

^{13}C NMR (176 MHz, $CDCl_3$) δ 177.4, 169.8, 148.1, 147.8, 135.8, 133.5, 130.2, 130.2, 129.3, 129.1, 125.5, 120.4, 108.4, 107.7, 101.8, 38.3, 14.6.

HRMS (ESI-TOF) m/z: $[M + H]^+$ calculated for $C_{19}H_{16}NO_4$ 322.1074; Found 322.1082.

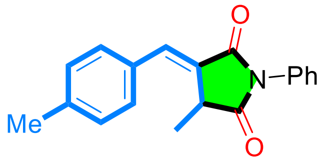

3-Methyl-4-(4-methylbenzylidene)-1-phenylpyrrolidine-2,5-dione (6u):

White solid, 36.1 mg, 62% yield, R_f=0.2 (PE/EtOAc 10/1)

^{1}H NMR (400 MHz, $CDCl_3$) δ 7.70 (d, J = 2.3 Hz, 1H), 7.50 (dd, J = 8.6, 6.4 Hz, 2H), 7.46 – 7.36 (m, 5H), 7.27 (d, J = 8.2 Hz, 2H), 3.94 (qd, J = 7.3, 2.2 Hz, 1H), 2.41 (s, 3H), 1.52 (d, J = 7.3 Hz, 3H).

^{13}C NMR (100 MHz, $CDCl_3$) δ 177.5, 169.8, 140.8, 135.8, 132.1, 130.7, 130.4, 129.9, 129.1, 128.5, 128.2, 126.5, 38.4, 21.6, 14.6.

HRMS (ESI-TOF) m/z: $[M + H]^+$ calculated for $C_{19}H_{18}NO_2$ 292.1332; Found 292.1340.

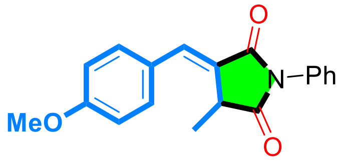

3-(4-Methoxybenzylidene)-4-methyl-1-phenylpyrrolidine-2,5-dione (6v):

Yellow solid, 40.5 mg, 66% yield, R_f=0.2 (PE/EtOAc 5/1)

^{1}H NMR (400 MHz, $CDCl_3$) δ 7.67 (d, J = 2.2 Hz, 1H), 7.53 – 7.46 (m, 4H), 7.43 – 7.36 (m, 3H), 6.98 (d, J = 8.9 Hz, 2H), 3.94 – 3.83 (m, 4H), 1.53 (d, J = 7.3 Hz, 3H).

^{13}C NMR (100 MHz, $CDCl_3$) δ 177.5, 169.9, 161.2, 135.4, 132.3, 132.2, 129.1, 128.5, 126.6, 126.5, 126.1, 114.6, 55.5, 38.4, 14.7.

HRMS (ESI-TOF) m/z: $[M + H]^+$ calculated for $C_{19}H_{18}NO_3$ 308.1281; Found 308.1290.

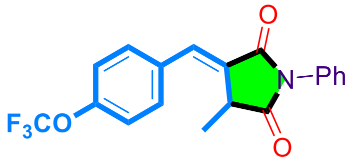

***E*-3-methyl-1-phenyl-4-(4-(trifluoromethoxy)benzylidene)pyrrolidine-2,5-dione (6w)**:

Light yellow solid, 25.1 mg (0.1 mmol), 70% yield, R_f=0.2 (PE/EtOAc 5/1)

^{1}H NMR (400 MHz, $CDCl_3$) δ 7.71 (d, J = 2.4 Hz, 1H), 7.60 – 7.54 (m, 2H), 7.50 (dd, J = 8.4, 6.8 Hz, 2H), 7.45 – 7.36 (m, 3H), 7.31 (d, J = 8.2 Hz, 2H), 3.92 (qd, J = 7.3, 2.3 Hz, 1H), 1.50 (d, J = 7.5 Hz, 3H).

^{13}C NMR (100 MHz, $CDCl_3$) δ 176.94, 169.32, 150.13 (q, J = 1.8 Hz), 133.97, 132.06, 131.93, 131.66, 130.26, 129.16, 128.63, 126.39, 121.21, 120.38 (q, J = 258.2 Hz), 38.22, 14.56.

^{19}F NMR (376 MHz, $CDCl_3$) δ -57.67.

HRMS (ESI-TOF) m/z: $[M + H]^+$ calculated for $C_{19}H_{15}F_3NO_3$ 362.0999; Found 362.1007.

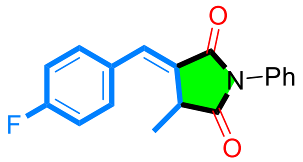

3-(4-Fluorobenzylidene)-4-methyl-1-phenylpyrrolidine-2,5-dione (6x):
White solid, 29.5 mg, 50% yield, R_f=0.2 (PE/EtOAc 10/1)
^{1}H NMR (400 MHz, $CDCl_3$) δ 7.70 (d, J = 2.3 Hz, 1H), 7.56 – 7.47 (m, 4H), 7.45 – 7.36 (m, 3H), 7.17 (t, J = 8.6 Hz, 2H), 3.91 (qd, J = 7.3, 2.3 Hz, 1H), 1.50 (d, J = 7.3 Hz, 3H).
^{13}C NMR (100 MHz, $CDCl_3$) δ 177.2, 169.5, 163.5 (d, J = 252.7 Hz), 134.5, 132.3 (d, J = 8.4 Hz), 132.0, 129.7 (d, J = 3.7 Hz), 129.2, 129.0 (d, J = 2.2 Hz), 128.6, 126.4, 116.4 (d, J = 22.0 Hz), 38.2, 14.5.
^{19}F NMR (376 MHz, $CDCl_3$) δ -108.70.
HRMS (ESI-TOF) m/z: $[M + H]^+$ calculated for $C_{18}H_{15}FNO_2$ 296.1081; Found 296.1090.

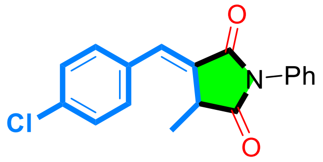

3-(4-Chlorobenzylidene)-4-methyl-1-phenylpyrrolidine-2,5-dione (6y):
White solid, 36.7 mg, 59% yield, R_f=0.2 (PE/EtOAc 10/1)
^{1}H NMR (400 MHz, $CDCl_3$) δ 7.68 (d, J = 2.2 Hz, 1H), 7.53 – 7.36 (m, 9H), 3.91 (qd, J = 7.3, 2.3 Hz, 1H), 1.50 (d, J = 7.5 Hz, 3H).
^{13}C NMR (100 MHz, $CDCl_3$) δ 177.0, 169.4, 136.2, 134.3, 132.0, 131.9, 131.4, 129.9, 129.4, 129.2, 128.6, 126.4, 38.3, 14.5.
HRMS (ESI-TOF) m/z: $[M + H]^+$ calculated for $C_{18}H_{15}ClNO_2$ 312.0786; Found 312.0796.

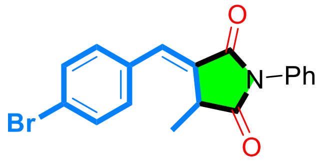

3-(4-Bromobenzylidene)-4-methyl-1-phenylpyrrolidine-2,5-dione (6z):
White solid, 32.0 mg, 45% yield, R_f=0.2 (PE/EtOAc 10/1).
^{1}H NMR (400 MHz, $CDCl_3$) δ 7.66 (d, J = 2.3 Hz, 1H), 7.64 – 7.57 (m, 2H), 7.50 (dd, J = 8.4, 6.7 Hz, 2H), 7.44 – 7.35 (m, 5H), 3.90 (qd, J = 7.3, 2.3 Hz, 1H), 1.49 (d, J = 7.3 Hz, 3H).
^{13}C NMR (100 MHz, $CDCl_3$) δ 177.0, 169.4, 134.4, 132.4, 131.9, 131.5, 130.1, 129.2, 128.6, 126.4, 124.6, 38.3, 14.5.
HRMS (ESI-TOF) m/z: $[M + H]^+$ calculated for $C_{18}H_{15}BrNO_2$ 356.0281; Found 356.0290.

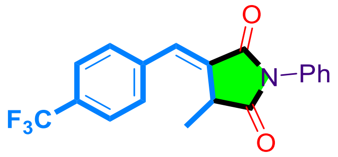

3-Methyl-1-phenyl-4-(4-(trifluoromethyl)benzylidene)pyrrolidine-2,5-dione (6aa):
Yellow solid, 42.8 mg, 62% yield, R_f=0.2 (PE/EtOAc 10/1). **6aa**:**6aa'** = 2.3:1
^{1}H NMR (400 MHz, $CDCl_3$) δ 7.71 (s, 1H), 7.64 (d, J = 8.3 Hz, 2H), 7.51 (dd, J = 8.3, 6.8 Hz, 2H), 7.44 – 7.38 (m, 4H), 7.35 – 7.32 (m, 1H), 3.96 (qd, J = 7.5, 2.4 Hz, 1H), 1.48 (d, J = 7.5 Hz, 3H).
^{13}C NMR (100 MHz, $CDCl_3$) δ 176.8, 169.1, 137.0, 133.9, 131.6 (q, J = 30.1 Hz), 130.2, 129.3, 129.2, 129.1, 126.4, 126.0 (q, J = 3.7 Hz), 125.7, 123.7 (q, J = 272.2 Hz), 38.3, 14.5.

^{19}**F NMR** (376 MHz, $CDCl_3$) δ -62.88.

HRMS (ESI-TOF) m/z: $[M + H]^+$ calculated for $C_{19}H_{15}F_3NO_2$ 346.1049; Found 346.1053.

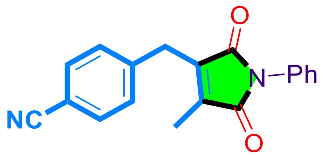

4-((4-Methyl-2,5-dioxo-1-phenyl-2,5-dihydro-1H-pyrrol-3-yl)methyl)benzonitrile (6ac'):

White solid, 45.1 mg, 75% yield, R_f=0.2 (PE/EtOAc 5/1).

^{1}**H NMR** (400 MHz, $CDCl_3$) δ 7.61 (d, J = 8.3 Hz, 2H), 7.48 – 7.38 (m, 4H), 7.34 (dd, J = 7.6, 5.5 Hz, 3H), 3.87 (s, 2H), 2.10 (s, 3H).

^{13}**C NMR** (100 MHz, $CDCl_3$) δ 170.4, 170.2, 142.2, 138.9, 137.8, 132.7, 131.6, 129.7, 129.1, 127.7, 125.7, 118.6, 111.1, 29.9, 9.3.

HRMS (ESI-TOF) m/z: $[M + H]^+$ calculated for $C_{20}H_{16}N_2O_2$ 316.1206; Found 316.1212.

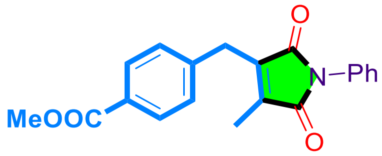

Methyl 4-((4-methyl-2,5-dioxo-1-phenyl-2,5-dihydro-1H-pyrrol-3-yl)methyl)benzoate (6ad'):

Light yellow solid, 21.4 mg, 32% yield, R_f=0.2 (PE/EtOAc 10/1).

^{1}**H NMR** (400 MHz, $CDCl_3$) δ 8.03 – 7.96 (m, 2H), 7.46 – 7.41 (m, 2H), 7.38 – 7.31 (m, 5H), 3.91 (s, 3H), 3.87 (s, 2H), 2.07 (s, 3H).

^{13}**C NMR** (100 MHz, $CDCl_3$) δ 170.6, 170.4, 166.8, 142.0, 138.5, 138.5, 131.7, 130.2, 129.0, 129.0, 128.9, 127.6, 125.7, 52.2, 29.8, 9.2.

HRMS (ESI-TOF) m/z: $[M + H]^+$ calculated for $C_{20}H_{18}NO_4$ 336.1230; Found 336.1233.

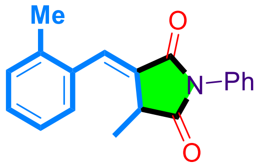

3-Methyl-4-(2-methylbenzylidene)-1-phenylpyrrolidine-2,5-dione (6ae):

Brown oil, 9.9 mg, 17% yield, R_f=0.2 (PE/EtOAc 10/1).

^{1}**H NMR** (700 MHz, $CDCl_3$) δ 7.95 (d, J = 2.5 Hz, 1H), 7.53 – 7.47 (m, 2H), 7.43 – 7.38 (m, 4H), 7.33 – 7.30 (m, 1H), 7.27 (d, J = 7.2 Hz, 2H), 3.89 (qd, J = 7.4, 2.4 Hz, 1H), 2.42 (s, 3H), 1.31 (d, J = 7.3 Hz, 3H).

^{13}**C NMR** (176 MHz, $CDCl_3$) δ 177.3, 169.3, 138.3, 134.7, 132.9, 132.1, 130.8, 130.5, 129.8, 129.1, 128.5, 127.7, 126.4, 126.3, 38.2, 20.0, 14.2.

HRMS (ESI-TOF) m/z: $[M + H]^+$ calculated for $C_{19}H_{18}NO_2$ 292.1332; Found 292.1339.

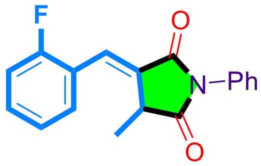

***E*-3-(2-fluorobenzylidene)-4-methyl-1-phenylpyrrolidine-2,5-dione (6af)**:

Yellow solid, 20.1 mg (0.1 mmol), 68% yield, R_f=0.2 (PE/EtOAc 10/1).

^{1}H NMR (700 MHz, $CDCl_3$) δ 7.88 (d, J = 2.7 Hz, 1H), 7.53 – 7.47 (m, 3H), 7.45 – 7.37 (m, 4H), 7.24 (t, J = 7.5 Hz, 1H), 7.17 (dd, J = 10.2, 8.5 Hz, 1H), 3.90 (qd, J = 7.4, 2.5 Hz, 1H), 1.40 (d, J = 7.4 Hz, 3H).

^{13}C NMR (176 MHz, $CDCl_3$) δ 177.1, 169.1, 160.9 (d, J = 252.9 Hz), 132.0 (d, J = 5.2 Hz), 131.9, 131.8, 130.1 (d, J = 3.3 Hz), 129.2, 128.6, 128.5 (d, J = 3.9 Hz), 126.4, 124.5 (d, J = 3.3 Hz), 121.7 (d, J = 13.0 Hz), 116.3 (d, J = 22.1 Hz), 38.7, 14.3.

HRMS (ESI-TOF) m/z: $[M + H]^+$ calculated for $C_{18}H_{15}FNO_2$ 296.1081; Found 296.1090.

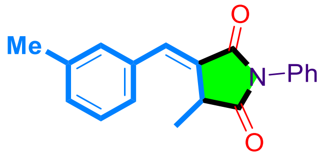

3-Methyl-4-(3-methylbenzylidene)-1-phenylpyrrolidine-2,5-dione (6aj):

Yellow solid, 34.9 mg, 60% yield, R_f=0.2 (PE/EtOAc 10/1).

^{1}H NMR (400 MHz, $CDCl_3$) δ 7.71 (d, J = 2.2 Hz, 1H), 7.53 – 7.46 (m, 2H), 7.43 – 7.32 (m, 6H), 7.25 – 7.21 (m, 1H), 3.96 (qd, J = 7.3, 2.3 Hz, 1H), 2.41 (s, 3H), 1.51 (d, J = 7.3 Hz, 3H).

^{13}C NMR (100 MHz, $CDCl_3$) δ 177.4, 169.7, 138.8, 136.0, 133.5, 132.1, 131.2, 131.0, 129.1, 129.1, 129.0, 128.5, 127.2, 126.5, 38.4, 21.5, 14.6.

HRMS (ESI-TOF) m/z: $[M + H]^+$ calculated for $C_{19}H_{18}NO_2$ 292.1332; Found 292.1340.

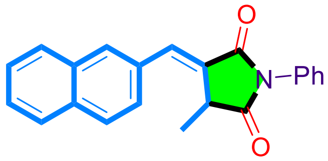

***E*-3-methyl-4-(naphthalen-2-ylmethylene)-1-phenylpyrrolidine-2,5-dione (6ah)**:

Yellow solid, 19.5 mg (0.1 mmol), 60% yield, R_f=0.2 (PE/EtOAc 10/1).

^{1}H NMR (400 MHz, $CDCl_3$) δ 8.03 (d, J = 1.8 Hz, 1H), 7.90 (dt, J = 11.4, 9.1 Hz, 4H), 7.64 (dd, J = 8.6, 2.0 Hz, 1H), 7.62 – 7.54 (m, 2H), 7.54 – 7.49 (m, 2H), 7.46 – 7.39 (m, 3H), 4.08 (qd, J = 8.1, 7.3, 3.0 Hz, 1H), 1.56 (d, J = 7.3 Hz, 3H).

^{13}C NMR (100 MHz, $CDCl_3$) δ 177.3, 169.7, 135.9, 133.8, 133.2, 132.1, 131.6, 131.0, 129.4, 129.1, 128.9, 128.6, 128.6, 127.8, 127.0, 126.5, 125.9, 38.5, 15.0.

HRMS (ESI-TOF) m/z: $[M + H]^+$ calculated for $C_{22}H_{18}NO_2$ 328.1332; Found 328.1335.

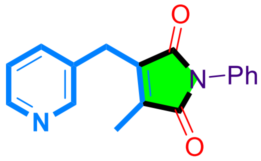

3-Methyl-1-phenyl-4-(pyridin-3-ylmethyl)-1H-pyrrole-2,5-dione (6al'):

Yellow oil, 10.9 mg (0.1 mmol), 39% yield, R_f=0.2 (PE/EtOAc 5/1).

^{1}H NMR (400 MHz, $CDCl_3$) δ 8.57 (s, 1H), 8.54 – 8.44 (m, 1H), 7.64 (dt, J = 7.8, 2.0 Hz, 1H), 7.48 – 7.40 (m, 2H), 7.33 (ddd, J = 7.1, 4.7, 2.6 Hz, 3H), 7.25 (d, J = 7.5 Hz, 1H), 3.82 (s, 2H), 2.11 (s, 3H).

^{13}C NMR (100 MHz, $CDCl_3$) 170.5, 170.3, 150.0, 148.4, 138.6, 138.2, 136.5, 132.5, 131.7, 129.0, 127.6, 125.7, 123.8, 27.1, 9.2.

HRMS (ESI-TOF) m/z: $[M + H]^+$ calculated for $C_{17}H_{15}N_2O_2$ 279.1128; Found 279.1124.

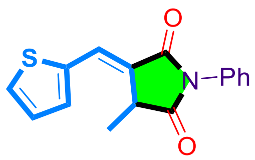

***E*-3-methyl-1-phenyl-4-(thiophen-2-ylmethylene)pyrrolidine-2,5-dione (6aj)**:

Yellow solid, 31.1 mg, 55% yield, R_f=0.2 (PE/EtOAc 10/1).

^{1}H NMR (400 MHz, $CDCl_3$) δ 7.88 (d, J = 2.1 Hz, 1H), 7.61 (dt, J = 5.1, 1.1 Hz, 1H), 7.50 (dd, J = 8.3, 6.7 Hz, 2H), 7.43 – 7.35 (m, 4H), 7.17 (dd, J = 5.1, 3.7 Hz, 1H), 3.80 (qd, J = 7.3, 2.0 Hz, 1H), 1.67 (d, J = 7.3 Hz, 3H).

^{13}C NMR (100 MHz, $CDCl_3$) δ 177.2, 169.5, 137.0, 133.8, 132.0, 131.2, 129.1, 128.5, 128.3, 128.0, 126.7, 126.4, 38.6, 15.5.

HRMS (ESI-TOF) m/z: $[M + H]^+$ calculated for $C_{16}H_{14}NO_2S$ 284.0740; Found 284.0731.

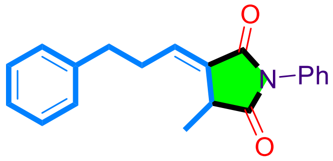

3-Methyl-1-phenyl-4-(3-phenylpropylidene)pyrrolidine-2,5-dione (6ak):

Yellow solid, 38.4mg, 63% yield, R_f=0.2 (PE/EtOAc 10/1). *Z:E* = 1:1

^{1}H NMR (700 MHz, $CDCl_3$) δ 7.46 (t, J = 7.9 Hz, 4H), 7.39 – 7.36 (m, 2H), 7.33 – 7.27 (m, 8H), 7.24 – 7.18 (m, 6H), 7.03 (qd, J = 7.3, 2.3 Hz, 1H), 6.95 (td, J = 7.8, 2.3 Hz, 1H), 3.58 – 3.52 (m, 1H), 3.35 – 3.29 (m, 1H), 2.84 (tt, J = 10.0, 5.2 Hz, 2H), 2.80 – 2.74 (m, 1H), 2.72 – 2.67 (m, 1H), 2.66 – 2.57 (m, 2H), 2.45 – 2.37 (m, 1H), 2.25 – 2.17 (m, 1H), 1.92 (dd, J = 7.3, 1.4 Hz, 3H), 1.42 (d, J = 7.4 Hz, 3H).

^{13}C NMR (176 MHz, $CDCl_3$) δ 177.1, 176.3, 168.7, 168.6, 140.6, 140.4, 139.2, 135.5, 132.0, 132.0, 131.4, 130.0, 129.1, 129.1, 128.7, 128.6, 128.6, 128.5, 128.4, 126.5, 126.5, 126.4, 126.4, 41.7, 37.5, 34.8, 32.1, 31.4, 31.3, 16.3, 15.0.

HRMS (ESI-TOF) m/z: $[M + H]^+$ calculated for $C_{20}H_{20}NO_2$ 306.1489; Found 306.1491.

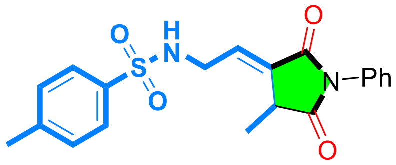

4-Methyl-N-(2-(4-methyl-2,5-dioxo-1-phenylpyrrolidin-3-ylidene)ethyl)benzenesulfonamide (6al):

White solid, 58.4 mg, 76% yield, R_f=0.2 (PE/EtOAc 2/1).

^{1}H NMR (400 MHz, $CDCl_3$) δ 8.47 (s, 1H), 7.93 (d, J = 8.3 Hz, 2H), 7.42 – 7.35 (m, 2H), 7.27 (dd, J = 13.1, 7.7 Hz, 4H), 7.11 (d, J = 1.6 Hz, 1H), 7.06 (t, J = 7.4 Hz, 1H), 4.43 (qt, J = 19.6, 1.8 Hz, 2H), 3.58 – 3.42 (m, 1H), 2.39 (s, 3H), 1.41 (d, J = 7.0 Hz, 3H).

^{13}C NMR (100 MHz, $CDCl_3$) δ 169.6, 169.0, 145.6, 140.9, 138.2, 137.9, 134.9, 129.9, 128.8, 128.0, 124.2, 119.7, 51.1, 38.4, 21.7, 14.7.

HRMS (ESI-TOF) m/z: $[M + H]^+$ calculated for $C_{20}H_{21}N_2O_4S$ 385.1217; Found 385.1225.

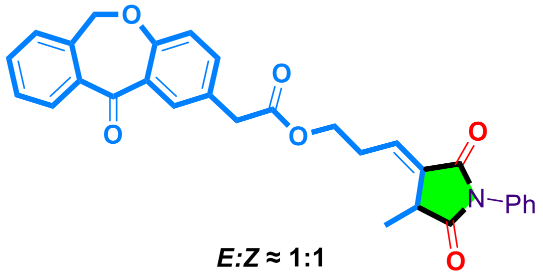

E:Z ≈ 1:1

3-(4-Methyl-2,5-dioxo-1-phenylpyrrolidin-3-ylidene)propyl 2-(11-oxo-6,11-dihydrodibenzo[b,e]oxepin-2-yl)acetate (6am):

Yellow oil, 35.8 mg (0.1 mmol), 72% yield, R_f=0.2 (PE/EtOAc 3/1).

^{1}H NMR (700 MHz, $CDCl_3$) δ 8.11 (d, J = 2.5 Hz, 1H), 8.06 (d, J = 2.5 Hz, 1H), 7.89 – 7.86 (m, 2H), 7.57 – 7.53 (m, 2H), 7.49 – 7.46 (m, 3H), 7.46 – 7.43 (m, 3H), 7.41 – 7.37 (m, 3H), 7.36 – 7.33 (m, 5H), 7.33 – 7.31 (m, 2H), 7.06 – 7.01 (m, 2H), 7.00 (d, J = 8.5 Hz, 1H), 6.84 (td, J = 7.6, 2.3 Hz, 1H), 5.17 (s, 2H), 5.15 (s, 2H), 4.36 (dt, J = 11.4, 5.6 Hz, 1H), 4.33 – 4.26 (m, 2H), 4.24 – 4.20 (m, 1H), 3.65 (s, 2H), 3.64 – 3.61 (m, 1H), 3.54 (d, J = 2.2 Hz, 2H), 3.45 – 3.40 (m, 1H), 2.72 – 2.60 (m, 2H), 2.47 – 2.40 (m, 1H), 2.28 – 2.22 (m, 1H), 1.92 (dd, J = 7.3, 1.4 Hz, 3H), 1.52 (d, J = 7.4 Hz, 3H).

^{13}C NMR (176 MHz, $CDCl_3$) δ 190.8, 190.8, 176.8, 175.9, 171.2, 171.2, 168.5, 168.2, 160.6, 160.5, 140.4, 140.4, 136.4, 136.2, 135.9, 135.6, 135.5, 135.0, 133.1, 132.8, 132.5, 131.9, 131.9, 129.5, 129.5, 129.4, 129.3, 129.3, 129.1, 129.0, 128.5, 128.5, 127.8, 127.4, 127.4, 126.4, 126.3, 125.2, 125.1, 121.1, 121.1, 121.1, 73.6, 73.6, 62.9, 61.4, 40.2, 39.9, 39.6, 37.5, 29.0, 28.7, 16.3, 15.0.

HRMS (ESI-TOF) m/z: $[M + H]^+$ calculated for $C_{30}H_{26}NO_6$ 496.1755; Found 496.1753.

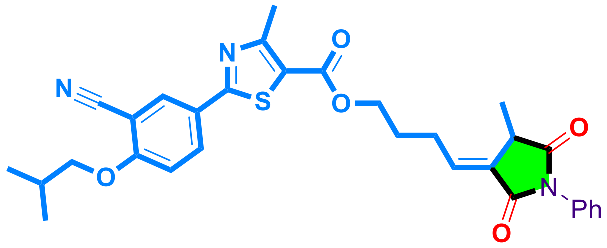

E:Z ≈ 1:1

4-(4-Methyl-2,5-dioxo-1-phenylpyrrolidin-3-ylidene)butyl 2-(3-cyano-4-isobutoxyphenyl)-4-methylthiazole-5-carboxylate (6an):

Yellow oil, 37.4 mg (0.1 mmol), 67% yield, R_f=0.2 (PE/EtOAc 3/1).

^{1}H NMR (700 MHz, $CDCl_3$) δ 8.18 (d, J = 2.4 Hz, 1H), 8.17 (d, J = 2.4 Hz, 1H), 8.08 (dd, J = 8.8, 2.4 Hz, 1H), 8.05 (dd, J = 8.8, 2.4 Hz, 1H), 7.48 (t, J = 7.9 Hz, 2H), 7.44 (t, J = 7.9 Hz, 2H), 7.41 – 7.38 (m, 1H), 7.38 – 7.36 (m, 1H), 7.35 – 7.32 (m, 2H), 7.31 – 7.29 (m, 2H), 7.10 – 7.06 (m, 1H), 7.01 (d, J = 8.8 Hz, 1H), 6.98 – 6.96 (m, 1H), 6.95 (d, J = 8.8 Hz, 1H), 4.42 – 4.36 (m, 2H), 4.35 – 4.32 (m, 2H), 3.90 (d, J = 6.6 Hz, 2H), 3.88 (d, J = 6.5 Hz, 2H), 3.65 – 3.61 (m, 1H), 3.53 – 3.48 (m, 1H), 2.77 (s, 3H), 2.76 (s, 3H), 2.53 (p, J = 7.7, 7.2 Hz, 1H), 2.51 – 2.45 (m, 1H), 2.28 – 2.22 (m, 1H), 2.22 – 2.17 (m, 2H), 2.06 – 2.02 (m, 3H), 2.00 (dd, J = 7.4, 1.3 Hz, 3H), 1.98 – 1.90 (m, 1H), 1.84 – 1.78 (m, 1H), 1.55 (d, J = 7.4 Hz, 3H), 1.10 (d, J = 1.4 Hz, 6H), 1.09 (d, J = 1.4 Hz, 6H).

^{13}C NMR (176 MHz, $CDCl_3$) δ 177.0, 176.1, 168.6, 168.5, 167.5, 167.4, 162.6, 162.6, 161.9, 161.9, 161.7, 161.5, 139.0, 135.9, 132.7, 132.6, 132.1, 132.1, 131.9, 131.9, 131.5, 129.7, 129.1, 129.1, 128.6, 128.5, 126.4, 126.4, 125.9, 125.8, 121.4, 121.1, 115.4, 115.4, 112.7, 112.6, 103.0, 103.0, 75.7, 75.7, 64.5, 64.5, 41.7, 37.5, 28.2, 28.1, 27.7, 27.0, 26.3, 24.5, 19.1, 17.5, 17.5, 16.4, 15.1.

HRMS (ESI-TOF) m/z: $[M + H]^+$ calculated for $C_{31}H_{32}N_3O_5S$ 558.2057; Found 558.2067.

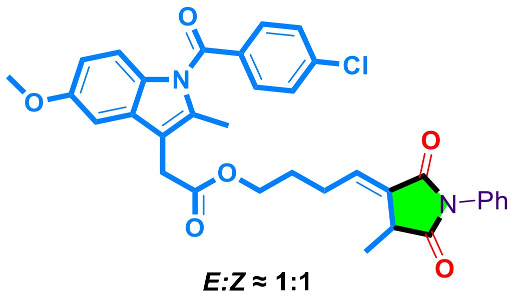

***E:Z* ≈ 1:1**

4-(4-Methyl-2,5-dioxo-1-phenylpyrrolidin-3-ylidene)butyl 2-(1-(4-chlorobenzoyl)-5-methoxy-2-methyl-1H-indol-3-yl)acetate (6ao):

Brown oil, 38.9 mg (0.1 mmol), 65% yield, R_f=0.2 (PE/EtOAc 3/1).

^{1}H NMR (700 MHz, $CDCl_3$) δ 7.65 (dd, J = 11.1, 8.5 Hz, 4H), 7.48 – 7.44 (m, 8H), 7.38 (t, J = 7.2 Hz, 2H), 7.32 (d, J = 7.9 Hz, 2H), 7.29 (d, J = 6.0 Hz, 2H), 7.00 (tt, J = 7.2, 3.6 Hz, 1H), 6.96 (dd, J = 16.2, 2.2 Hz, 2H), 6.86 (d, J = 9.0 Hz, 2H), 6.84 – 6.81 (m, 1H), 6.67 (td, J = 8.7, 2.3 Hz, 2H), 4.20 – 4.16 (m, 2H), 4.15 – 4.10 (m, 2H), 3.83 (s, 3H), 3.81 (s, 3H), 3.69 (s, 2H), 3.66 (s, 2H), 3.51 (s, 1H), 3.30 (q, J = 7.0 Hz, 1H), 2.41 (s, 3H), 2.38 (s, 3H), 2.34 – 2.30 (m, 1H), 2.26 (dt, J = 15.1, 7.7 Hz, 1H), 2.11 – 2.05 (m, 1H), 1.90 – 1.87 (m, 1H), 1.85 (d, J = 7.3 Hz, 3H), 1.81 – 1.75 (m, 1H), 1.72 – 1.65 (m, 3H), 1.39 (d, J = 7.5 Hz, 3H).

^{13}C NMR (176 MHz, $CDCl_3$) δ 176.9, 176.1, 170.8, 168.5, 168.5, 168.3, 156.1, 156.1, 139.3, 139.3, 138.5, 136.1, 136.0, 135.8, 133.9, 133.8, 131.9, 131.9, 131.6, 131.2, 131.2, 130.8, 130.8, 130.6, 130.6, 129.7, 129.2, 129.1, 129.1, 129.1, 128.5, 128.5, 126.4, 115.0, 115.0, 112.4, 112.4, 111.6, 111.4, 101.5, 101.3, 64.5, 64.0, 55.7, 55.7, 41.7, 37.4, 30.4, 30.3, 27.7, 27.0, 25.8, 24.4, 16.3, 14.9, 13.4.

HRMS (ESI-TOF) m/z: $[M + H]^+$ calculated for $C_{34}H_{32}ClN_2O_6$ 599.1943; Found 599.1941.

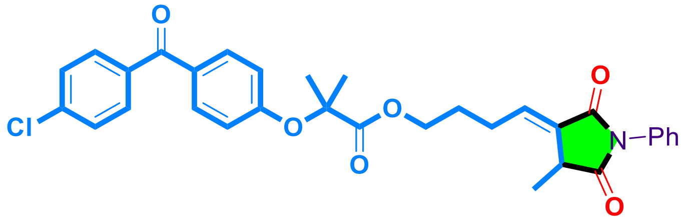

***E:Z* ≈ 1:1**

4-(4-Methyl-2,5-dioxo-1-phenylpyrrolidin-3-ylidene)butyl 2-(4-(4-chlorobenzoyl)phenoxy)-2-methylpropanoate (6ap):

Yellow oil, 44.0 mg (0.1 mmol), 49% yield, R_f=0.2 (PE/EtOAc 3/1).

^{1}H NMR (700 MHz, $CDCl_3$) δ 7.75 (d, J = 8.6 Hz, 2H), 7.72 (d, J = 5.9 Hz, 2H), 7.69 (d, J = 5.4 Hz, 2H), 7.68 (s, 2H), 7.48 – 7.45 (m, 3H), 7.43 (d, J = 7.4 Hz, 5H), 7.40 – 7.37 (m, 2H), 7.32 (d, J = 7.9 Hz, 2H), 7.28 (s, 2H), 7.03 – 6.98 (m, 1H), 6.87 (d, J = 8.6 Hz, 2H), 6.85 (d, J = 8.6 Hz, 2H), 6.78 (t, J = 7.3 Hz, 1H), 4.24 – 4.22 (m, 2H), 4.19 (t, J = 6.2 Hz, 2H), 3.50 (s, 1H), 3.34 (q, J = 7.3 Hz, 1H), 2.26 – 2.21 (m, 1H), 2.21 – 2.15 (m, 1H), 1.90 (d, J = 7.3 Hz, 3H), 1.86 – 1.82 (m, 3H), 1.80 – 1.75 (m, 3H), 1.70 (s, 6H), 1.67 (s, 6H), 1.43 (d, J = 7.5 Hz, 3H).

^{13}C NMR (176 MHz, $CDCl_3$) δ 194.2, 194.1, 176.9, 176.0, 173.7, 173.7, 168.5, 168.4, 159.6, 159.6, 138.5, 138.4, 138.2, 136.3, 136.2, 135.9, 132.1, 132.1, 132.1, 132.0, 131.9, 131.8, 131.7, 131.2, 131.2, 130.5, 130.5, 129.6, 129.1, 129.1, 128.6, 128.6, 128.5, 126.4, 126.4, 117.2, 117.0, 79.4, 79.4, 65.0, 64.6, 41.6, 37.4, 27.5, 26.8, 25.6, 25.5, 25.5, 25.4, 24.2, 16.3, 15.0.

HRMS (ESI-TOF) m/z: $[M + H]^+$ calculated for $C_{32}H_{31}ClNO_6$ 560.1834; Found 560.1850.

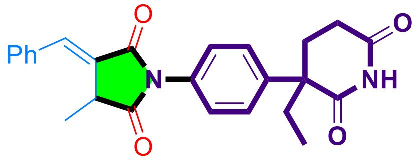

3-(4-(3-(E)-benzylidene)-4-methyl-2,5-dioxopyrrolidin-1-yl)phenyl)-3-ethylpiperidine-2,6-dione (6aq):

White solid, 33.1 mg (0.1 mmol), 80% yield, R_f=0.2 (PE/EtOAc 2/1).

^{1}H NMR (400 MHz, $CDCl_3$) δ 8.15 (s, 1H), 7.75 (d, J = 2.3 Hz, 1H), 7.54 (d, J = 7.2 Hz, 2H), 7.50 – 7.39 (m, 7H), 3.96 (qd, J = 7.3, 2.3 Hz, 1H), 2.63 (dt, J = 18.3, 3.8 Hz, 1H), 2.51 – 2.37 (m, 2H), 2.27 (td, J = 14.2, 4.5 Hz, 1H), 2.10 (dt, J = 14.7, 7.3 Hz, 1H), 1.93 (dq, J = 14.5, 7.3 Hz, 1H), 1.51 (d, J = 7.3 Hz, 3H), 0.90 (t, J = 7.5 Hz, 3H).

^{13}C NMR (100 MHz, $CDCl_3$) δ 177.2, 174.7, 172.0, 169.4, 139.1, 136.1, 133.4, 131.5, 130.2, 129.1, 129.1, 127.0, 126.7, 51.0, 38.4, 33.0, 29.3, 26.9, 14.6, 9.1.

HRMS (ESI-TOF) m/z: $[M + H]^+$ calculated for $C_{25}H_{25}N_2O_4$ 417.1089; Found 417.1818.

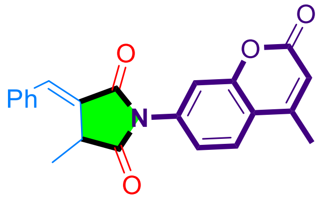

***E*-3-benzylidene-4-methyl-1-(4-methyl-2-oxo-2H-chromen-7-yl)pyrrolidine-2,5-dione (6ar)**:

Yellow solid, 28.5 mg, 79% yield, R_f=0.2 (PE/EtOAc 3/1).

^{1}H NMR (700 MHz, $CDCl_3$) δ 7.77 (d, J = 2.5 Hz, 1H), 7.71 (d, J = 8.5 Hz, 1H), 7.58 – 7.52 (m, 2H), 7.49 (dd, J = 4.4, 2.4 Hz, 2H), 7.46 – 7.40 (m, 3H), 6.36 – 6.33 (m, 1H), 3.99 (qd, J = 7.4, 2.4 Hz, 1H), 2.46 (d, J = 1.4 Hz, 3H), 1.53 (d, J = 7.4 Hz, 3H).

^{13}C NMR (176 MHz, $CDCl_3$) δ 176.7, 169.0, 160.3, 153.6, 151.7, 136.7, 134.9, 133.3, 131.1, 130.4, 130.3, 129.2, 125.1, 122.0, 119.7, 115.7, 115.0, 38.5, 18.7, 14.5.

HRMS (ESI-TOF) m/z: $[M + H]^+$ calculated for $C_{22}H_{18}NO_4$ 366.1230; Found 366.1240.

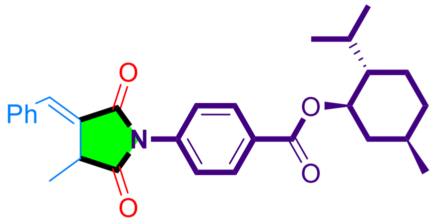

(1R,5R)-2-isopropyl-5-methylcyclohexyl 4-(3-((E)-benzylidene)-4-methyl-2,5-dioxopyrrolidin-1-yl)benzoate (6as):

White solid, 28.8 mg (0.1 mmol), 63% yield, R_f=0.2 (PE/EtOAc 10/1).

^{1}H NMR (400 MHz, $CDCl_3$) δ 8.28 – 8.08 (m, 2H), 7.76 (d, J = 2.2 Hz, 1H), 7.61 – 7.50 (m, 4H), 7.50 – 7.40 (m, 3H), 4.95 (td, J = 10.9, 4.4 Hz, 1H), 3.97 (qt, J = 7.3, 1.9 Hz, 1H), 2.19 – 2.09 (m, 1H), 1.95 (qt, J = 7.0, 3.5 Hz, 1H), 1.77 – 1.70 (m, 2H), 1.60 – 1.49 (m, 5H), 1.21 – 1.05 (m, 3H), 0.93 (t, J = 6.5 Hz, 6H), 0.80 (d, J = 7.0 Hz, 3H).

^{13}C NMR (100 MHz, $CDCl_3$) δ 176.9, 169.2, 165.2, 136.3, 135.9, 133.4, 130.6, 130.4, 130.3, 130.3, 129.1, 129.0, 126.1, 75.2, 47.3, 40.9, 38.5, 34.3, 31.5, 26.5, 23.6, 22.1, 20.8, 16.5, 14.6.

HRMS (ESI-TOF) m/z: $[M + H]^+$ calculated for $C_{29}H_{34}NO_4$ 460.2482; Found 460.2485.

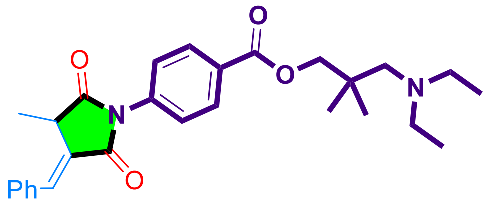

3-(Diethylamino)-2,2-dimethylpropyl (E)-4-(3-benzylidene-4-methyl-2,5-dioxopyrrolidin-1-yl)benzoate (6at):

Yellow solid, 19.1 mg (0.1 mmol), 41% yield, R_f=0.2 (PE/EtOAc 5/1).

^{1}H NMR (400 MHz, $CDCl_3$) δ 8.22 – 8.10 (m, 2H), 7.76 (d, J = 2.3 Hz, 1H), 7.61 – 7.51 (m, 4H), 7.51 – 7.41 (m, 3H), 4.13 (s, 2H), 3.98 (qd, J = 7.3, 2.3 Hz, 1H), 2.54 (q, J = 7.2 Hz, 4H), 2.35 (s, 2H), 1.52 (d, J = 7.3 Hz, 3H), 0.97 (d, J = 5.7 Hz, 12H).

^{13}C NMR (100 MHz, $CDCl_3$) δ 176.9, 169.2, 165.7, 136.4, 136.0, 133.4, 130.3, 130.3, 130.3, 129.1, 129.0, 126.2, 71.5, 61.9, 49.1, 38.5, 36.5, 23.3, 14.6, 12.1.

HRMS (ESI-TOF) m/z: $[M + H]^+$ calculated for $C_{28}H_{35}N_2O_4$ 463.2591; Found 463.2600.

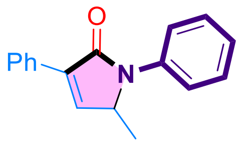

5-Methyl -1,3-diphenyl-1,5-dihydro-2H-pyrrol-2-one (7a):

White solid, 16.1 mg, 65% yield, R_f=0.2 (PE/EtOAc 10/1)

^{1}H NMR (400 MHz, $CDCl_3$) δ 8.02 – 7.81 (m, 2H), 7.55 (d, J = 8.6 Hz, 2H), 7.45 – 7.35 (m, 5H), 7.27 – 7.24 (m, 1H), 7.21 (t, J = 7.4 Hz, 1H), 4.80 (qd, J = 6.8, 2.2 Hz, 1H), 1.33 (d, J = 6.8 Hz, 3H).

^{13}C NMR (100 MHz, $CDCl_3$) δ 168.2, 141.8, 137.0, 136.0, 131.5, 129.1, 128.7, 128.5, 127.3, 125.1, 122.7, 56.2, 17.3.

HRMS (ESI-TOF) m/z: $[M + H]^+$ calculated for $C_{17}H_{16}NO$ 250.1226; Found 250.1231.

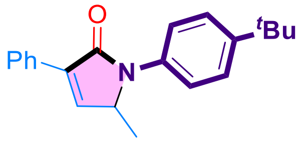

1-(4-(tert-Butyl)phenyl)-5-methyl-3-phenyl-1,5-dihydro-2H-pyrrol-2-one (7b):

Light yellow solid, 15.2 mg, 50% yield, R_f=0.2 (PE/EtOAc 10/1).

^{1}H NMR (400 MHz, $CDCl_3$) δ 7.98 – 7.85 (m, 2H), 7.47 – 7.35 (m, 8H), 4.78 (qd, J = 6.8, 2.2 Hz, 1H), 1.36 – 1.32 (m, 12H).

^{13}C NMR (100 MHz, $CDCl_3$) δ 168.2, 148.0, 141.6, 136.1, 134.3, 131.6, 128.6, 128.5, 127.3, 125.9, 122.4, 56.2, 34.5, 31.4, 17.4.

HRMS (ESI-TOF) m/z: $[M + H]^+$ calculated for $C_{21}H_{24}NO$ 306.1852; Found 306.1861.

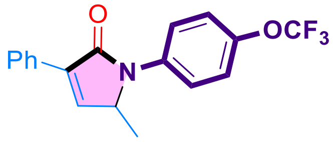

5-Methyl-3-phenyl-1-(4-(trifluoromethoxy)phenyl)-1,5-dihydro-2H-pyrrol-2-one (7c):

White solid, 20.8 mg, 62% yield, R_f=0.2 (PE/EtOAc 5/1).

^{1}H NMR (400 MHz, $CDCl_3$) δ 7.97 – 7.85 (m, 2H), 7.64 – 7.55 (m, 2H), 7.46 – 7.34 (m, 3H), 7.32 – 7.26 (m, 3H), 4.79 (qd, J = 6.8, 2.2 Hz, 1H), 1.36 (d, J = 6.8 Hz, 3H).

^{13}C NMR (100 MHz, $CDCl_3$) δ 168.3, 145.9, 141.8, 136.0, 135.7, 131.2, 128.9, 128.6, 127.3, 123.5, 121.8, 118.0 (q, J = 257.1 Hz), 56.1, 17.3.
^{19}F NMR (376 MHz, $CDCl_3$) δ -57.99.
HRMS (ESI-TOF) m/z: $[M + H]^+$ calculated for $C_{18}H_{15}F_3NO_2$ 334.1049; Found 334.1057.

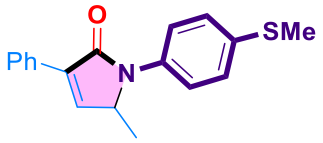

5-Methyl-1-(4-(methylthio)phenyl)-3-phenyl-1,5-dihydro-2H-pyrrol-2-one (7d):
Yellow solid, 14.8 mg, 50% yield, R_f=0.2 (PE/EtOAc 5/1).
^{1}H NMR (400 MHz, $CDCl_3$) δ 7.95 – 7.87 (m, 2H), 7.51 – 7.45 (m, 2H), 7.45 – 7.36 (m, 3H), 7.35 – 7.31 (m, 2H), 7.27 (d, J = 2.1 Hz, 1H), 4.76 (qd, J = 6.8, 2.1 Hz, 1H), 2.50 (s, 3H), 1.33 (d, J = 6.8 Hz, 3H).
^{13}C NMR (100 MHz, $CDCl_3$) δ 168.2, 141.6, 136.1, 134.7, 134.5, 131.4, 128.7, 128.5, 127.7, 127.3, 123.1, 56.2, 17.3, 16.4.
HRMS (ESI-TOF) m/z: $[M + H]^+$ calculated for $C_{18}H_{18}NOS$ 296.1104; Found 296.1111.

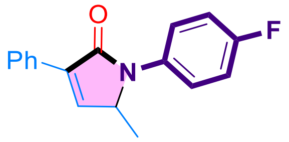

1-(4-Fluorophenyl)-5-methyl-3-phenyl-1,5-dihydro-2H-pyrrol-2-one (7e):
White solid, 17.0 mg, 64% yield, R_f=0.2 (PE/EtOAc 10/1).
^{1}H NMR (400 MHz, $CDCl_3$) δ 7.98 – 7.85 (m, 2H), 7.50 – 7.46 (m, 2H), 7.44 – 7.36 (m, 3H), 7.27 (d, J = 2.2 Hz, 1H), 7.16 – 7.09 (m, 2H), 4.74 (qd, J = 6.8, 2.1 Hz, 1H), 1.32 (d, J = 6.8 Hz, 3H).
^{13}C NMR (100 MHz, $CDCl_3$) δ 168.3, 160.1 (d, J = 245.0 Hz), 141.7, 136.0, 133.0 (d, J = 2.9 Hz), 131.4, 128.8, 128.5, 127.3, 124.6 (d, J = 8.1 Hz), 115.9 (d, J = 22.7 Hz), 56.5, 17.3.
^{19}F NMR (376 MHz, $CDCl_3$) δ -116.94.
HRMS (ESI-TOF) m/z: $[M + H]^+$ calculated for $C_{17}H_{15}FNO$ 268.1132; Found 268.1137.

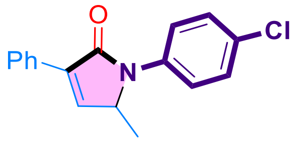

1-(4-Chlorophenyl)-5-methyl-3-phenyl-1,5-dihydro-2H-pyrrol-2-one (7f):
Yellow solid, 19.3 mg, 68% yield, R_f=0.2 (PE/EtOAc 10/1).
^{1}H NMR (400 MHz, $CDCl_3$) δ 7.95 – 7.85 (m, 2H), 7.54 – 7.48 (m, 2H), 7.47 – 7.33 (m, 5H), 7.28 (d, J = 2.2 Hz, 1H), 4.77 (qd, J = 6.8, 2.2 Hz, 1H), 1.34 (d, J = 6.8 Hz, 3H).
^{13}C NMR (100 MHz, $CDCl_3$) δ 168.2, 141.8, 136.0, 135.6, 131.2, 130.3, 129.1, 128.8, 128.5, 127.3, 123.6, 56.0, 17.3.
HRMS (ESI-TOF) m/z: $[M + H]^+$ calculated for $C_{17}H_{15}ClNO$ 284.0837; Found 2284.0840.

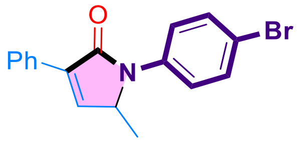

1-(4-Bromophenyl)-5-methyl-3-phenyl-1,5-dihydro-2H-pyrrol-2-one (7g):

White solid, 17.2 mg, 53% yield, R_f=0.2 (PE/EtOAc 10/1).

^{1}H NMR (400 MHz, $CDCl_3$) δ 7.95 – 7.83 (m, 2H), 7.57 – 7.50 (m, 2H), 7.48 – 7.34 (m, 5H), 7.27 (d, J = 2.2 Hz, 1H), 4.76 (qd, J = 6.8, 2.2 Hz, 1H), 1.34 (d, J = 6.8 Hz, 3H).

^{13}C NMR (100 MHz, $CDCl_3$) δ 168.2, 141.8, 136.2, 136.0, 132.1, 131.2, 128.8, 128.5, 127.3, 123.8, 118.0, 56.0, 17.3.

HRMS (ESI-TOF) m/z: $[M + H]^+$ calculated for $C_{17}H_{15}BrNO$ 328.0332; Found 328.0346.

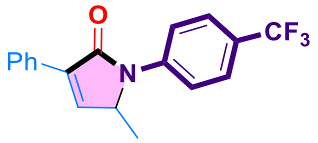

5-Methyl-3-phenyl-1-(4-(trifluoromethyl)phenyl)-1,5-dihydro-2H-pyrrol-2-one (7h):

White solid, 15.5 mg, 49% yield, R_f=0.2 (PE/EtOAc 10/1).

^{1}H NMR (400 MHz, $CDCl_3$) δ 7.96 – 7.84 (m, 2H), 7.74 (d, J = 8.9 Hz, 2H), 7.68 (d, J = 8.9 Hz, 2H), 7.47 – 7.36 (m, 3H), 7.31 (d, J = 2.2 Hz, 1H), 4.86 (qd, J = 6.8, 2.4 Hz, 1H), 1.39 (d, J = 6.8 Hz, 3H).

^{13}C NMR (100 MHz, $CDCl_3$) δ 168.4, 142.1, 140.2, 136.0, 131.0, 129.0, 128.6, 127.3, 126.6, 126.2 (q, J = 3.7 Hz), 124.1 (q, J = 271.8 Hz), 121.5, 55.7, 17.3.

^{19}F NMR (376 MHz, $CDCl_3$) δ -62.16.

HRMS (ESI-TOF) m/z: $[M + H]^+$ calculated for $C_{18}H_{15}F_3NO$ 318.1100; Found 318.1110.

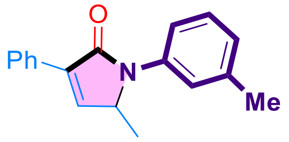

5-Methyl-3-phenyl-1-(m-tolyl)-1,5-dihydro-2H-pyrrol-2-one (7l):

White solid, 18.3 mg, 70% yield, R_f=0.2 (PE/EtOAc 10/1).

^{1}H NMR (400 MHz, $CDCl_3$) δ 7.96 – 7.89 (m, 2H), 7.44 – 7.32 (m, 5H), 7.30 (d, J = 7.3 Hz, 1H), 7.26 (d, J = 3.1 Hz, 1H), 7.06 – 7.01 (m, 1H), 4.78 (qd, J = 6.8, 2.2 Hz, 1H), 2.40 (s, 3H), 1.33 (d, J = 6.8 Hz, 3H).

^{13}C NMR (100 MHz, $CDCl_3$) δ 168.2, 141.7, 139.0, 136.9, 136.0, 131.5, 128.8, 128.7, 128.5, 127.3, 126.0, 123.6, 119.7, 56.3, 21.6, 17.4.

HRMS (ESI-TOF) m/z: $[M + H]^+$ calculated for $C_{18}H_{18}NO$ 264.1383; Found 264.1390.

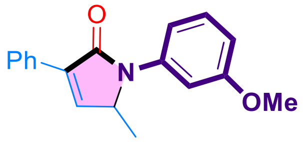

1-(3-Methoxyphenyl)-5-methyl-3-phenyl-1,5-dihydro-2H-pyrrol-2-one (7j):

Yellow solid, 14.5 mg, 52% yield, R_f=0.2 (PE/EtOAc 5/1).

^{1}H NMR (400 MHz, $CDCl_3$) δ 7.98 – 7.84 (m, 2H), 7.45 – 7.39 (m, 2H), 7.39 – 7.35 (m, 1H), 7.32 (t, J = 8.1 Hz, 1H), 7.29 (t, J = 2.3 Hz, 1H), 7.27 (d, J = 2.2 Hz, 1H), 7.04 (ddd, J = 8.1, 2.1, 0.9 Hz, 1H), 6.77 (ddd, J = 8.3, 2.5, 0.9 Hz, 1H), 4.78 (qd, J = 6.8, 2.1 Hz, 1H), 3.84 (s, 3H), 1.36 (d, J = 6.8 Hz, 3H).

^{13}C NMR (100 MHz, $CDCl_3$) δ 168.3, 160.2, 141.8, 138.3, 136.1, 131.4, 129.7, 128.7, 128.5, 127.3, 114.4, 110.9, 108.4, 56.3, 55.4, 17.4.

HRMS (ESI-TOF) m/z: $[M + H]^+$ calculated for $C_{18}H_{18}NO_2$ 280.1332; Found 280.1340.

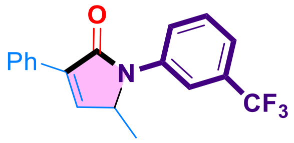

5-Methyl-3-phenyl-1-(3-(trifluoromethyl)phenyl)-1,5-dihydro-2H-pyrrol-2-one (7k):
Yellow solid, 17.2 mg, 54% yield, R_f=0.2 (PE/EtOAc 10/1).
^{1}H NMR (400 MHz, $CDCl_3$) δ 7.90 (ddd, J = 9.0, 7.2, 1.9 Hz, 3H), 7.82 – 7.75 (m, 1H), 7.55 (t, J = 7.9 Hz, 1H), 7.47 – 7.35 (m, 4H), 7.31 (d, J = 2.2 Hz, 1H), 4.85 (qd, J = 6.8, 2.2 Hz, 1H), 1.38 (d, J = 6.8 Hz, 3H).
^{13}C NMR (100 MHz, $CDCl_3$) δ 168.3, 142.0, 137.7, 136.0, 131.5 (q, J = 32.6 Hz), 131.1, 129.6, 128.9, 128.6, 127.3, 125.1, 123.9 (q, J = 272.5 Hz), 121.4 (q, J = 4.0 Hz), 118.7 (q, J = 4.0 Hz), 55.9, 17.3.
^{19}F NMR (376 MHz, $CDCl_3$) δ -62.62.
HRMS (ESI-TOF) m/z: $[M + H]^+$ calculated for $C_{18}H_{15}F_3NO$ 318.1100; Found 318.1108.

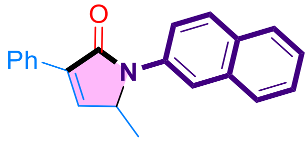

5-Methyl-1-(naphthalen-2-yl)-3-phenyl-1,5-dihydro-2H-pyrrol-2-one (7l):
Brown oil, 12.7 mg, 42% yield, R_f=0.2 (PE/EtOAc 10/1).
^{1}H NMR (400 MHz, $CDCl_3$) δ 8.01 – 7.93 (m, 3H), 7.90 (d, J = 8.8 Hz, 1H), 7.84 (dd, J = 7.8, 1.6 Hz, 2H), 7.76 (dd, J = 8.9, 2.1 Hz, 1H), 7.48 (ddd, J = 8.9, 7.4, 1.8 Hz, 2H), 7.45 – 7.41 (m, 2H), 7.40 – 7.37 (m, 1H), 7.32 (d, J = 2.1 Hz, 1H), 4.94 (qd, J = 6.8, 2.2 Hz, 1H), 1.38 (d, J = 6.8 Hz, 3H).
^{13}C NMR (100 MHz, $CDCl_3$) δ 168.4, 141.8, 136.1, 134.6, 133.7, 131.4, 131.0, 128.8, 128.8, 128.5, 127.7, 127.6, 127.3, 126.5, 125.5, 121.9, 120.1, 56.4, 17.5.
HRMS (ESI-TOF) m/z: $[M + H]^+$ calculated for $C_{21}H_{18}NO$ 300.1383; Found 300.1390.

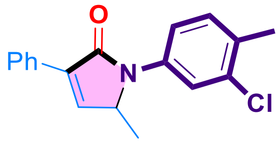

1-(3-Chloro-4-methylphenyl)-5-methyl-3-phenyl-1,5-dihydro-2H-pyrrol-2-one (7m):
Yellow solid, 15.9 mg, 54% yield, R_f=0.2 (PE/EtOAc 10/1).
^{1}H NMR (700 MHz, $CDCl_3$) δ 7.90 (d, J = 7.3 Hz, 2H), 7.59 (d, J = 2.1 Hz, 1H), 7.41 (t, J = 7.5 Hz, 2H), 7.38 – 7.34 (m, 2H), 7.26 (dd, J = 5.1, 3.1 Hz, 2H), 4.73 (qd, J = 6.8, 1.9 Hz, 1H), 2.37 (s, 3H), 1.34 (d, J = 6.9 Hz, 3H).
^{13}C NMR (176 MHz, $CDCl_3$) δ 168.2, 141.8, 136.0, 135.9, 134.6, 132.7, 131.3, 131.1, 128.8, 128.5, 127.3, 123.0, 120.7, 56.1, 19.6, 17.3.
HRMS (ESI-TOF) m/z: $[M + H]^+$ calculated for $C_{18}H_{17}ClNO$ 298.0993; Found 298.1002.

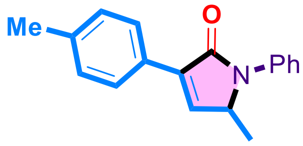

5-Methyl-1-phenyl-3-(p-tolyl)-1,5-dihydro-2H-pyrrol-2-one (7o):
Brown solid, 16.2 mg, 62% yield, R_f=0.2 (PE/EtOAc 10/1).
^{1}H NMR (400 MHz, $CDCl_3$) δ 7.87 – 7.78 (m, 2H), 7.58 – 7.52 (m, 2H), 7.45 – 7.40 (m, 2H), 7.25 –

7.18 (m, 4H), 4.79 (qd, J = 6.8, 2.2 Hz, 1H), 2.38 (s, 3H), 1.33 (d, J = 6.8 Hz, 3H).

^{13}C NMR (100 MHz, $CDCl_3$) δ 168.4, 140.8, 138.6, 137.1, 135.9, 129.2, 129.0, 128.6, 127.1, 125.0, 122.6, 56.1, 21.4, 17.4.

HRMS (ESI-TOF) m/z: $[M + H]^+$ calculated for $C_{18}H_{18}NO$ 264.1383; Found 264.1383.

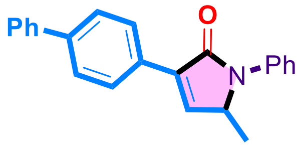

3-([1,1'-Biphenyl]-4-yl)-5-methyl-1-phenyl-1,5-dihydro-2H-pyrrol-2-one (7p):

White solid, 19.8 mg, 61% yield, R_f=0.2 (PE/EtOAc 10/1).

^{1}H NMR (400 MHz, $CDCl_3$) δ 8.02 (d, J = 8.4 Hz, 2H), 7.68 – 7.61 (m, 4H), 7.58 – 7.54 (m, 2H), 7.47 – 7.41 (m, 4H), 7.38 – 7.34 (m, 1H), 7.31 (d, J = 2.1 Hz, 1H), 7.24 – 7.19 (m, 1H), 4.82 (qd, J = 6.9, 2.2 Hz, 1H), 1.35 (d, J = 6.8 Hz, 3H).

^{13}C NMR (100 MHz, $CDCl_3$) δ 168.3, 141.6, 141.4, 140.7, 137.0, 135.6, 130.4, 129.1, 128.8, 127.7, 127.5, 127.2, 127.1, 125.1, 122.7, 56.2, 17.4.

HRMS (ESI-TOF) m/z: $[M + H]^+$ calculated for $C_{23}H_{20}NO$ 326.1539; Found 326.1545.

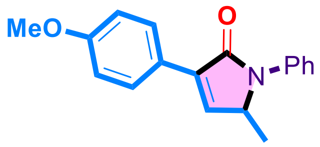

3-(4-Methoxyphenyl)-5-methyl-1-phenyl-1,5-dihydro-2H-pyrrol-2-one (7q):

White solid, 16.5 mg, 59% yield, R_f=0.2 (PE/EtOAc 5/1).

^{1}H NMR (400 MHz, $CDCl_3$) δ 7.90 (d, J = 8.9 Hz, 2H), 7.59 – 7.51 (m, 2H), 7.42 (dd, J = 8.6, 7.5 Hz, 2H), 7.20 (tt, J = 7.1, 1.2 Hz, 1H), 7.17 (d, J = 2.2 Hz, 1H), 6.94 (d, J = 9.0 Hz, 2H), 4.78 (qd, J = 6.8, 2.2 Hz, 1H), 3.84 (s, 3H), 1.32 (d, J = 6.8 Hz, 3H).

^{13}C NMR (100 MHz, $CDCl_3$) δ 168.5, 160.0, 139.7, 137.1, 135.3, 129.0, 128.6, 125.0, 124.1, 122.7, 113.9, 56.0, 55.3, 17.4.

HRMS (ESI-TOF) m/z: $[M + H]^+$ calculated for $C_{18}H_{18}NO_2$ 280.1332; Found 280.1340.

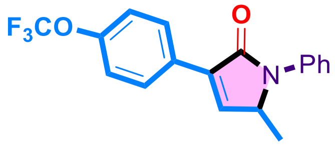

5-Methyl-1-phenyl-3-(4-(trifluoromethoxy)phenyl)-1,5-dihydro-2H-pyrrol-2-one (7r):

White solid, 17.7 mg, 53% yield, R_f=0.2 (PE/EtOAc 5/1).

^{1}H NMR (400 MHz, $CDCl_3$) δ 8.07 – 7.90 (m, 2H), 7.58 – 7.48 (m, 2H), 7.48 – 7.39 (m, 2H), 7.32 – 7.25 (m, 2H), 7.28 – 7.18 (m, 2H), 4.82 (qd, J = 6.8, 2.1 Hz, 1H), 1.35 (d, J = 6.8 Hz, 3H).

^{13}C NMR (100 MHz, $CDCl_3$) δ 167.9, 149.4, 142.2, 136.8, 134.9, 130.1, 129.1, 128.8, 125.3, 122.7, 120.9, 120.5 (q, J = 257.5 Hz), 56.3, 17.3.

^{19}F NMR (376 MHz, $CDCl_3$) δ -57.73.

HRMS (ESI-TOF) m/z: $[M + H]^+$ calculated for $C_{18}H_{15}F_3NO_2$ 334.1049; Found 334.1054.

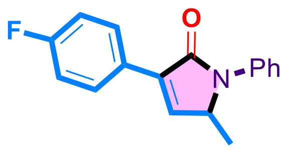

3-(4-Fluorophenyl)-5-methyl-1-phenyl-1,5-dihydro-2H-pyrrol-2-one (7s):

White solid, 16.9 mg, 63% yield, R_f=0.2 (PE/EtOAc 10/1).

^{1}H NMR (400 MHz, $CDCl_3$) δ 7.98 – 7.88 (m, 2H), 7.57 – 7.49 (m, 2H), 7.43 (t, J = 7.9 Hz, 2H), 7.25 – 7.18 (m, 2H), 7.14 – 7.06 (m, 2H), 4.80 (qd, J = 6.8, 2.2 Hz, 1H), 1.34 (d, J = 6.8 Hz, 3H).

^{13}C NMR (100 MHz, $CDCl_3$) δ 168.1, 163.0 (d, J = 248.3 Hz), 141.3 (d, J = 1.8 Hz), 136.9, 135.0, 129.2, 129.1, 127.6 (d, J = 3.3 Hz), 125.2, 122.7, 115.5 (d, J = 21.3 Hz), 56.2, 17.3.

^{19}F NMR (376 MHz, $CDCl_3$) δ -112.48.

HRMS (ESI-TOF) m/z: $[M + H]^+$ calculated for $C_{17}H_{15}FNO$ 268.1132; Found 268.1140.

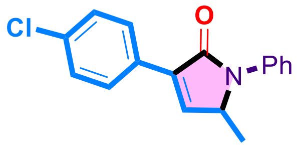

3-(4-Chlorophenyl)-5-methyl-1-phenyl-1,5-dihydro-2H-pyrrol-2-one (7t):

White solid, 16.8 mg, 59% yield, R_f=0.2 (PE/EtOAc 10/1).

^{1}H NMR (400 MHz, $CDCl_3$) δ 7.96 – 7.83 (m, 2H), 7.56 – 7.49 (m, 2H), 7.46 – 7.41 (m, 2H), 7.40 – 7.35 (m, 2H), 7.28 (d, J = 2.2 Hz, 1H), 7.25 – 7.19 (m, 1H), 4.80 (qd, J = 6.8, 2.1 Hz, 1H), 1.34 (d, J = 7.0 Hz, 3H).

^{13}C NMR (100 MHz, $CDCl_3$) δ 167.9, 141.9, 136.8, 134.9, 134.7, 129.9, 129.1, 128.7, 128.6, 125.3, 122.7, 56.2, 17.3.

HRMS (ESI-TOF) m/z: $[M + H]^+$ calculated for $C_{17}H_{15}ClNO$ 284.0837; Found 284.0845.

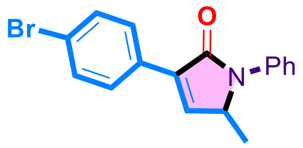

3-(4-Bromophenyl)-5-methyl-1-phenyl-1,5-dihydro-2H-pyrrol-2-one (7u):

Yellow solid, 17.3 mg, 53% yield, R_f=0.2 (PE/EtOAc 10/1).

^{1}H NMR (400 MHz, $CDCl_3$) δ 7.88 – 7.79 (m, 2H), 7.58 – 7.48 (m, 4H), 7.47 – 7.40 (m, 2H), 7.30 (d, J = 2.2 Hz, 1H), 7.25 – 7.19 (m, 1H), 4.80 (qd, J = 7.0, 2.2 Hz, 1H), 1.34 (d, J = 6.8 Hz, 3H).

^{13}C NMR (100 MHz, $CDCl_3$) δ 167.9, 141.9, 136.8, 135.0, 131.7, 130.3, 129.1, 128.8, 125.3, 123.0, 122.7, 56.3, 17.3.

HRMS (ESI-TOF) m/z: $[M + H]^+$ calculated for $C_{17}H_{15}BrNO$ 328.0332; Found 328.0341.

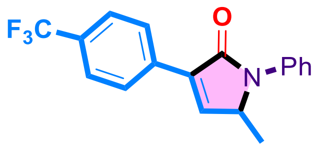

5-Methyl-1-phenyl-3-(4-(trifluoromethyl)phenyl)-1,5-dihydro-2H-pyrrol-2-one (7v):

Yellow solid, 21.6 mg, 68% yield, R_f=0.2 (PE/EtOAc 10/1).

^{1}H NMR (700 MHz, $CDCl_3$) δ 8.06 (d, J = 8.4 Hz, 2H), 7.67 (d, J = 8.4 Hz, 2H), 7.55 – 7.51 (m, 2H), 7.44 (t, J = 8.0 Hz, 2H), 7.39 (d, J = 2.2 Hz, 1H), 7.25 – 7.21 (m, 1H), 4.84 (qd, J = 6.9, 2.2 Hz, 1H), 1.36 (d, J = 6.9 Hz, 3H).

^{13}C NMR (176 MHz, $CDCl_3$) δ 167.7, 143.4, 136.7, 135.0, 134.9, 130.5 (q, J = 32.8 Hz), 129.2, 127.6, 125.4 (q, J = 2.6 Hz), 125.4, 124.1 (q, J = 271.8 Hz), 122.8, 56.4, 17.2.
^{19}F NMR (376 MHz, $CDCl_3$) δ -62.67.
HRMS (ESI-TOF) m/z: $[M + H]^+$ calculated for $C_{18}H_{15}F_3NO$ 318.1100; Found 318.1111.

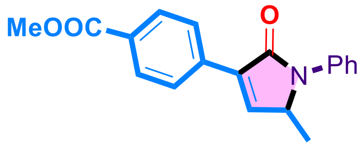

Methyl-4-(5-methyl-2-oxo-1-phenyl-2,5-dihydro-1H-pyrrol-3-yl)benzoate (7w):
Yellow solid, 12.6 mg, 41% yield, R_f=0.2 (PE/EtOAc 10/1).
^{1}H NMR (700 MHz, $CDCl_3$) δ 8.11 – 8.06 (m, 2H), 8.05 – 8.01 (m, 2H), 7.56 – 7.51 (m, 2H), 7.44 (dd, J = 8.5, 7.4 Hz, 2H), 7.40 (d, J = 2.2 Hz, 1H), 7.23 (tt, J = 7.4, 1.2 Hz, 1H), 4.84 (qd, J = 6.9, 2.2 Hz, 1H), 3.93 (s, 3H), 1.36 (d, J = 6.9 Hz, 3H).
^{13}C NMR (176 MHz, $CDCl_3$) δ 167.7, 166.9, 143.4, 136.8, 135.8, 135.3, 130.0, 129.7, 129.1, 127.2, 125.3, 122.8, 56.4, 52.2, 17.2.
HRMS (ESI-TOF) m/z: $[M + H]^+$ calculated for $C_{19}H_{18}NO_3$ 308.1281; Found 328.1293.

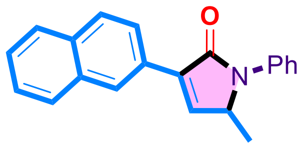

5-Methyl-3-(naphthalen-2-yl)-1-phenyl-1,5-dihydro-2H-pyrrol-2-one (7x):
Brown solid, 18.0 mg, 60% yield, R_f=0.2 (PE/EtOAc 10/1).
^{1}H NMR (400 MHz, $CDCl_3$) δ 8.76 – 8.68 (m, 1H), 7.94 – 7.90 (m, 1H), 7.86 (d, J = 1.3 Hz, 2H), 7.84 – 7.81 (m, 1H), 7.59 – 7.55 (m, 2H), 7.49 (dd, J = 6.2, 3.2 Hz, 2H), 7.47 – 7.42 (m, 2H), 7.38 (d, J = 2.1 Hz, 1H), 7.25 – 7.19 (m, 1H), 4.82 (qd, J = 6.8, 2.2 Hz, 1H), 1.36 (d, J = 6.8 Hz, 3H).
^{13}C NMR (100MHz, $CDCl_3$) δ 168.4, 141.9, 137.0, 135.6, 133.4, 133.3, 129.1, 128.9, 128.7, 128.1, 127.6, 126.9, 126.5, 126.3, 125.2, 124.6, 122.7, 56.2, 17.4.
HRMS (ESI-TOF) m/z: $[M + H]^+$ calculated for $C_{21}H_{18}NO$ 300.1383; Found 300.1386.

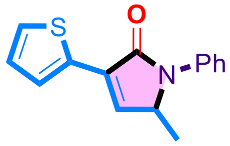

5-Methyl-1-phenyl-3-(thiophen-2-yl)-1,5-dihydro-2H-pyrrol-2-one (7z):
Brown solid, 11.4 mg, 45% yield, R_f=0.2 (PE/EtOAc 10/1).
^{1}H NMR (400 MHz, $CDCl_3$) δ 7.79 (dd, J = 3.7, 1.3 Hz, 1H), 7.57 – 7.51 (m, 2H), 7.46 – 7.40 (m, 2H), 7.37 (dd, J = 5.1, 1.2 Hz, 1H), 7.24 – 7.19 (m, 1H), 7.17 (d, J = 2.3 Hz, 1H), 7.10 (dd, J = 5.1, 3.6 Hz, 1H), 4.83 (qd, J = 6.8, 2.3 Hz, 1H), 1.34 (d, J = 6.8 Hz, 3H).
^{13}C NMR (100MHz, $CDCl_3$) δ 167.4, 137.9, 136.8, 133.1, 130.8, 129.1, 127.4, 126.7, 126.5, 125.2, 122.6, 56.7, 17.4.
HRMS (ESI-TOF) m/z: $[M + H]^+$ calculated for $C_{15}H_{14}NOS$ 256.0791; Found 256.0800.

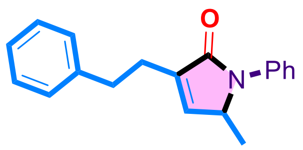

5-Methyl-3-phenethyl-1-phenyl-1,5-dihydro-2H-pyrrol-2-one (7aa):

White solid, 18.9 mg, 68% yield, R_f=0.2 (PE/EtOAc 10/1).

^{1}H NMR (400 MHz, $CDCl_3$) δ 7.55 – 7.48 (m, 2H), 7.40 (dd, J = 8.7, 7.3 Hz, 2H), 7.29 (t, J = 7.3 Hz, 2H), 7.26 – 7.13 (m, 4H), 6.64 (q, J = 1.7 Hz, 1H), 4.62 (qd, J = 6.8, 1.8 Hz, 1H), 2.93 (t, J = 7.4 Hz, 2H), 2.73 – 2.61 (m, 2H), 1.20 (d, J = 6.8 Hz, 3H).

^{13}C NMR (100MHz, $CDCl_3$) δ 169.8, 141.7, 141.3, 138.1, 137.1, 129.0, 128.5, 128.4, 126.0, 124.7, 122.1, 56.6, 33.6, 27.4, 17.3.

HRMS (ESI-TOF) m/z: $[M + H]^+$ calculated for $C_{19}H_{20}NO$ 278.1539; Found 275.1548.

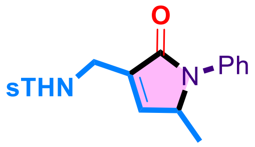

4-Methyl-N-((5-methyl-2-oxo-1-phenyl-2,5-dihydro-1H-pyrrol-3-yl)methyl)benzenesulfonamide (7ab):

Brown solid, 16.8 mg, 47% yield, R_f=0.2 (PE/EtOAc 5/1).

^{1}H NMR (400 MHz, $CDCl_3$) δ 7.80 – 7.67 (m, 2H), 7.40 (dd, J = 8.7, 7.0 Hz, 2H), 7.38 – 7.31 (m, 2H), 7.26 (d, J = 1.3 Hz, 1H), 7.24 (s, 1H), 7.24 – 7.13 (m, 1H), 6.83 (q, J = 1.5 Hz, 1H), 5.68 (t, J = 6.4 Hz, 1H), 4.50 (qd, J = 6.8, 1.7 Hz, 1H), 3.95 (dt, J = 6.4, 1.5 Hz, 2H), 2.38 (s, 3H), 1.13 (d, J = 6.8 Hz, 3H).

^{13}C NMR (100MHz, $CDCl_3$) δ 168.5, 143.5, 143.4, 137.1, 136.2, 133.4, 129.6, 129.1, 127.3, 125.4, 122.3, 57.3, 39.8, 21.5, 16.7.

HRMS (ESI-TOF) m/z: $[M + H]^+$ calculated for $C_{19}H_{21}N_2O_3S$ 357.1267; Found 357.1268.

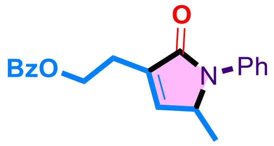

2-(5-Methyl-2-oxo-1-phenyl-2,5-dihydro-1H-pyrrol-3-yl)ethyl benzoate (7ac):

Yellow oil, 20.6 mg, 64% yield, R_f=0.2 (PE/EtOAc 10/1).

^{1}H NMR (400 MHz, $CDCl_3$) δ 8.09 – 7.99 (m, 2H), 7.59 – 7.54 (m, 1H), 7.53 – 7.48 (m, 2H), 7.47 – 7.38 (m, 4H), 7.19 (td, J = 7.3, 1.2 Hz, 1H), 6.90 (q, J = 1.6 Hz, 1H), 4.72 – 4.65 (m, 1H), 4.65 – 4.53 (m, 2H), 2.86 (tt, J = 6.6, 1.6 Hz, 2H), 1.24 (d, J = 6.8 Hz, 3H).

^{13}C NMR (100MHz, $CDCl_3$) δ 169.4, 166.5, 142.9, 136.9, 135.1, 133.0, 130.1, 129.6, 129.1, 128.4, 124.9, 122.2, 62.6, 56.9, 25.4, 17.2.

HRMS (ESI-TOF) m/z: $[M + H]^+$ calculated for $C_{20}H_{20}NO_3$ 322.1438; Found 322.1442.

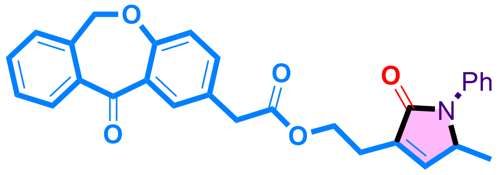

2-(5-Methyl-2-oxo-1-phenyl-2,5-dihydro-1H-pyrrol-3-yl)ethyl 2-(11-oxo-6,11-dihydrodibenzo[b,e]oxepin-2-yl)acetate (7ad):

Yellow oil, 32.5 mg, 70% yield, R_f=0.2 (PE/EtOAc 2/1).

^{1}H NMR (700 MHz, $CDCl_3$) δ 8.11 (d, J = 2.6 Hz, 1H), 7.90 – 7.86 (m, 1H), 7.56 (td, J = 7.4, 1.2 Hz, 1H), 7.49 – 7.45 (m, 3H), 7.43 (dd, J = 8.4, 2.5 Hz, 1H), 7.40 – 7.35 (m, 3H), 7.16 (t, J = 7.4 Hz, 1H), 7.02 (d, J = 8.4 Hz, 1H), 6.73 (d, J = 1.7 Hz, 1H), 5.17 (s, 2H), 4.61 (qd, J = 6.8, 1.7 Hz, 1H), 4.41 – 4.31 (m, 2H), 3.65 (s, 2H), 2.69 (td, J = 6.5, 1.9 Hz, 2H), 1.18 (d, J = 6.9 Hz, 3H).

^{13}C NMR (176 MHz, $CDCl_3$) δ 190.8, 171.2, 169.3, 160.5, 143.0, 140.4, 136.9, 136.4, 135.6, 134.8, 132.9, 132.4, 129.5, 129.3, 129.0, 127.9, 127.8, 125.1, 124.8, 122.1, 121.1, 73.6, 62.6, 56.8, 40.4, 25.2, 17.1.

HRMS (ESI-TOF) m/z: $[M + H]^+$ calculated for $C_{29}H_{26}NO_5$ 468.1805; Found 468.1816.

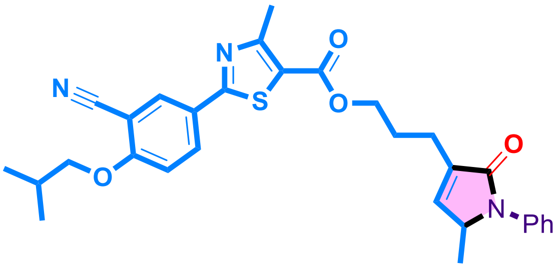

3-(5-Methyl-2-oxo-1-phenyl-2,5-dihydro-1H-pyrrol-3-yl)propyl 2-(3-cyano-4-isobutoxyphenyl)-4-methylthiazole-5-carboxylate (7ae):

White solid, 33.6 mg, 64% yield, R_f=0.2 (PE/EtOAc 2/1).

^{1}H NMR (700 MHz, $CDCl_3$) δ 8.16 (d, J = 2.3 Hz, 1H), 8.06 (dd, J = 8.8, 2.3 Hz, 1H), 7.50 – 7.48 (m, 2H), 7.39 (dd, J = 8.6, 7.4 Hz, 2H), 7.17 (tt, J = 7.4, 1.3 Hz, 1H), 7.00 (d, J = 8.8 Hz, 1H), 6.82 (q, J = 1.7 Hz, 1H), 4.66 (qd, J = 6.9, 1.8 Hz, 1H), 4.37 (td, J = 6.3, 2.3 Hz, 2H), 3.90 (d, J = 6.5 Hz, 2H), 2.77 (s, 3H), 2.55 – 2.49 (m, 2H), 2.22 – 2.18 (m, 1H), 2.13 – 2.07 (m, 2H), 1.26 (d, J = 6.9 Hz, 3H), 1.09 (d, J = 6.8 Hz, 6H).

^{13}C NMR (176 MHz, $CDCl_3$) δ 169.5, 167.3, 162.5, 162.0, 161.3, 141.6, 137.8, 137.0, 132.6, 132.1, 129.0, 126.0, 124.8, 122.0, 121.6, 115.4, 112.6, 103.0, 75.7, 64.7, 56.7, 28.2, 26.5, 22.6, 19.1, 17.5, 17.3.

HRMS (ESI-TOF) m/z: $[M + H]^+$ calculated for $C_{30}H_{32}N_3O_4S$ 530.2108; Found 530.2121.

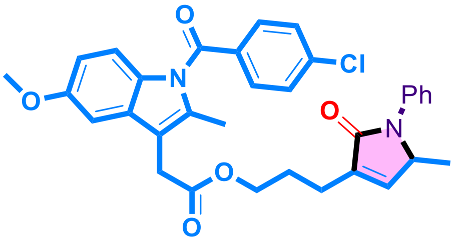

3-(5-Methyl-2-oxo-1-phenyl-2,5-dihydro-1H-pyrrol-3-yl)propyl 2-(1-(4-chlorobenzoyl)-5-methoxy-2-methyl-1H-indol-3-yl)acetate (7af):

Yellow oil, 34.9 mg, 61% yield, R_f=0.2 (PE/EtOAc 2/1).

^{1}H NMR (700 MHz, $CDCl_3$) δ 7.68 – 7.64 (m, 2H), 7.49 – 7.45 (m, 4H), 7.41 – 7.37 (m, 2H), 7.17 (tt, J = 7.4, 1.2 Hz, 1H), 6.99 (d, J = 2.6 Hz, 1H), 6.88 (d, J = 8.9 Hz, 1H), 6.67 (dd, J = 9.0, 2.5 Hz, 1H), 6.64 (q, J = 1.6 Hz, 1H), 4.62 (qd, J = 6.8, 3.4 Hz, 1H), 4.16 (t, J = 6.5 Hz, 2H), 3.83 (s, 3H), 3.68 (s, 2H), 2.39 (s, 3H), 2.38 – 2.35 (m, 2H), 1.94 (dq, J = 8.4, 6.5 Hz, 2H), 1.22 (d, J = 6.9 Hz, 3H).

^{13}C NMR (176 MHz, $CDCl_3$) δ 170.9, 169.5, 168.3, 156.1, 141.6, 139.3, 137.7, 137.0, 135.9, 133.9, 131.2, 130.8, 130.7, 129.1, 129.0, 124.8, 122.1, 115.0, 112.7, 111.7, 101.3, 64.2, 56.6, 55.7, 30.4, 26.4, 22.2, 17.3, 13.4.

HRMS (ESI-TOF) m/z: $[M + H]^+$ calculated for $C_{33}H_{32}ClN_2O_5$ 571.1994; Found 571.1997.

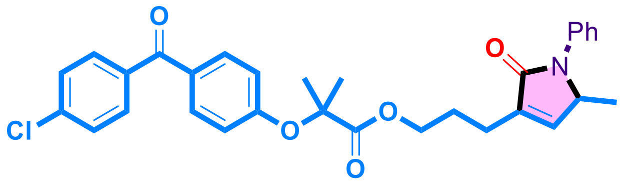

3-(5-Methyl-2-oxo-1-phenyl-2,5-dihydro-1H-pyrrol-3-yl)propyl 2-(4-(4-chlorobenzoyl)phenoxy)-2-methylpropanoate (7ag):

Yellow oil, 34.6 mg, 65% yield, R_f=0.2 (PE/EtOAc 2/1).

^{1}H NMR (700 MHz, $CDCl_3$) δ 7.74 (d, J = 8.8 Hz, 2H), 7.69 (d, J = 8.4 Hz, 2H), 7.46 (d, J = 7.7 Hz, 2H), 7.42 (d, J = 8.4 Hz, 2H), 7.38 (t, J = 7.9 Hz, 2H), 7.16 (t, J = 7.4 Hz, 1H), 6.88 (d, J = 8.8 Hz, 2H), 6.67 – 6.63 (m, 1H), 4.65 – 4.59 (m, 1H), 4.27 – 4.21 (m, 2H), 2.29 (t, J = 7.6 Hz, 2H), 1.92 (p, J = 6.6 Hz, 2H), 1.70 (d, J = 1.9 Hz, 6H), 1.22 (d, J = 6.8 Hz, 3H).

^{13}C NMR (176 MHz, $CDCl_3$) δ 194.2, 173.7, 169.4, 159.7, 141.6, 138.4, 137.6, 136.9, 136.3, 132.1, 131.2, 130.3, 129.0, 128.6, 124.8, 122.1, 117.2, 79.4, 65.0, 56.7, 26.4, 25.5, 25.5, 22.2, 17.3.

HRMS (ESI-TOF) m/z: $[M + H]^+$ calculated for $C_{31}H_{31}ClNO_5$ 532.1885; Found 532.1900.

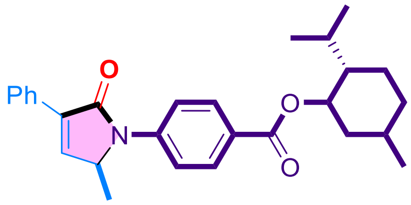

2-Isopropyl-5-methylcyclohexyl 4-((S)-5-methyl-2-oxo-3-phenyl-2,5-dihydro-1H-pyrrol-1-yl)benzoate (7aj):

White solid, 15.9 mg, 37% yield, R_f=0.2 (PE/EtOAc 10/1).

^{1}H NMR (700 MHz, $CDCl_3$) δ 8.11 (dd, J = 8.7, 3.1 Hz, 2H), 7.90 (d, J = 7.3 Hz, 2H), 7.69 (dd, J = 8.7, 3.2 Hz, 2H), 7.43 (t, J = 7.5 Hz, 2H), 7.39 (d, J = 7.2 Hz, 1H), 7.31 (d, J = 1.9 Hz, 1H), 4.94 (tt, J = 10.7, 4.7 Hz, 1H), 4.87 (q, J = 6.8 Hz, 1H), 2.15 – 2.12 (m, 1H), 2.00 – 1.95 (m, 1H), 1.74 (d, J = 12.2 Hz, 2H), 1.59 – 1.55 (m, 2H), 1.39 (dd, J = 6.8, 1.7 Hz, 3H), 1.16 – 1.10 (m, 2H), 0.94 – 0.92 (m, 7H), 0.80 (d, J = 6.9 Hz, 3H).

^{13}C NMR (100 MHz, $CDCl_3$) δ 168.3, 165.7, 142.1, 141.1, 136.1, 131.1, 130.6, 128.9, 128.5, 127.3, 126.7, 120.9, 74.8, 55.7, 47.3, 41.0, 34.3, 31.5, 26.5, 23.7, 22.1, 20.8, 17.3, 16.6.

HRMS (ESI-TOF) m/z: $[M + H]^+$ calculated for $C_{28}H_{34}NO_3$ 432.2533; Found 432.2539.

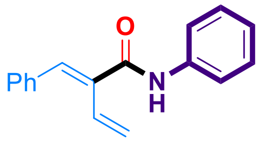

(*E*)-2-benzylidene-N-phenylbut-3-enamide (3a):

White solid, 23.2 mg, 93% yield (*E:Z* = 5.6), R_f=0.2 (PE/EtOAc 10/1). NMR data for main isomer **3a** was provided.

^{1}H NMR (700 MHz, $CDCl_3$) δ 7.82 (s, 1H), 7.61 (d, J = 7.9 Hz, 2H), 7.42 (t, J = 6.8 Hz, 3H), 7.40 – 7.32 (m, 5H), 7.14 (t, J = 7.4 Hz, 1H), 6.81 (dd, J = 18.0, 11.4 Hz, 1H), 5.70 (d, J = 18.0 Hz, 1H), 5.62 (d, J = 11.4 Hz, 1H).

^{13}C NMR (176 MHz, $CDCl_3$) δ 165.9, 137.9, 135.9, 135.3, 134.6, 131.9, 130.1, 129.1, 128.7, 128.4, 124.5, 121.6, 119.9.

HRMS (ESI-TOF) m/z: $[M + H]^+$ calculated for $C_{17}H_{16}NO$ 250.1226; Found 250.1235.

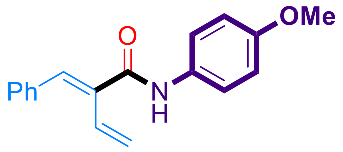

(*E*)-2-benzylidene-N-(4-methoxyphenyl)but-3-enamide (3b):

White solid, 22.5 mg, 80% yield (*E:Z* = 2.8), R_f=0.2 (PE/EtOAc 5/1). NMR data for main isomer **3b** was provided.

^{1}H NMR (400 MHz, $CDCl_3$) δ 7.74 (s, 1H), 7.52 (d, J = 8.9 Hz, 2H), 7.46 – 7.30 (m, 6H), 6.94 – 6.85 (m, 2H), 6.85 – 6.74 (m, 1H), 5.76 – 5.65 (m, 1H), 5.60 (d, J = 11.4 Hz, 1H), 3.80 (s, 3H).

^{13}C NMR (100 MHz, $CDCl_3$) δ 165.7, 156.6, 135.6, 135.3, 134.7, 132.0, 131.0, 130.1, 128.6, 128.4, 121.7, 121.5, 114.2, 55.5.

HRMS (ESI-TOF) m/z: $[M + H]^+$ calculated for $C_{18}H_{18}NO_2$ 280.1332; Found 280.1342.

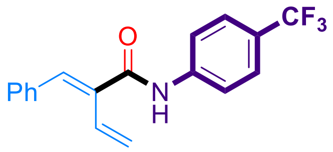

(*E*)-2-benzylidene-N-(4-(trifluoromethyl)phenyl)but-3-enamide (3c):

White solid, 19.5 mg, 61% yield (*E:Z* = 4), R_f=0.2 (PE/EtOAc 10/1). NMR data for main isomer **3c** was provided.

^{1}H NMR (400 MHz, $CDCl_3$) δ 7.97 (s, 1H), 7.74 (d, J = 8.5 Hz, 2H), 7.61 (d, J = 8.6 Hz, 2H), 7.48 – 7.32 (m, 6H), 6.82 (dd, J = 18.0, 11.4 Hz, 1H), 5.74 – 5.60 (m, 2H).

^{13}C NMR (100 MHz, $CDCl_3$) δ 166.0, 140.9, 136.8, 135.0, 134.0, 131.9, 130.2, 129.0, 128.5, 127.4 (q, J = 3.8 Hz), 124.1 (q, J = 271.6 Hz), 121.9, 119.5.

HRMS (ESI-TOF) m/z: $[M + H]^+$ calculated for $C_{18}H_{15}F_3NO$ 318.1100; Found 318.1110.

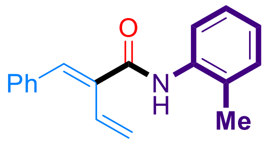

(*E*)-2-benzylidene-N-(o-tolyl)but-3-enamide (3d):

Brown solid, 23.4 mg, 89% yield (*E:Z* = 4.4), R_f=0.2 (PE/EtOAc 10/1). NMR data for main isomer **3d** was provided.

^{1}H NMR (400 MHz, $CDCl_3$) δ 8.08 (d, J = 7.9 Hz, 1H), 7.76 (s, 1H), 7.54 – 7.43 (m, 3H), 7.41 – 7.32 (m, 3H), 7.28 – 7.23 (m, 1H), 7.20 (d, J = 7.3 Hz, 1H), 7.12 – 7.06 (m, 1H), 6.84 (dd, J = 17.8, 11.3 Hz, 1H), 5.81 – 5.71 (m, 1H), 5.66 (d, J = 11.4 Hz, 1H), 2.27 (s, 3H).

^{13}C NMR (100 MHz, $CDCl_3$) δ 165.6, 136.3, 136.0, 135.3, 134.3, 132.5, 130.5, 130.2, 128.7, 128.4, 127.0, 125.0, 122.3, 121.7, 18.0.

HRMS (ESI-TOF) m/z: $[M + H]^+$ calculated for $C_{18}H_{18}NO$ 264.1383; Found 264.1388.

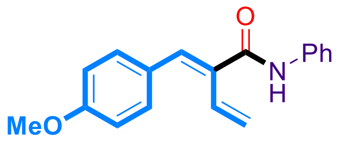

(*E*)-2-(4-methoxybenzylidene)-N-phenylbut-3-enamide (3e):

Brown solid, 21.4 mg, 77% yield (*E:Z* = 5.9), R_f=0.2 (PE/EtOAc 10/1). NMR data for main isomer **3e** was provided.

^{1}**H NMR** (400 MHz, $CDCl_3$) δ 7.85 (s, 1H), 7.61 (d, J = 7.7 Hz, 2H), 7.41 (d, J = 8.7 Hz, 3H), 7.35 (t, J = 7.9 Hz, 2H), 7.13 (t, J = 7.4 Hz, 1H), 6.91 (d, J = 8.8 Hz, 2H), 6.81 (ddd, J = 18.0, 11.4, 1.1 Hz, 1H), 5.73 – 5.65 (m, 1H), 5.63 (d, J = 11.4 Hz, 1H), 3.84 (s, 3H).

^{13}**C NMR** (100 MHz, $CDCl_3$) δ 165.9, 160.1, 138.0, 136.1, 132.5, 132.3, 131.9, 129.1, 127.9, 124.4, 121.3, 119.9, 113.9, 55.3.

HRMS (ESI-TOF) m/z: $[M + H]^+$ calculated for $C_{18}H_{18}NO_2$ 280.1332; Found 280.1340.

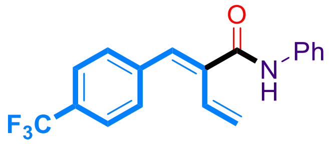

(*E*)-N-phenyl-2-(4-(trifluoromethyl)benzylidene)but-3-enamide (3f):

Yellow solid, 13.5 mg, 42% yield (*E:Z* = 2.5), R_f=0.2 (PE/EtOAc 10/1). NMR data for main isomer **3f** was provided.

^{1}**H NMR** (700 MHz, $CDCl_3$) δ 7.76 (s, 1H), 7.64 (d, J = 8.1 Hz, 2H), 7.61 (d, J = 7.9 Hz, 2H), 7.53 (d, J = 7.9 Hz, 2H), 7.42 (s, 1H), 7.37 (t, J = 7.9 Hz, 2H), 7.16 (t, J = 7.4 Hz, 1H), 6.75 (dd, J = 17.8, 11.3 Hz, 1H), 5.75 (d, J = 17.9 Hz, 1H), 5.68 (d, J = 11.4 Hz, 1H).

^{13}**C NMR** (176 MHz, $CDCl_3$) δ 165.3, 138.8, 137.6, 136.6, 134.1, 131.2, 130.3 (q, J = 32.7 Hz), 130.2, 129.2, 125.3 (q, J = 3.8 Hz), 124.8, 123.9 (q, J = 272.7 Hz), 122.9, 119.9.

HRMS (ESI-TOF) m/z: $[M + H]^+$ calculated for $C_{18}H_{15}F_3NO$ 318.1100; Found 318.1096.

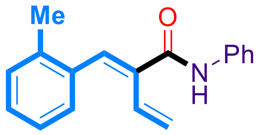

(*E*)-2-(2-methylbenzylidene)-N-phenylbut-3-enamide (3g):

Brown solid, 17.1 mg, 65% yield (*E:Z* = 2.7), R_f=0.2 (PE/EtOAc 10/1). NMR data for main isomer **3g** was provided.

^{1}**H NMR** (700 MHz, $CDCl_3$) δ 7.77 (s, 1H), 7.66 (d, J = 7.6 Hz, 2H), 7.46 (s, 1H), 7.39 (t, J = 7.8 Hz, 2H), 7.31 (d, J = 7.3 Hz, 1H), 7.26 (dd, J = 16.0, 7.3 Hz, 2H), 7.24 – 7.21 (m, 1H), 7.18 (t, J = 7.4 Hz, 1H), 6.67 (dd, J = 17.9, 11.4 Hz, 1H), 5.70 (d, J = 17.9 Hz, 1H), 5.55 (d, J = 11.3 Hz, 1H), 2.35 (s, 3H).

^{13}**C NMR** (176 MHz, $CDCl_3$) δ 166.1, 137.8, 137.4, 135.6, 134.9, 134.3, 131.7, 130.2, 130.0, 129.1, 128.6, 125.5, 124.5, 120.8, 119.8, 20.1.

HRMS (ESI-TOF) m/z: $[M + H]^+$ calculated for $C_{18}H_{18}NO$ 264.1383; Found 264.1377.

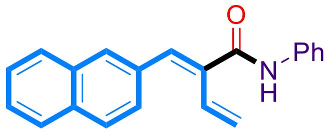

(*E*)-2-(naphthalen-2-ylmethylene)-N-phenylbut-3-enamide (3h):

Yellow solid, 15.8 mg, 53% yield (*E:Z* = 2.5), R_f=0.2 (PE/EtOAc 10/1). NMR data for main isomer **3h** was provided.

^{1}**H NMR** (700 MHz, $CDCl_3$) δ 7.93 (s, 1H), 7.89 – 7.85 (m, 4H), 7.66 (d, J = 7.8 Hz, 2H), 7.61 (s, 1H), 7.57 (d, J = 8.3 Hz, 1H), 7.54 (dt, J = 9.8, 5.6 Hz, 2H), 7.40 (t, J = 7.9 Hz, 2H), 7.18 (t, J = 7.4 Hz, 1H), 6.92 (dd, J = 17.9, 11.4 Hz, 1H), 5.78 (d, J = 17.9 Hz, 1H), 5.70 (d, J = 11.3 Hz, 1H).

^{13}**C NMR** (176 MHz, $CDCl_3$) δ 165.9, 137.9, 136.1, 134.8, 133.1, 133.1, 132.8, 132.1, 130.1, 129.1, 128.4, 128.0, 127.7, 127.3, 126.9, 126.5, 124.6, 121.9, 119.9.

HRMS (ESI-TOF) m/z: $[M + H]^+$ calculated for $C_{21}H_{18}NO$ 300.1383; Found 300.1376.

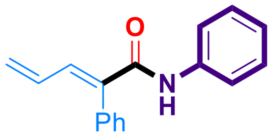

(*E*)-N,2-diphenylpenta-2,4-dienamide (4a):

Yellow solid, 17.9 mg, 72% yield (*E:Z* = 2.3), R_f=0.2 (PE/EtOAc 10/1). NMR data for main isomer **4a** was provided.

^{1}H NMR (700 MHz, $CDCl_3$) δ 7.55 (d, J = 11.4 Hz, 1H), 7.50 (t, J = 7.3 Hz, 2H), 7.46 (d, J = 7.3 Hz, 1H), 7.43 (d, J = 7.8 Hz, 2H), 7.33 – 7.31 (m, 2H), 7.28 (t, J = 7.9 Hz, 2H), 7.15 (s, 1H), 7.08 (t, J = 7.4 Hz, 1H), 6.24 (ddd, J = 16.9, 11.3, 10.2 Hz, 1H), 5.71 – 5.65 (m, 1H), 5.42 – 5.37 (m, 1H).

^{13}C NMR (176 MHz, $CDCl_3$) δ 164.6, 138.6, 137.8, 135.8, 134.8, 133.0, 130.2, 129.2, 128.9, 128.8, 125.3, 124.5, 119.9.

HRMS (ESI-TOF) m/z: $[M + H]^+$ calculated for $C_{17}H_{16}NO$ 250.1226; Found 250.1220.

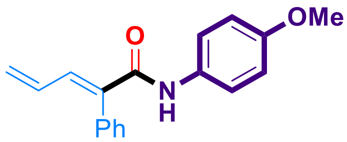

(*E*)-N-(4-methoxyphenyl)-2-phenylpenta-2,4-dienamide (4b):

Brown solid, 18.7 mg, 67% yield (*E:Z* = 1.8), R_f=0.2 (PE/EtOAc 5/1). NMR data for main isomer **4b** was provided.

^{1}H NMR (700 MHz, $CDCl_3$) δ 7.54 (d, J = 11.4 Hz, 1H), 7.49 (t, J = 7.3 Hz, 2H), 7.47 – 7.43 (m, 1H), 7.35 (d, J = 9.0 Hz, 2H), 7.34 – 7.29 (m, 2H), 7.06 (s, 1H), 6.85 – 6.80 (m, 2H), 6.23 (ddd, J = 16.9, 11.3, 10.1 Hz, 1H), 5.70 – 5.64 (m, 1H), 5.41 – 5.36 (m, 1H), 3.77 (s, 3H).

^{13}C NMR (176 MHz, $CDCl_3$) δ 164.5, 156.5, 138.3, 135.8, 135.0, 133.0, 131.0, 130.2, 129.2, 128.7, 125.1, 121.7, 114.1, 55.5.

HRMS (ESI-TOF) m/z: $[M + H]^+$ calculated for $C_{18}H_{18}NO_2$ 280.1330; Found 280.1326.

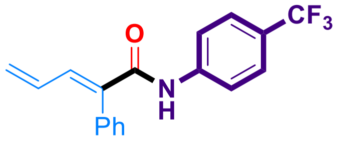

(*E*)-2-phenyl-N-(4-(trifluoromethyl)phenyl)penta-2,4-dienamide (4c):

Brown solid, 23.2 mg, 93% yield (*E:Z* = 5.6), R_f=0.2 (PE/EtOAc 10/1). NMR data for main isomer **4c** was provided.

^{1}H NMR (700 MHz, $CDCl_3$) δ 7.59 – 7.55 (m, 3H), 7.55 – 7.50 (m, 4H), 7.48 (dd, J = 8.7, 6.1 Hz, 1H), 7.34 – 7.31 (m, 2H), 7.28 (s, 1H), 6.24 (ddd, J = 16.9, 11.3, 10.1 Hz, 1H), 5.71 (d, J = 16.9 Hz, 1H), 5.44 (d, J = 10.1 Hz, 1H).

^{13}C NMR (176 MHz, $CDCl_3$) δ 164.8, 140.8, 139.4, 135.3, 134.5, 132.9, 130.2, 129.4, 129.0, 126.2 (q, J = 3.8 Hz), 126.0, 124.1 (q, J = 271.7 Hz), 119.4.

HRMS (ESI-TOF) m/z: $[M + H]^+$ calculated for $C_{18}H_{15}F_3NO$ 318.1100; Found 318.1105.

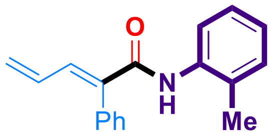

(*E*)-2-phenyl-N-(o-tolyl)penta-2,4-dienamide (4d):

Red solid, 18.8 mg, 72% yield (*E:Z* = 3.3), R_f=0.2 (PE/EtOAc 10/1). NMR data for main isomer **4d** was provided.

^{1}H NMR (400 MHz, $CDCl_3$) δ 8.10 (d, J = 8.1 Hz, 1H), 7.57 (d, J = 11.4 Hz, 1H), 7.53 – 7.42 (m, 3H), 7.38 – 7.33 (m, 2H), 7.21 (t, J = 7.7 Hz, 1H), 7.08 (d, J = 7.1 Hz, 2H), 7.03 – 6.99 (m, 1H), 6.24 (ddd, J = 16.9, 11.4, 10.1 Hz, 1H), 5.72 – 5.64 (m, 1H), 5.43 – 5.36 (m, 1H), 1.83 (s, 3H).

^{13}C NMR (100 MHz, $CDCl_3$) δ 164.4, 138.3, 136.0, 135.1, 133.0, 130.3, 130.2, 129.2, 128.8, 127.6, 126.9, 125.2, 124.7, 121.5, 17.1.

HRMS (ESI-TOF) m/z: $[M + H]^+$ calculated for $C_{18}H_{18}NO$ 264.1383; Found 264.1393.

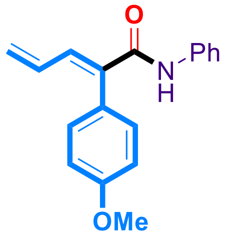

(*E*)-2-(4-methoxyphenyl)-N-phenylpenta-2,4-dienamide (4e):

White solid, 18.5 mg, 66% yield (*E:Z* = 1.7), R_f=0.2 (PE/EtOAc 10/1). NMR data for main isomer **4e** was provided.

^{1}H NMR (400 MHz, $CDCl_3$) δ 7.51 (d, J = 11.4 Hz, 1H), 7.45 (d, J = 7.7 Hz, 2H), 7.31 – 7.20 (m, 5H), 7.08 (t, J = 7.4 Hz, 1H), 7.01 (d, J = 8.7 Hz, 2H), 6.27 (ddd, J = 16.9, 11.2, 10.2 Hz, 1H), 5.72 – 5.60 (m, 1H), 5.42 – 5.34 (m, 1H), 3.88 (s, 3H).

^{13}C NMR (100 MHz, $CDCl_3$) δ 165.0, 159.9, 138.4, 137.9, 135.5, 133.2, 131.5, 128.9, 126.7, 124.9, 124.4, 119.8, 114.6, 55.4.

HRMS (ESI-TOF) m/z: $[M + H]^+$ calculated for $C_{18}H_{18}NO_2$ 280.1332; Found 280.1342.

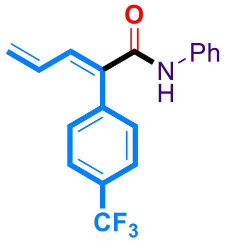

(*E*)-N-phenyl-2-(4-(trifluoromethyl)phenyl)penta-2,4-dienamide (4f):

White solid, 15.8 mg, 49% yield (*E:Z* = 1.3), R_f=0.2 (PE/EtOAc 10/1). NMR data for main isomer **4f** was provided.

^{1}H NMR (700 MHz, $CDCl_3$) δ 7.79 (d, J = 8.0 Hz, 2H), 7.59 (d, J = 11.5 Hz, 1H), 7.50 (d, J = 8.0 Hz, 2H), 7.47 (d, J = 7.7 Hz, 2H), 7.32 (t, J = 8.0 Hz, 2H), 7.13 (t, J = 7.4 Hz, 1H), 7.02 (s, 1H), 6.21 (ddd, J = 16.8, 11.4, 10.1 Hz, 1H), 5.75 (d, J = 16.8 Hz, 1H), 5.49 (d, J = 10.4 Hz, 1H).

^{13}C NMR (176 MHz, $CDCl_3$) δ 163.9, 139.4, 138.7, 137.5, 134.6, 132.3, 131.0 (q, J = 32.8 Hz), 130.7, 129.0, 126.6, 126.2 (q, J = 3.7 Hz), 124.8, 123.8 (q, J = 272.4 Hz), 120.0.

HRMS (ESI-TOF) m/z: $[M + H]^+$ calculated for $C_{18}H_{15}F_3NO$ 318.1100; Found 318.1111.

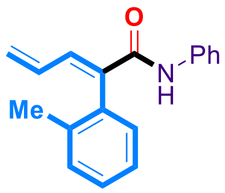

(*E*)-N-phenyl-2-(o-tolyl)penta-2,4-dienamide (4g):

White solid, 21.8 mg, 83% yield (*E:Z* = 2.2), R_f=0.2 (PE/EtOAc 10/1). NMR data for main isomer **4g** was provided.

¹H NMR (700 MHz, $CDCl_3$) δ 7.60 (d, J = 11.4 Hz, 1H), 7.41 (d, J = 8.3 Hz, 2H), 7.39 – 7.33 (m, 2H), 7.32 (t, J = 7.1 Hz, 1H), 7.27 (t, J = 7.8 Hz, 2H), 7.20 (d, J = 7.4 Hz, 1H), 7.08 (t, J = 7.5 Hz, 1H), 7.04 (s, 1H), 6.05 (dt, J = 16.9, 10.7 Hz, 1H), 5.67 (d, J = 16.9 Hz, 1H), 5.38 (d, J = 10.1 Hz, 1H), 2.22 (s, 3H).

¹³C NMR (176 MHz, $CDCl_3$) δ 164.3, 138.7, 137.8, 137.7, 135.2, 134.0, 133.0, 130.9, 130.5, 129.2, 128.9, 126.6, 125.3, 124.5, 119.9, 19.7.

HRMS (ESI-TOF) m/z: $[M + H]^+$ calculated for $C_{18}H_{18}NO$ 264.1383; Found 264.1390.

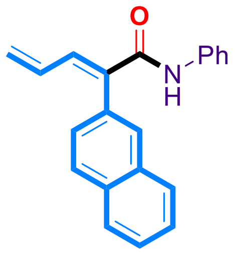

(*E*)-2-(naphthalen-2-yl)-N-phenylpenta-2,4-dienamide (4h):

Brown solid, 21.3 mg, 68% yield (*E:Z* = 1.6), R_f=0.2 (PE/EtOAc 10/1). NMR data for main isomer **4h** was provided.

¹H NMR (400 MHz, $CDCl_3$) δ 7.97 (d, J = 8.4 Hz, 1H), 7.95 – 7.86 (m, 2H), 7.81 (s, 1H), 7.63 (d, J = 11.4 Hz, 1H), 7.61 – 7.55 (m, 2H), 7.45 – 7.38 (m, 3H), 7.28 – 7.24 (m, 2H), 7.20 (s, 1H), 7.07 (t, J = 7.4 Hz, 1H), 6.27 (ddd, J = 16.9, 11.4, 10.1 Hz, 1H), 5.76 – 5.62 (m, 1H), 5.44 – 5.35 (m, 1H).

¹³C NMR (100 MHz, $CDCl_3$) δ 164.6, 138.9, 137.8, 135.8, 133.3, 133.1, 132.2, 129.7, 129.1, 128.9, 128.1, 127.9, 127.5, 127.0, 127.0, 125.5, 124.5, 119.9.

HRMS (ESI-TOF) m/z: $[M + H]^+$ calculated for $C_{21}H_{18}NO$ 300.1383; Found 300.1380.

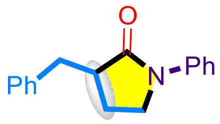

3-Benzyl-1-phenylpyrrolidin-2-one (5a1):

White solid, 24.0 mg, 96% yield, R_f=0.2 (PE/EtOAc 10/1).

¹H NMR (700 MHz, $CDCl_3$) δ 7.61 (d, J = 8.1 Hz, 2H), 7.36 (t, J = 8.0 Hz, 2H), 7.30 (t, J = 7.6 Hz, 2H), 7.25 – 7.21 (m, 3H), 7.14 (t, J = 7.4 Hz, 1H), 3.70 (dt, J = 9.6, 7.7 Hz, 1H), 3.60 (td, J = 8.9, 3.3 Hz, 1H), 3.31 (dd, J = 13.8, 4.2 Hz, 1H), 2.91 (td, J = 9.0, 4.2 Hz, 1H), 2.79 (dd, J = 13.8, 9.4 Hz, 1H), 2.16 (dtd, J = 12.3, 7.7, 3.4 Hz, 1H), 1.85 (dq, J = 12.7, 8.6 Hz, 1H).

¹³C NMR (176 MHz, $CDCl_3$) δ 175.1, 139.5, 139.3, 129.1, 128.8, 128.5, 126.4, 124.5, 119.9, 46.7, 45.2, 37.0, 24.2.

HRMS (ESI-TOF) m/z: $[M + H]^+$ calculated for $C_{17}H_{18}NO$ 252.1383; Found 252.1390.

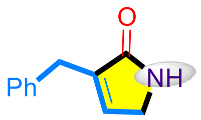

3-Benzyl-1,5-dihydro-2H-pyrrol-2-one (5d1):

Yellow solid, 13.5 mg, 78% yield, R_f=0.2 (PE/EtOAc 10/1).

^{1}H NMR (700 MHz, $CDCl_3$) δ 7.31 (t, J = 7.6 Hz, 2H), 7.26 – 7.23 (m, 3H), 7.08 (s, 1H), 6.54 (d, J = 1.8 Hz, 1H), 3.93 – 3.87 (m, 2H), 3.60 (d, J = 2.2 Hz, 2H).

^{13}C NMR (176 MHz, $CDCl_3$) δ 174.7, 139.6, 139.0, 138.5, 129.0, 128.6, 126.4, 46.6, 31.9.

HRMS (ESI-TOF) m/z: $[M + H]^+$ calculated for $C_{11}H_{12}NO$ 174.0913; Found 174.0915.

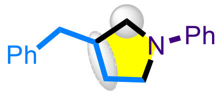

3-Benzyl-1-phenylpyrrolidine (5a2):

Brown oil, 16.7 mg, 70% yield, R_f=0.2 (PE/EtOAc 100/1).

^{1}H NMR (400 MHz, $CDCl_3$) δ 7.31 (dd, J = 8.1, 6.4 Hz, 2H), 7.21 (tt, J = 7.1, 2.8 Hz, 5H), 6.65 (t, J = 7.3 Hz, 1H), 6.53 (d, J = 7.9 Hz, 2H), 3.44 – 3.33 (m, 2H), 3.28 (dt, J = 9.2, 7.5 Hz, 1H), 3.01 (dd, J = 9.3, 7.2 Hz, 1H), 2.80 – 2.70 (m, 2H), 2.60 (dq, J = 14.8, 7.2 Hz, 1H), 2.09 (dtd, J = 13.6, 6.8, 3.8 Hz, 1H), 1.74 (dq, J = 12.2, 8.2 Hz, 1H).

^{13}C NMR (100 MHz, $CDCl_3$) δ 147.9, 140.8, 129.1, 128.8, 128.5, 126.1, 115.4, 111.5, 53.0, 47.3, 40.5, 39.9, 31.4.

HRMS (ESI-TOF) m/z: $[M + H]^+$ calculated for $C_{17}H_{20}N$ 238.1590; Found 238.1600.

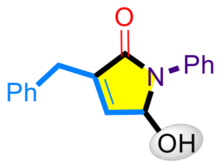

3-Benzyl-3-hydroxy-1-phenyl-1,3-dihydro-2H-pyrrol-2-one (5a3):

White solid, 22.1 mg, 83% yield, R_f=0.2 (PE/EtOAc 10/1).

^{1}H NMR (700 MHz, $CDCl_3$) δ 7.70 (d, J = 7.8 Hz, 2H), 7.38 (t, J = 7.9 Hz, 2H), 7.31 (t, J = 7.5 Hz, 2H), 7.25 (d, J = 6.1 Hz, 1H), 7.22 (d, J = 7.3 Hz, 2H), 7.17 (t, J = 7.4 Hz, 1H), 6.48 – 6.38 (m, 1H), 5.85 (s, 1H), 3.60 (d, J = 16.7 Hz, 1H), 3.56 (d, J = 16.7 Hz, 1H), 2.70 (s, 1H).

^{13}C NMR (176 MHz, $CDCl_3$) δ 168.5, 141.8, 137.9, 137.4, 137.0, 129.1, 129.1, 128.7, 126.7, 124.9, 120.6, 82.2, 31.9.

HRMS (ESI-TOF) m/z: $[M + H]^+$ calculated for $C_{17}H_{16}NO_2$ 266.1176; Found 266.1176.

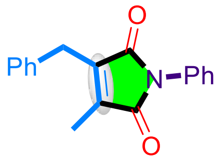

3-Benzyl-4-methyl-1-phenyl-1H-pyrrole-2,5-dione (6a'):

White solid, 20.4 mg, 74% yield, R_f=0.2 (PE/EtOAc 5/1).

^{1}H NMR (400 MHz, $CDCl_3$) δ 7.46 – 7.40 (m, 2H), 7.35 (d, J = 1.7 Hz, 1H), 7.35 – 7.29 (m, 5H), 7.29 – 7.26 (m, 1H), 7.26 – 7.22 (m, 1H), 3.81 (s, 2H), 2.06 (s, 3H).

^{13}C NMR (100 MHz, $CDCl_3$) δ 170.9, 170.6, 139.4, 137.9, 136.8, 131.9, 129.0, 128.9, 127.5, 127.0, 125.7, 29.8, 9.2.

HRMS (ESI-TOF) m/z: $[M + H]^+$ calculated for $C_{18}H_{16}NO_2$ 278.1176; Found 278.1184.

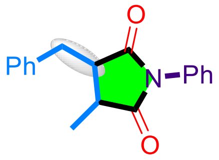

3-Benzyl-4-methyl-1-phenylpyrrolidine-2,5-dione (6a1):

White solid, 23.3 mg, 84% yield, R_f=0.2 (PE/EtOAc 10/1).

^{1}H NMR (400 MHz, $CDCl_3$) δ 7.51 – 7.43 (m, 2H), 7.41 – 7.36 (m, 1H), 7.36 – 7.31 (m, 2H), 7.28 (ddq, J = 5.1, 3.4, 2.3, 1.8 Hz, 4H), 7.26 – 7.20 (m, 1H), 3.50 (ddd, J = 10.1, 8.6, 4.5 Hz, 1H), 3.44 (dd, J = 15.1, 4.6 Hz, 1H), 3.18 – 3.08 (m, 1H), 2.93 (dd, J = 15.0, 10.3 Hz, 1H), 1.34 (d, J = 7.6 Hz, 3H).

^{13}C NMR (100 MHz, $CDCl_3$) δ 179.3, 177.9, 138.4, 131.9, 129.2, 128.8, 128.6, 128.4, 126.7, 126.4, 44.7, 38.6, 31.7, 13.0.

HRMS (ESI-TOF) m/z: $[M + H]^+$ calculated for $C_{18}H_{18}NO_2$ 280.1332; Found 280.1337.

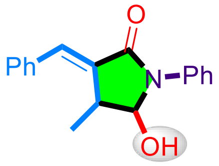

3-((*E*)-Benzylidene)-5-hydroxy-4-methyl-1-phenylpyrrolidin-2-one (6a3):

White solid, 19.8 mg, 71% yield, R_f=0.2 (PE/EtOAc 10/1).

^{1}H NMR (700 MHz, $CDCl_3$) δ 7.58 (d, J = 8.0 Hz, 2H), 7.44 (d, J = 2.6 Hz, 1H), 7.42 (d, J = 7.3 Hz, 2H), 7.38 (q, J = 8.0 Hz, 4H), 7.34 (t, J = 7.2 Hz, 1H), 7.22 (t, J = 7.4 Hz, 1H), 5.73 (dd, J = 8.9, 6.5 Hz, 1H), 3.68 (pd, J = 7.1, 2.6 Hz, 1H), 3.28 (d, J = 8.9 Hz, 1H), 1.24 (d, J = 7.2 Hz, 3H).

^{13}C NMR (176 MHz, $CDCl_3$) δ 167.5, 137.1, 134.9, 134.8, 132.7, 129.7, 129.0, 128.8, 128.6, 126.1, 123.3, 83.2, 35.8, 11.7.

HRMS (ESI-TOF) m/z: $[M + H]^+$ calculated for $C_{18}H_{18}NO_2$ 280.1332; Found 280.1340.

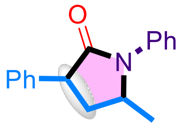

5-Methyl-1,3-diphenylpyrrolidin-2-one (7a1):

White solid, 16.3 mg, 65% yield, R_f=0.2 (PE/EtOAc 10/1).

^{1}H NMR (400 MHz, $CDCl_3$) δ 7.45 – 7.31 (m, 8H), 7.31 – 7.26 (m, 1H), 7.23 (t, J = 7.2 Hz, 1H), 4.36 – 4.23 (m, 1H), 3.84 (dd, J = 11.0, 9.1 Hz, 1H), 2.83 (ddd, J = 12.8, 9.0, 6.5 Hz, 1H), 1.88 (ddd, J = 12.7, 11.1, 8.7 Hz, 1H), 1.26 (d, J = 6.1 Hz, 3H).

^{13}C NMR (100 MHz, $CDCl_3$) δ 174.2, 139.4, 137.5, 128.9, 128.7, 128.2, 127.1, 126.0, 124.7, 53.0, 48.6, 37.1, 20.7.

HRMS (ESI-TOF) m/z: $[M + H]^+$ calculated for $C_{17}H_{18}NO$ 252.1383; Found 252.1390.

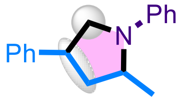

2-Methyl-1,4-diphenylpyrrolidine (7a2):

Red solid, 14.5 mg, 61% yield, R_f=0.2 (PE/EtOAc 100/1).

^{1}H NMR (700 MHz, $CDCl_3$) δ 7.41 – 7.28 (m, 5H), 7.25 – 7.23 (m, 2H), 6.69 (t, J = 7.2 Hz, 1H),

6.65 (d, J = 8.1 Hz, 2H), 3.99 (dq, J = 12.4, 6.1 Hz, 1H), 3.74 (t, J = 8.6 Hz, 1H), 3.57 (t, J = 9.5 Hz, 1H), 3.38 – 3.31 (m, 1H), 2.65 (dt, J = 12.9, 6.8 Hz, 1H), 1.91 (td, J = 11.7, 8.2 Hz, 1H), 1.31 (d, J = 5.9 Hz, 3H).

^{13}C NMR (176 MHz, $CDCl_3$) δ 147.5, 142.0, 129.0, 128.5, 127.3, 126.6, 115.9, 113.1, 56.8, 53.8, 43.0, 42.6, 20.1.

HRMS (ESI-TOF) m/z: $[M + H]^+$ calculated for $C_{17}H_{20}N$ 238.1590; Found 238.1596.

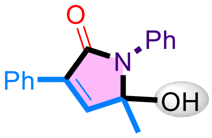

5-hydroxy-5-methyl-1,3-diphenyl-1,5-dihydro-2H-pyrrol-2-one (7a3):

Brown oil, 15.5 mg, 58% yield, R_f=0.2 (PE/EtOAc 5/1).

^{1}H NMR (400 MHz, $CDCl_3$) δ 7.89 – 7.80 (m, 2H), 7.51 – 7.47 (m, 2H), 7.40 – 7.35 (m, 5H), 7.31 – 7.26 (m, 1H), 7.04 (s, 1H), 3.07 (s, 1H), 1.51 (s, 3H).

^{13}C NMR (100 MHz, $CDCl_3$) δ 167.8, 142.3, 135.6, 135.0, 130.4, 129.2, 129.0, 128.5, 127.6, 126.9, 126.5, 88.9, 23.1.

HRMS (ESI-TOF) m/z: $[M + H]^+$ calculated for $C_{17}H_{16}NO_2$ 266.1176; Found 266.1180.

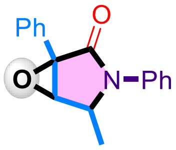

4-Methyl-1,3-diphenyl-6-oxa-3-azabicyclo[3.1.0]hexan-2-one (7a4):

Light yellow oil, 13.9 mg, 52% yield, R_f=0.2 (PE/EtOAc 10/1).

^{1}H NMR (400 MHz, $CDCl_3$) δ 7.61 (dt, J = 8.4, 2.1 Hz, 2H), 7.50 (dd, J = 8.6, 1.1 Hz, 2H), 7.46 – 7.42 (m, 2H), 7.40 (dd, J = 8.6, 1.8 Hz, 3H), 7.23 (d, J = 7.4 Hz, 1H), 4.44 (q, J = 6.7 Hz, 1H), 3.92 (s, 1H), 1.34 (d, J = 6.7 Hz, 3H).

^{13}C NMR (100 MHz, $CDCl_3$) δ 168.4, 137.2, 130.5, 129.2, 128.9, 128.5, 127.2, 126.1, 123.3, 64.3, 61.9, 55.0, 17.0.

HRMS (ESI-TOF) m/z: $[M + H]^+$ calculated for $C_{17}H_{16}NO_2$ 266.1176; Found 266.1180.

7. NMR Spectra of the Products

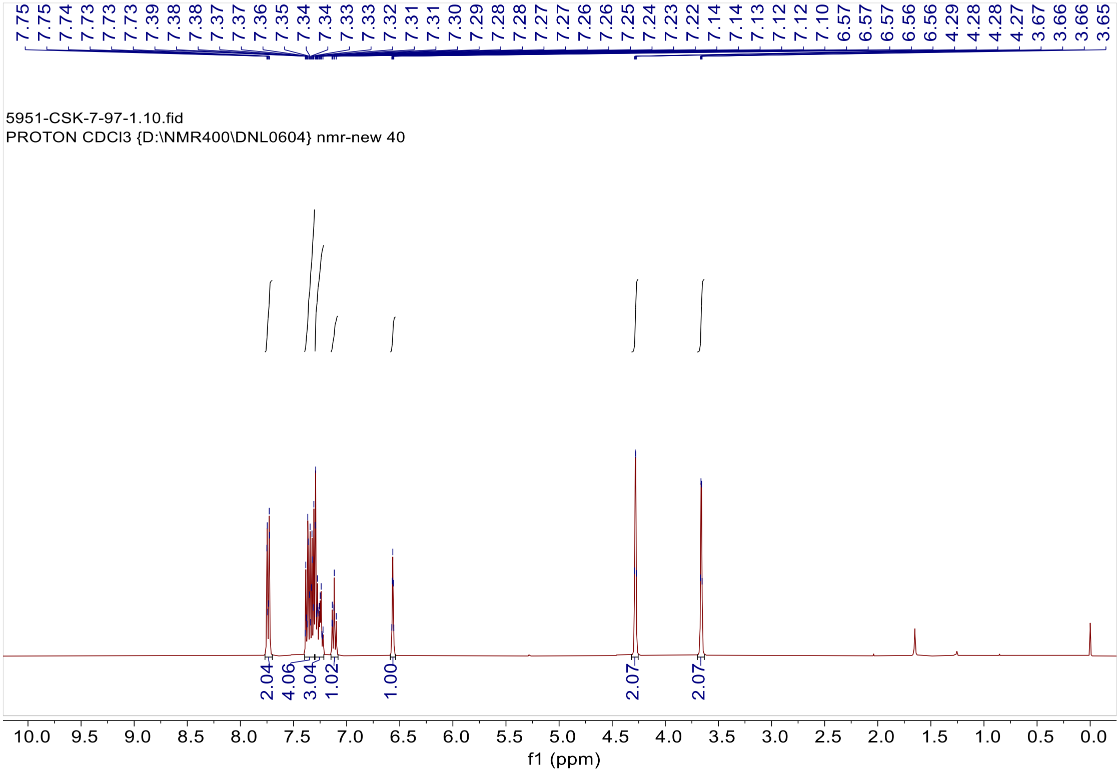

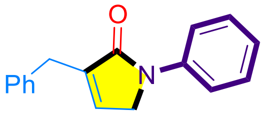

5a

^{1}H NMR (400 MHz, $CDCl_3$)
^{13}C NMR (100 MHz, $CDCl_3$)

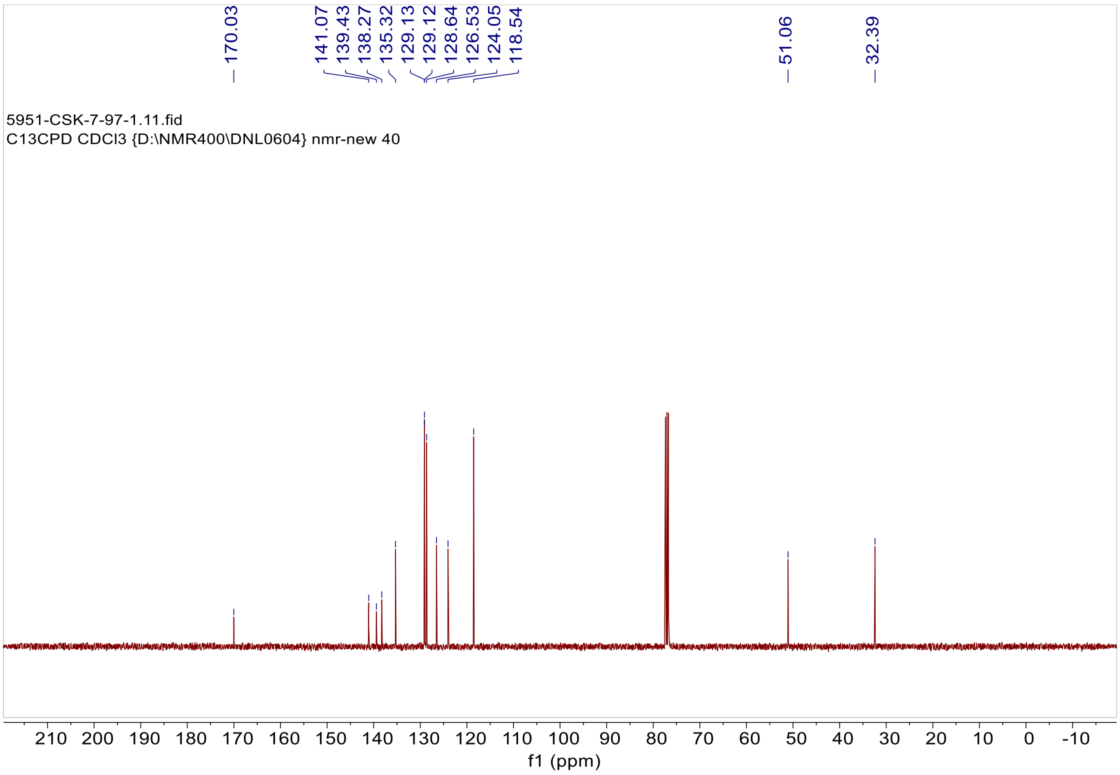

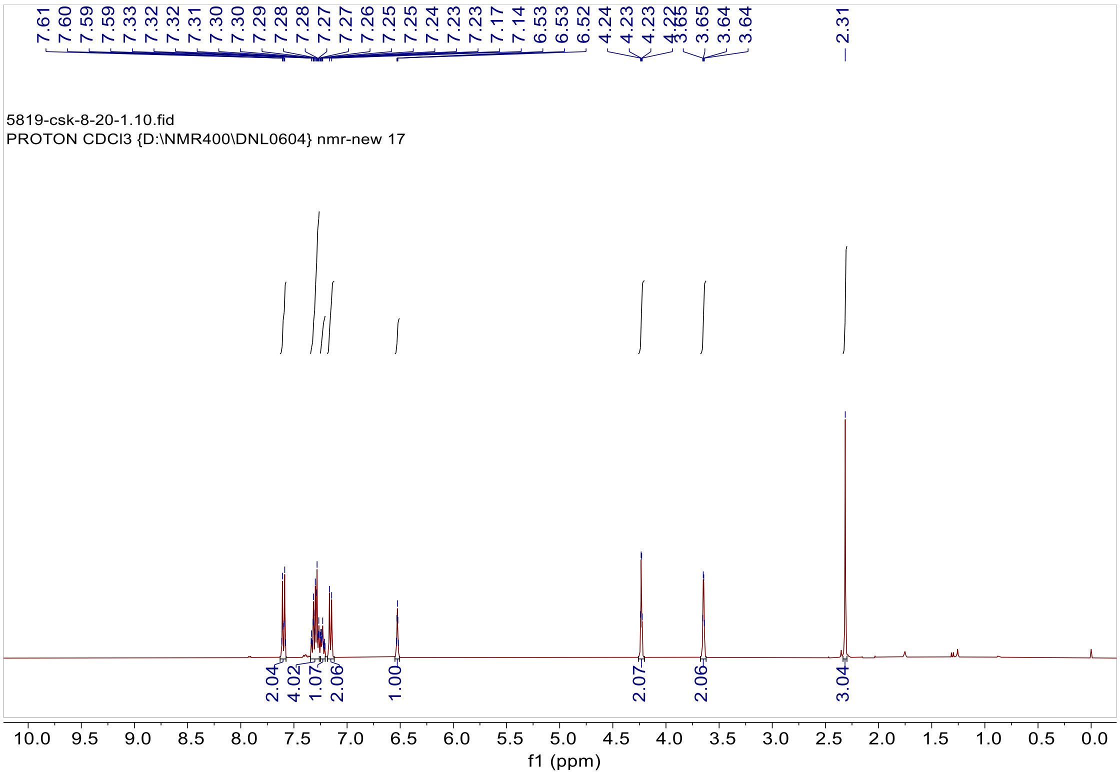

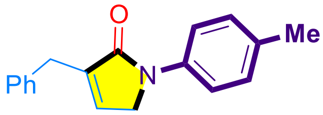

5b

^{1}H NMR (400 MHz, $CDCl_3$)

^{13}C NMR (100 MHz, $CDCl_3$)

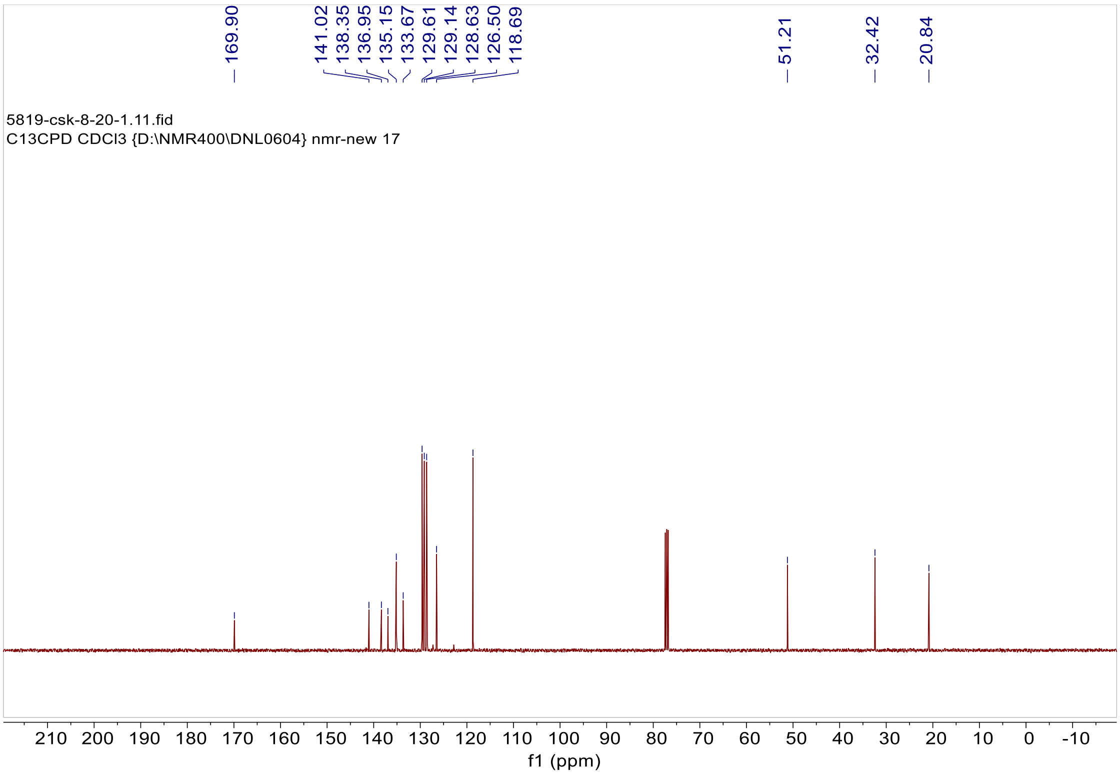

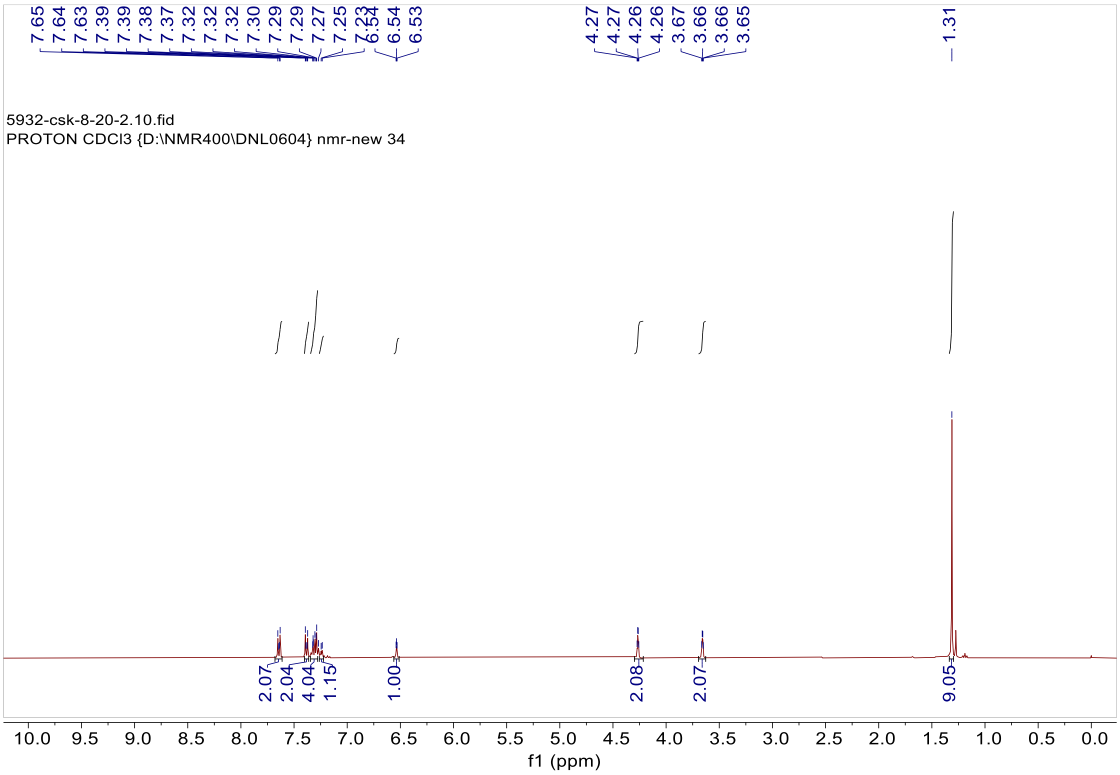

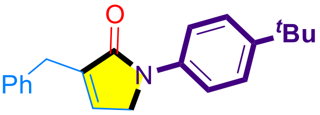

5c

^{1}H NMR (400 MHz, $CDCl_3$)

^{13}C NMR (100 MHz, $CDCl_3$)

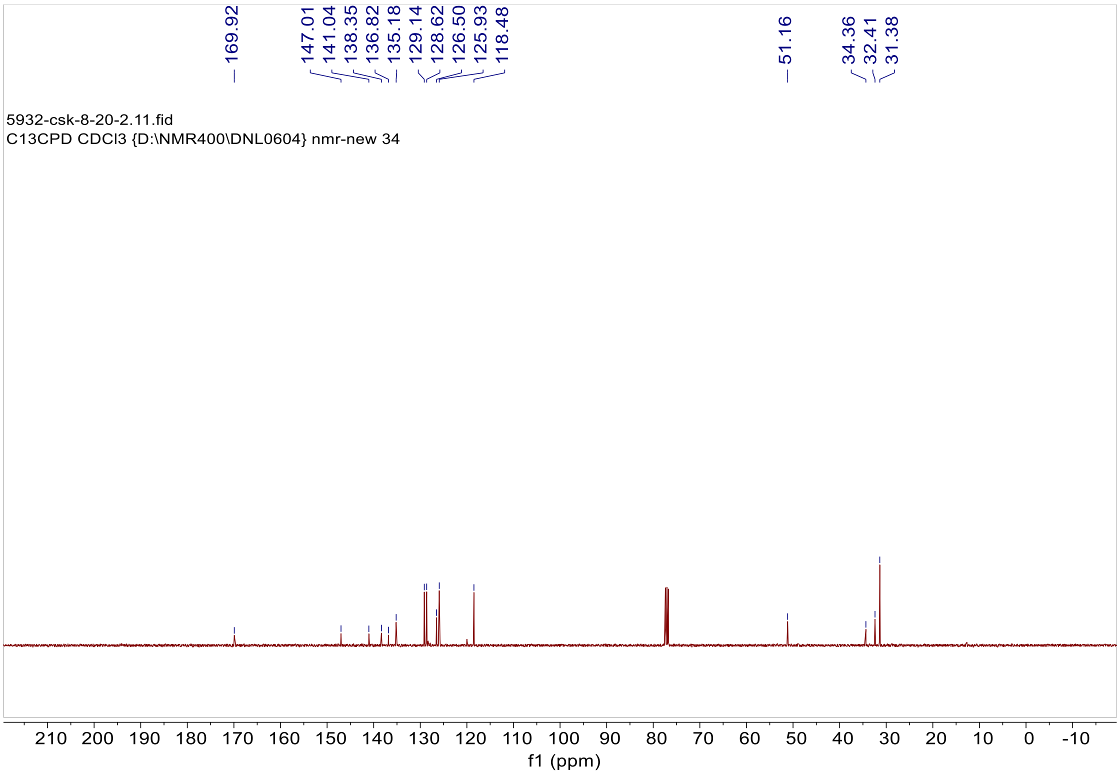

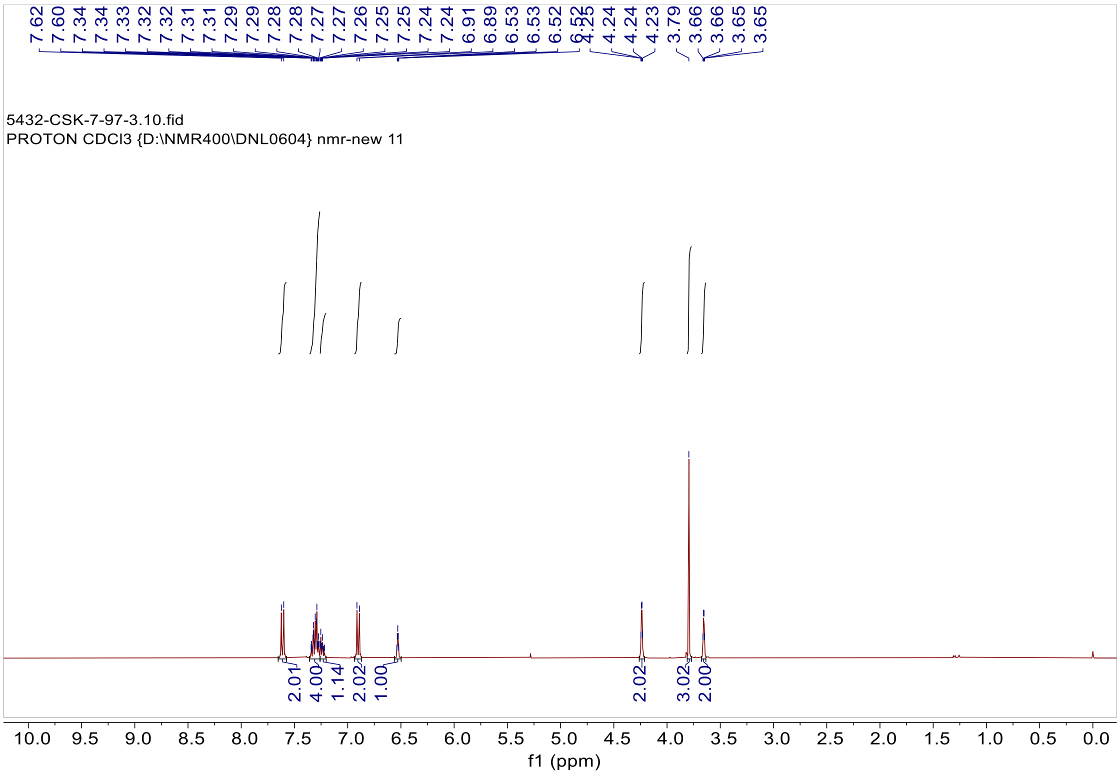

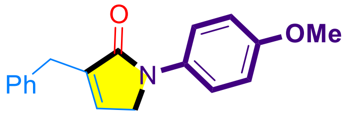

5d

^{1}H NMR (400 MHz, $CDCl_3$)

^{13}C NMR (100 MHz, $CDCl_3$)

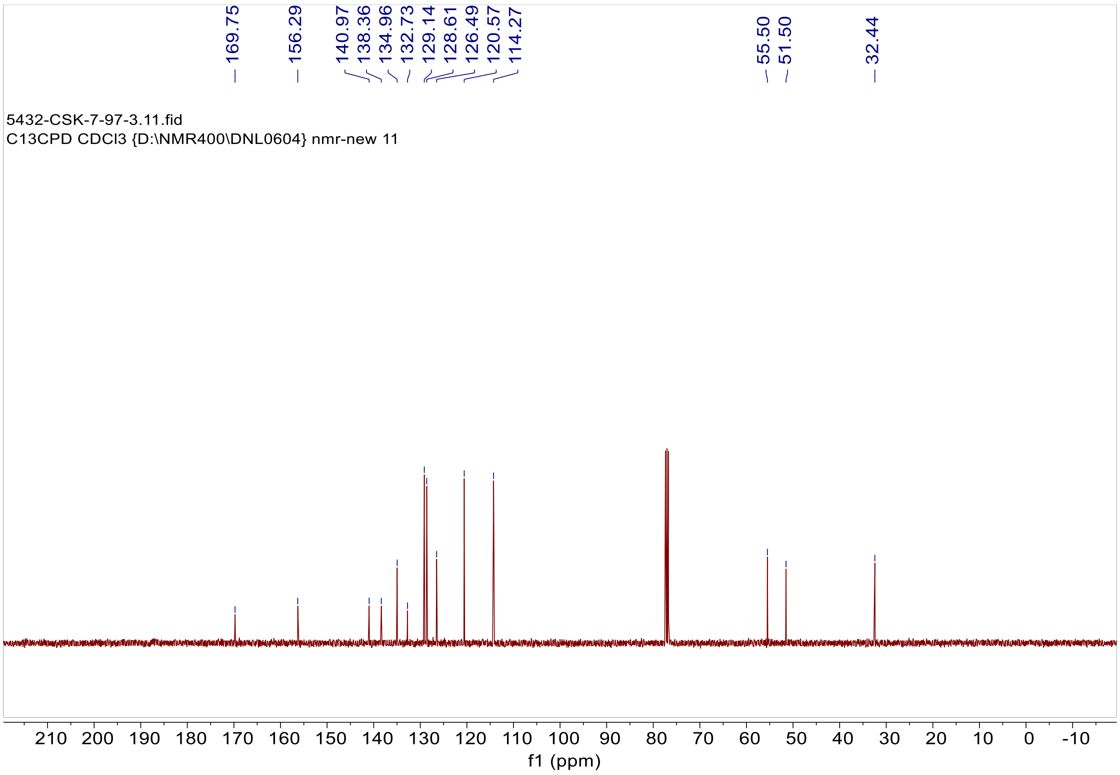

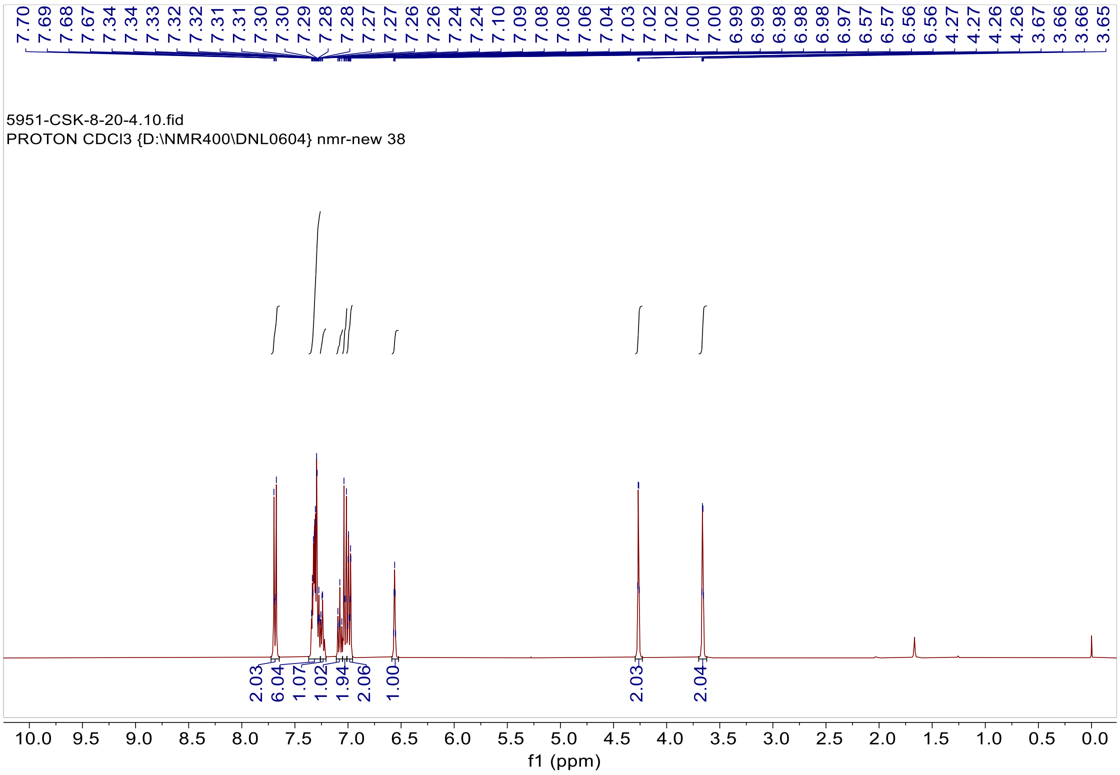

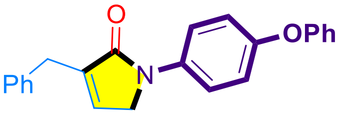

5e

^{1}H NMR (400 MHz, $CDCl_3$)
^{13}C NMR (100 MHz, $CDCl_3$)

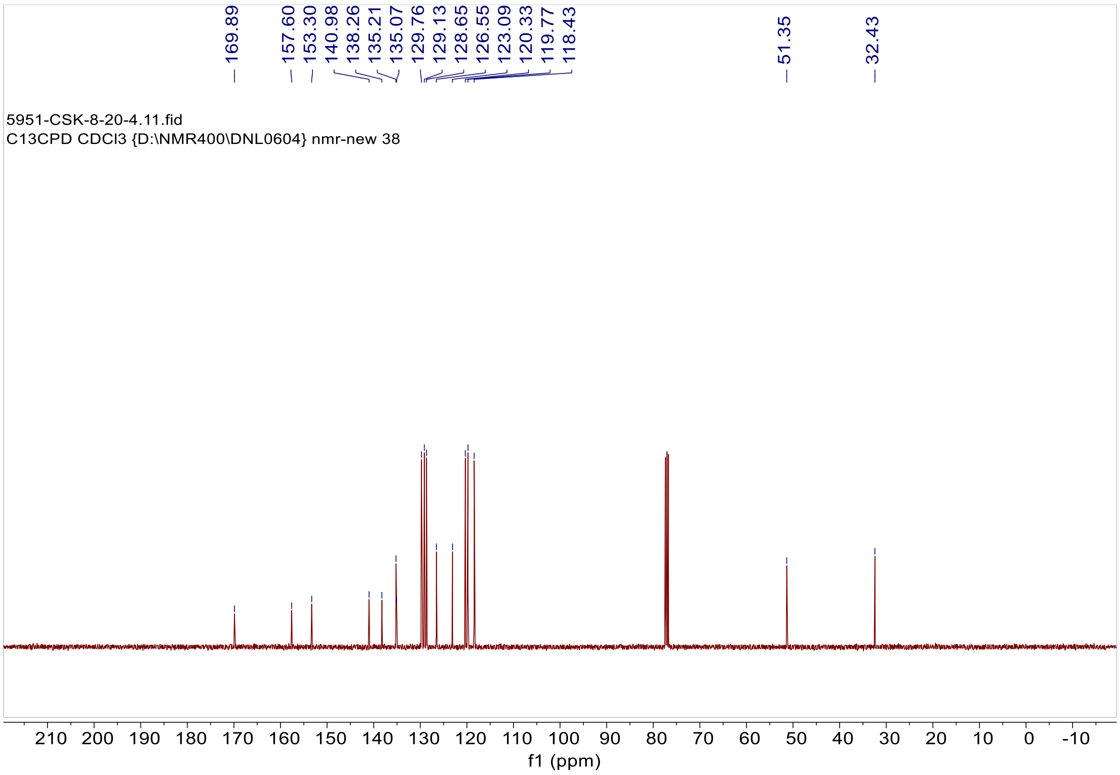

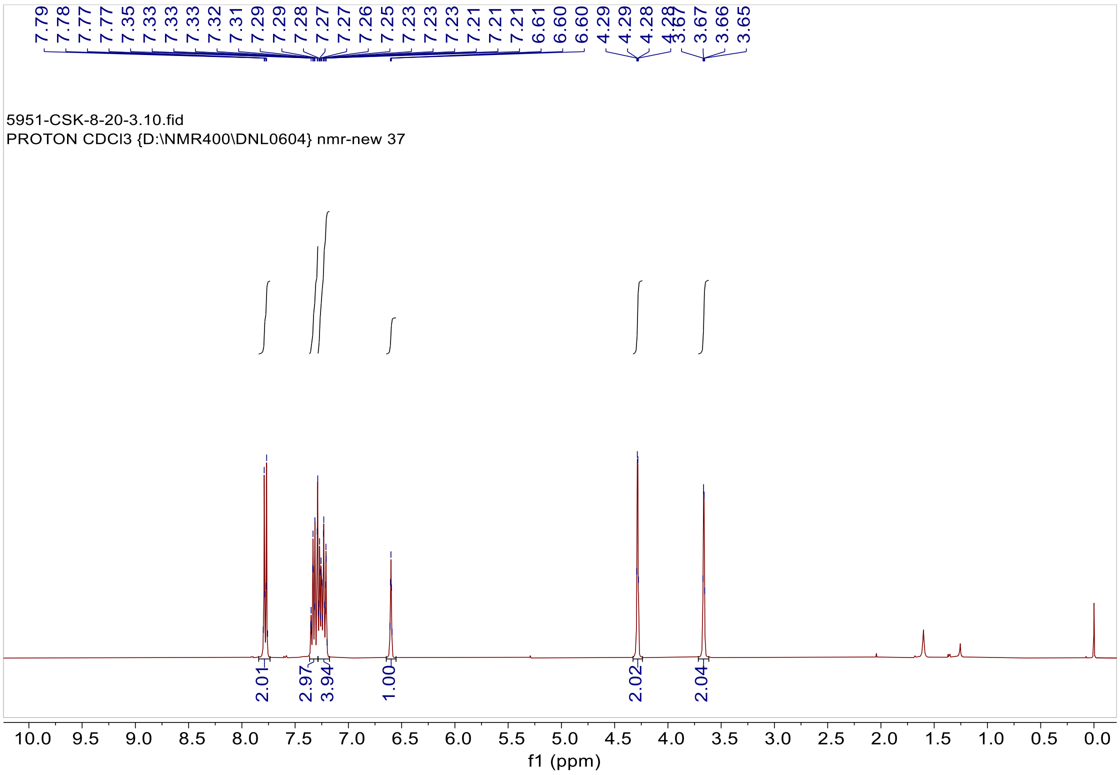

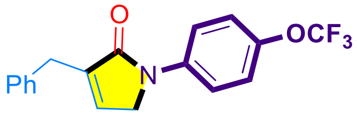

5f

^{1}H NMR (400 MHz, $CDCl_3$)
^{13}C NMR (100 MHz, $CDCl_3$)

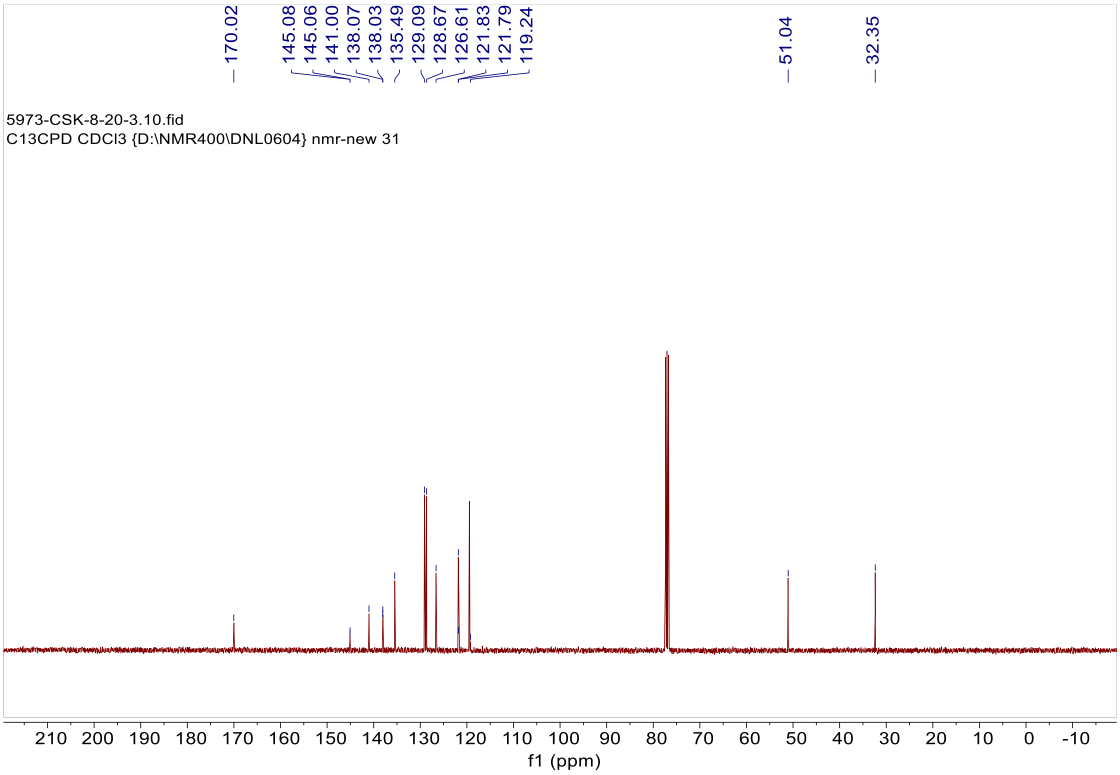

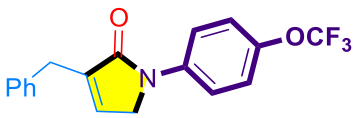

5f ^{19}F NMR (376 MHz, $CDCl_3$)

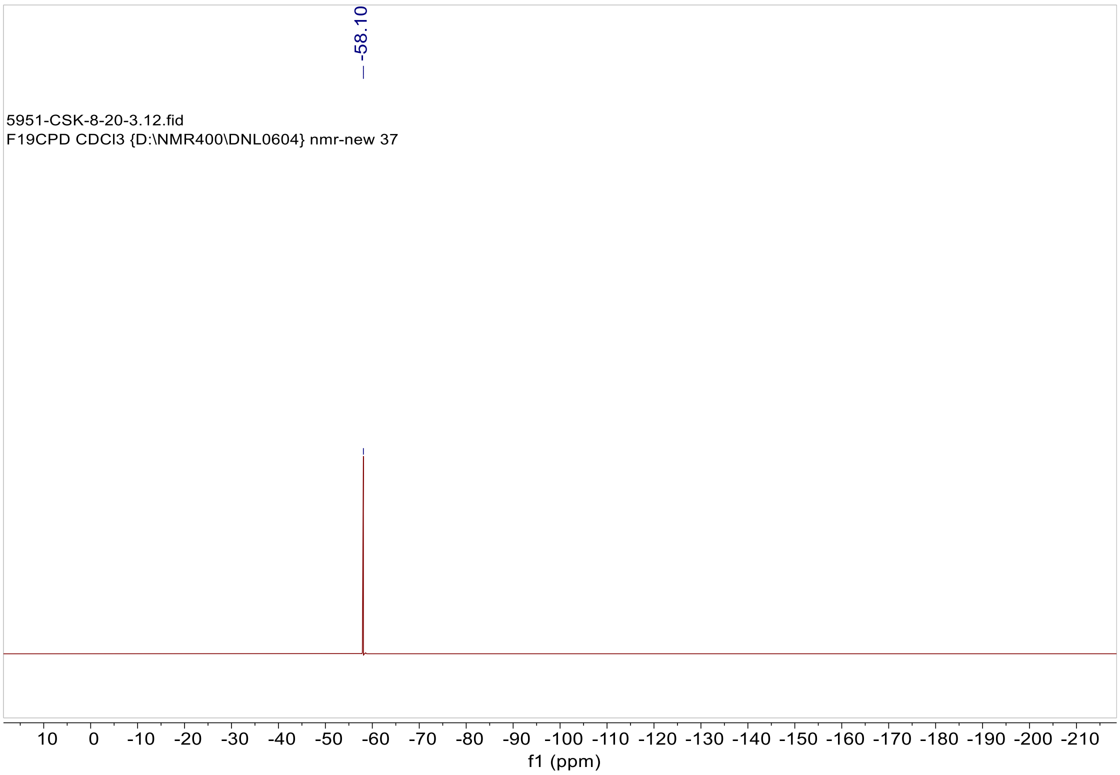

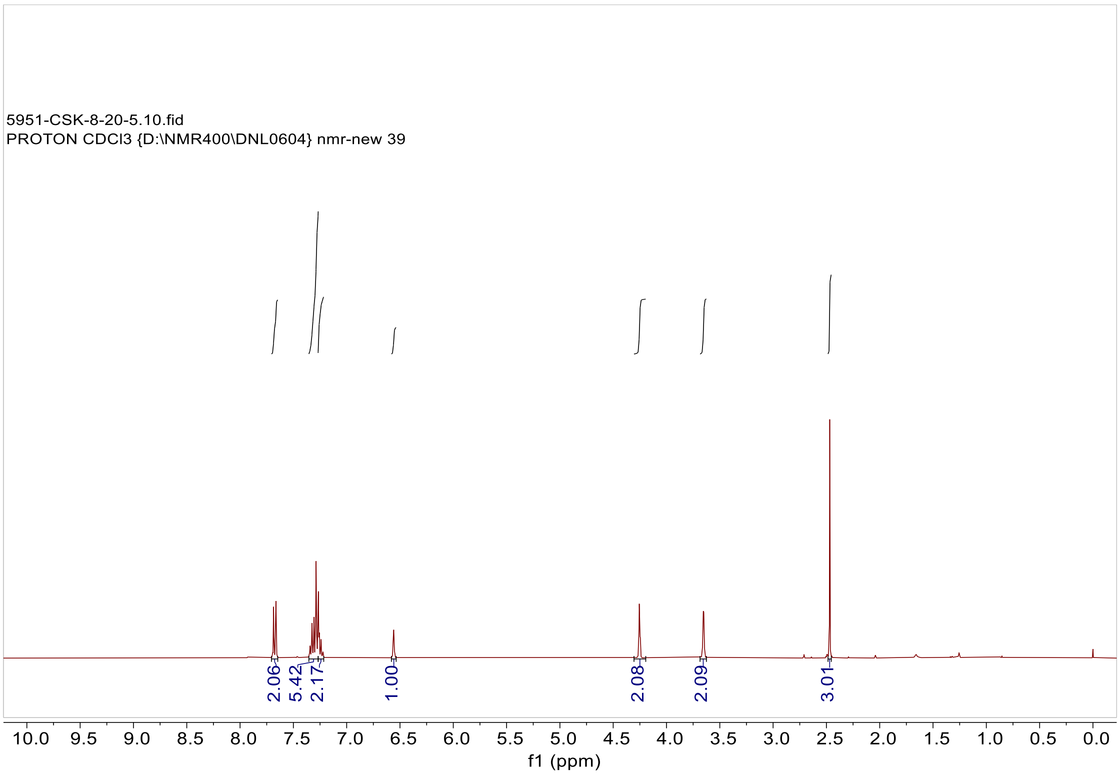

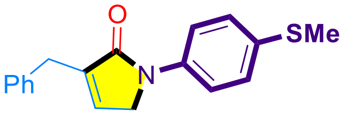

5g

^{1}H NMR (400 MHz, $CDCl_3$)
^{13}C NMR (100 MHz, $CDCl_3$)

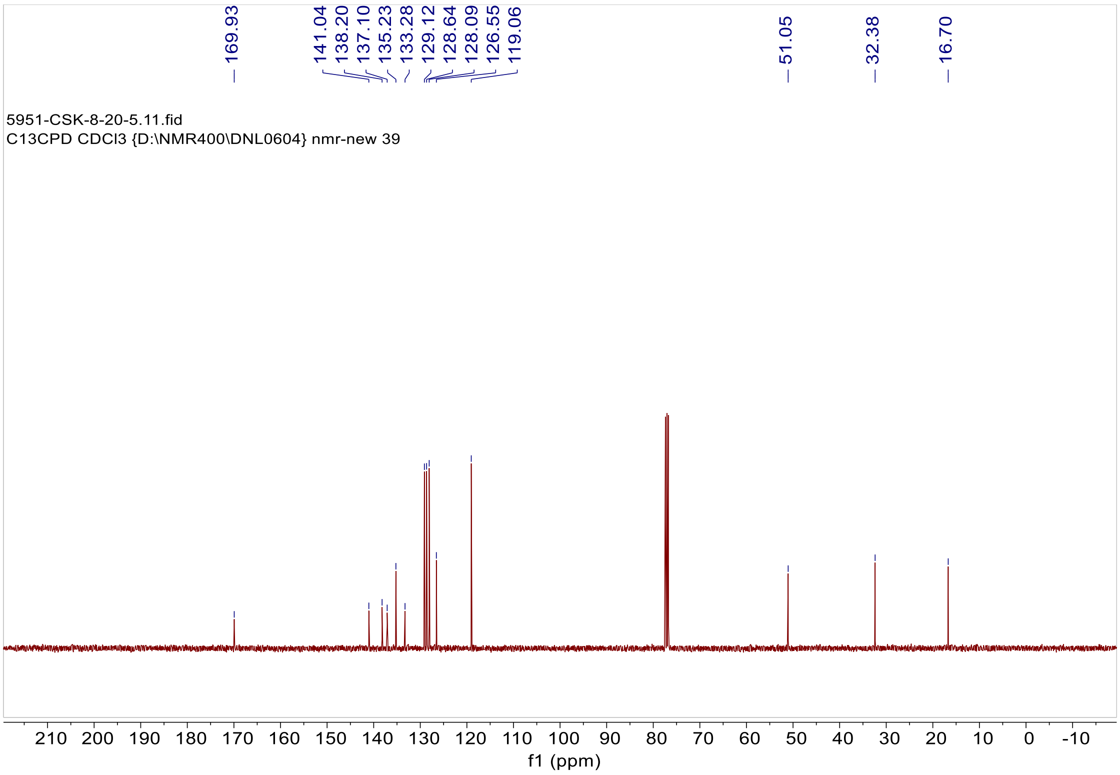

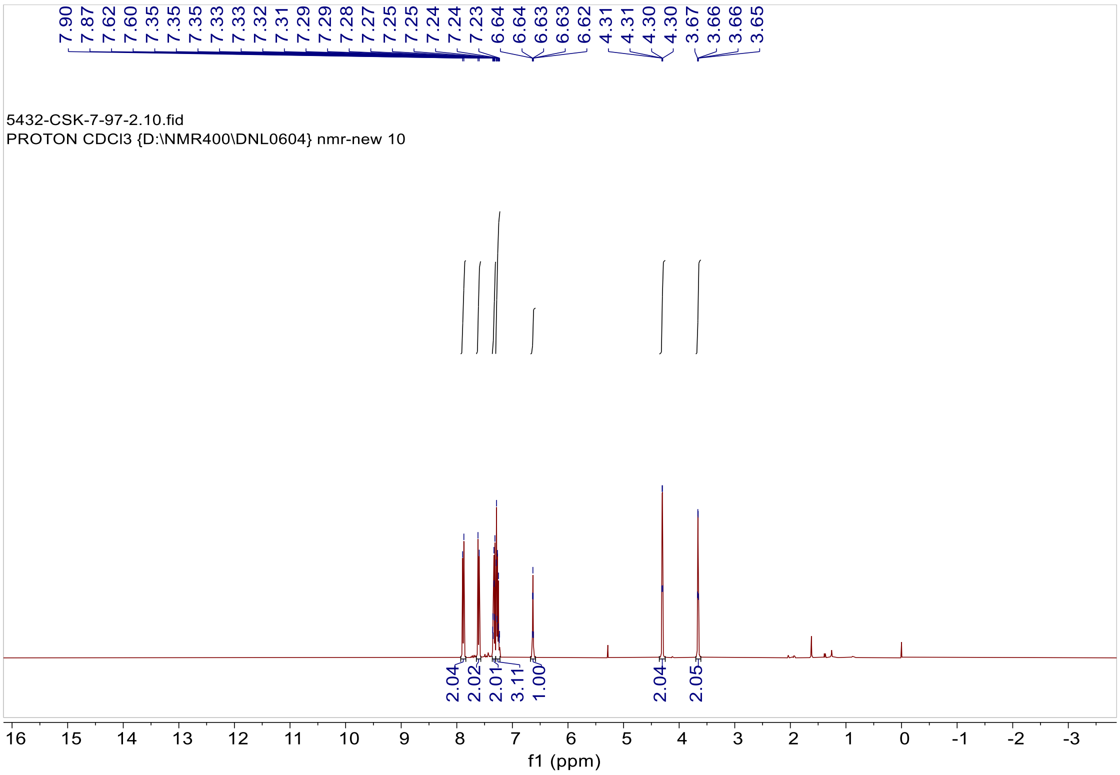

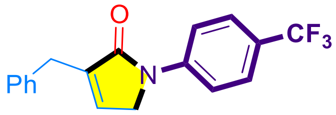

5h

^{1}H NMR (400 MHz, $CDCl_3$)

^{13}C NMR (100 MHz, $CDCl_3$)

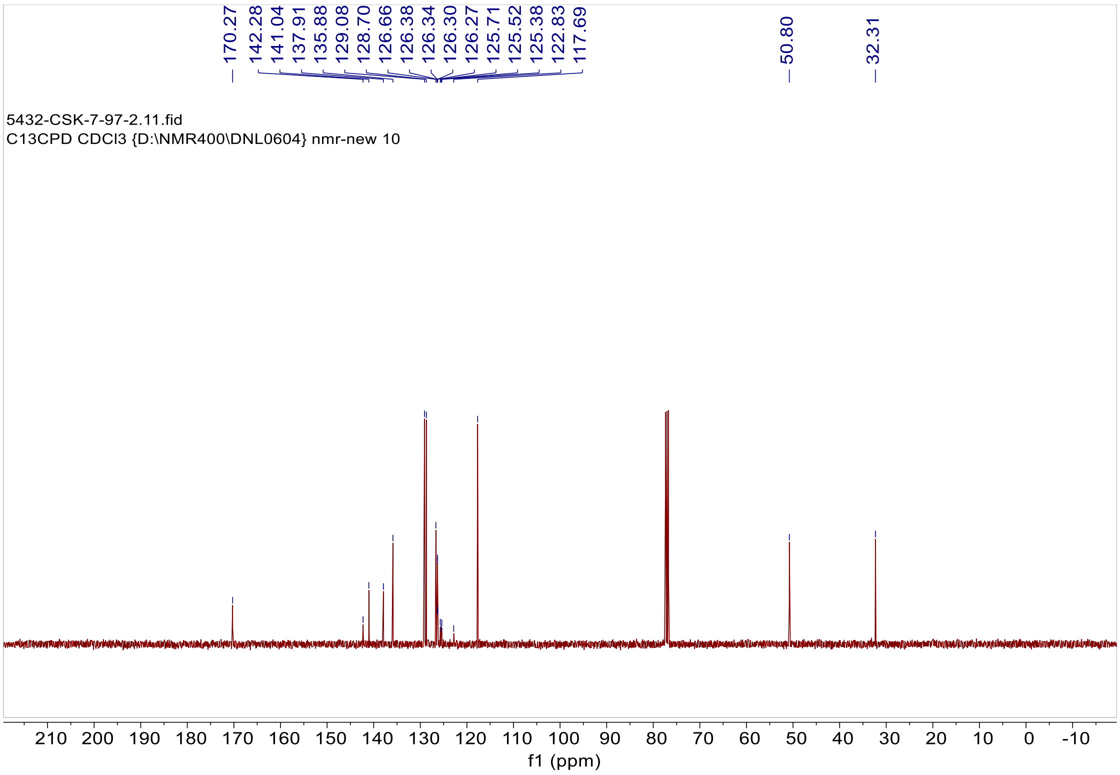

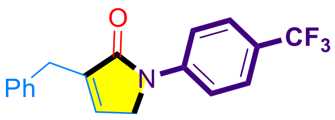

5h ^{19}F NMR (376 MHz, $CDCl_3$)

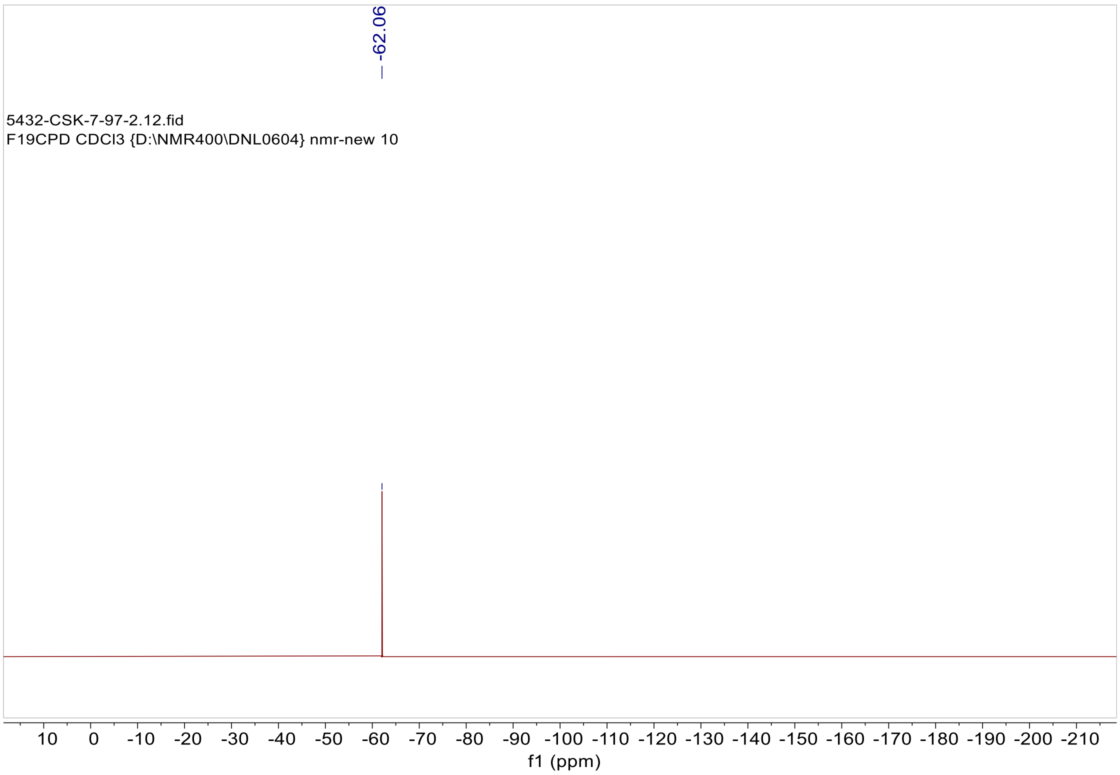

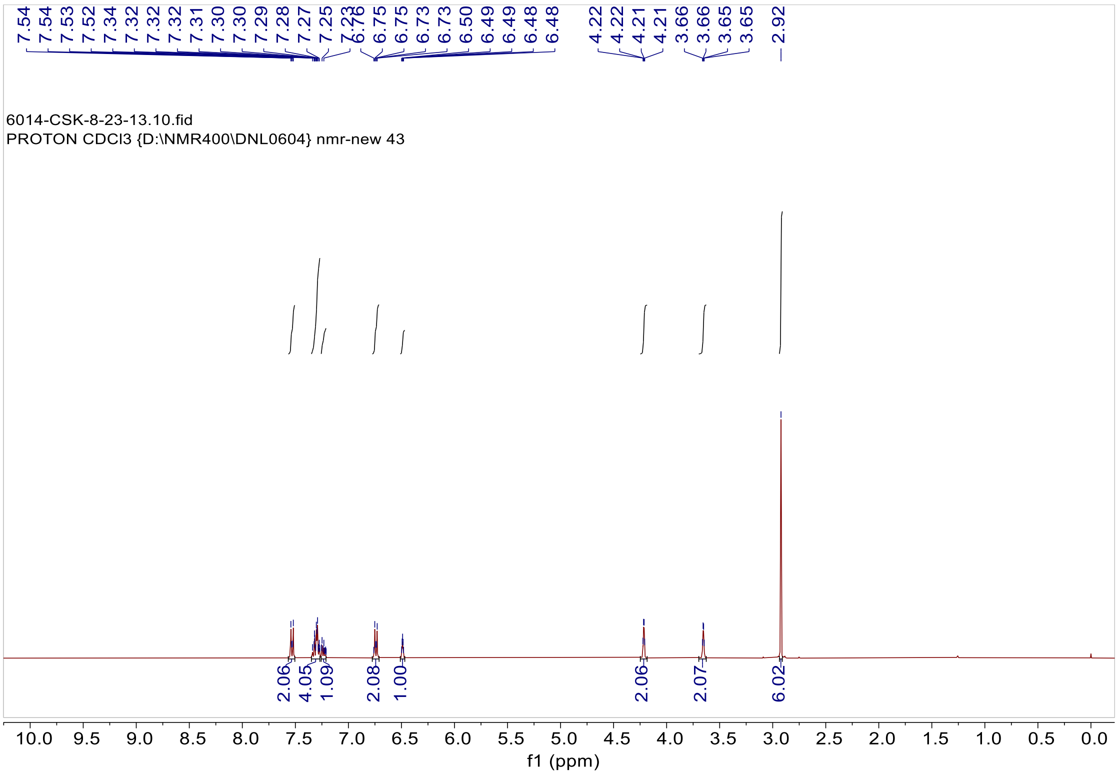

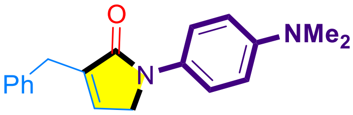

5i

^{1}H NMR (400 MHz, $CDCl_3$)
^{13}C NMR (100 MHz, $CDCl_3$)

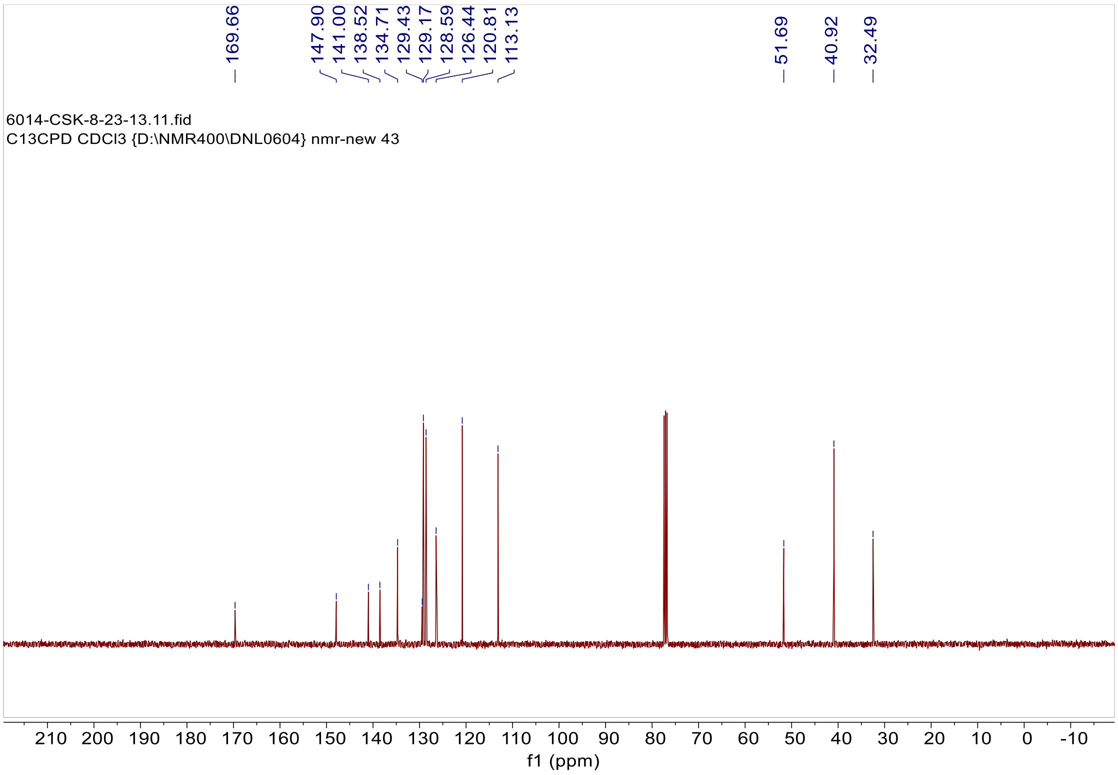

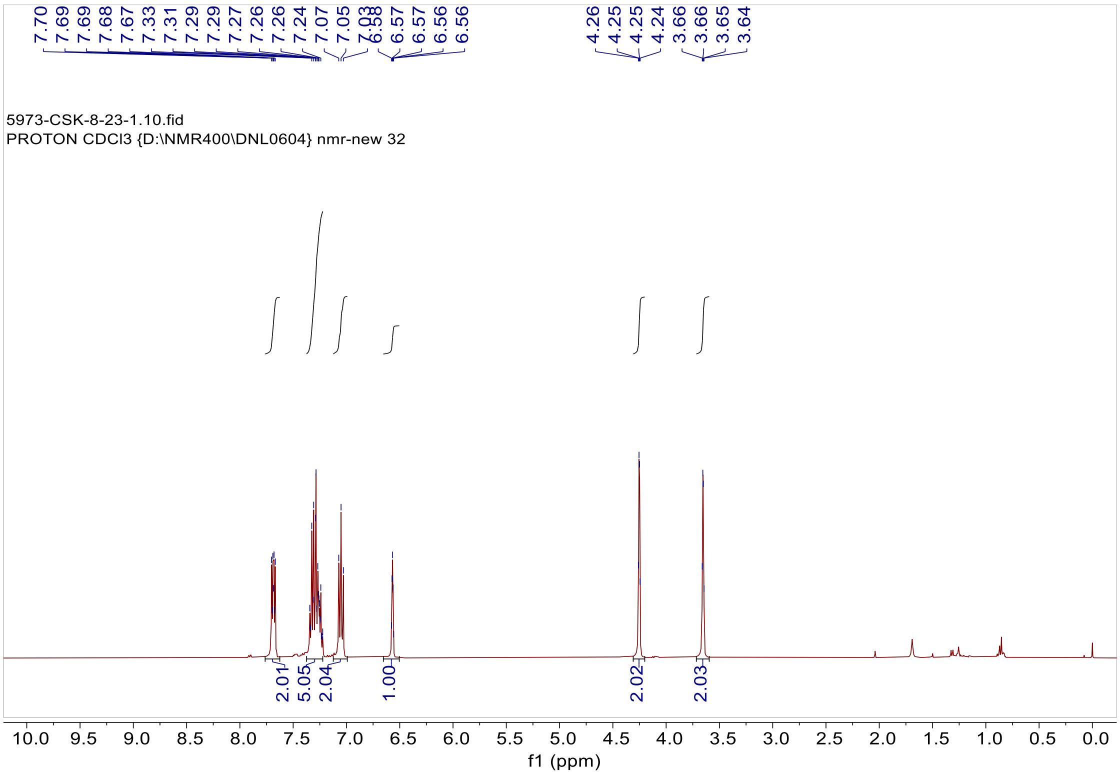

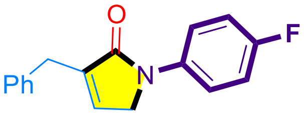

5j

^{1}H NMR (400 MHz, CDCl$_3$)

^{13}C NMR (100 MHz, CDCl$_3$)

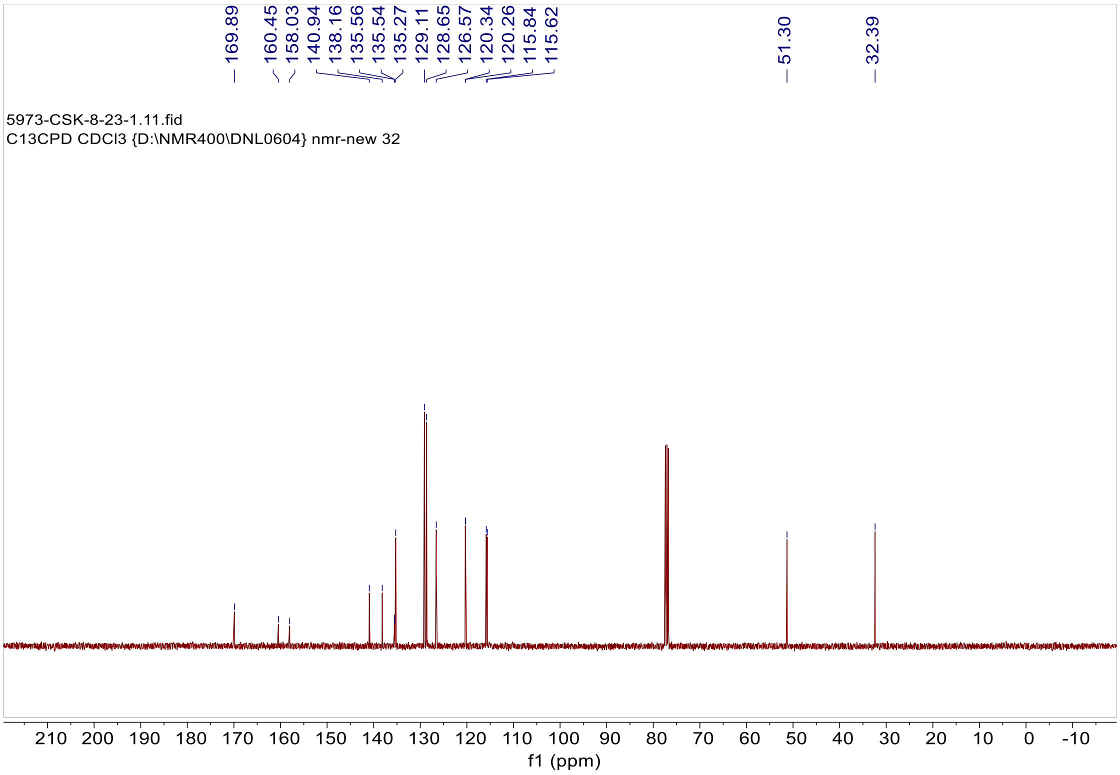

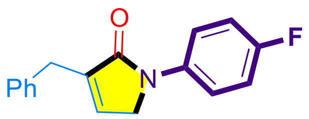

5j ^{19}F NMR (376 MHz, $CDCl_3$)

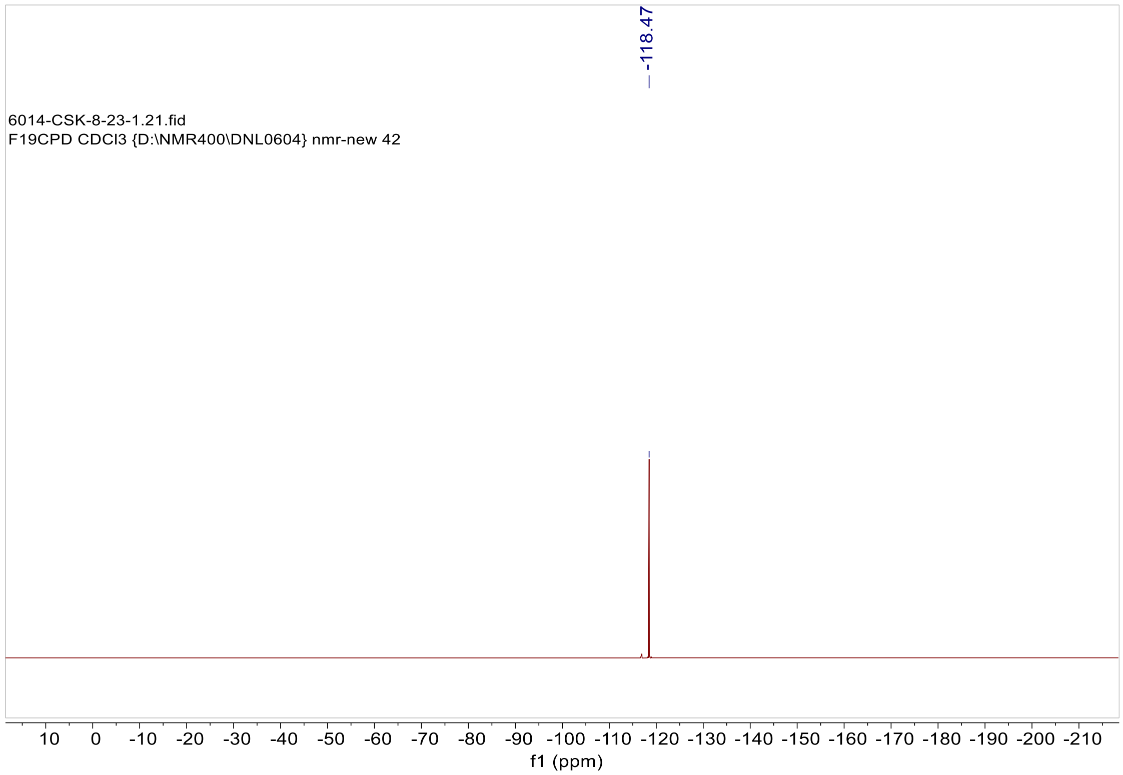

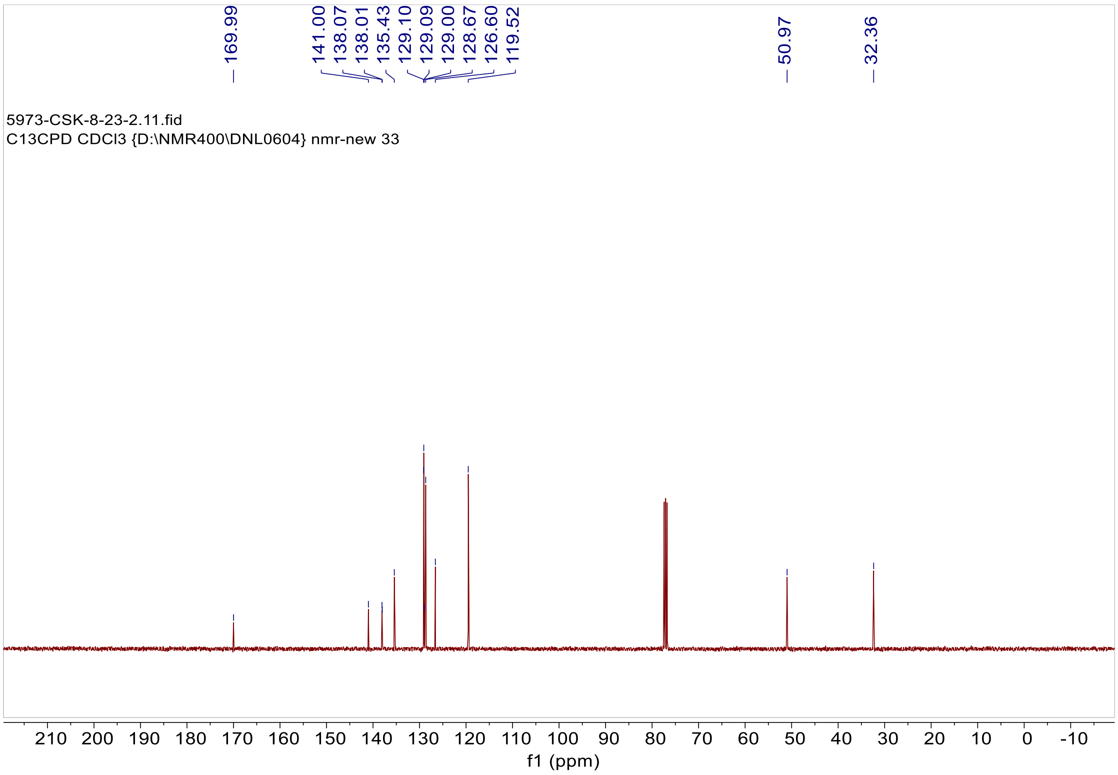

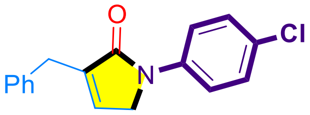

5k

^{1}H NMR (400 MHz, $CDCl_3$)

^{13}C NMR (100 MHz, $CDCl_3$)

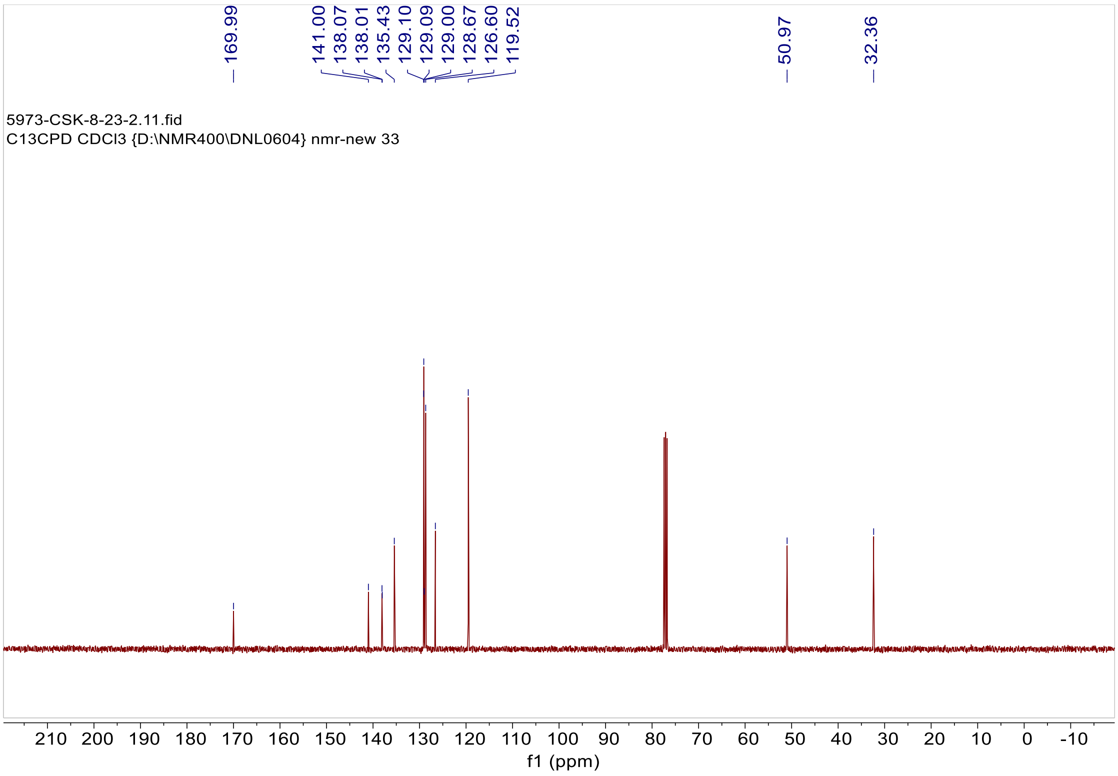

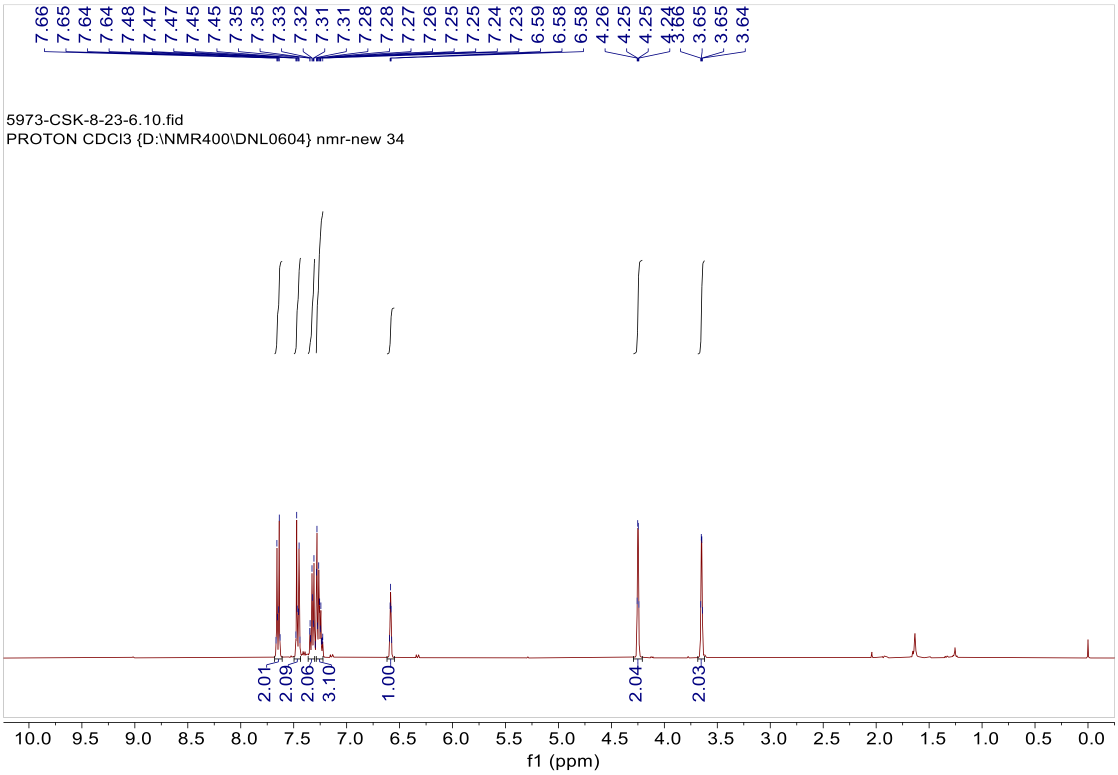

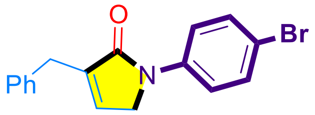

5l

^{1}H NMR (400 MHz, $CDCl_3$)
^{13}C NMR (100 MHz, $CDCl_3$)

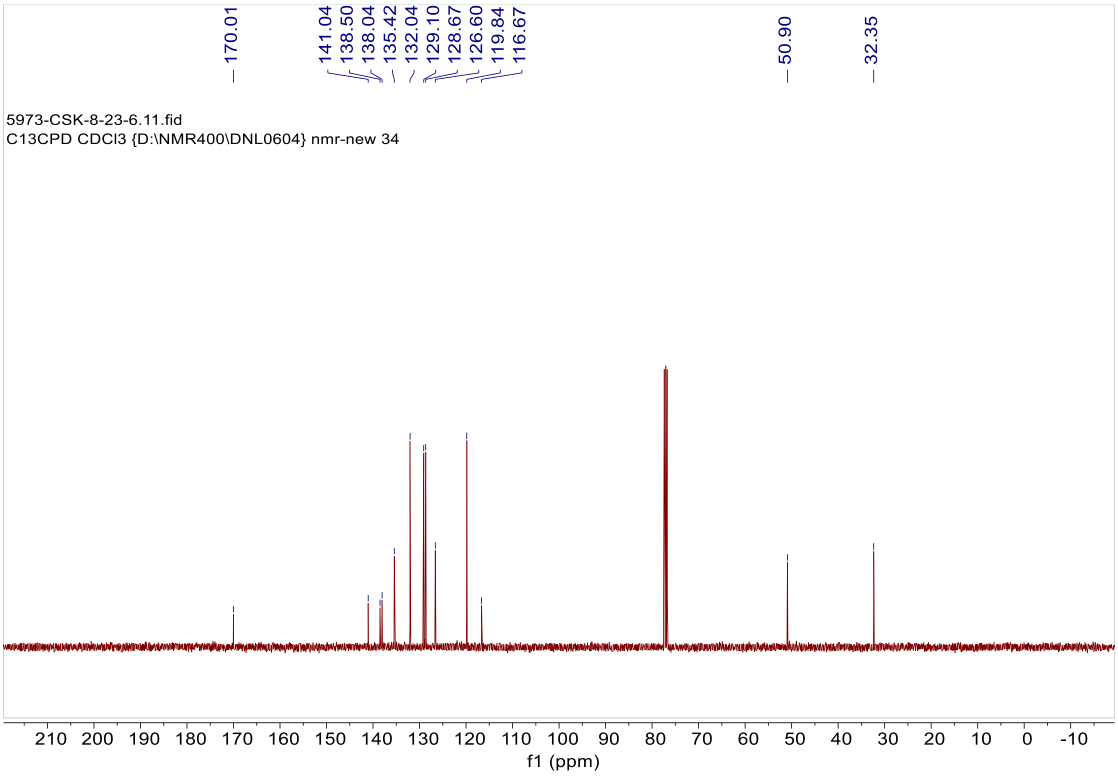

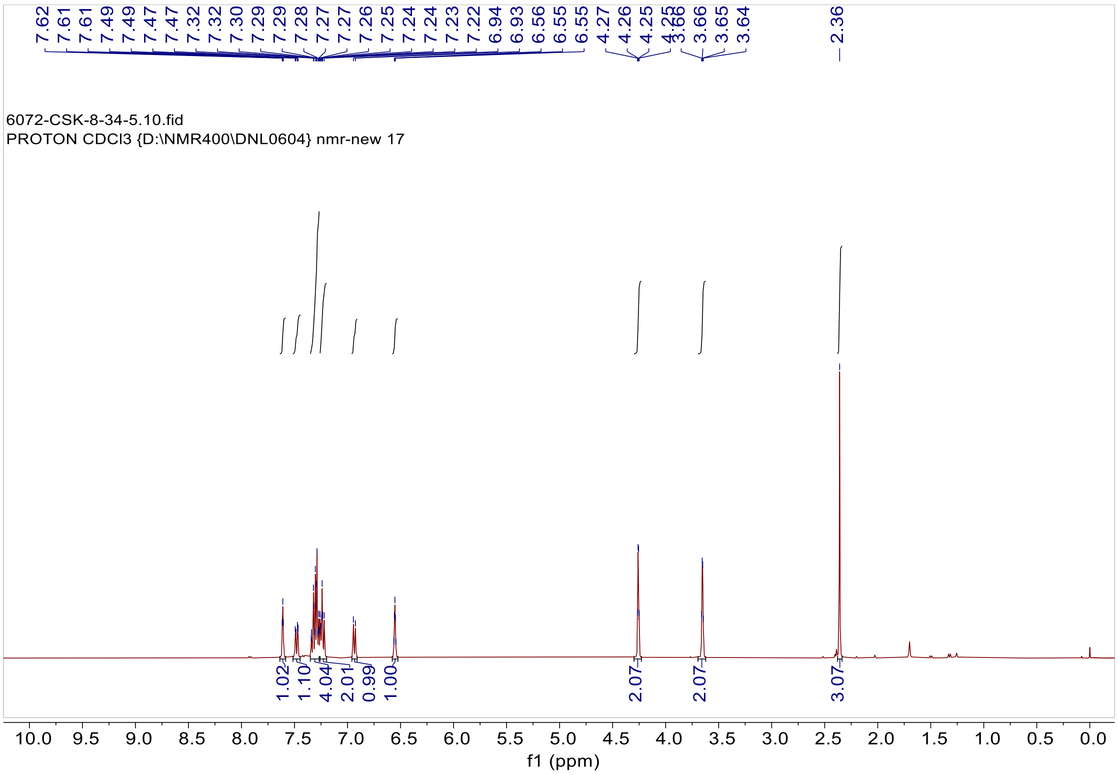

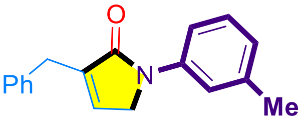

5m

^{1}H NMR (400 MHz, $CDCl_3$)
^{13}C NMR (100 MHz, $CDCl_3$)

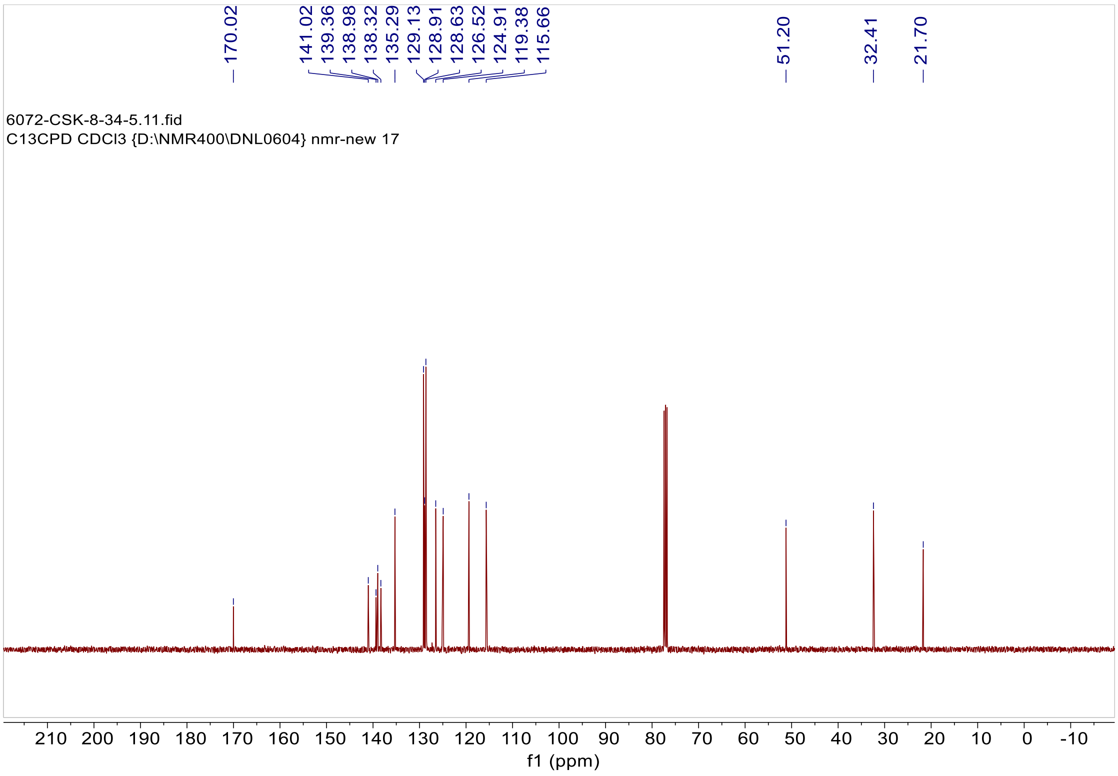

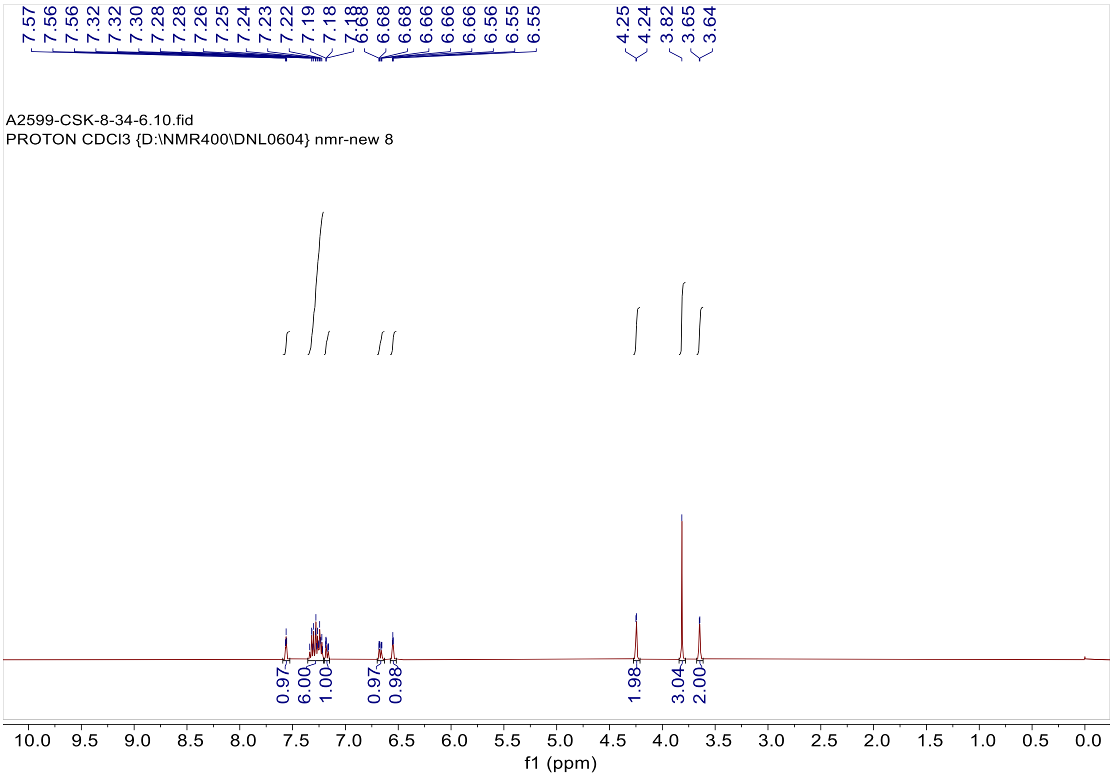

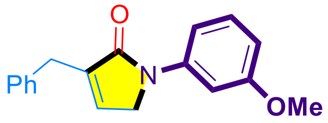

5n

^{1}H NMR (400 MHz, $CDCl_3$)
^{13}C NMR (100Hz, $CDCl_3$)

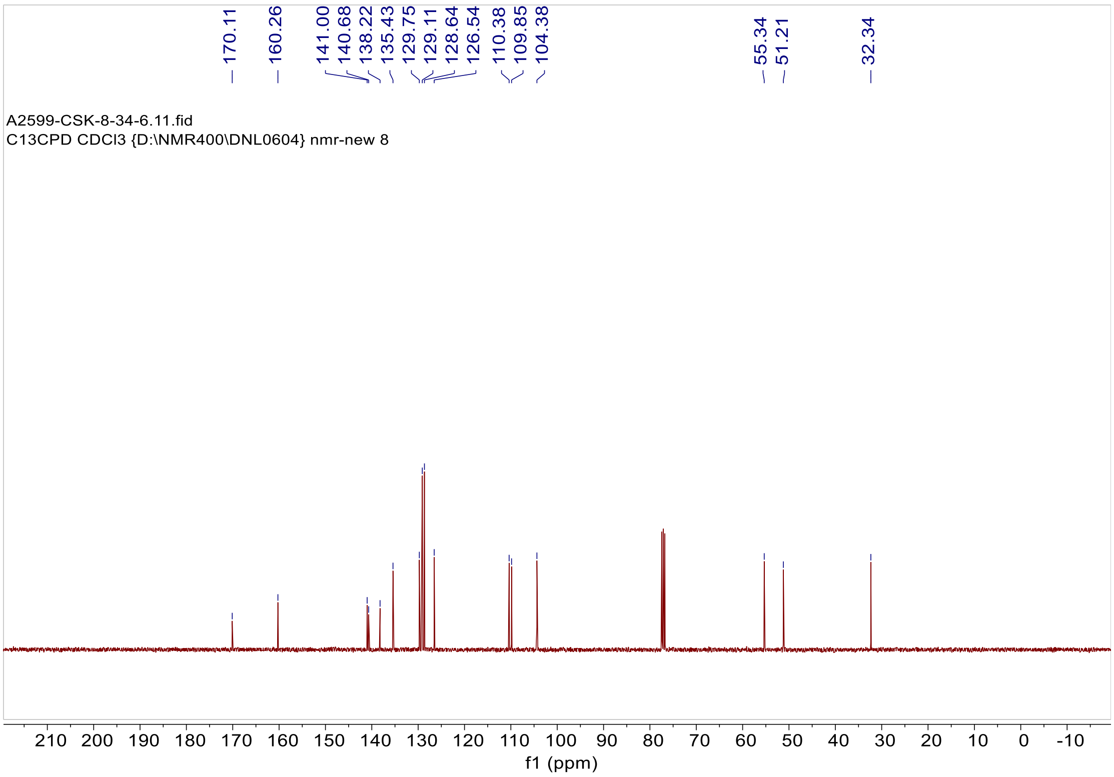

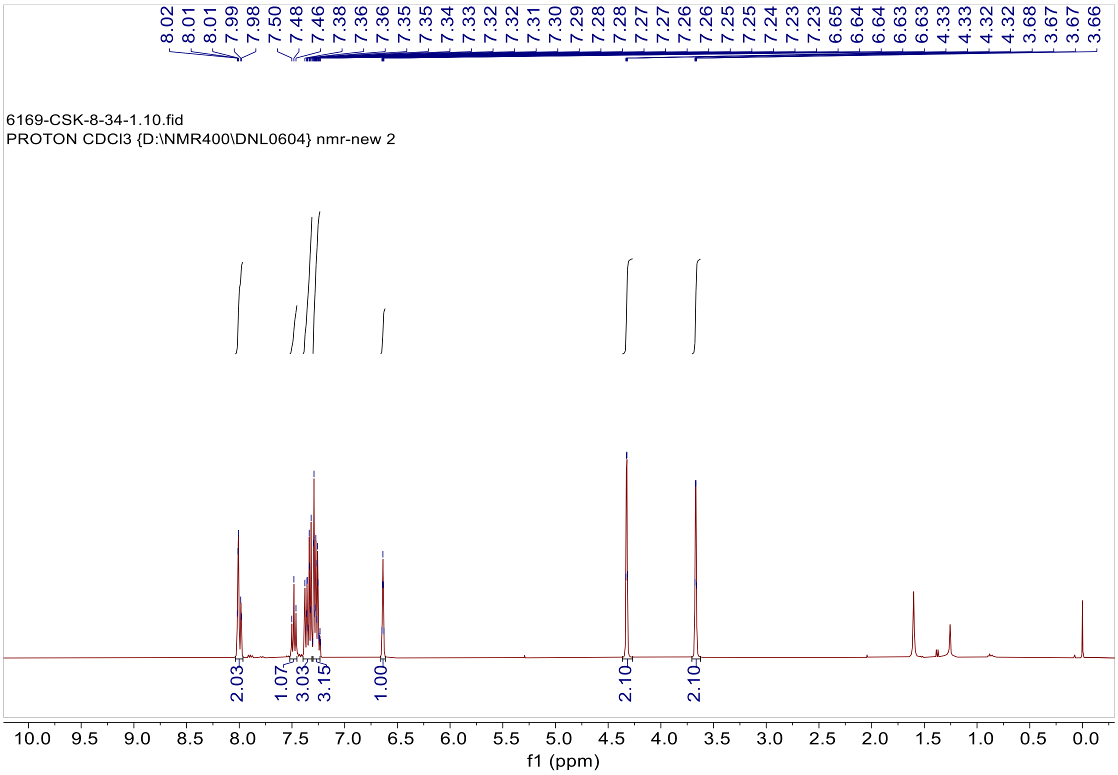

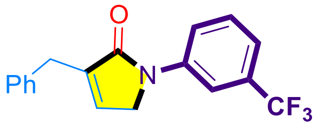

5o

^{1}H NMR (400 MHz, $CDCl_3$)
^{13}C NMR (100 MHz, $CDCl_3$)

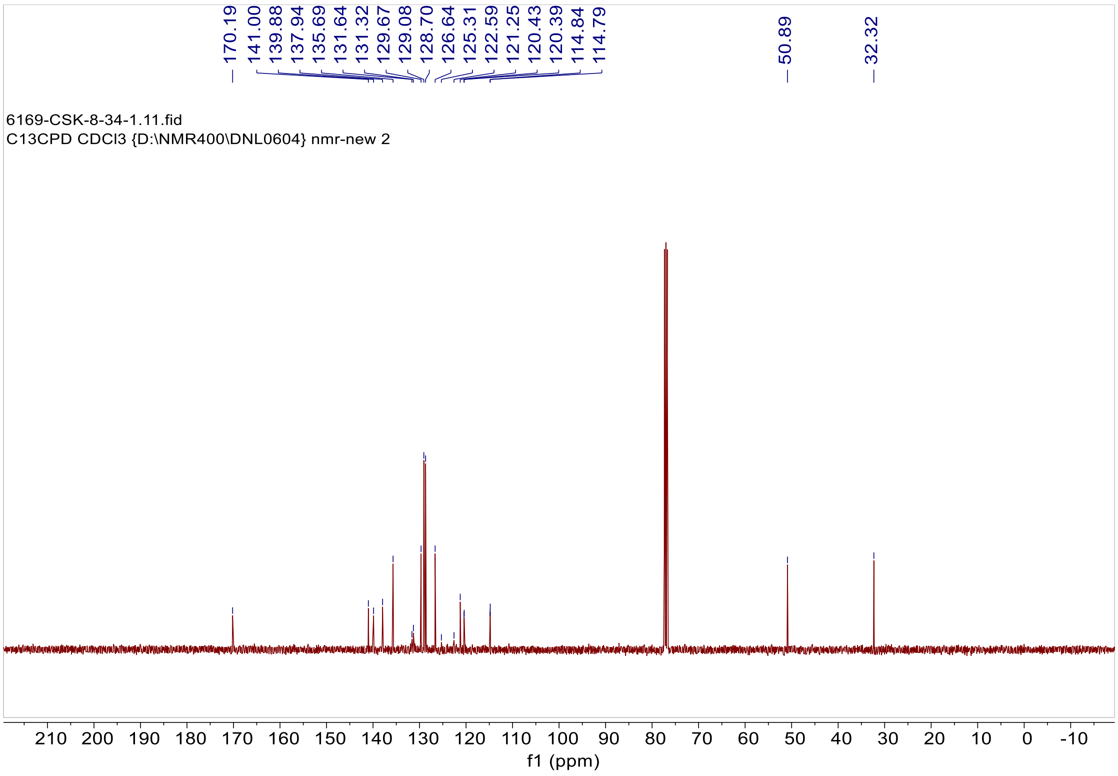

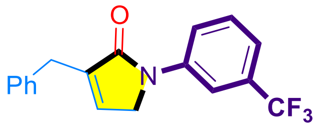

5o

^{19}F NMR (376 MHz, $CDCl_3$)

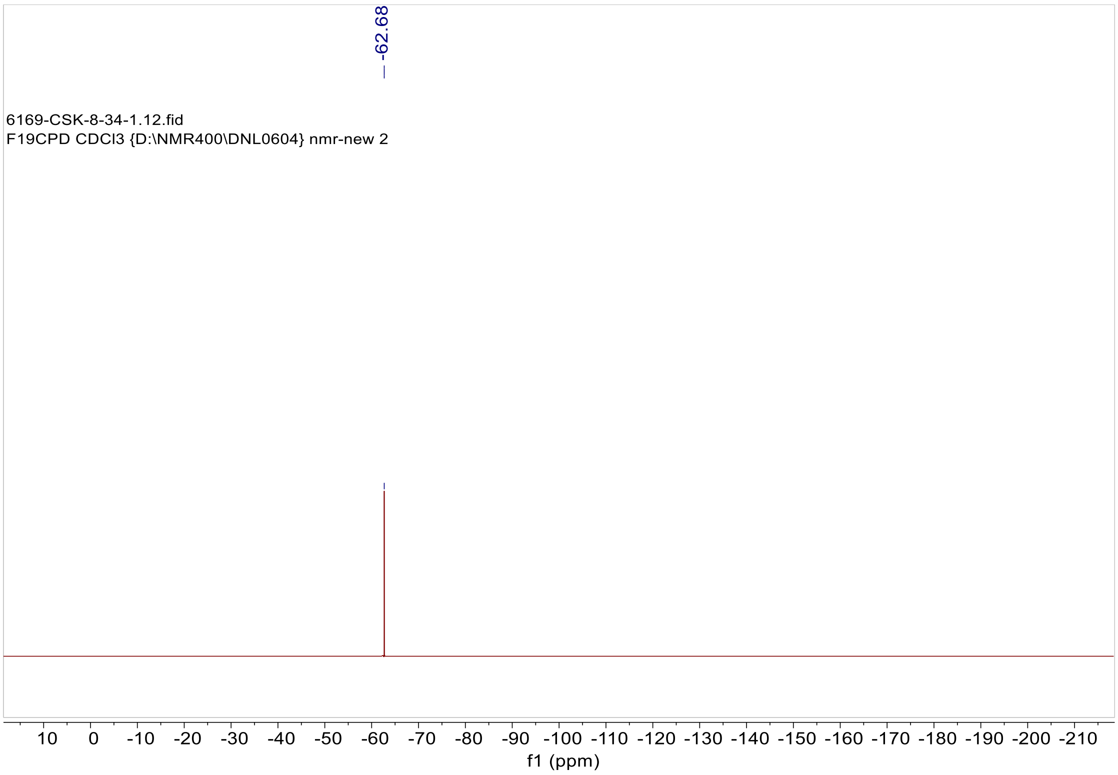

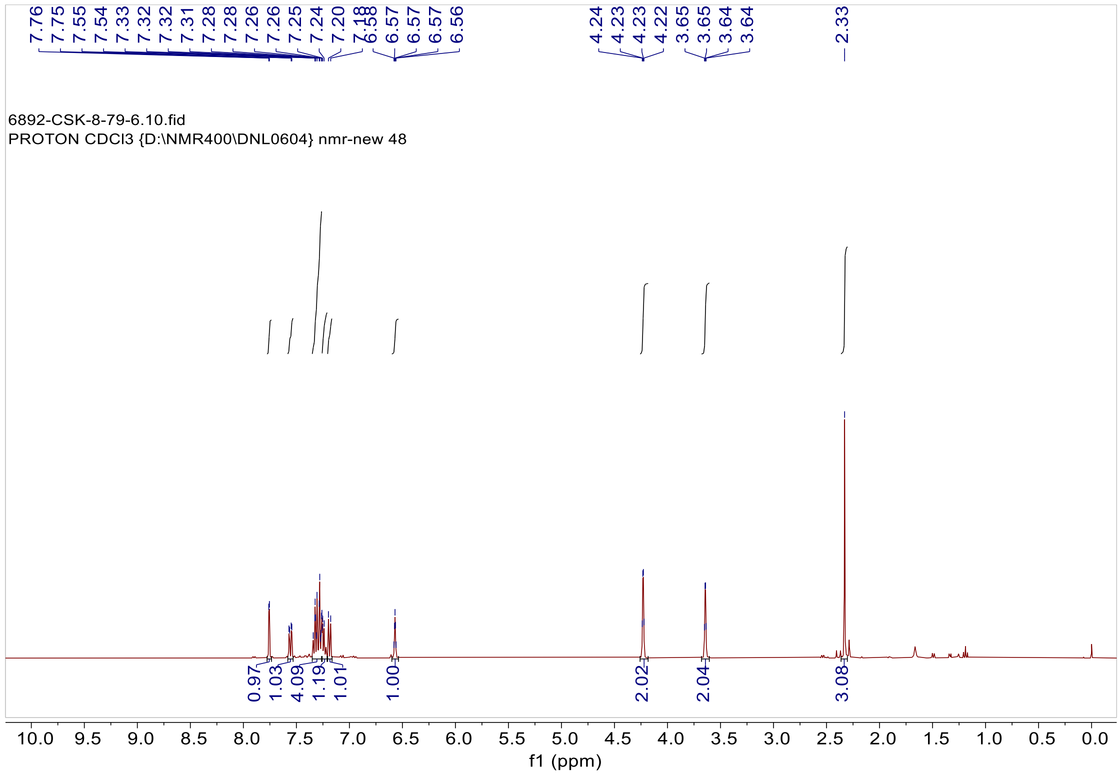

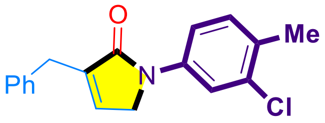

5p

^1H NMR (400 MHz, $CDCl_3$)
^{13}C NMR (100 MHz, $CDCl_3$)

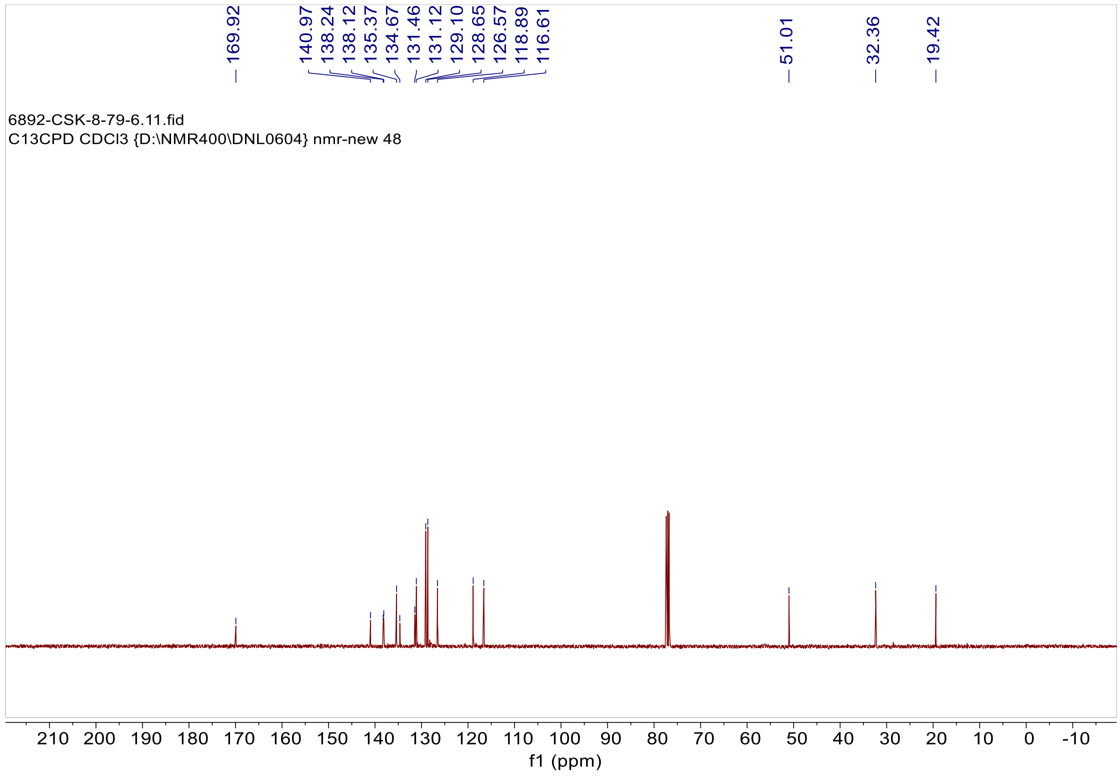

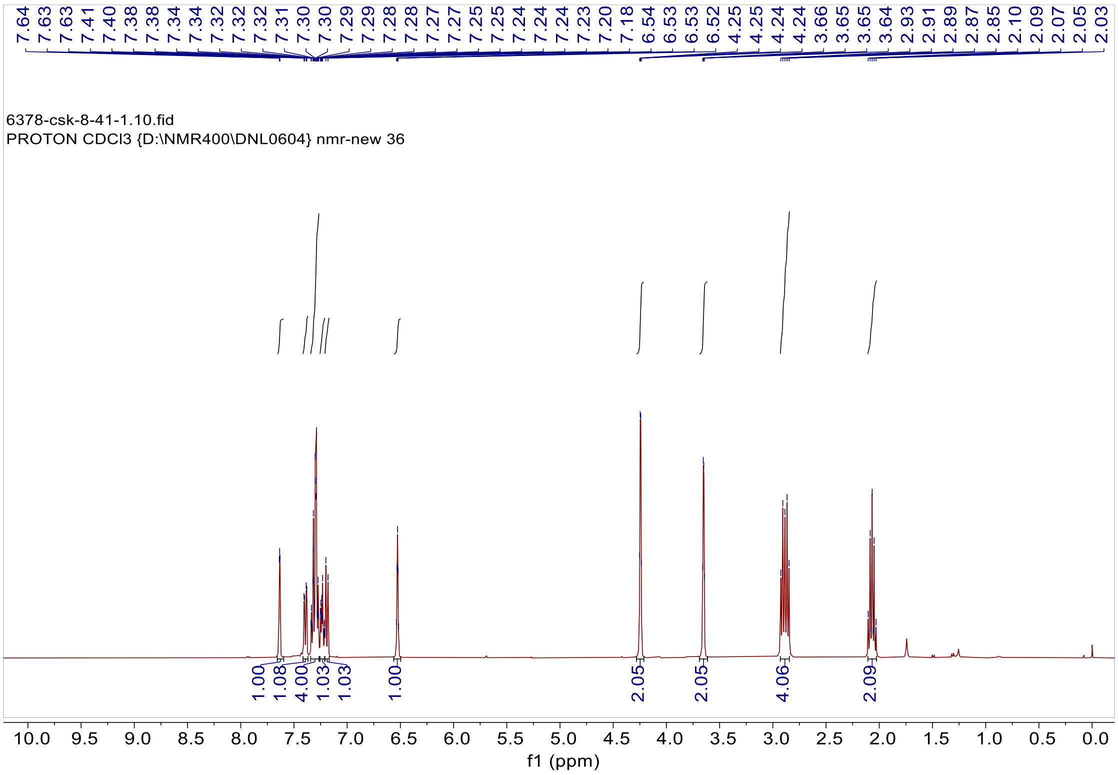

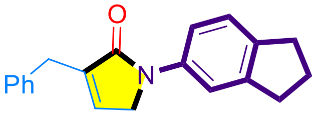

5r

^{1}H NMR (400 MHz, $CDCl_3$)

^{13}C NMR (100 MHz, $CDCl_3$)

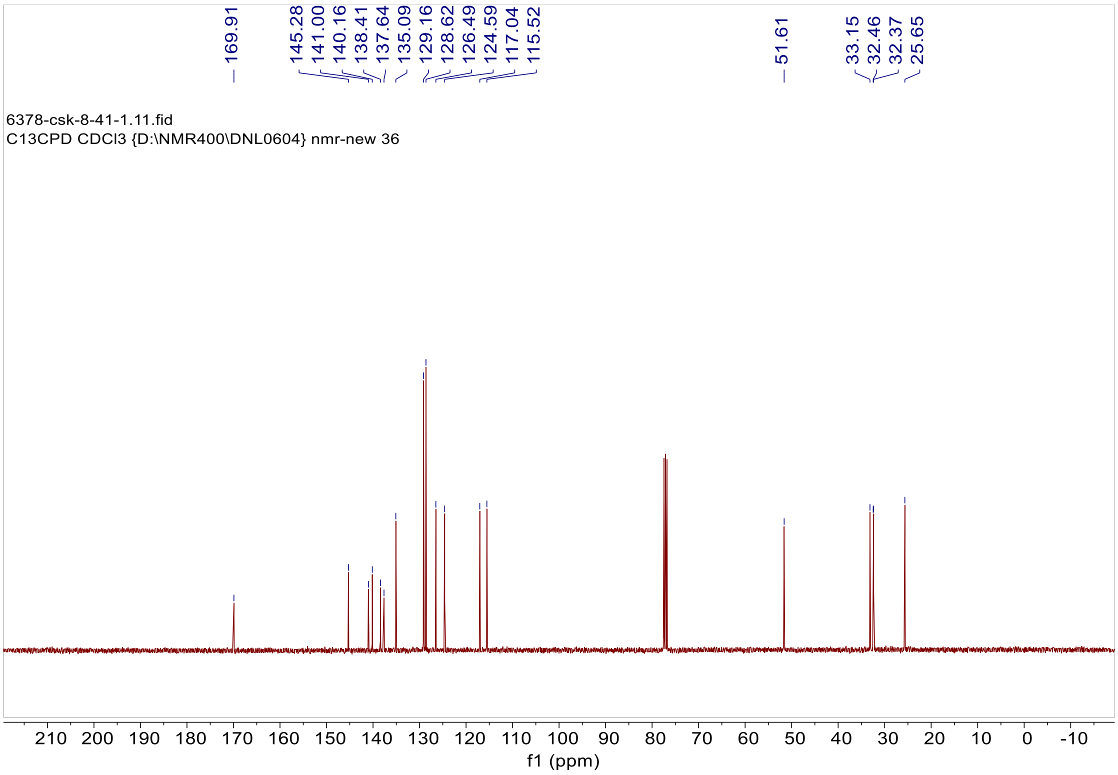

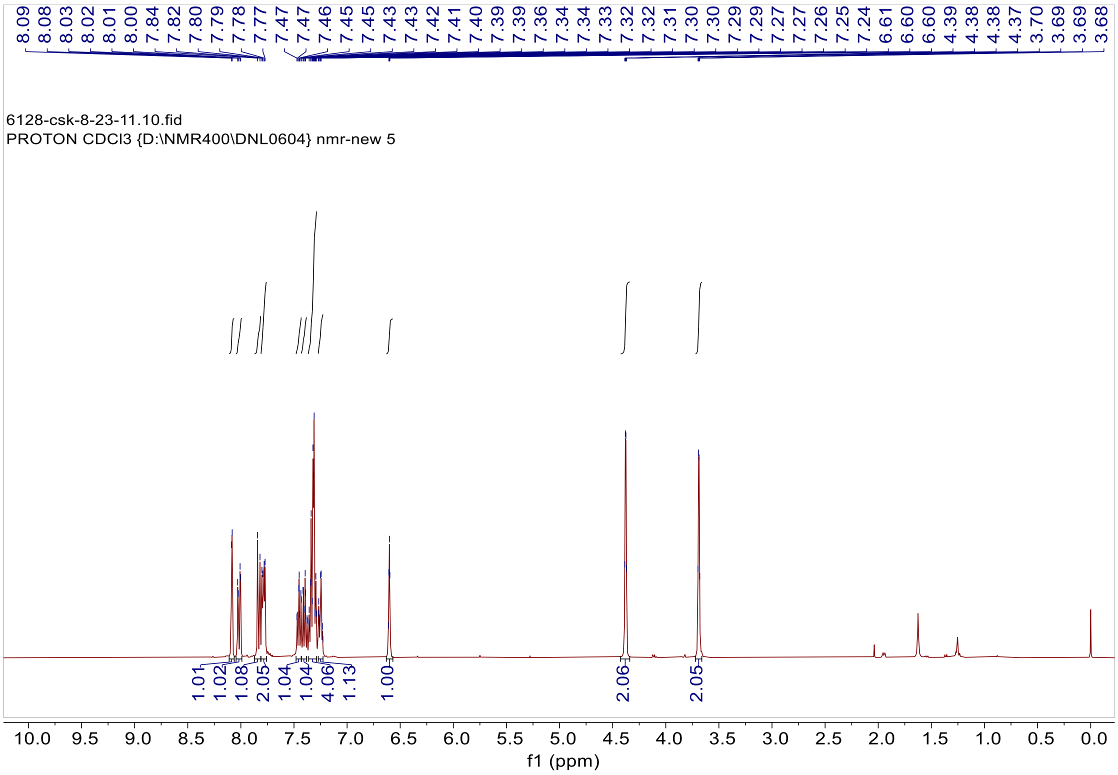

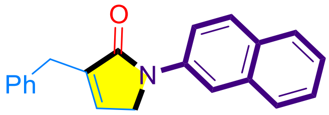

5s

^{1}H NMR (400 MHz, CDCl$_3$)
^{13}C NMR (100 MHz, CDCl$_3$)

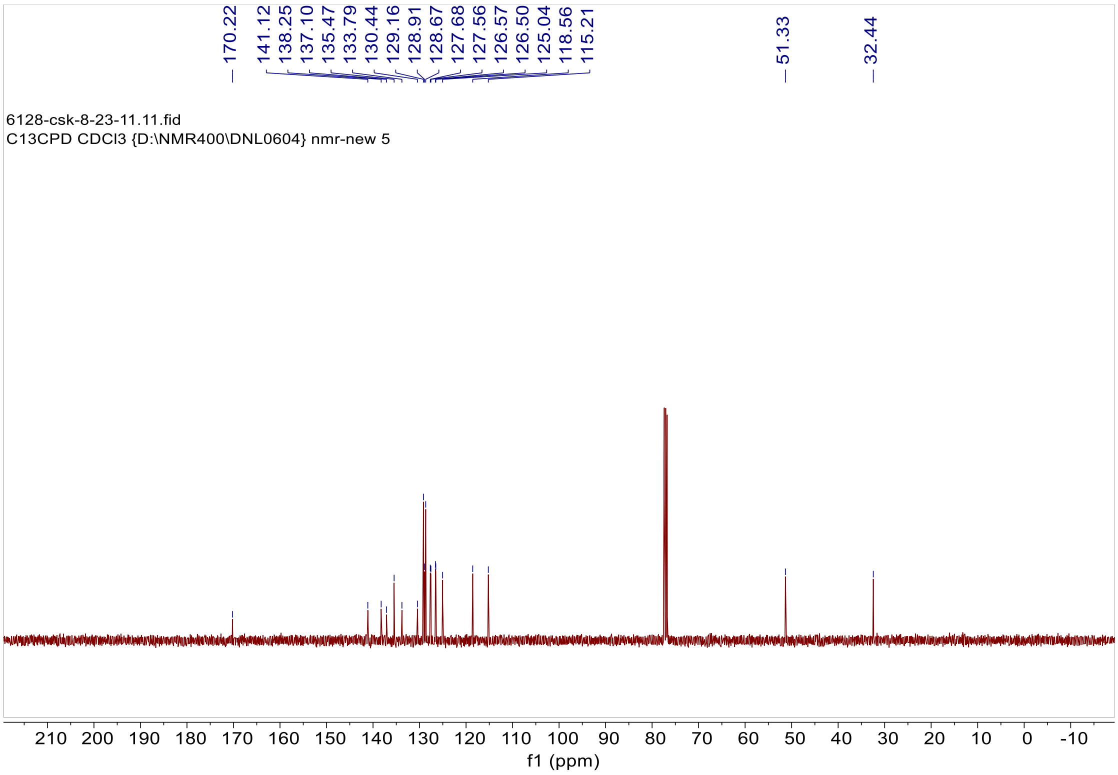

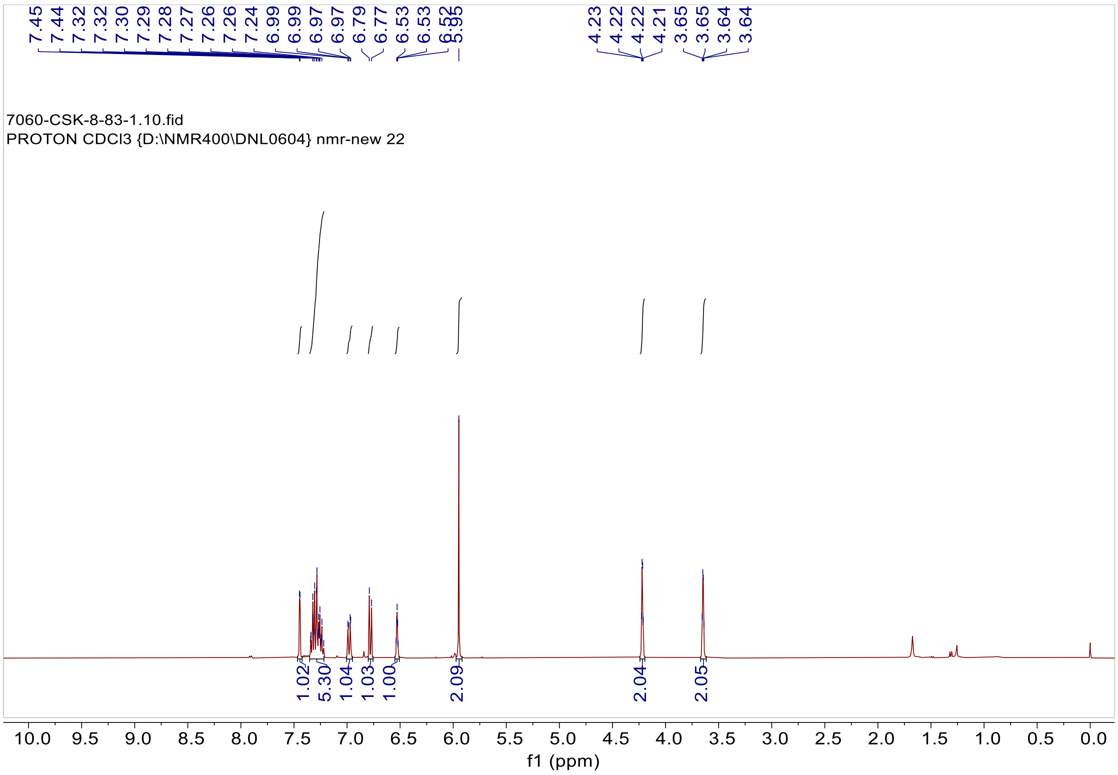

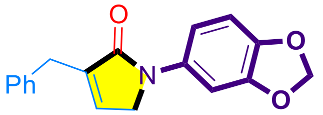

5t

^{1}H NMR (400 MHz, $CDCl_3$)
^{13}C NMR (100 MHz, $CDCl_3$)

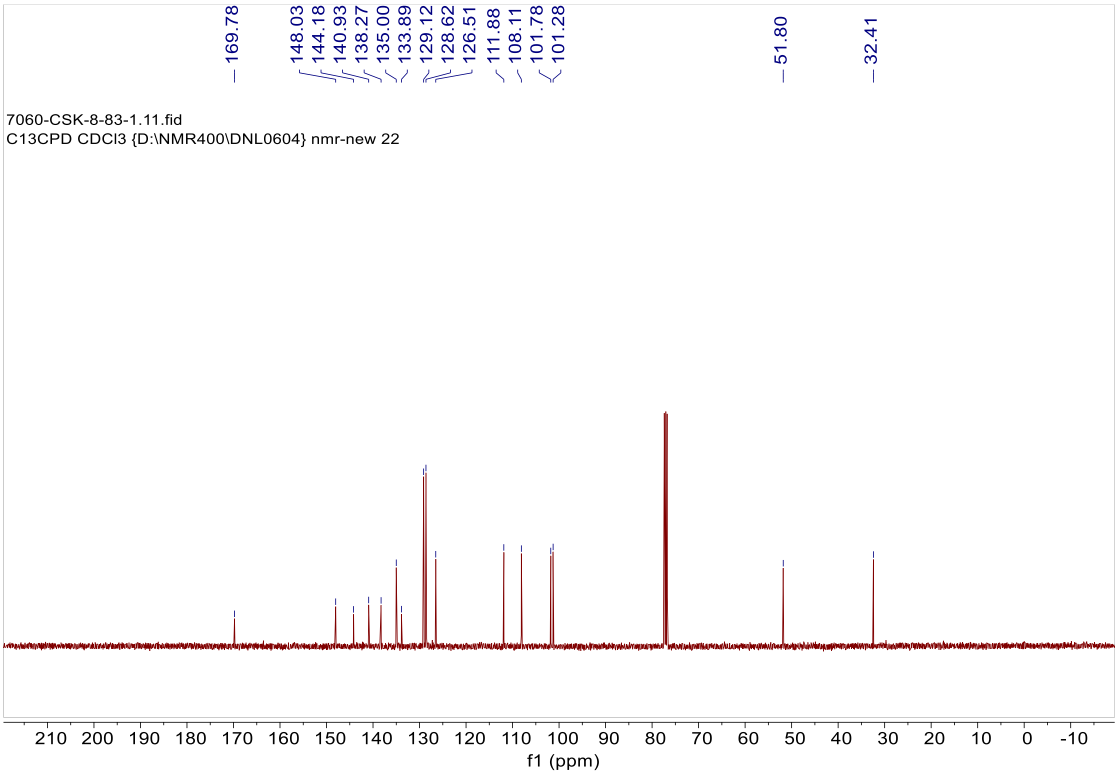

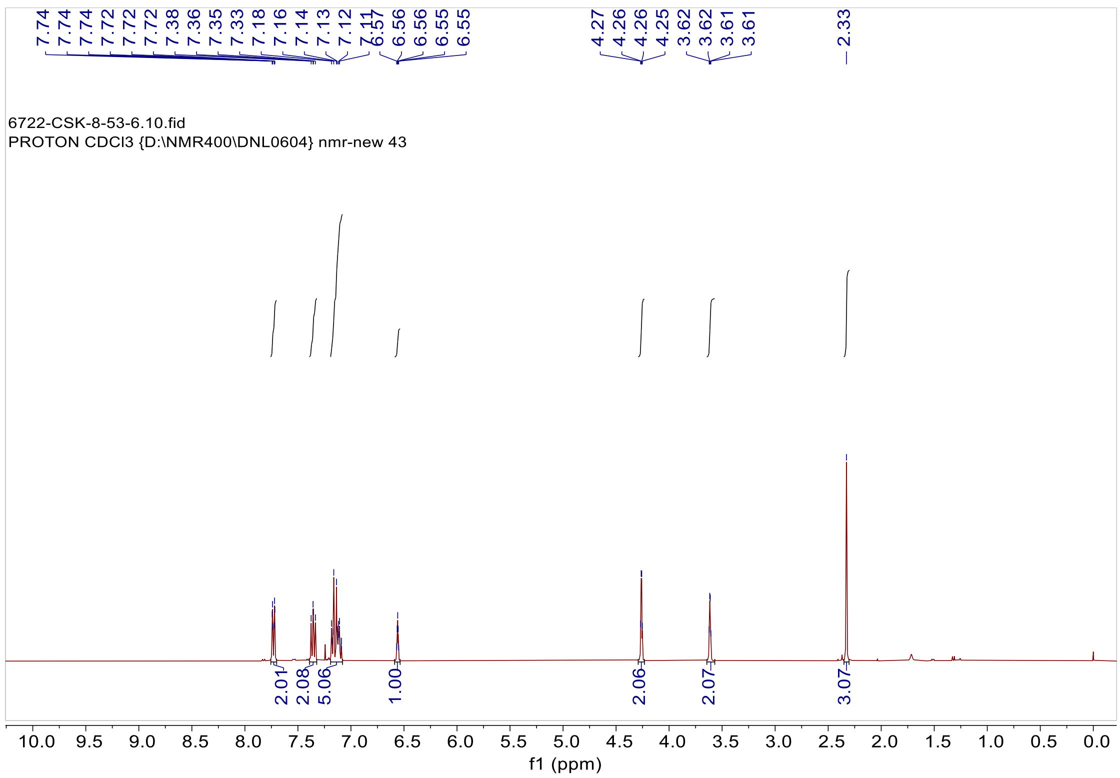

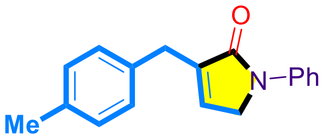

5u

^{1}H NMR (400 MHz, $CDCl_3$)

^{13}C NMR (100 MHz, $CDCl_3$)

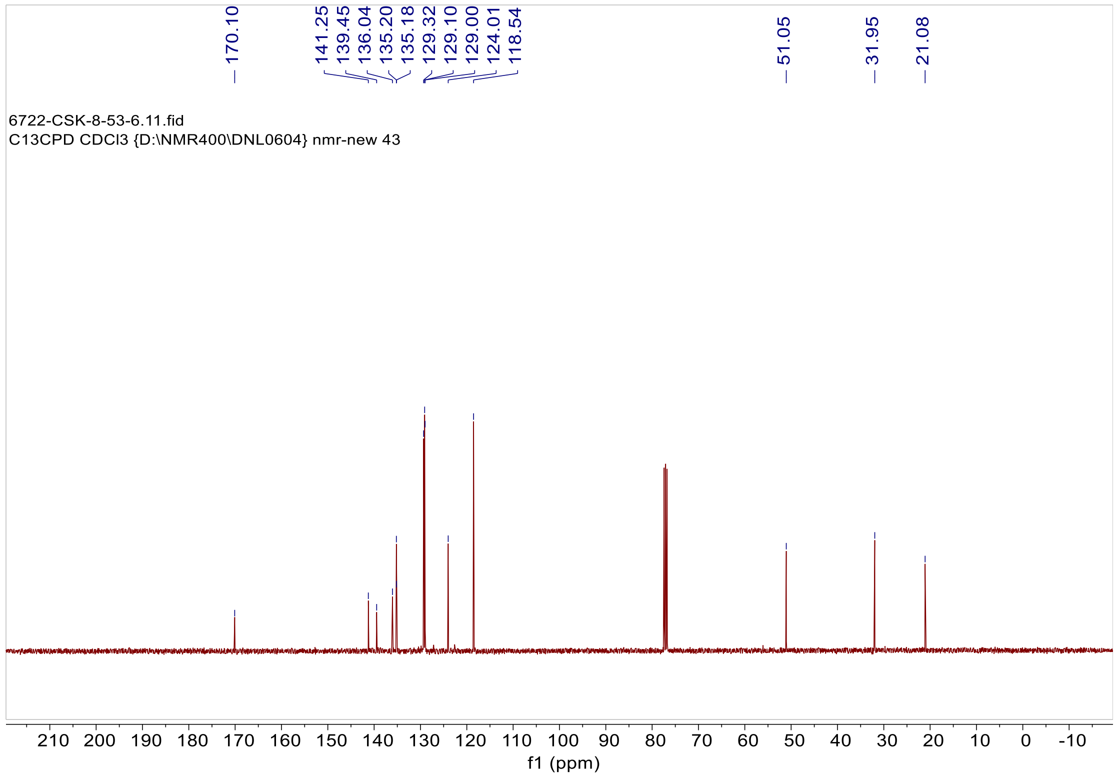

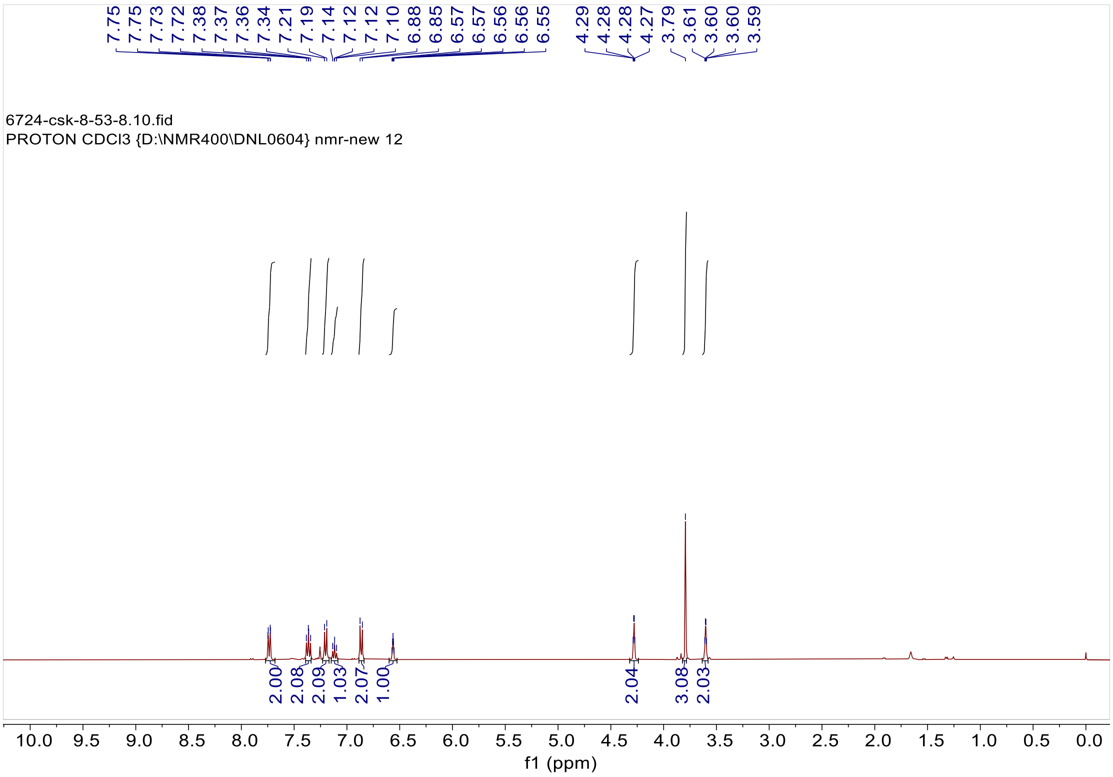

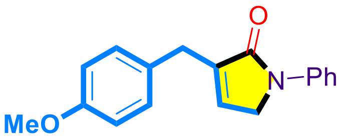

5v

^{1}H NMR (400 MHz, $CDCl_3$)
^{13}C NMR (100 MHz, $CDCl_3$)

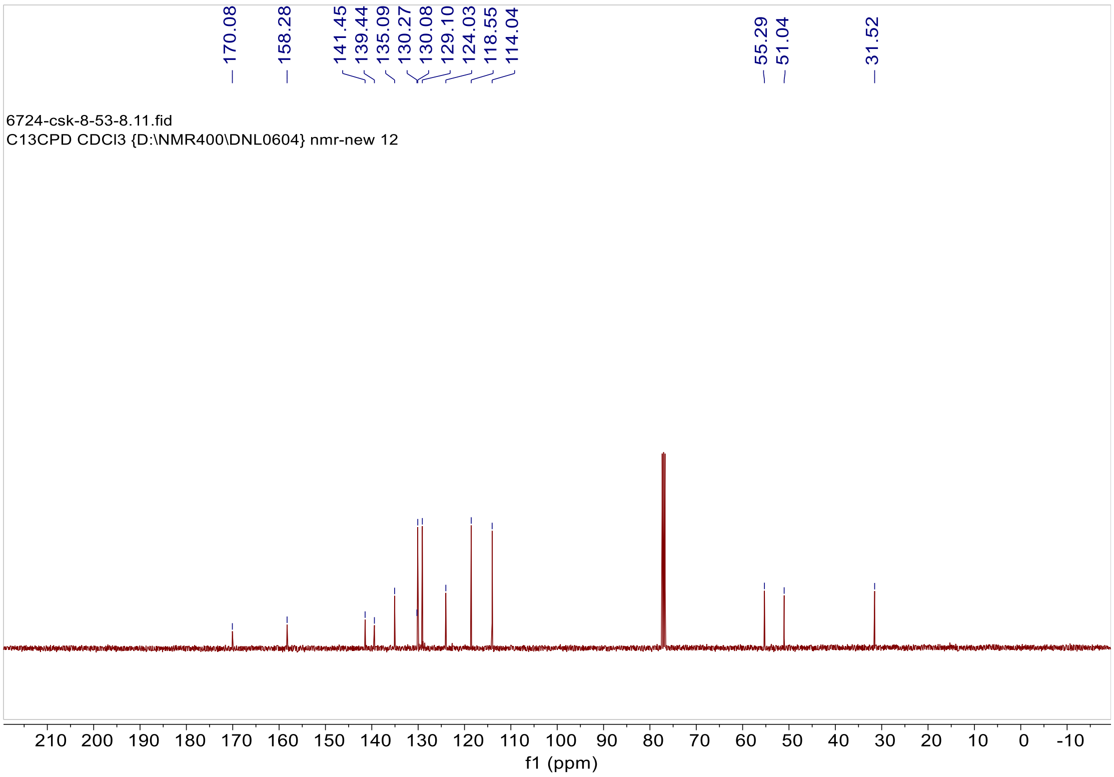

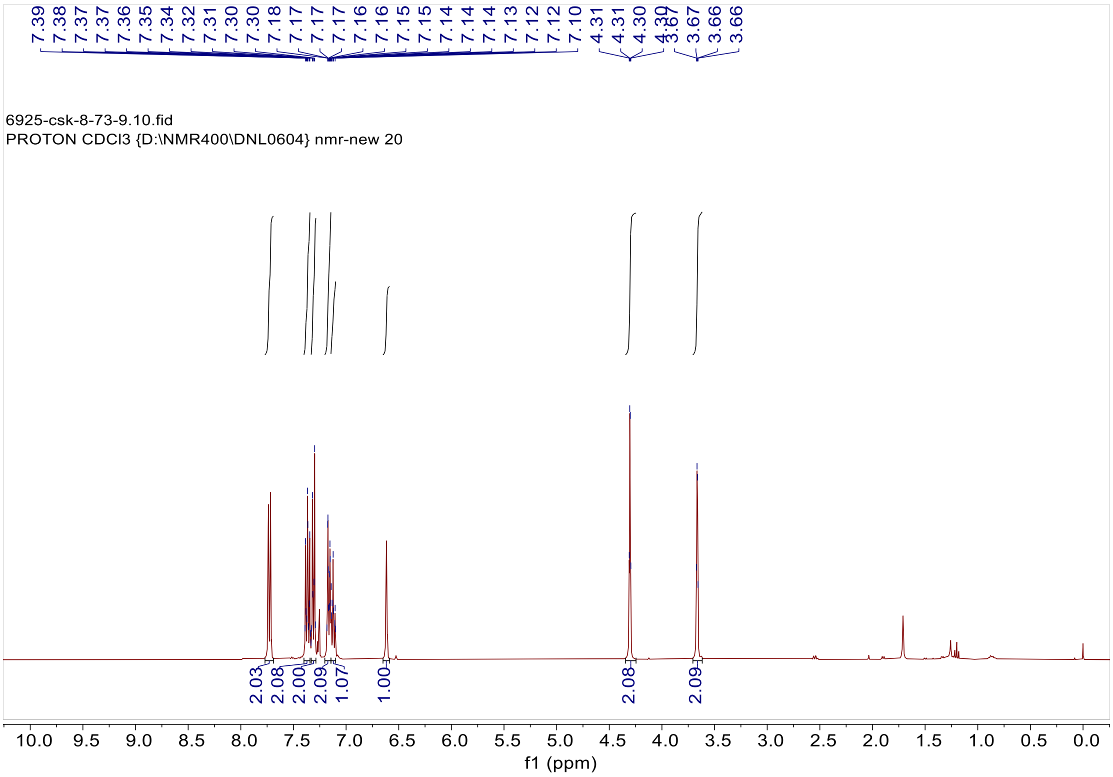

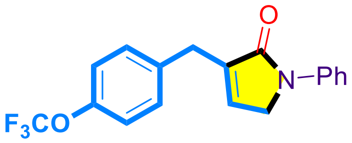

5w

^{1}H NMR (400 MHz, $CDCl_3$)

^{13}C NMR (100 MHz, $CDCl_3$)

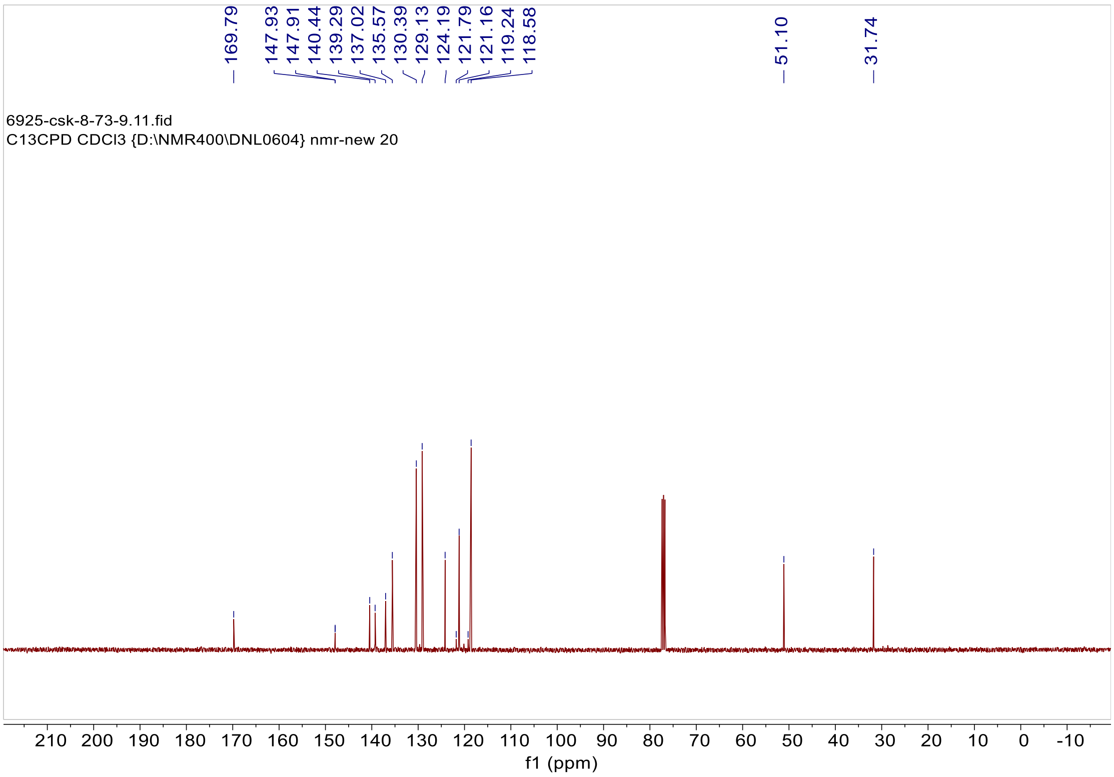

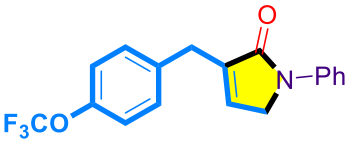

5w

^{19}F NMR (376 MHz, $CDCl_3$)

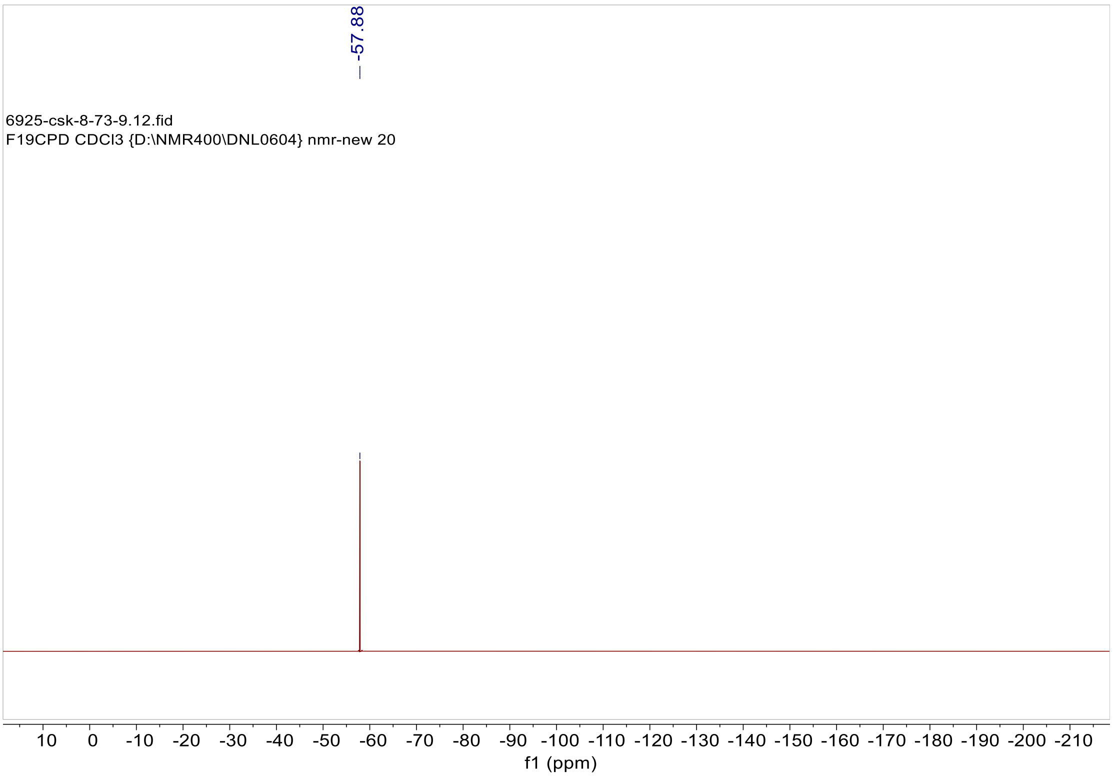

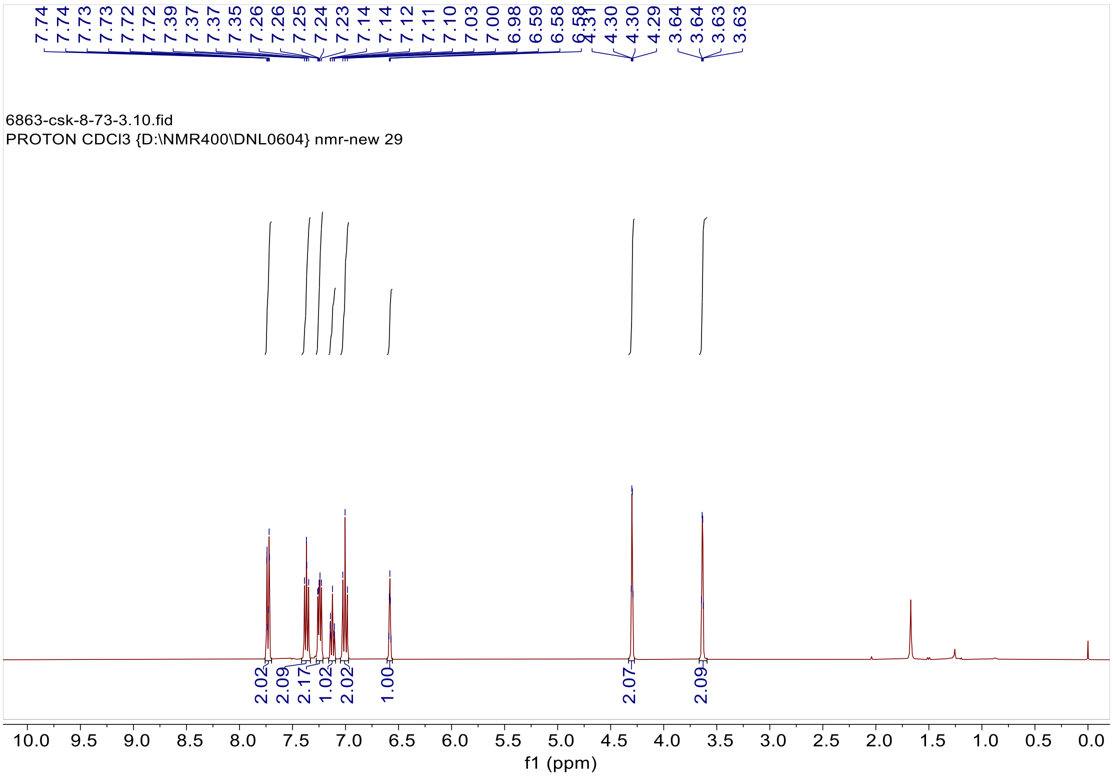

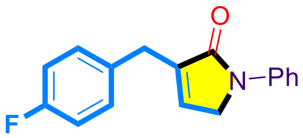

5x

^{1}H NMR (400 MHz, $CDCl_3$)

^{13}C NMR (100 MHz, $CDCl_3$)

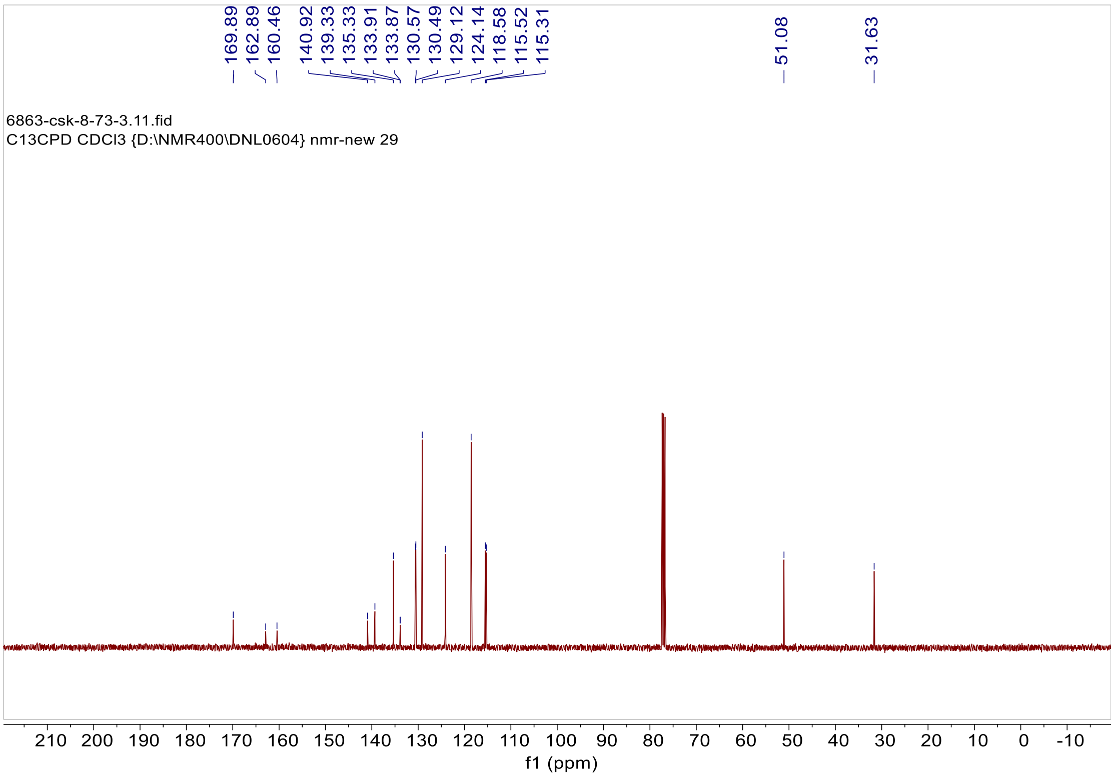

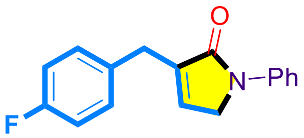

5x ^{19}F NMR (376 MHz, $CDCl_3$)

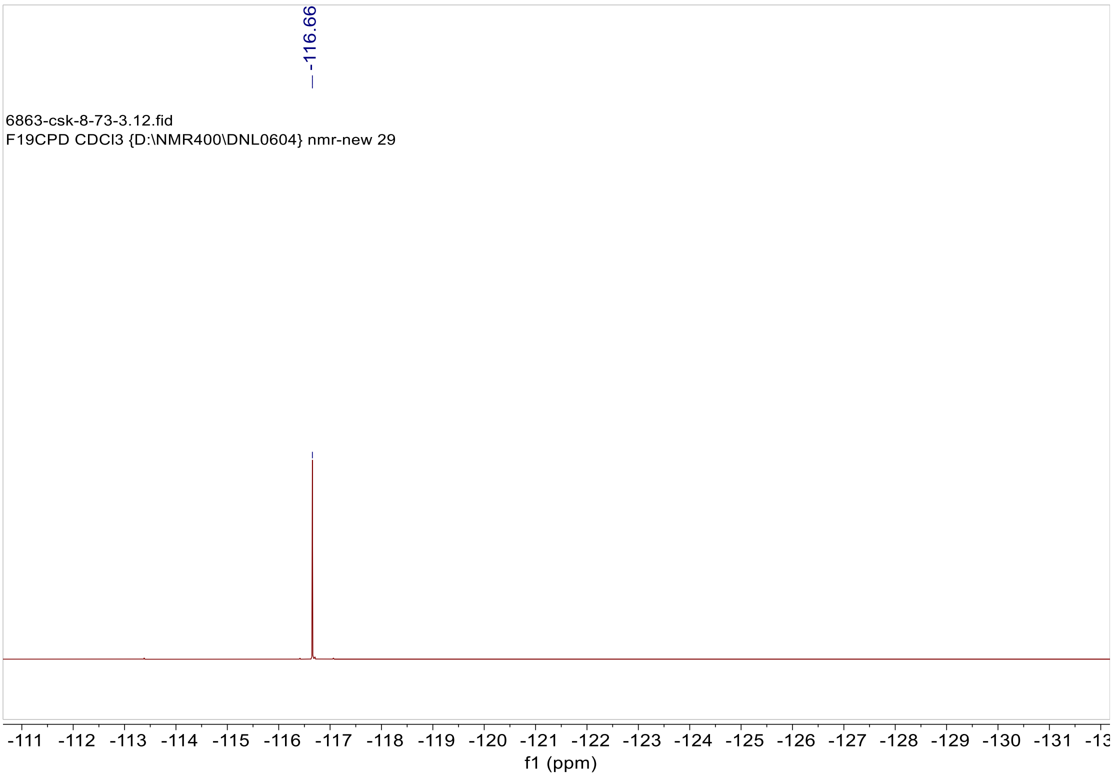

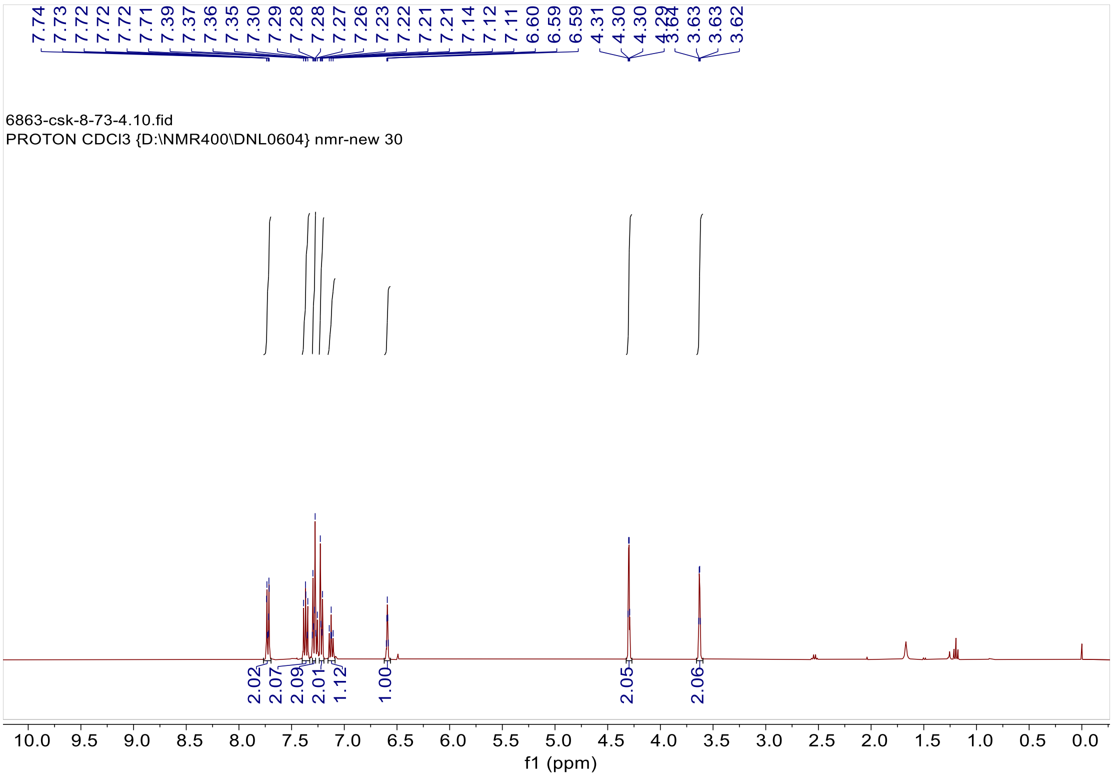

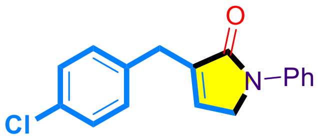

5y

^{1}H NMR (400 MHz, $CDCl_3$)

^{13}C NMR (100 MHz, $CDCl_3$)

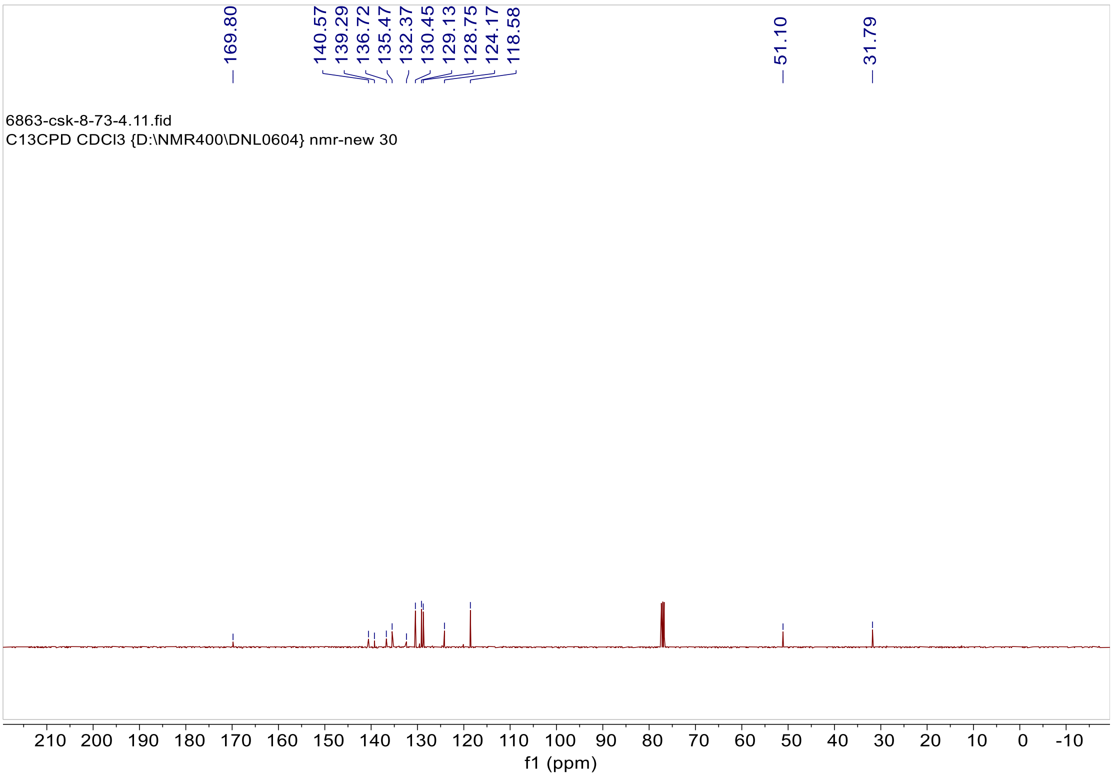

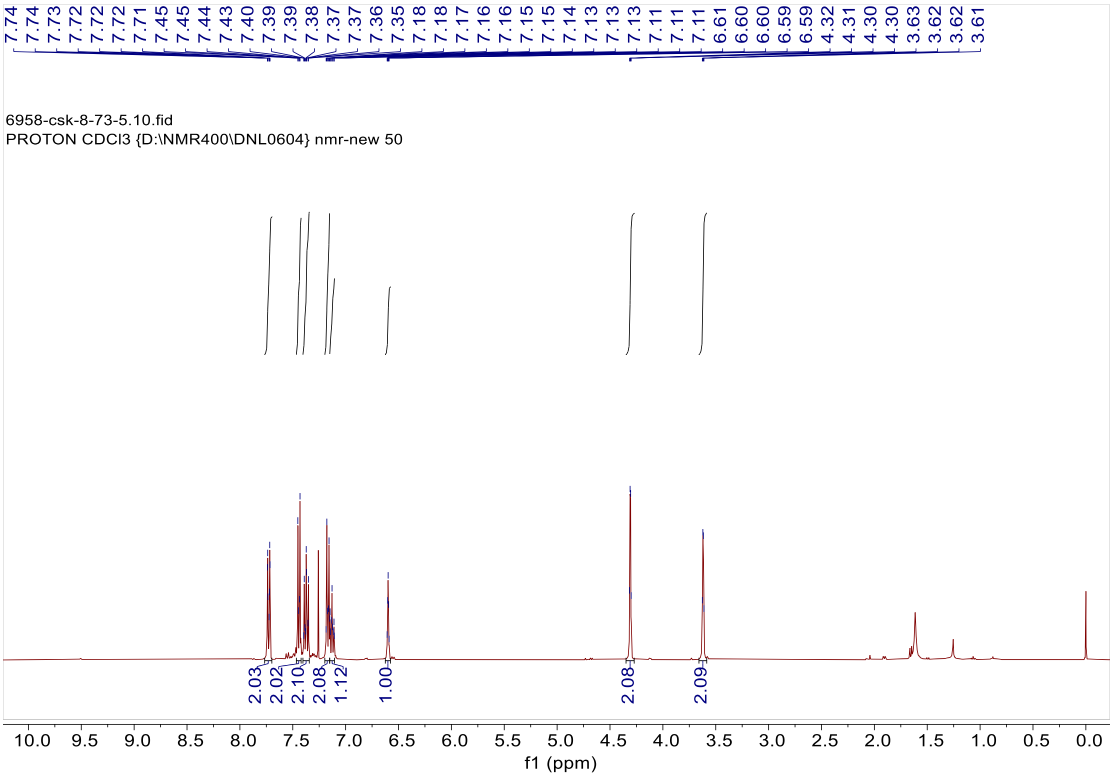

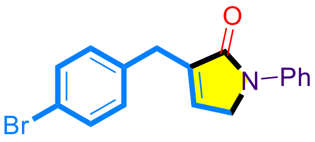

5z

^{1}H NMR (400 MHz, $CDCl_3$)
^{13}C NMR (100 MHz, $CDCl_3$)

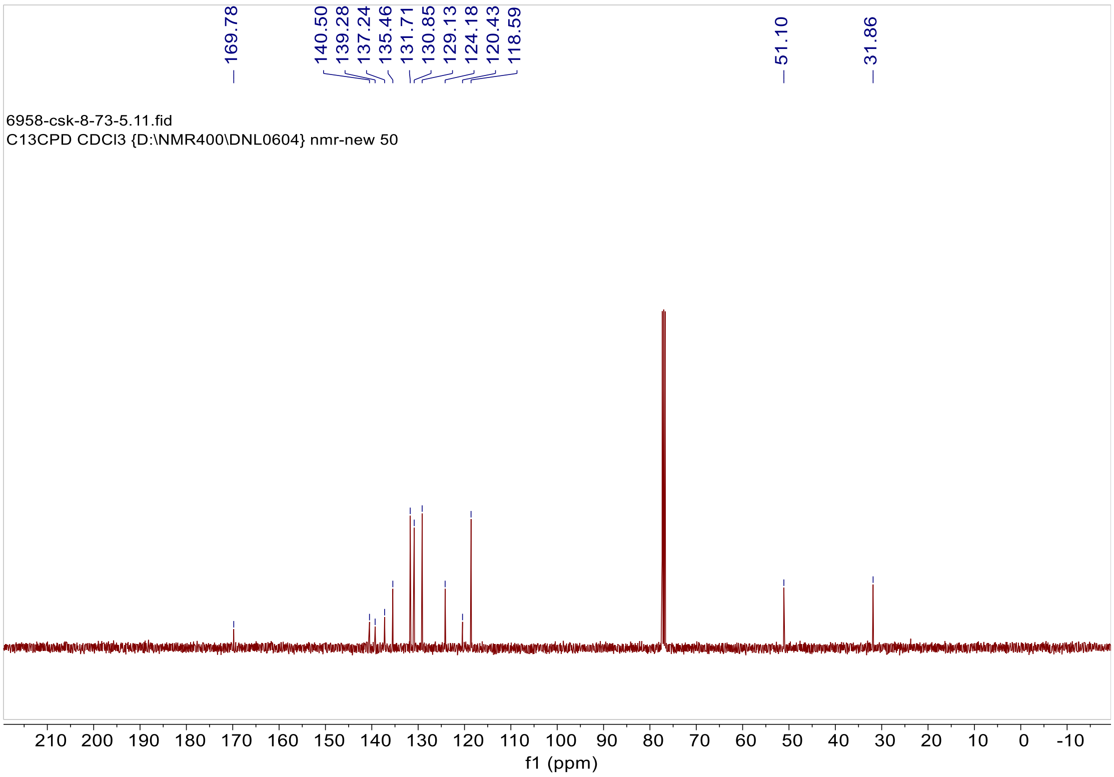

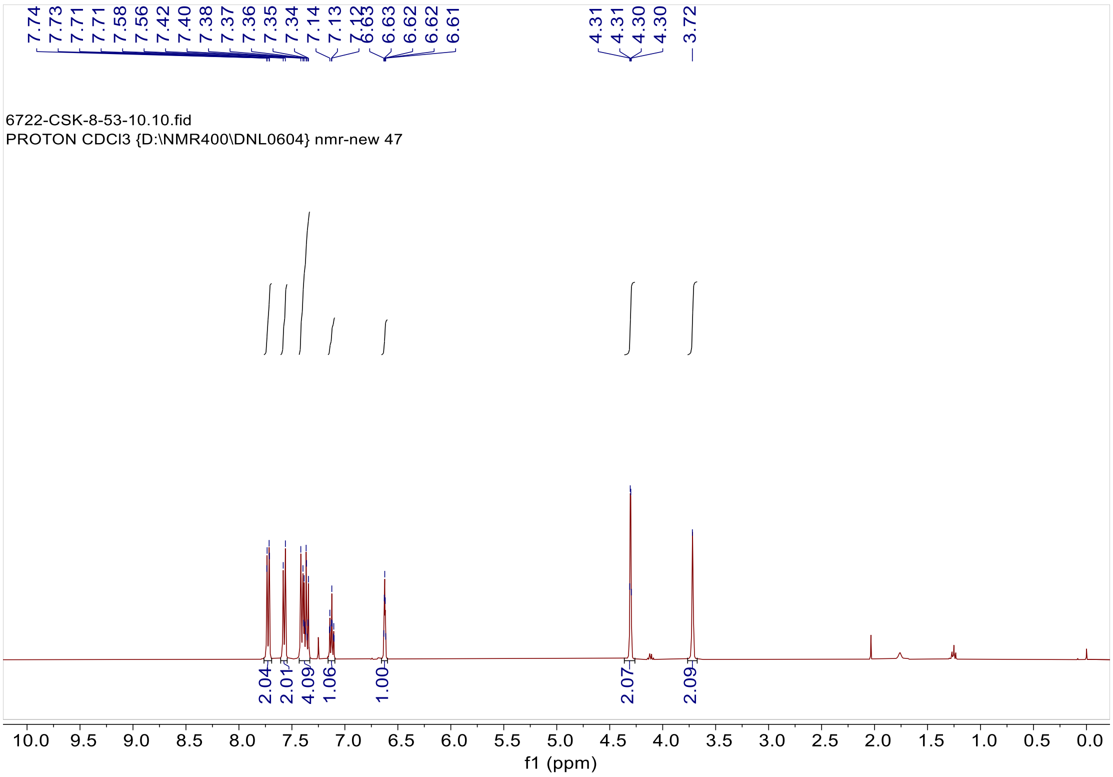

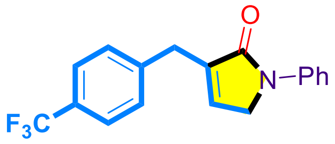

5aa

^{1}H NMR (400 MHz, $CDCl_3$)
^{13}C NMR (100 MHz, $CDCl_3$)

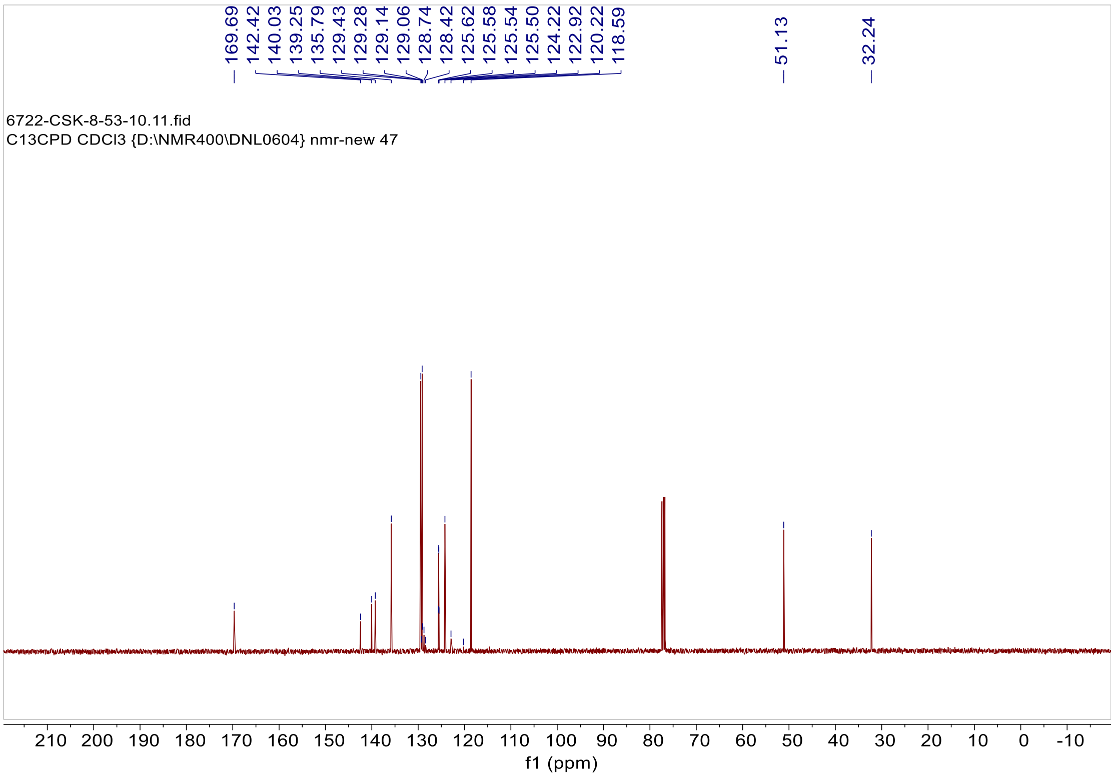

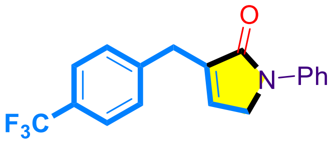

5aa ^{19}F NMR (376 MHz, $CDCl_3$)

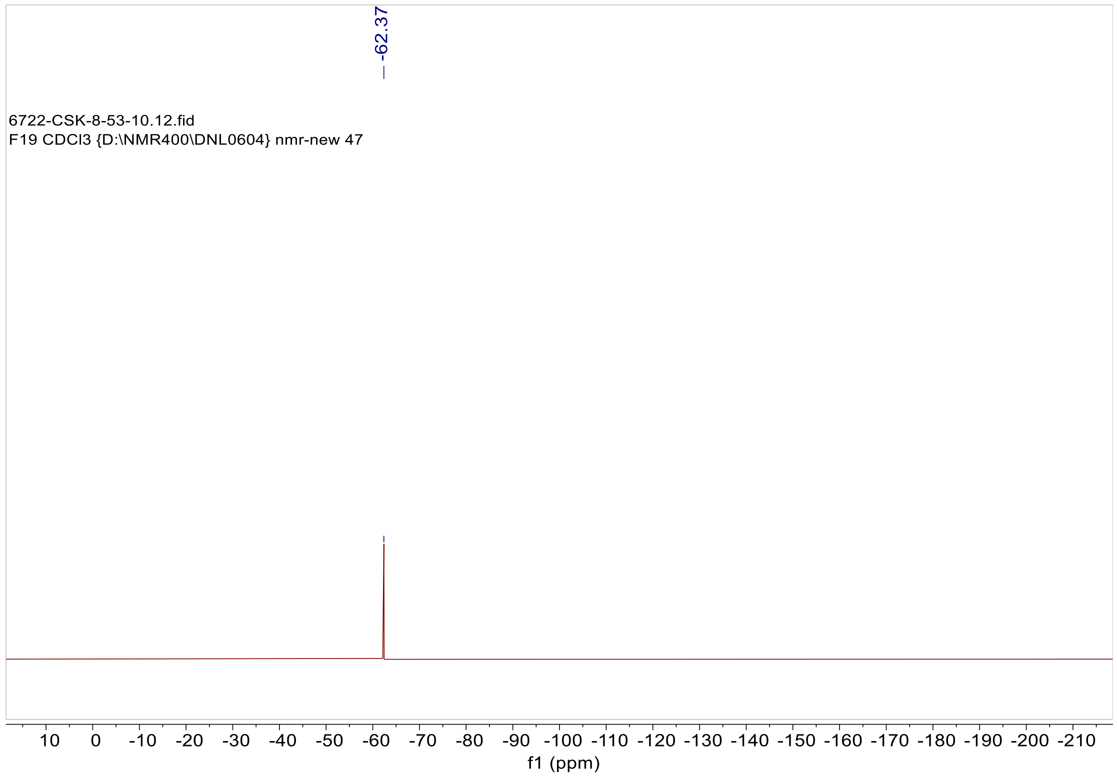

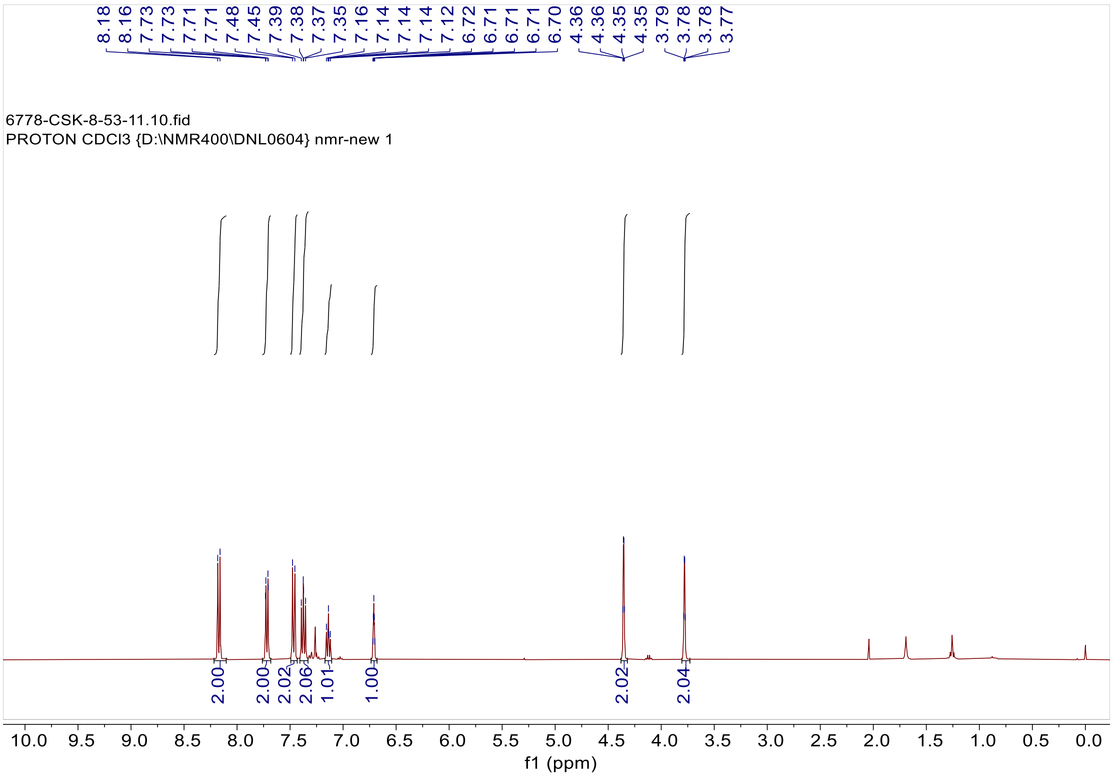

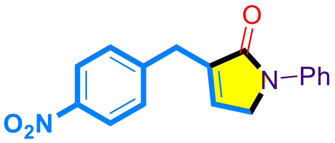

5ab

^{1}H NMR (400 MHz, $CDCl_3$)

^{13}C NMR (100 MHz, $CDCl_3$)

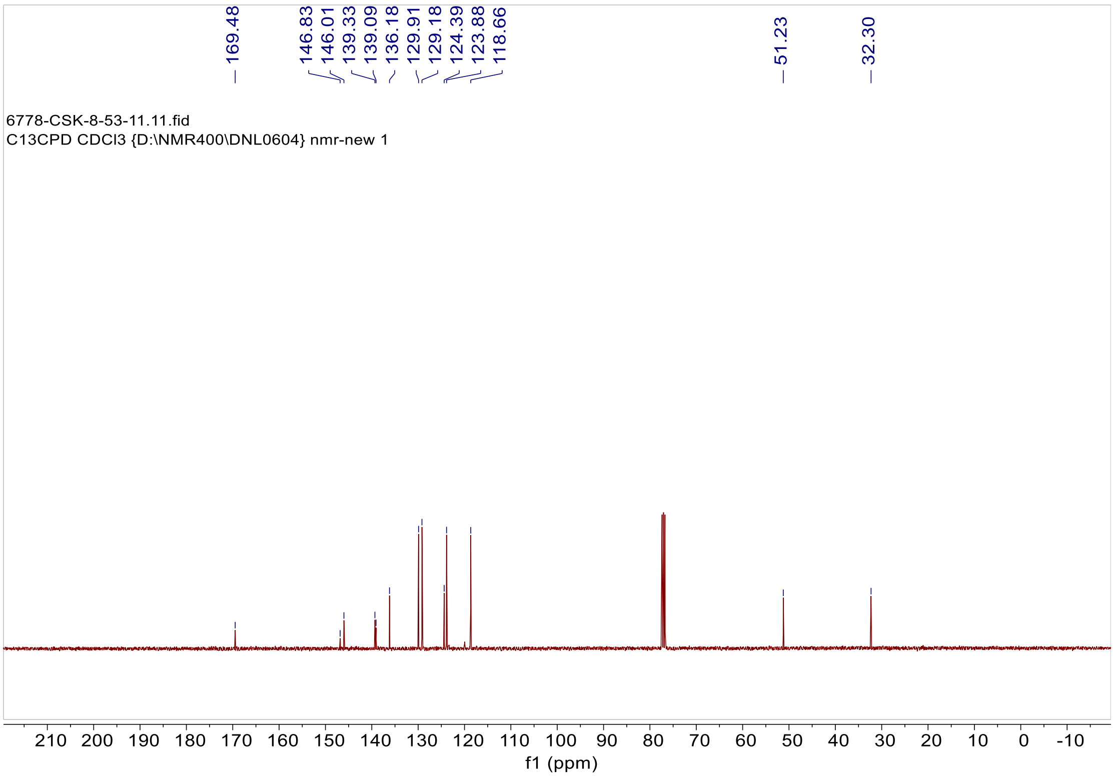

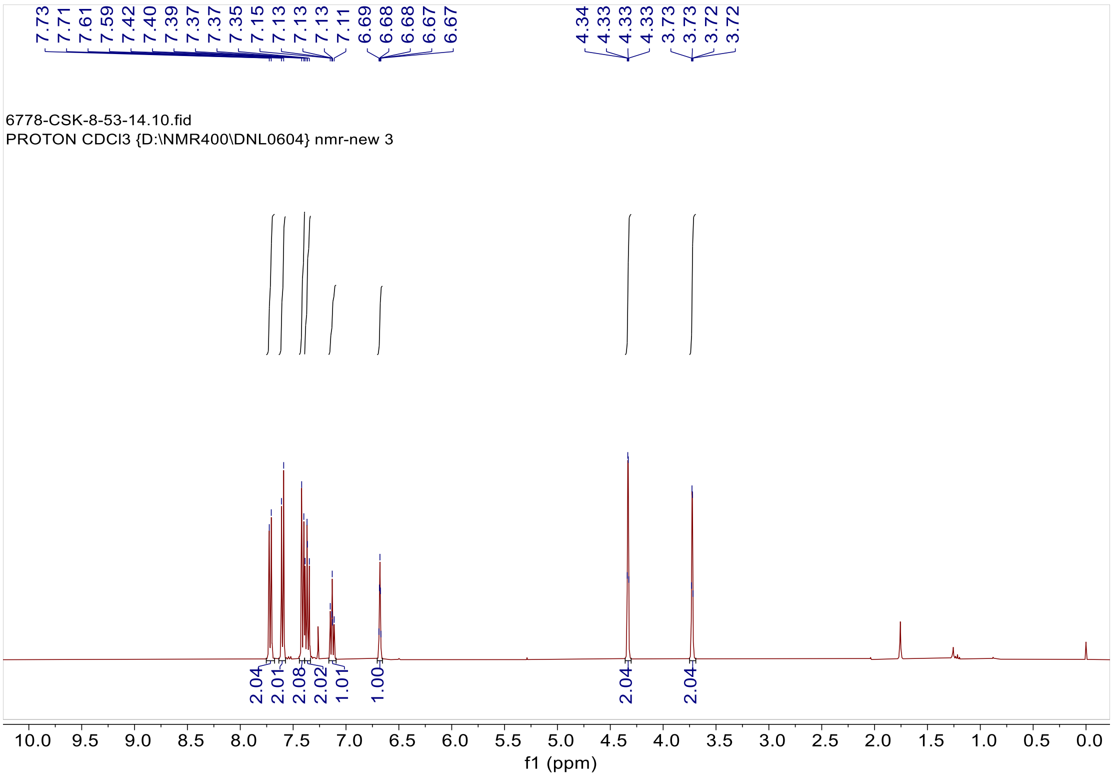

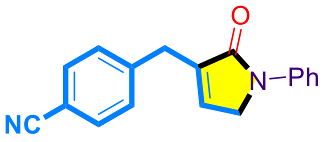

5ac

^{1}H NMR (400 MHz, $CDCl_3$)
^{13}C NMR (100 MHz, $CDCl_3$)

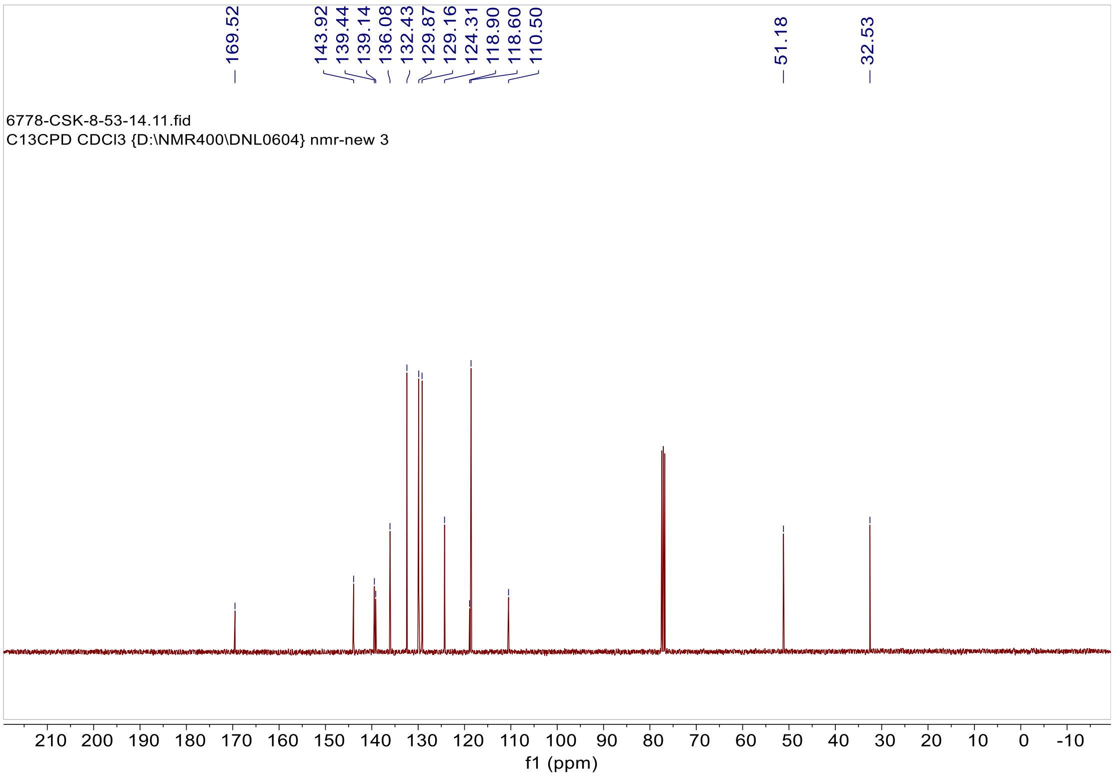

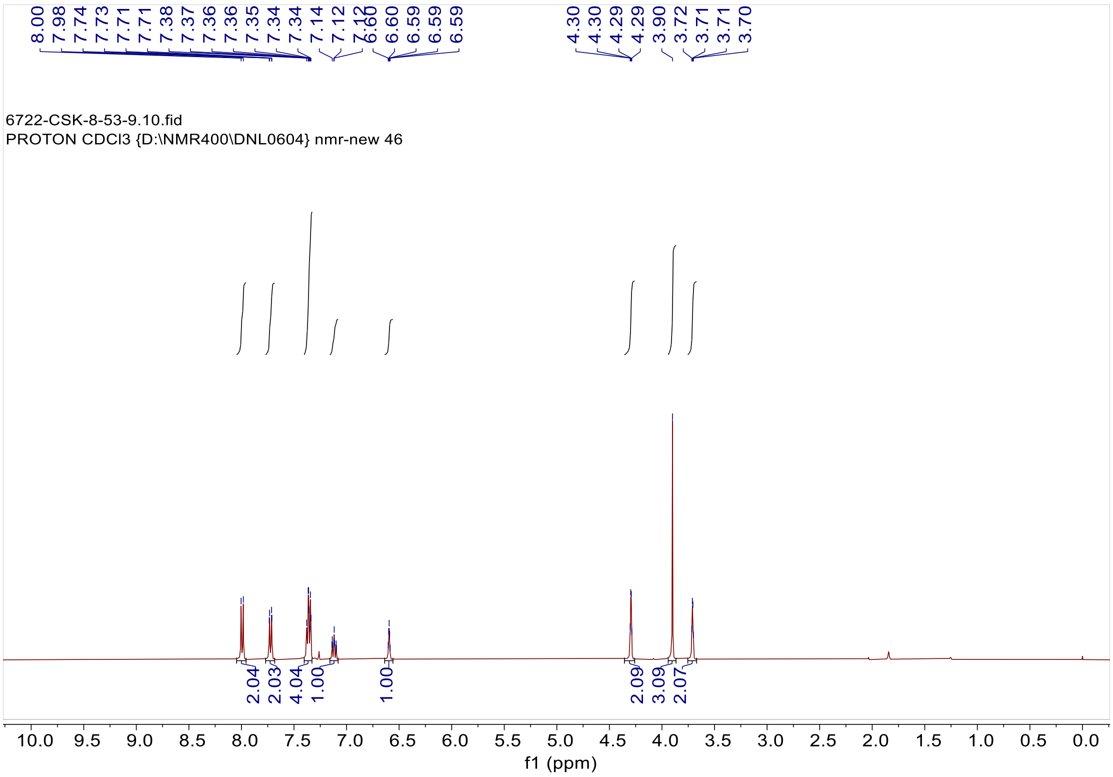

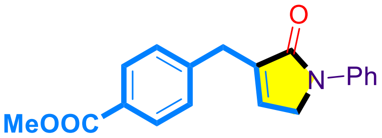

5ad

^{1}H NMR (400 MHz, $CDCl_3$)

^{13}C NMR (100 MHz, $CDCl_3$)

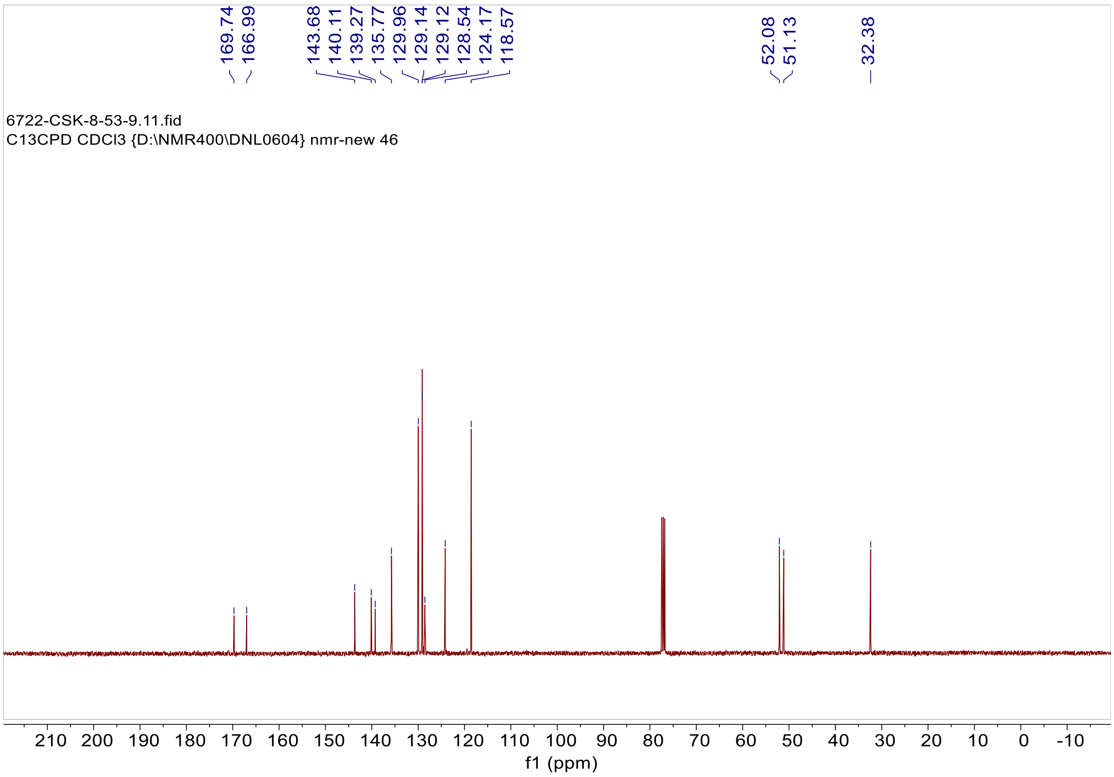

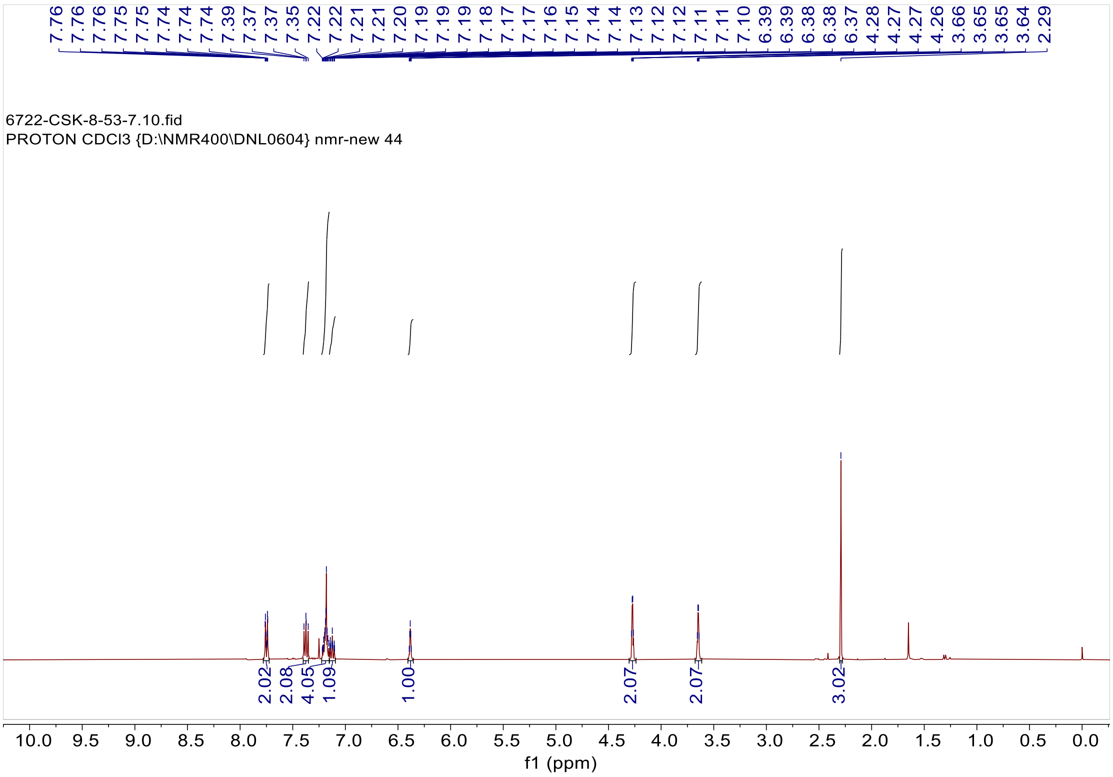

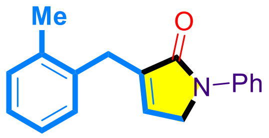

5ae

^{1}H NMR (400 MHz, $CDCl_3$)

^{13}C NMR (100 MHz, $CDCl_3$)

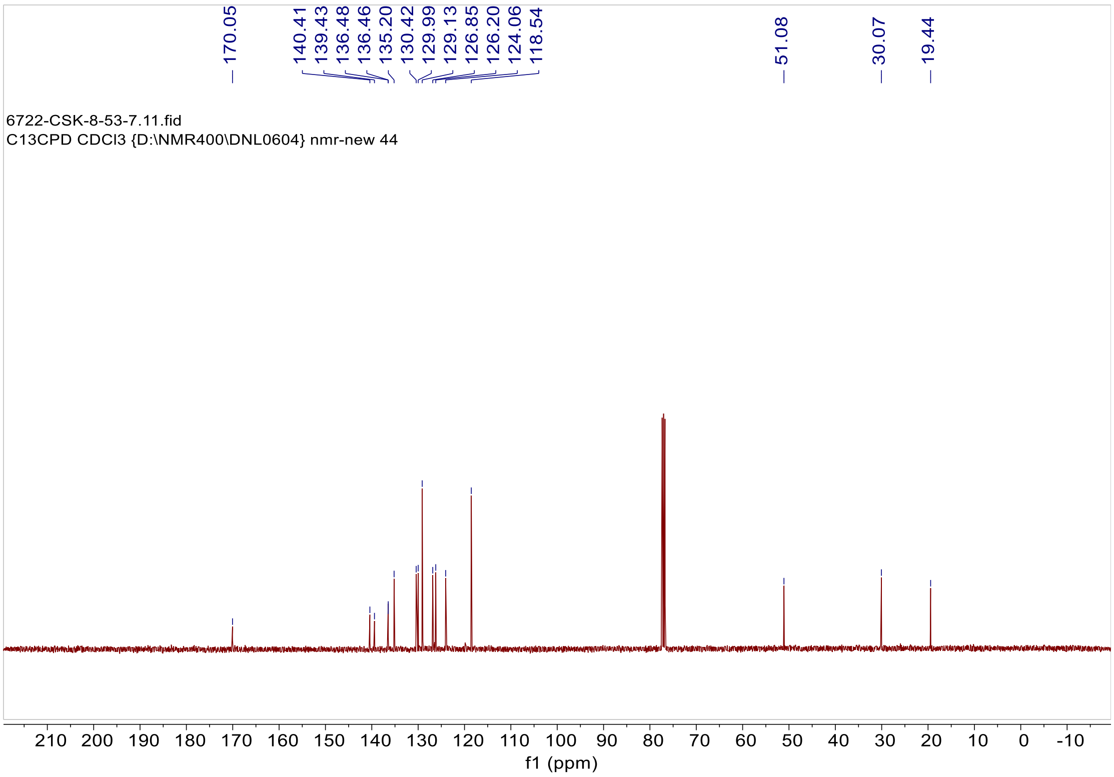

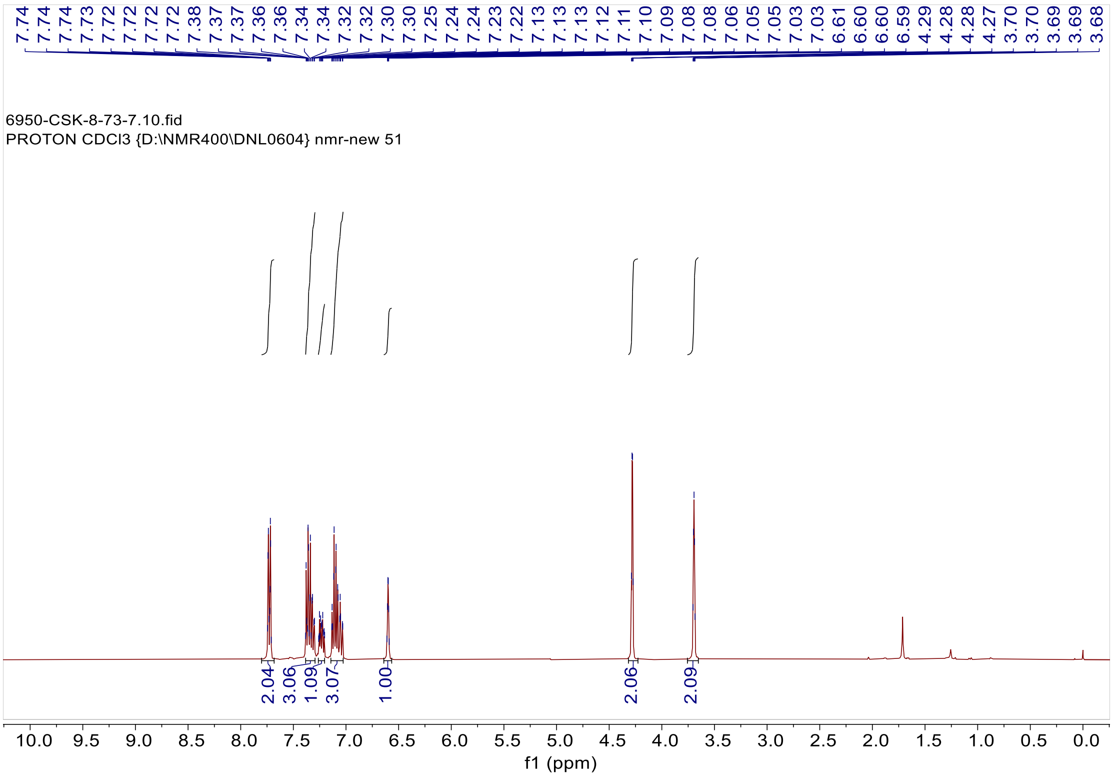

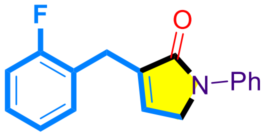

5af

^{1}H NMR (400 MHz, $CDCl_3$)
^{13}C NMR (100 MHz, $CDCl_3$)

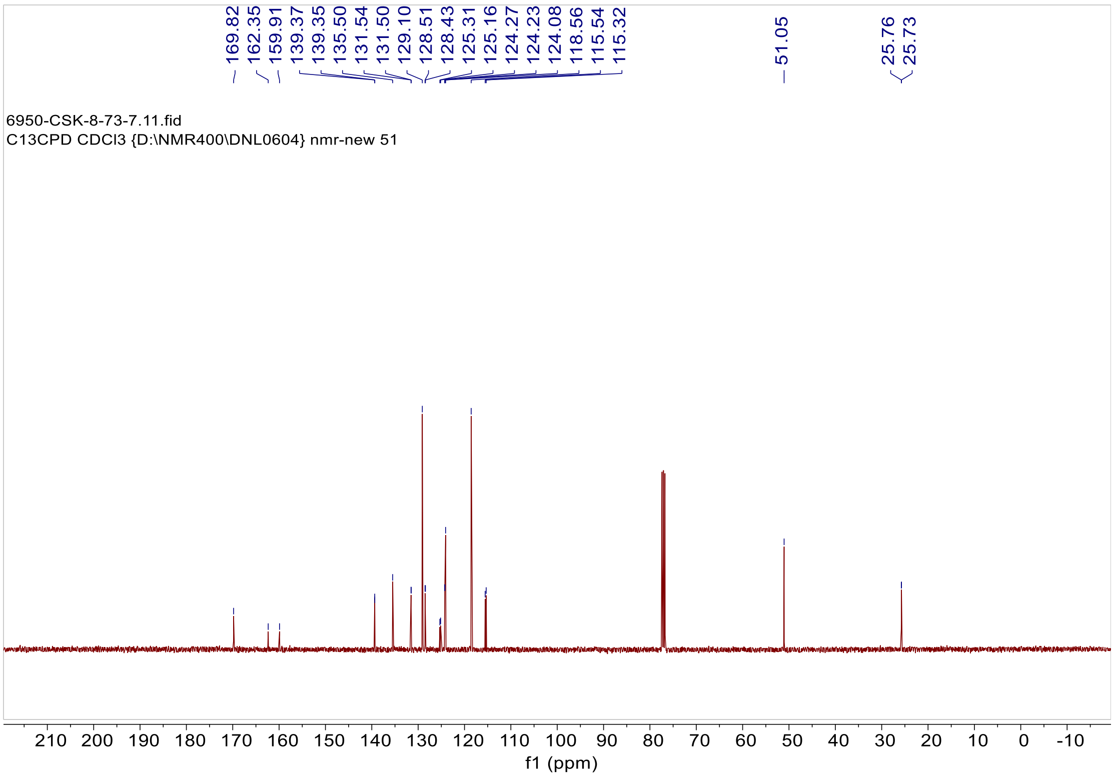

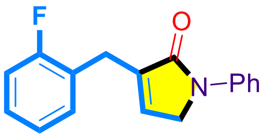

5af ^{19}F NMR (376 MHz, $CDCl_3$)

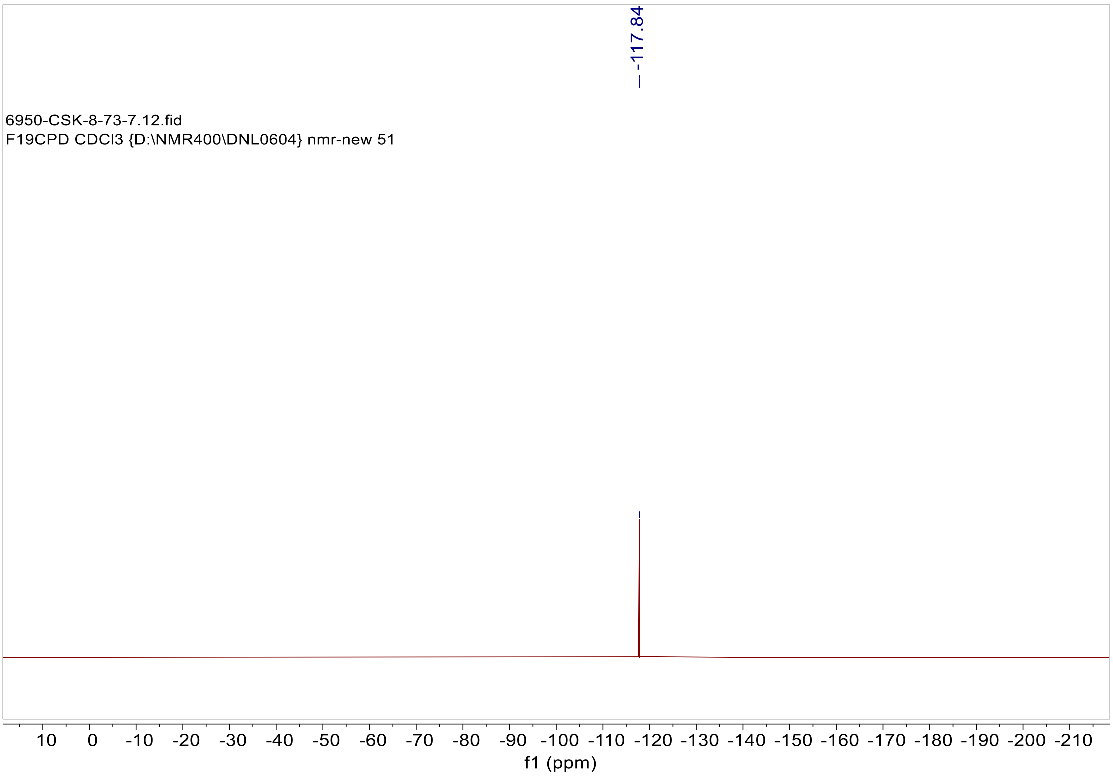

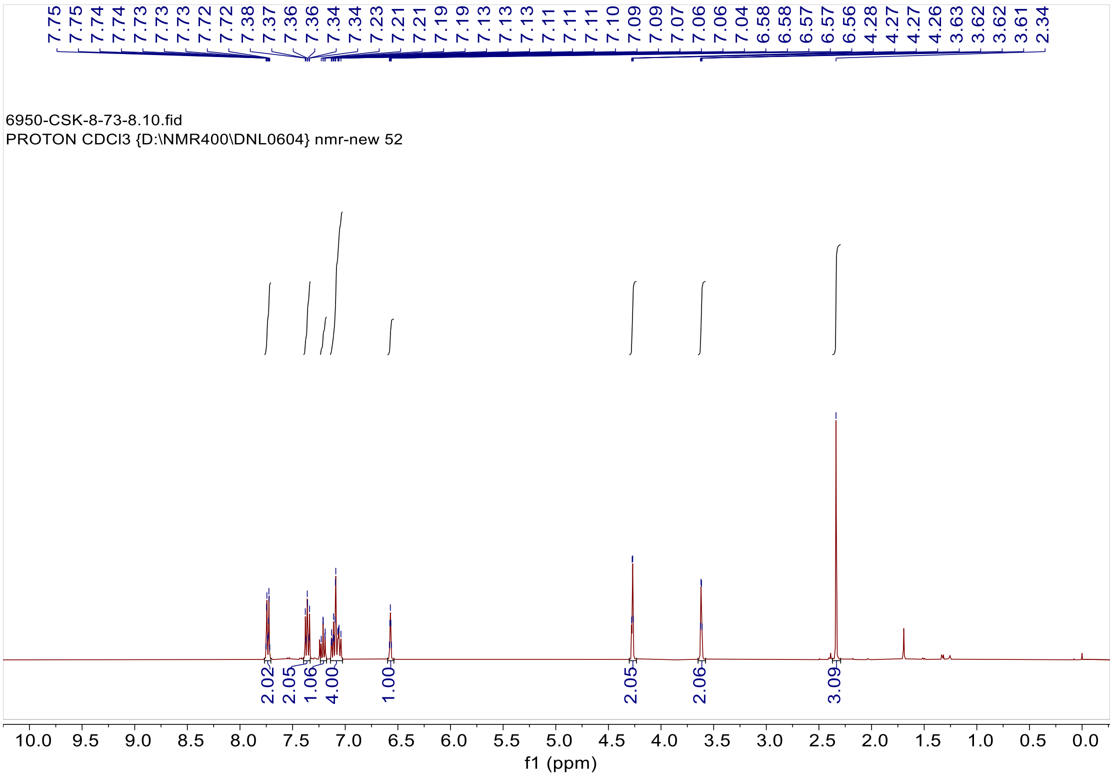

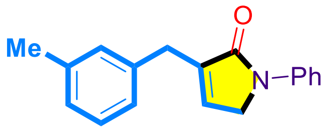

5ag

^{1}H NMR (400 MHz, $CDCl_3$)
^{13}C NMR (100 MHz, $CDCl_3$)

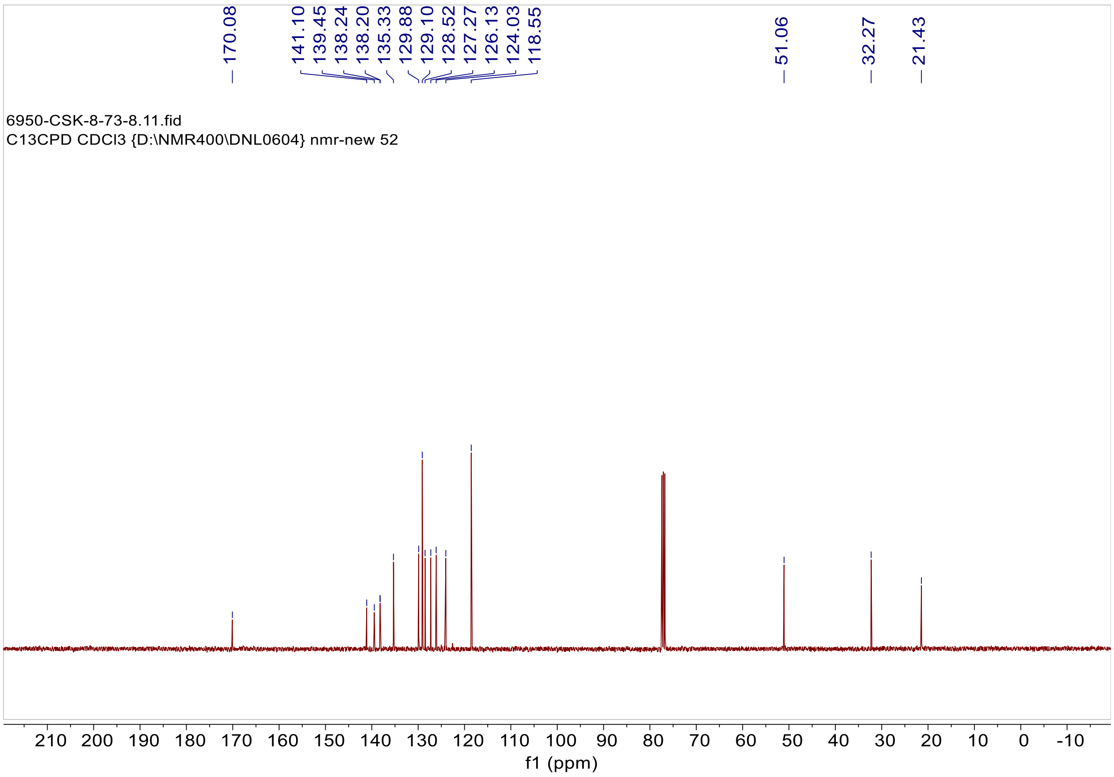

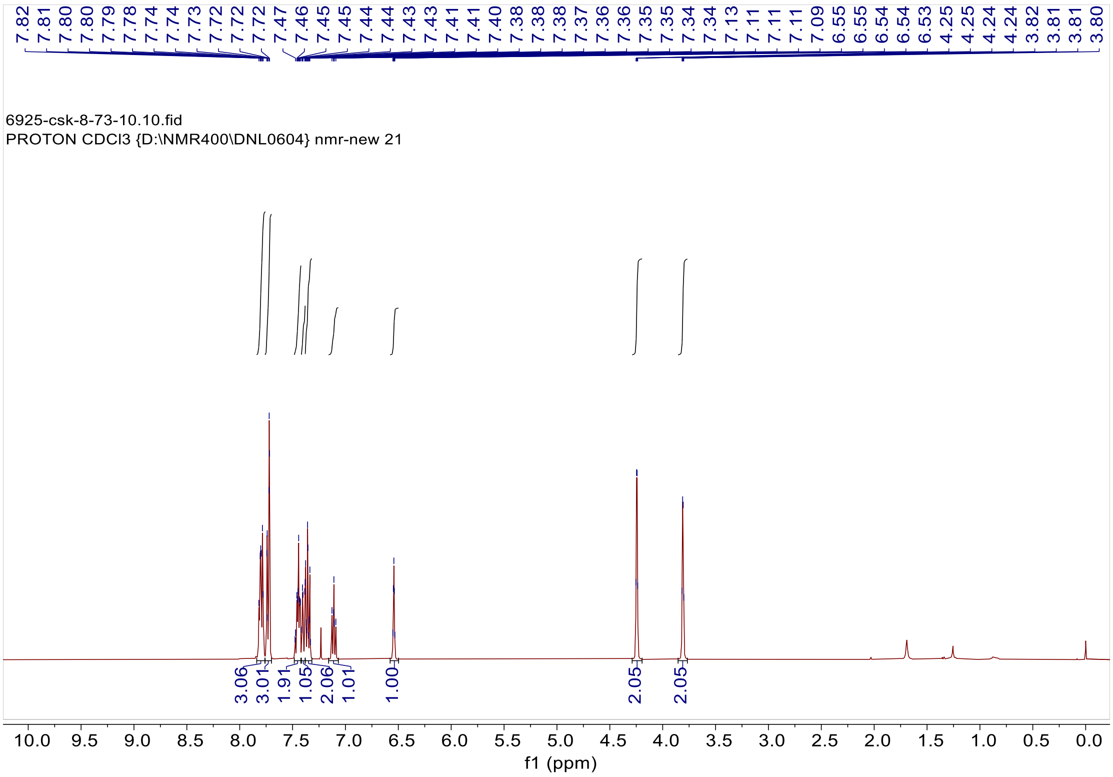

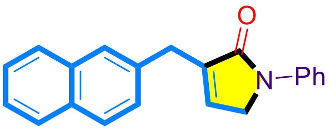

5ah

^{1}H NMR (400 MHz, $CDCl_3$)
^{13}C NMR (100 MHz, $CDCl_3$)

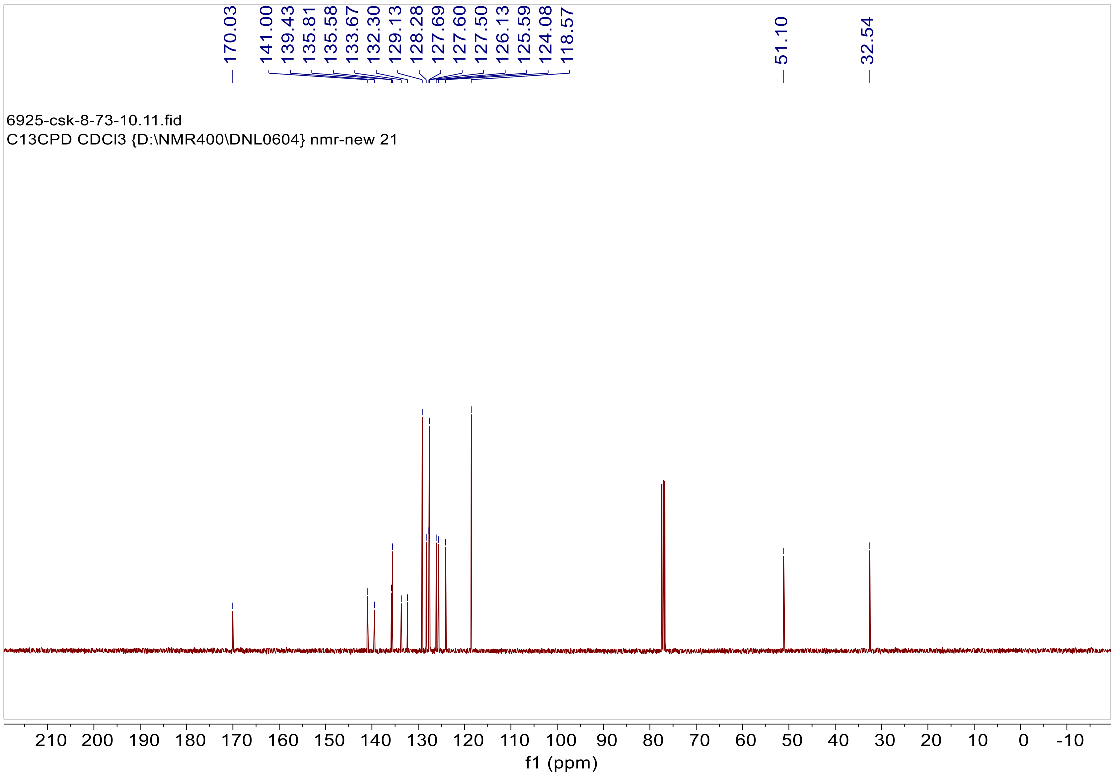

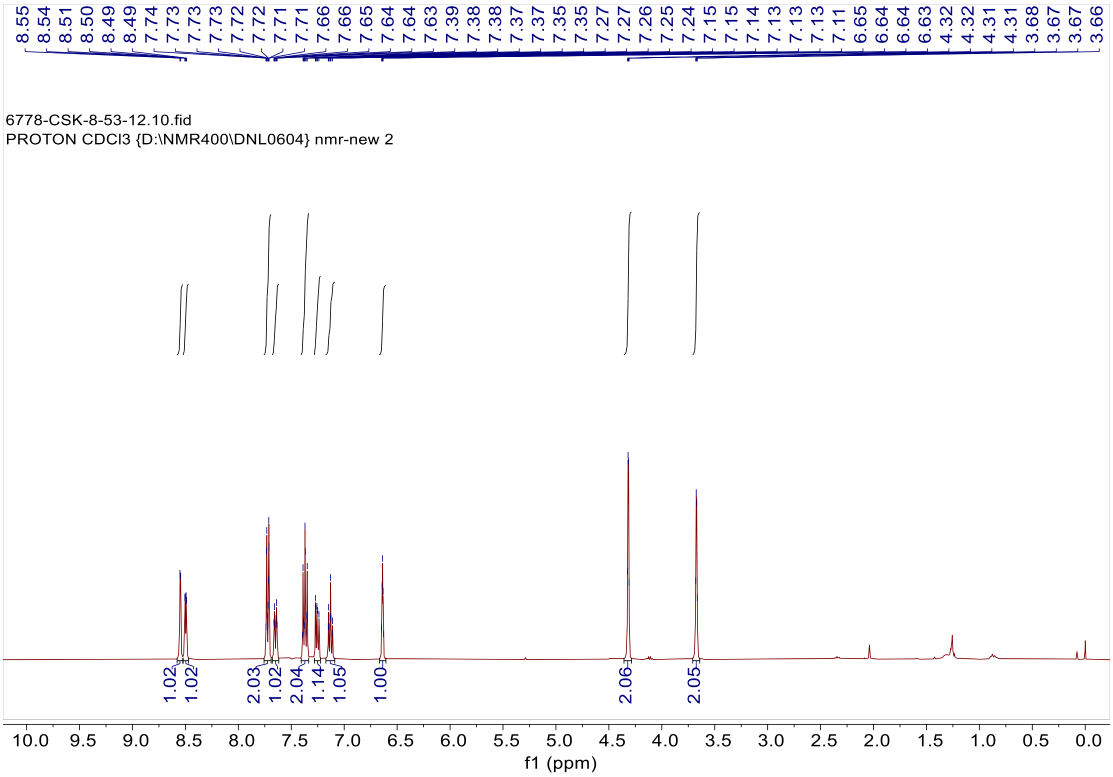

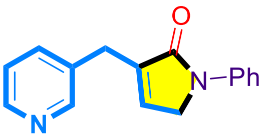

5ai

^{1}H NMR (400 MHz, $CDCl_3$)
^{13}C NMR (100 MHz, $CDCl_3$)

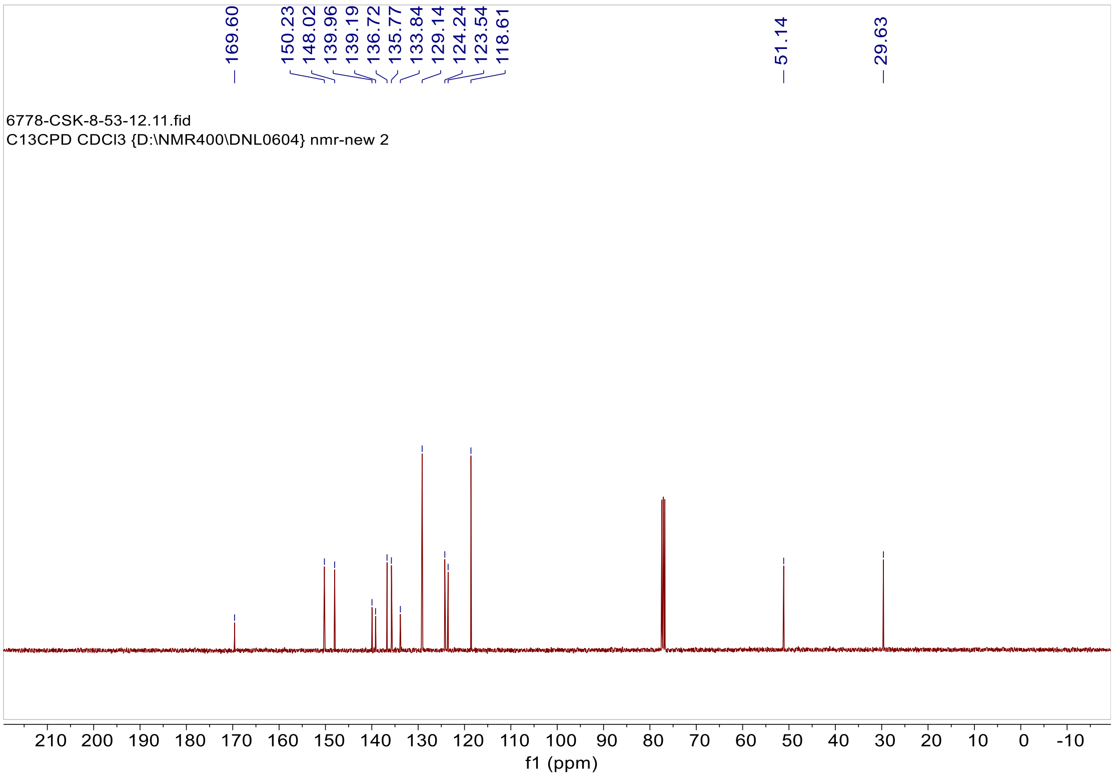

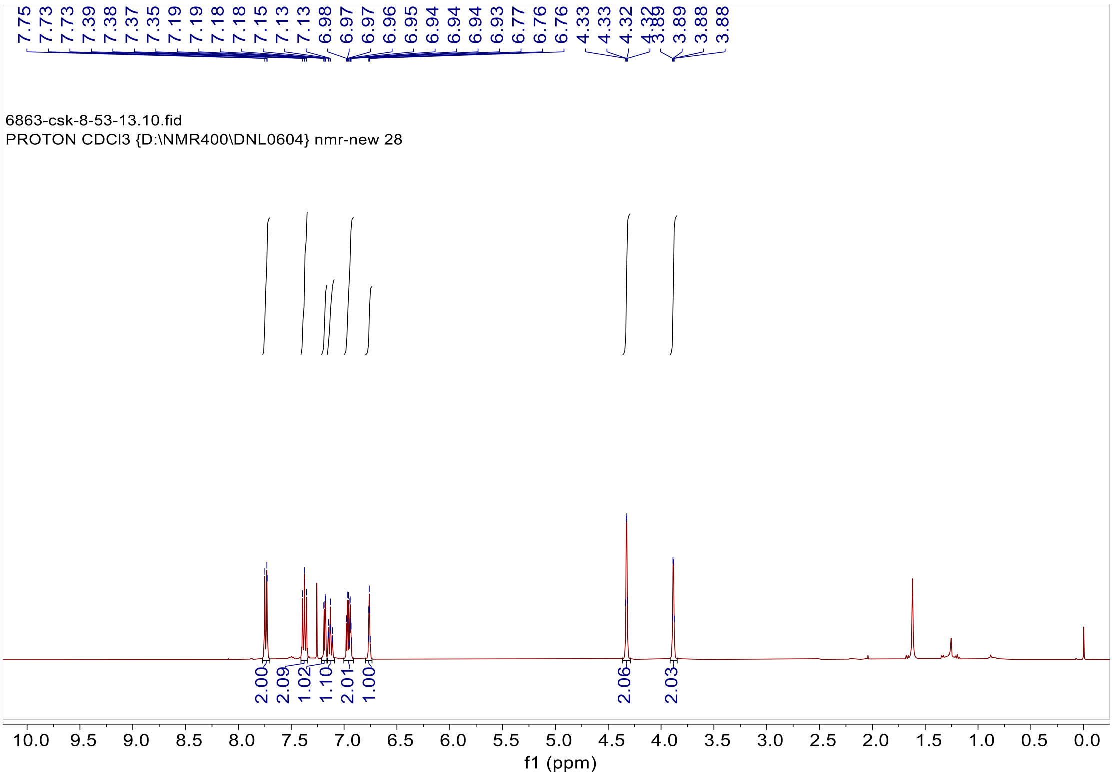

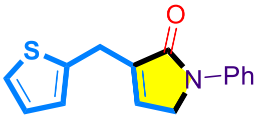

5aj

^{1}H NMR (400 MHz, $CDCl_3$)

^{13}C NMR (100 MHz, $CDCl_3$)

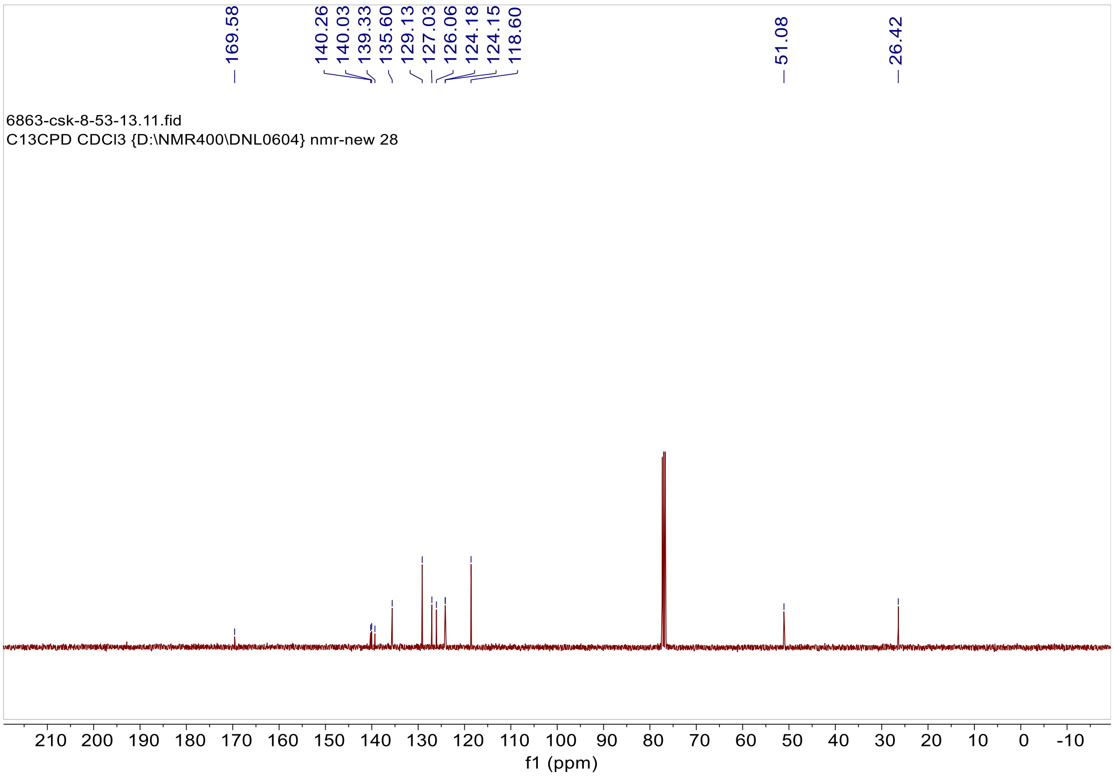

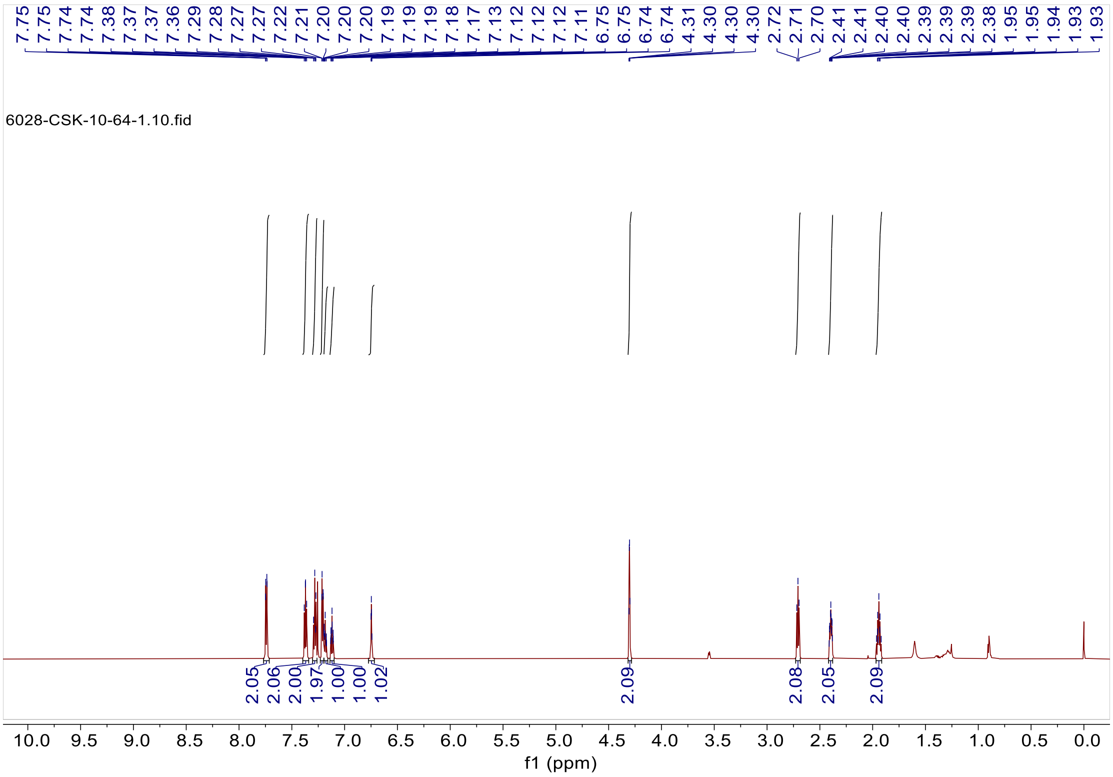

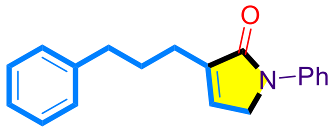

5ak

^{1}H NMR (700 MHz, $CDCl_3$)
^{13}C NMR (176 MHz, $CDCl_3$)

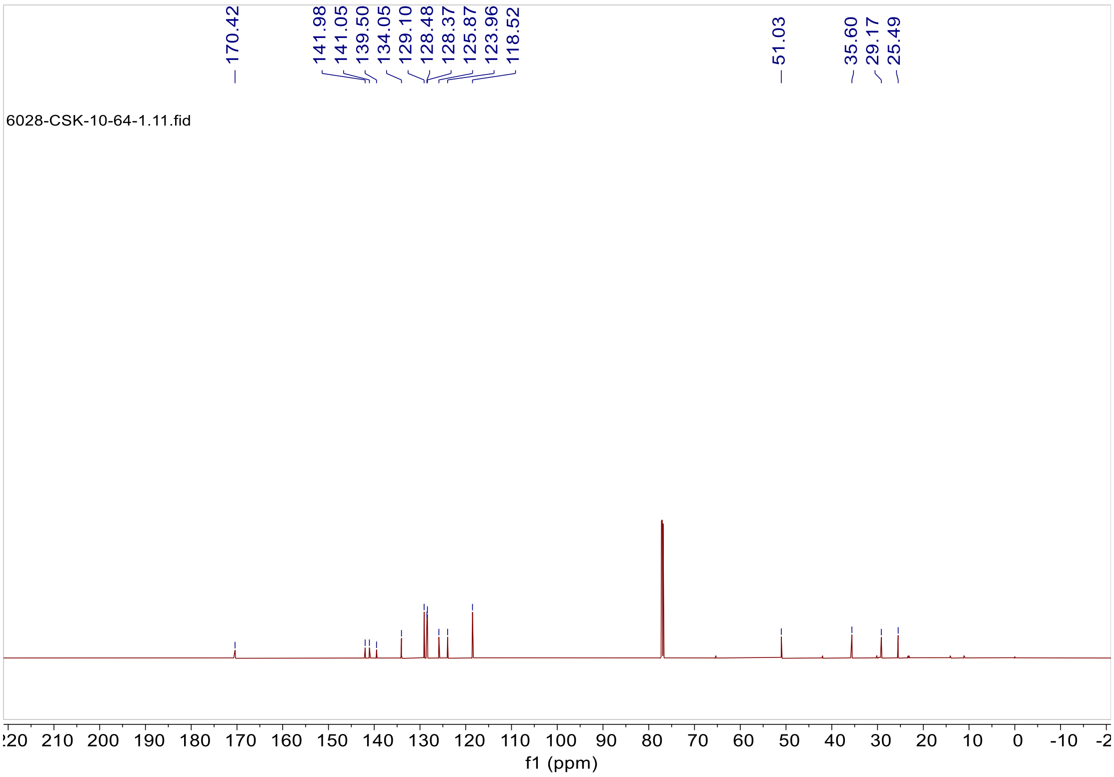

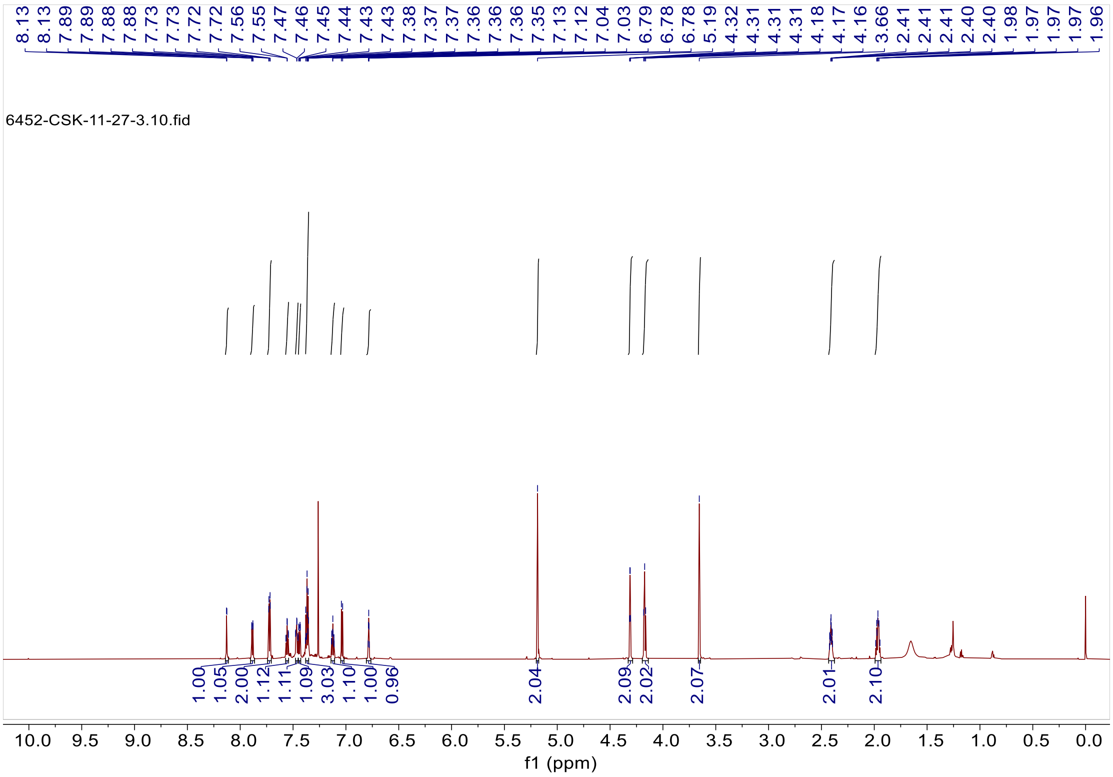

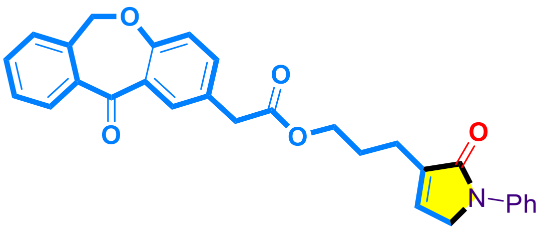

5am

^{1}H NMR (700 MHz, $CDCl_3$)

^{13}C NMR (176 MHz, $CDCl_3$)

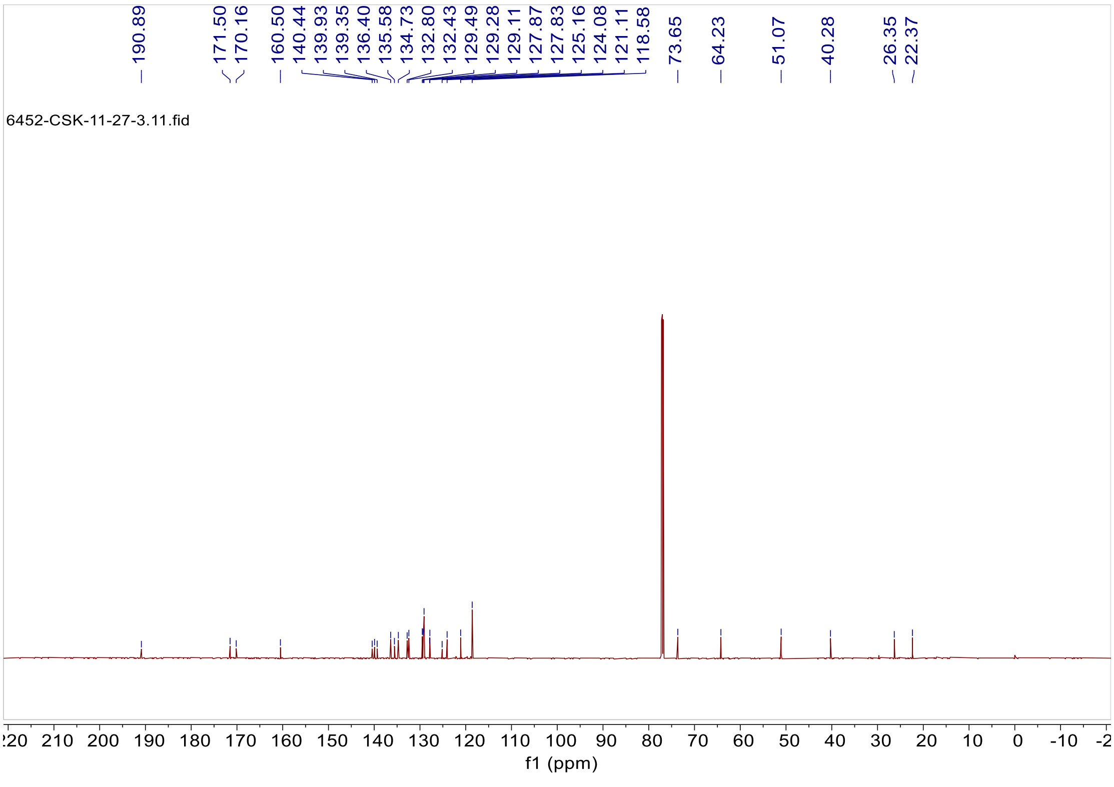

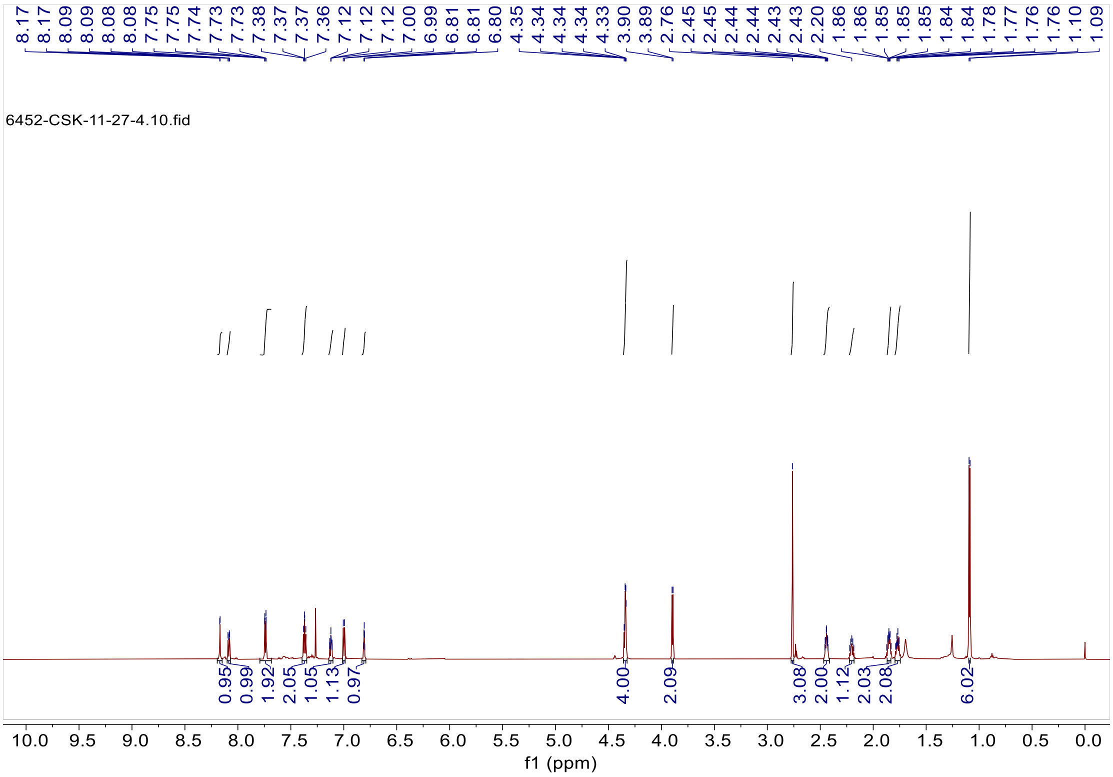

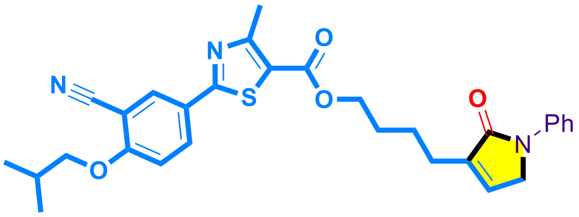

5an

^{1}H NMR (700 MHz, $CDCl_3$)
^{13}C NMR (176 MHz, $CDCl_3$)

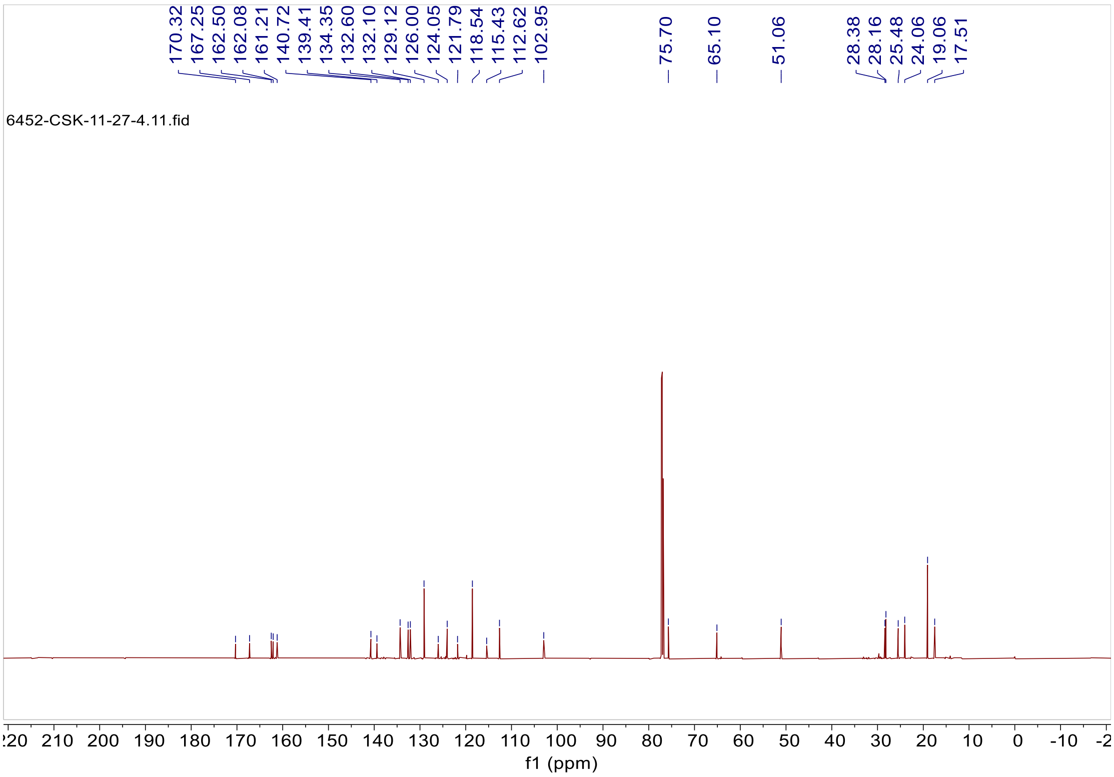

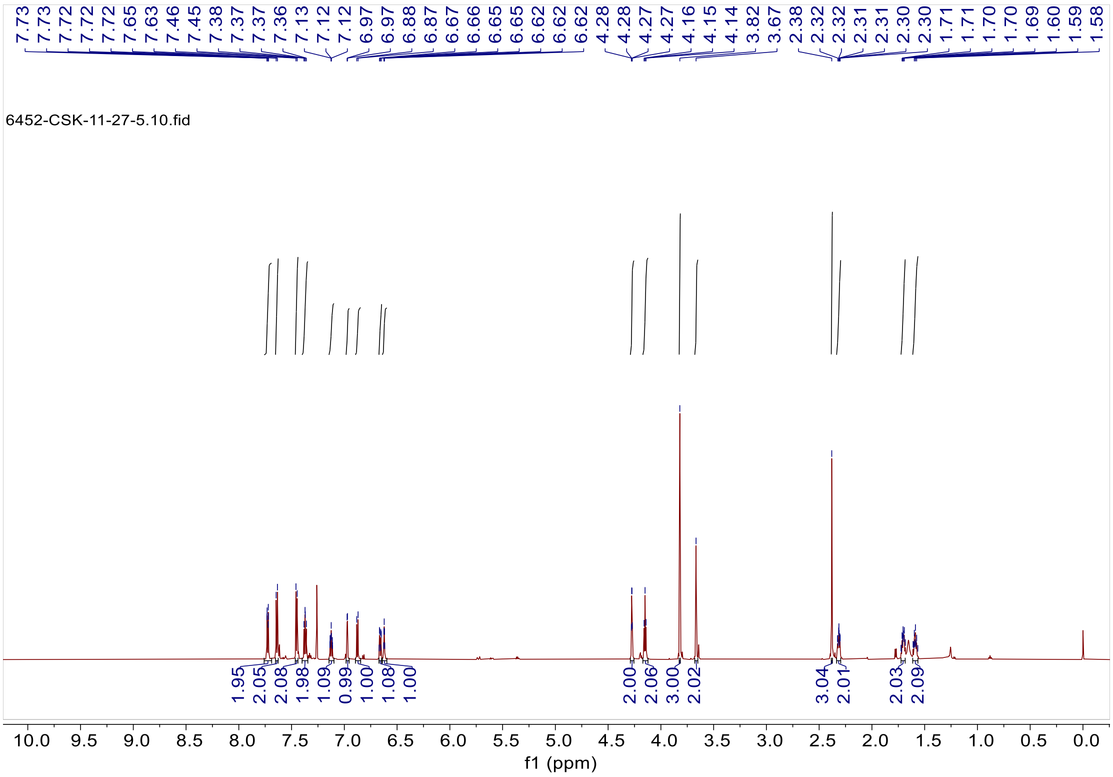

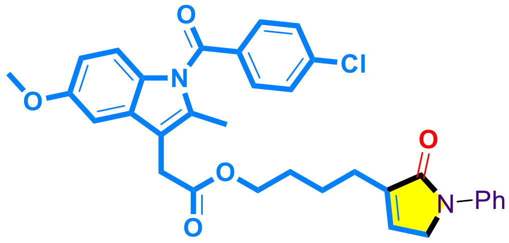

5ao

^{1}H NMR (700 MHz, $CDCl_3$)
^{13}C NMR (176 MHz, $CDCl_3$)

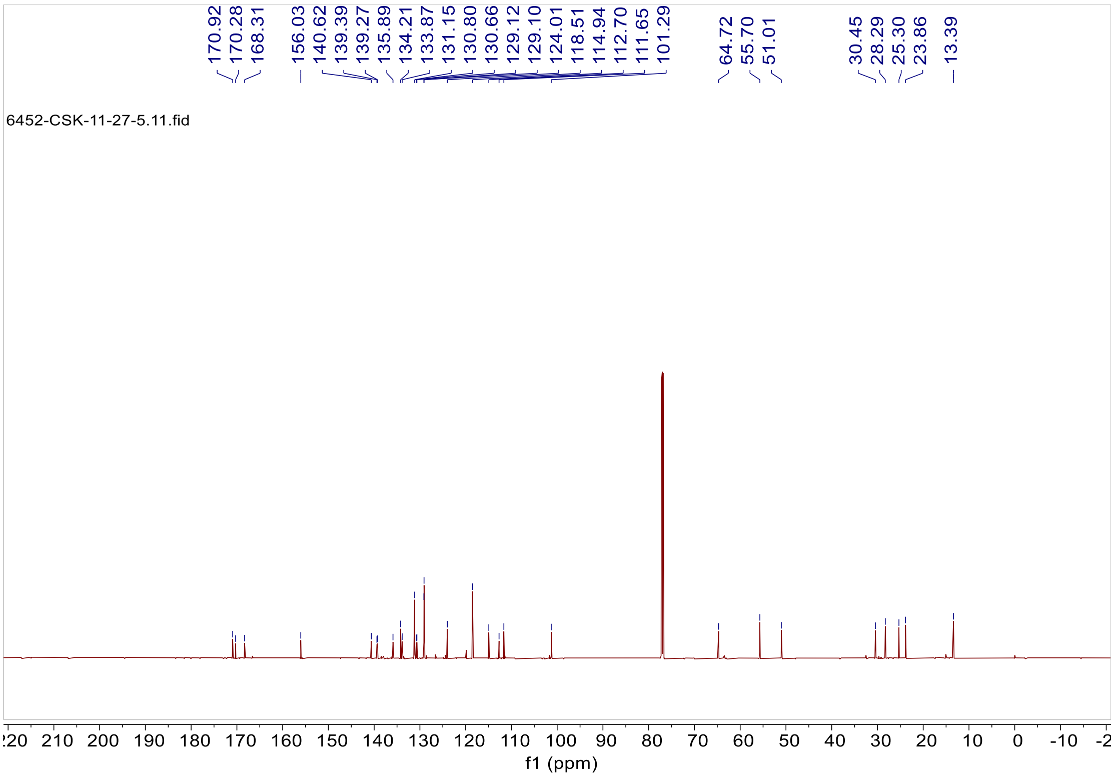

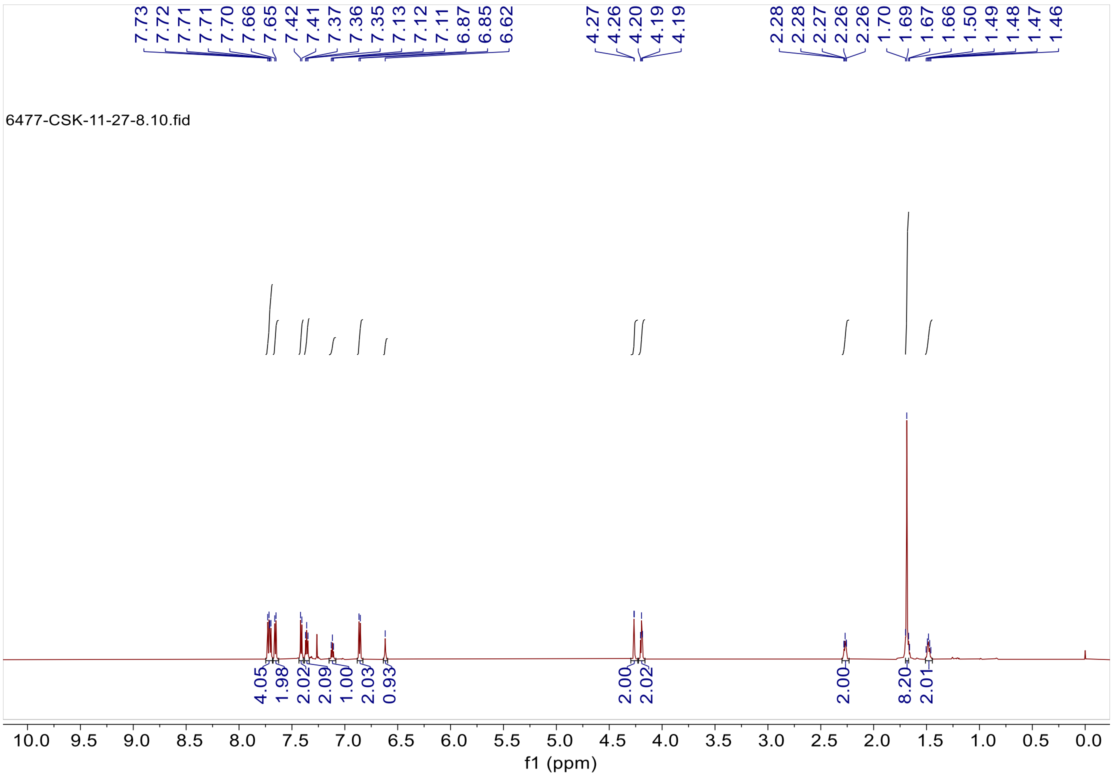

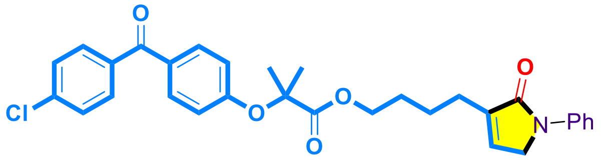

5ap

^{1}H NMR (700 MHz, $CDCl_3$)
^{13}C NMR (176 MHz, $CDCl_3$)

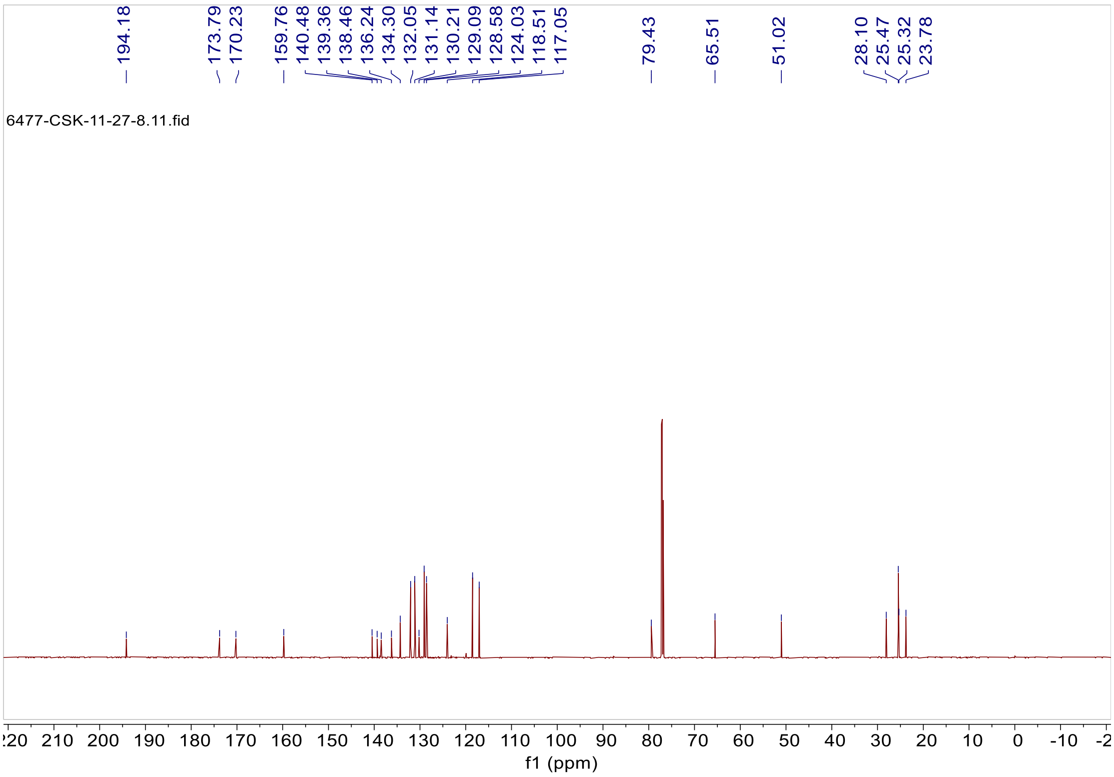

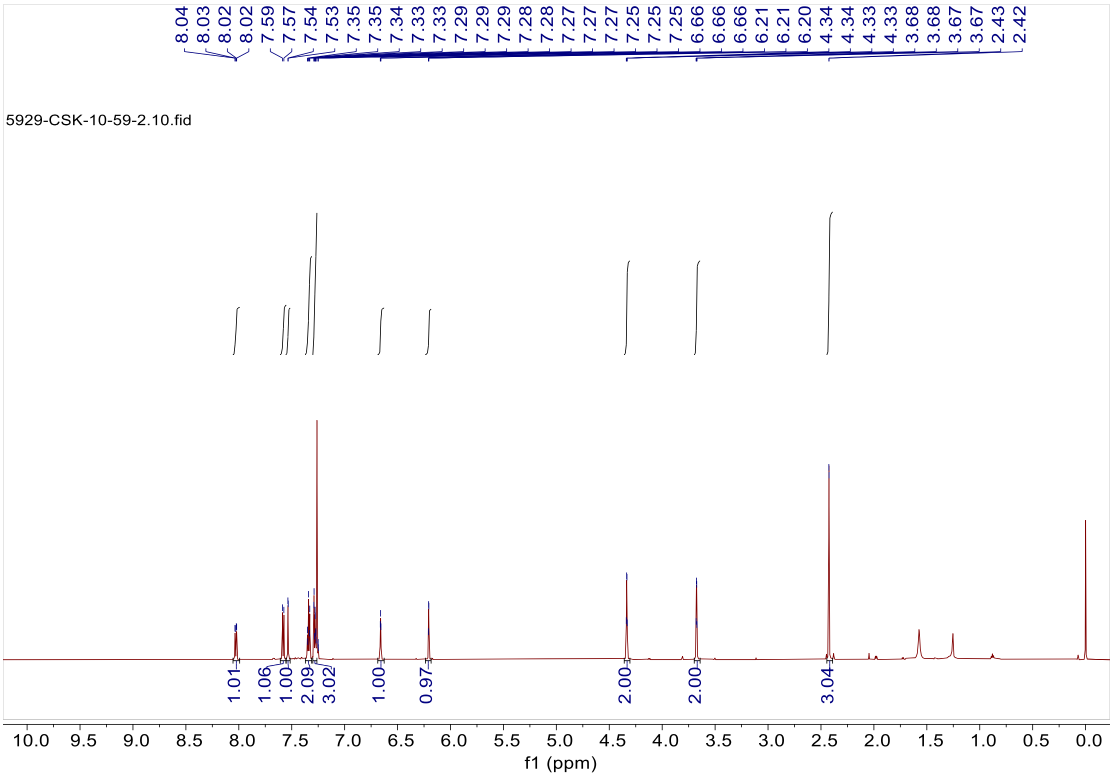

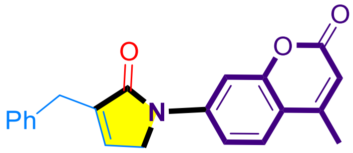

5ar

^{1}H NMR (700 MHz, $CDCl_3$)

^{13}C NMR (176 MHz, $CDCl_3$)

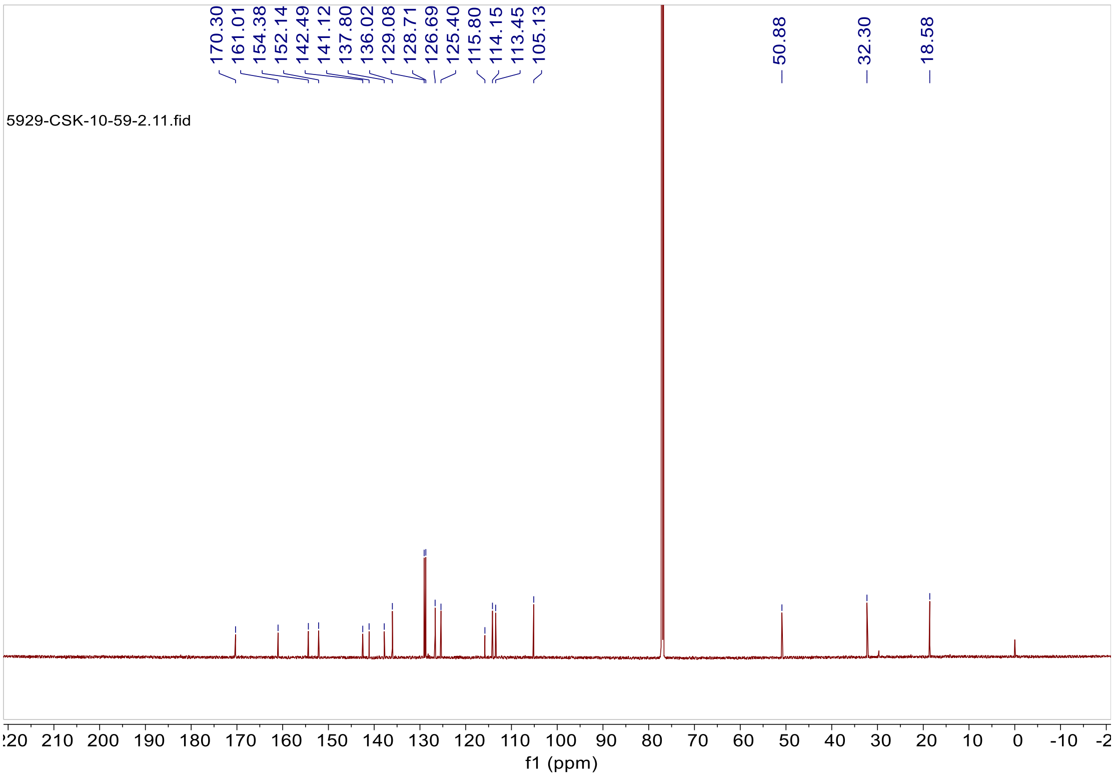

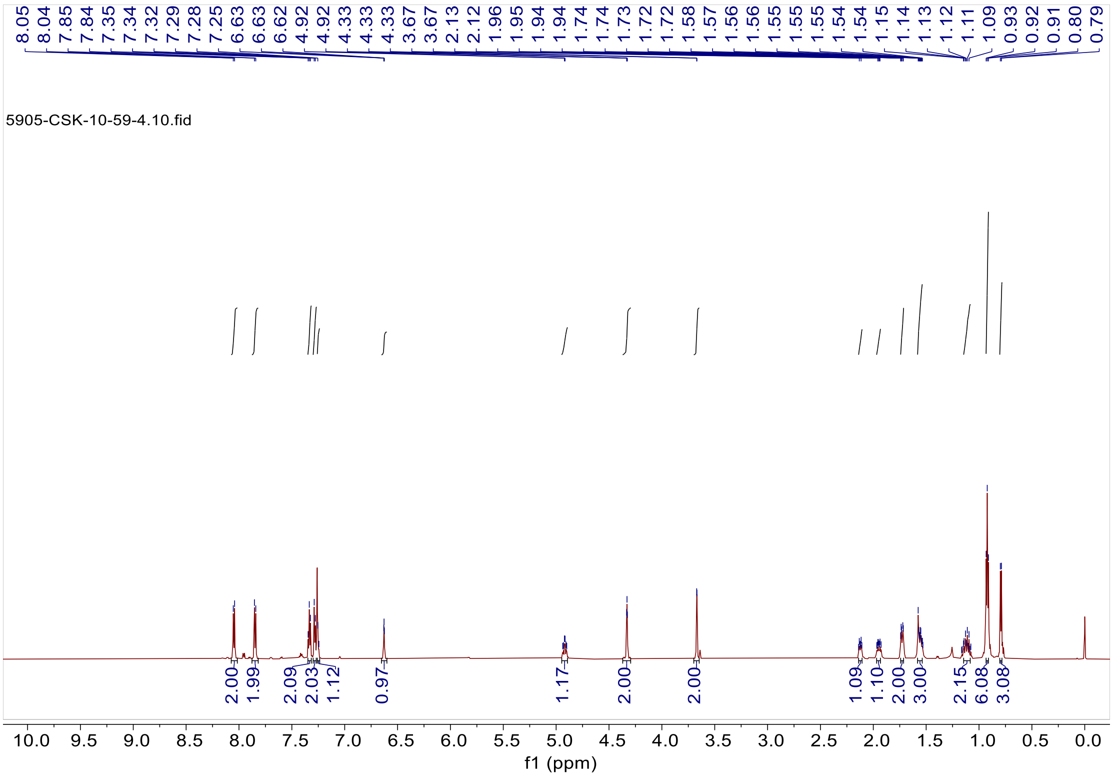

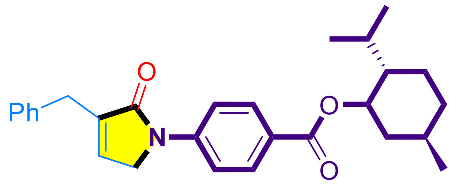

5as

^{1}H NMR (700 MHz, $CDCl_3$)
^{13}C NMR (176 MHz, $CDCl_3$)

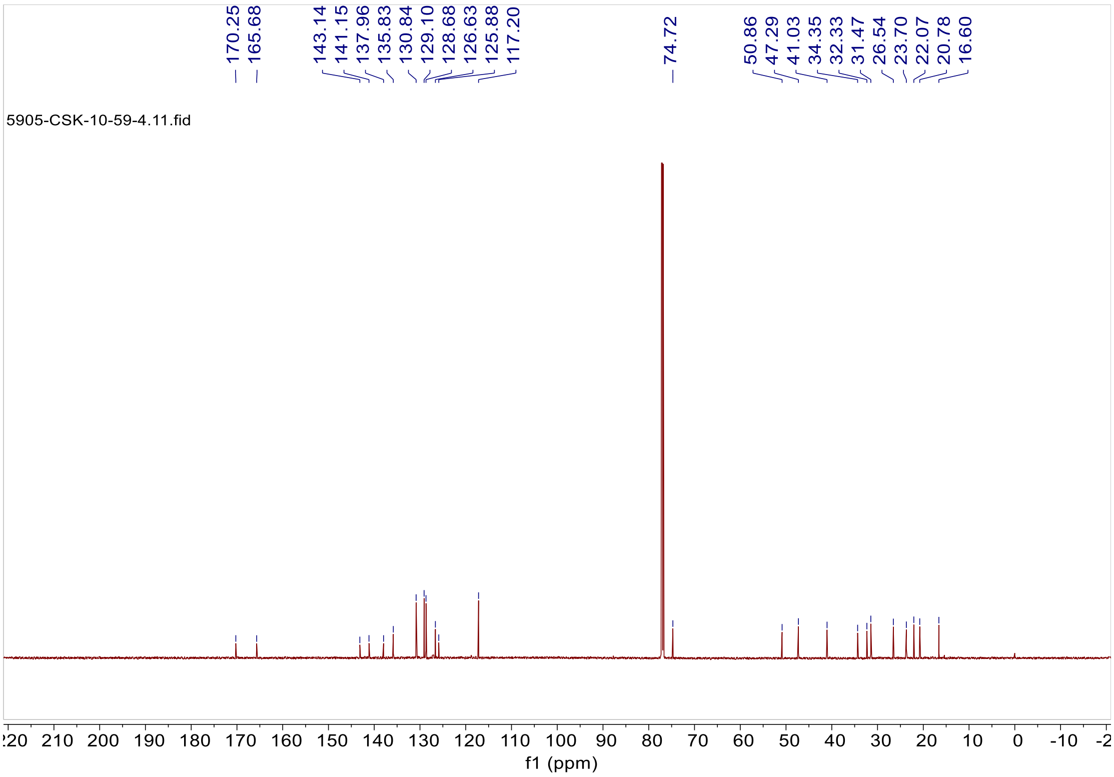

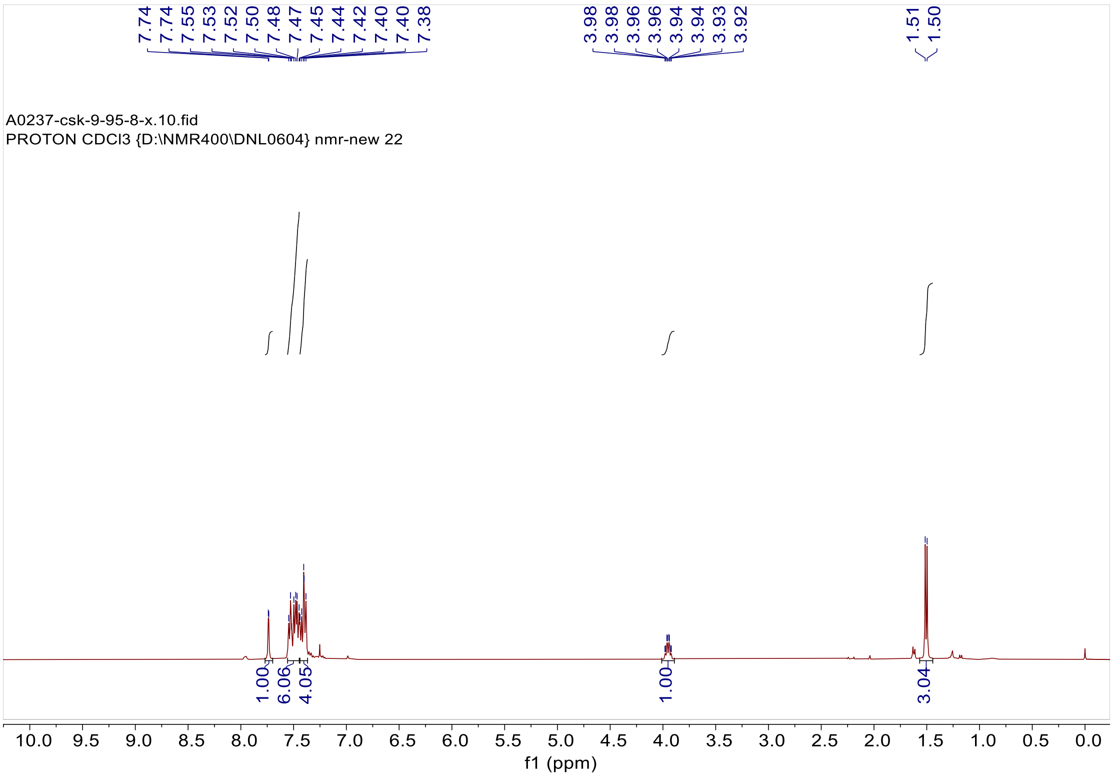

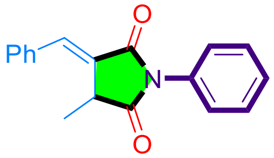

6a

^{1}H NMR (400 MHz, $CDCl_3$)

^{13}C NMR (100 MHz, $CDCl_3$)

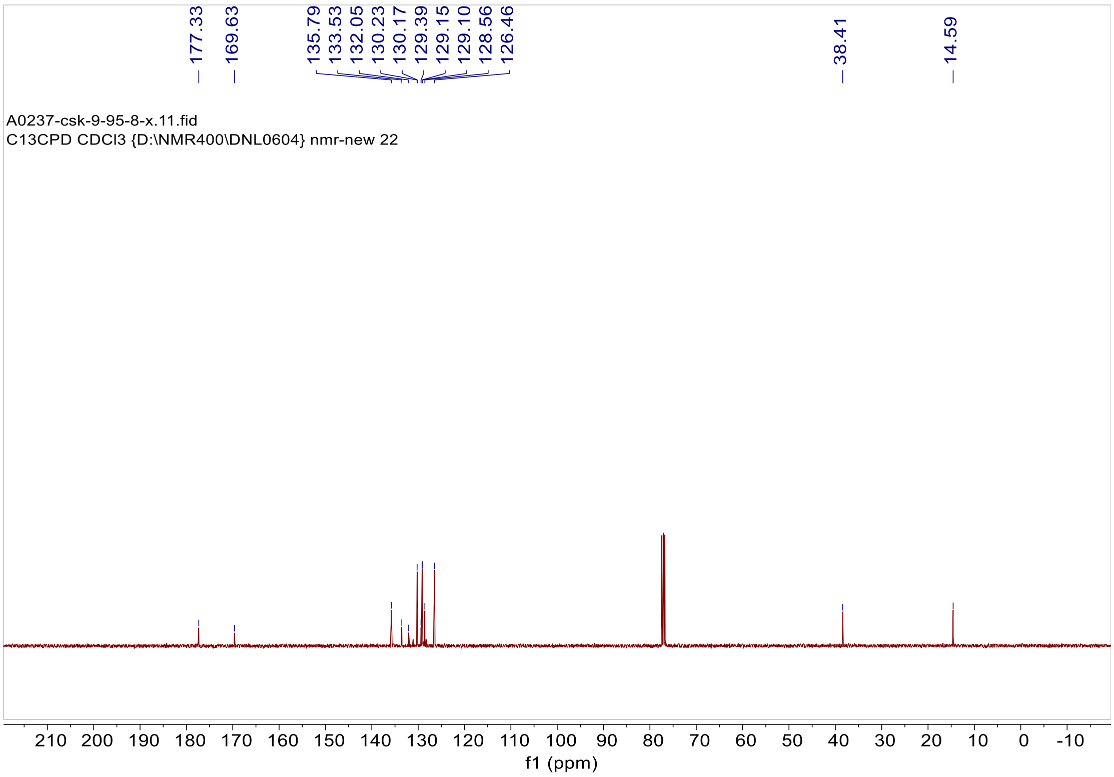

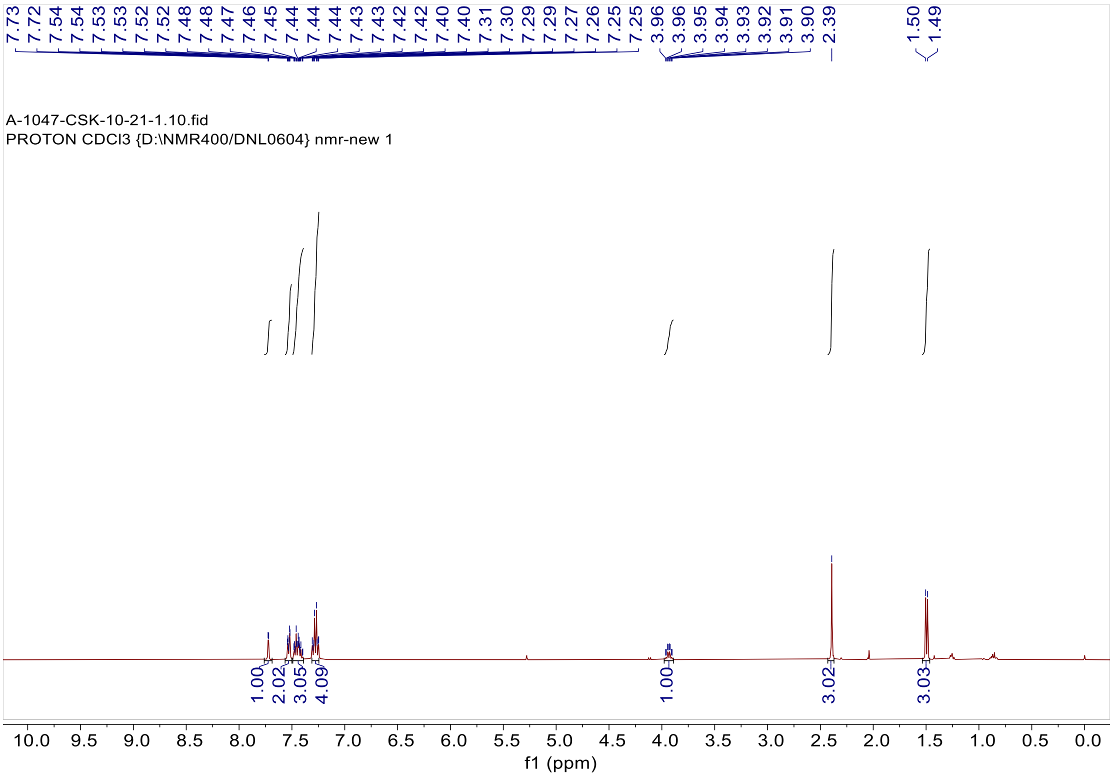

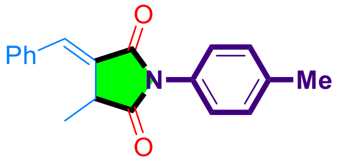

6b

^{1}H NMR (400 MHz, $CDCl_3$)
^{13}C NMR (100 MHz, $CDCl_3$)

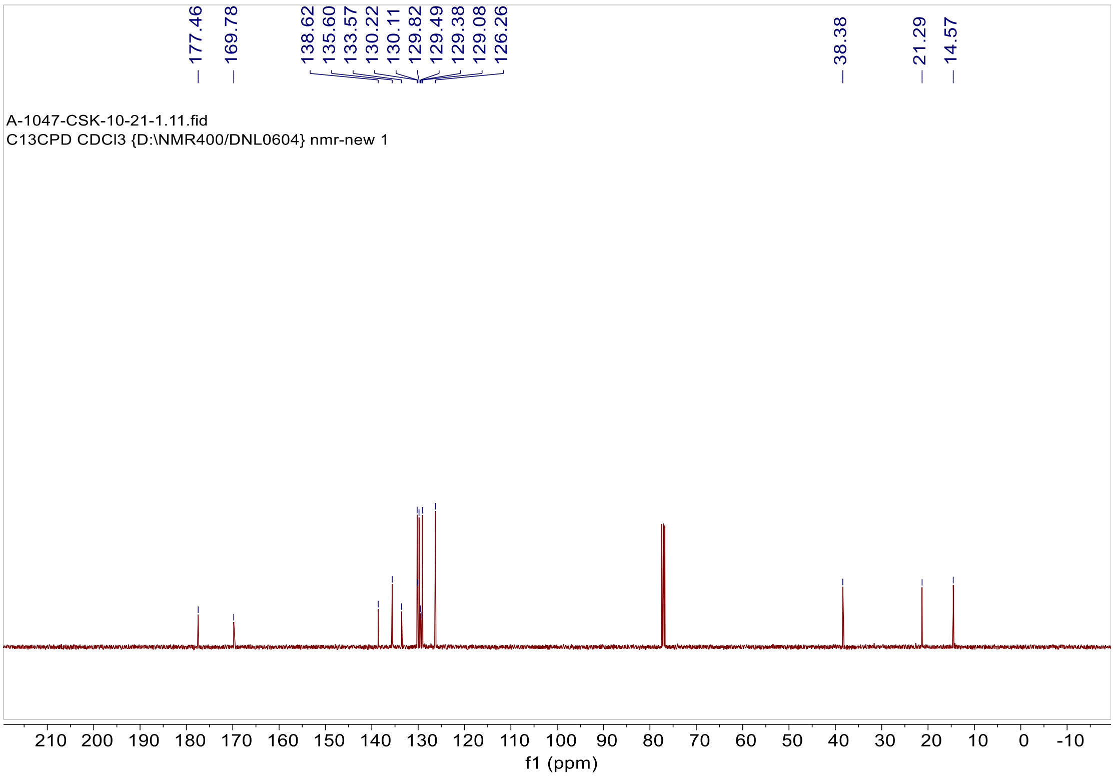

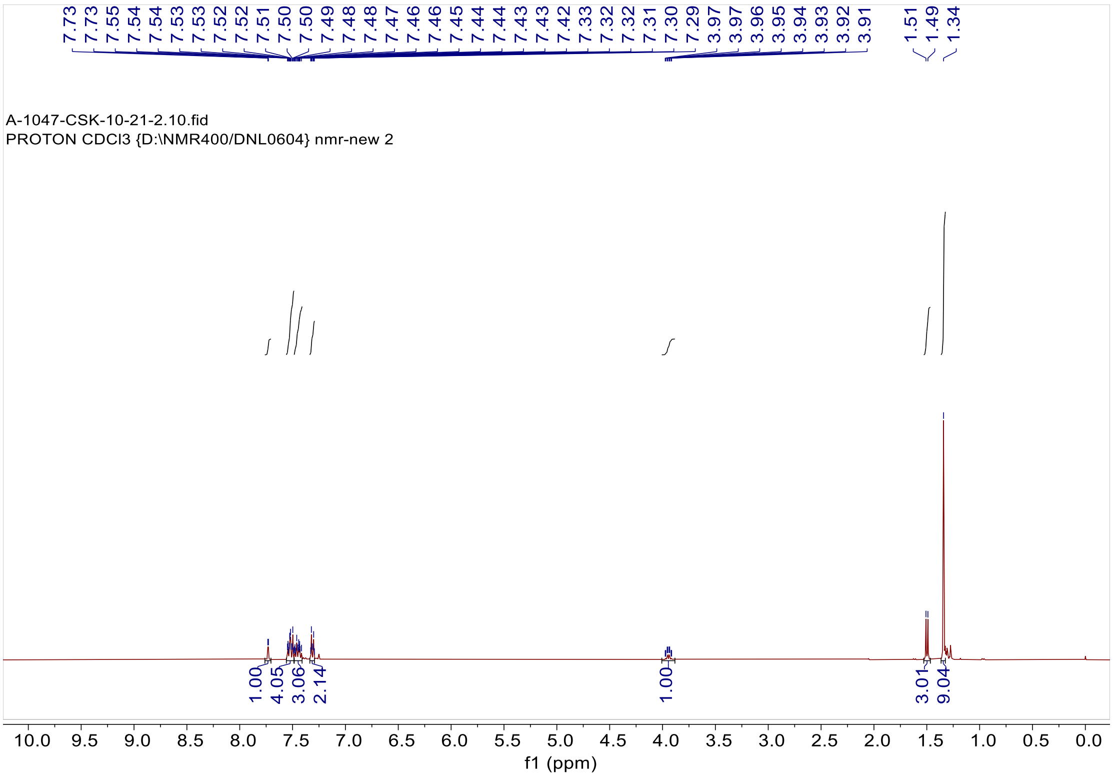

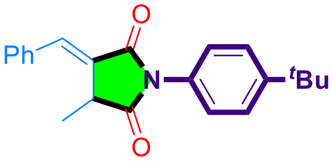

6c

^{1}H NMR (400 MHz, $CDCl_3$)
^{13}C NMR (100 MHz, $CDCl_3$)

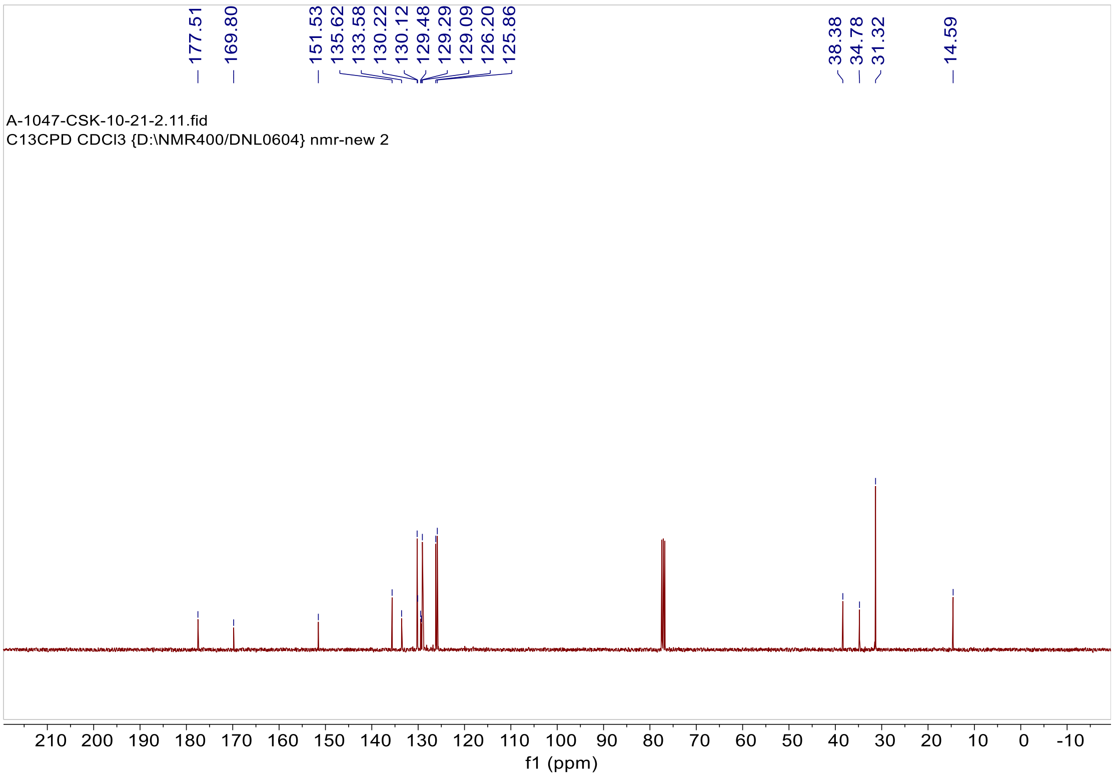

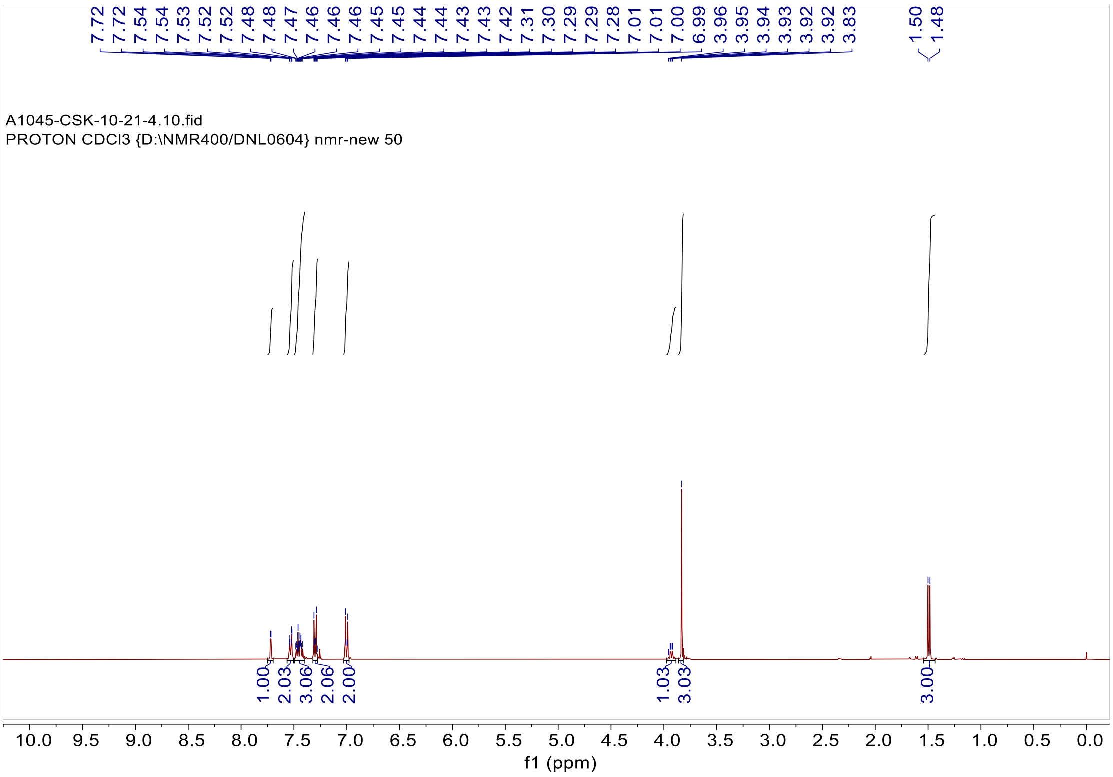

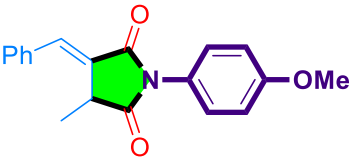

6d

^{1}H NMR (400 MHz, $CDCl_3$)

^{13}C NMR (100 MHz, $CDCl_3$)

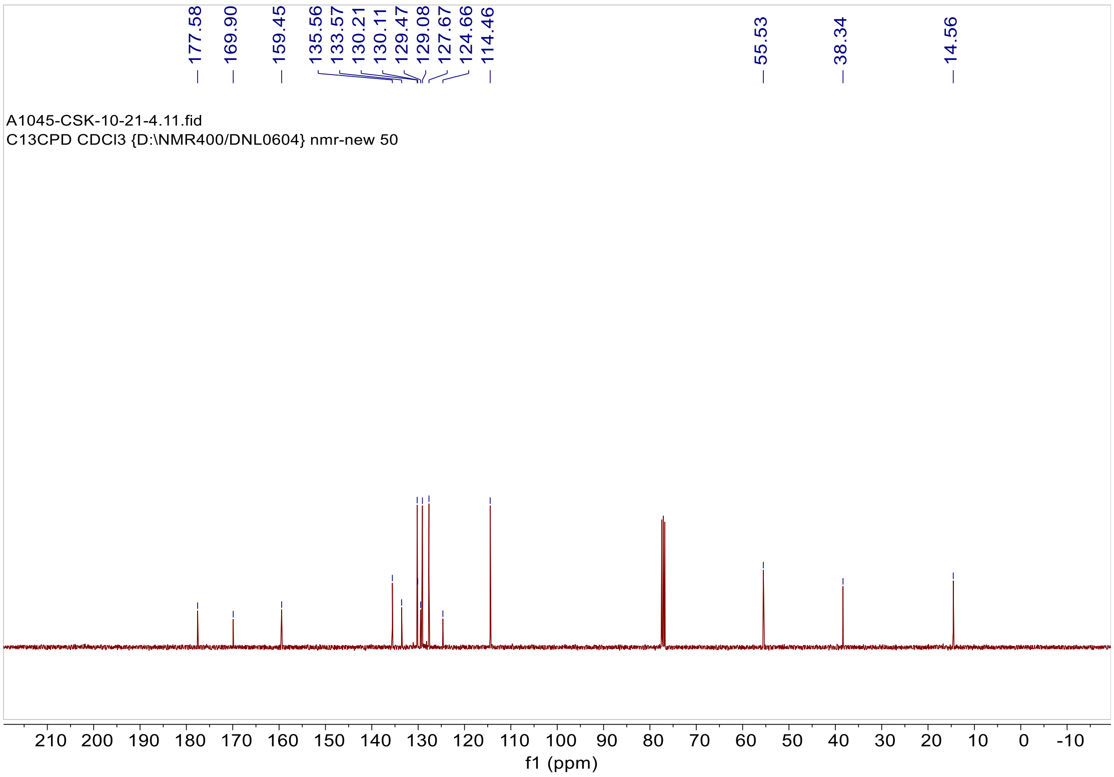

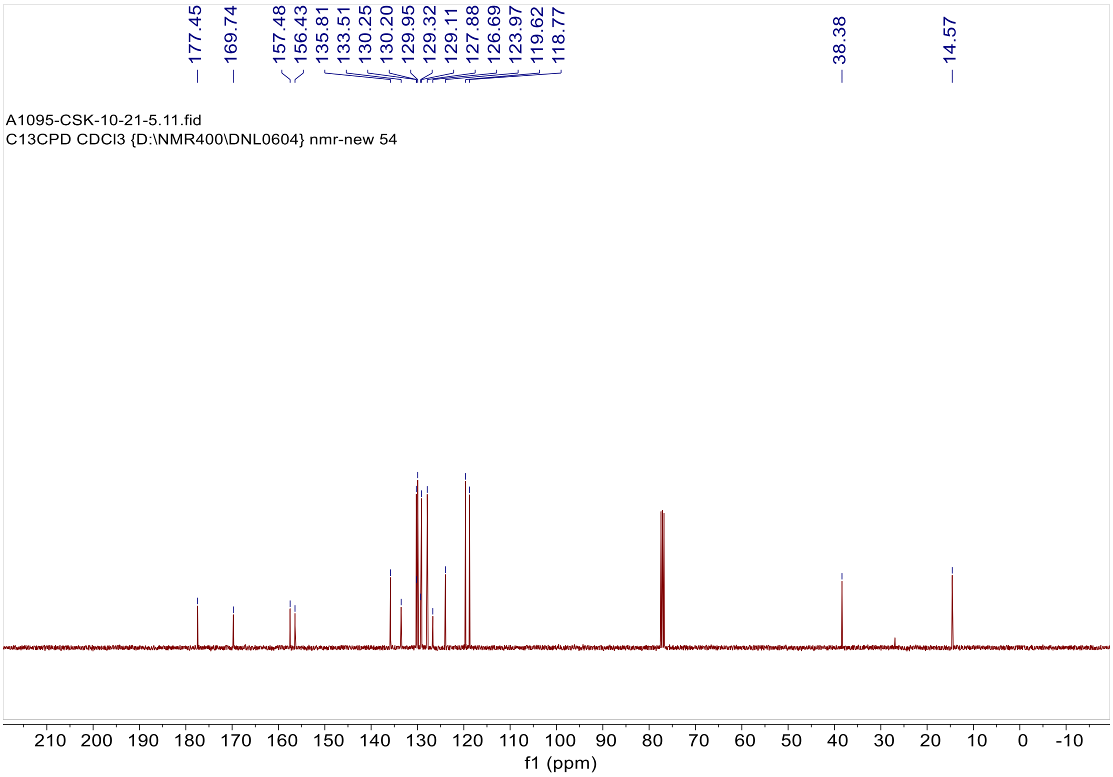

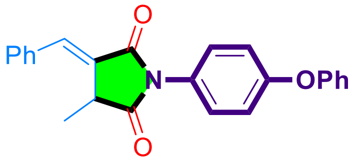

6e

^{1}H NMR (400 MHz, $CDCl_3$)
^{13}C NMR (100 MHz, $CDCl_3$)

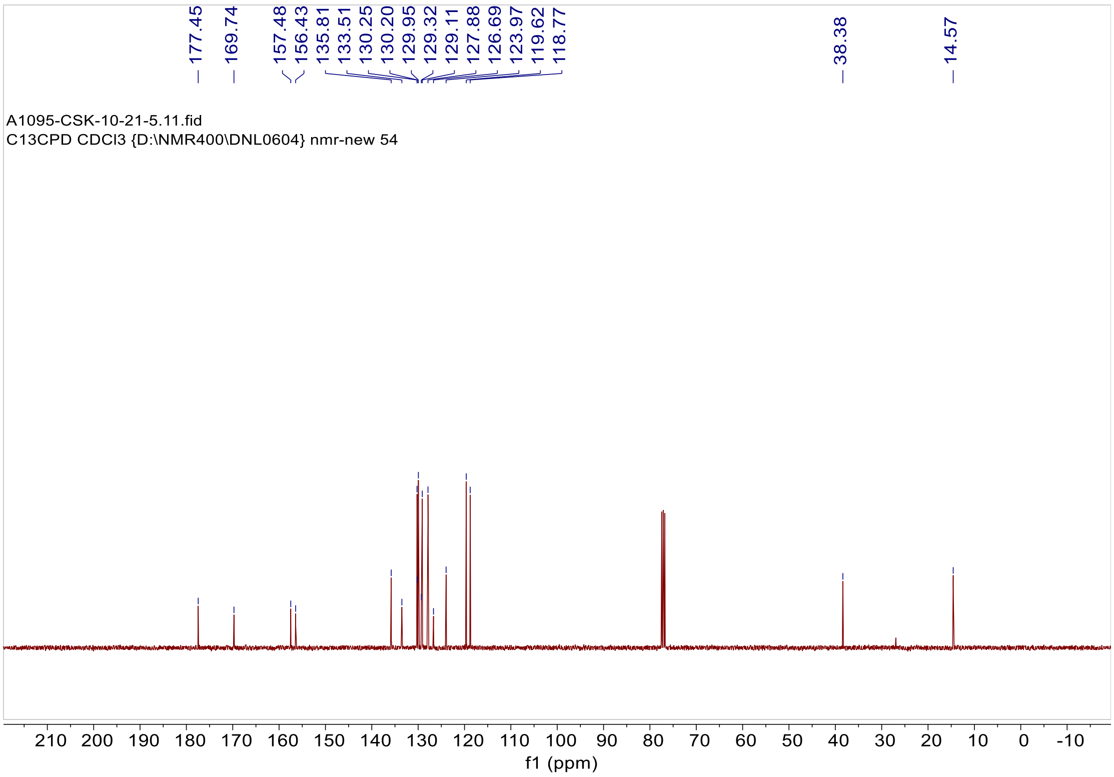

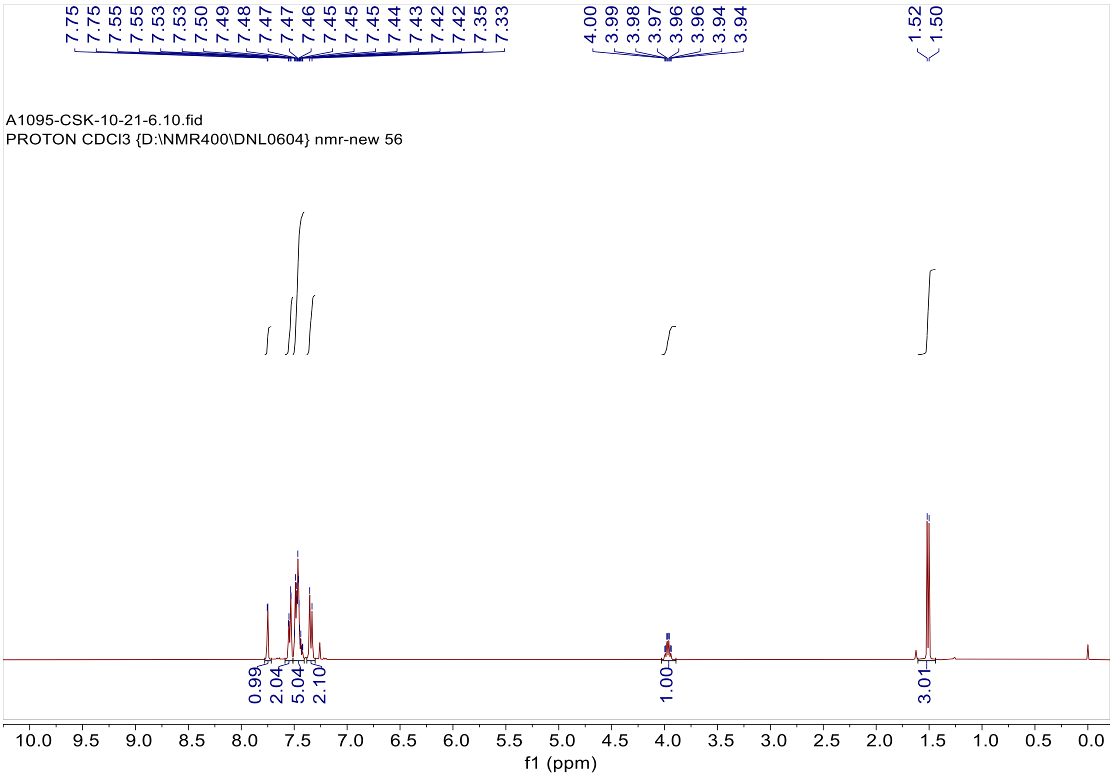

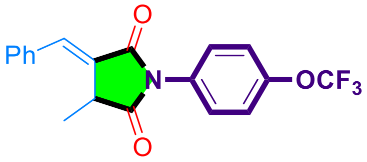

6f

^{1}H NMR (400 MHz, $CDCl_3$)
^{13}C NMR (100 MHz, $CDCl_3$)

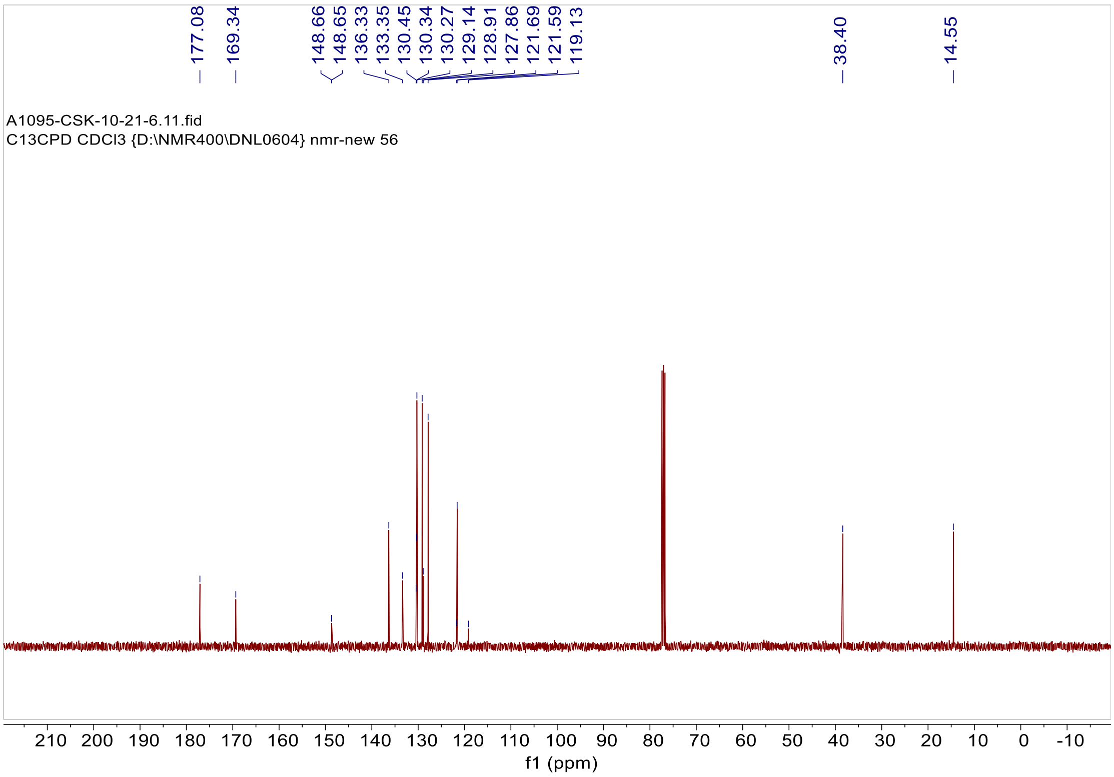

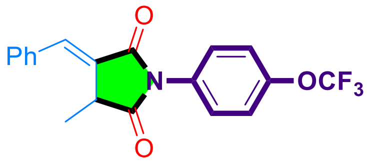

6f

^{19}F NMR (376 MHz, $CDCl_3$)

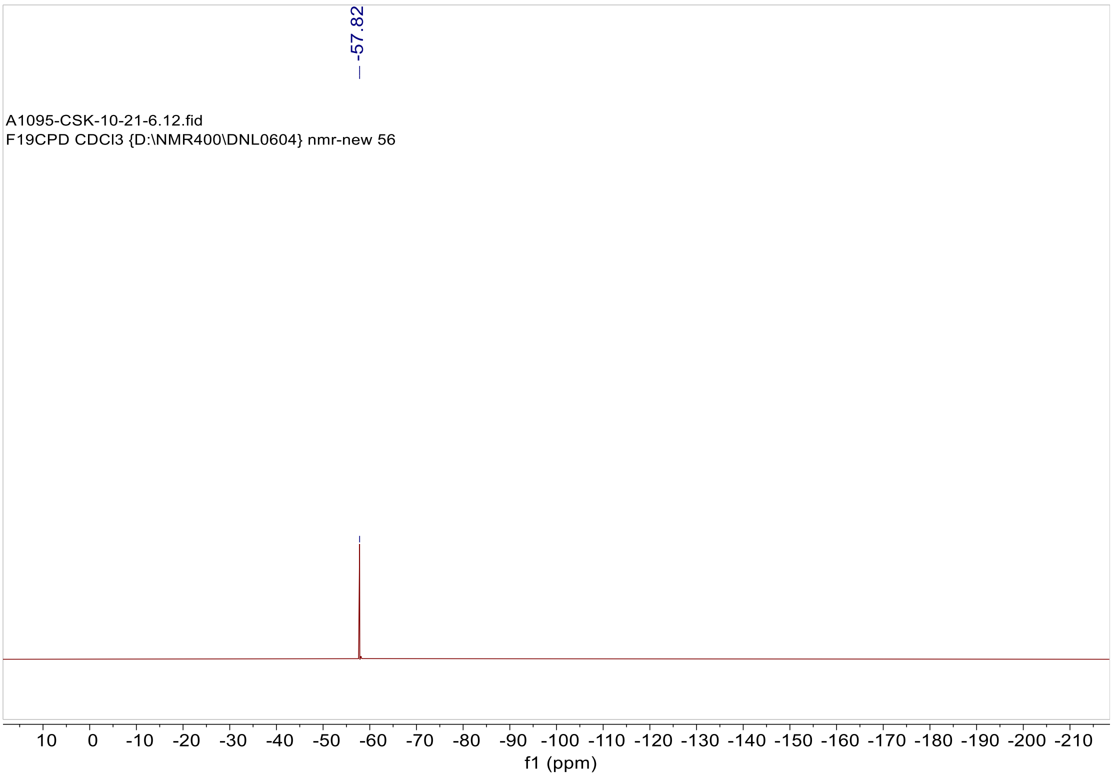

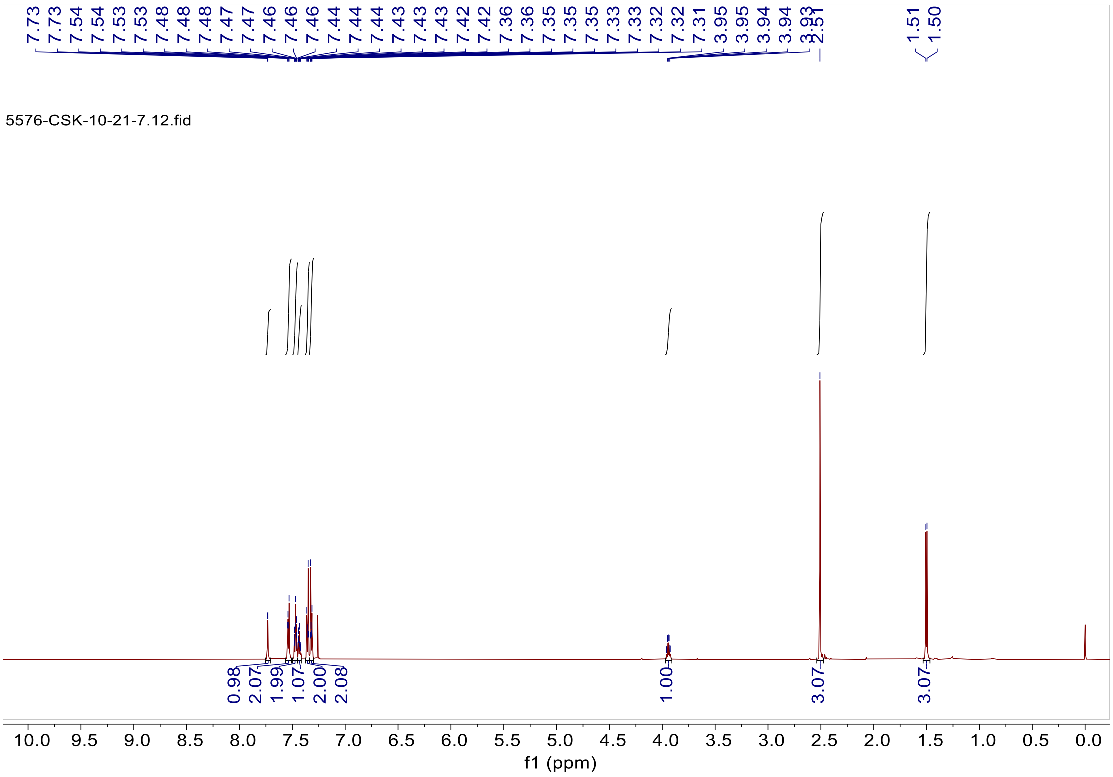

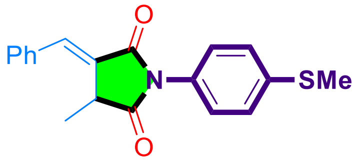

6g

^{1}H NMR (400 MHz, $CDCl_3$)
^{13}C NMR (100 MHz, $CDCl_3$)

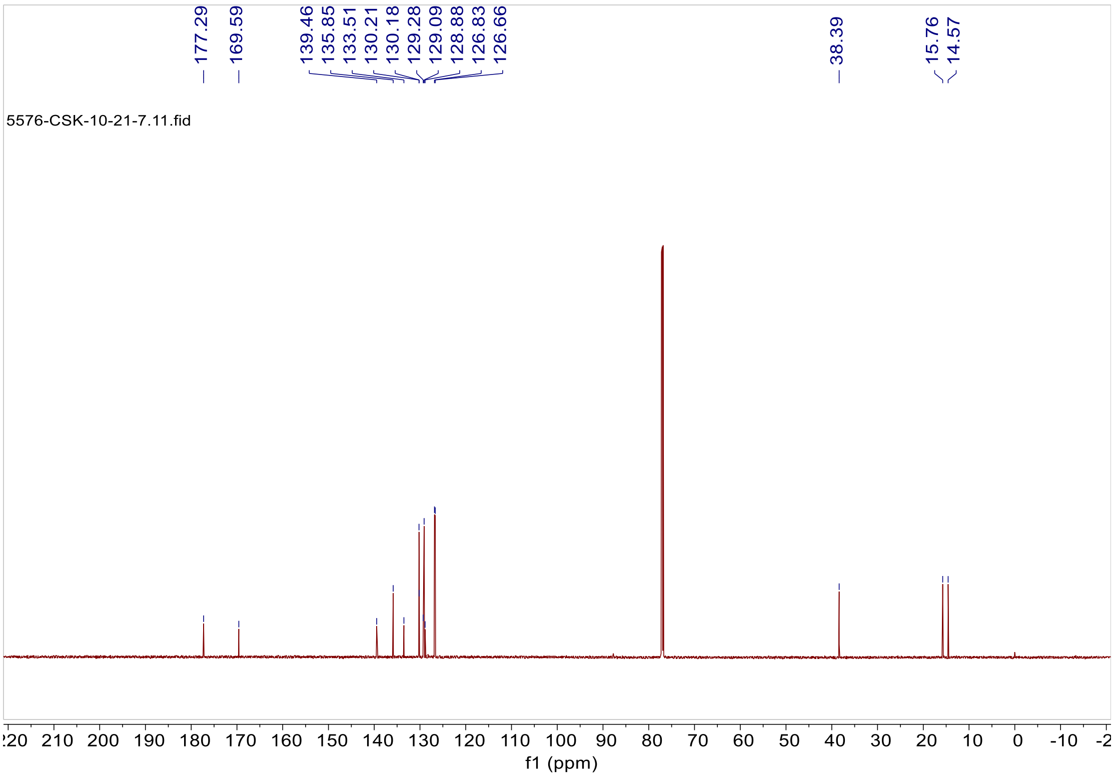

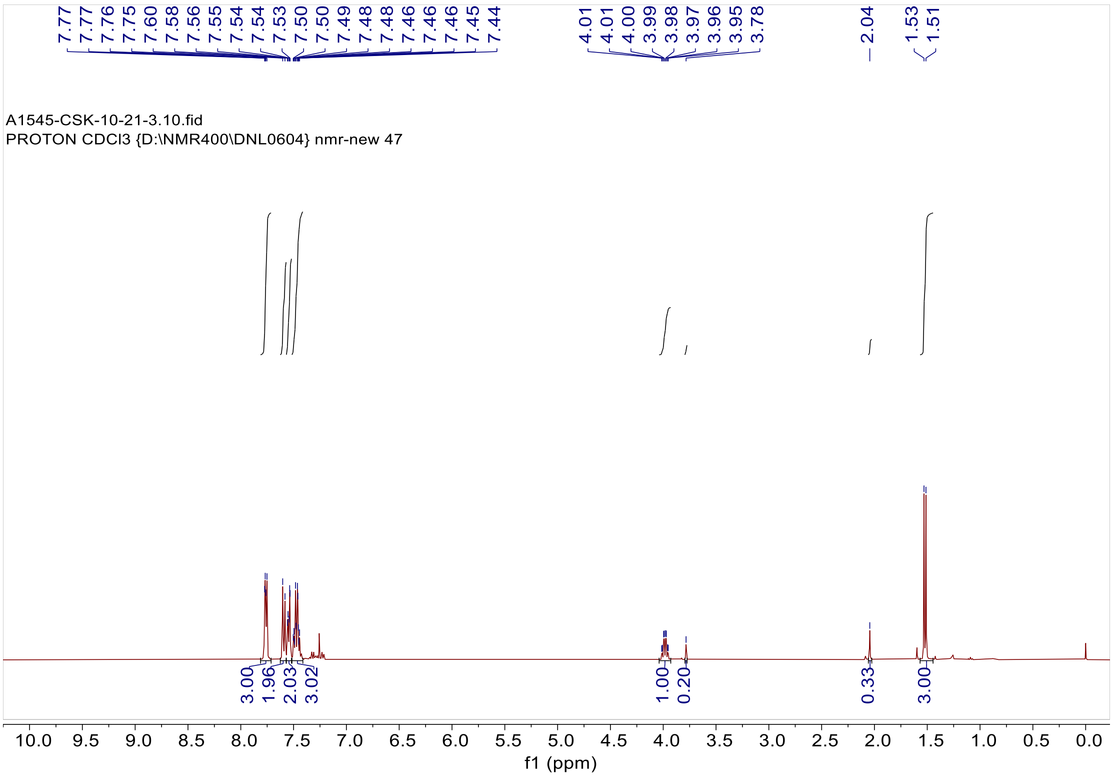

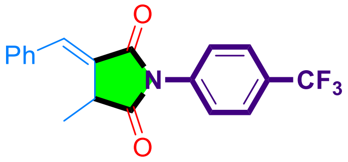

6h

^{1}H NMR (400 MHz, $CDCl_3$)
^{13}C NMR (100 MHz, $CDCl_3$)

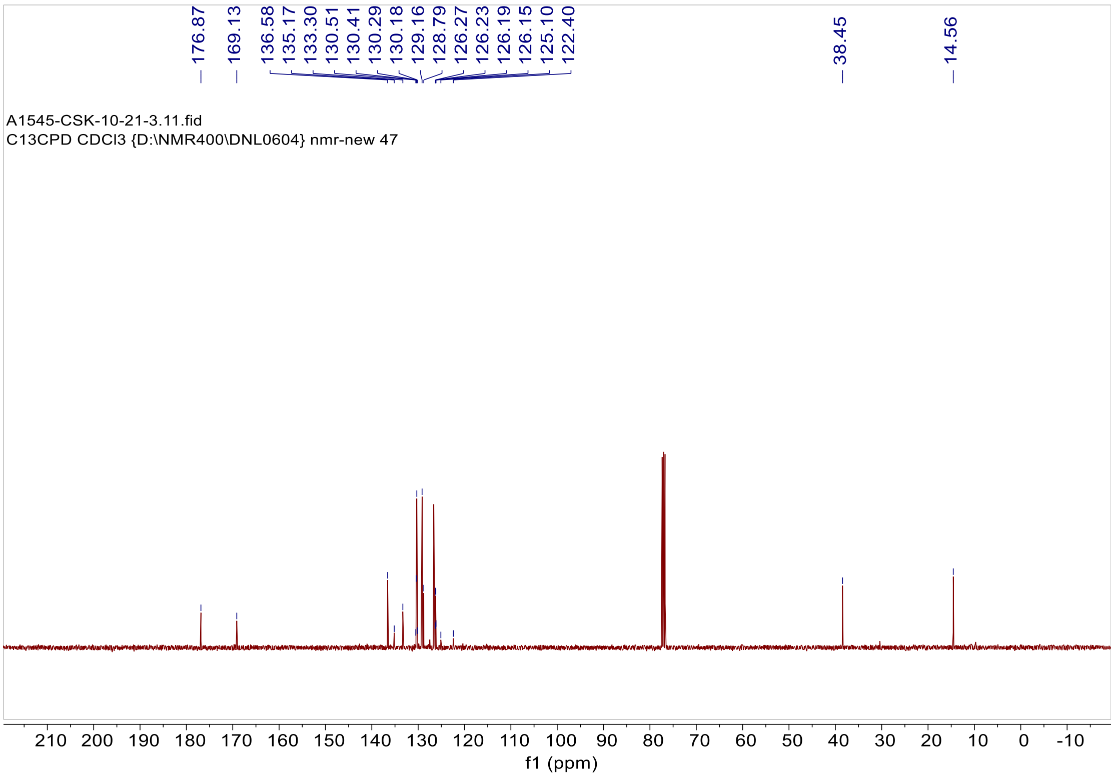

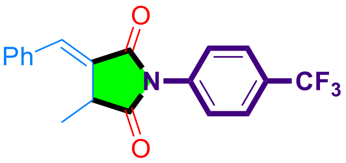

6h

^{19}F NMR (376 MHz, $CDCl_3$)

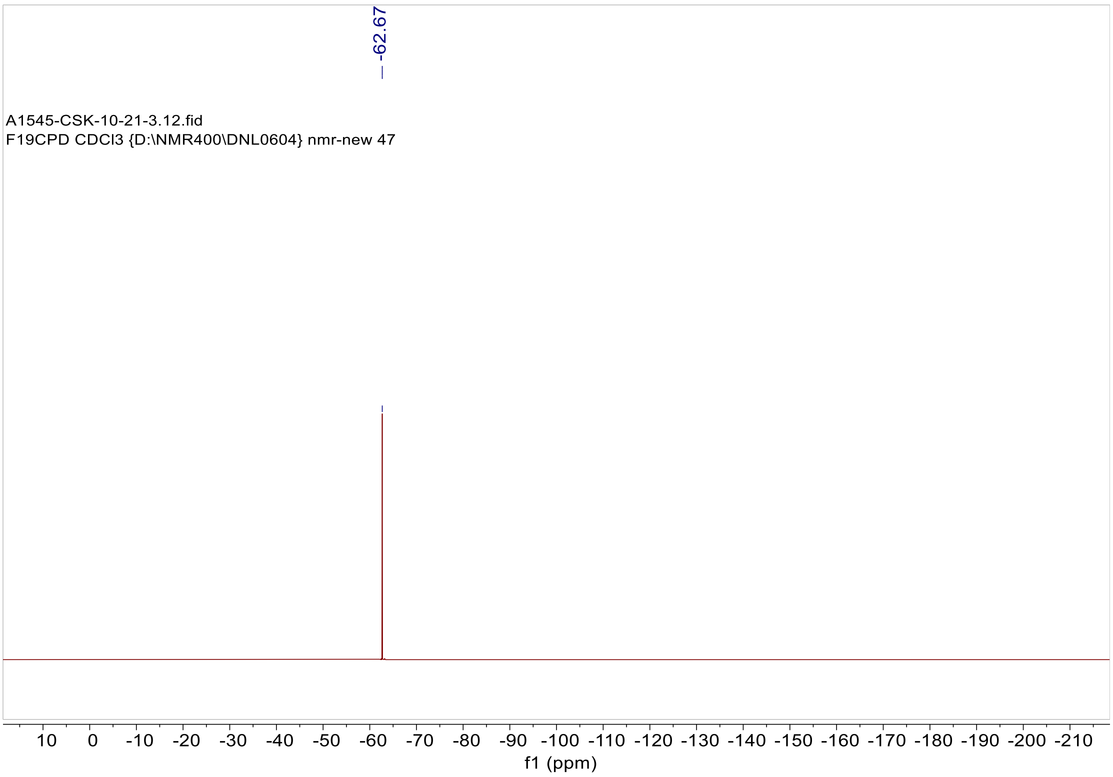

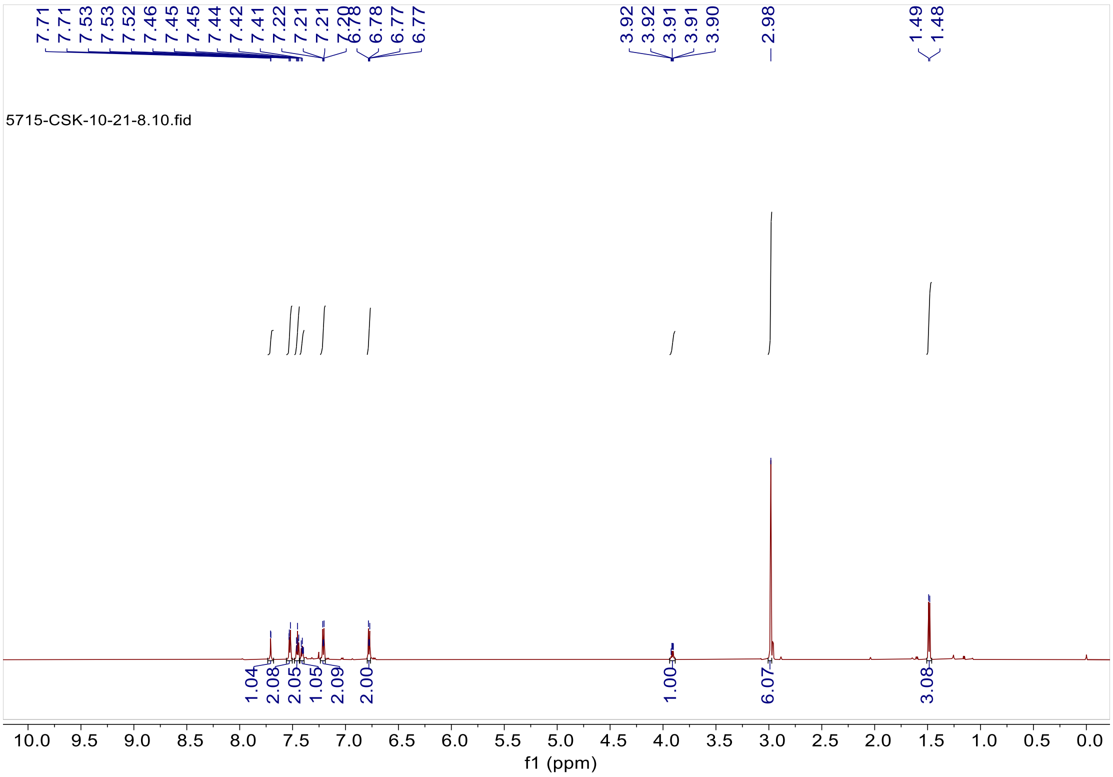

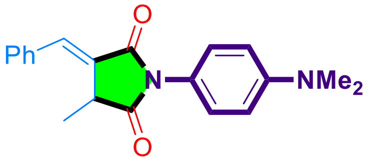

6i

^{1}H NMR (700 MHz, $CDCl_3$)

^{13}C NMR (176 MHz, $CDCl_3$)

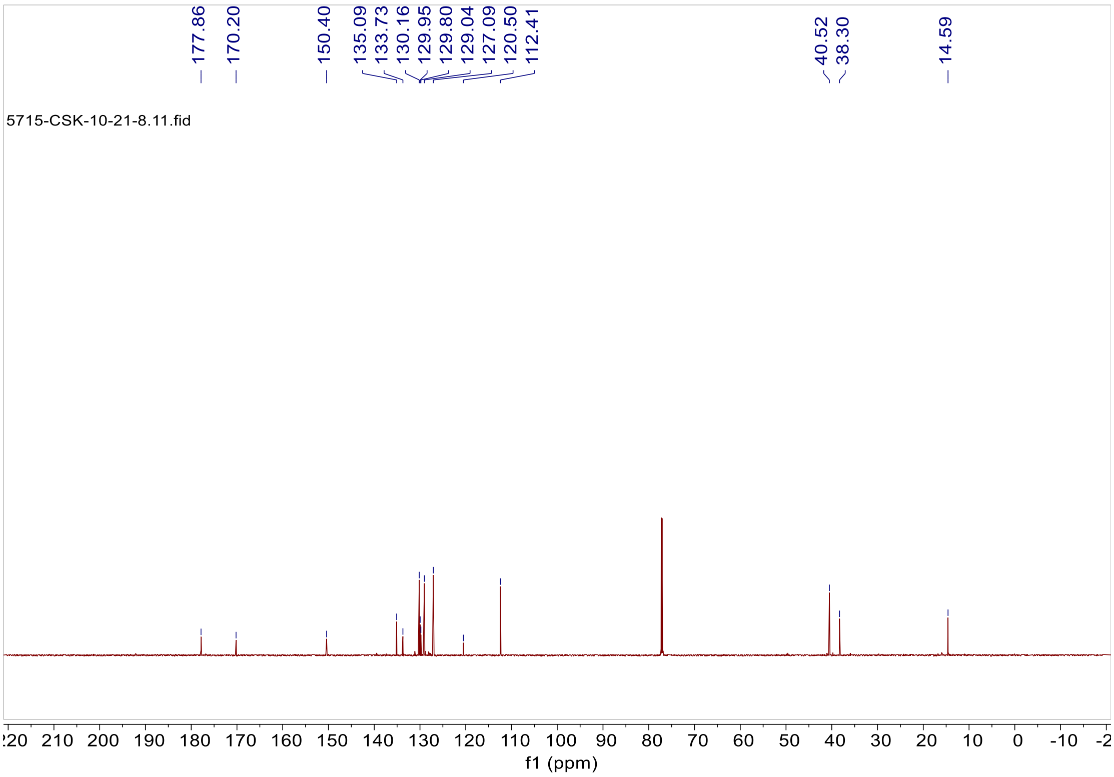

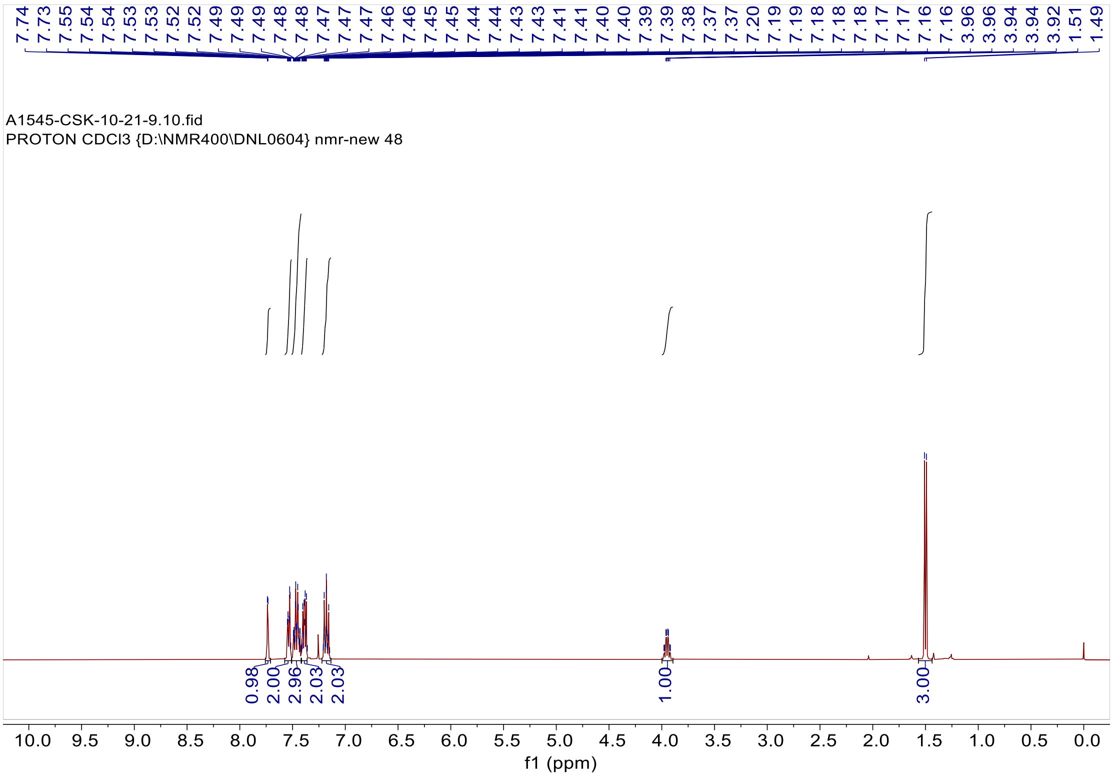

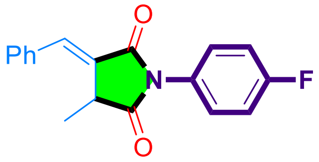

6j

^{1}H NMR (400 MHz, $CDCl_3$)
^{13}C NMR (100 MHz, $CDCl_3$)

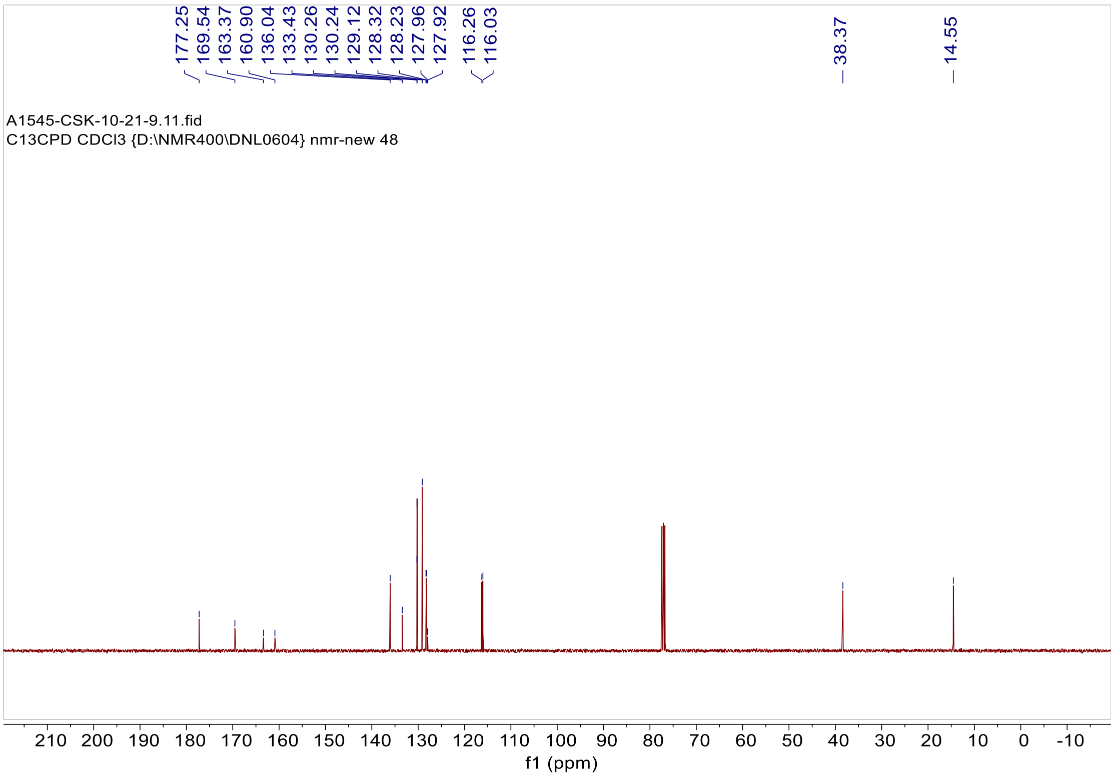

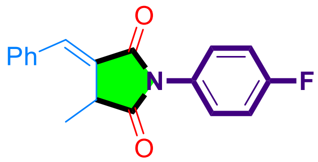

6j

^{19}F NMR (376 MHz, CDCl$_3$)

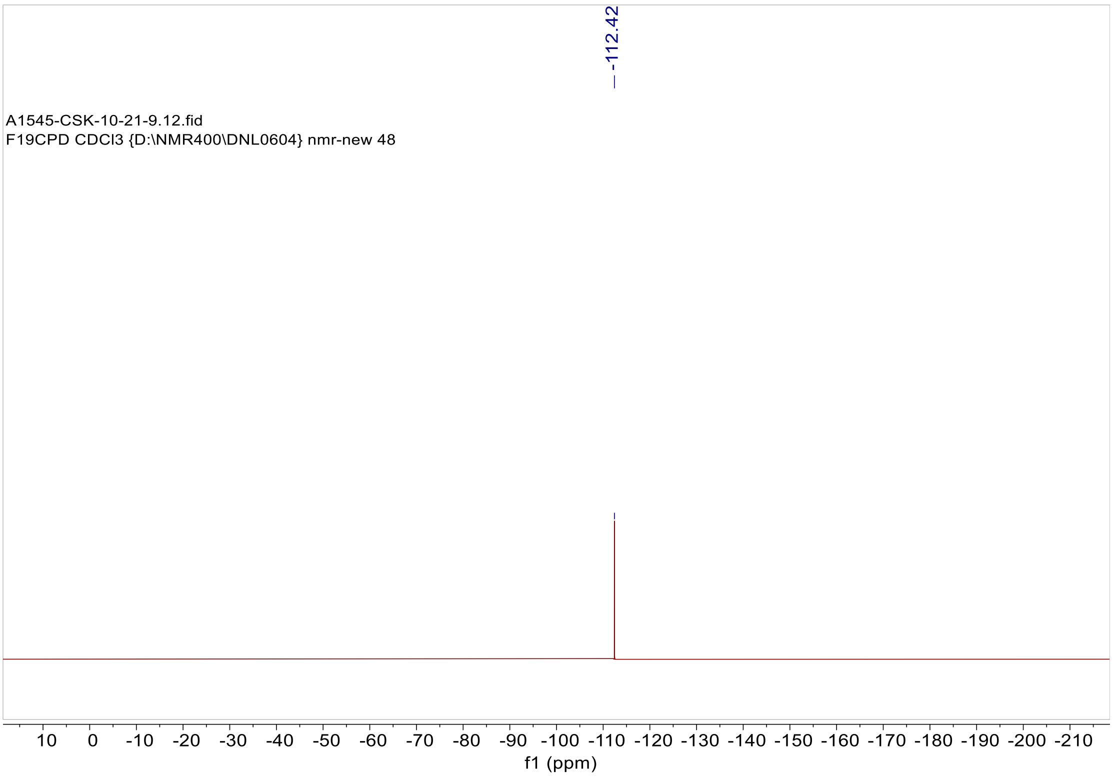

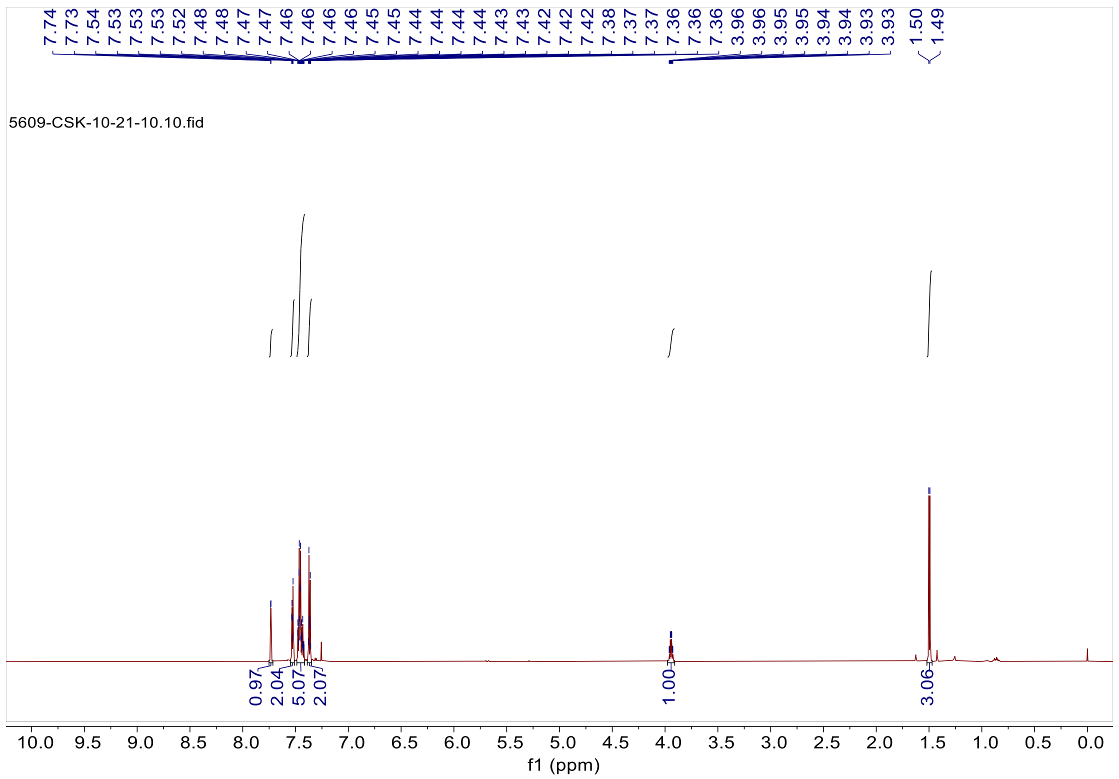

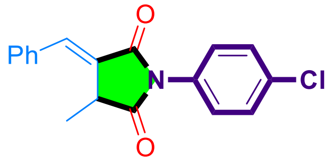

6k

^{1}H NMR (700 MHz, $CDCl_3$)
^{13}C NMR (176 MHz, $CDCl_3$)

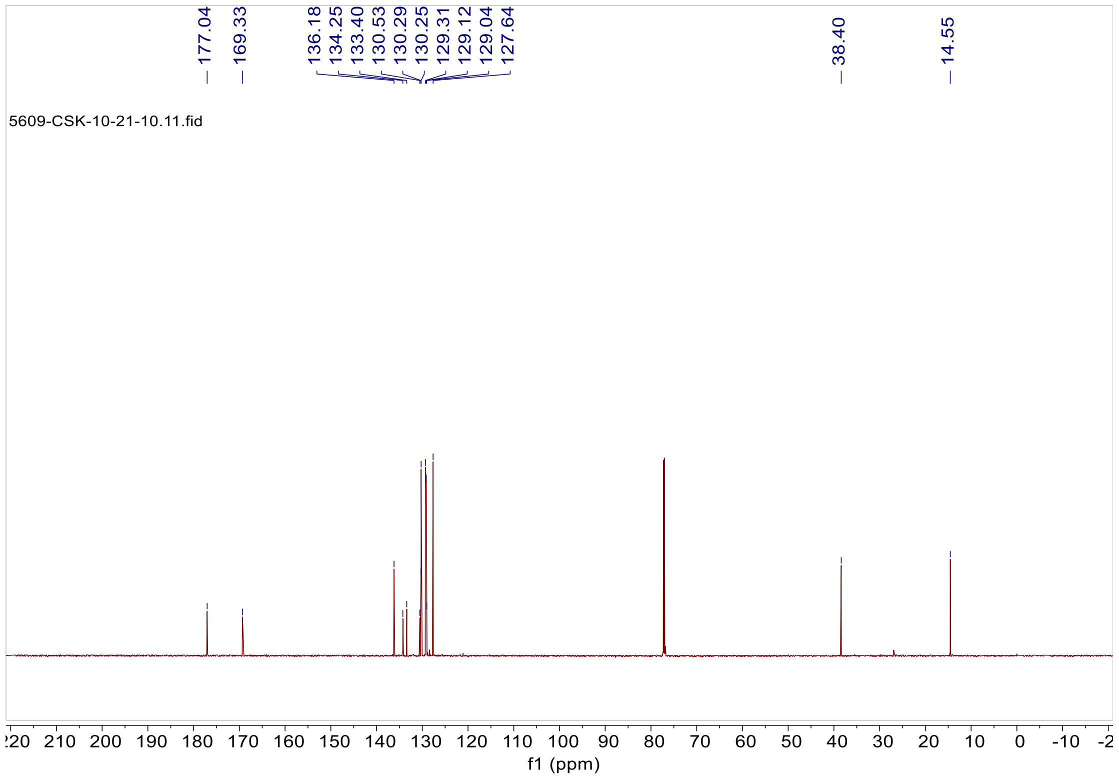

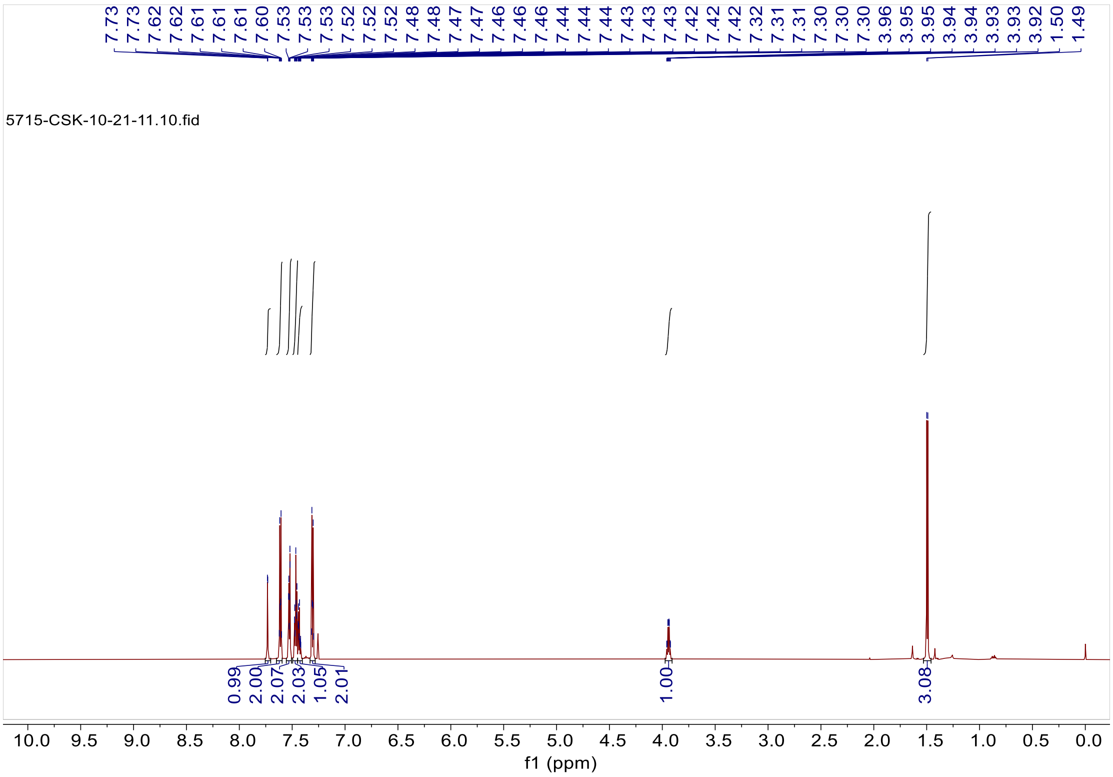

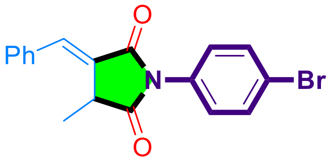

6l

^{1}H NMR (700 MHz, $CDCl_3$)
^{13}C NMR (176 MHz, $CDCl_3$)

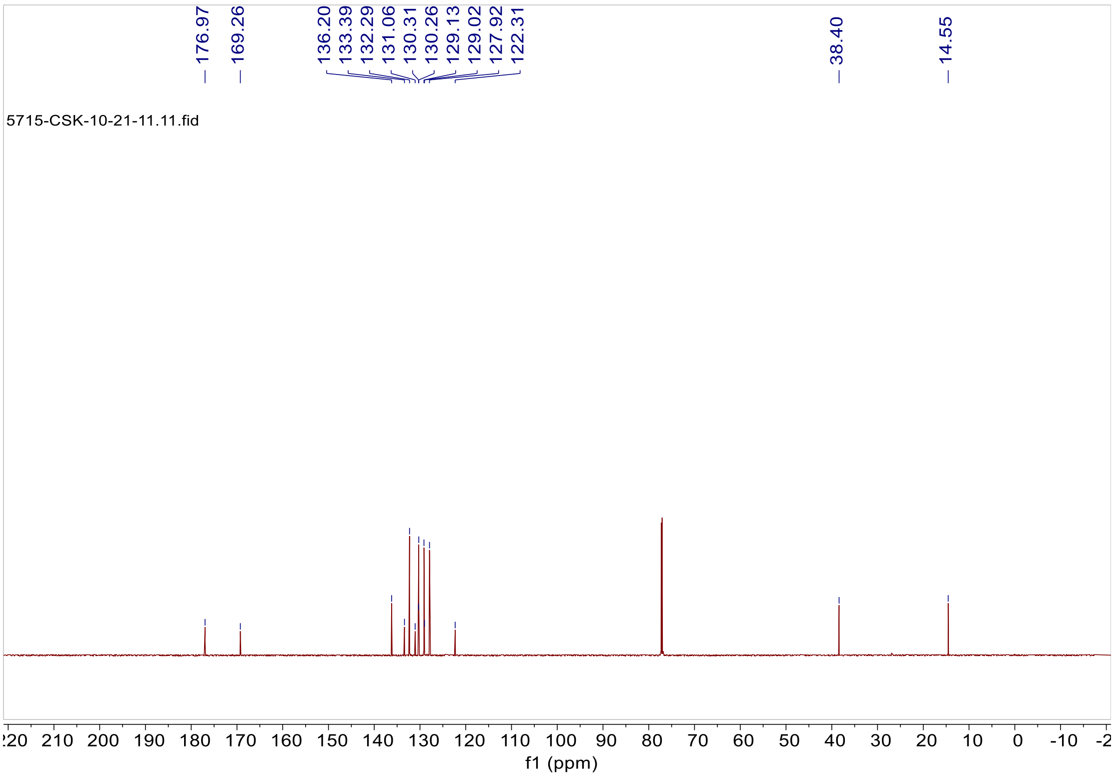

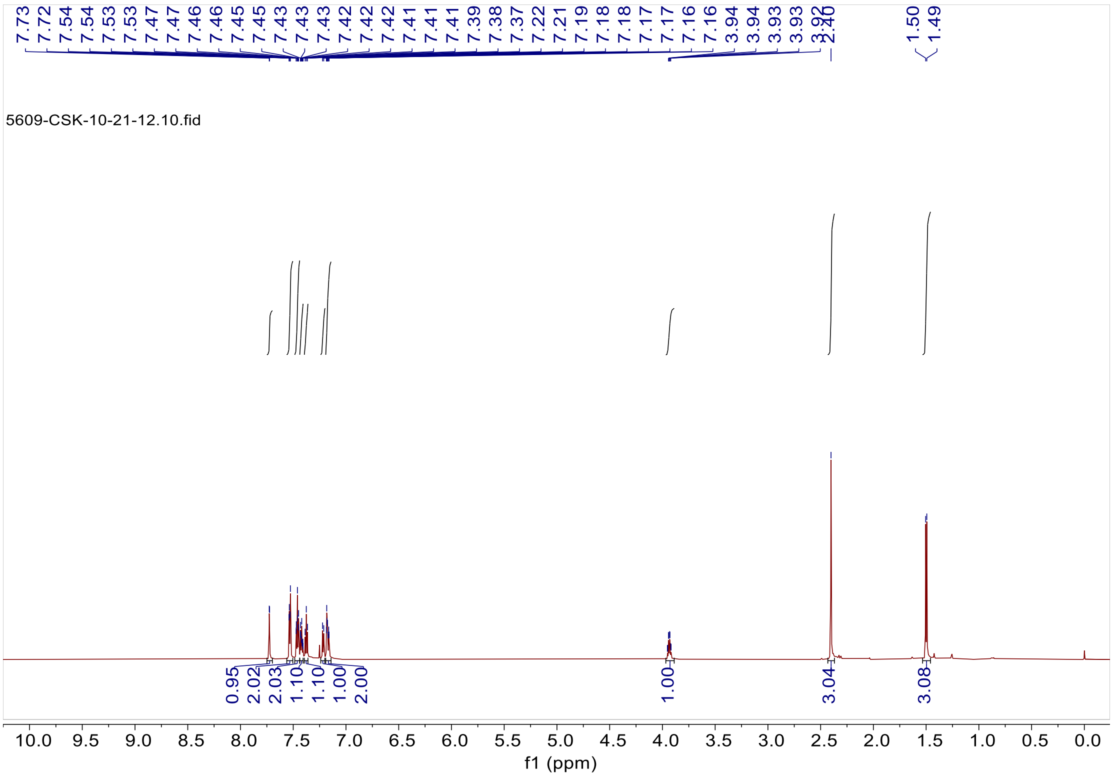

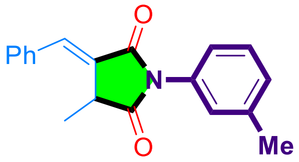

6m

^{1}H NMR (700 MHz, CDCl$_3$)

^{13}C NMR (176 MHz, CDCl$_3$)

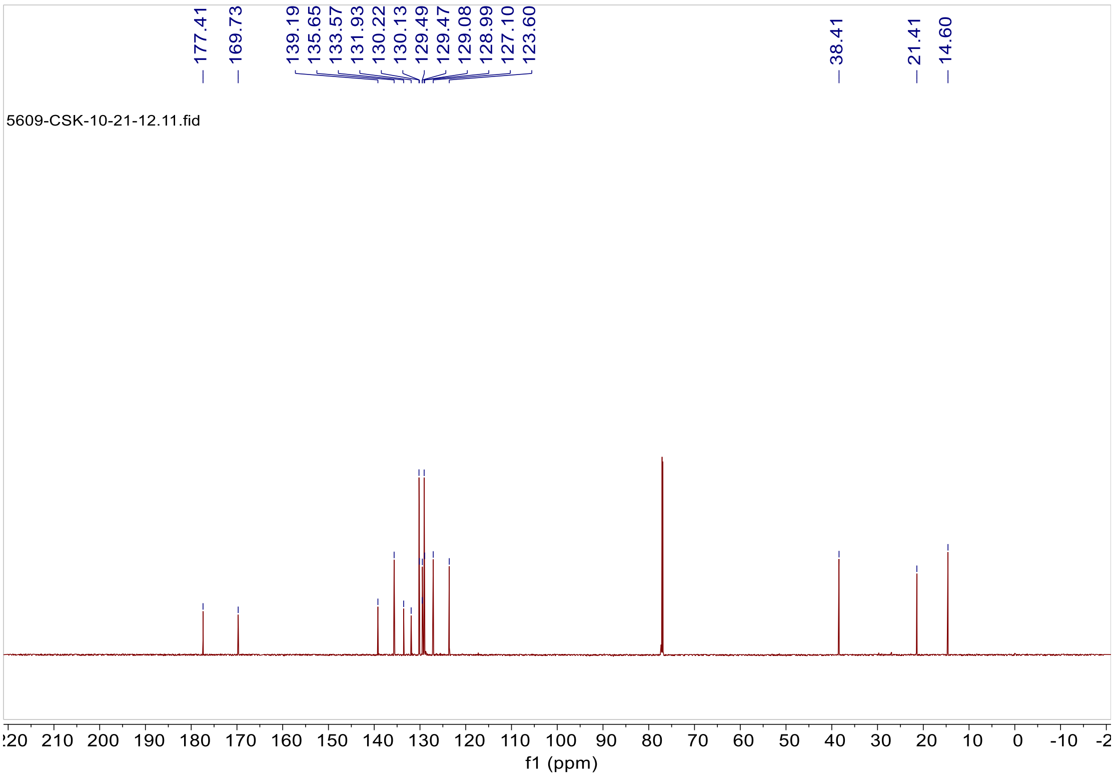

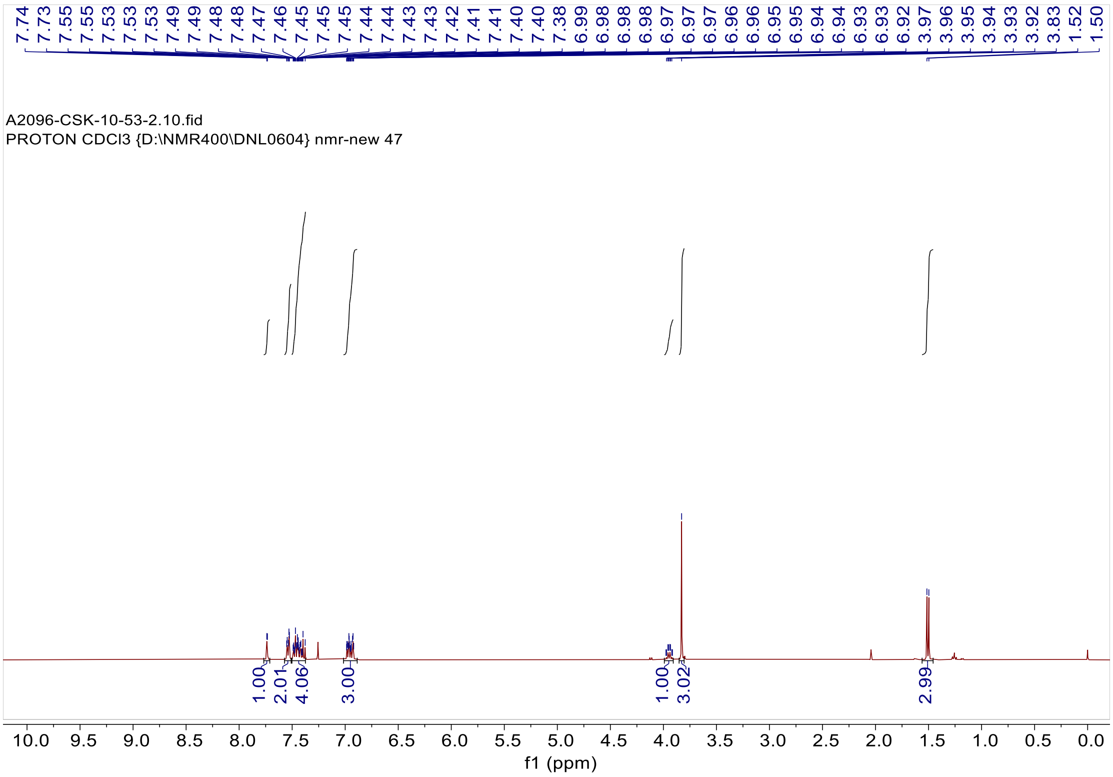

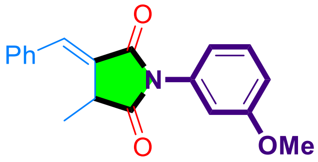

6n

^{1}H NMR (400 MHz, $CDCl_3$)

^{13}C NMR (100 MHz, $CDCl_3$)

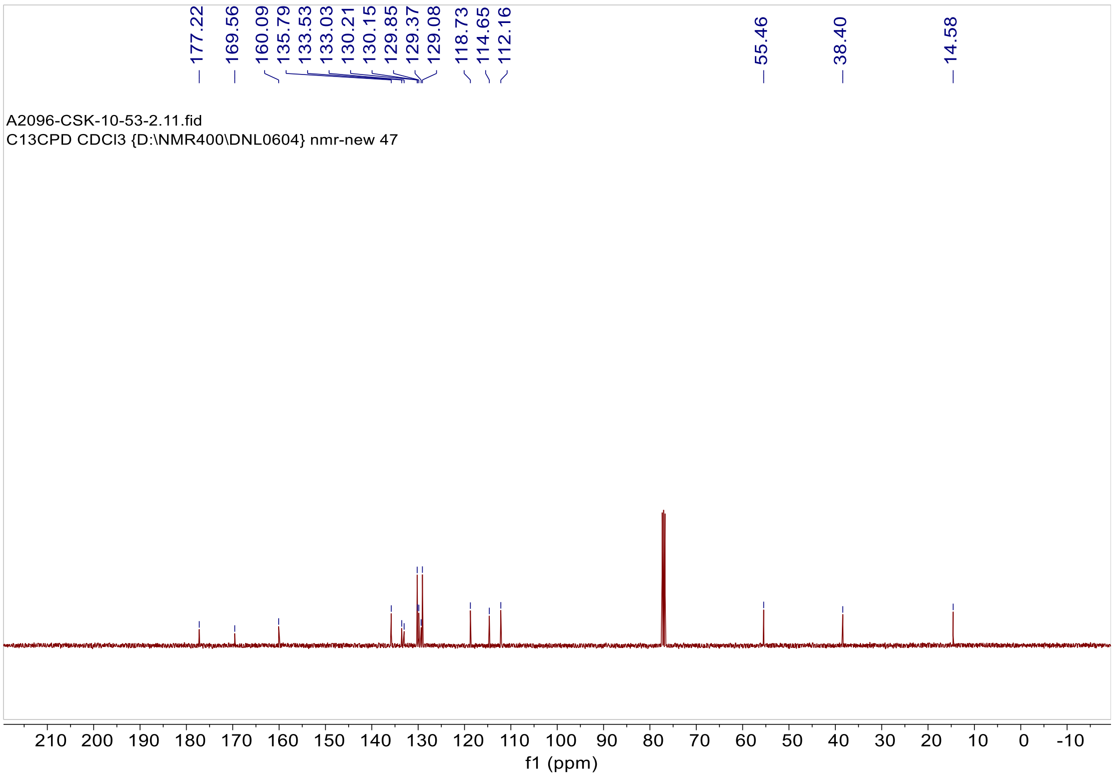

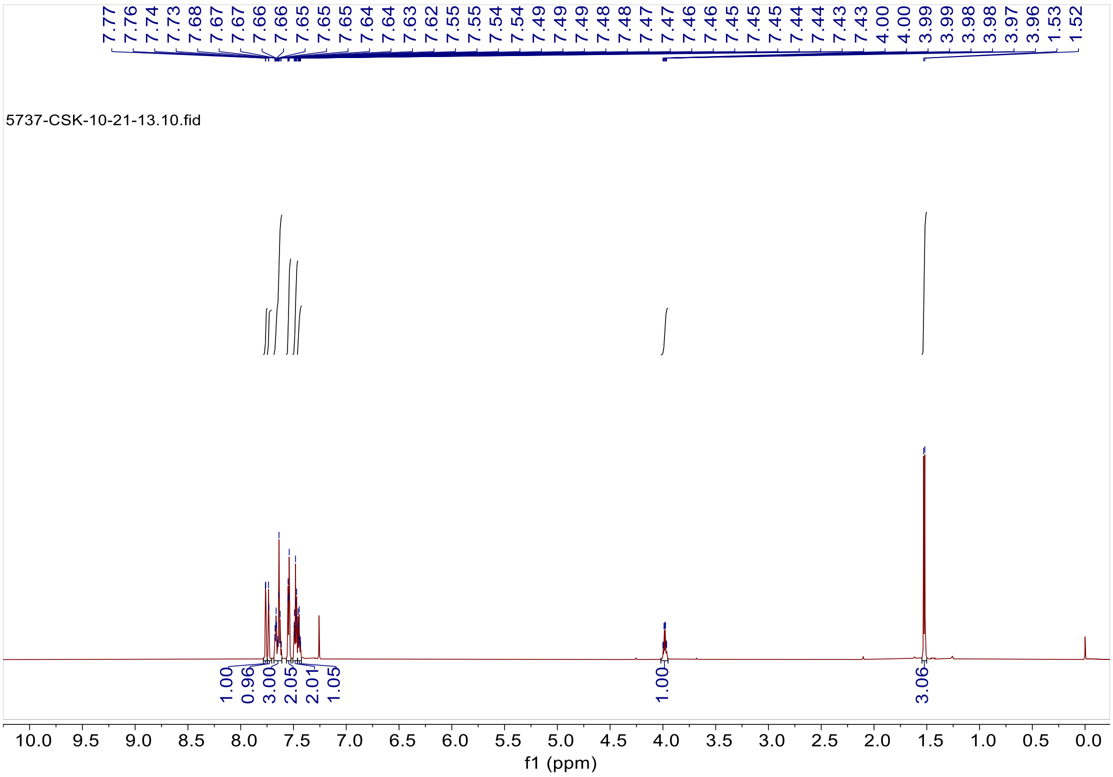

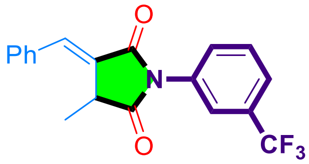

6o

^{1}H NMR (700 MHz, $CDCl_3$)

^{13}C NMR (176 MHz, $CDCl_3$)

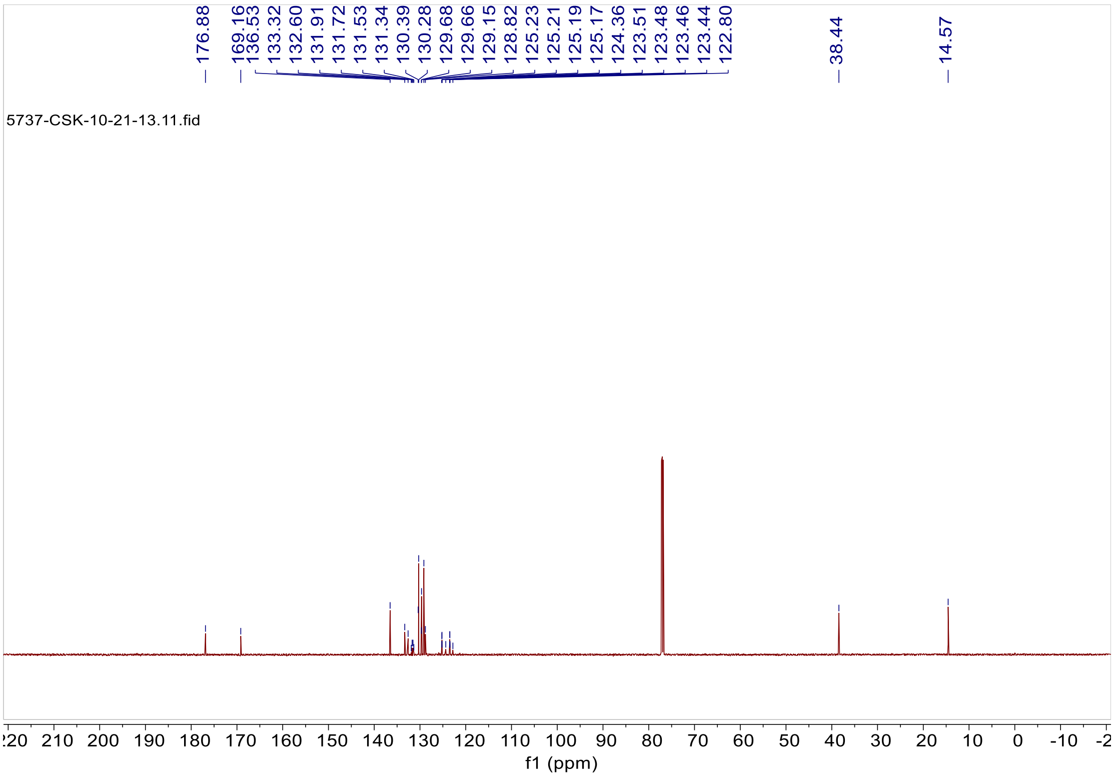

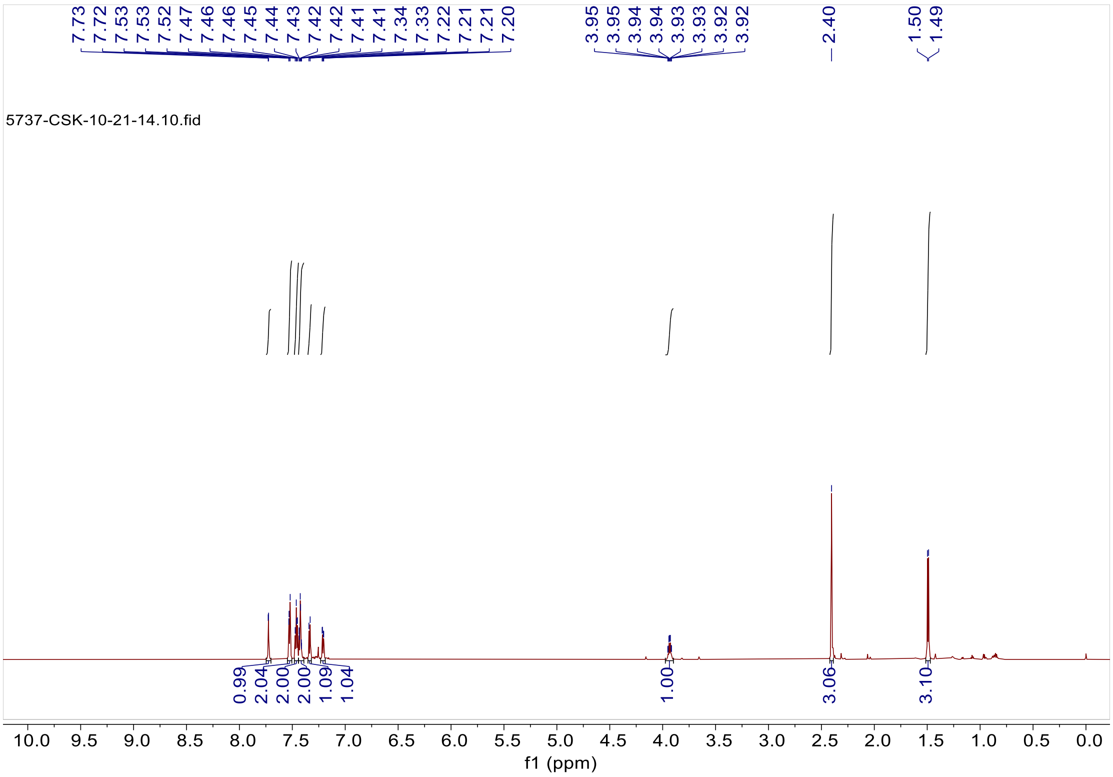

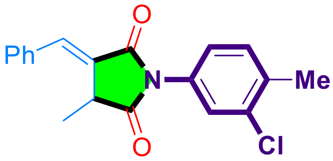

6p

^{1}H NMR (700 MHz, $CDCl_3$)
^{13}C NMR (176 MHz, $CDCl_3$)

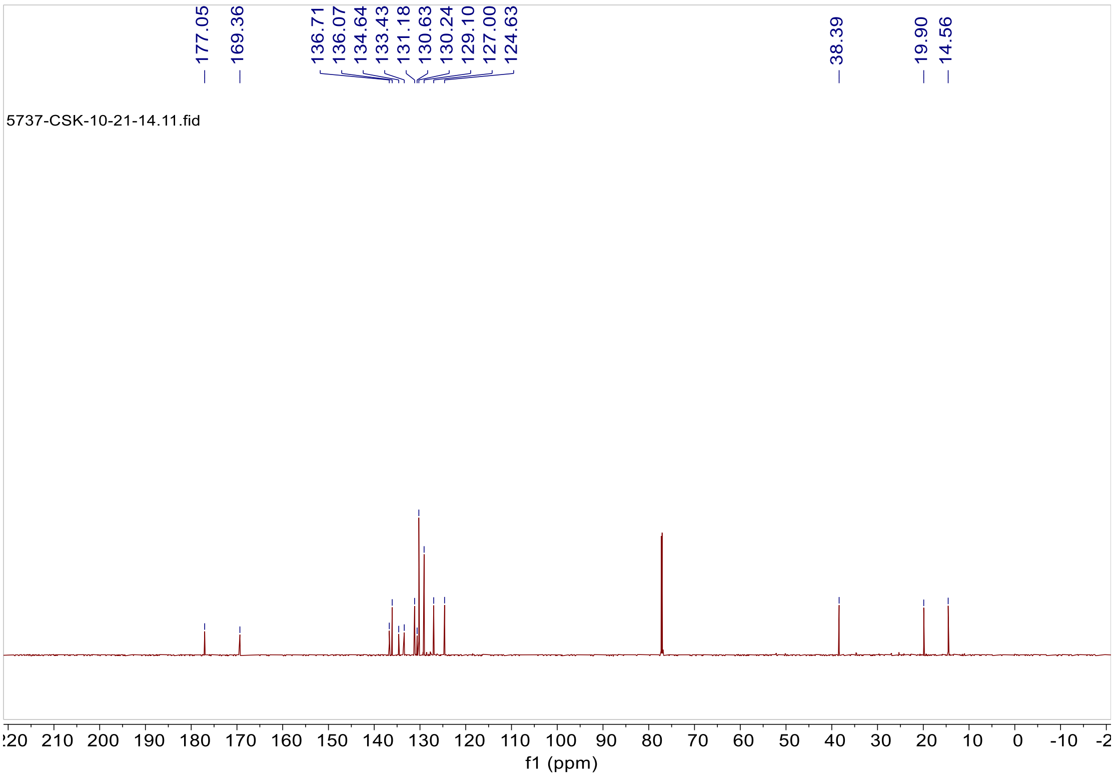

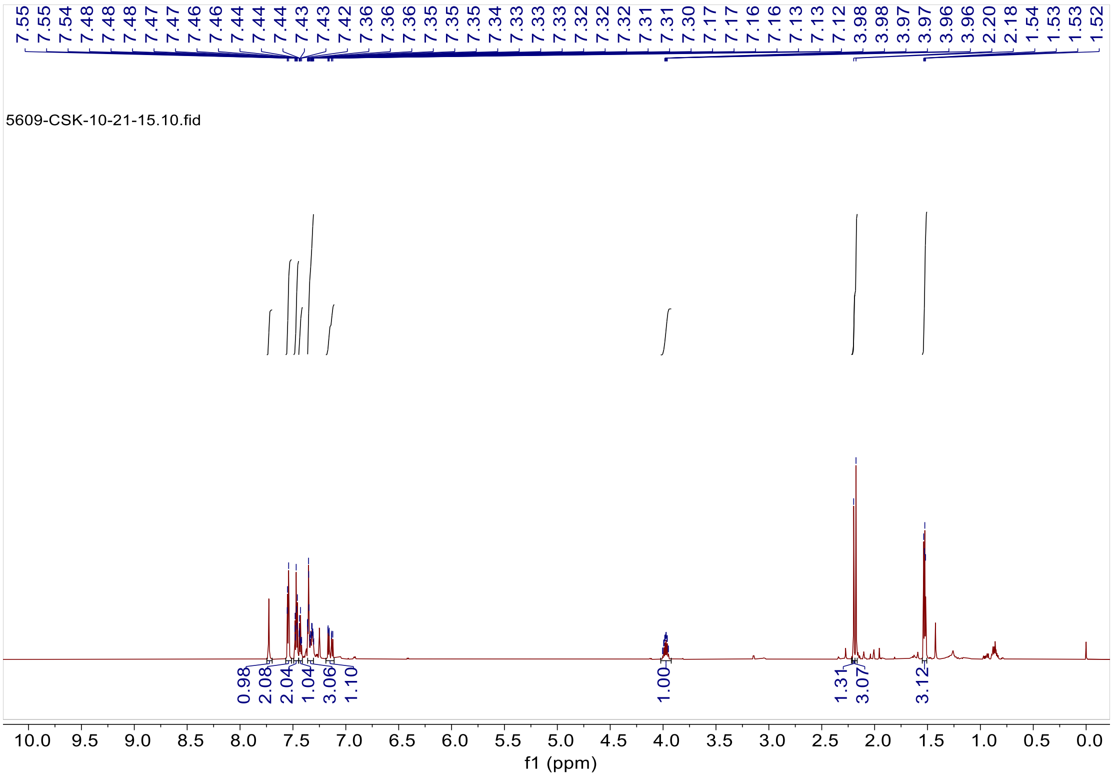

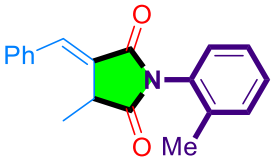

6q

^{1}H NMR (700 MHz, $CDCl_3$)
^{13}C NMR (176 MHz, $CDCl_3$)

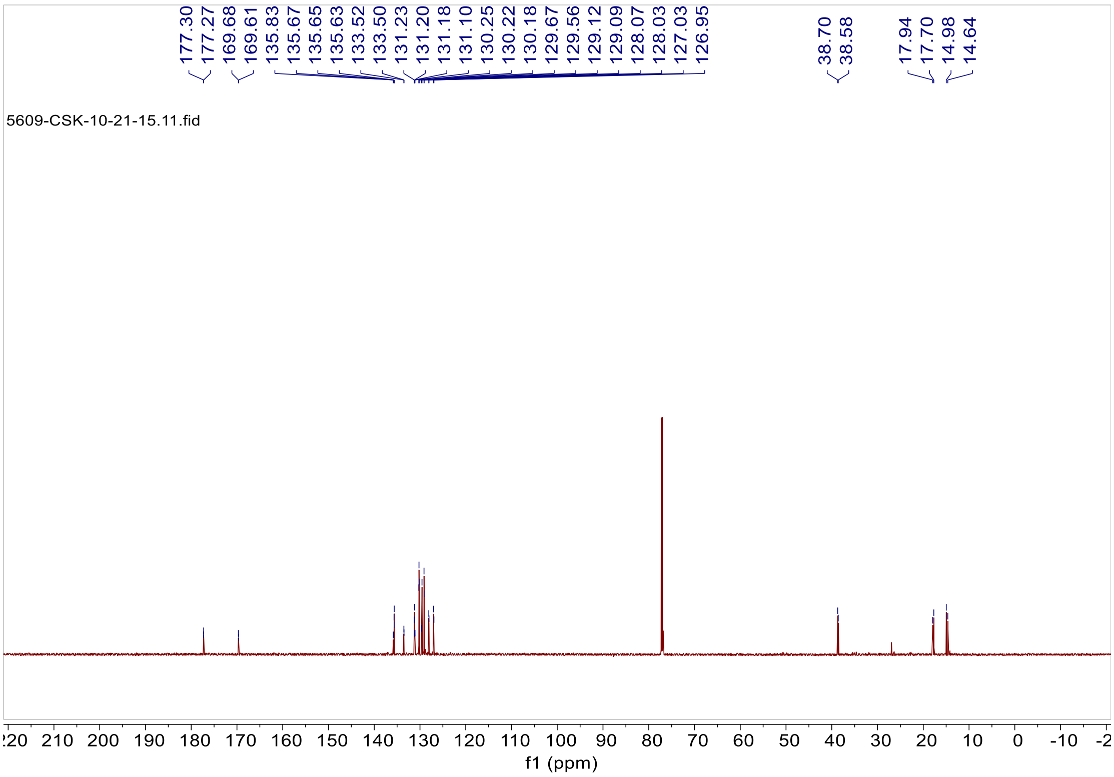

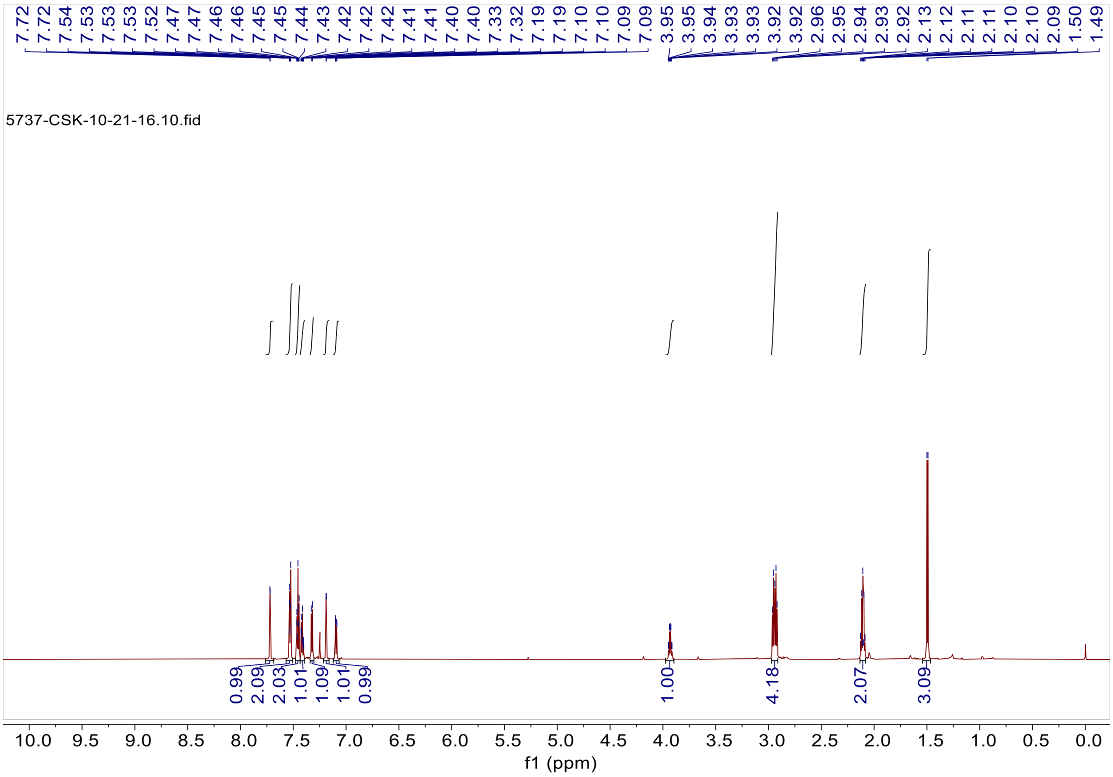

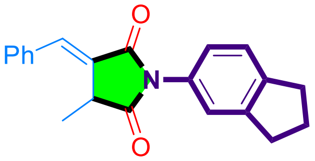

6r

^{1}H NMR (700 MHz, $CDCl_3$)
^{13}C NMR (176 MHz, $CDCl_3$)

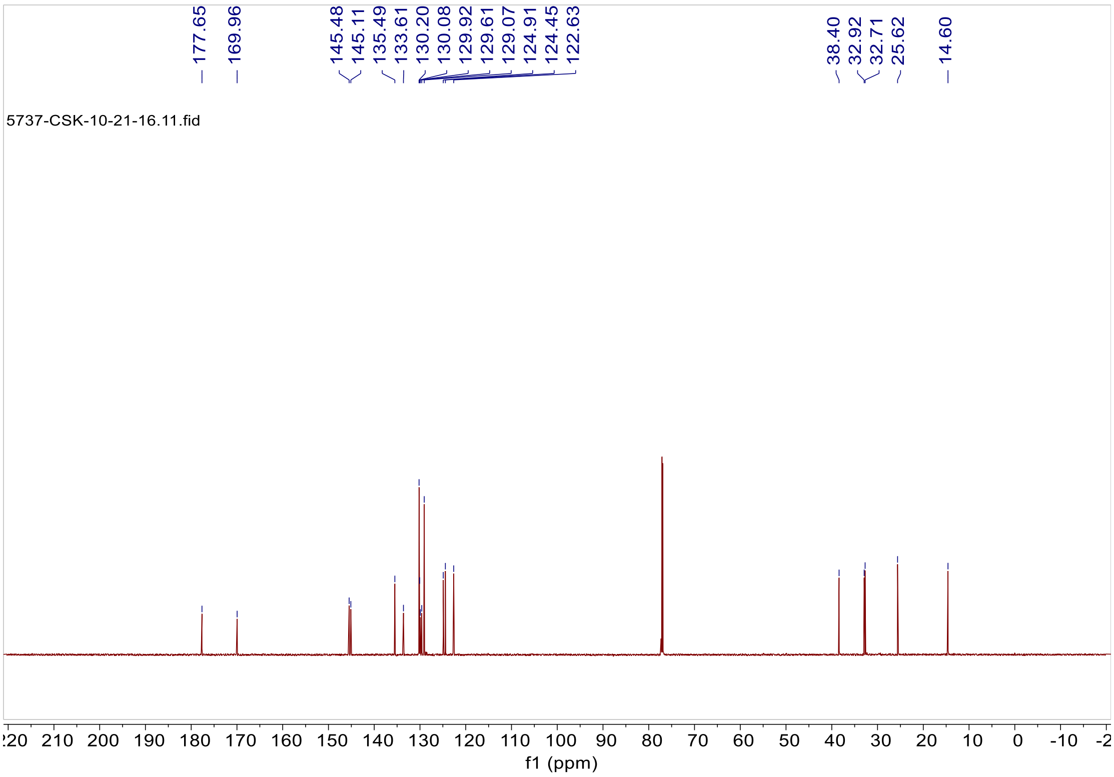

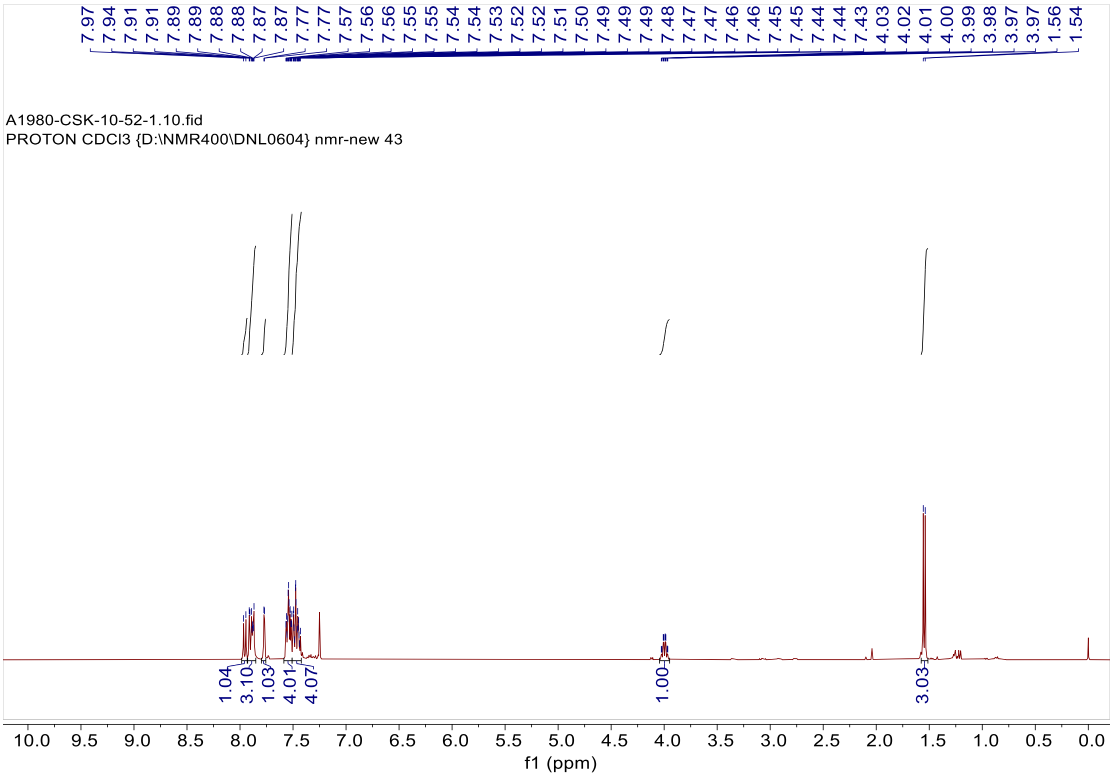

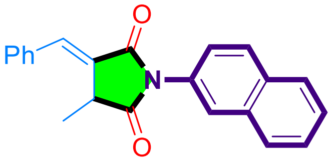

6s

^{1}H NMR (400 MHz, $CDCl_3$)

^{13}C NMR (100 MHz, $CDCl_3$)

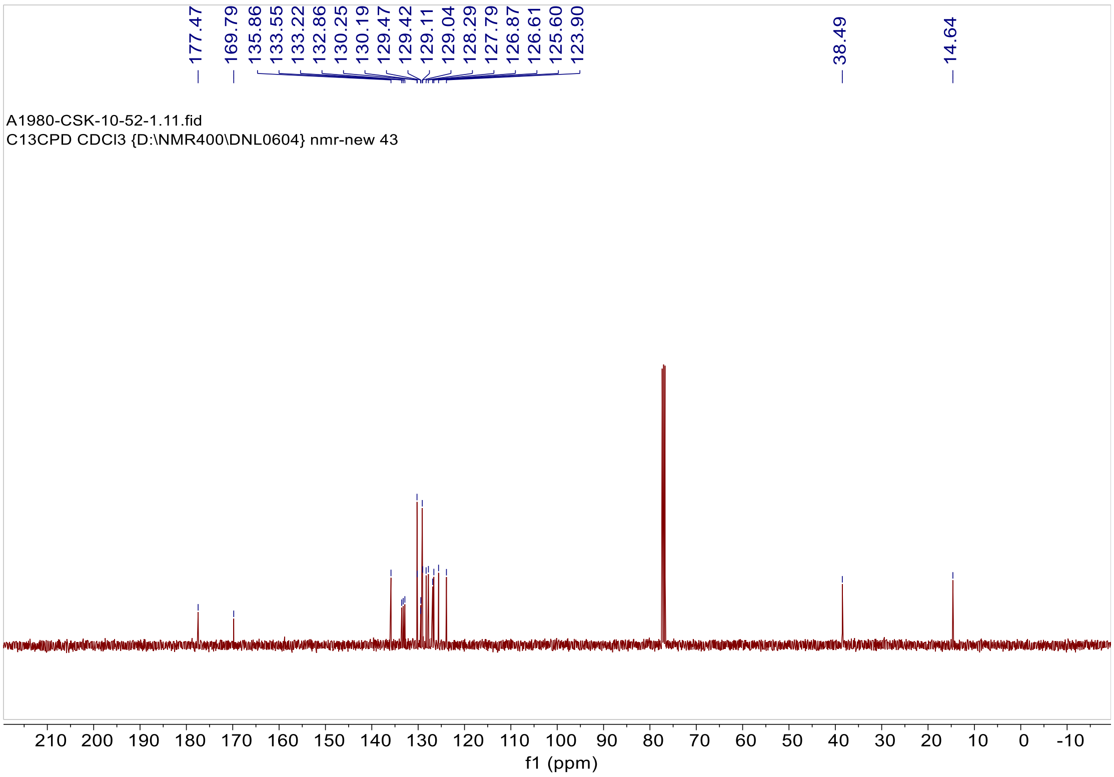

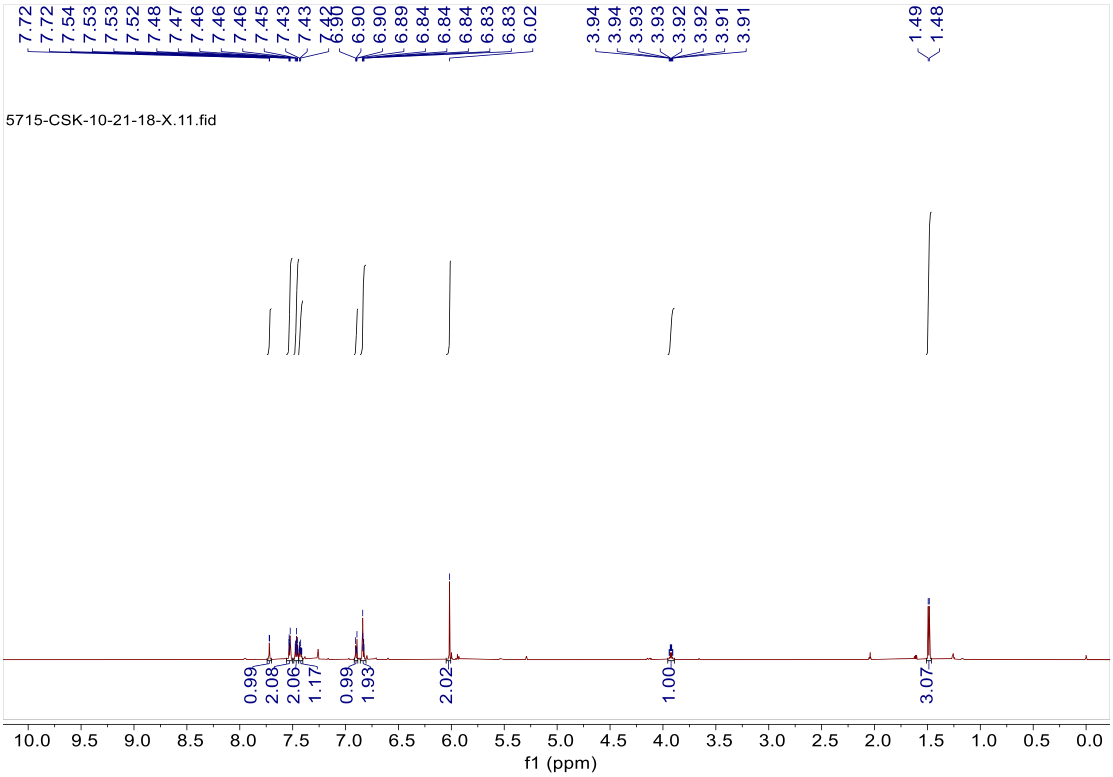

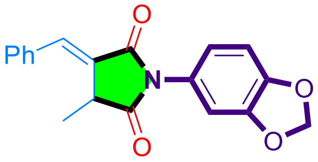

6t

^{1}H NMR (700 MHz, $CDCl_3$)

^{13}C NMR (176 MHz, $CDCl_3$)

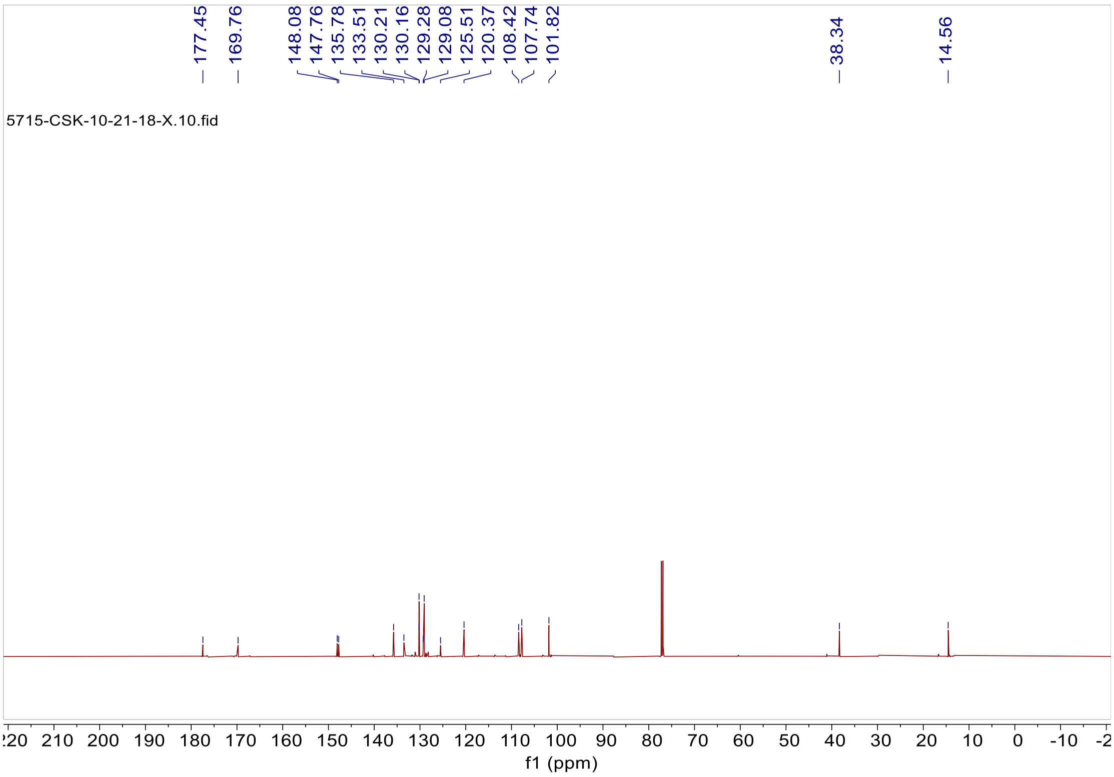

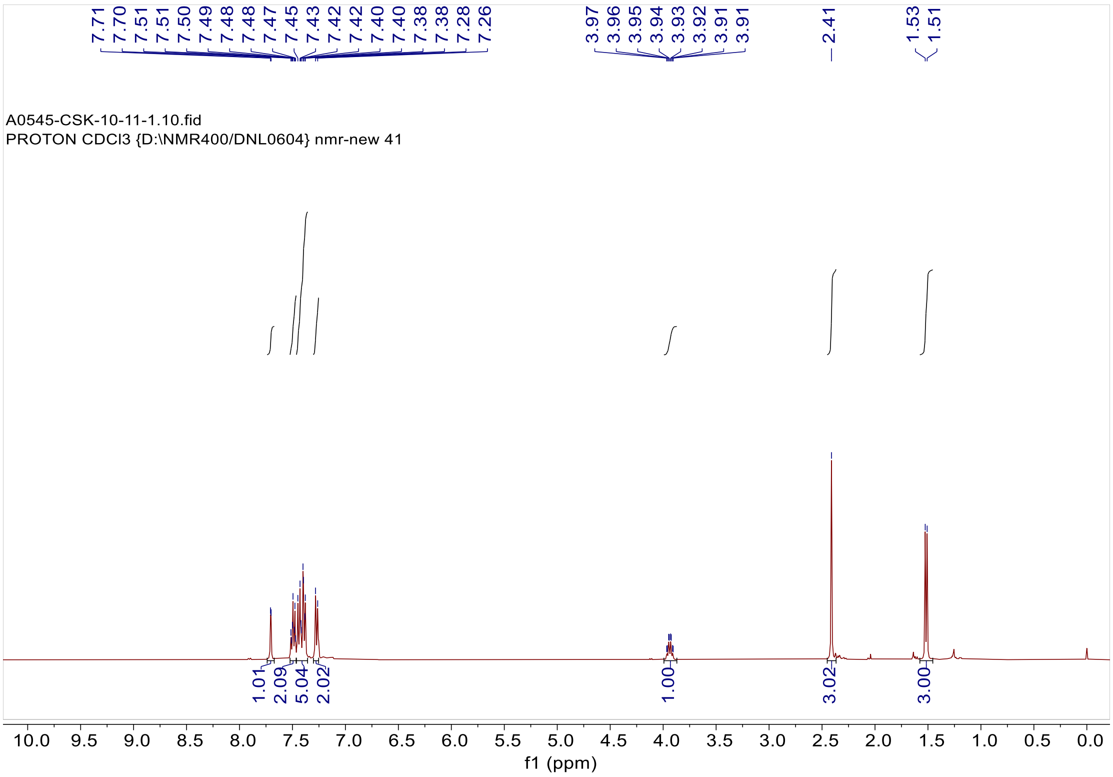

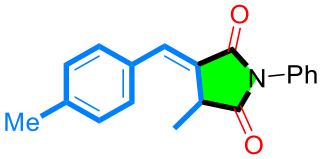

6u

^{1}H NMR (400 MHz, $CDCl_3$)
^{13}C NMR (100 MHz, $CDCl_3$)

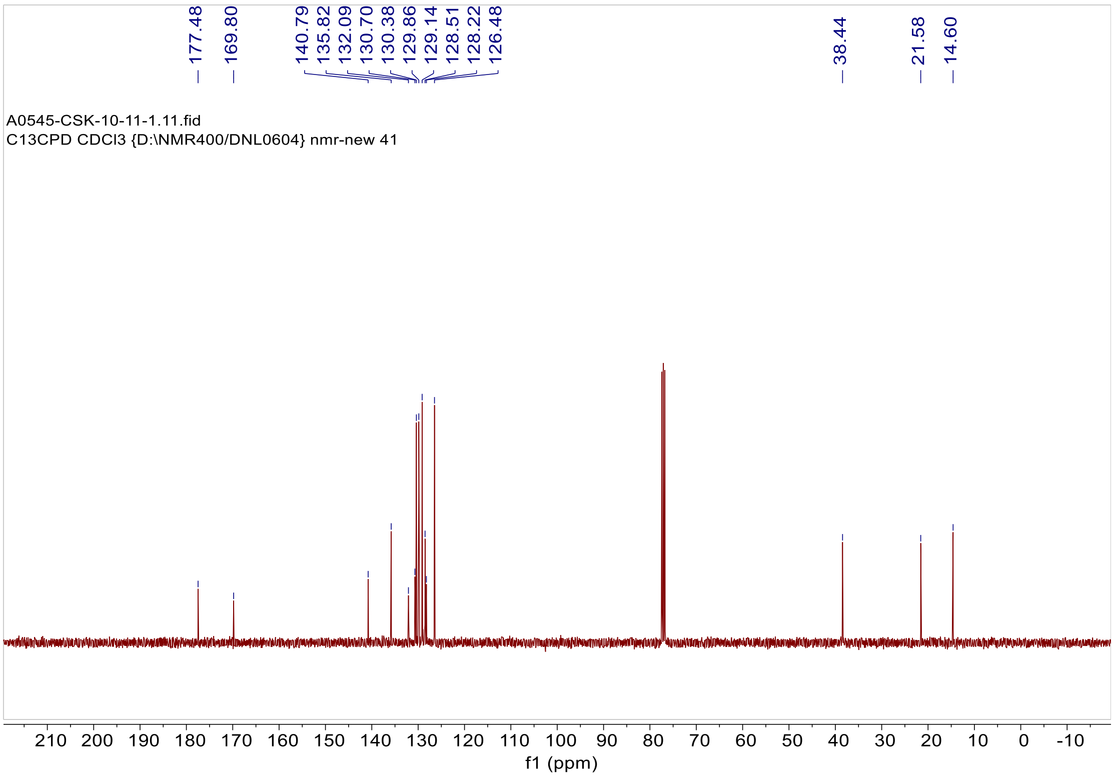

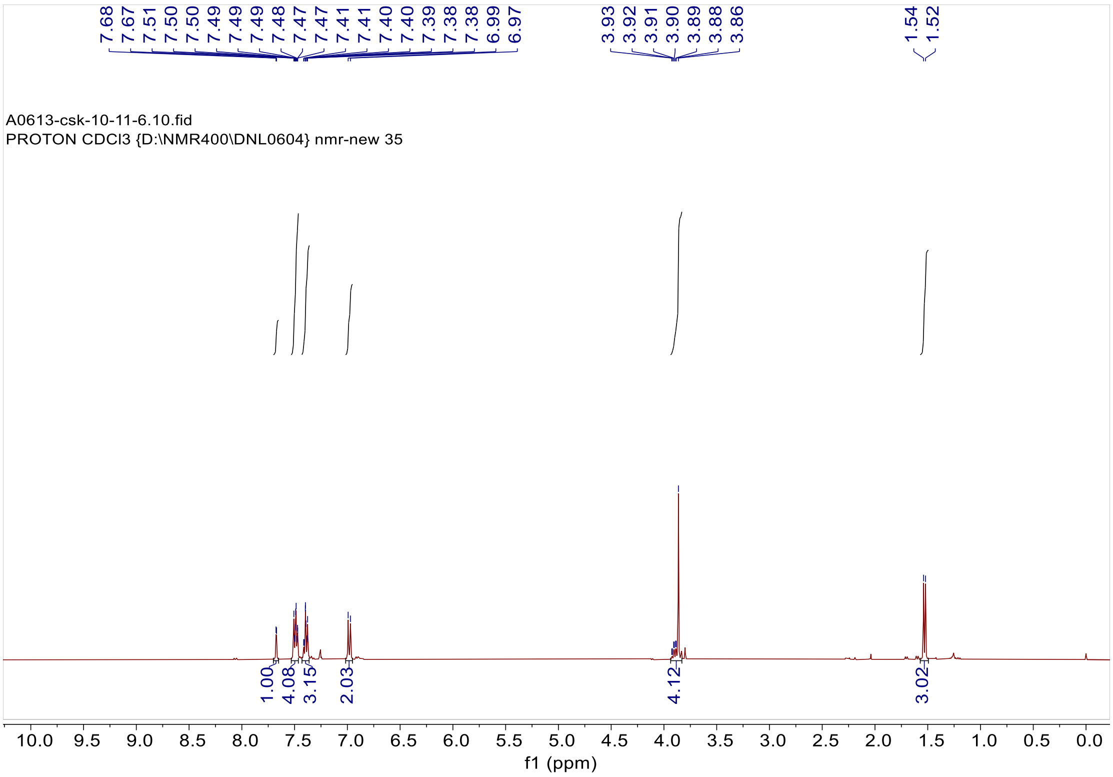

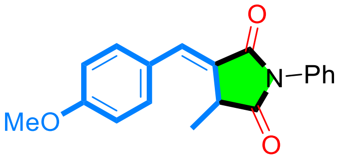

6v

^{1}H NMR (400 MHz, $CDCl_3$)

^{13}C NMR (100 MHz, $CDCl_3$)

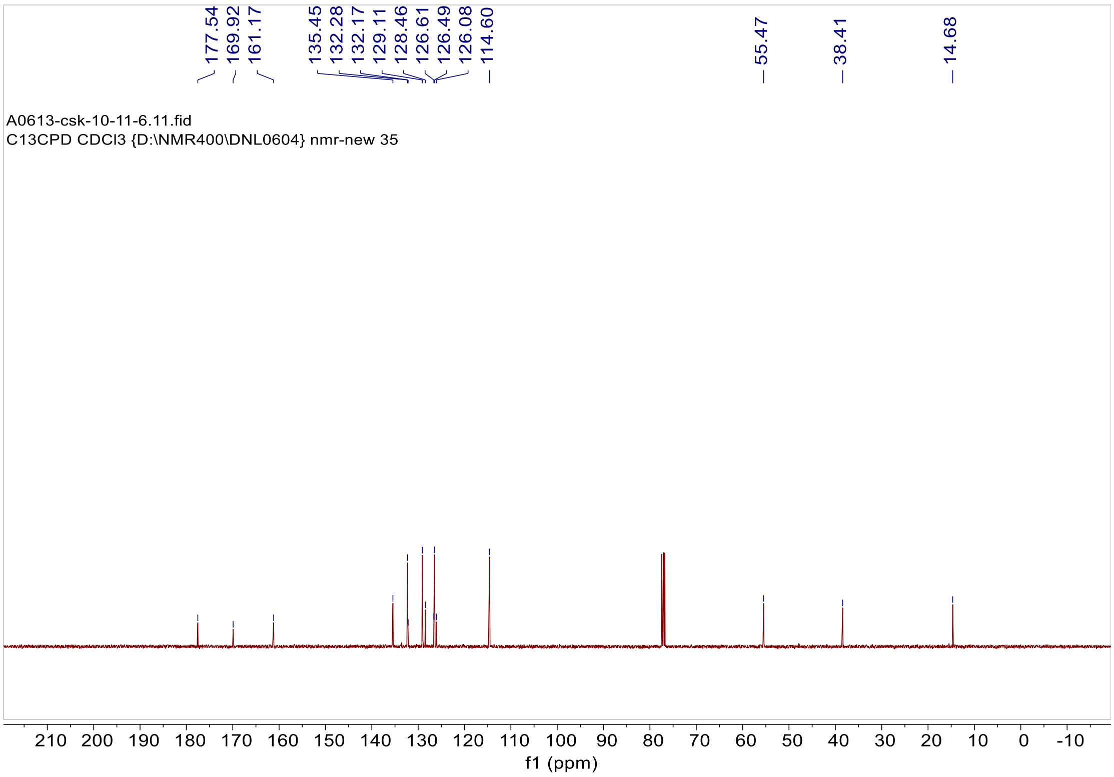

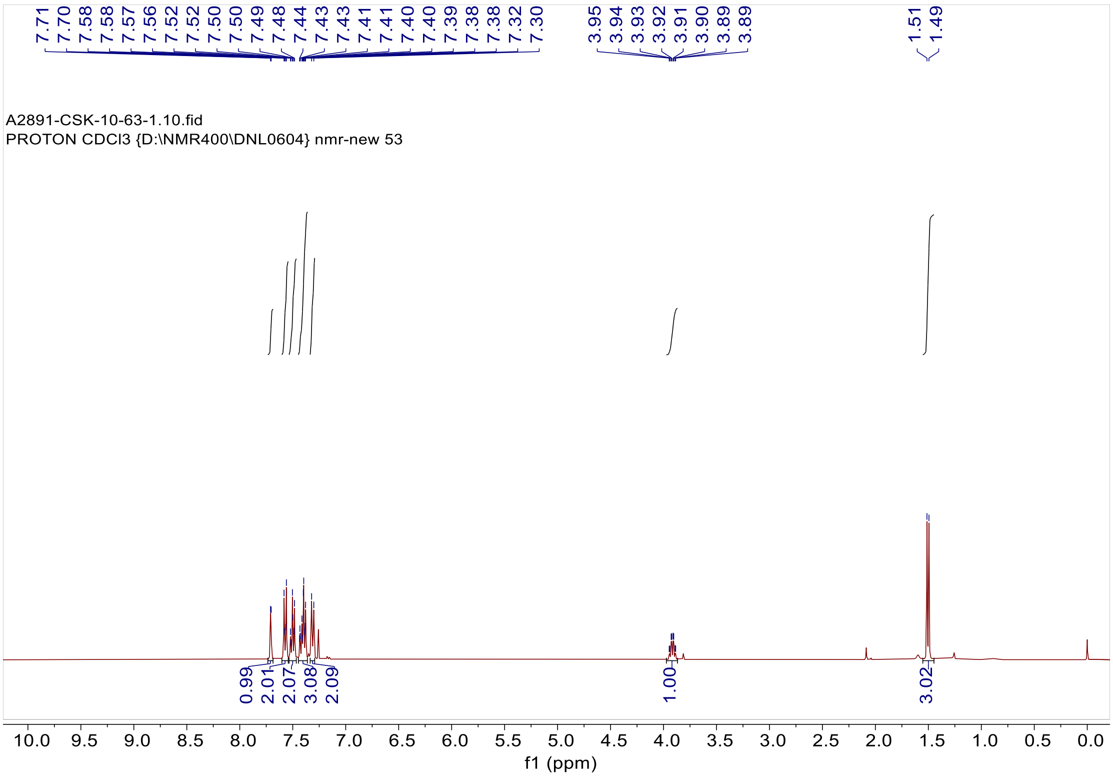

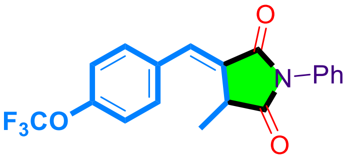

6w

^{1}H NMR (400 MHz, $CDCl_3$)
^{13}C NMR (100 MHz, $CDCl_3$)

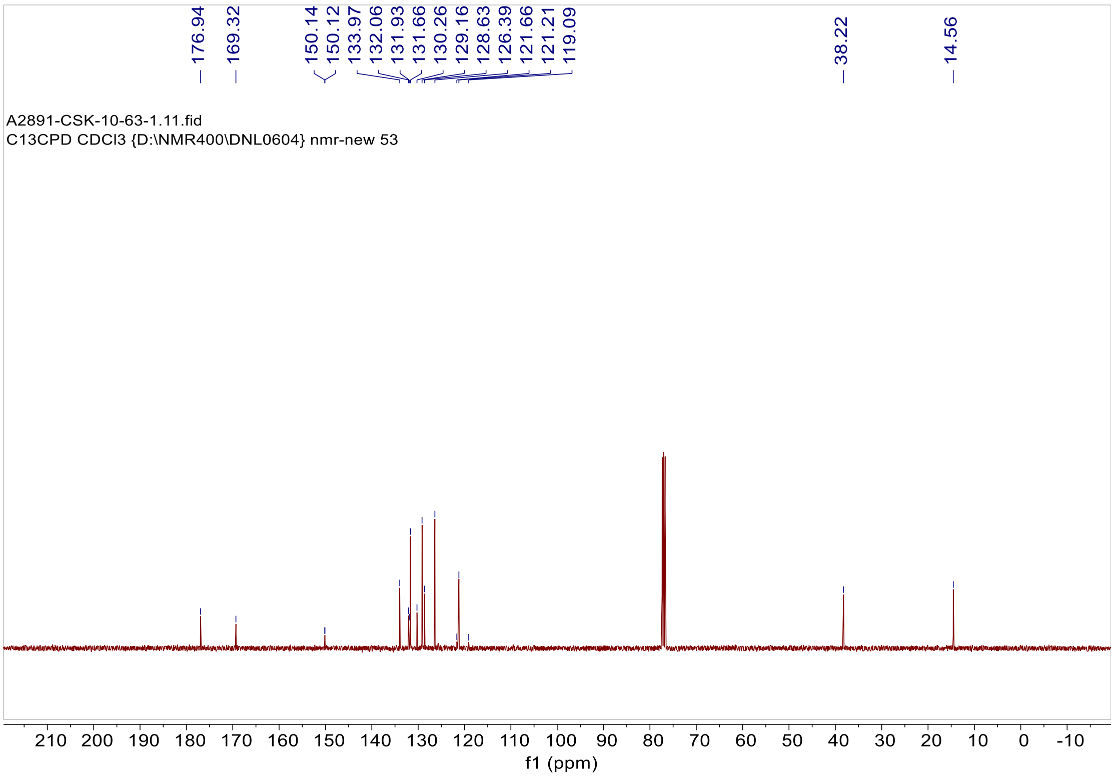

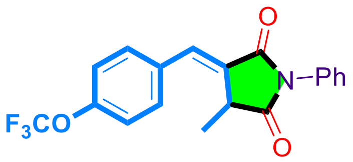

6w

^{19}F NMR (376 MHz, $CDCl_3$)

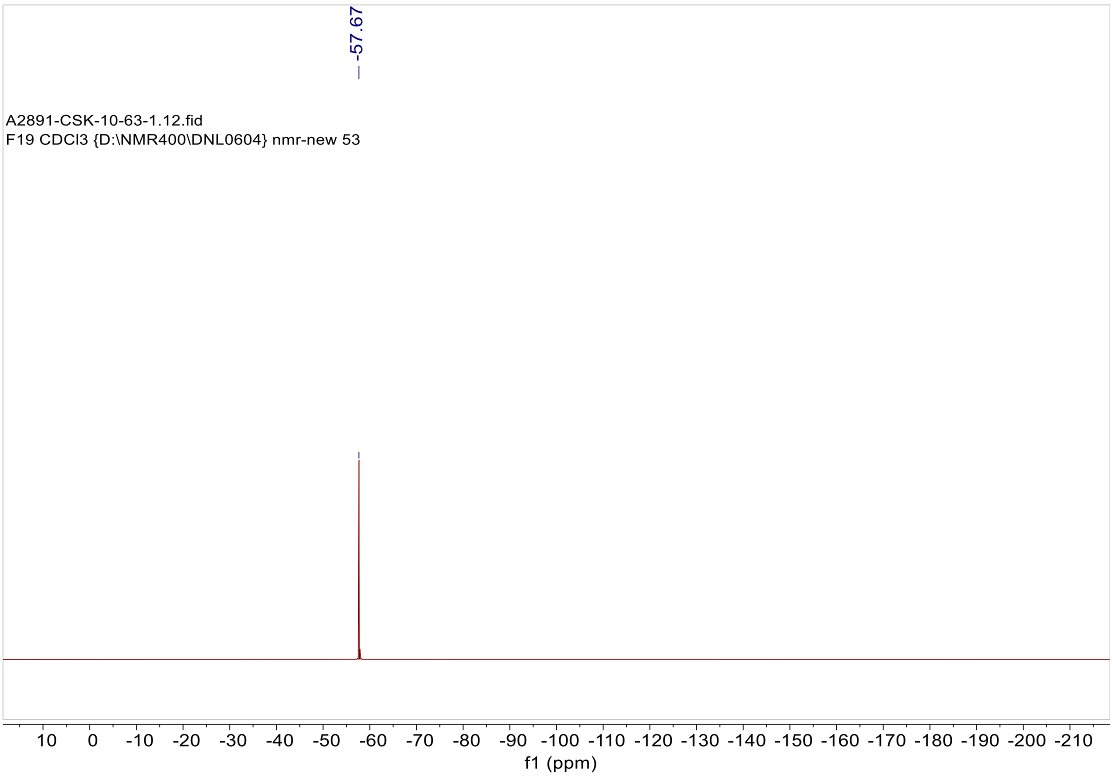

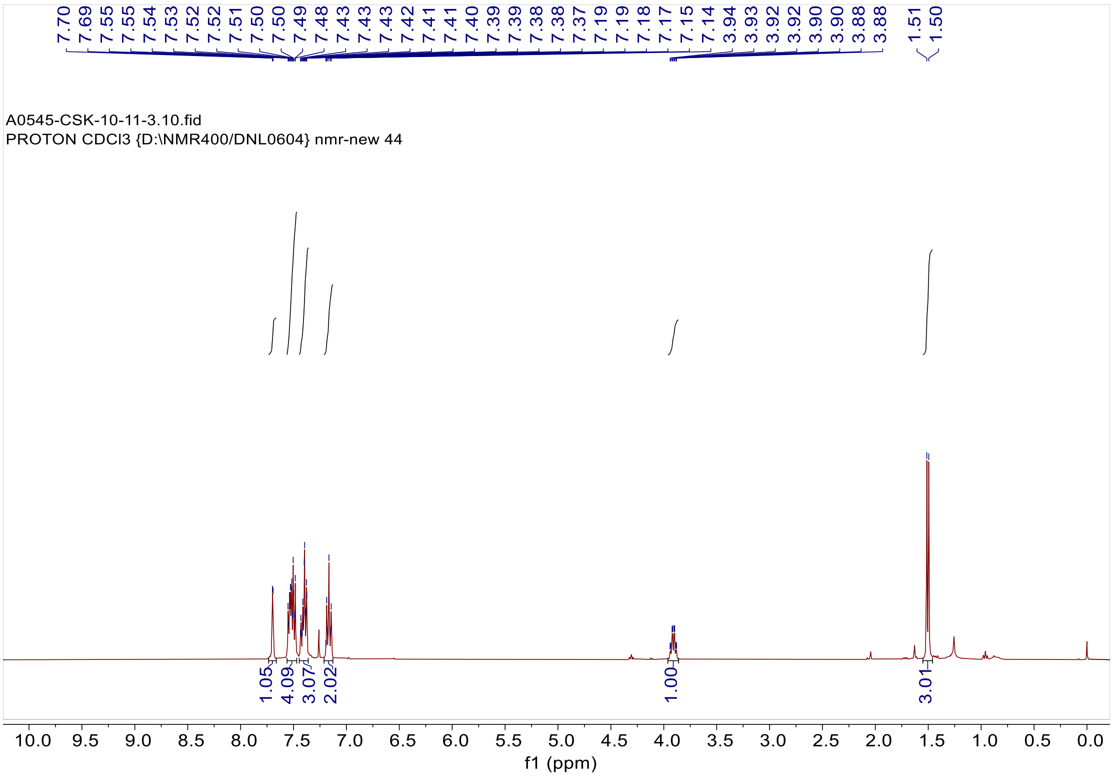

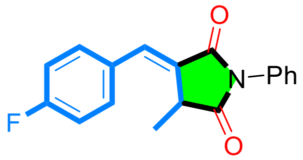

6x

^{1}H NMR (400 MHz, $CDCl_3$)
^{13}C NMR (100 MHz, $CDCl_3$)

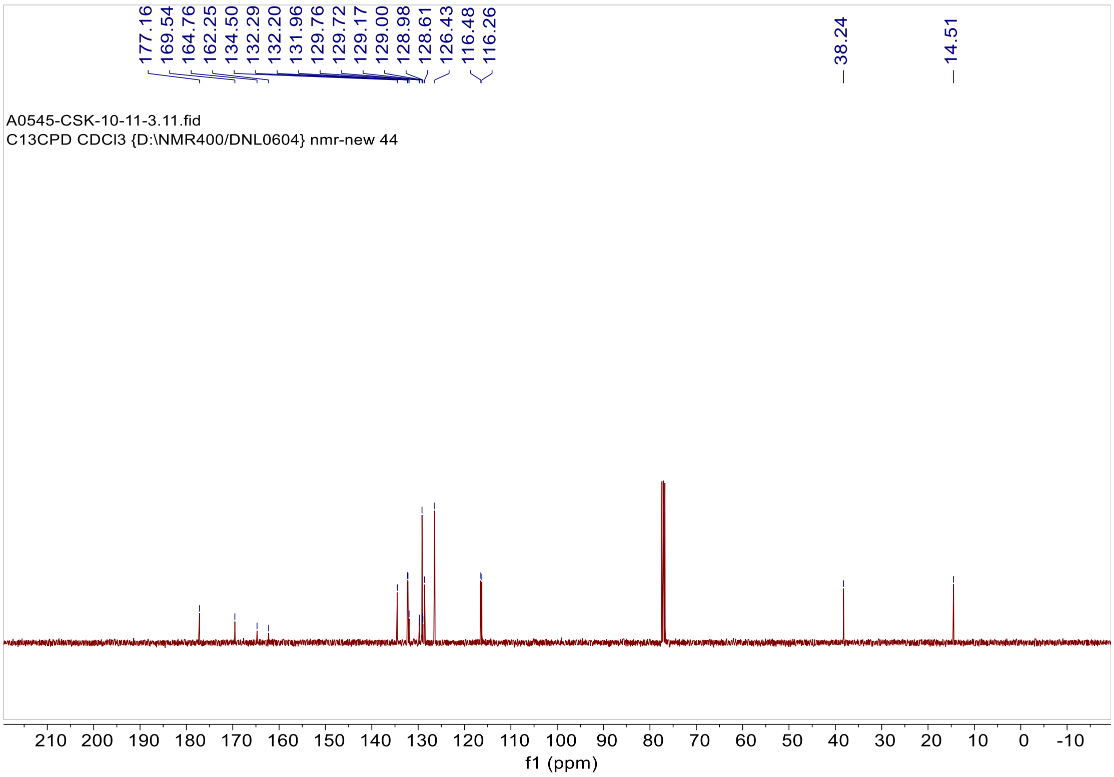

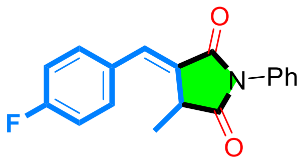

6x

^{19}F NMR (376 MHz, $CDCl_3$)

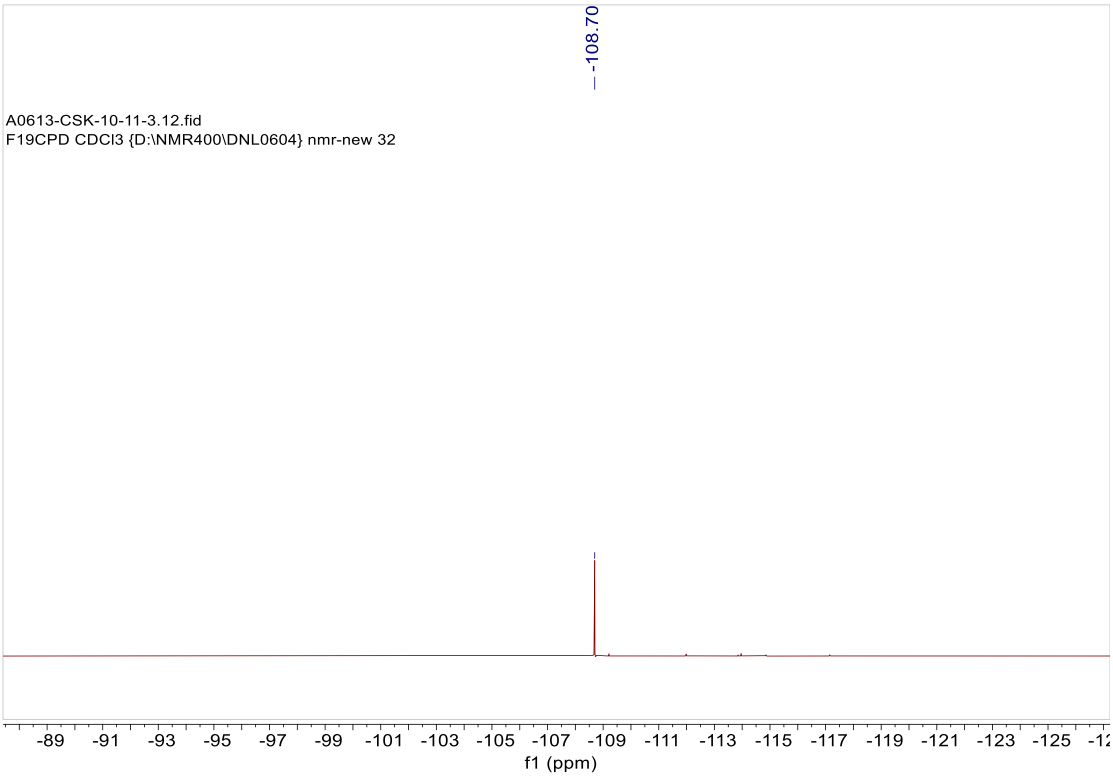

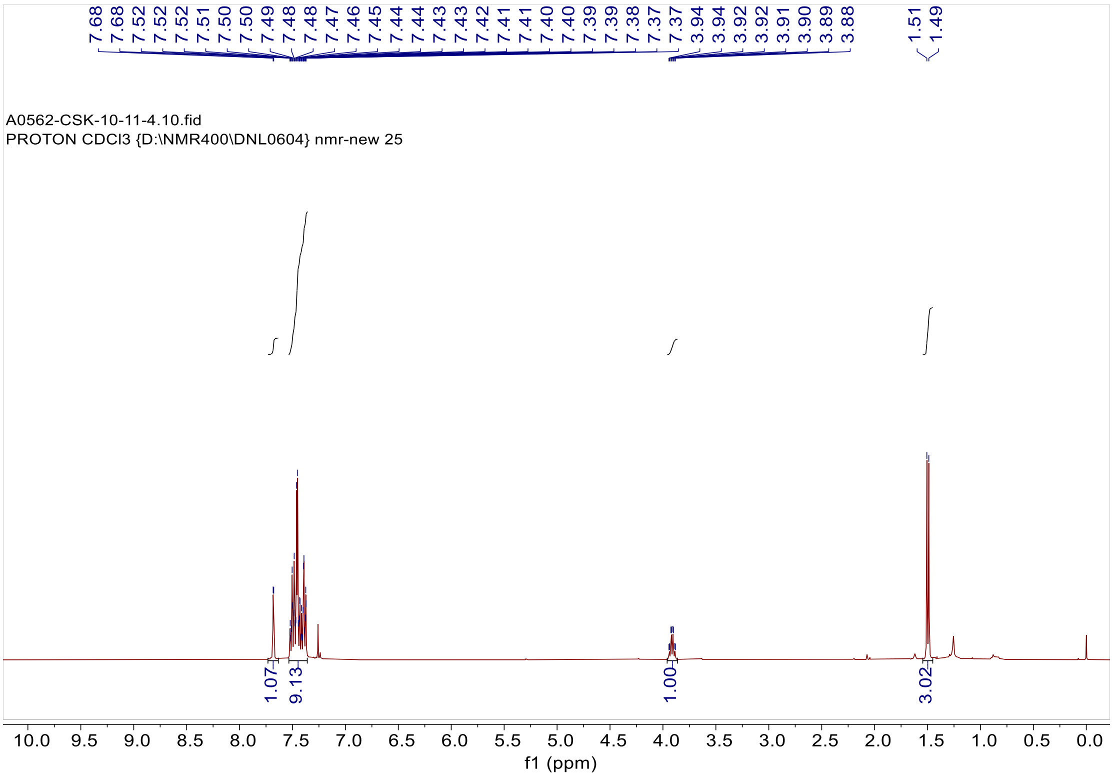

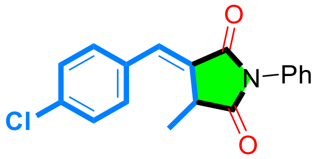

6y

^{1}H NMR (400 MHz, $CDCl_3$)
^{13}C NMR (100 MHz, $CDCl_3$)

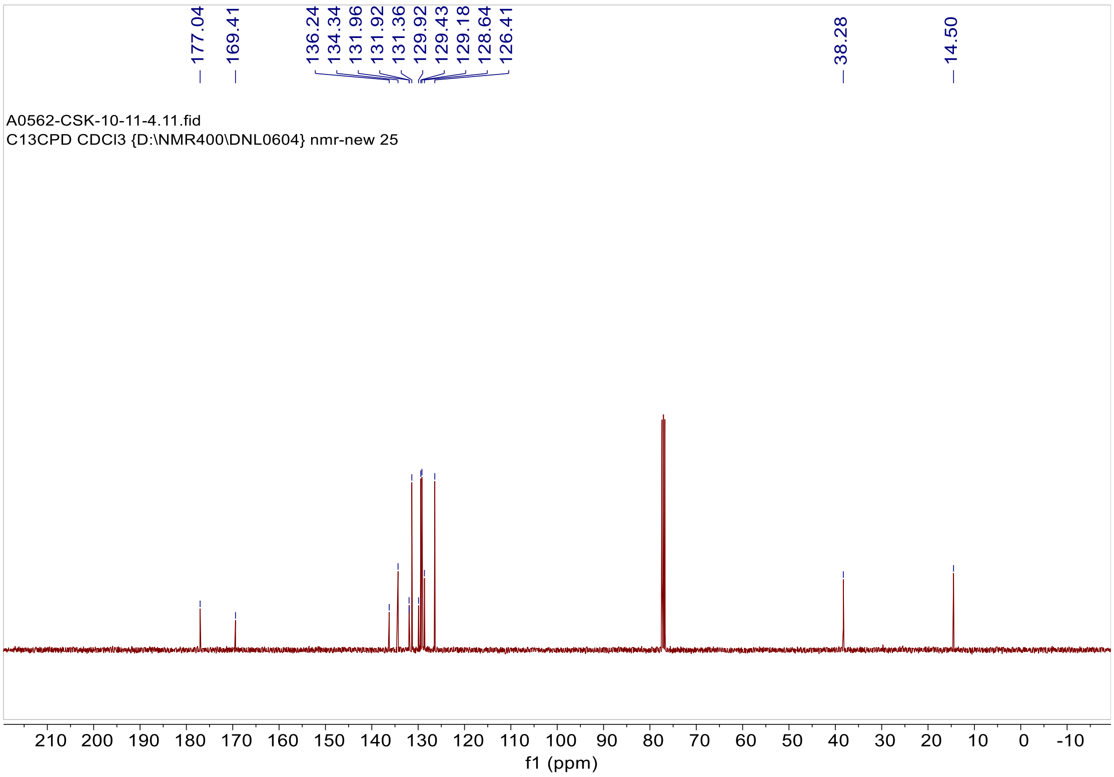

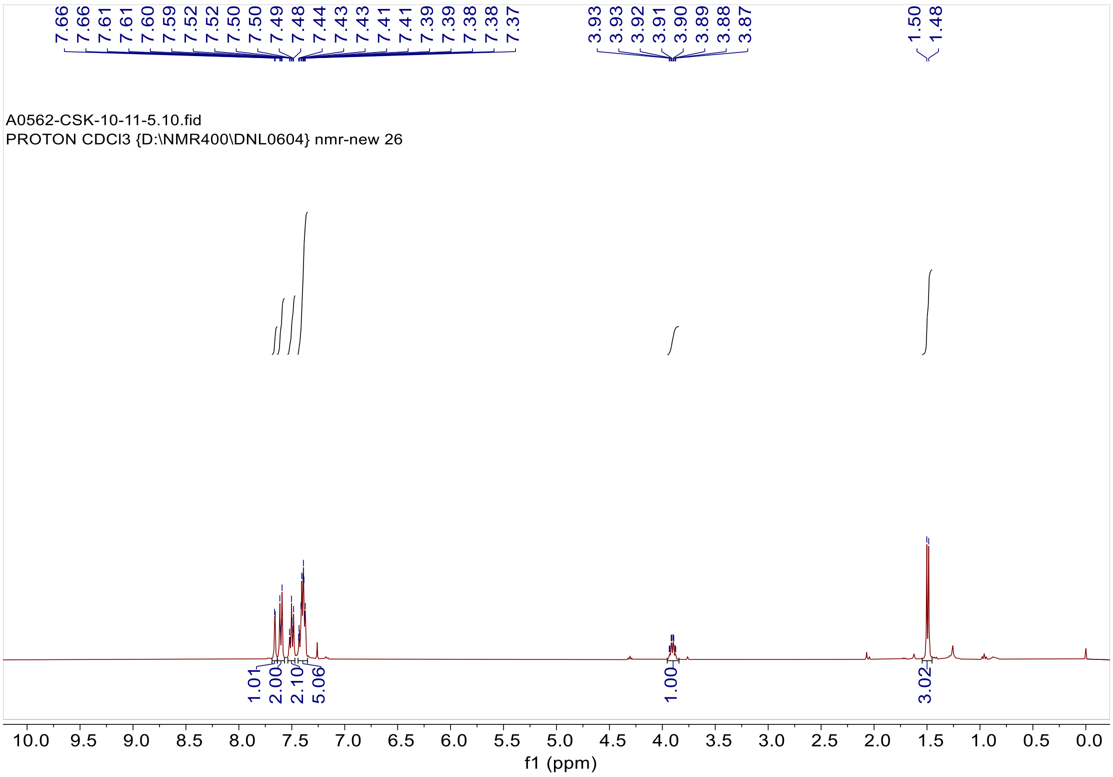

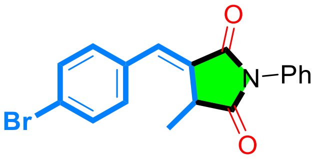

6z

^{1}H NMR (400 MHz, $CDCl_3$)
^{13}C NMR (100 MHz, $CDCl_3$)

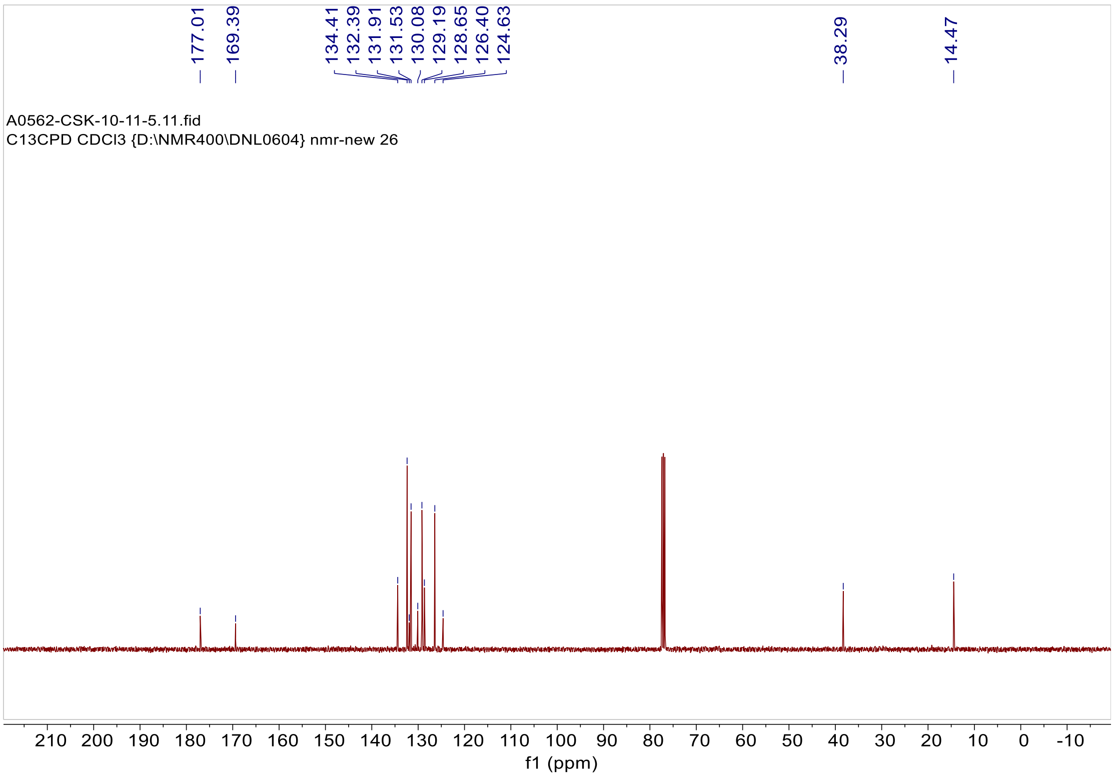

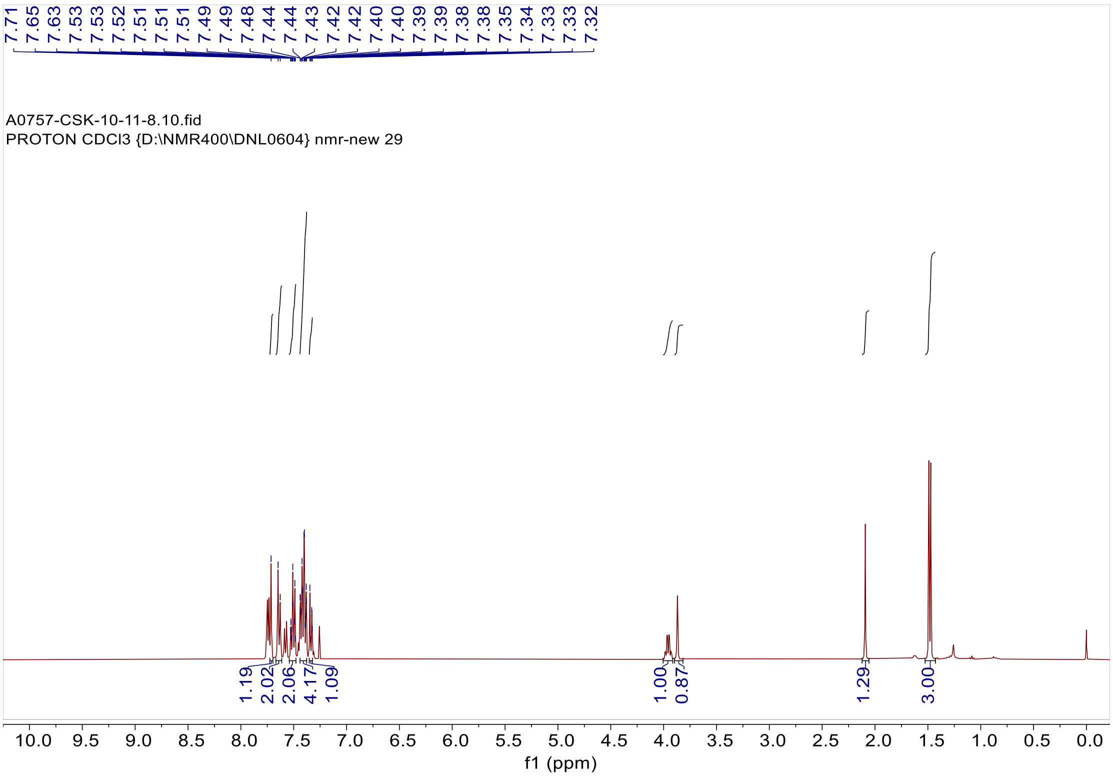

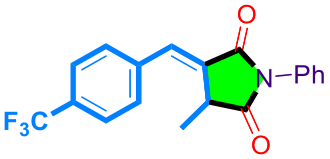

6aa

^{1}H NMR (400 MHz, $CDCl_3$)
^{13}C NMR (100 MHz, $CDCl_3$)

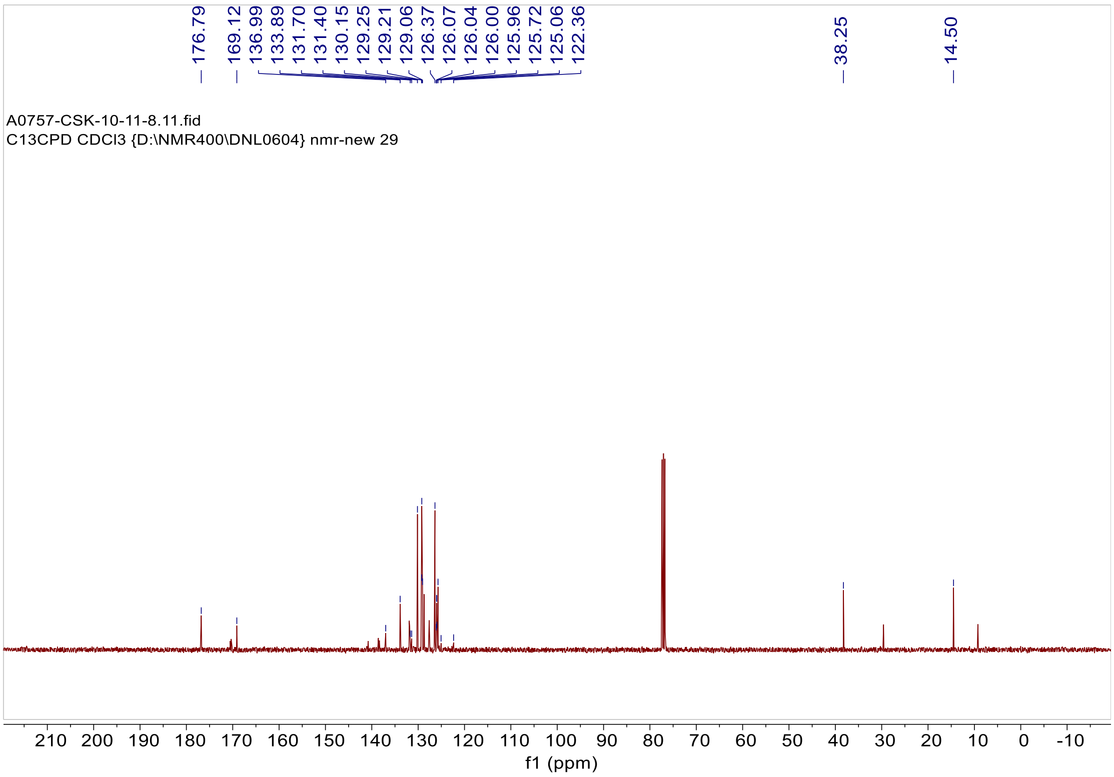

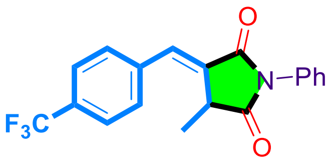

6aa

^{19}F NMR (376 MHz, $CDCl_3$)

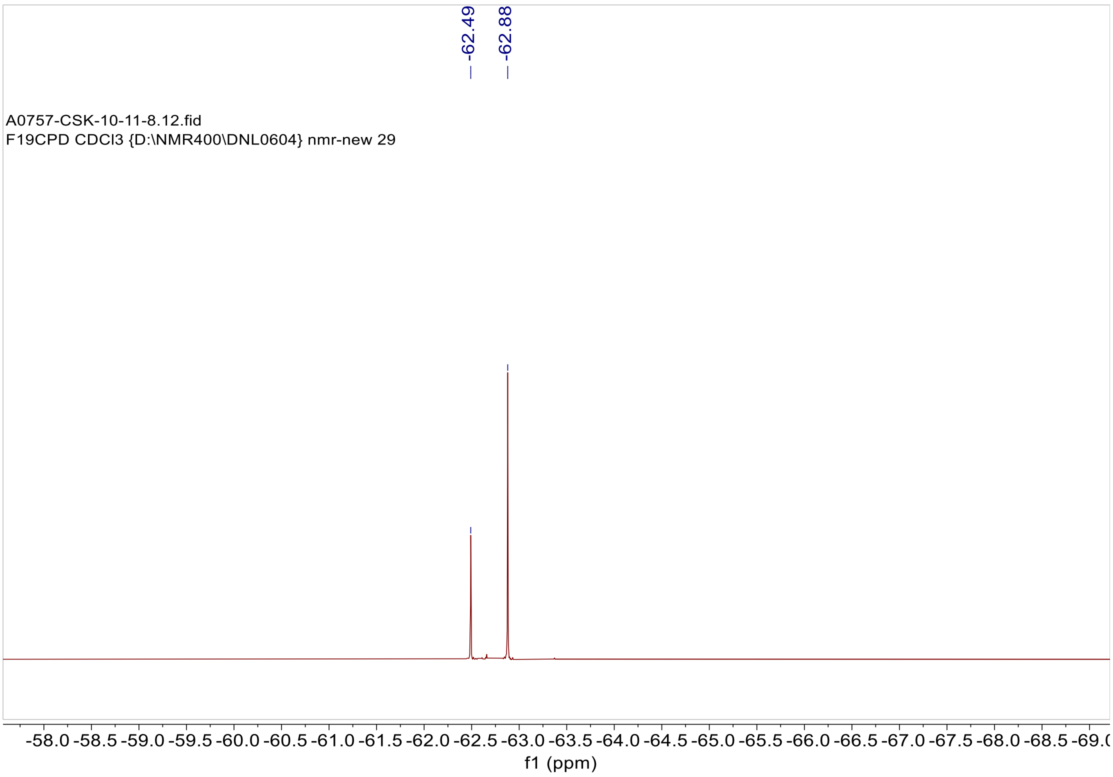

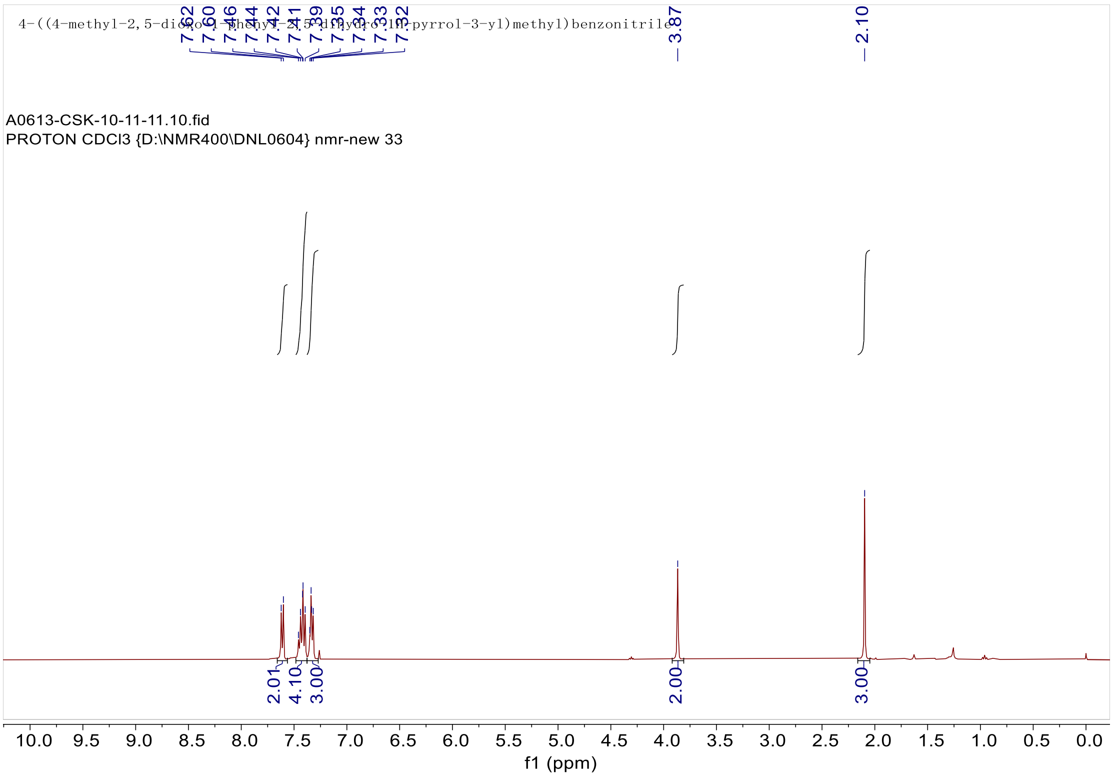

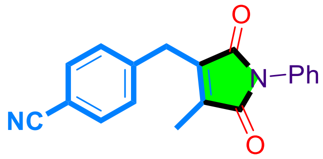

6ac'

^{1}H NMR (400 MHz, $CDCl_3$)

^{13}C NMR (100 MHz, $CDCl_3$)

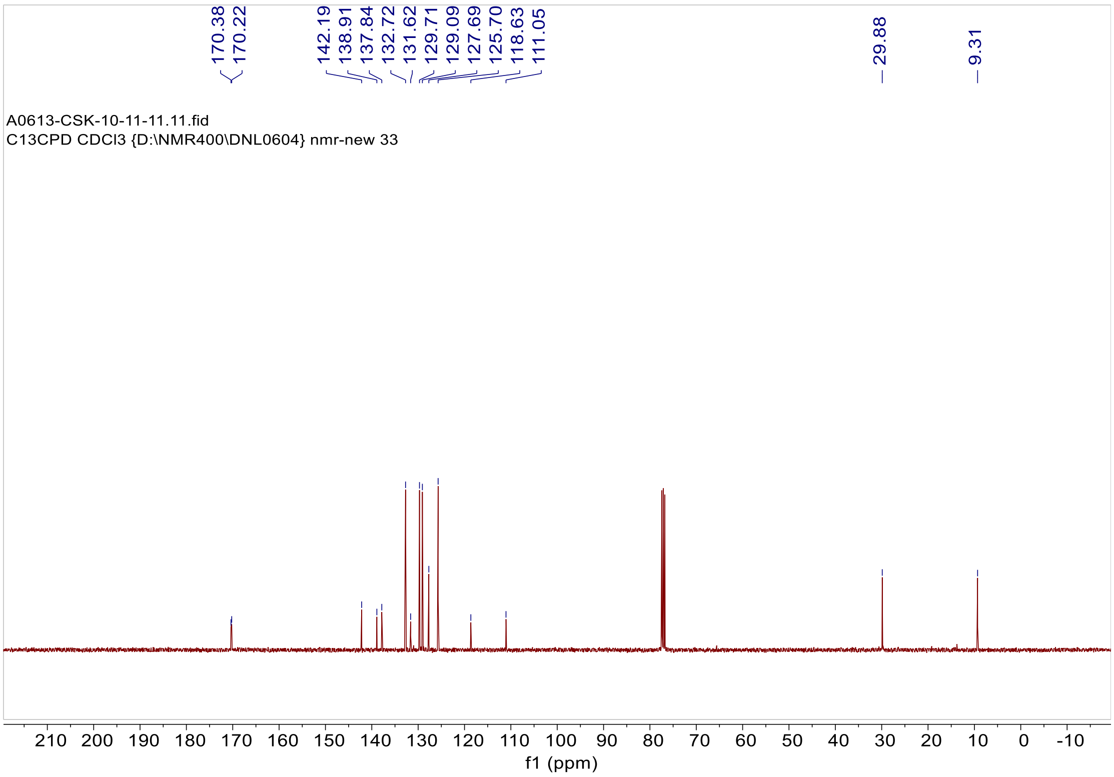

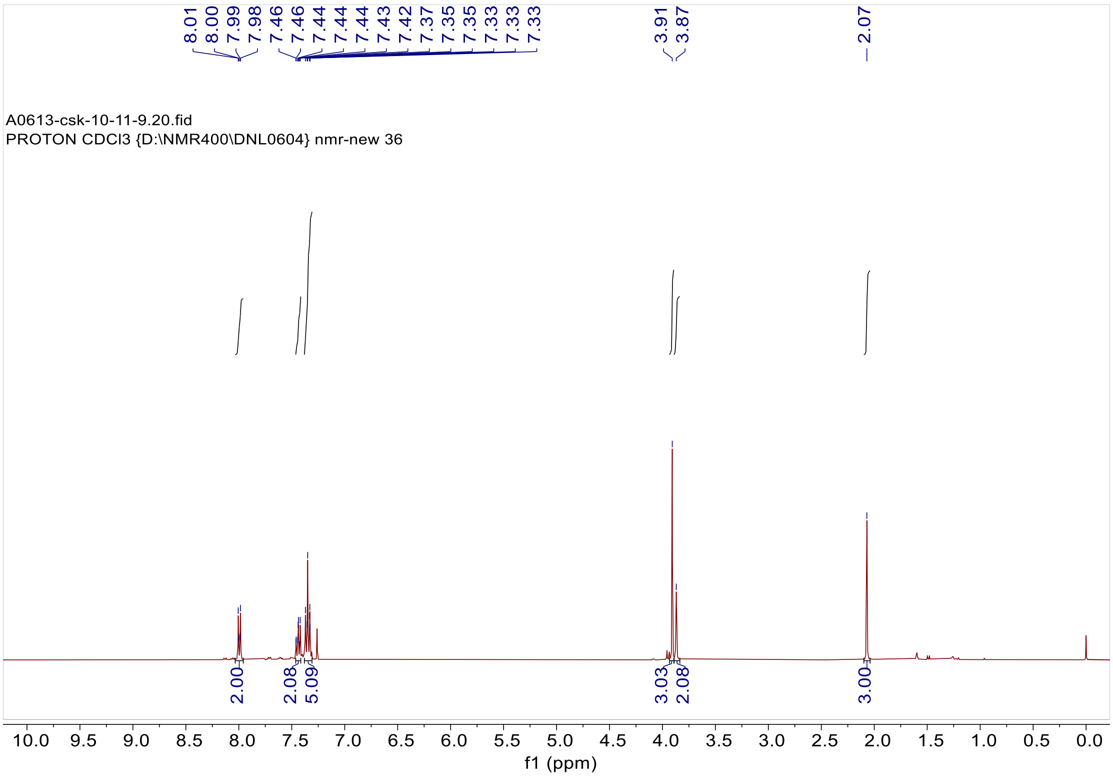

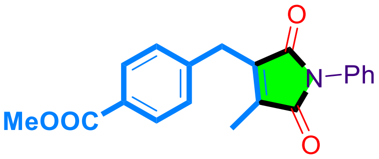

6ad'

^{1}H NMR (400 MHz, $CDCl_3$)

^{13}C NMR (100 MHz, $CDCl_3$)

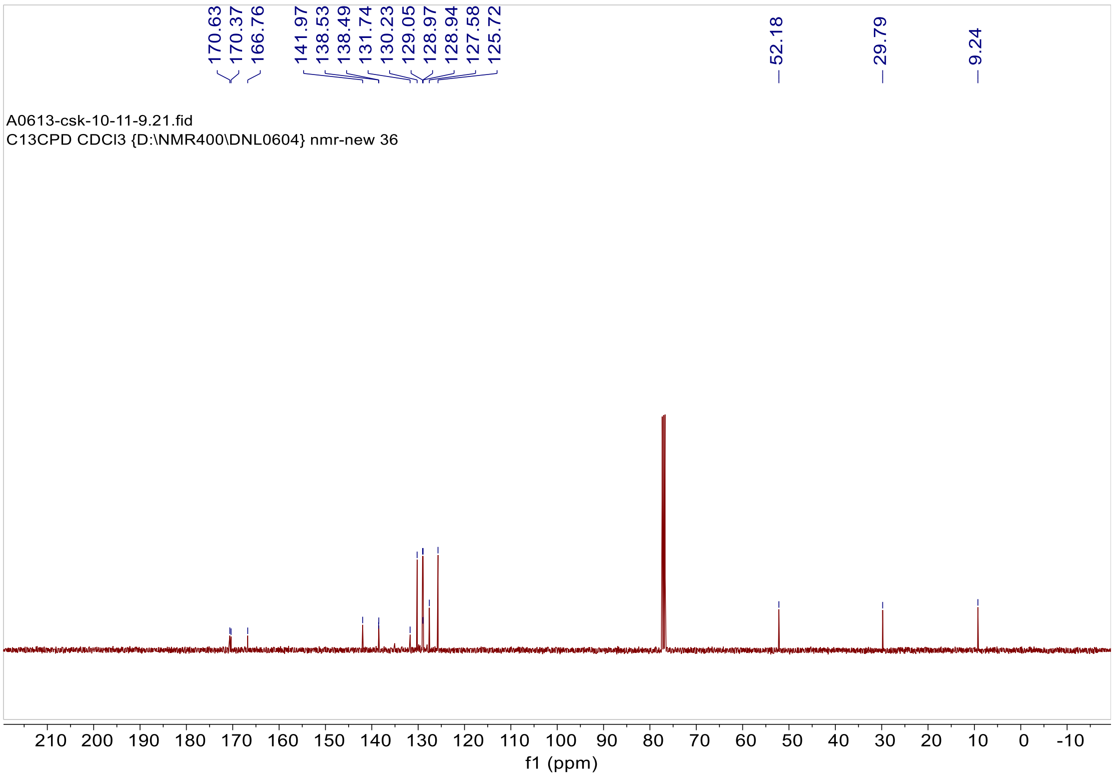

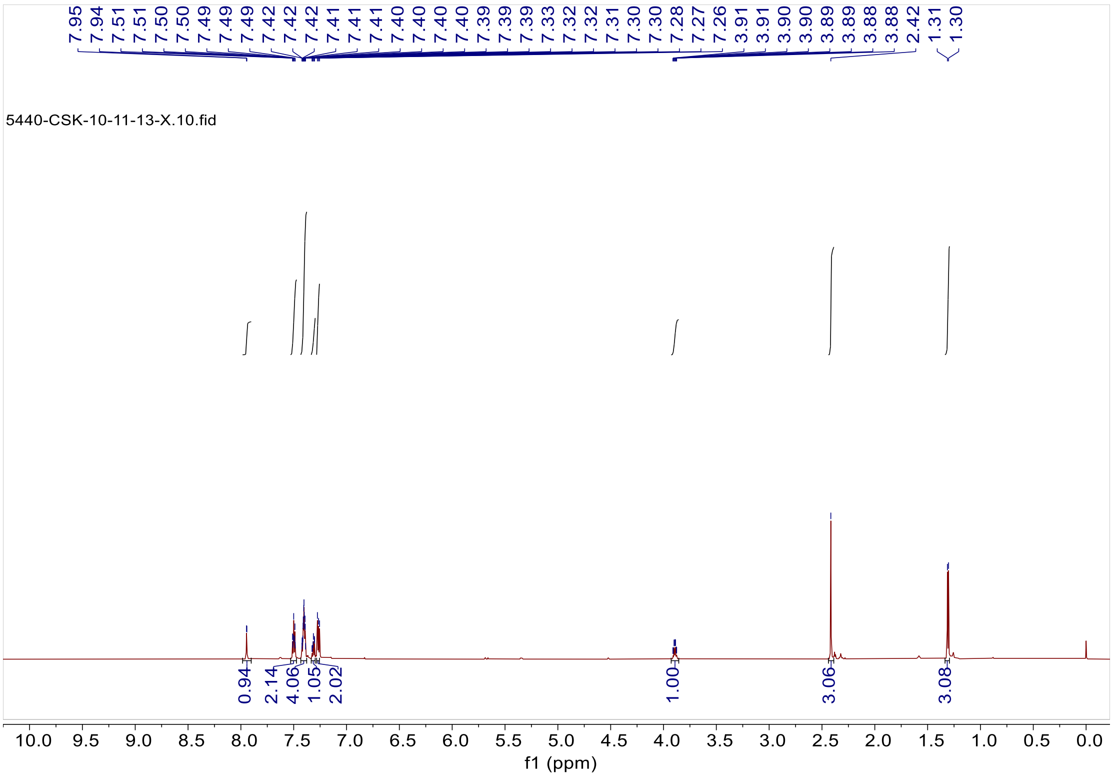

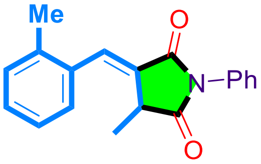

6ae

^{1}H NMR (700 MHz, $CDCl_3$)

^{13}C NMR (176 MHz, $CDCl_3$)

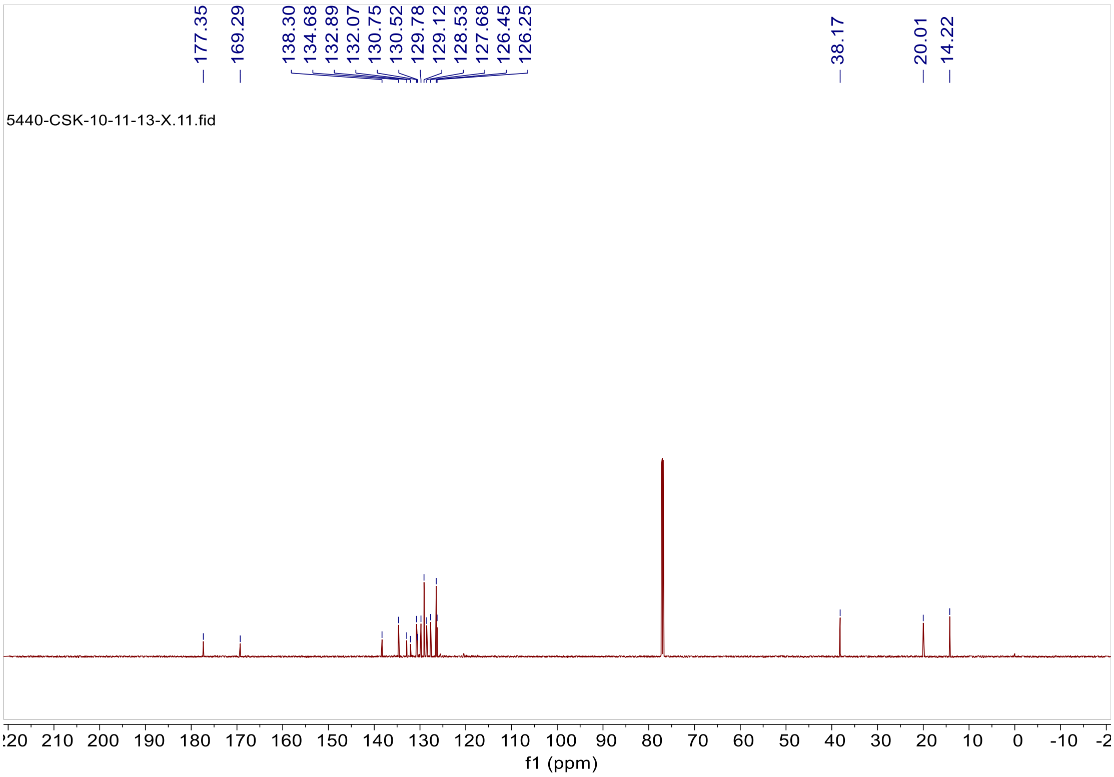

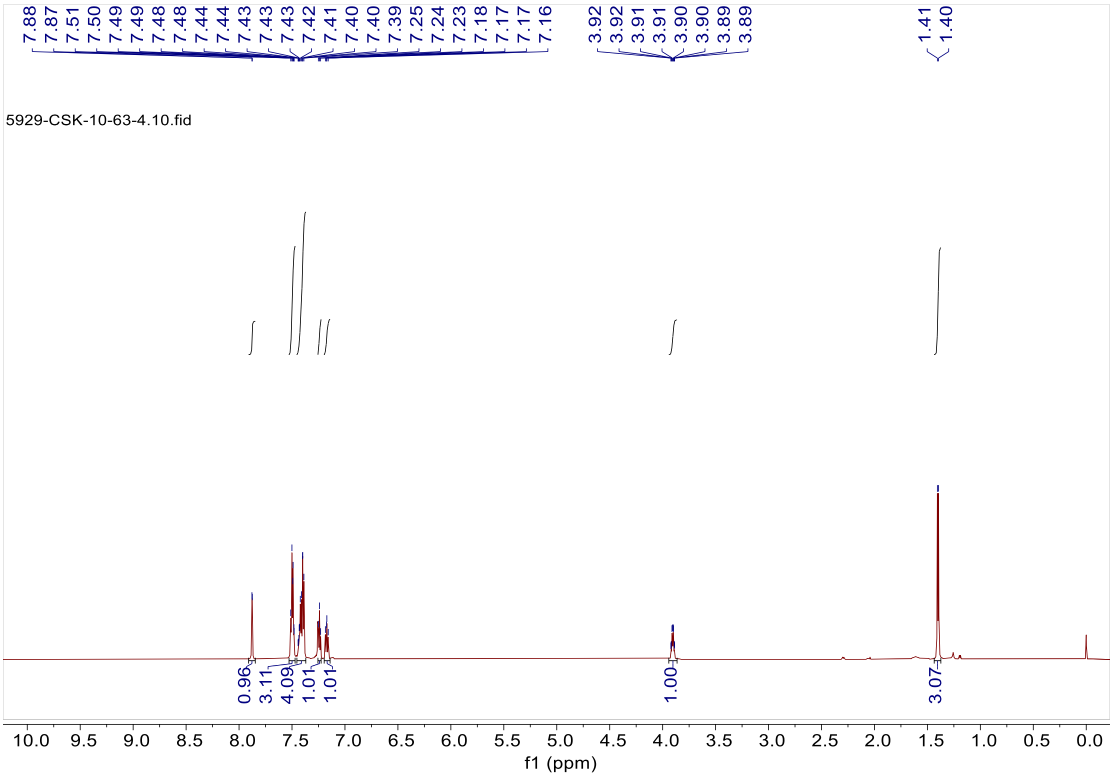

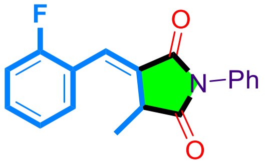

6af

^{1}H NMR (700 MHz, $CDCl_3$)
^{13}C NMR (176 MHz, $CDCl_3$)

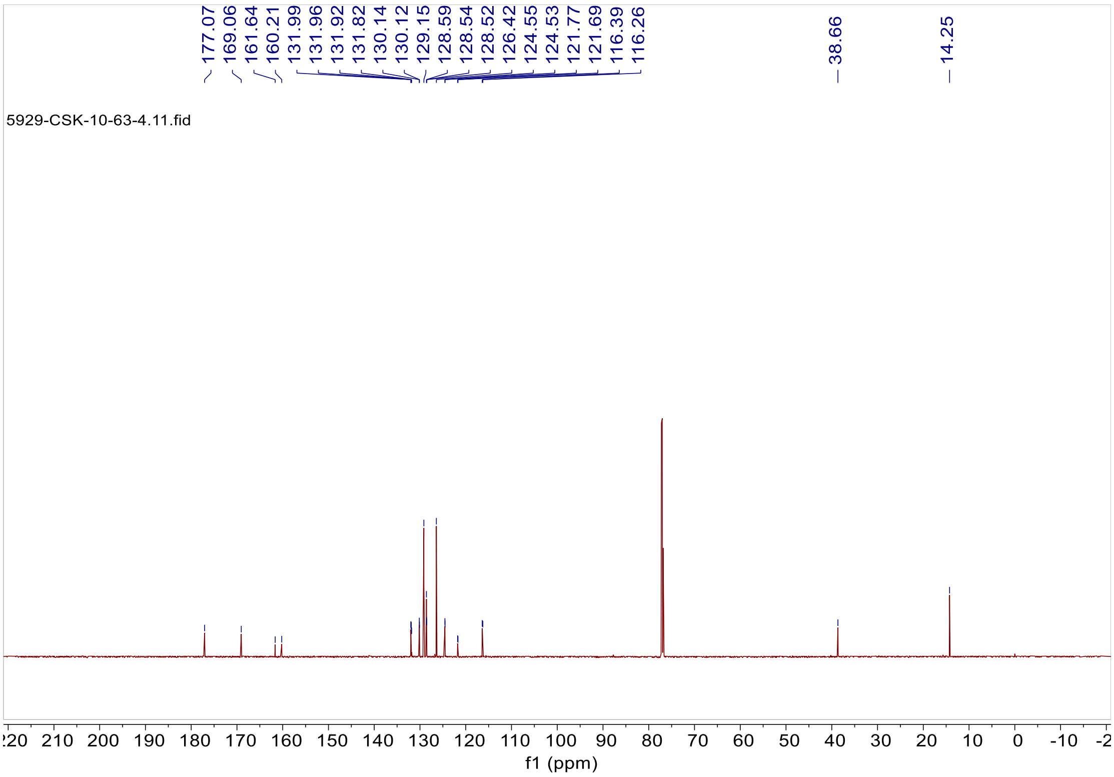

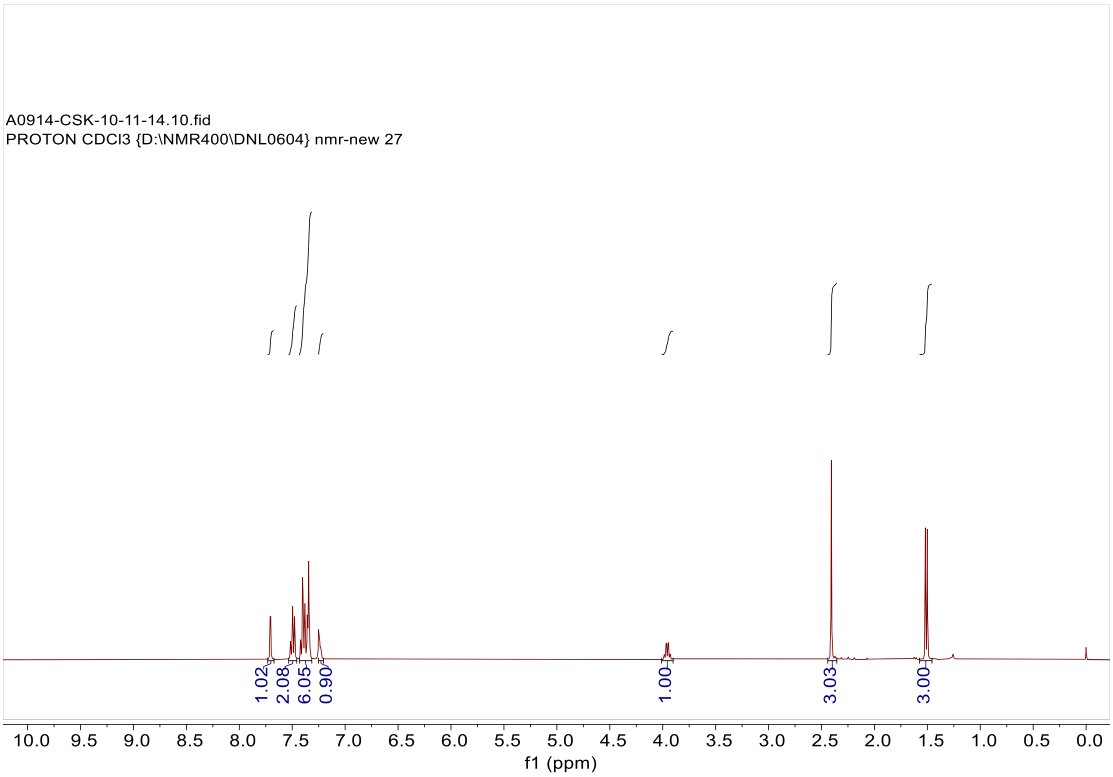

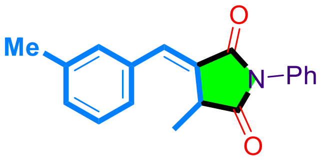

6ag

^{1}H NMR (400 MHz, $CDCl_3$)
^{13}C NMR (100 MHz, $CDCl_3$)

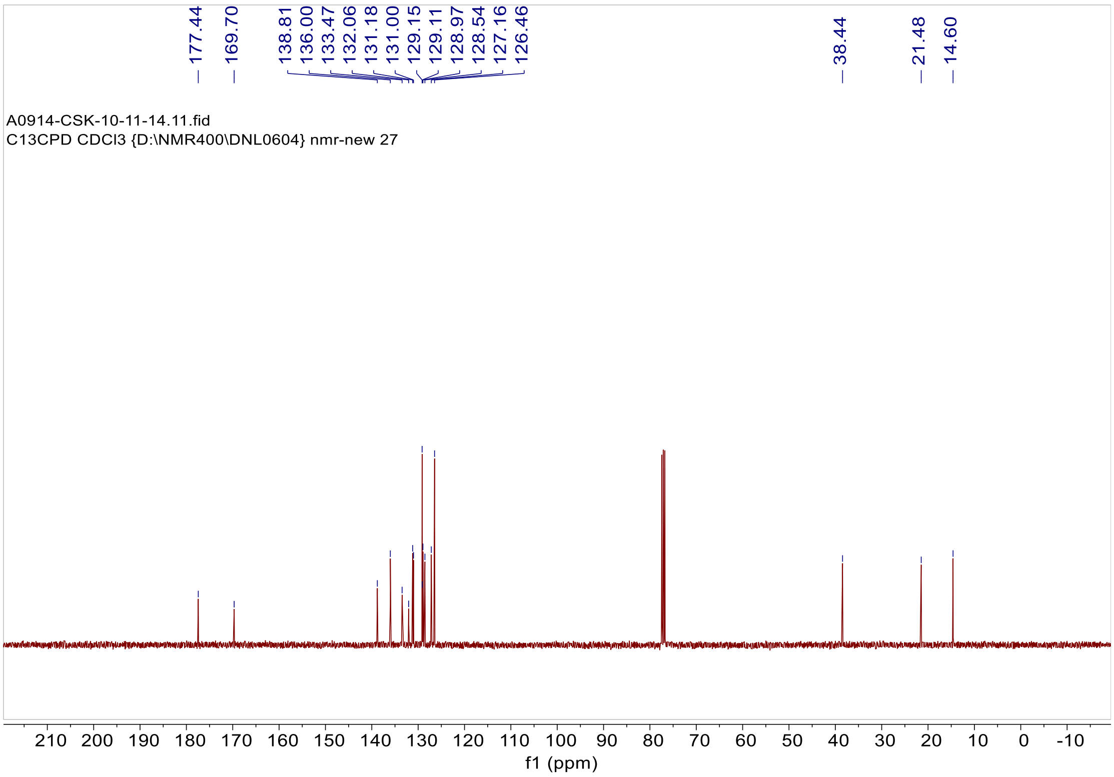

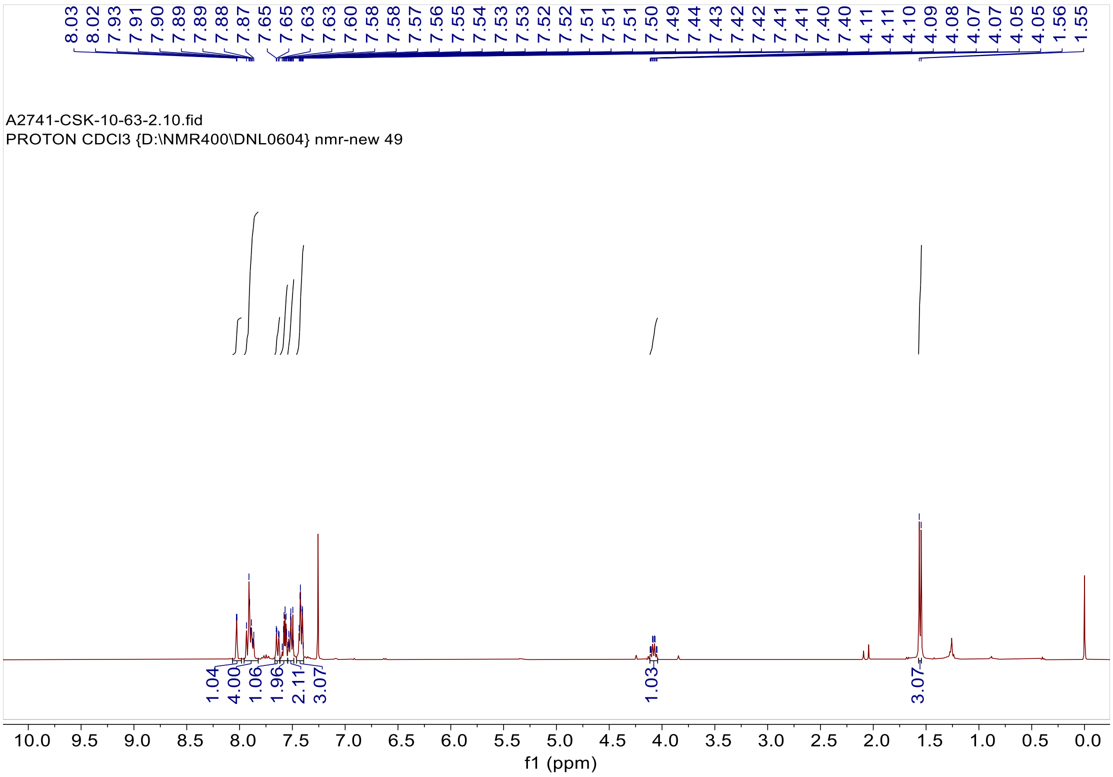

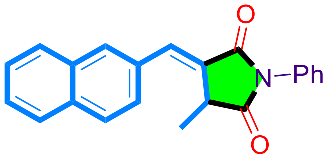

6ah

^{1}H NMR (400 MHz, $CDCl_3$)
^{13}C NMR (100 MHz, $CDCl_3$)

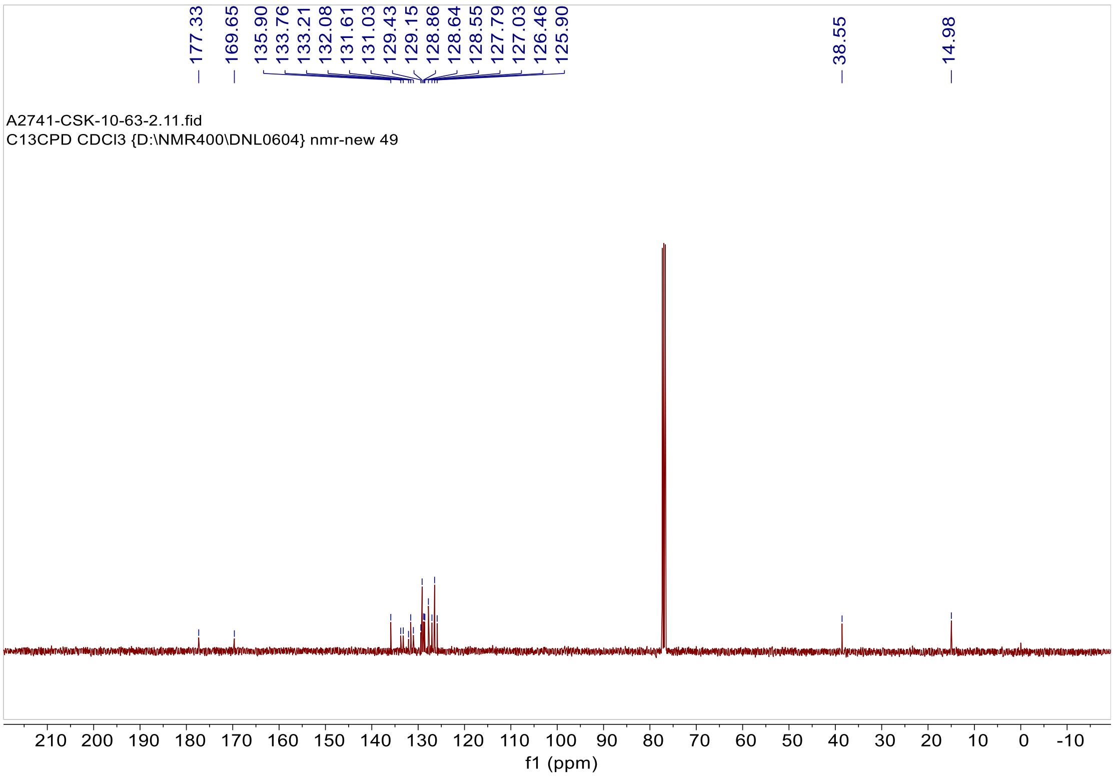

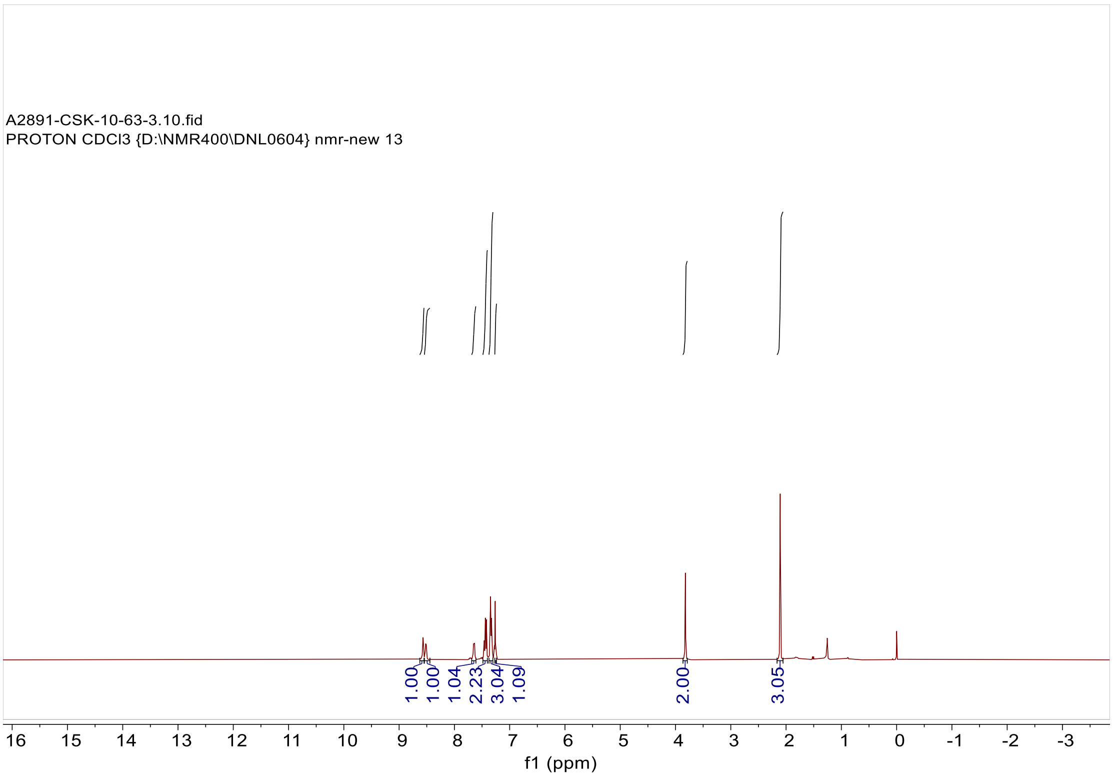

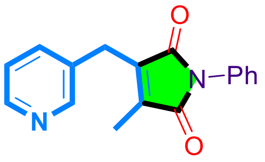

6ai'

^{1}H NMR (400 MHz, $CDCl_3$)
^{13}C NMR (100 MHz, $CDCl_3$)

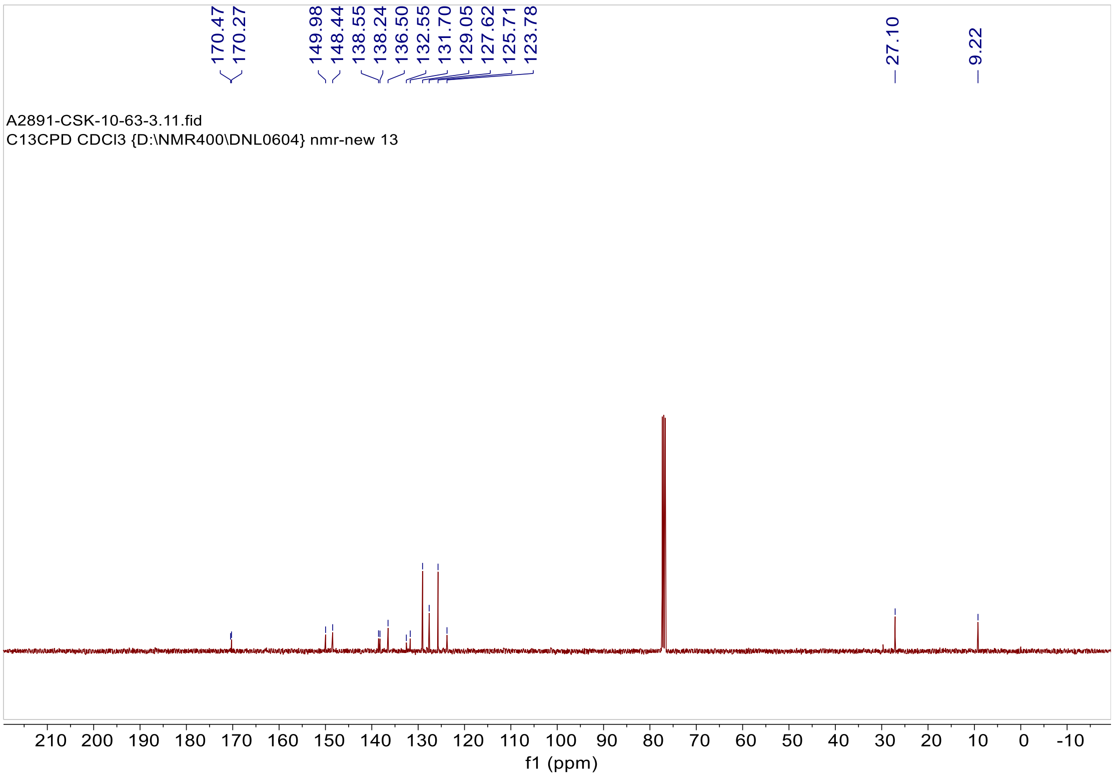

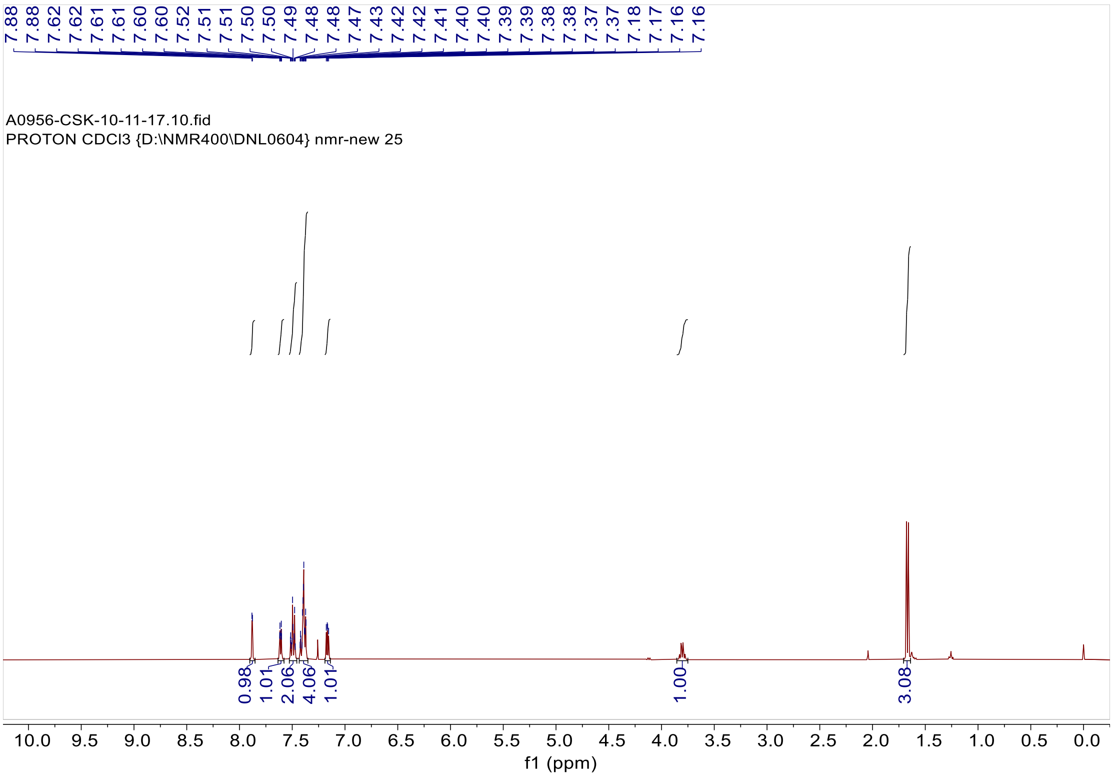

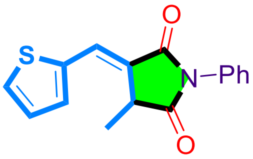

6aj

^{1}H NMR (400 MHz, $CDCl_3$)

^{13}C NMR (100 MHz, $CDCl_3$)

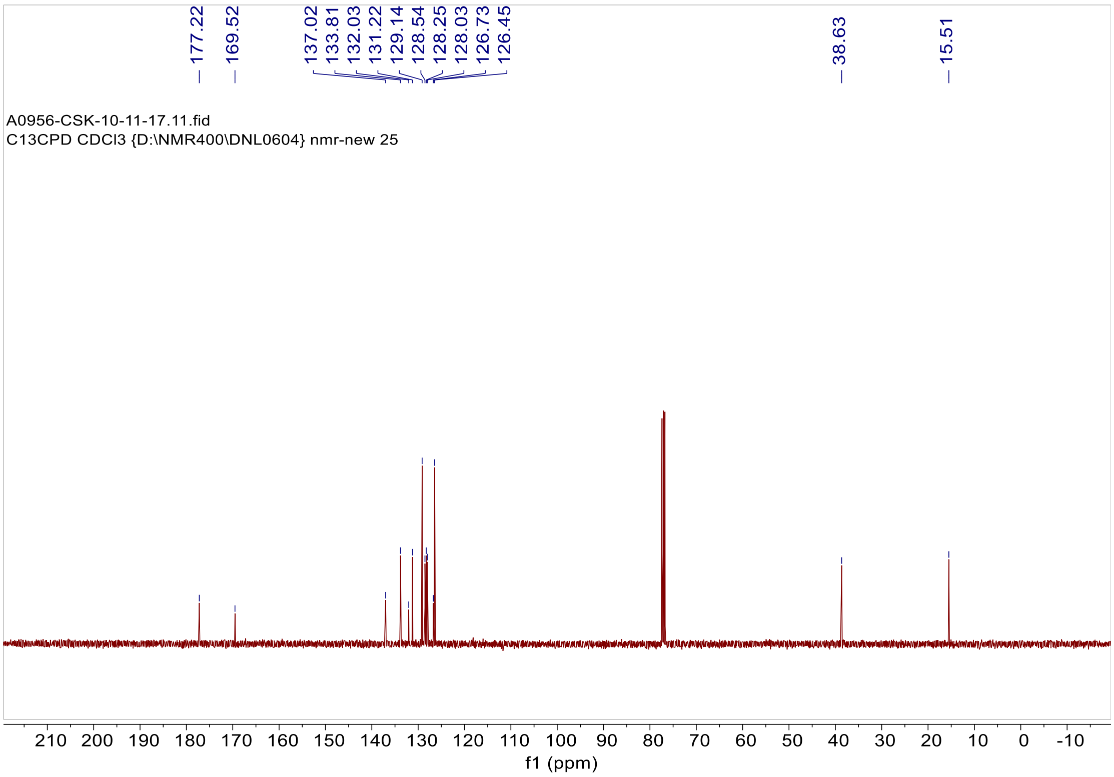

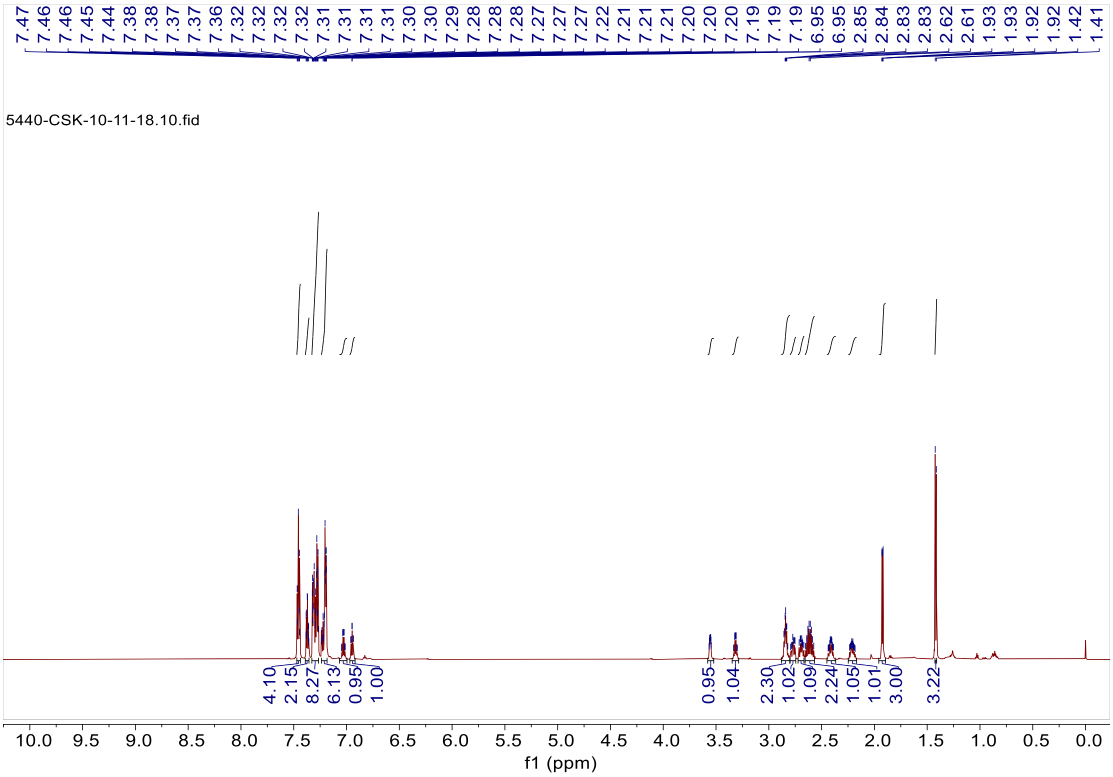

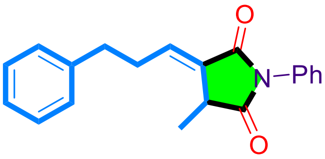

6ak, *E*:*Z* = 1:1

^{1}H NMR (700 MHz, $CDCl_3$)
^{13}C NMR (176 MHz, $CDCl_3$)

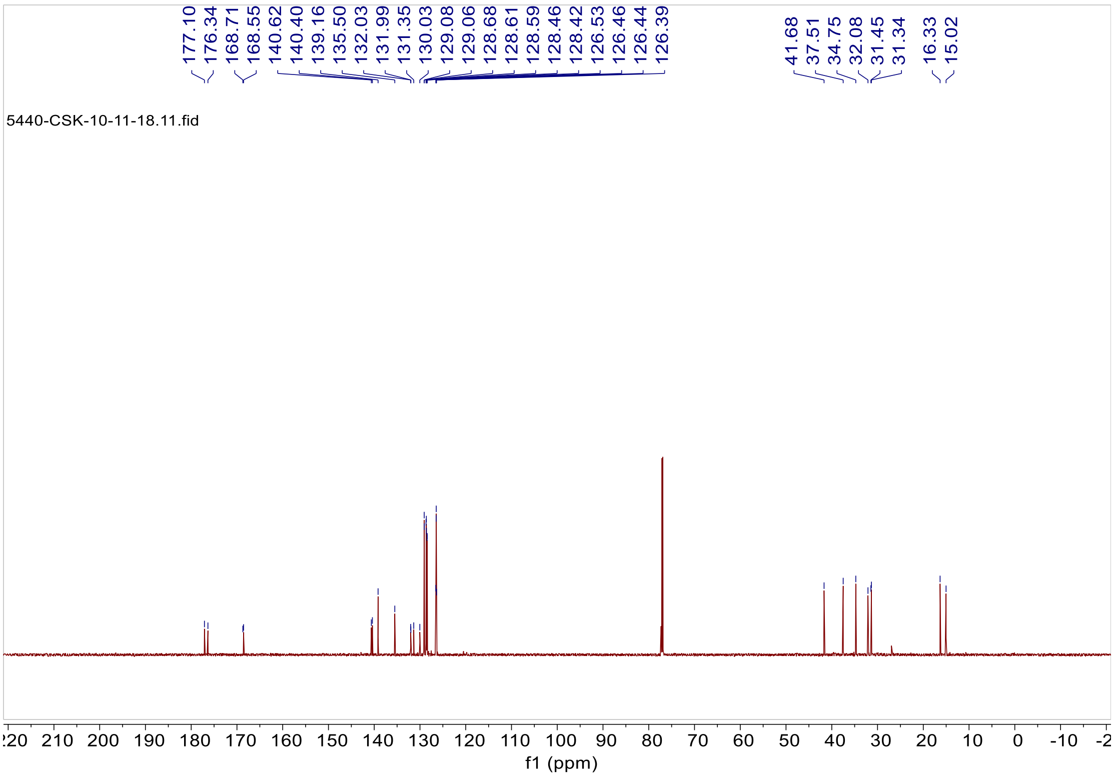

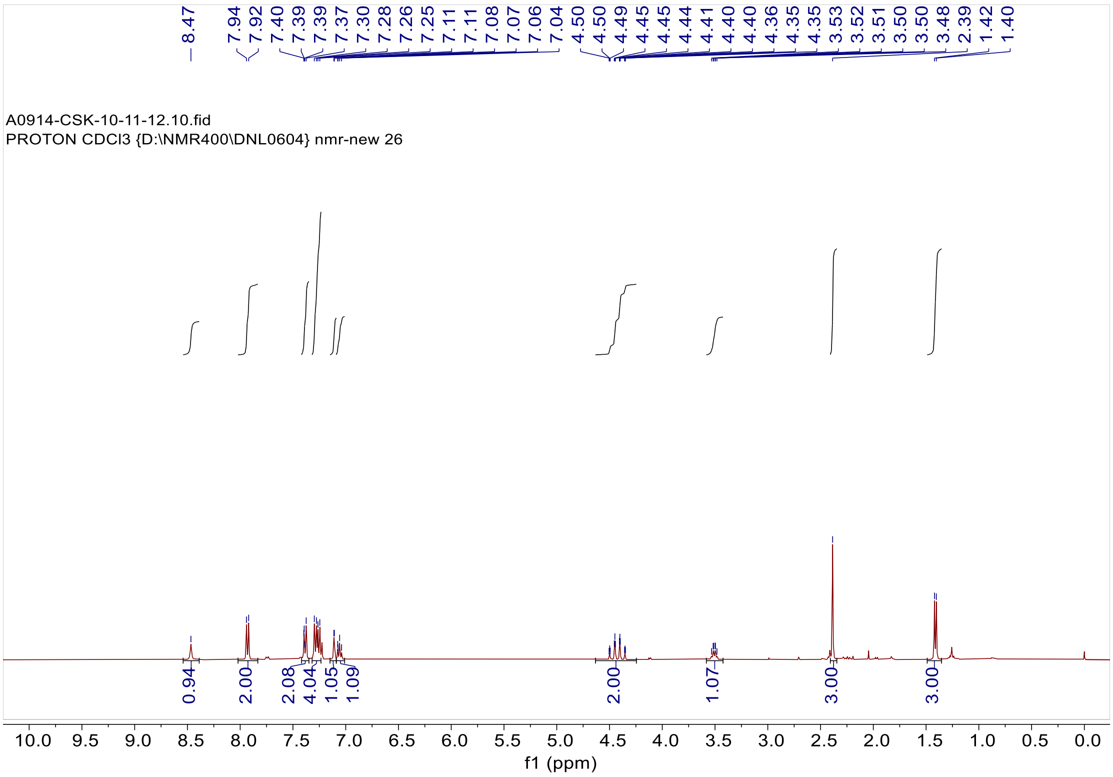

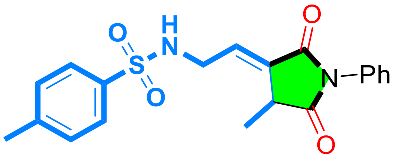

6al

^{1}H NMR (400 MHz, $CDCl_3$)
^{13}C NMR (100 MHz, $CDCl_3$)

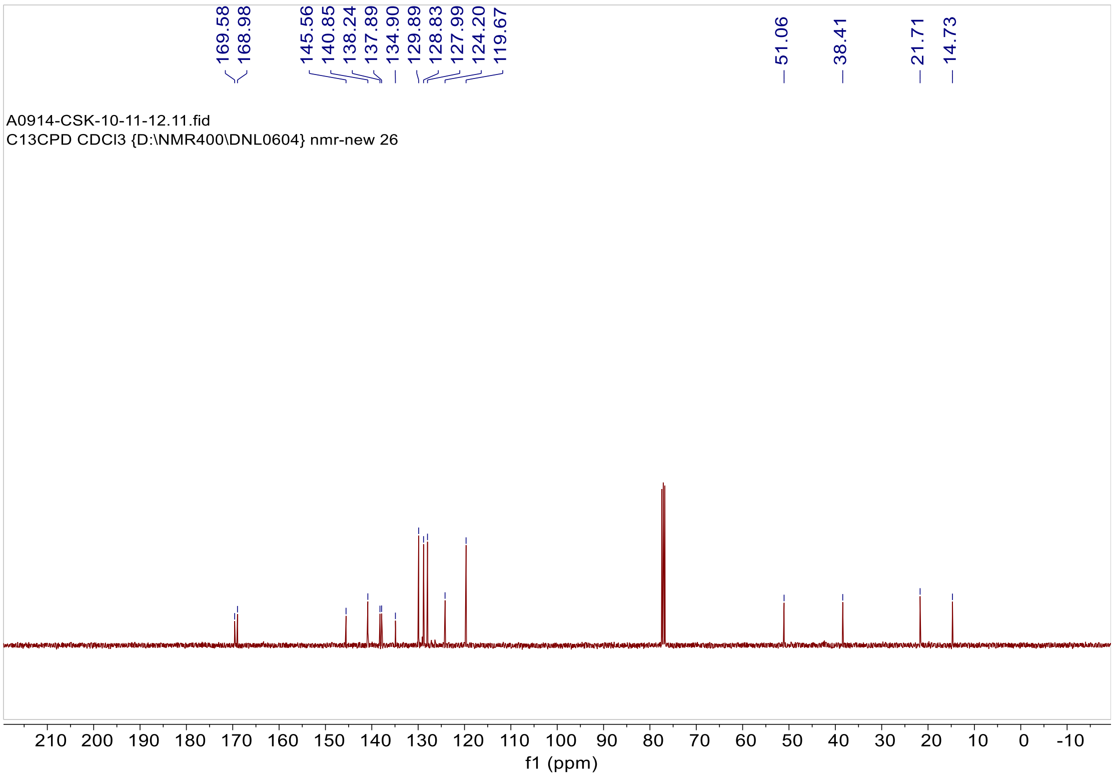

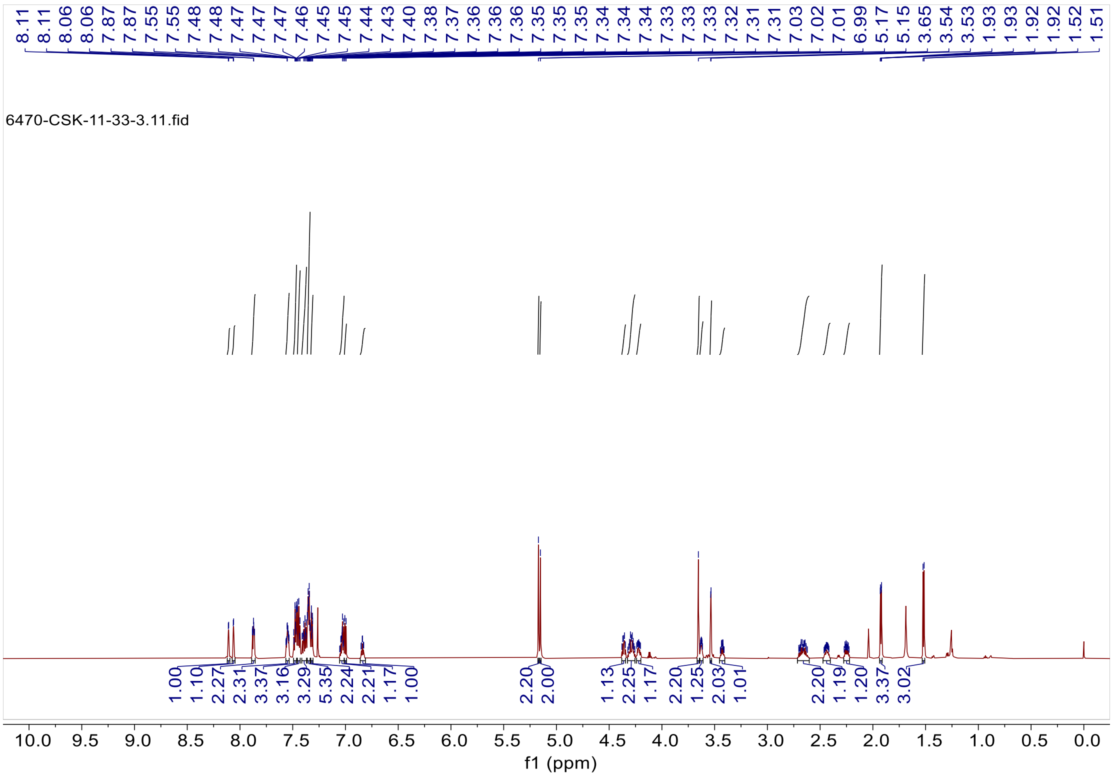

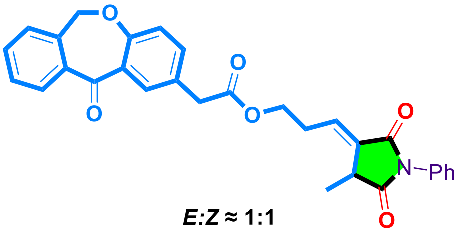

***E:Z* ≈ 1:1**

6am

^{1}H NMR (700 MHz, $CDCl_3$)

^{13}C NMR (176 MHz, $CDCl_3$)

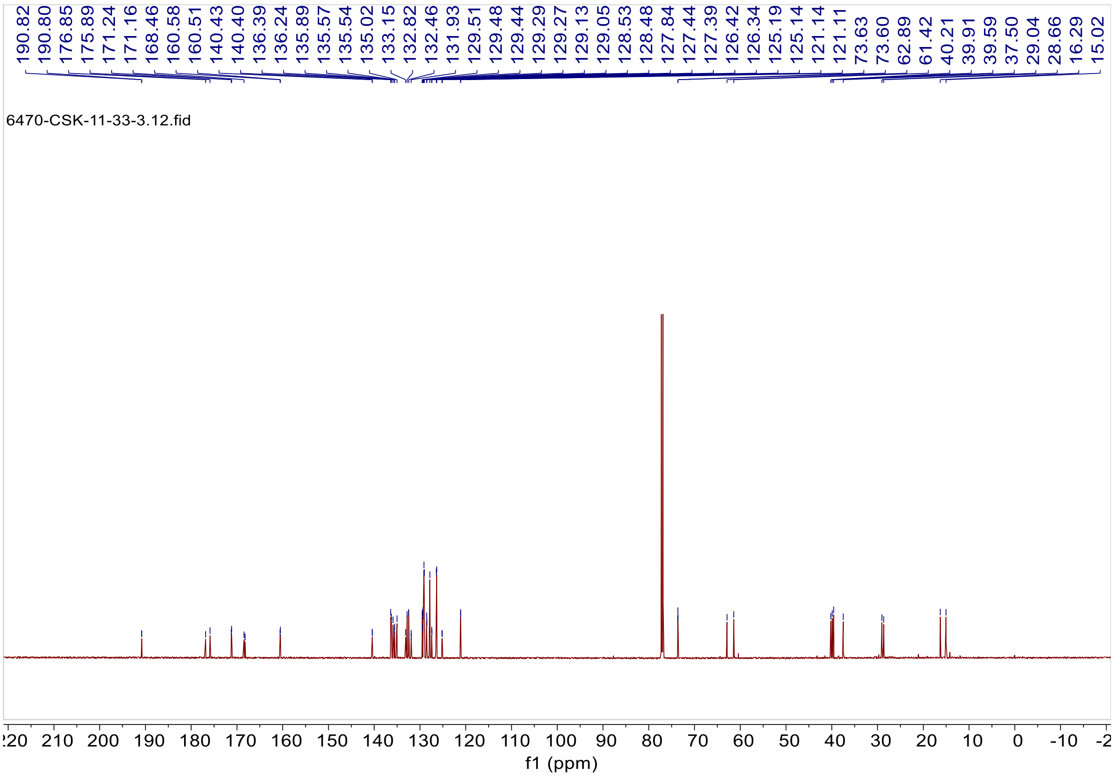

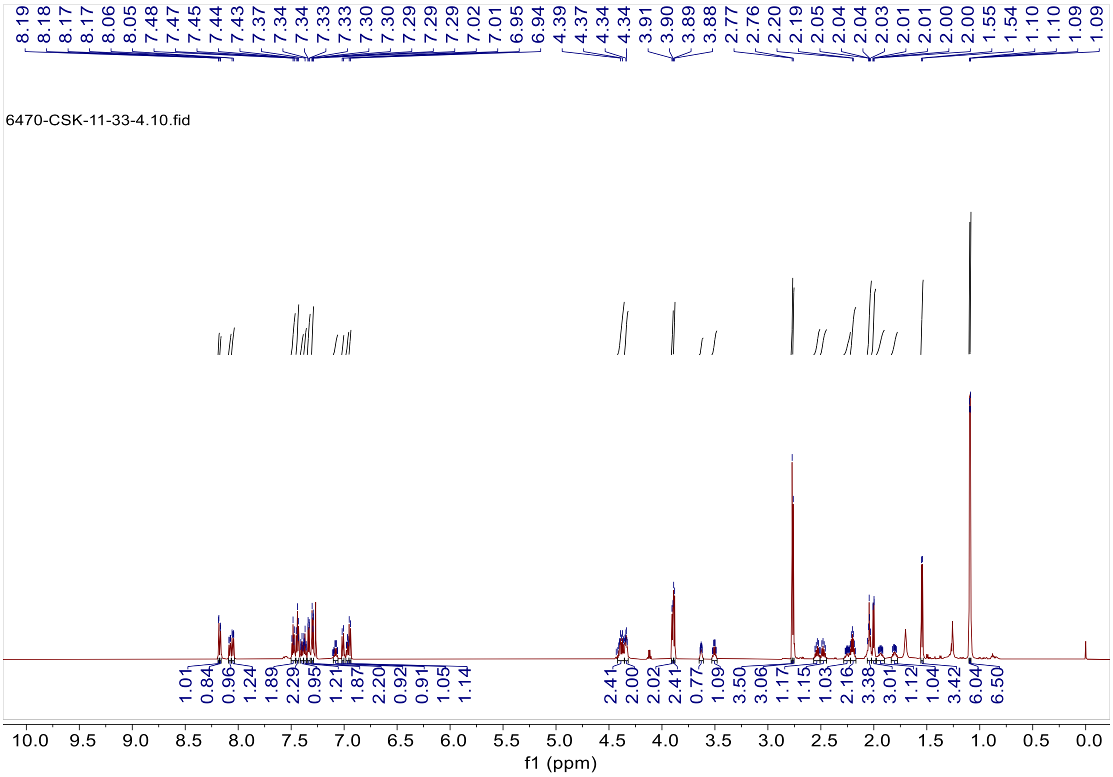

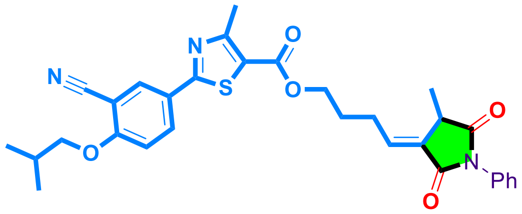

***E:Z* ≈ 1:1**

6an

^{1}H NMR (700 MHz, $CDCl_3$)

^{13}C NMR (176 MHz, $CDCl_3$)

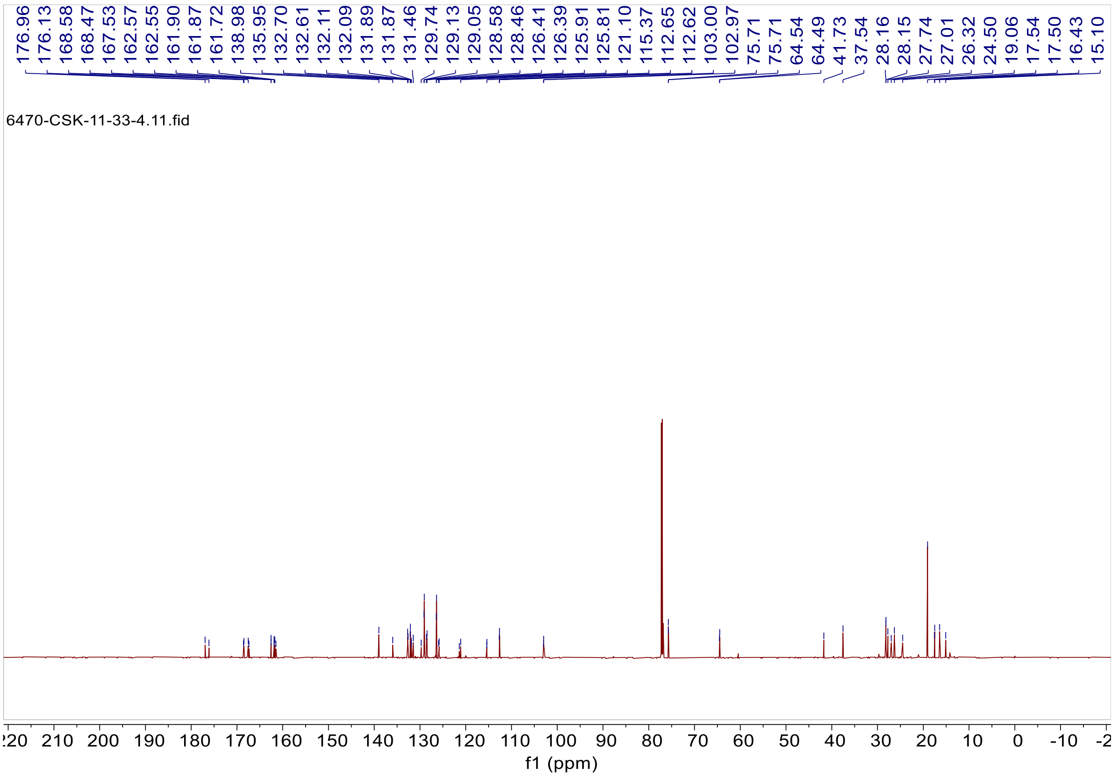

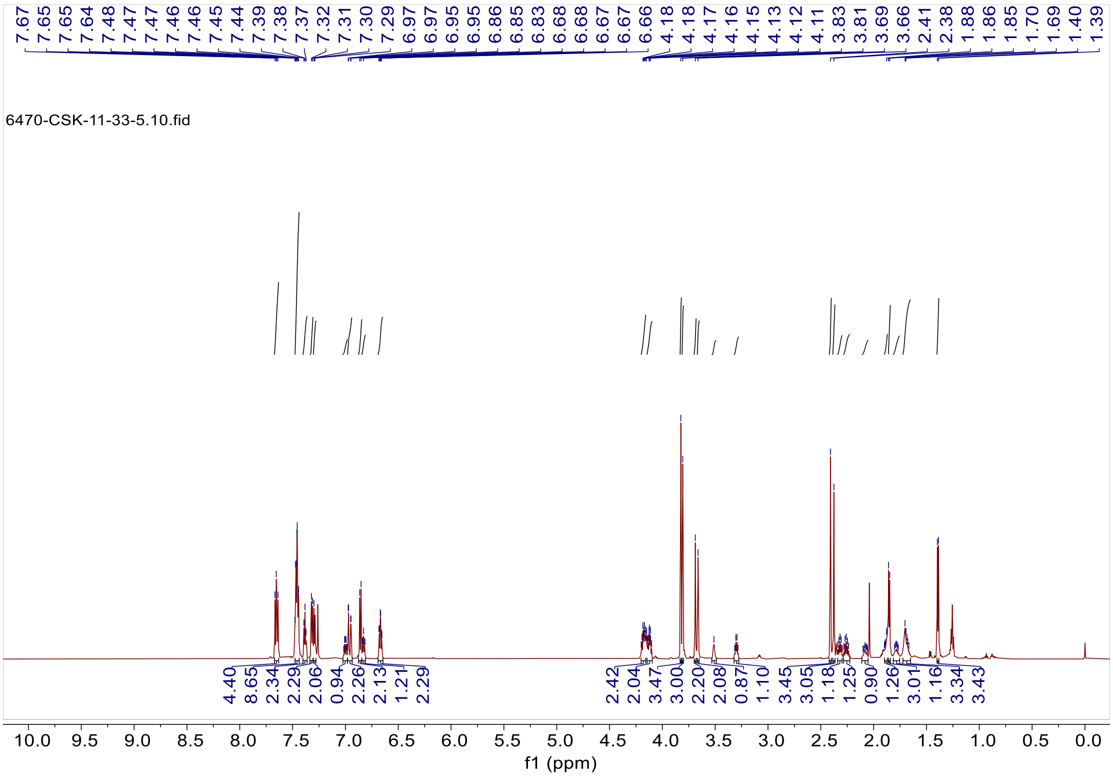

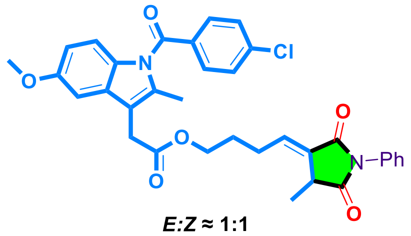

***E*:*Z* ≈ 1:1**

6ao

^{1}H NMR (700 MHz, $CDCl_3$)
^{13}C NMR (176 MHz, $CDCl_3$)

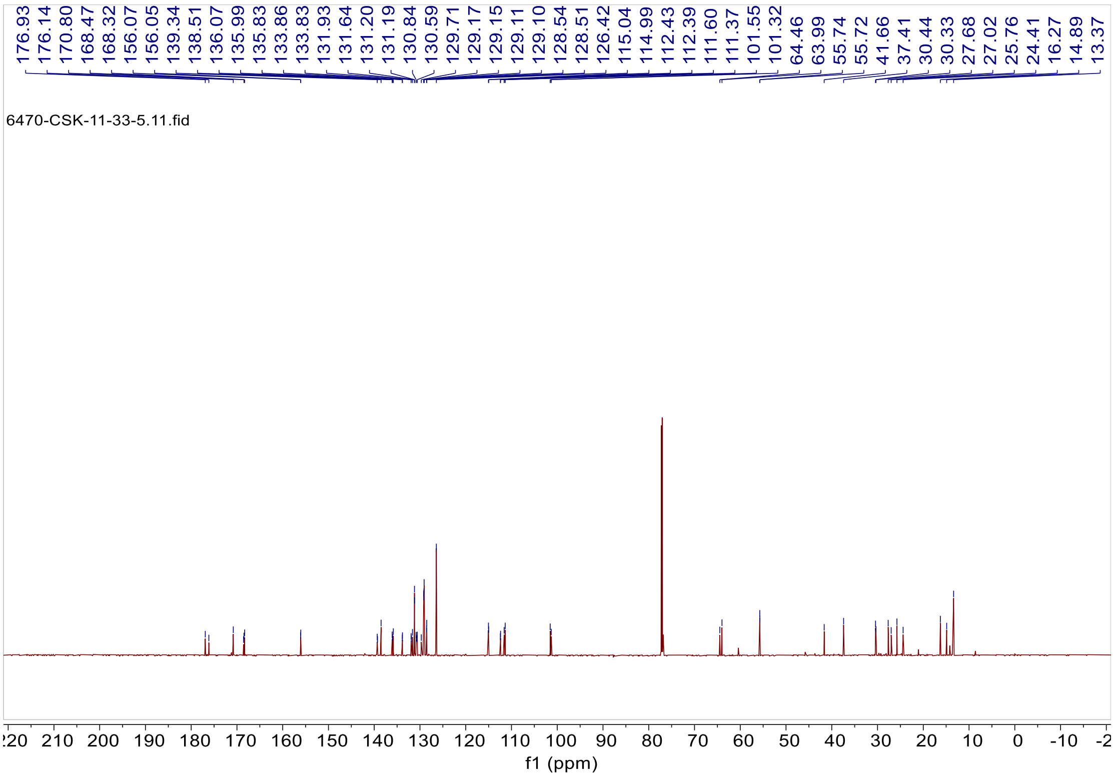

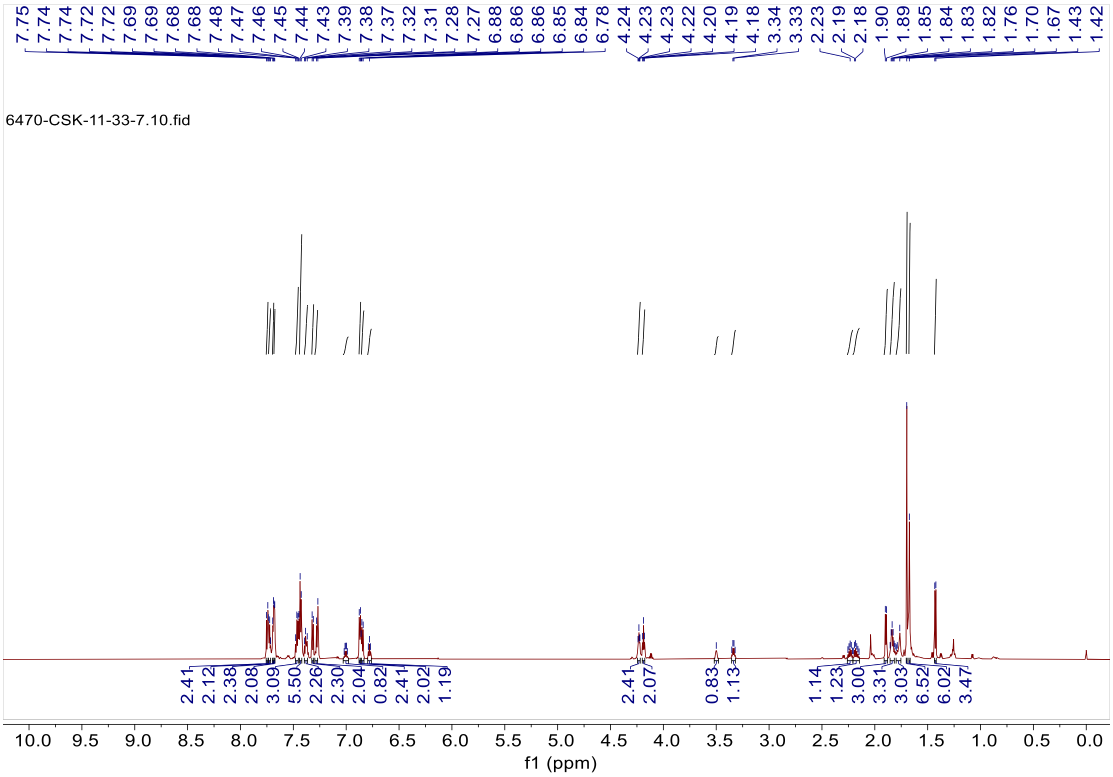

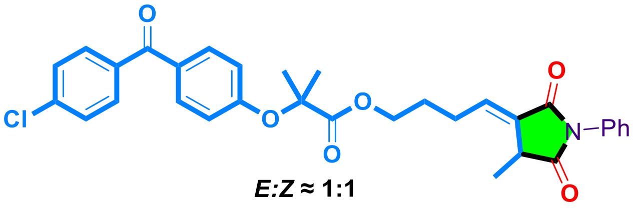

***E:Z* ≈ 1:1**

6ap

^{1}H NMR (700 MHz, $CDCl_3$)
^{13}C NMR (176 MHz, $CDCl_3$)

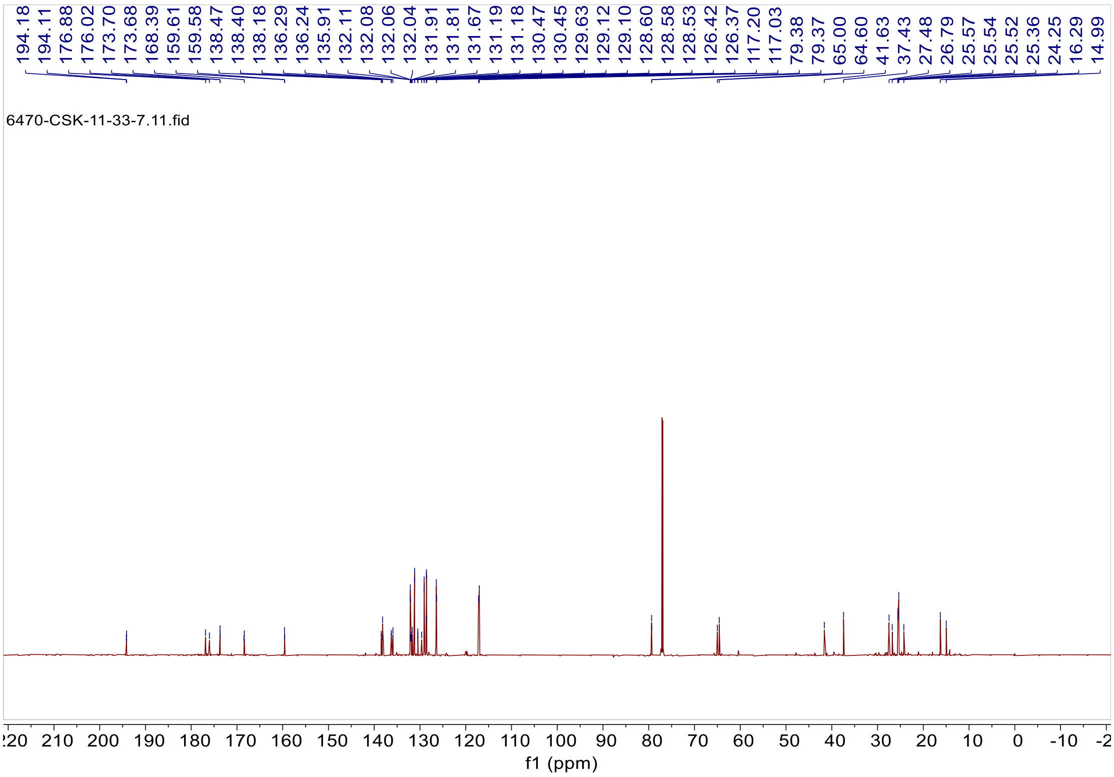

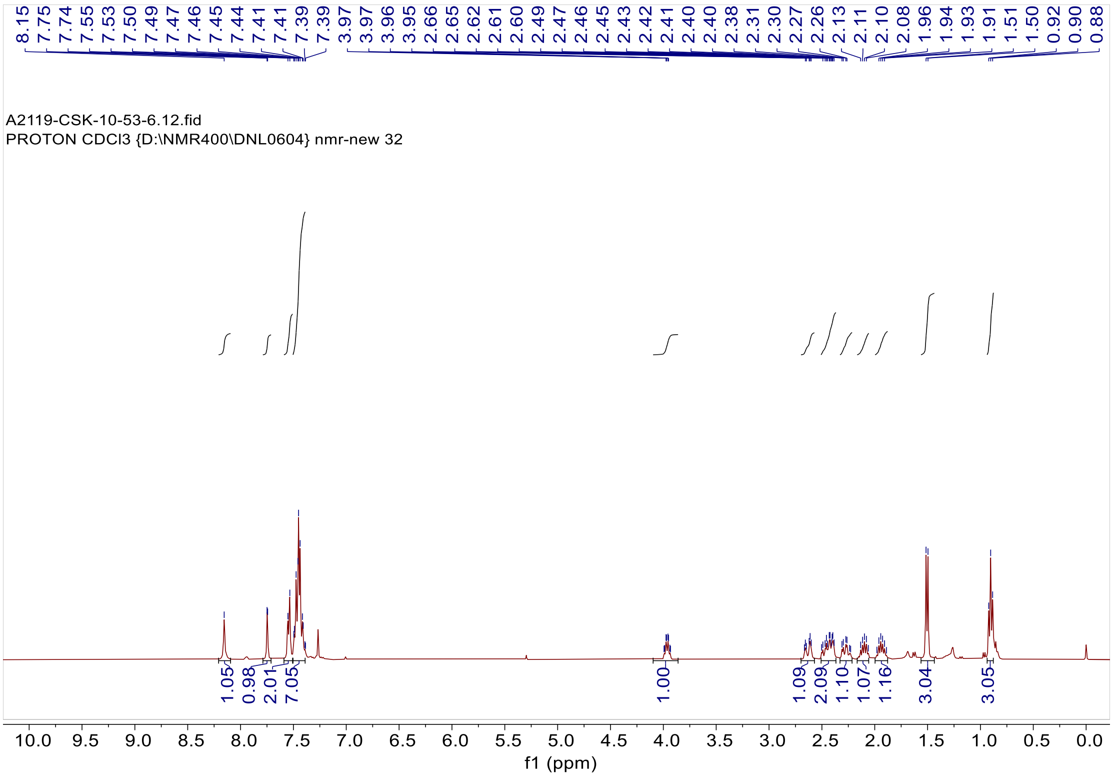

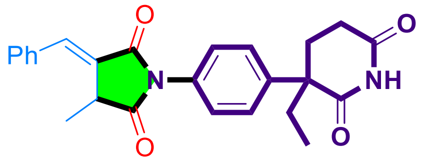

6aq

^{1}H NMR (400 MHz, $CDCl_3$)
^{13}C NMR (100 MHz, $CDCl_3$)

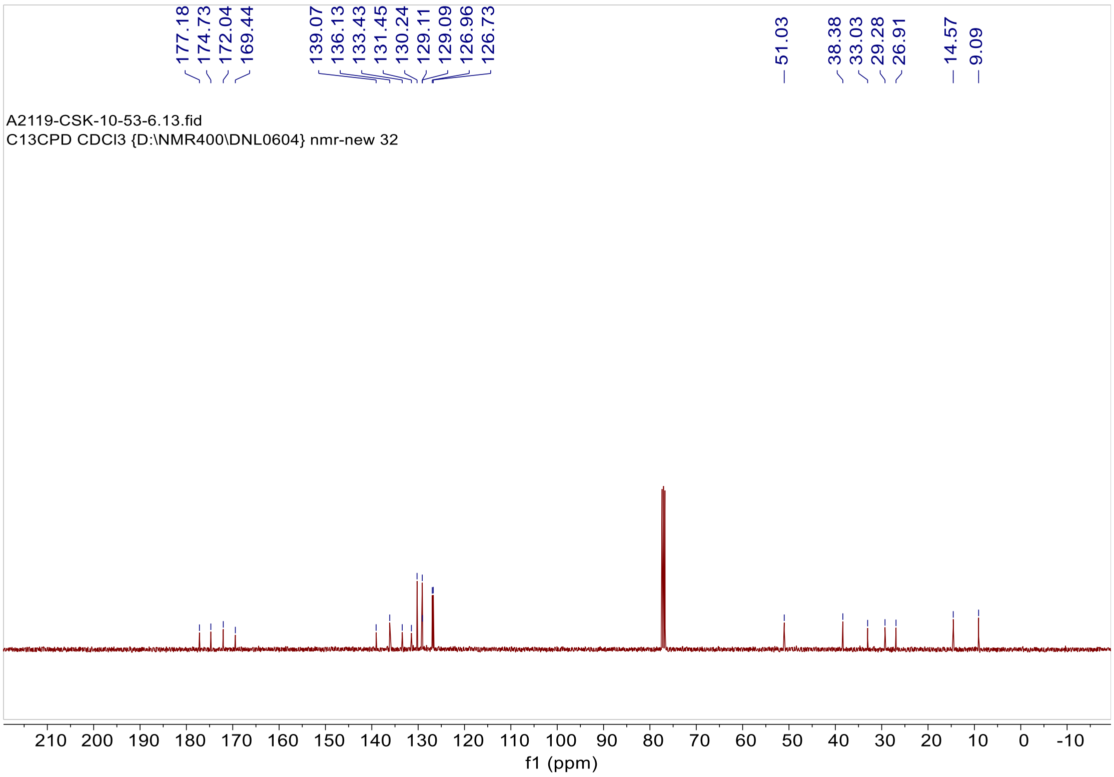

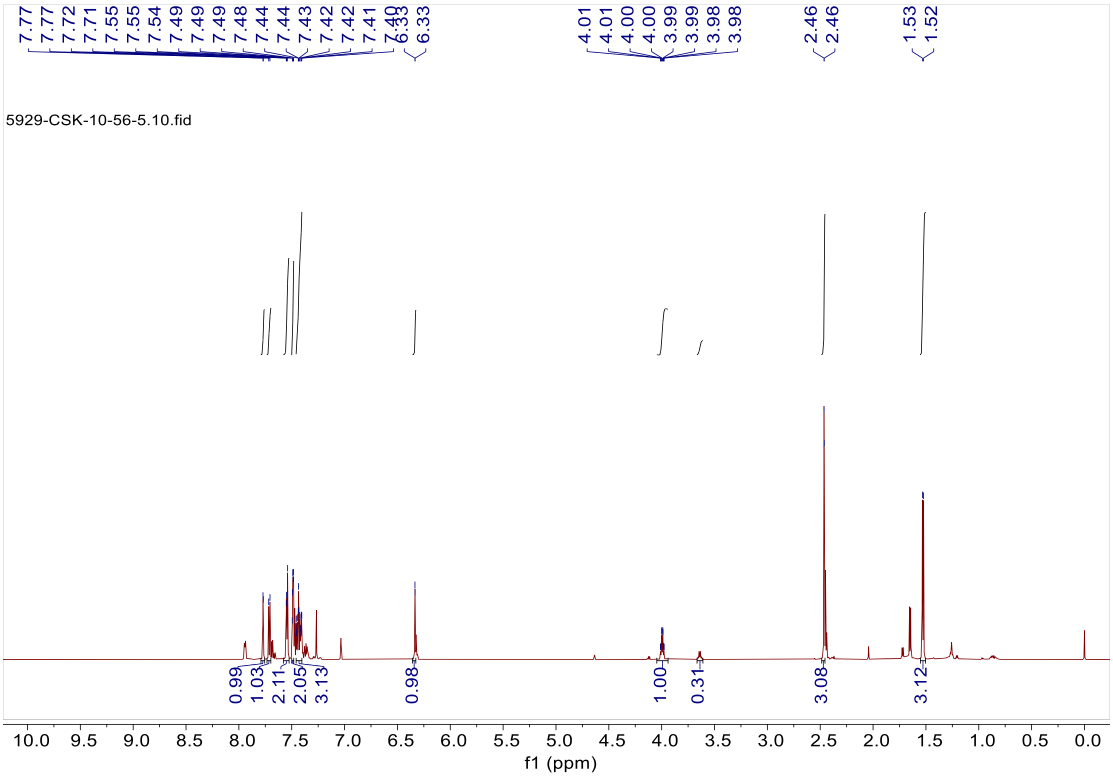

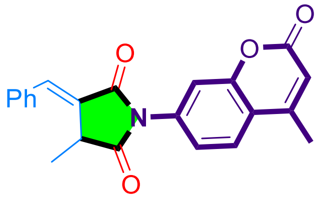

6ar

^{1}H NMR (700 MHz, $CDCl_3$)

^{13}C NMR (176 MHz, $CDCl_3$)

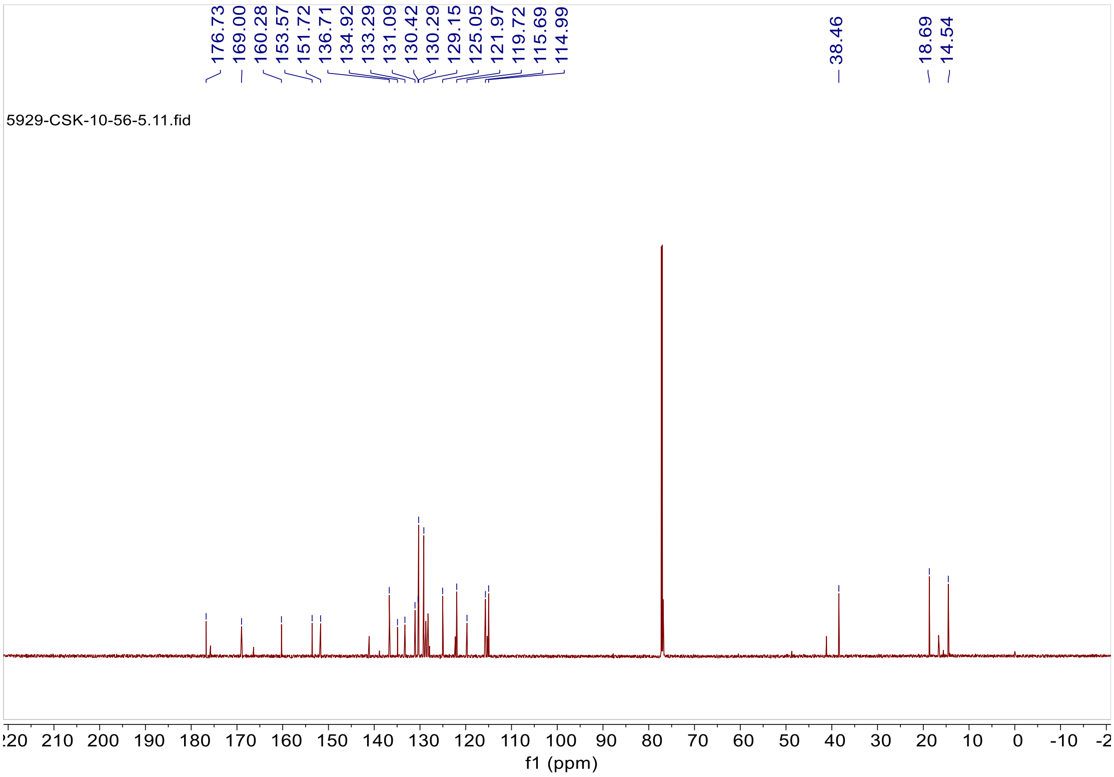

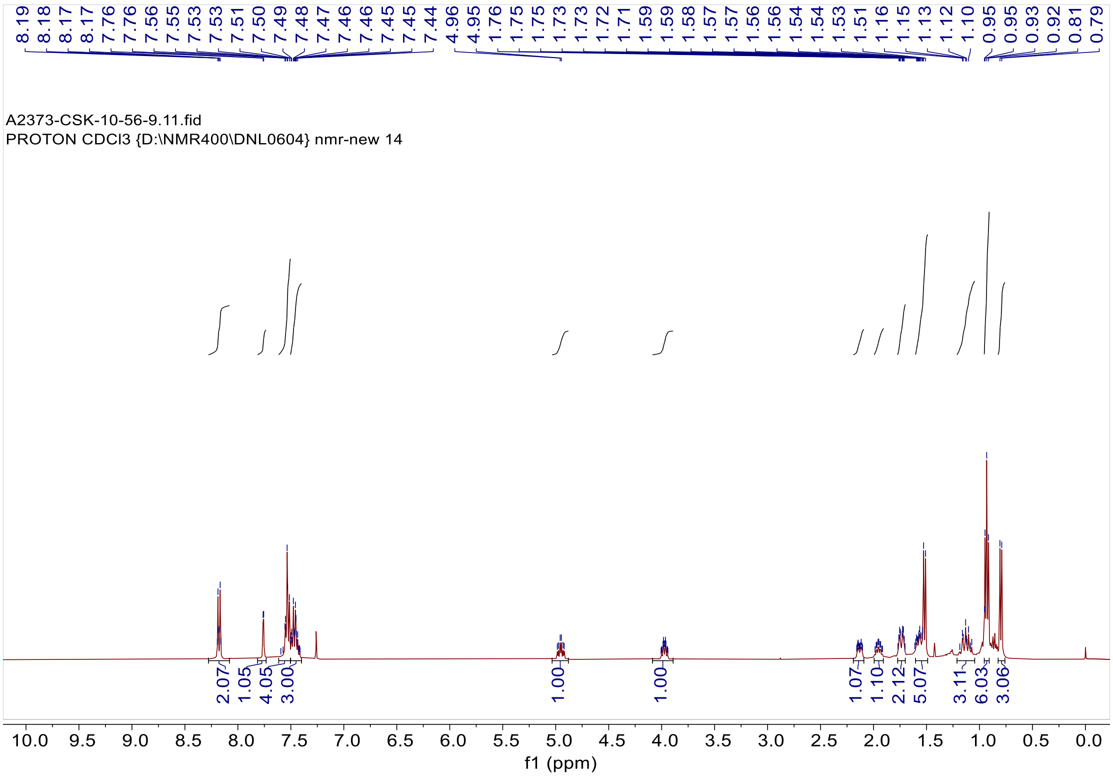

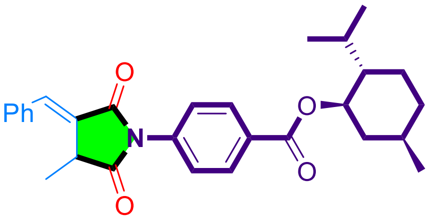

6as

^{1}H NMR (400 MHz, $CDCl_3$)
^{13}C NMR (100 MHz, $CDCl_3$)

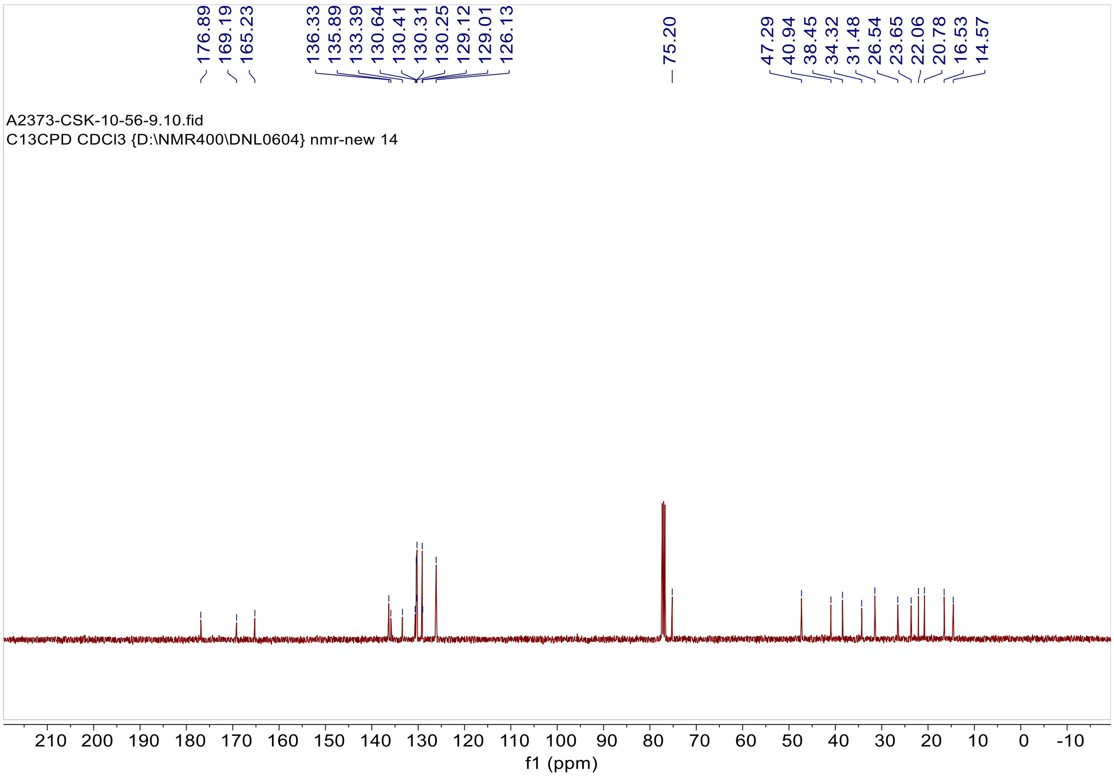

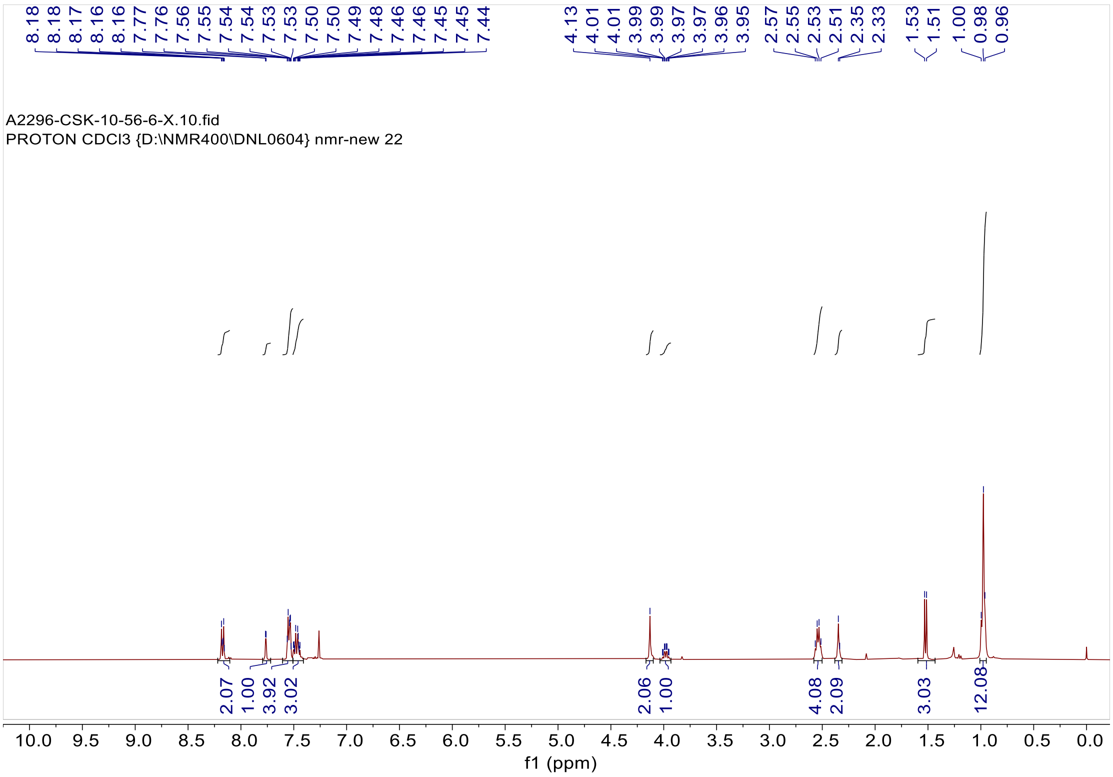

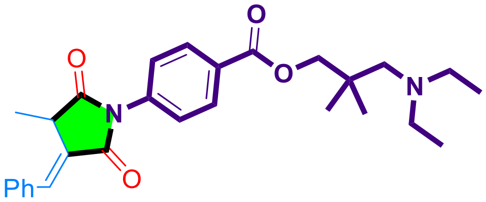

6at

^{1}H NMR (400 MHz, $CDCl_3$)
^{13}C NMR (100 MHz, $CDCl_3$)

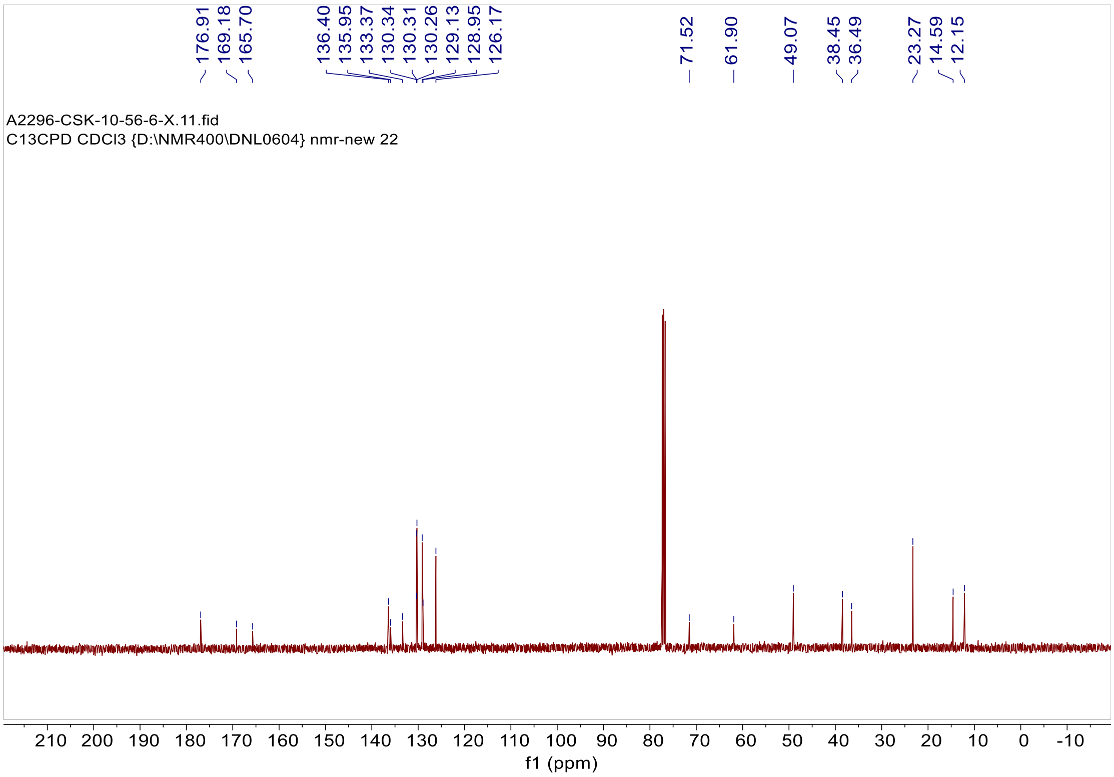

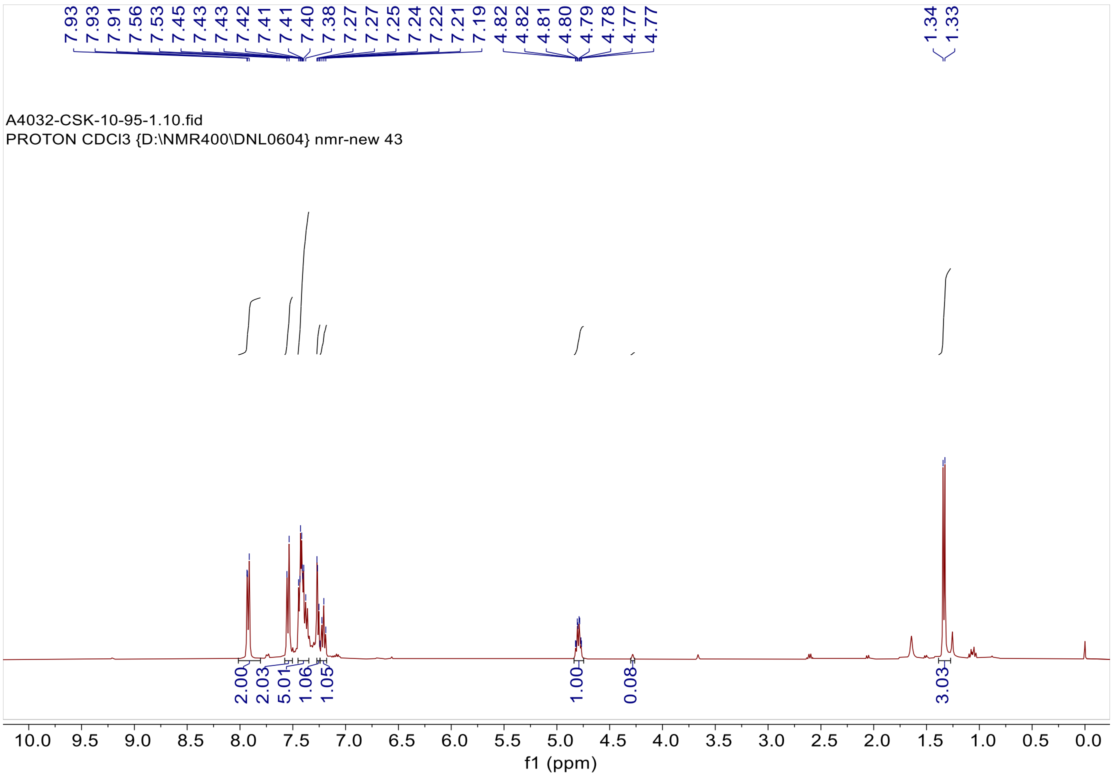

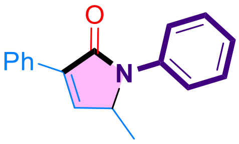

7a

^{1}H NMR (400 MHz, $CDCl_3$)
^{13}C NMR (100 MHz, $CDCl_3$)

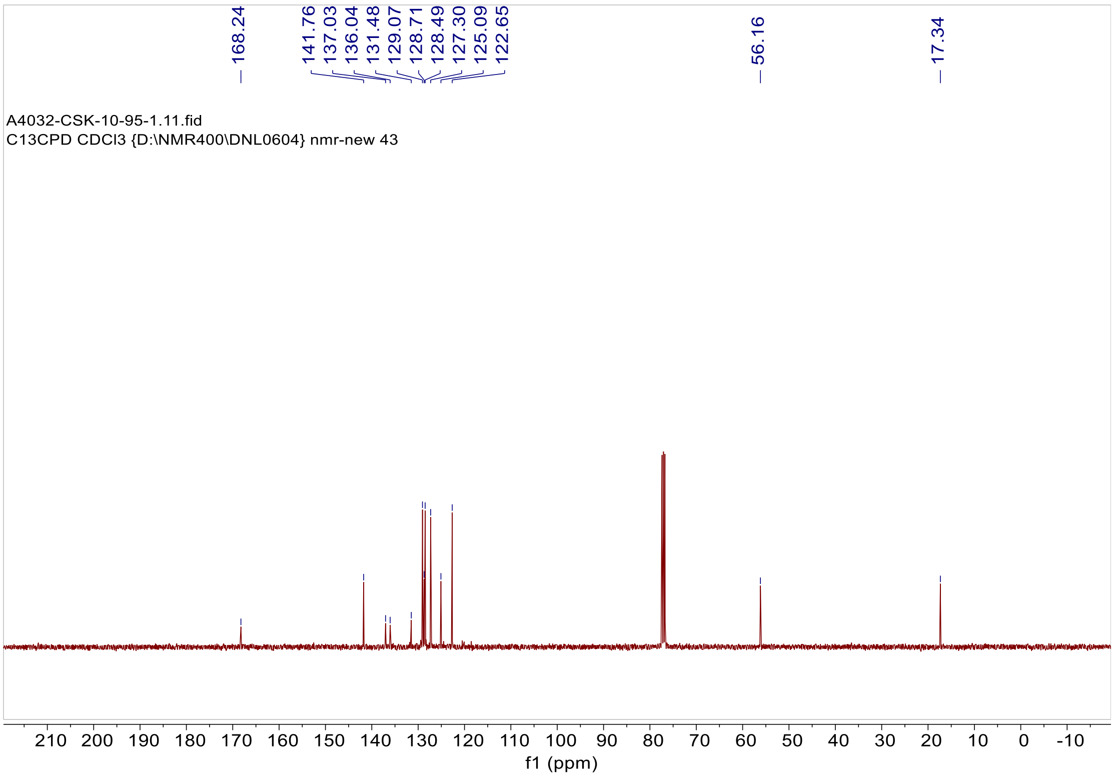

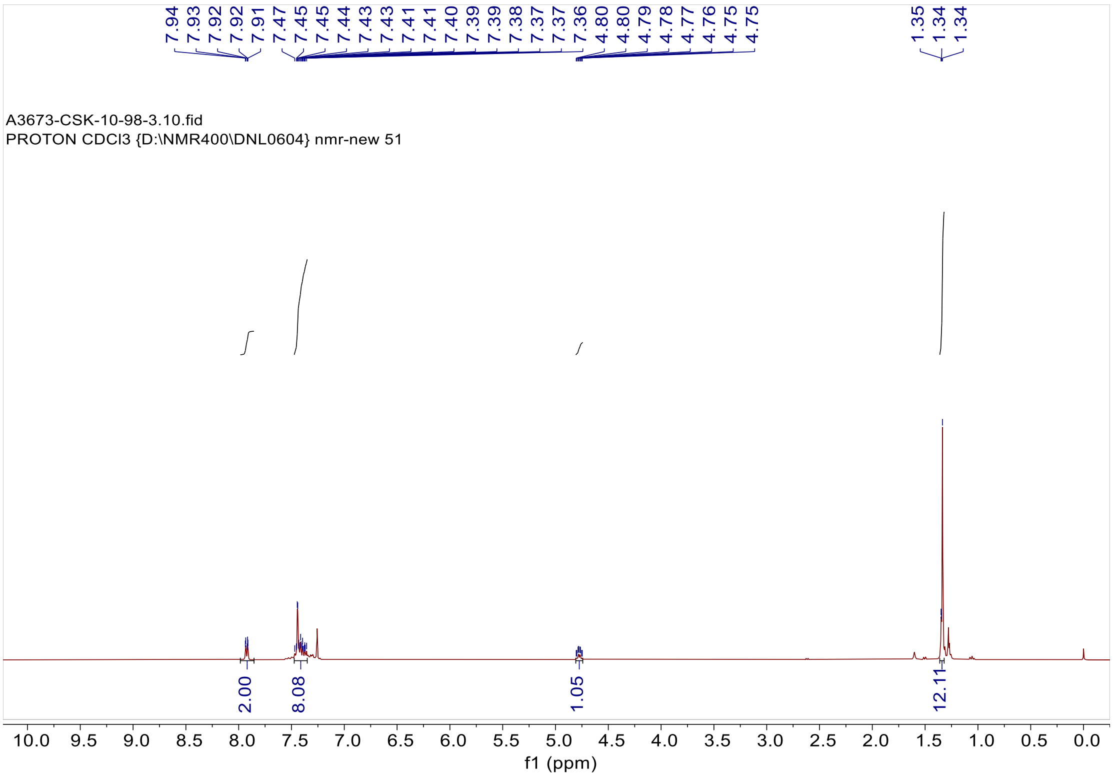

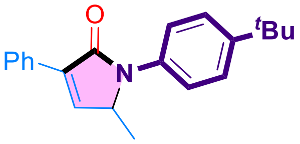

7b

^{1}H NMR (400 MHz, $CDCl_3$)
^{13}C NMR (100 MHz, $CDCl_3$)

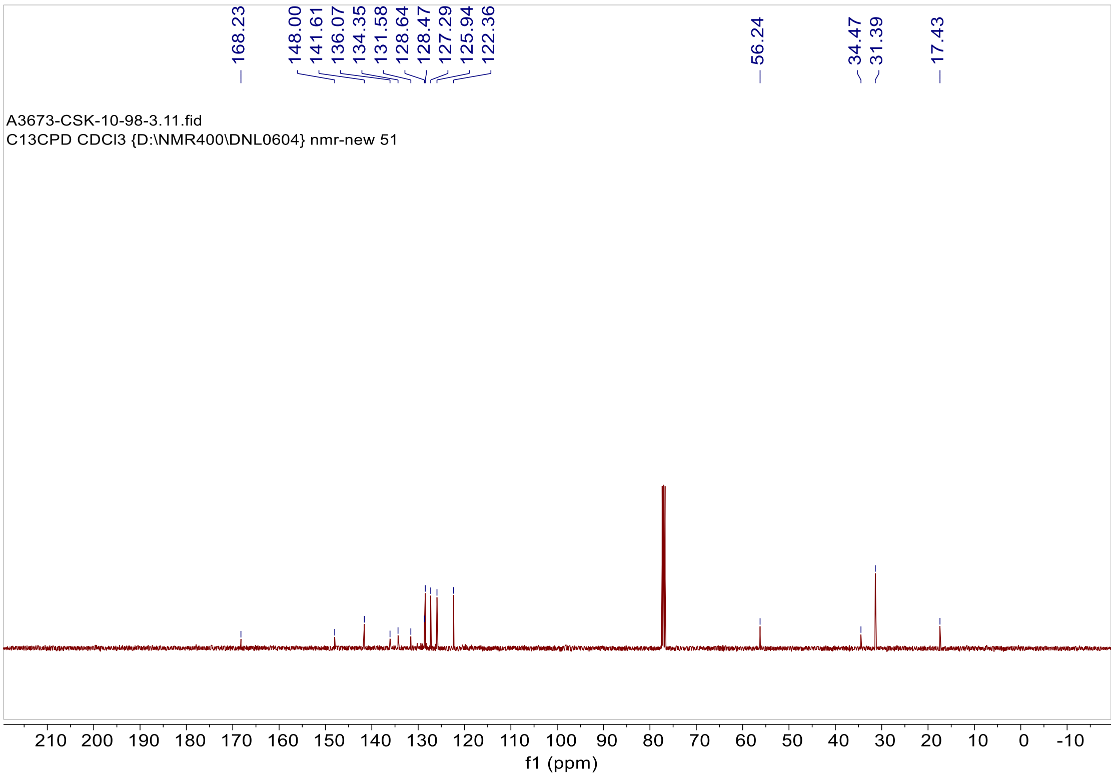

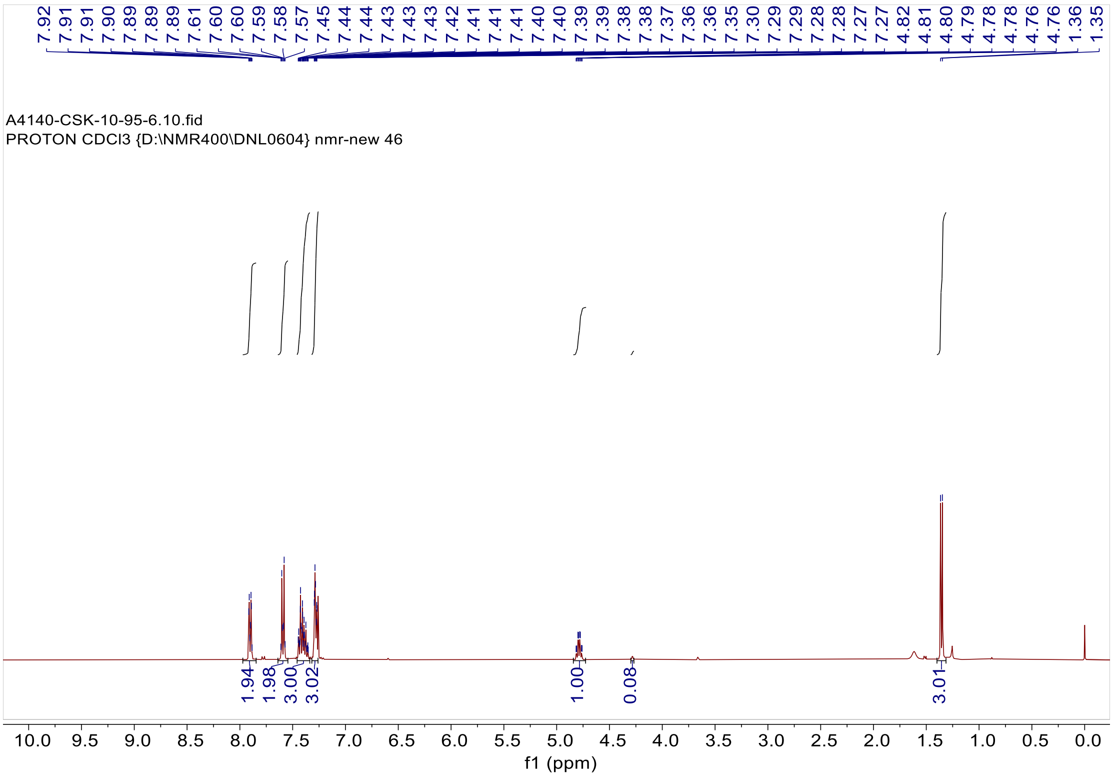

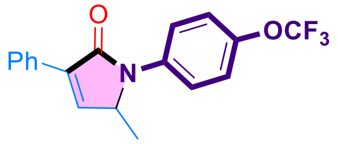

7c

^{1}H NMR (400 MHz, $CDCl_3$)

^{13}C NMR (100 MHz, $CDCl_3$)

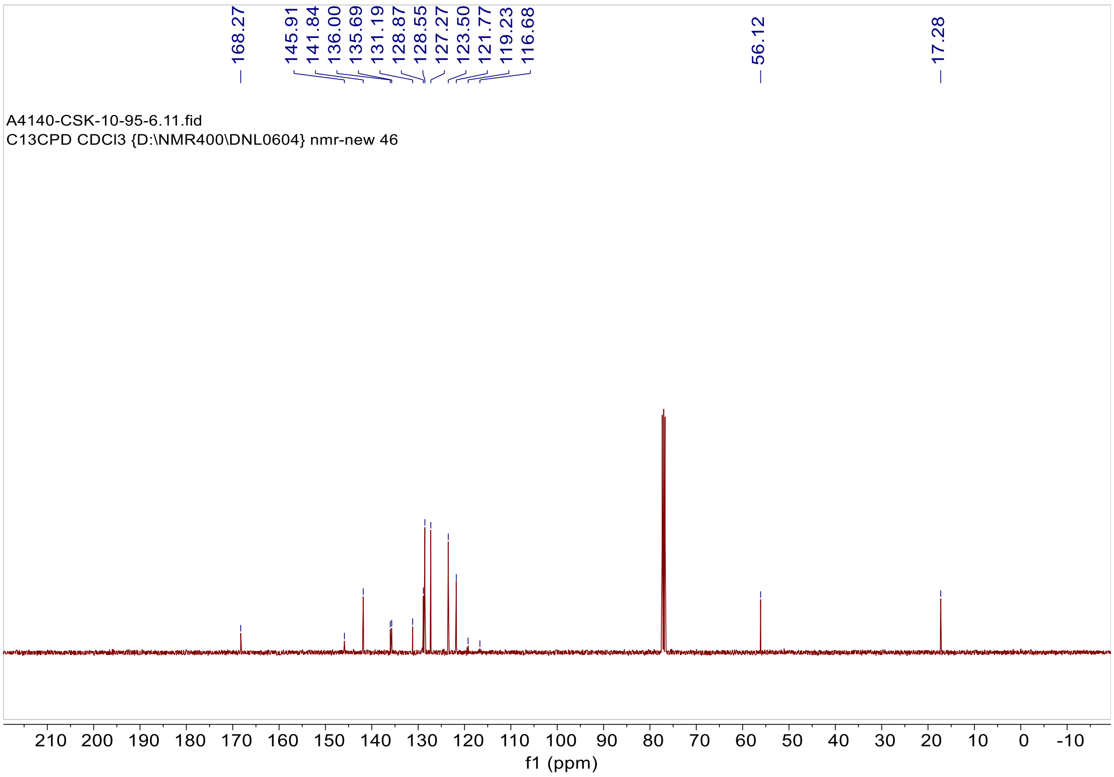

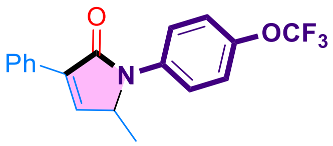

7c ^{19}F NMR (376 MHz, $CDCl_3$)

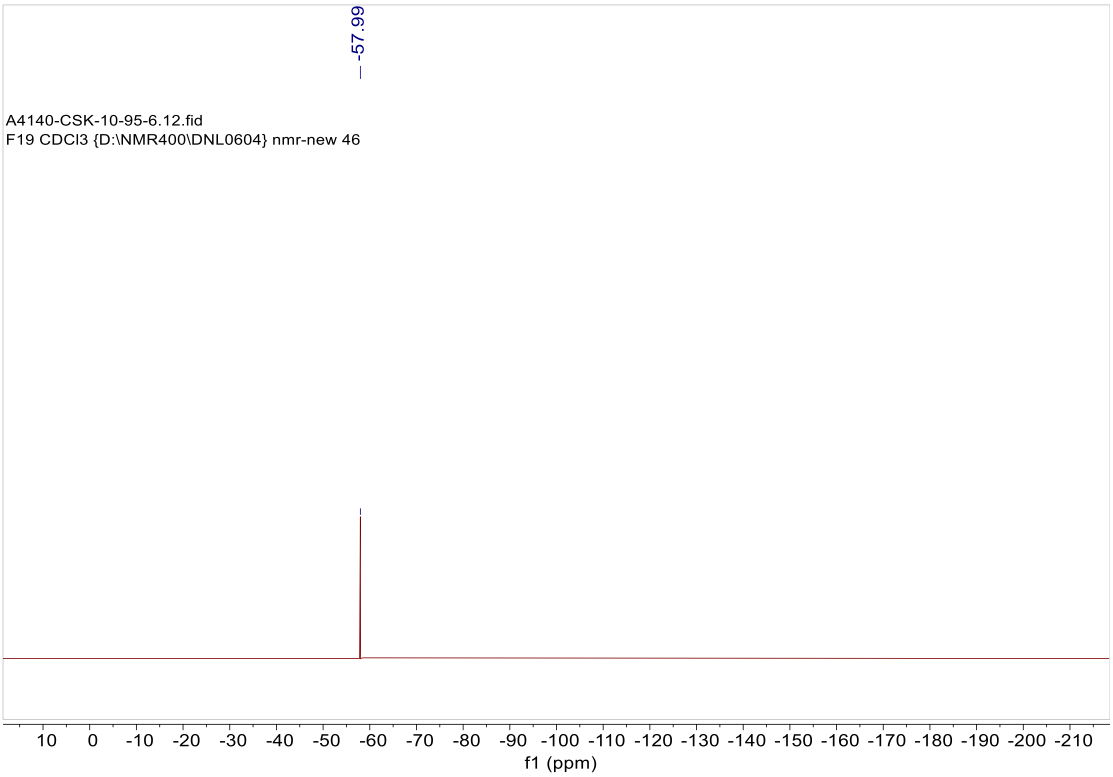

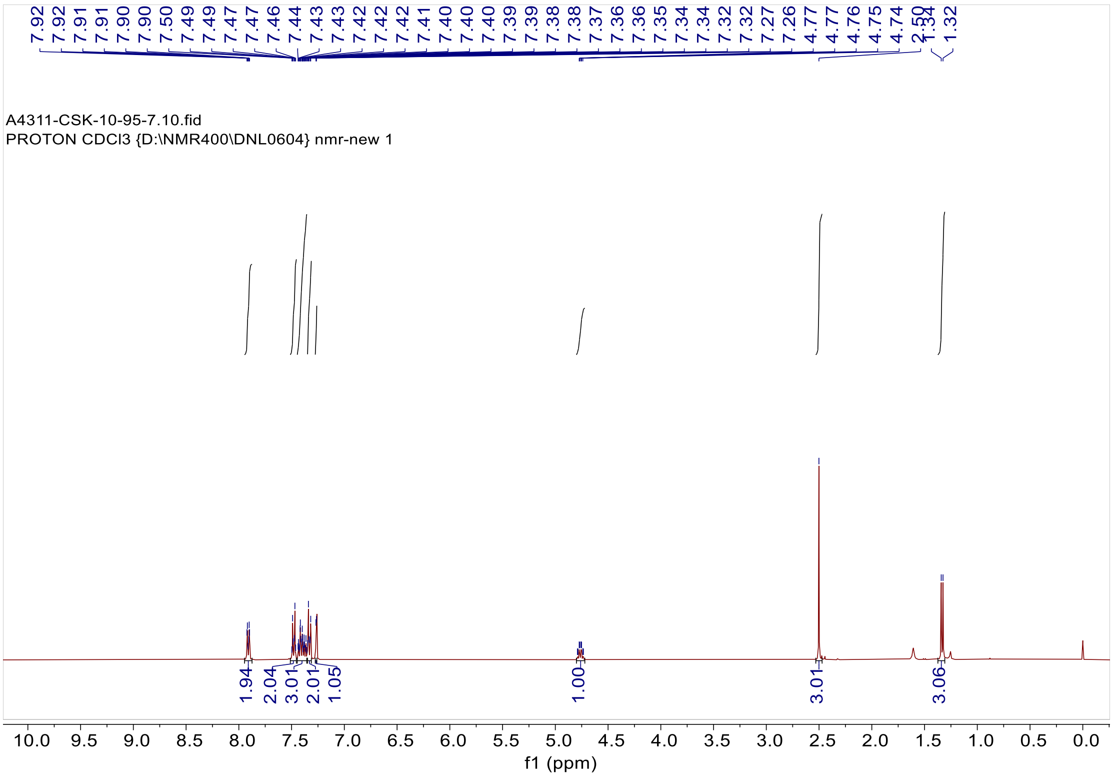

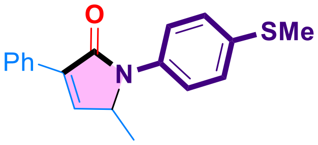

7d

^{1}H NMR (400 MHz, $CDCl_3$)
^{13}C NMR (100 MHz, $CDCl_3$)

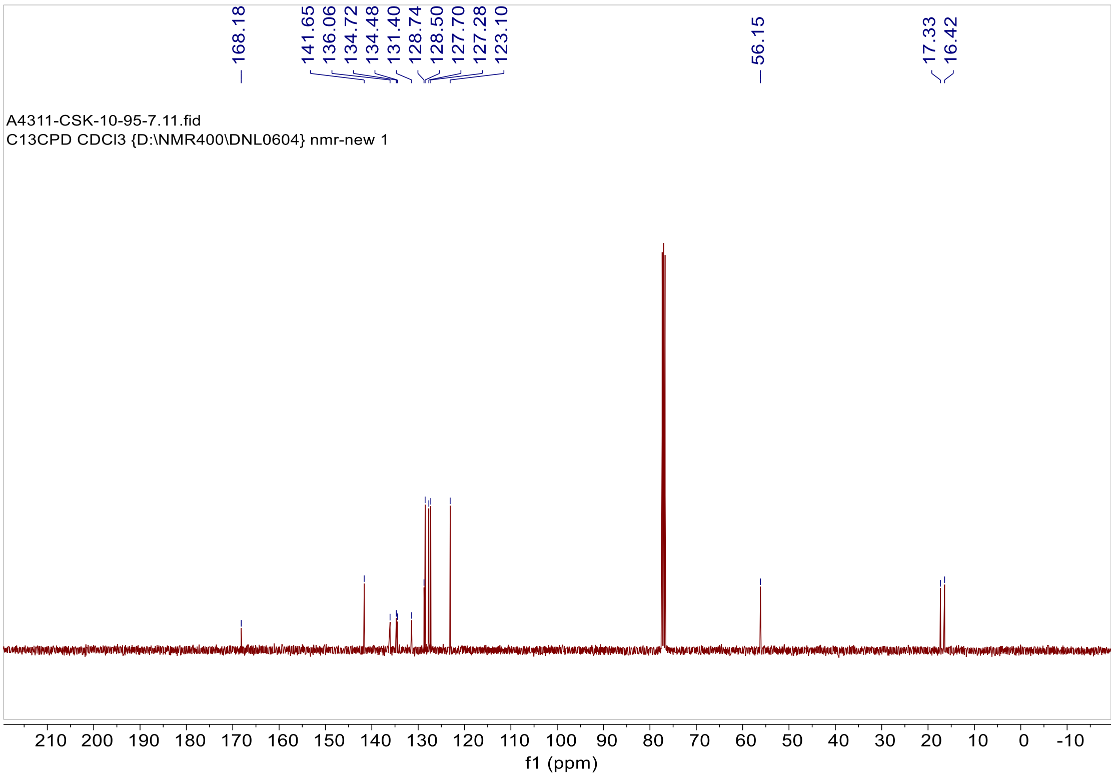

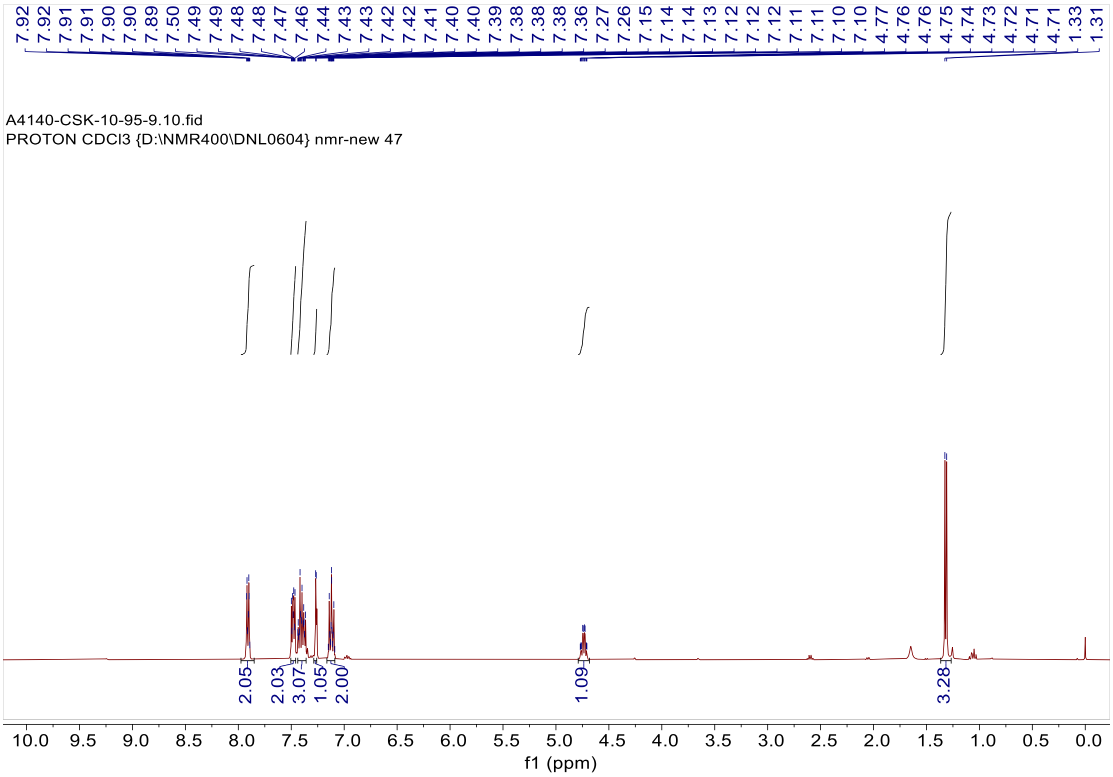

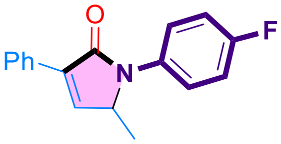

7e

^{1}H NMR (400 MHz, $CDCl_3$)
^{13}C NMR (100 MHz, $CDCl_3$)

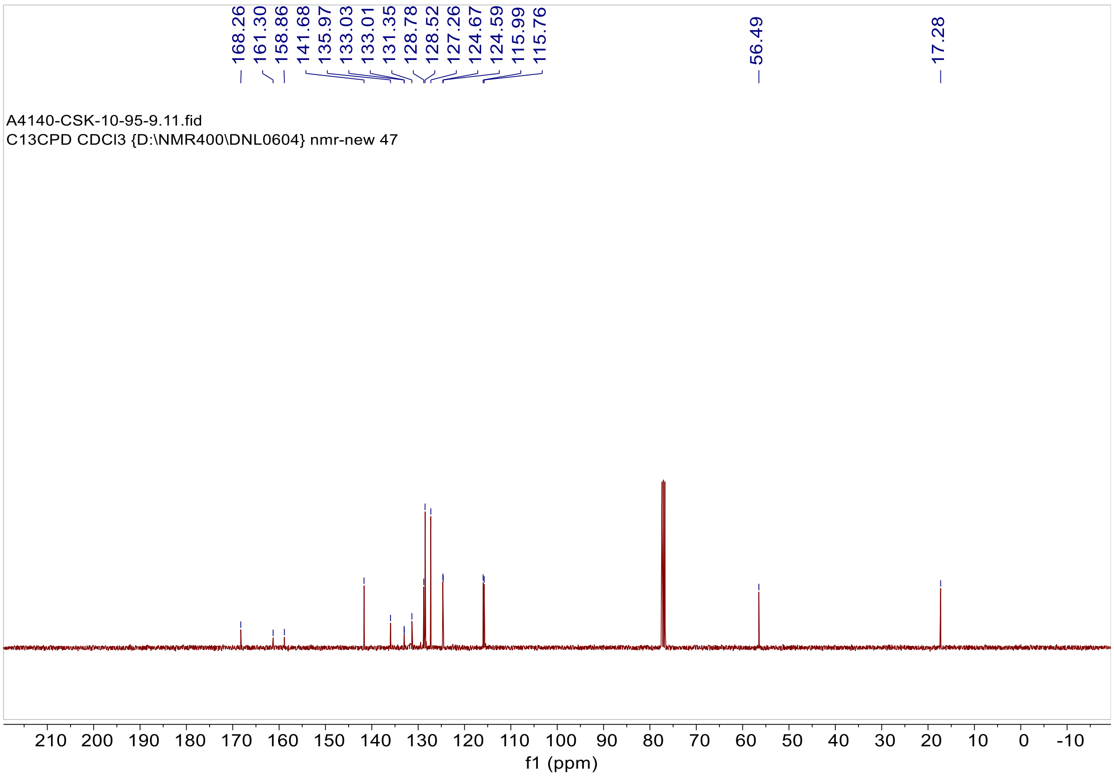

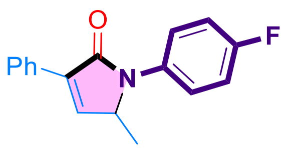

7e ^{19}F NMR (376 MHz, $CDCl_3$)

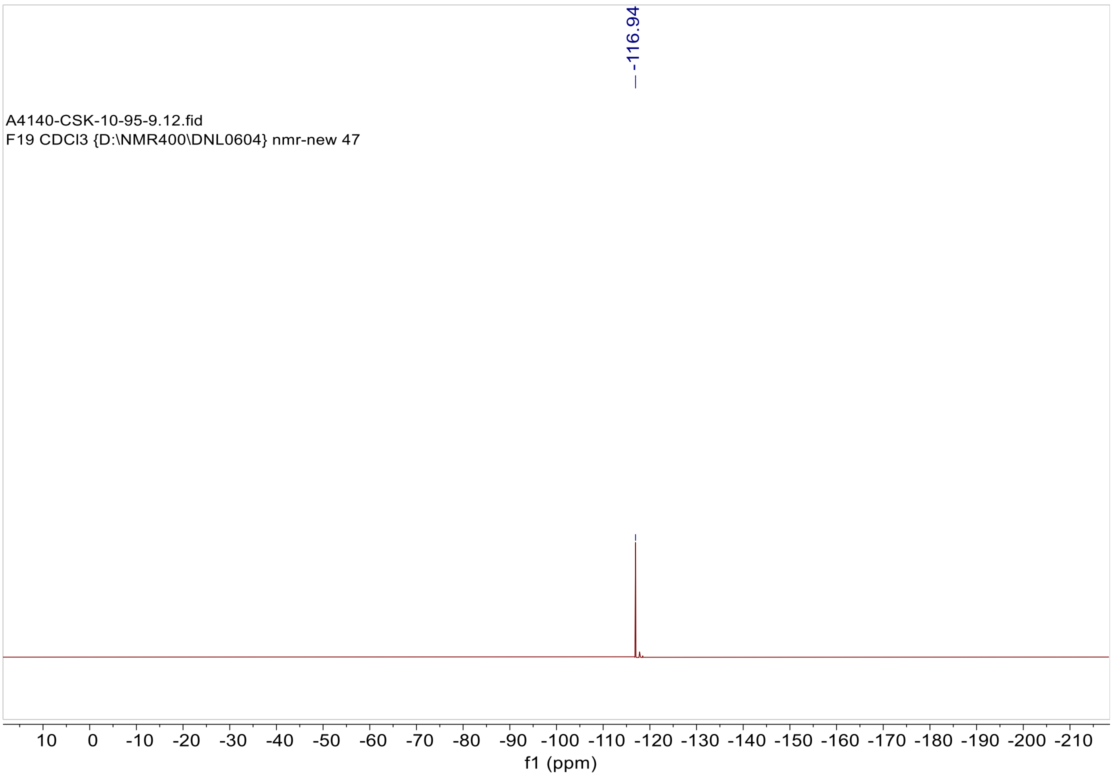

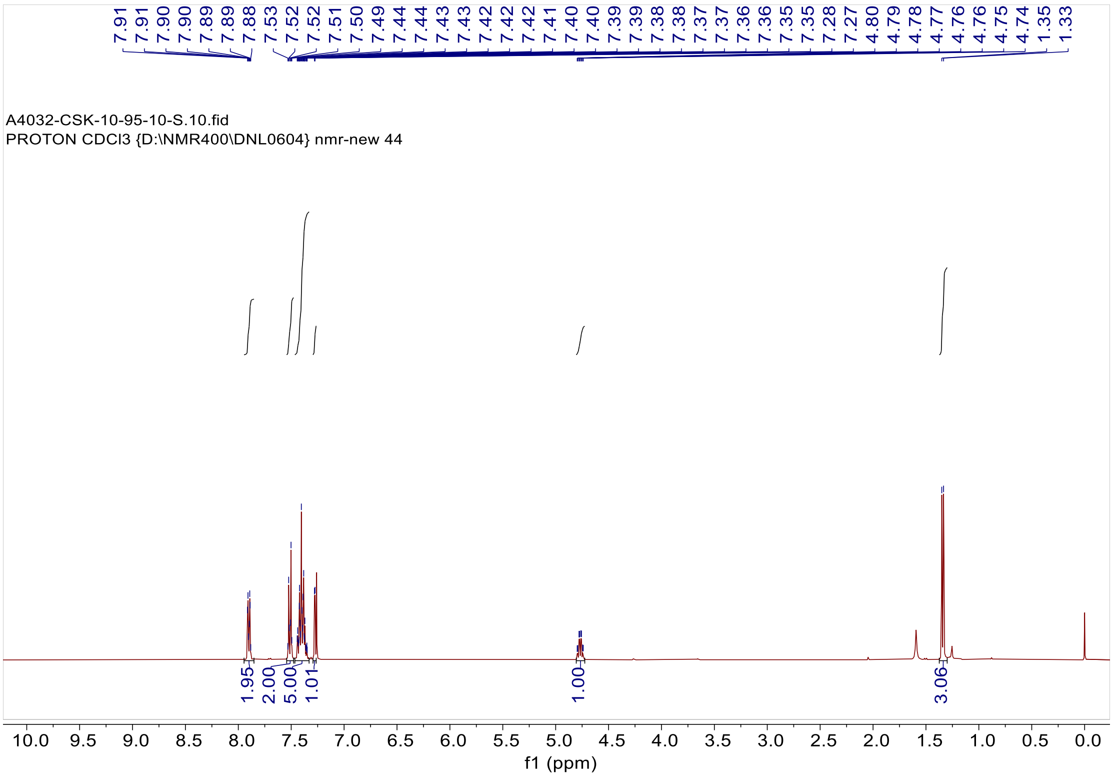

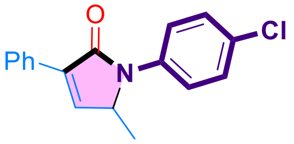

7f

^{1}H NMR (400 MHz, $CDCl_3$)
^{13}C NMR (100 MHz, $CDCl_3$)

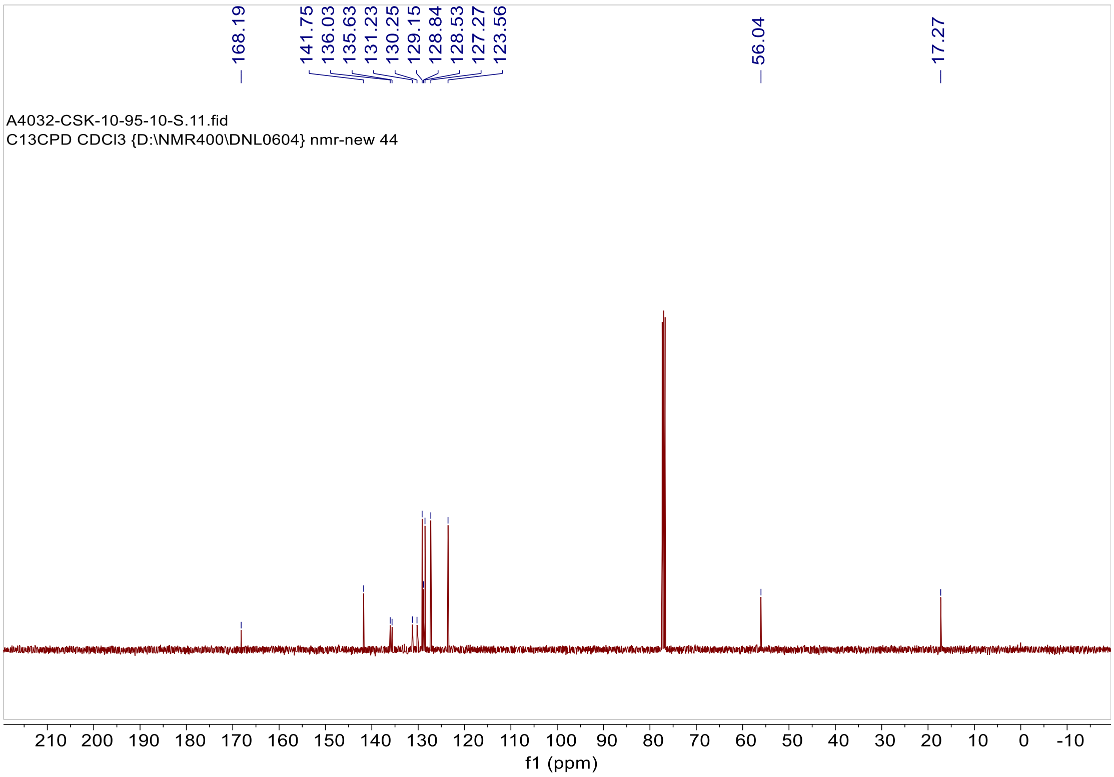

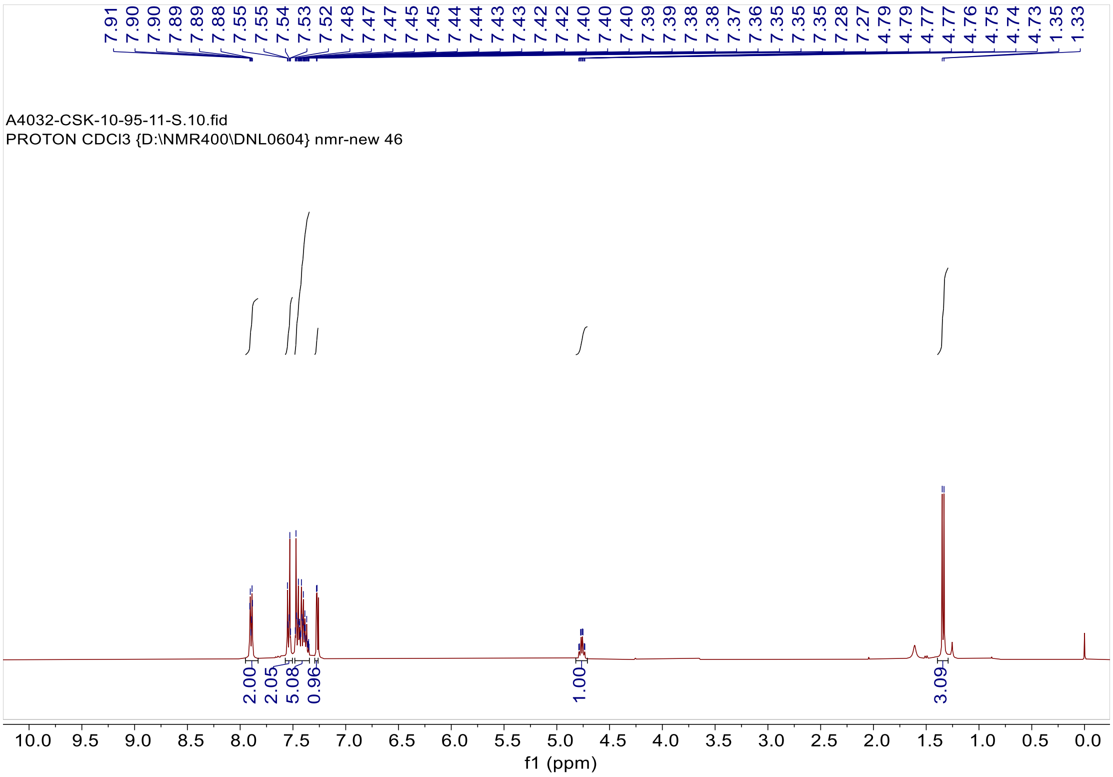

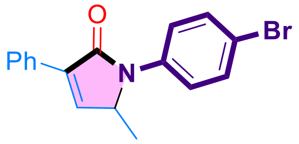

7g

^{1}H NMR (400 MHz, $CDCl_3$)
^{13}C NMR (100 MHz, $CDCl_3$)

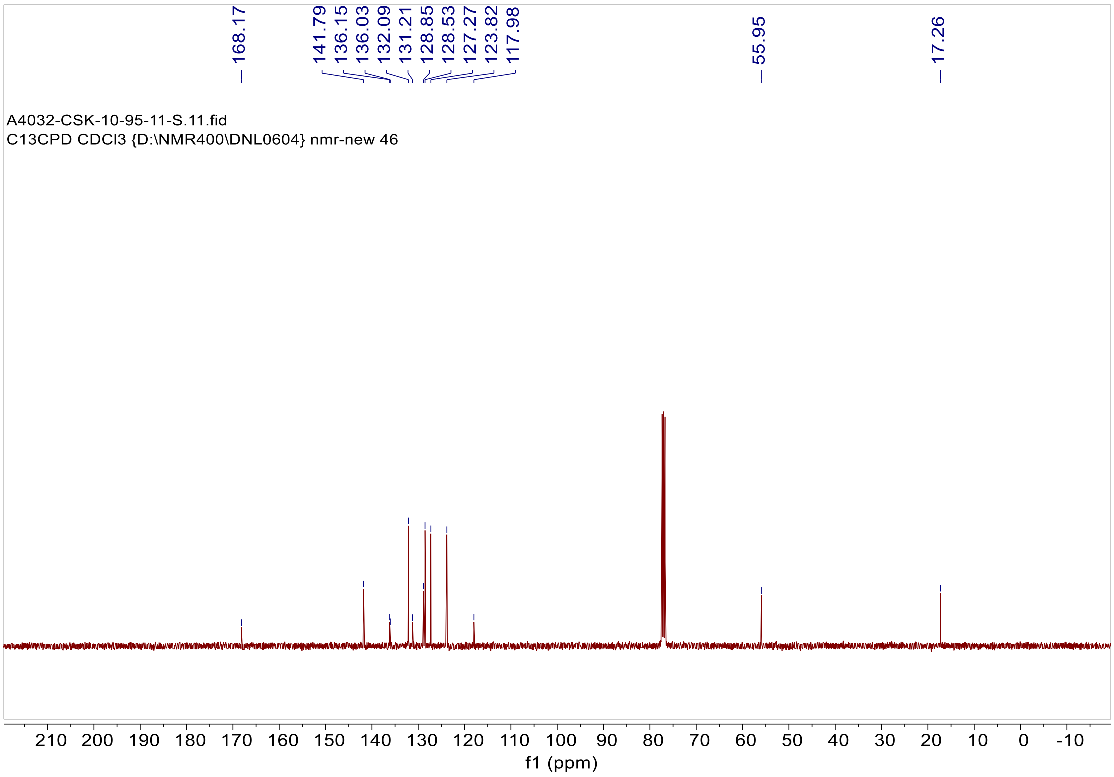

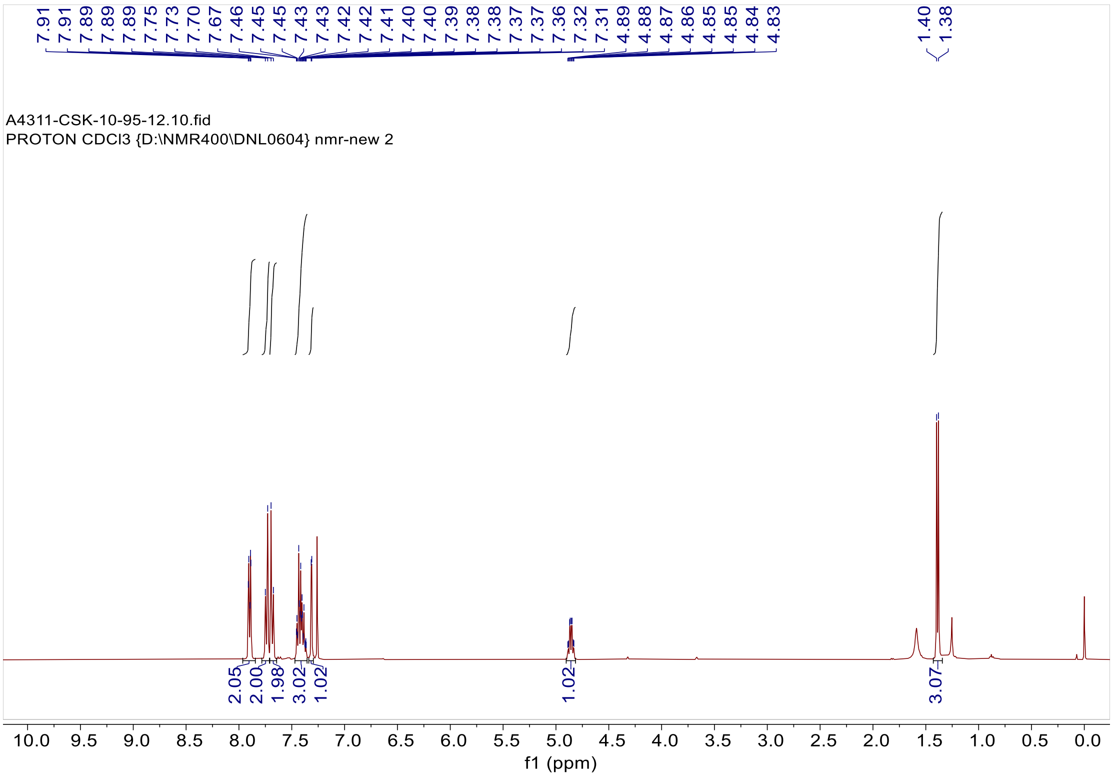

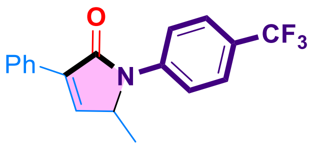

7h

^{1}H NMR (400 MHz, $CDCl_3$)
^{13}C NMR (100 MHz, $CDCl_3$)

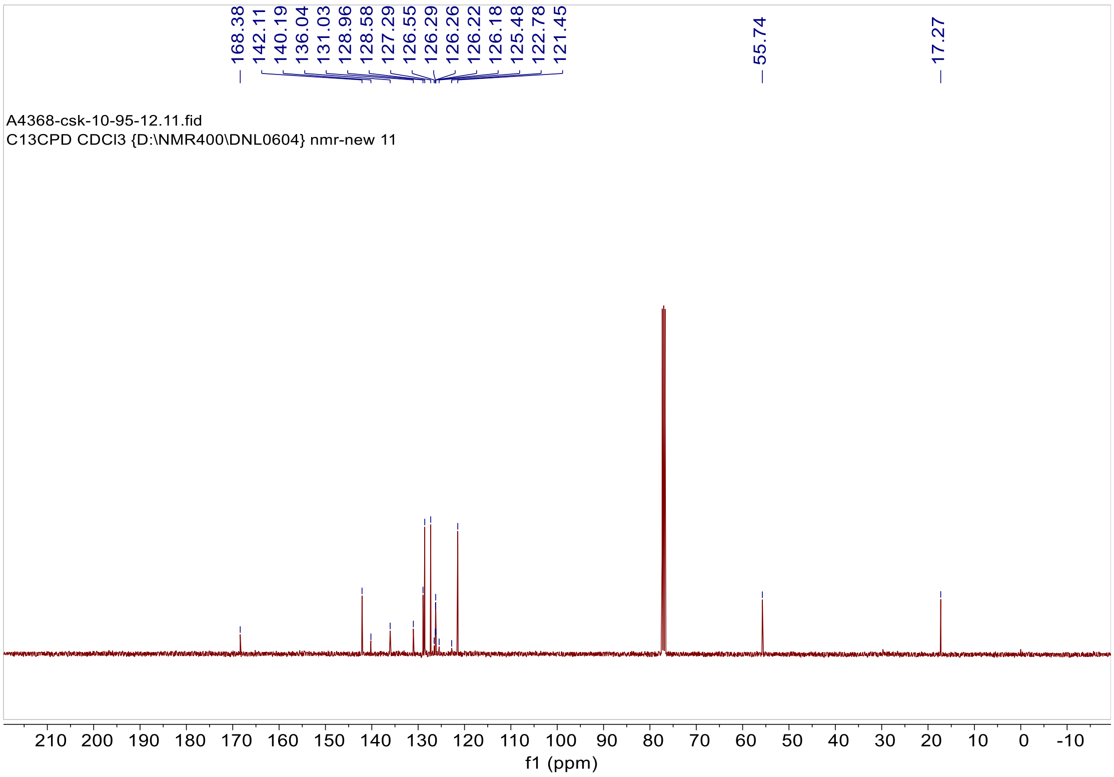

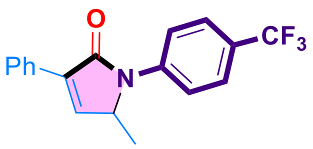

7h

^{19}F NMR (376 MHz, $CDCl_3$)

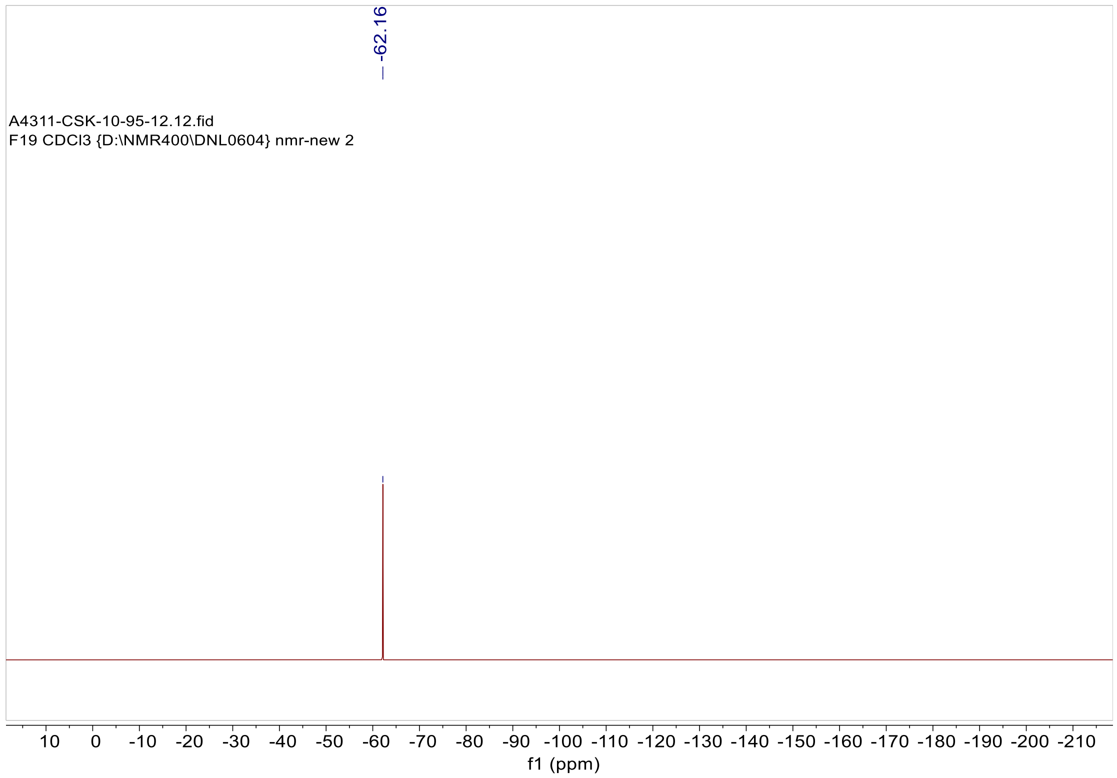

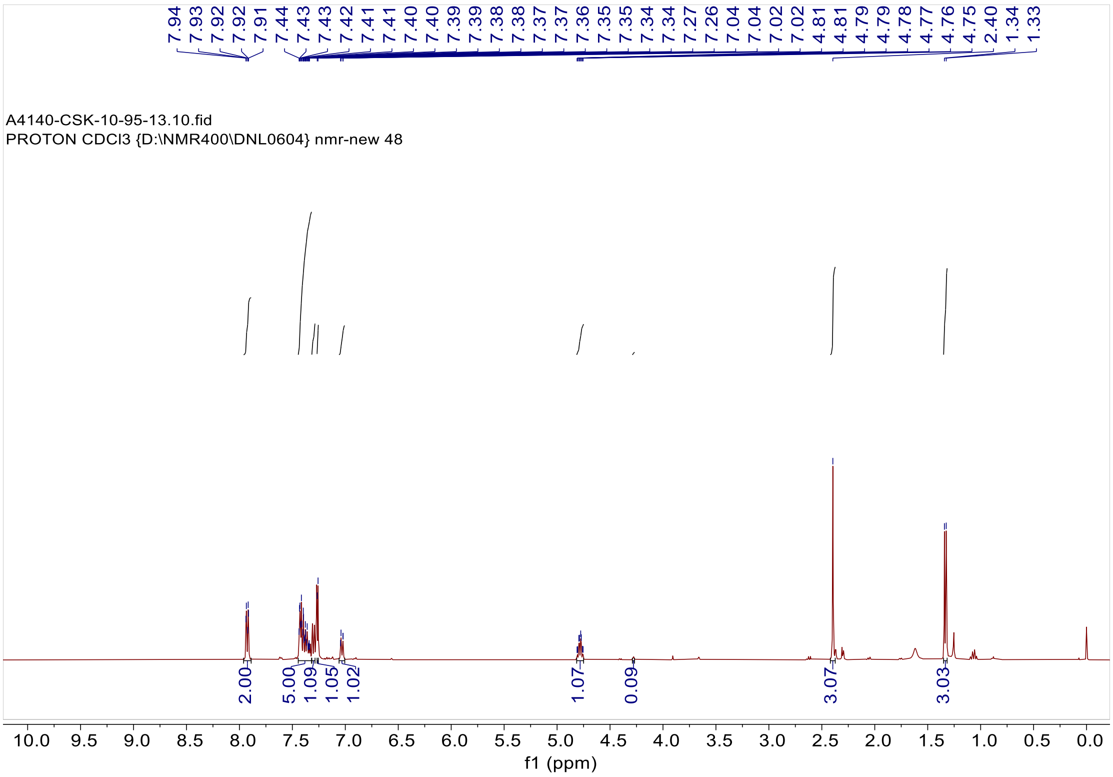

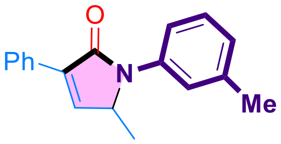

7i

^{1}H NMR (400 MHz, $CDCl_3$)

^{13}C NMR (100 MHz, $CDCl_3$)

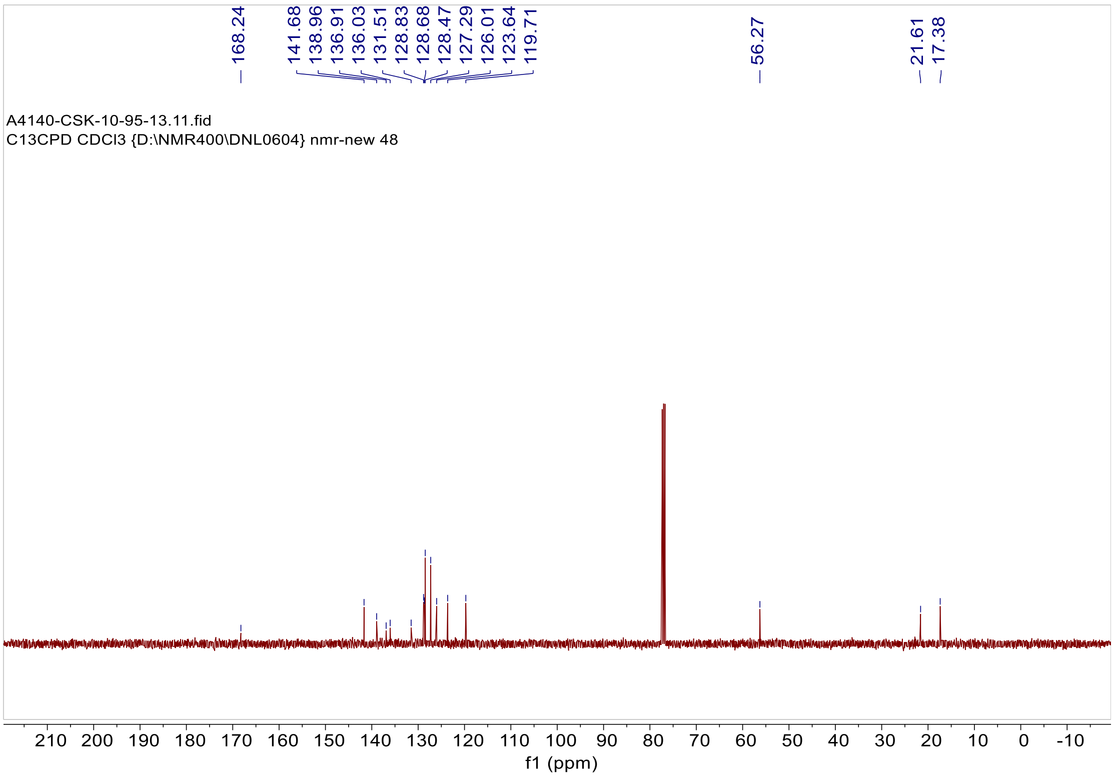

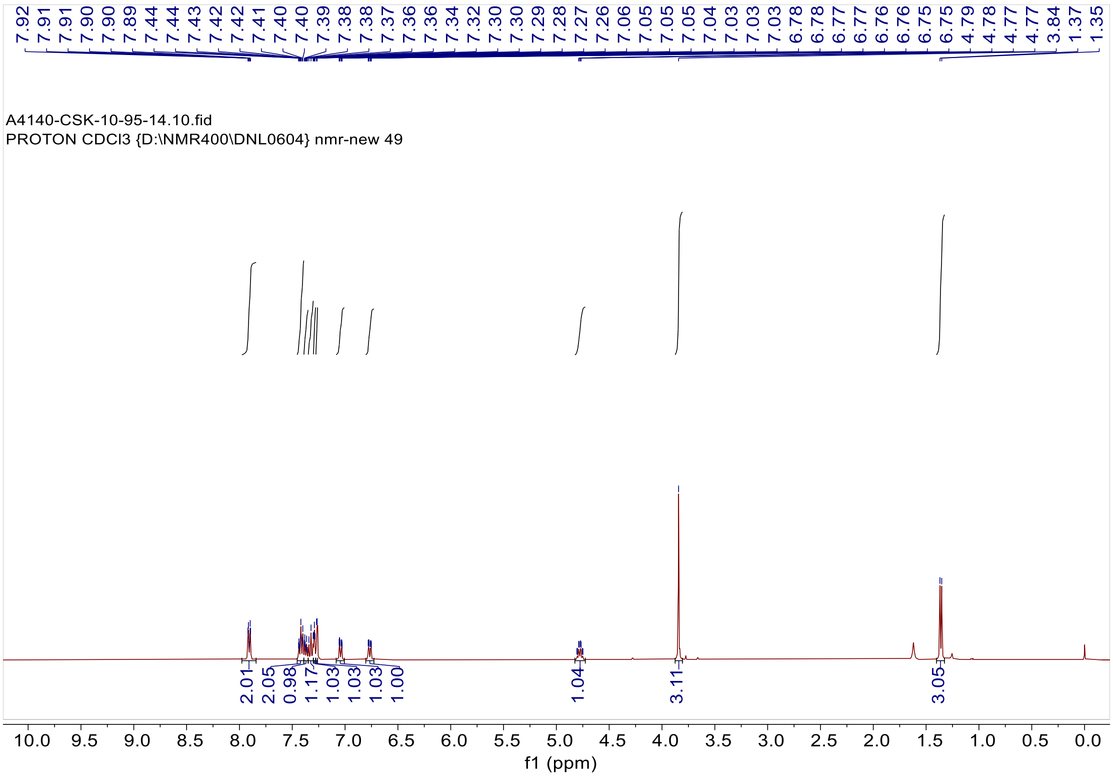

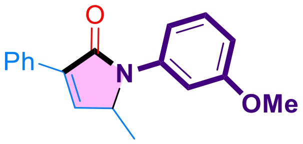

7j

^{1}H NMR (400 MHz, $CDCl_3$)
^{13}C NMR (100 MHz, $CDCl_3$)

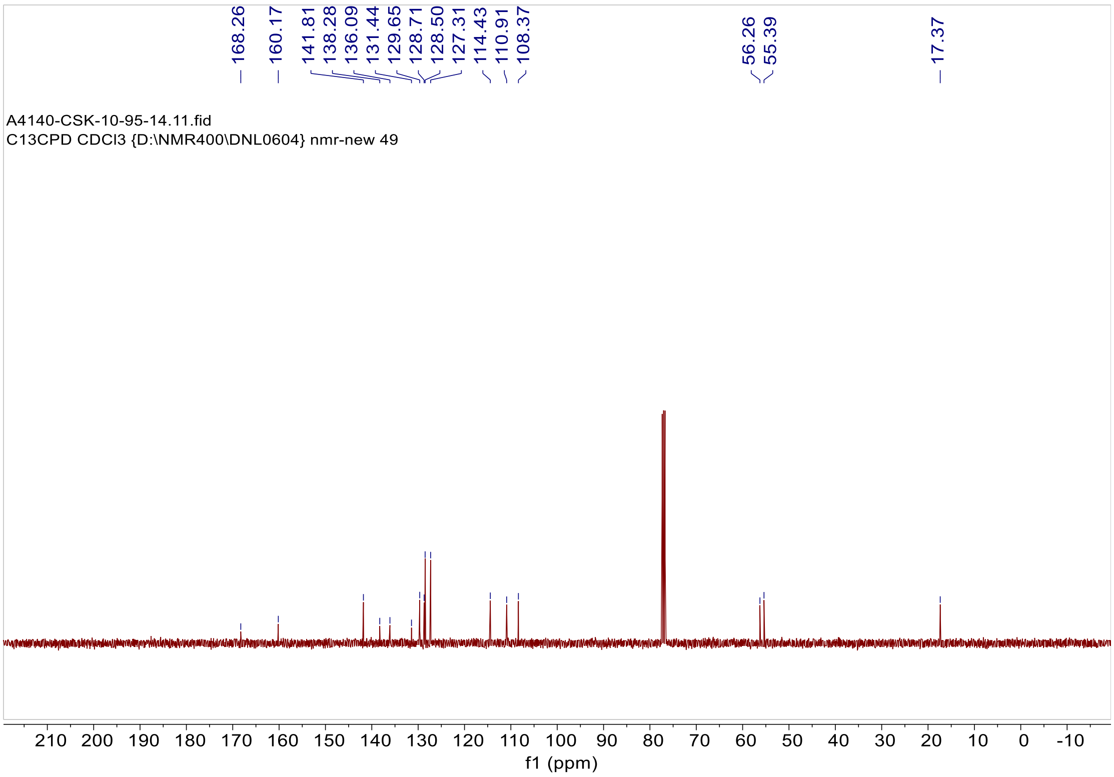

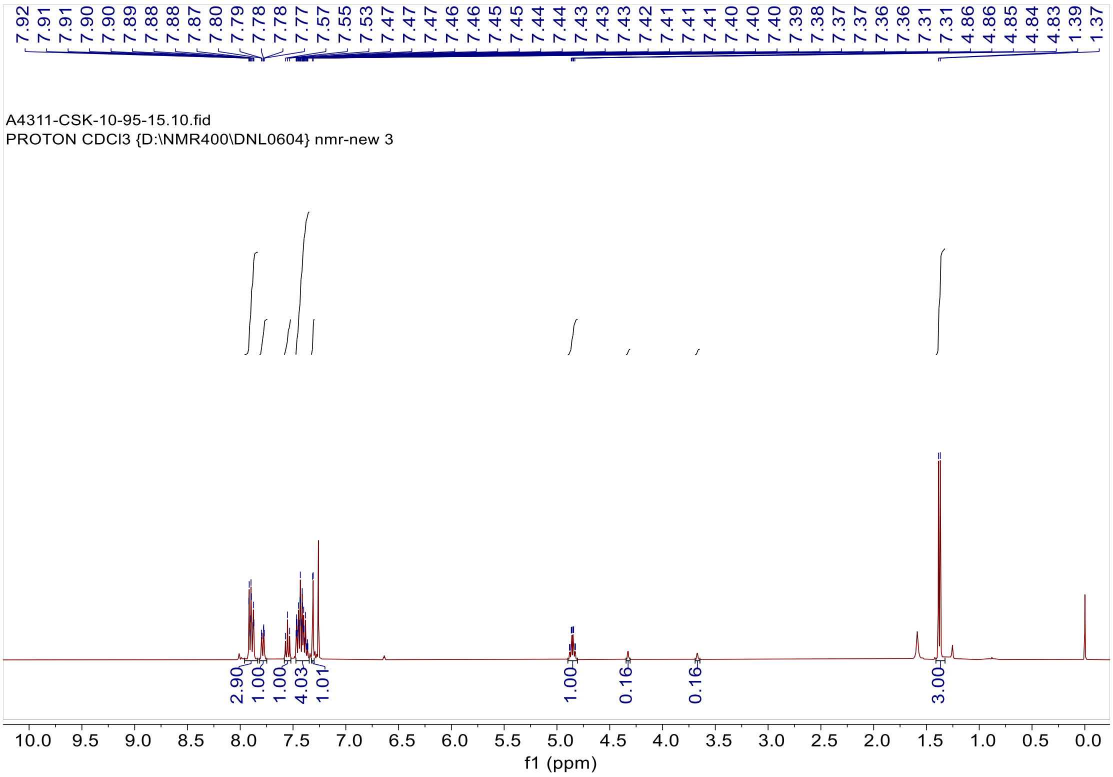

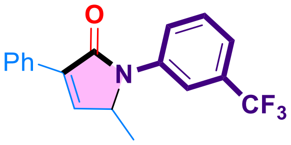

7k

^{1}H NMR (400 MHz, $CDCl_3$)
^{13}C NMR (100 MHz, $CDCl_3$)

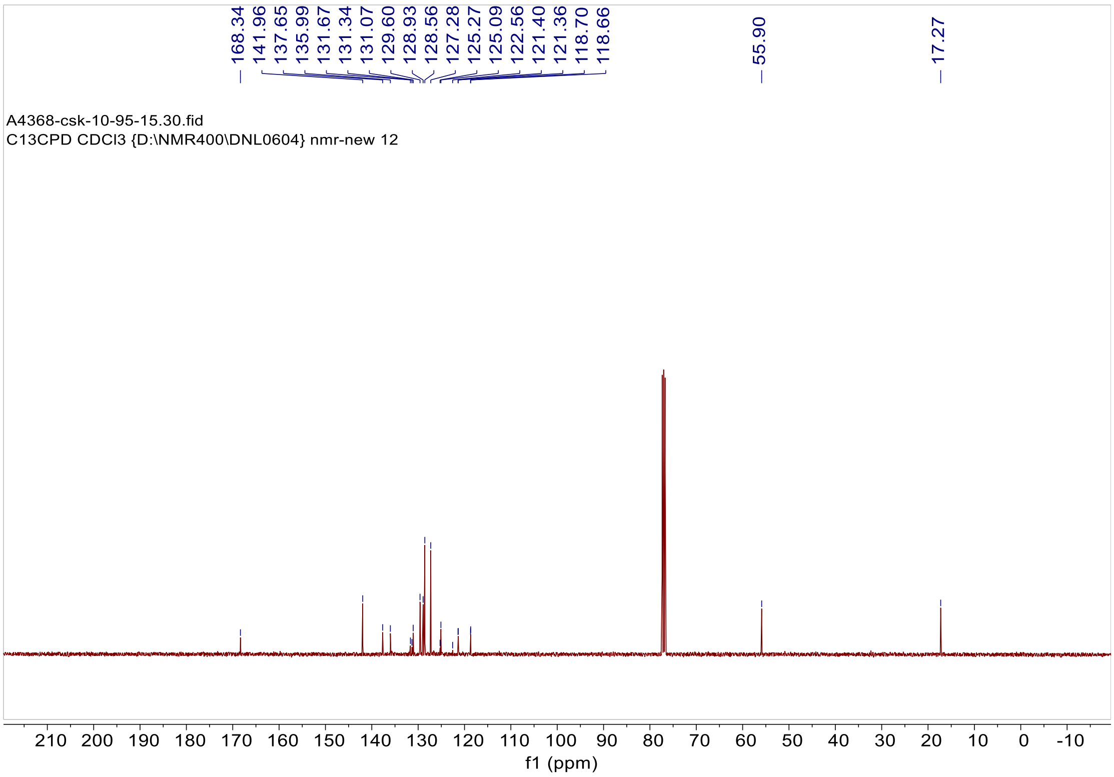

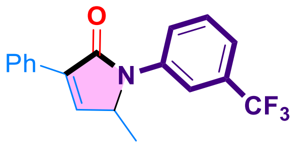

7k

^{19}F NMR (376 MHz, $CDCl_3$)

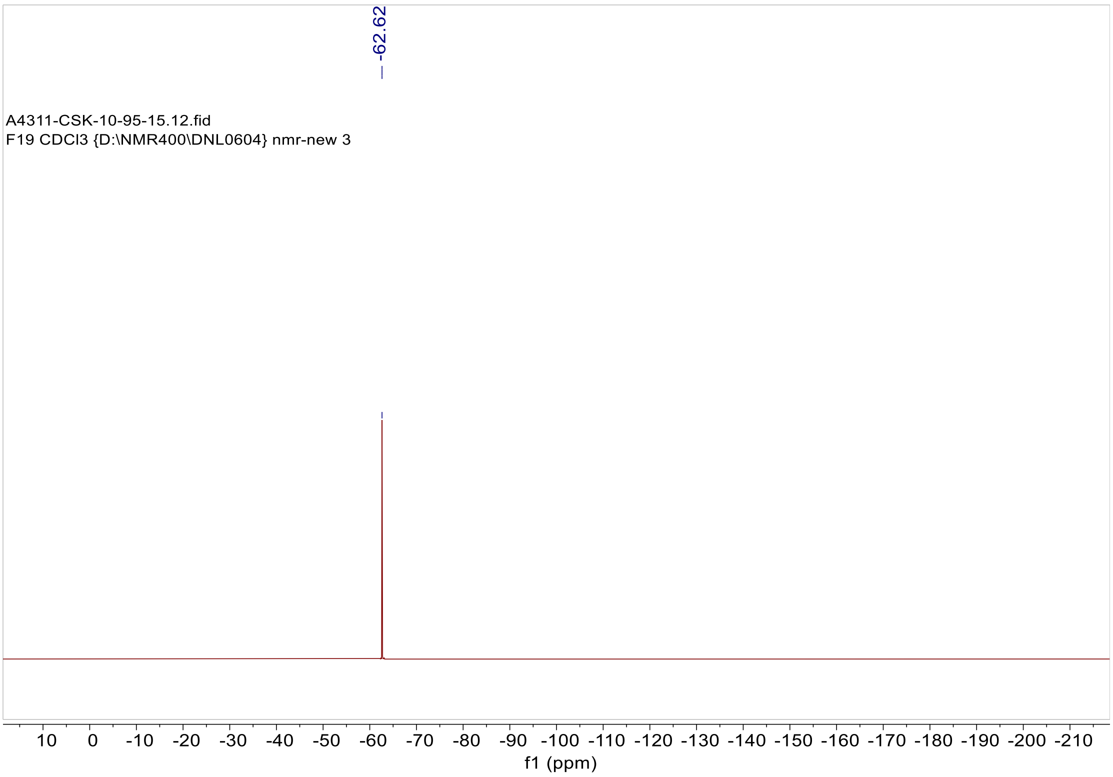

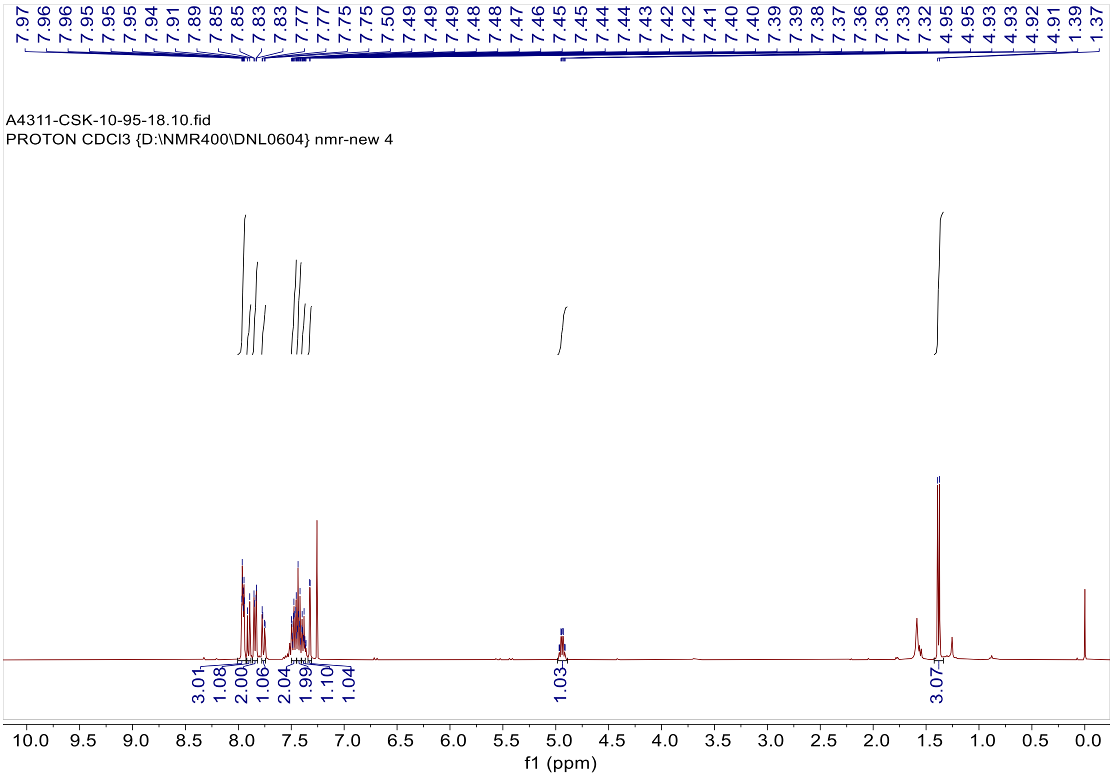

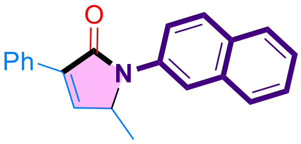

7l

^{1}H NMR (400 MHz, CDCl$_3$)
^{13}C NMR (100 MHz, CDCl$_3$)

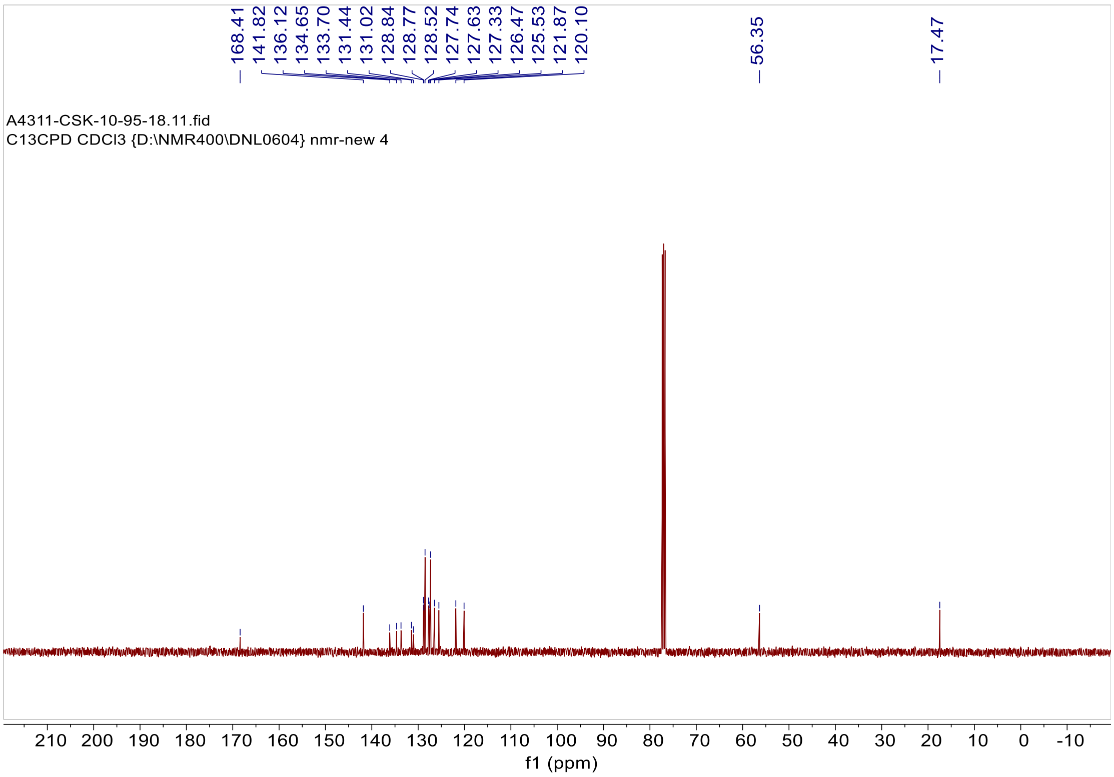

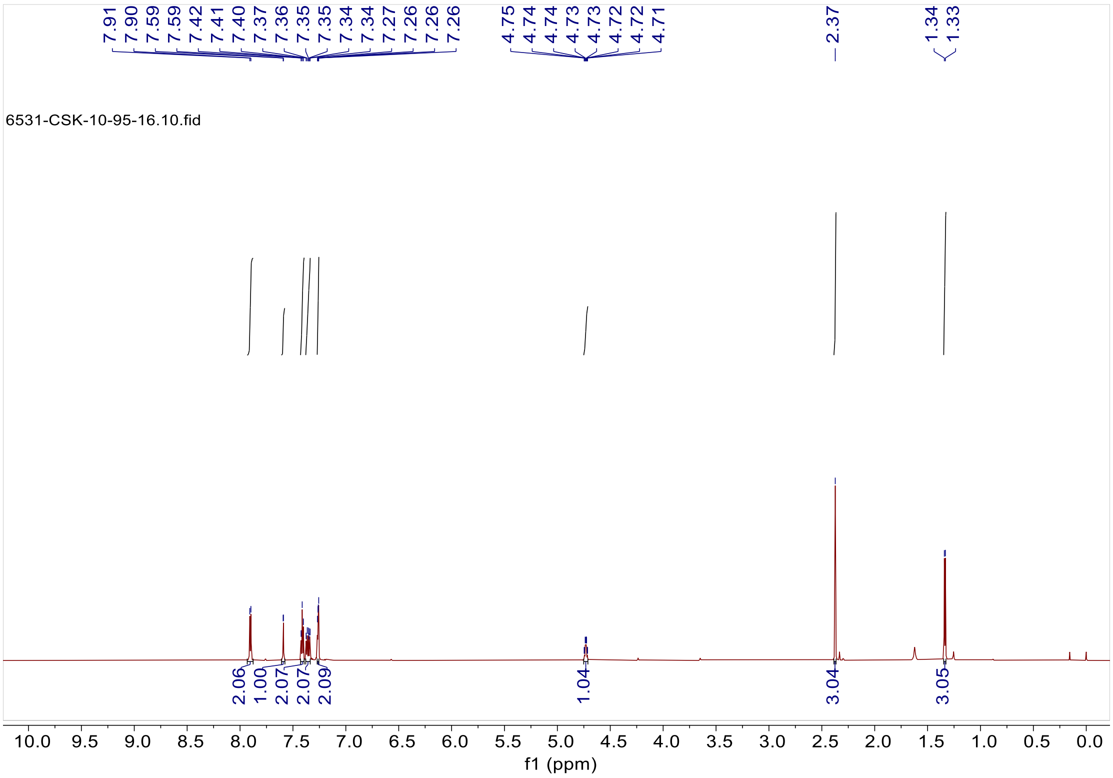

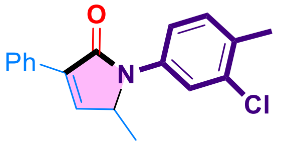

7m

^{1}H NMR (700 MHz, $CDCl_3$)
^{13}C NMR (176 MHz, $CDCl_3$)

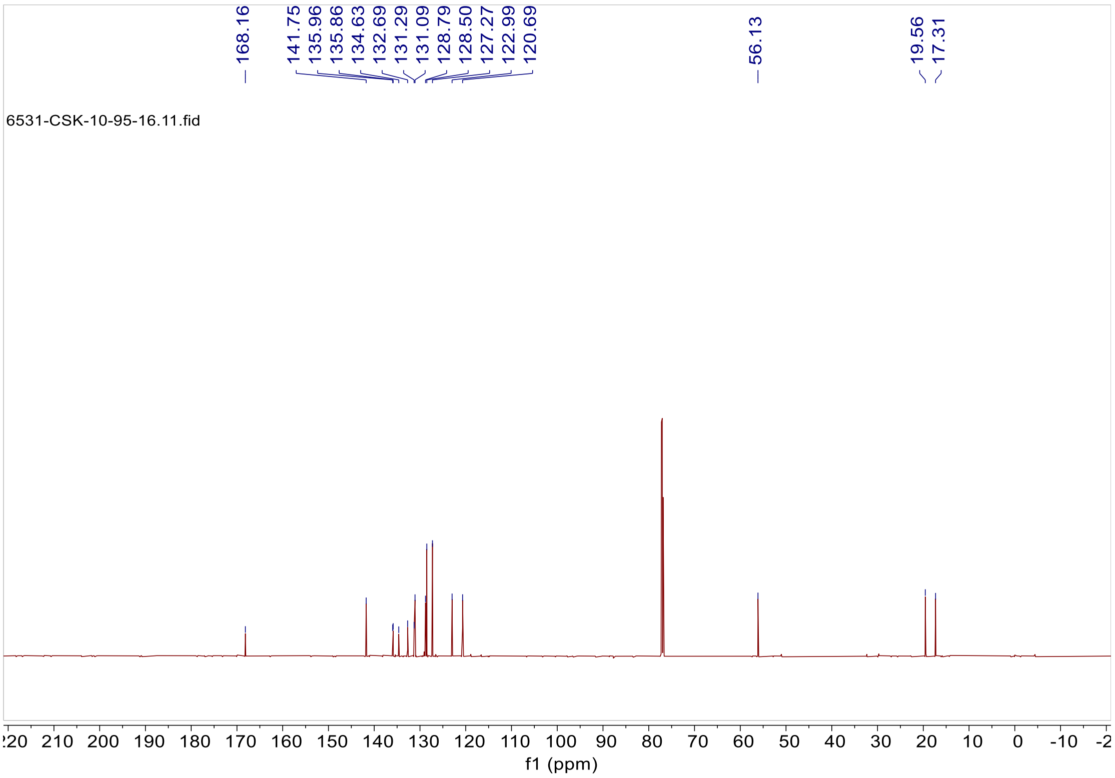

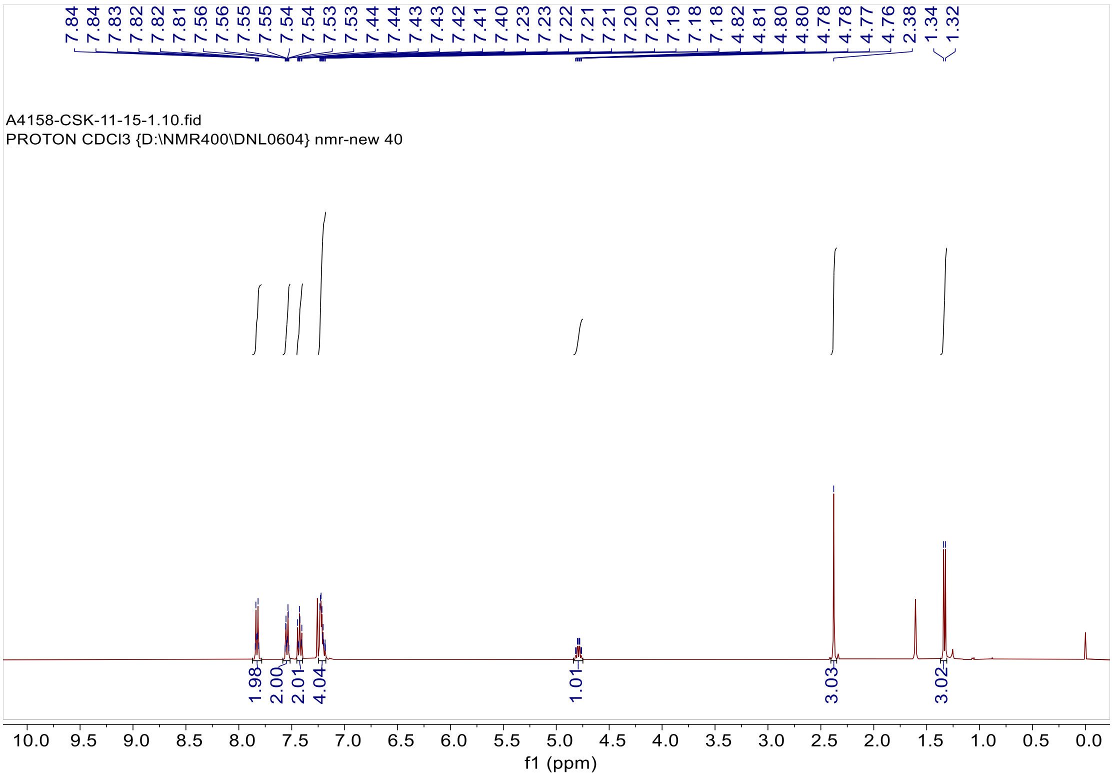

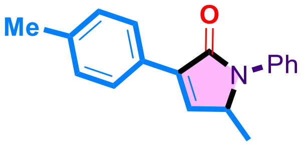

7o

^{1}H NMR (400 MHz, $CDCl_3$)

^{13}C NMR (100 MHz, $CDCl_3$)

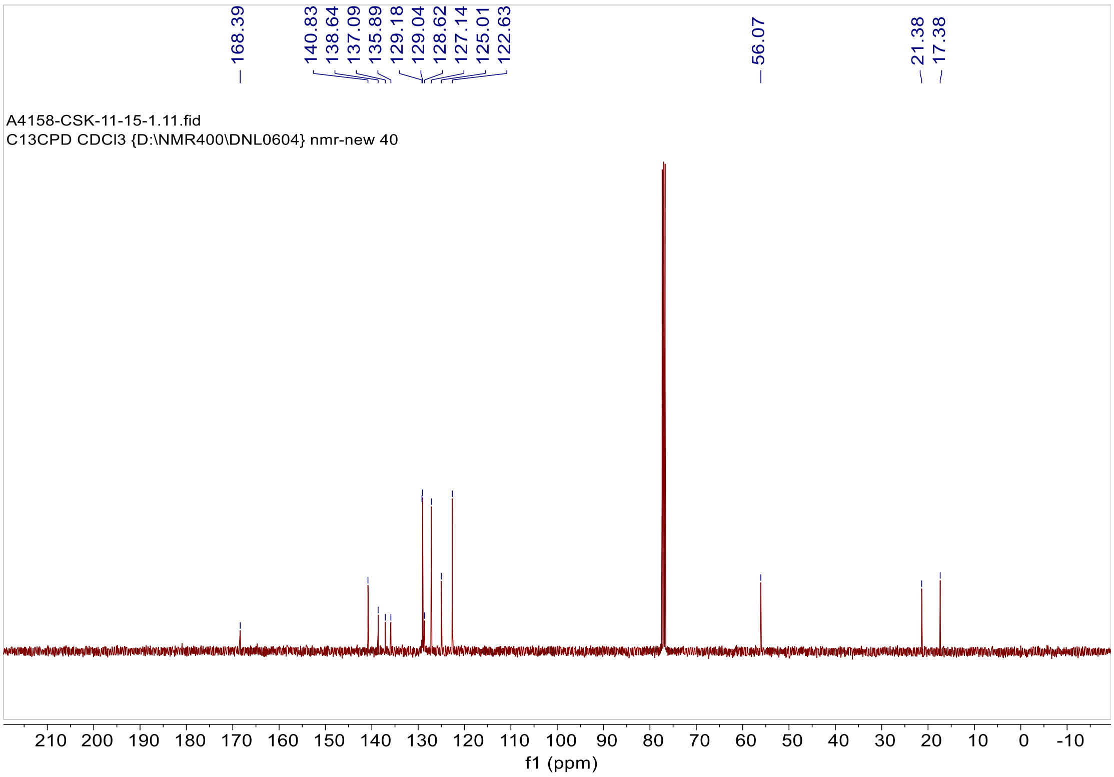

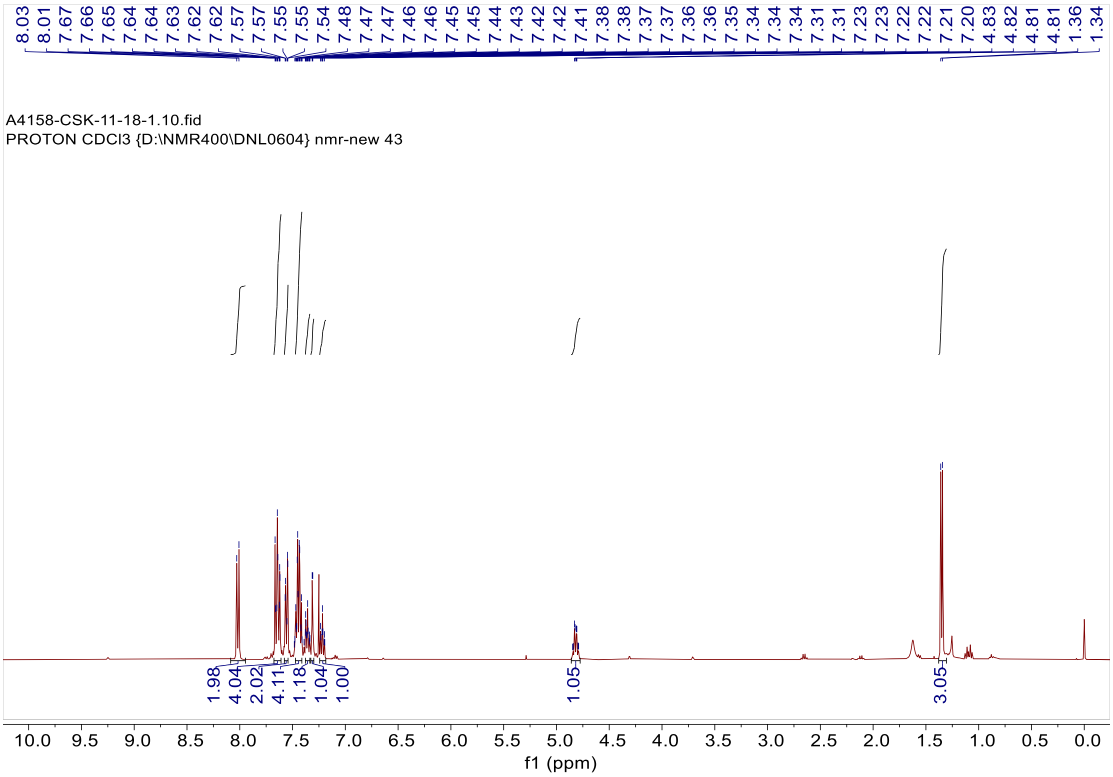

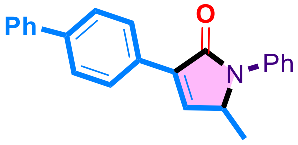

7p

^{1}H NMR (400 MHz, $CDCl_3$)

^{13}C NMR (100 MHz, $CDCl_3$)

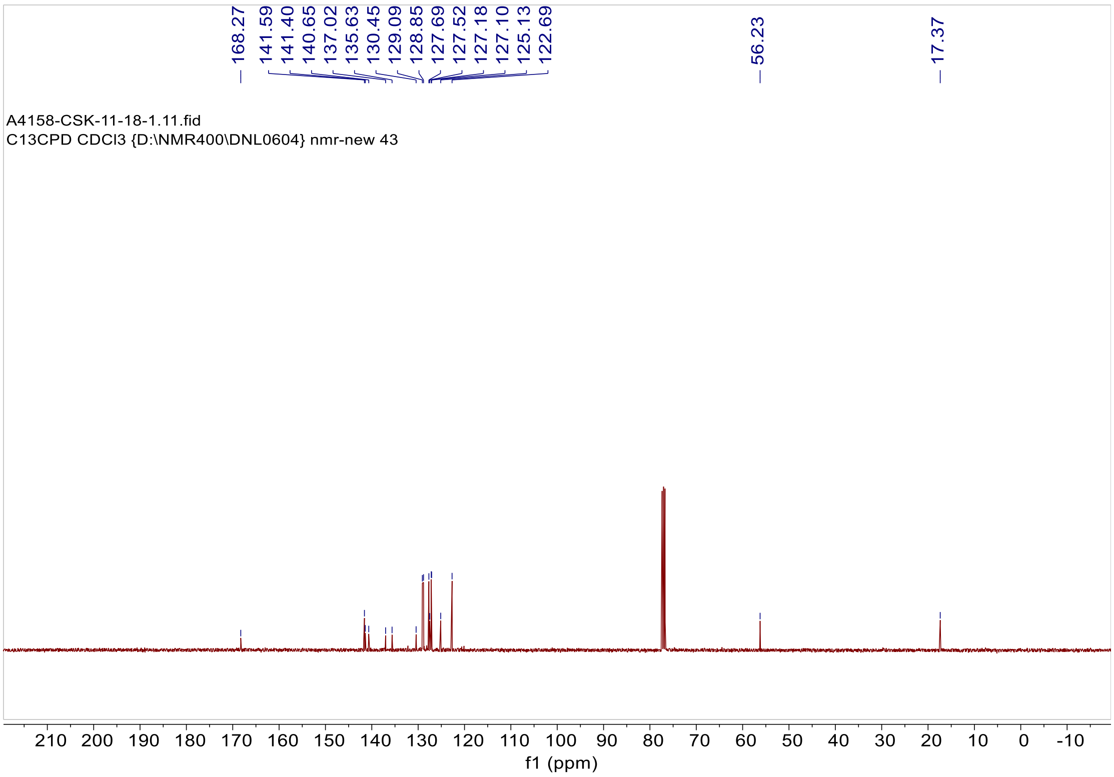

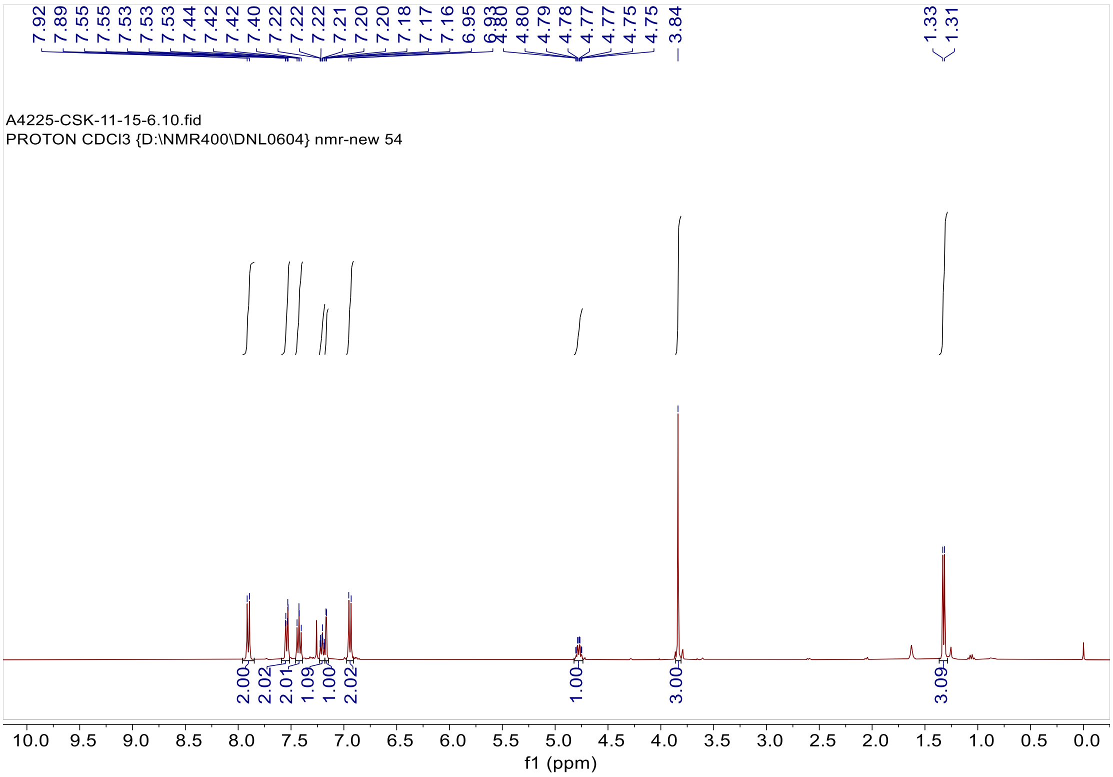

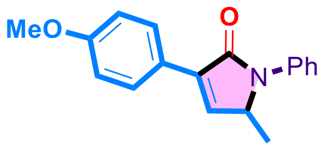

7q

^{1}H NMR (400 MHz, $CDCl_3$)

^{13}C NMR (100 MHz, $CDCl_3$)

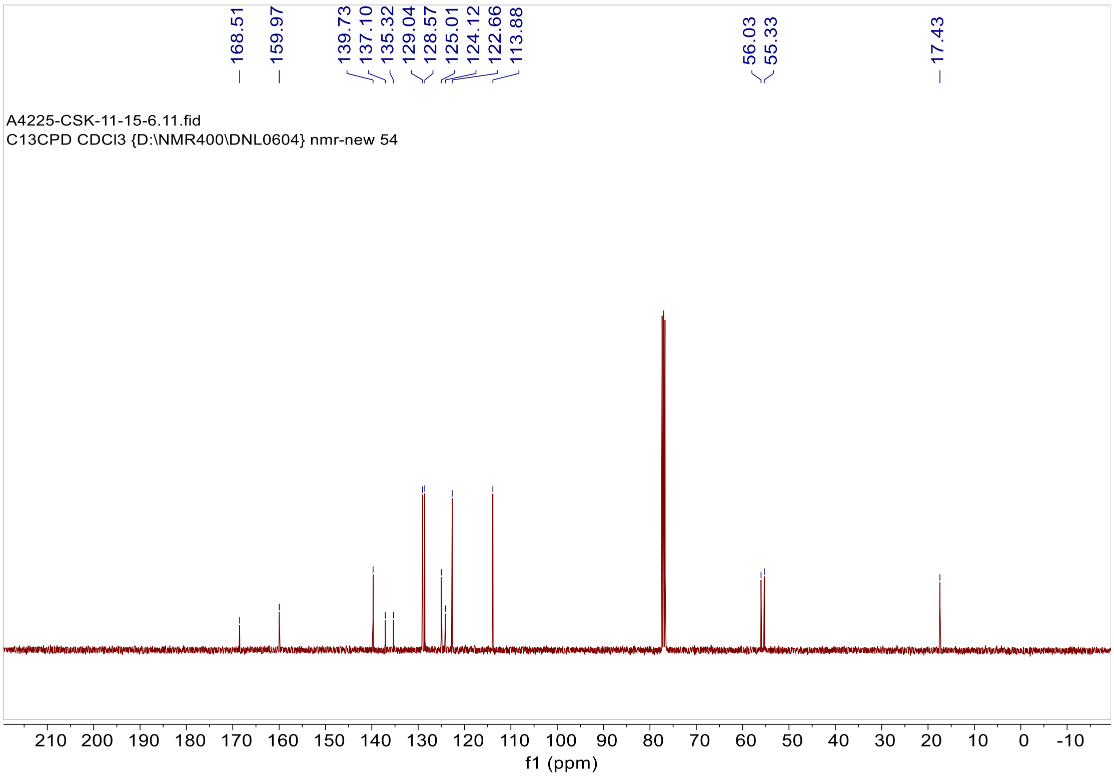

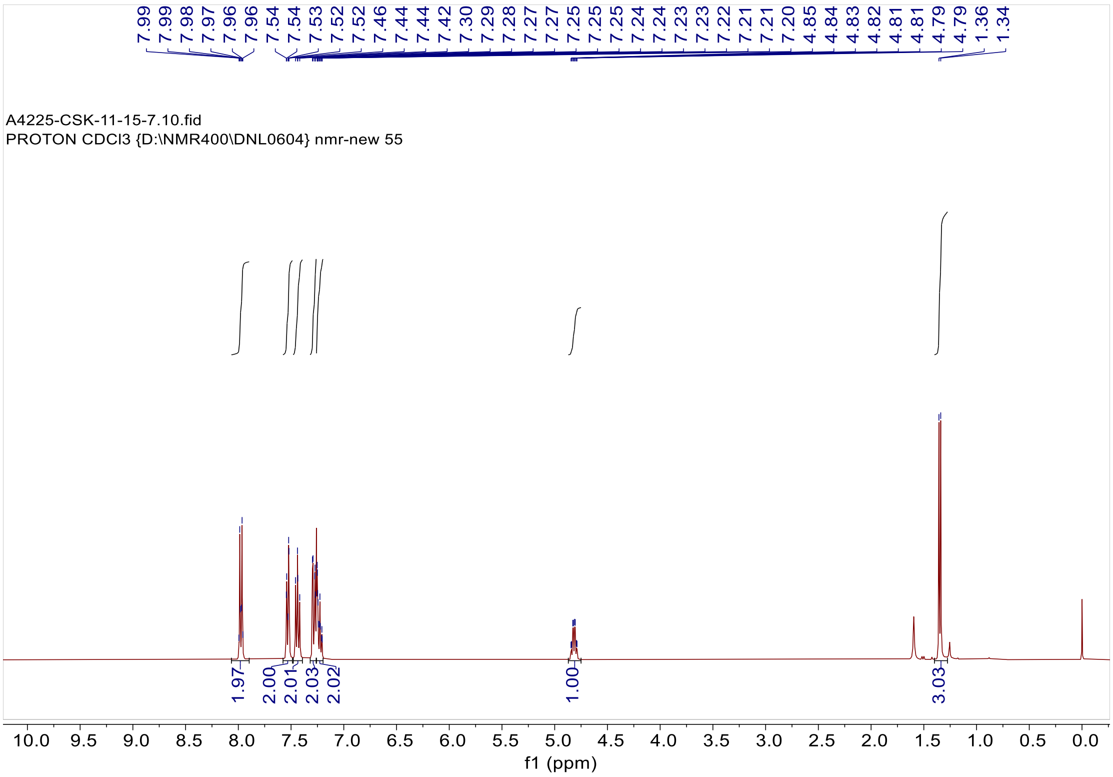

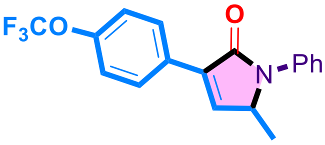

7r

^{1}H NMR (400 MHz, $CDCl_3$)

^{13}C NMR (100 MHz, $CDCl_3$)

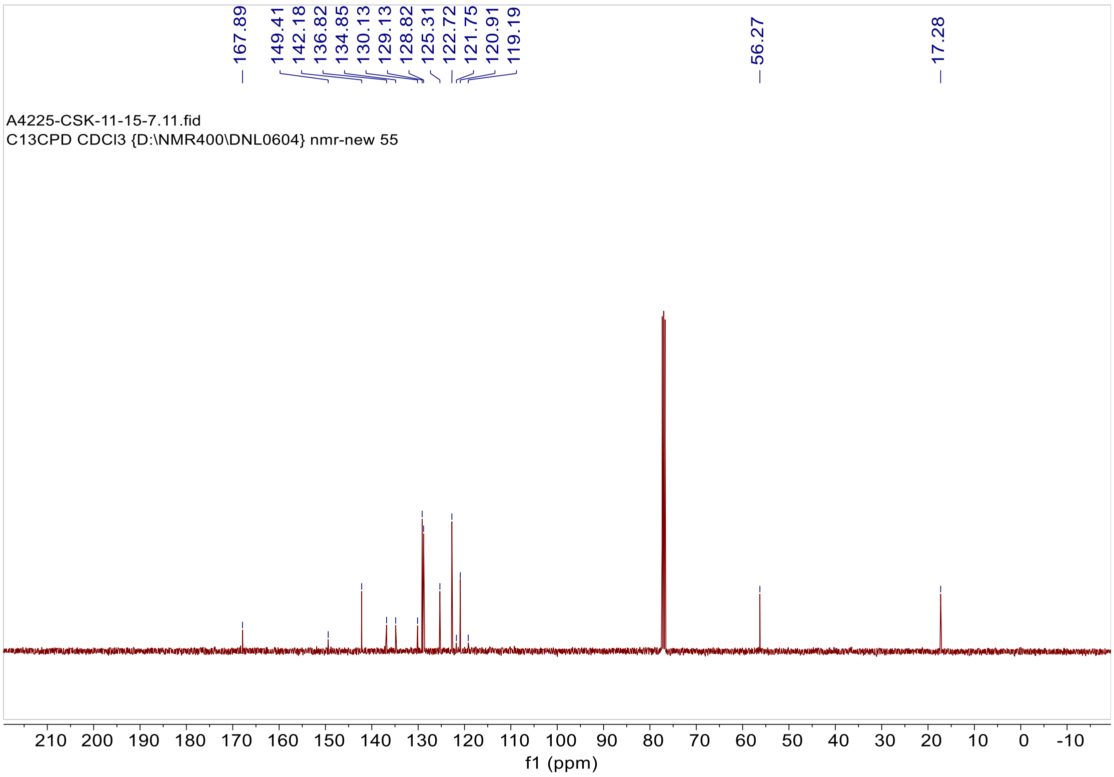

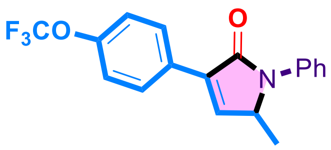

7r ^{19}F NMR (376 MHz, $CDCl_3$)

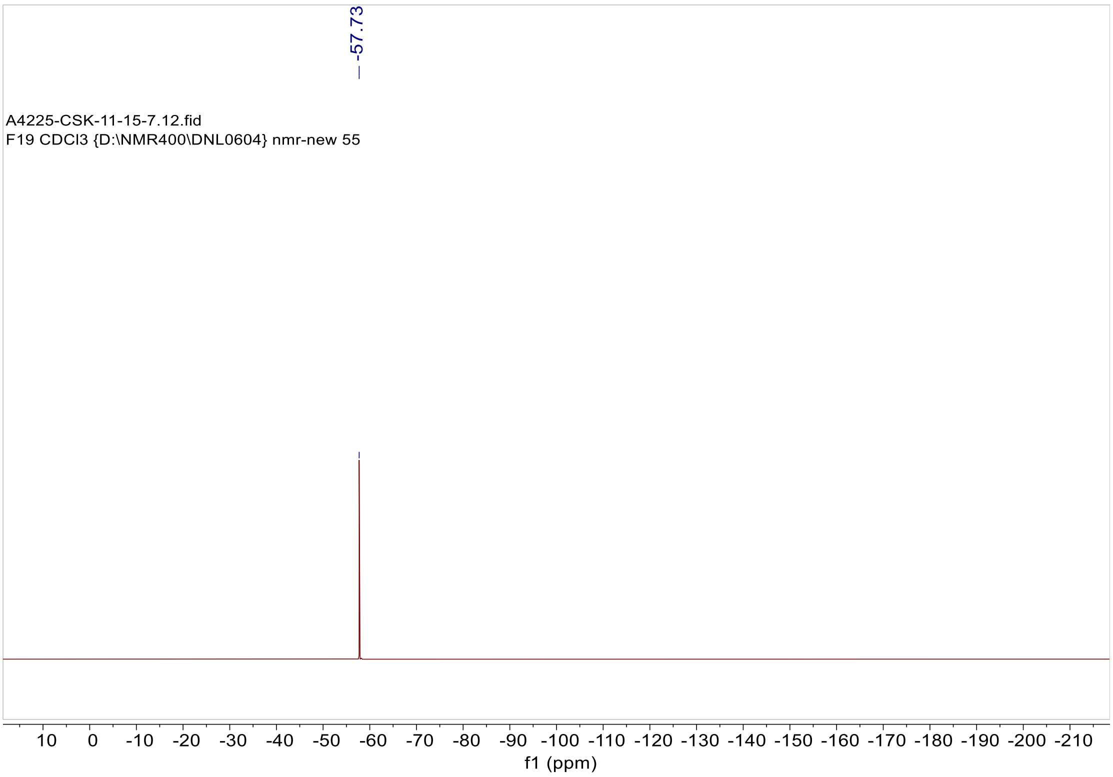

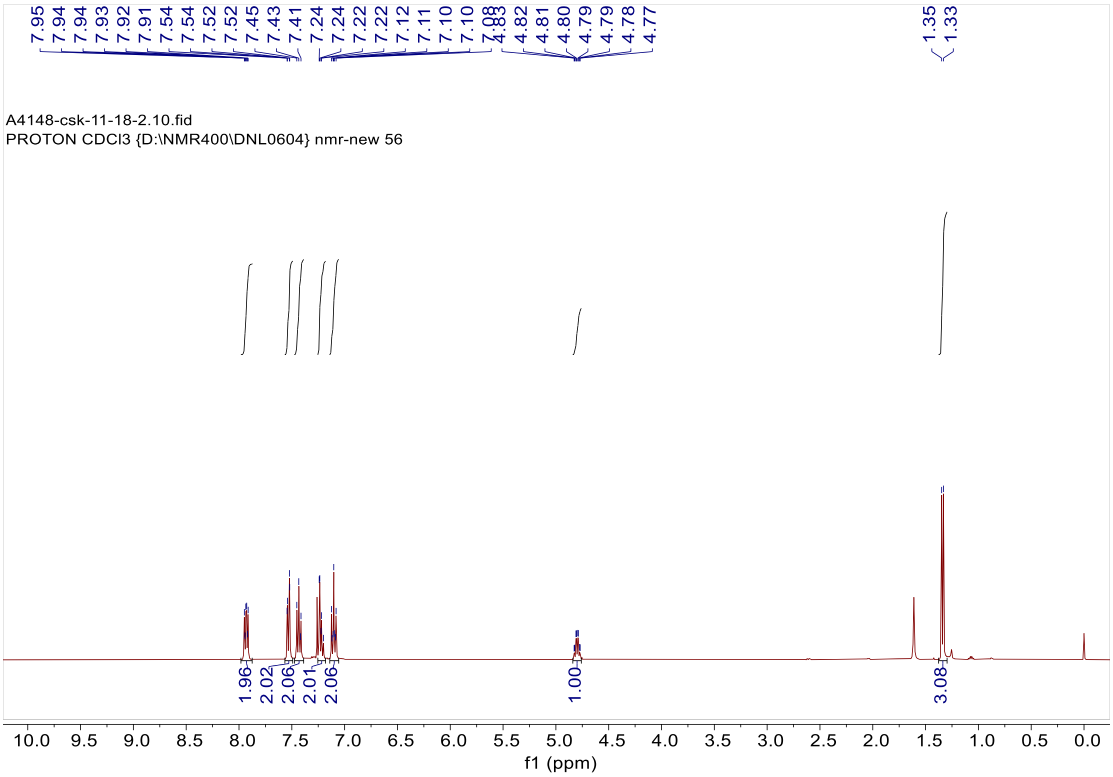

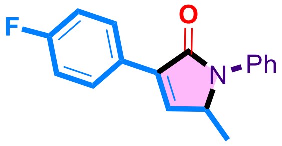

7s

^{1}H NMR (400 MHz, $CDCl_3$)
^{13}C NMR (100 MHz, $CDCl_3$)

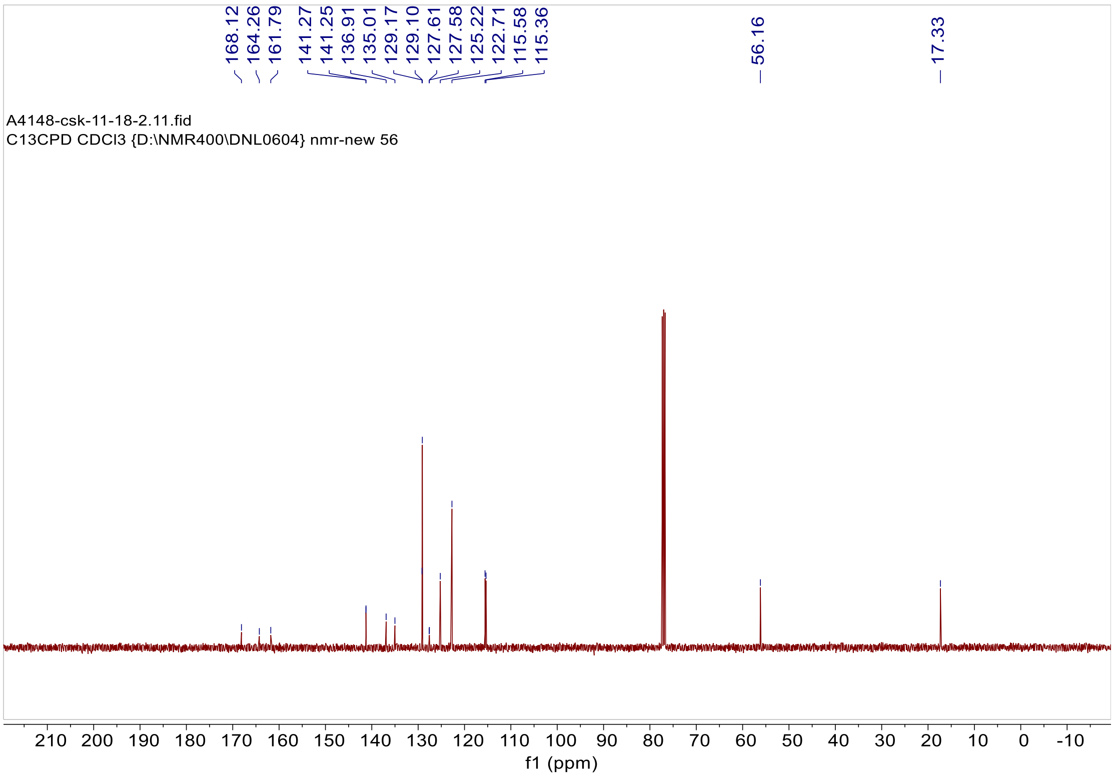

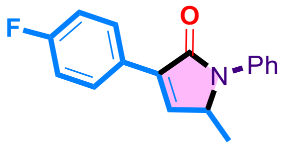

7s

^{19}F NMR (376 MHz, $CDCl_3$)

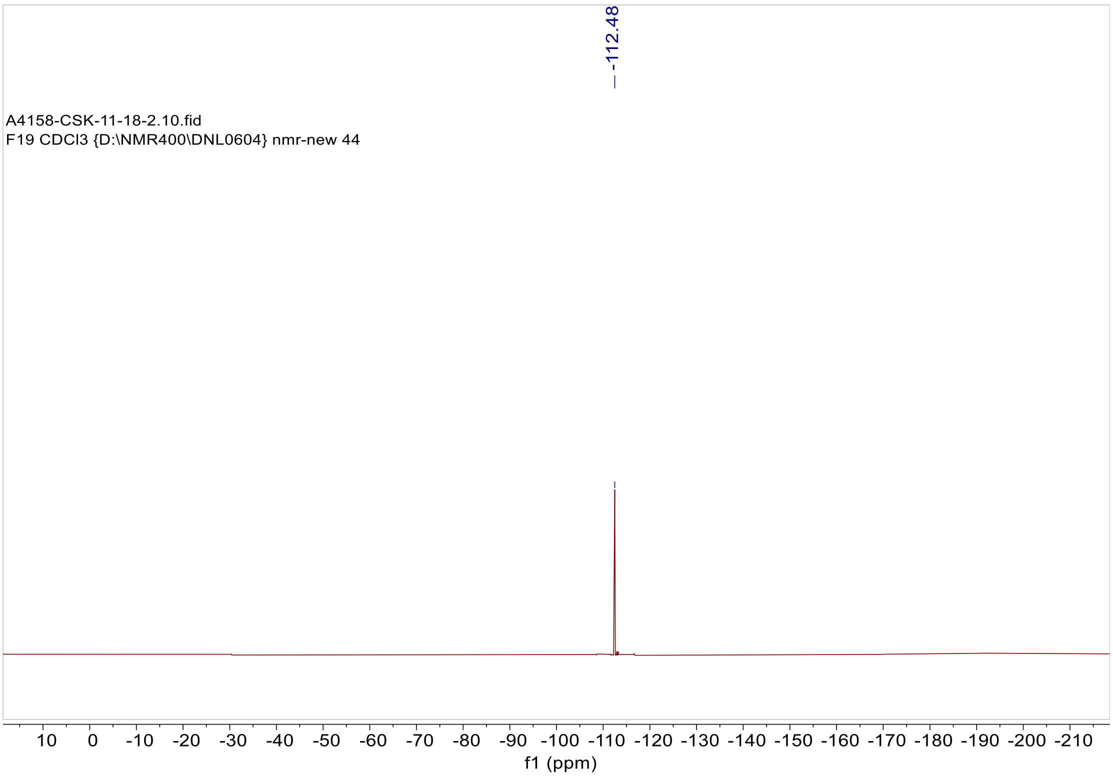

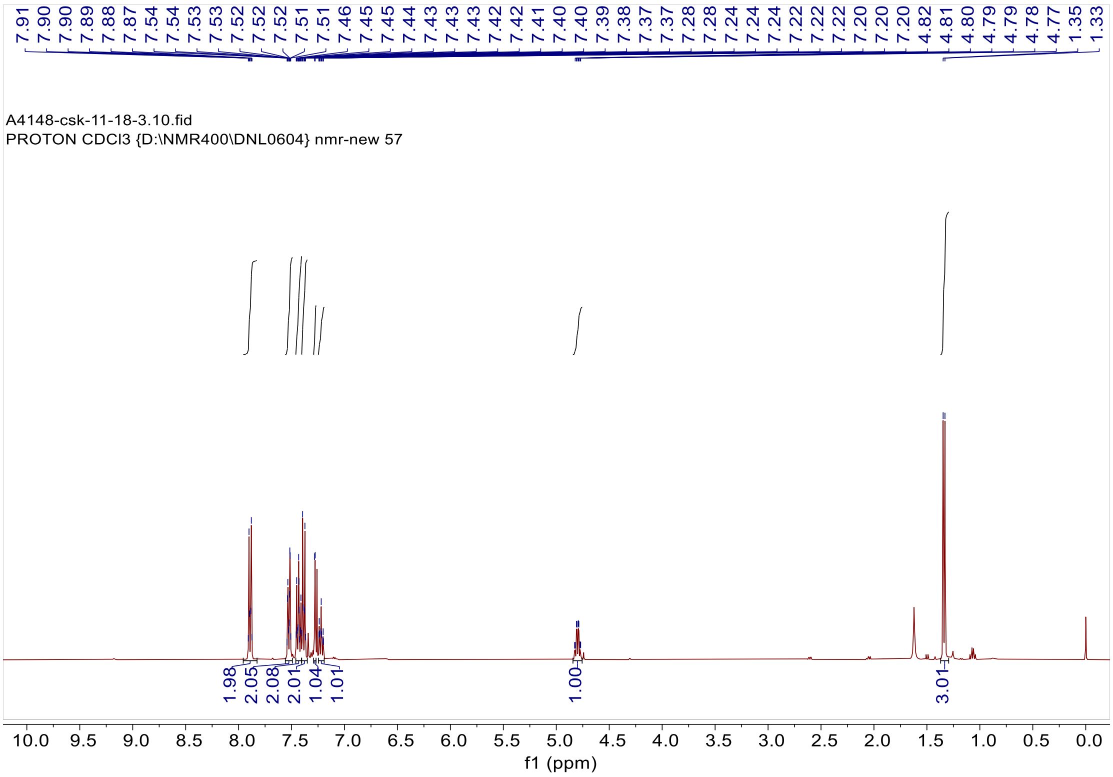

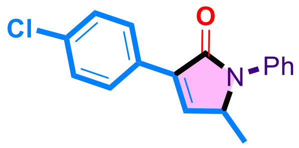

7t

^{1}H NMR (400 MHz, $CDCl_3$)
^{13}C NMR (100 MHz, $CDCl_3$)

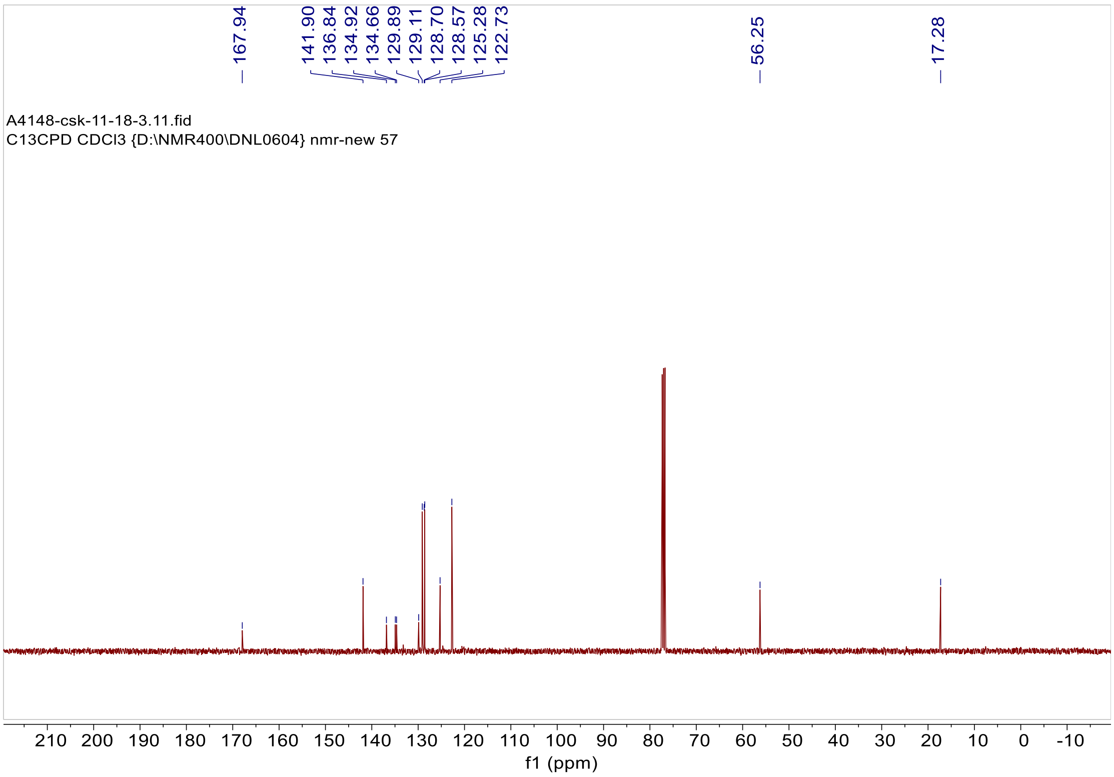

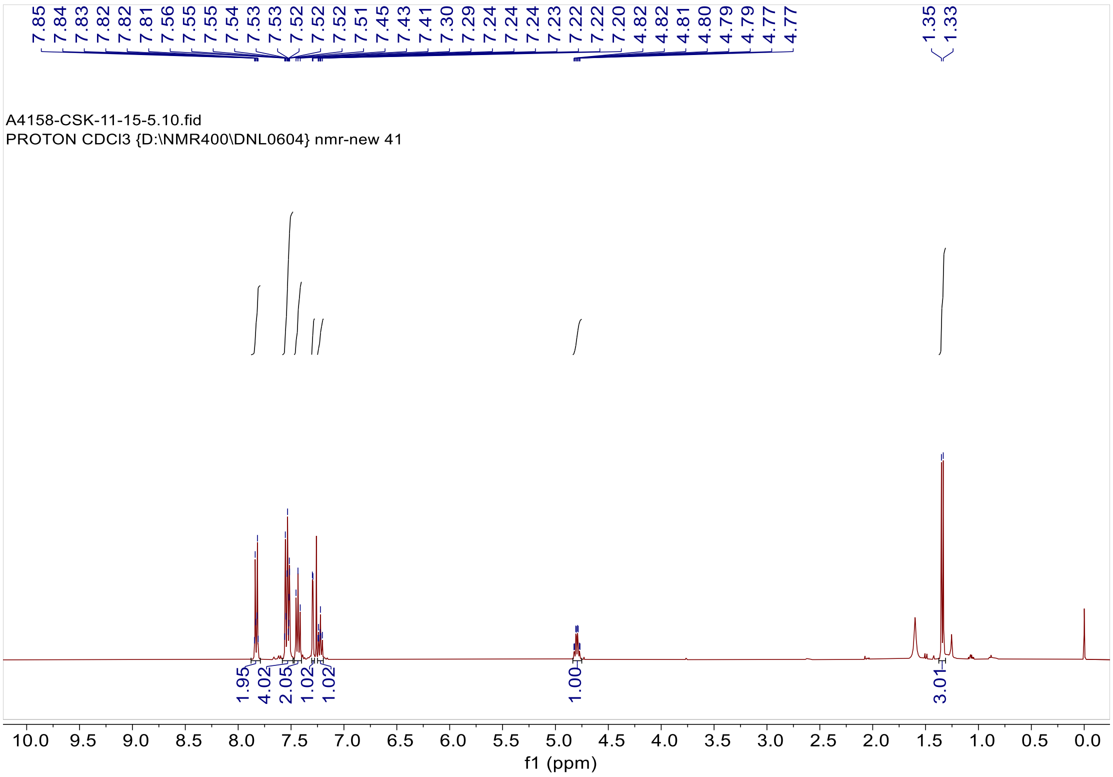

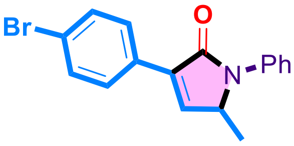

7u

^{1}H NMR (400 MHz, $CDCl_3$)
^{13}C NMR (100 MHz, $CDCl_3$)

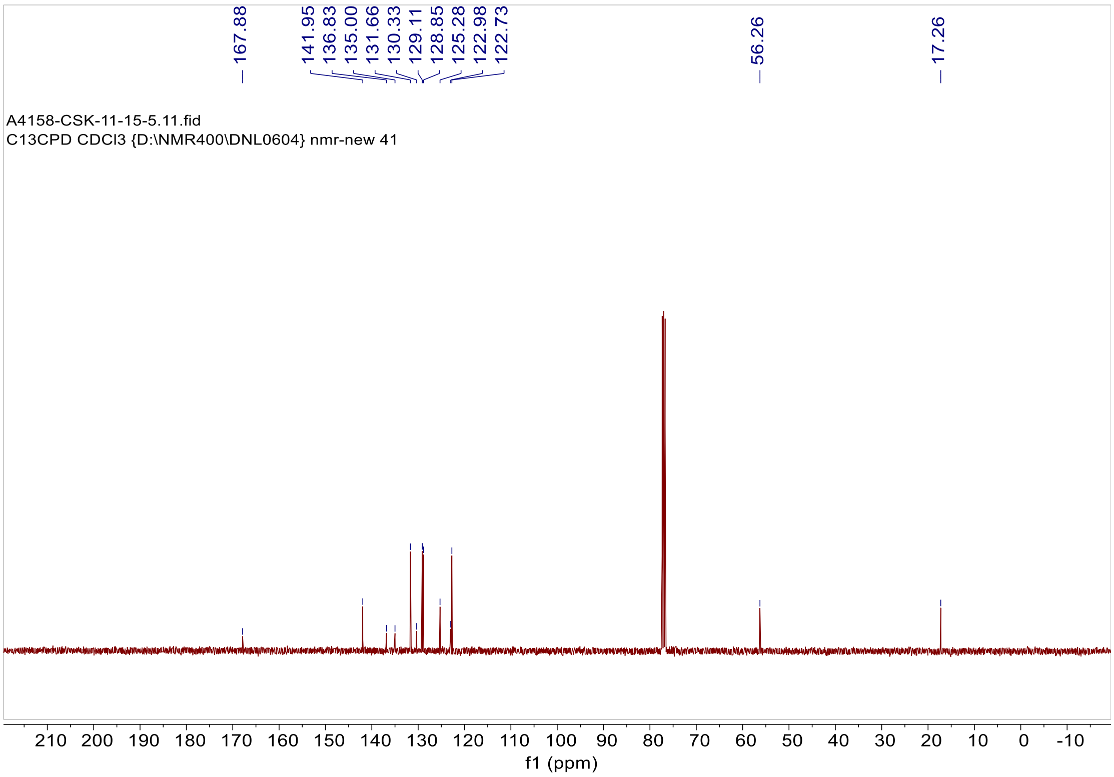

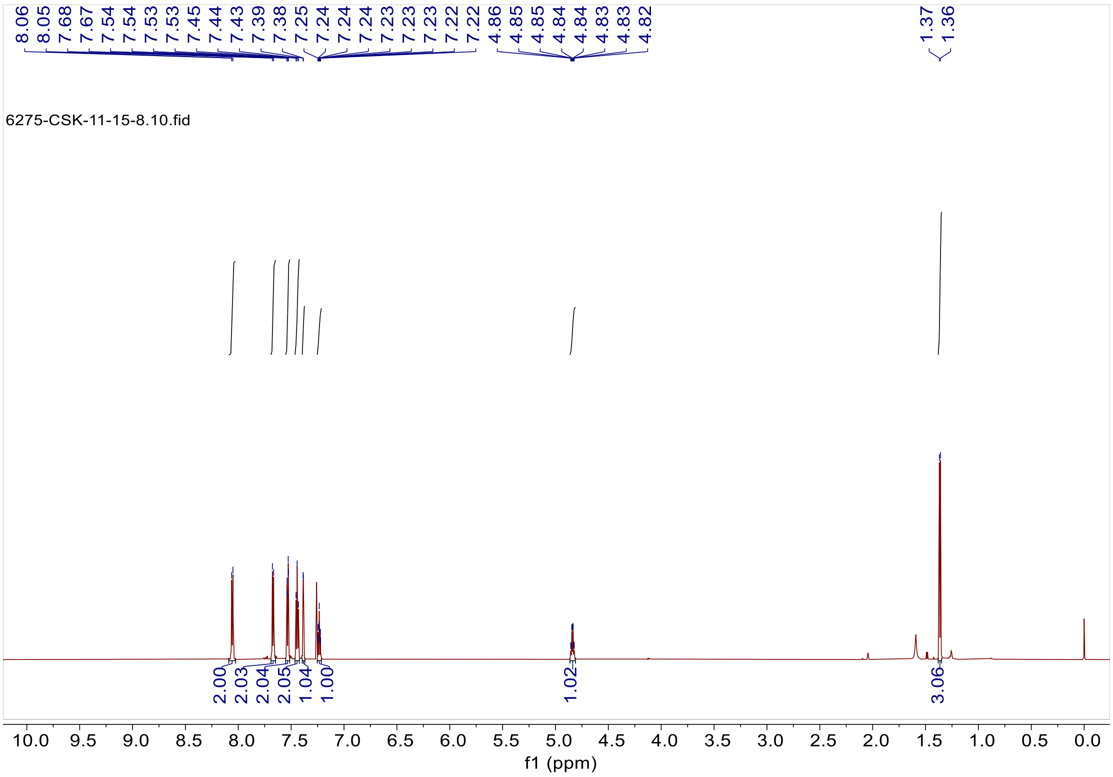

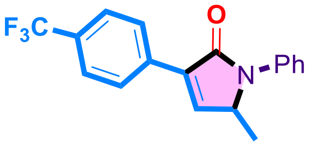

7v

^{1}H NMR (700 MHz, $CDCl_3$)
^{13}C NMR (176 MHz, $CDCl_3$)

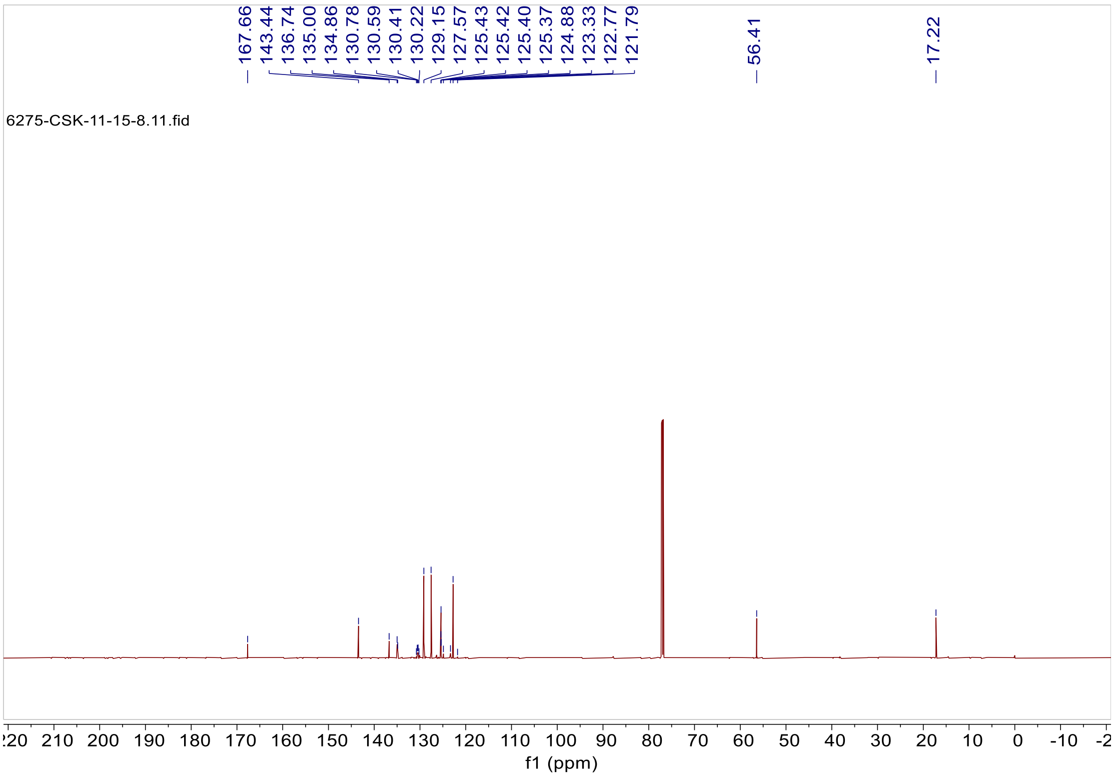

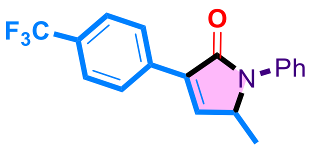

7v ^{19}F NMR (376 MHz, $CDCl_3$)

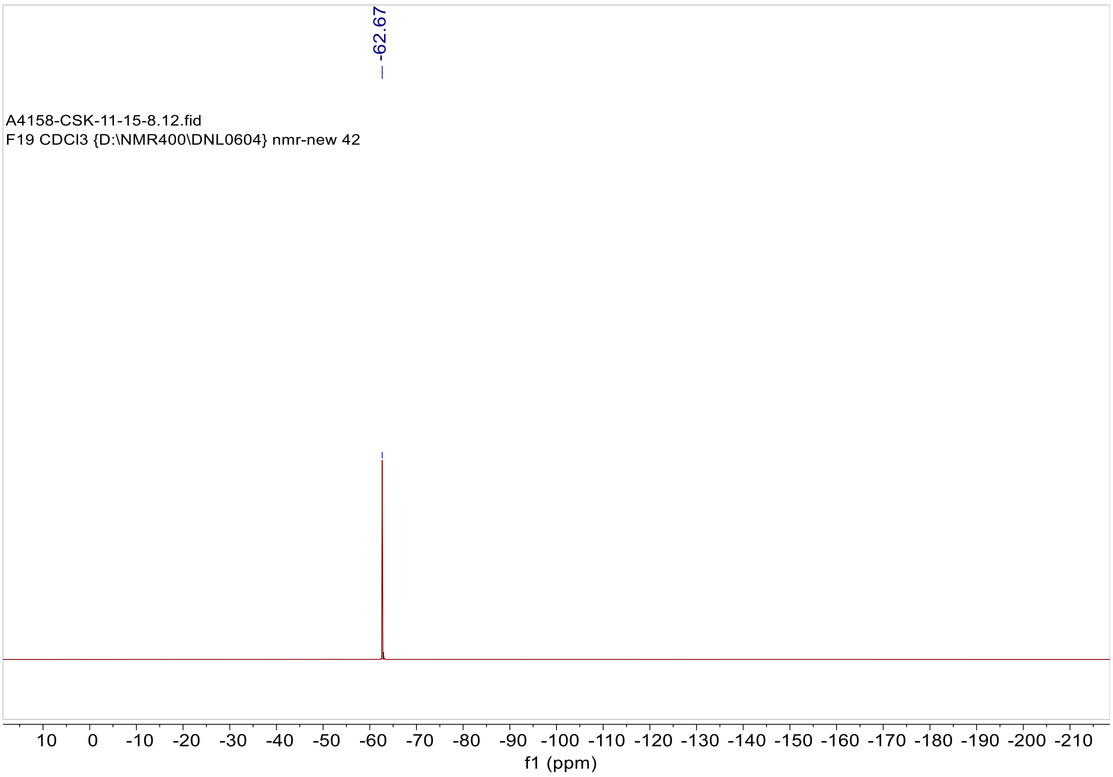

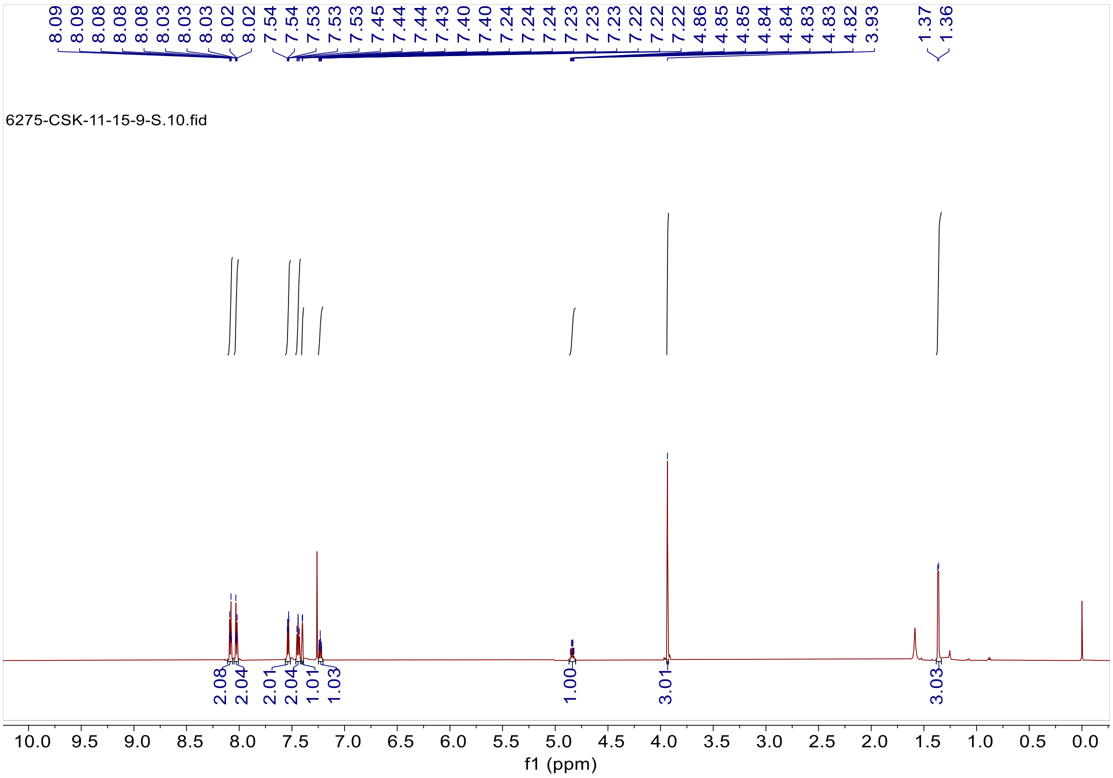

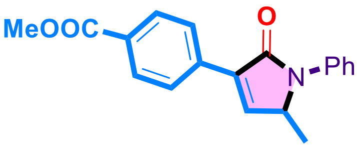

7w

^{1}H NMR (700 MHz, $CDCl_3$)
^{13}C NMR (176 MHz, $CDCl_3$)

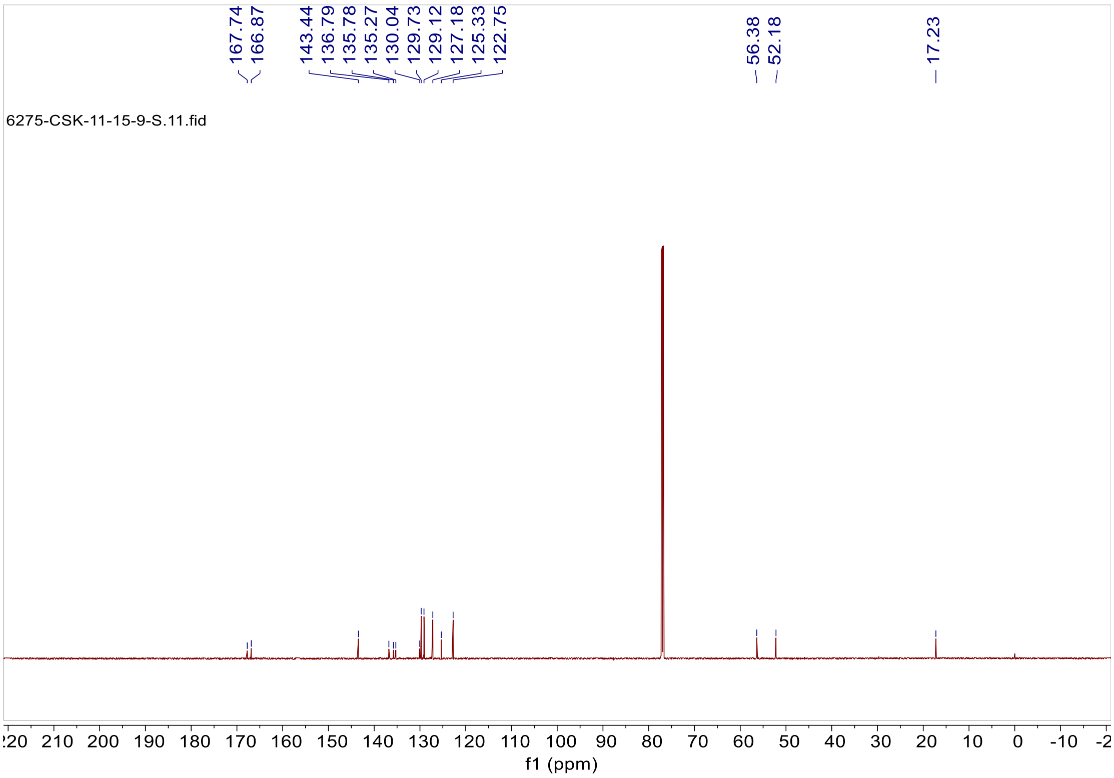

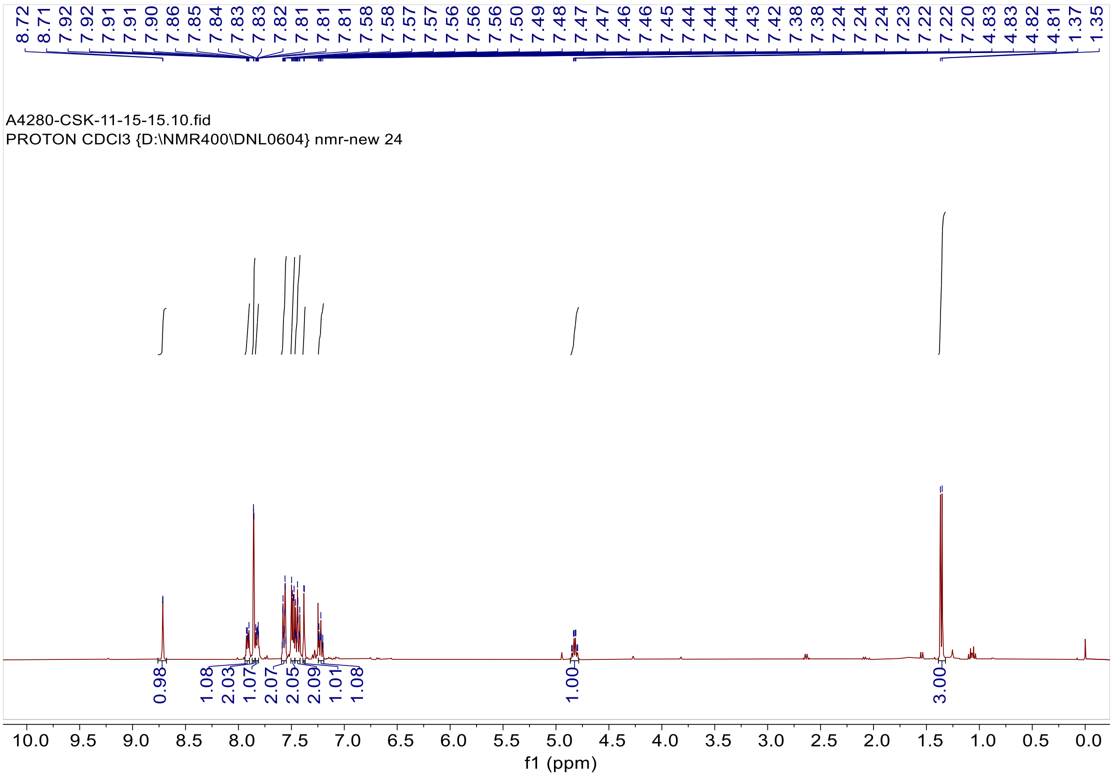

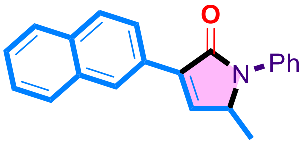

7x

^{1}H NMR (400 MHz, $CDCl_3$)

^{13}C NMR (100 MHz, $CDCl_3$)

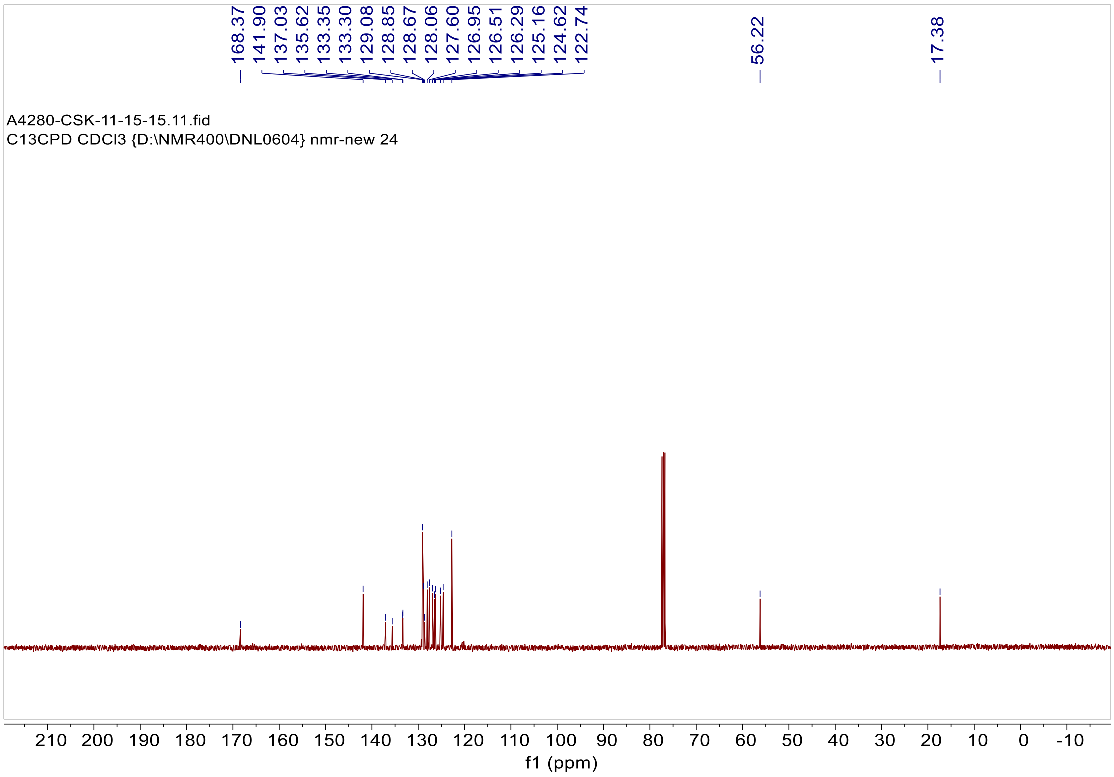

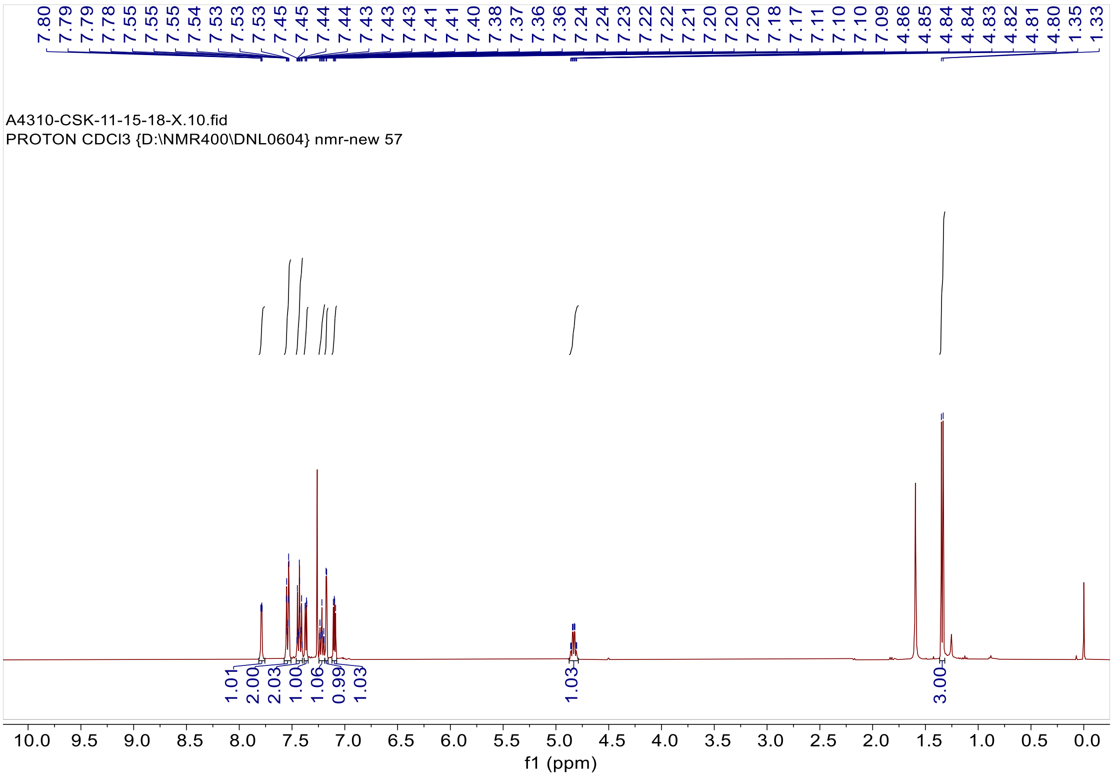

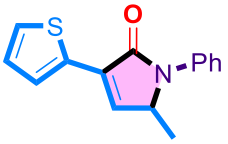

7z

^{1}H NMR (400 MHz, $CDCl_3$)
^{13}C NMR (100 MHz, $CDCl_3$)

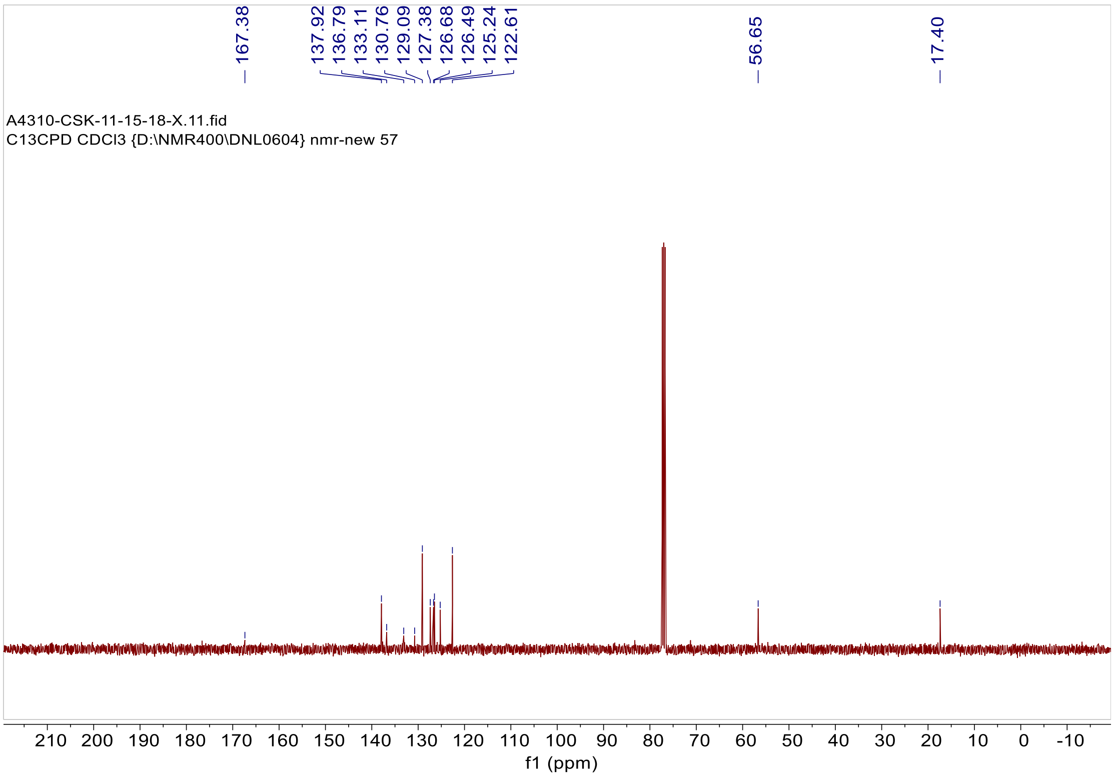

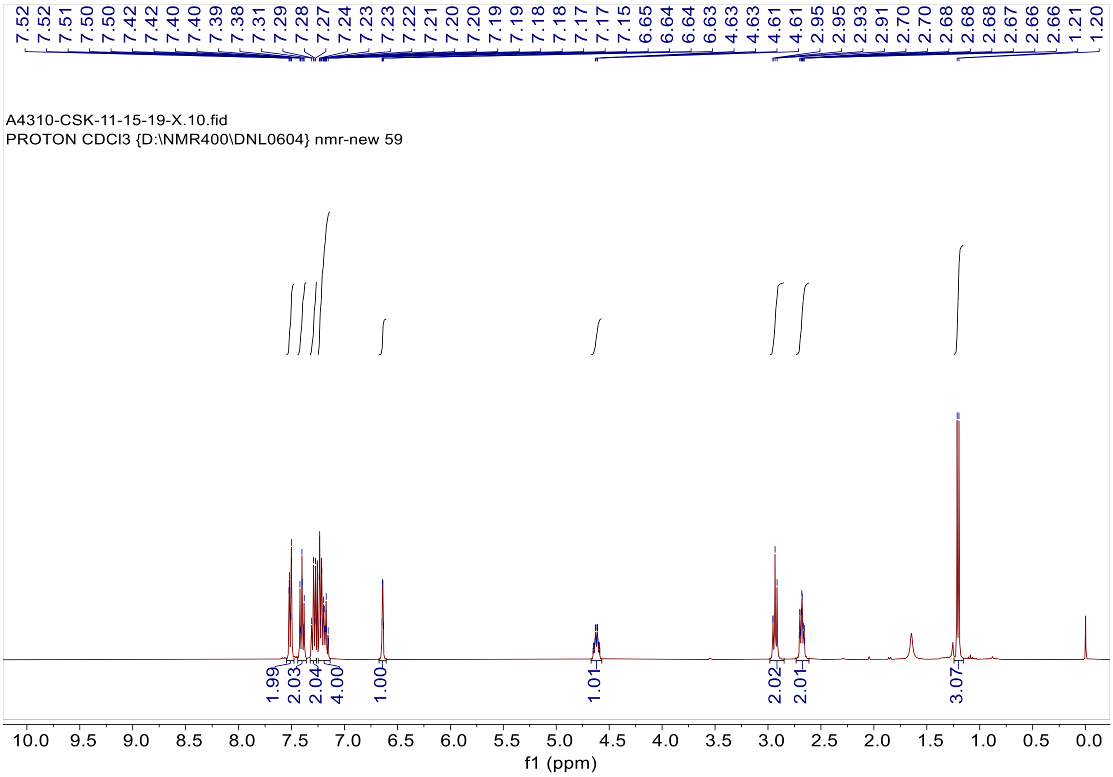

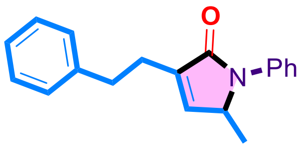

7aa

^{1}H NMR (400 MHz, $CDCl_3$)
^{13}C NMR (100 MHz, $CDCl_3$)

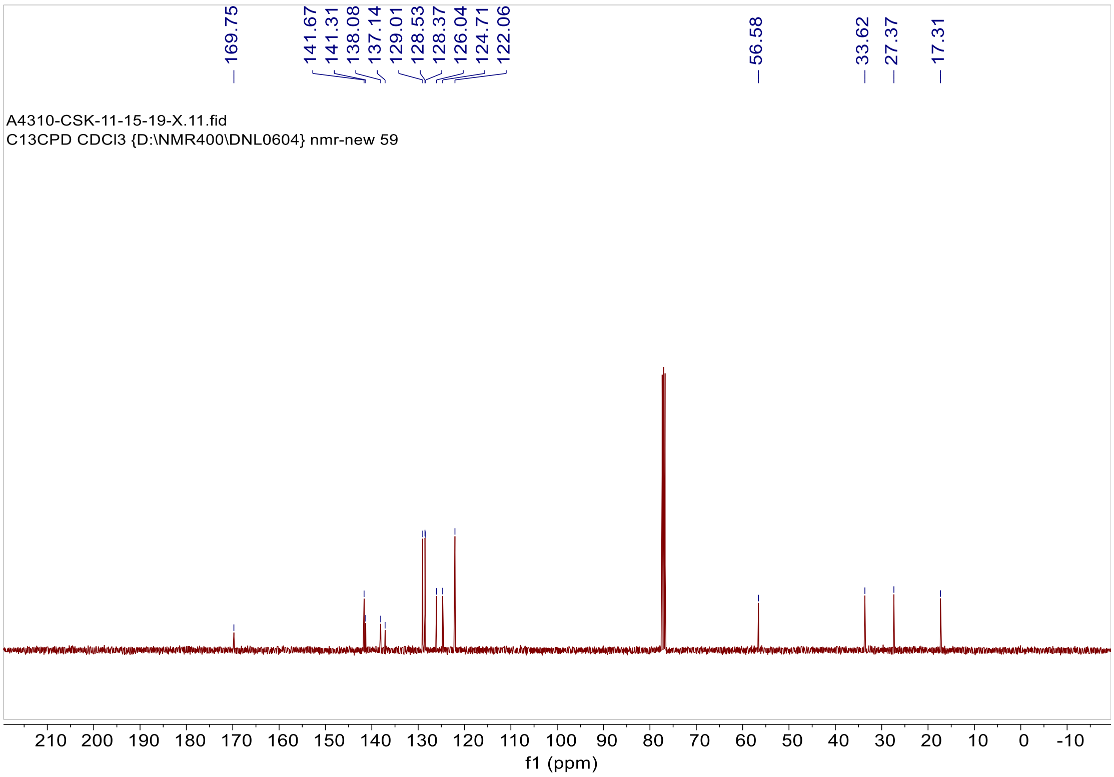

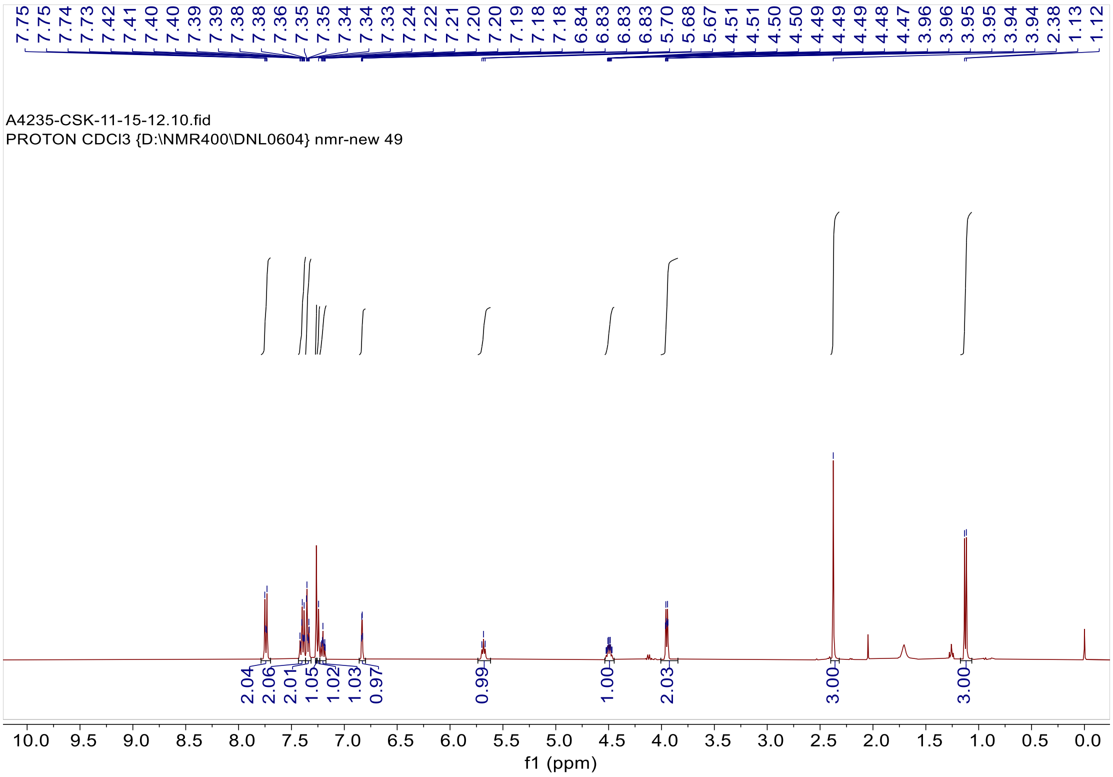

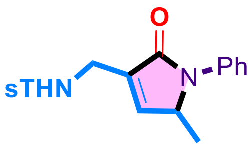

7ab

^{1}H NMR (400 MHz, $CDCl_3$)
^{13}C NMR (100 MHz, $CDCl_3$)

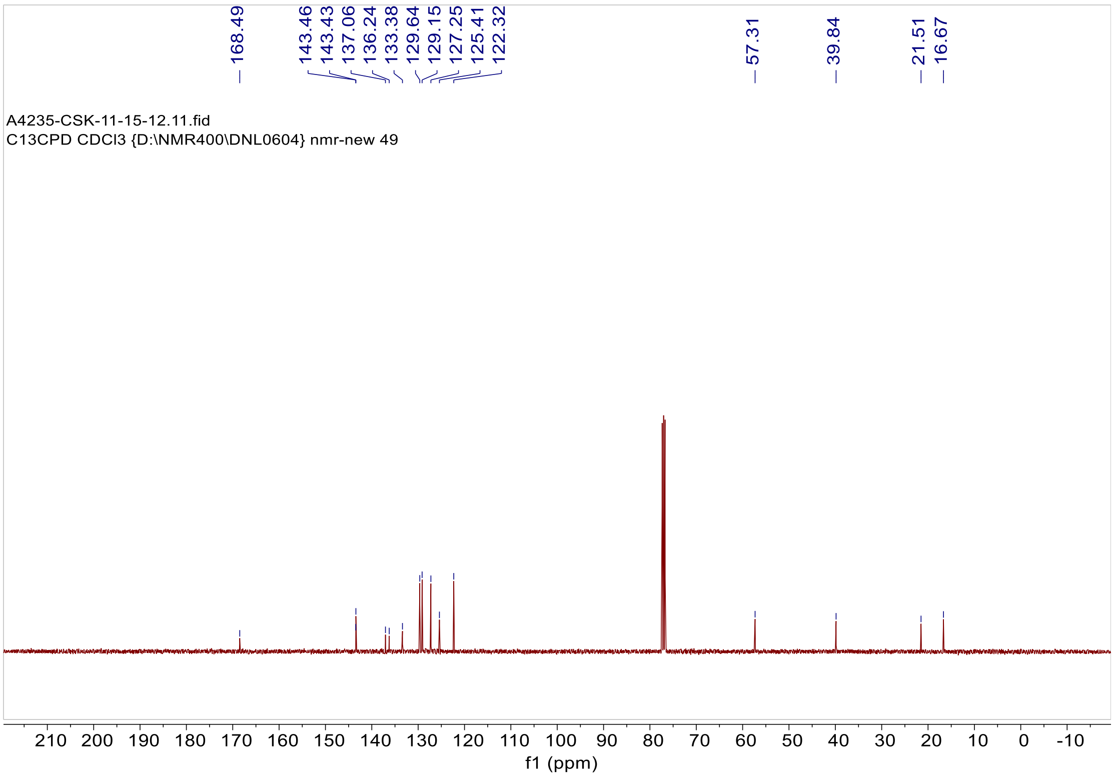

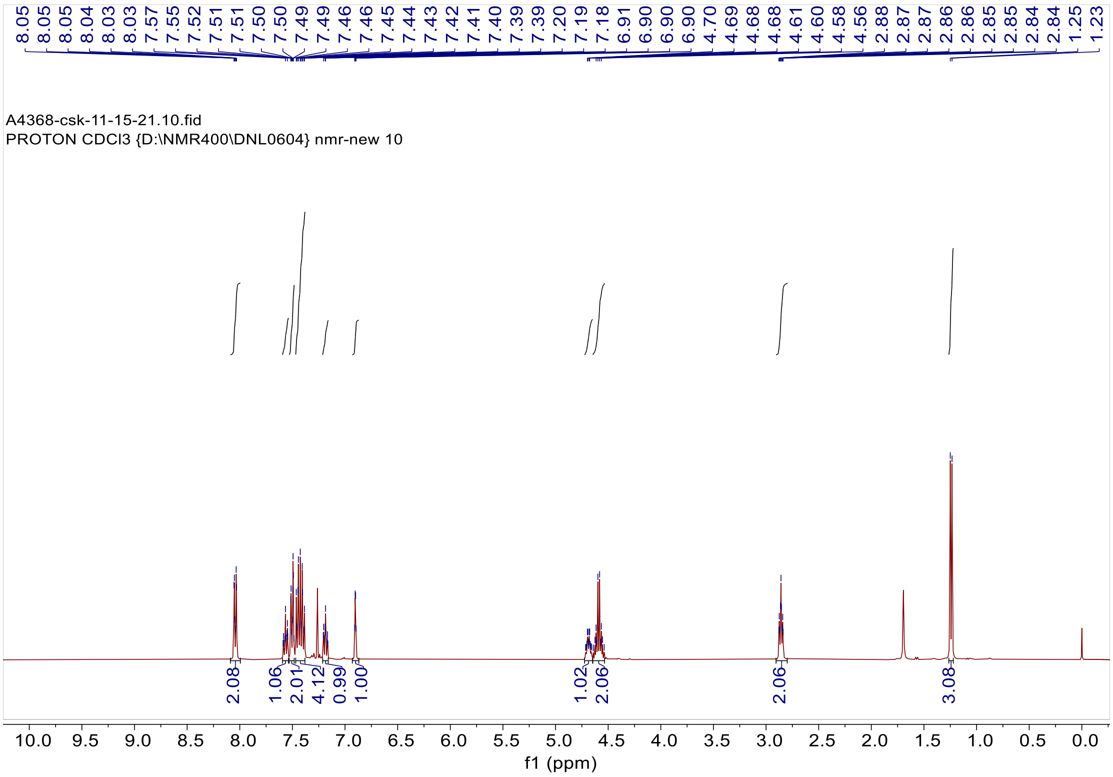

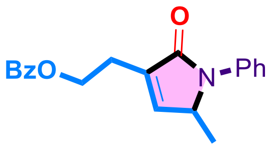

7ac

^{1}H NMR (400 MHz, $CDCl_3$)

^{13}C NMR (100 MHz, $CDCl_3$)

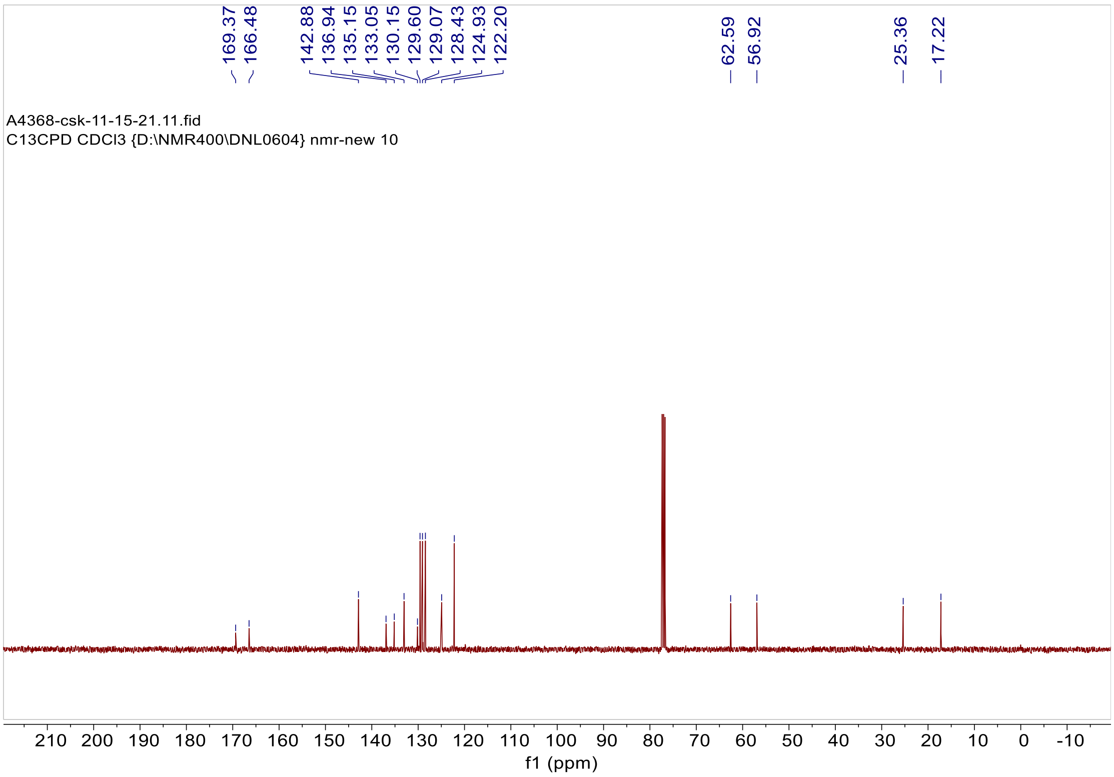

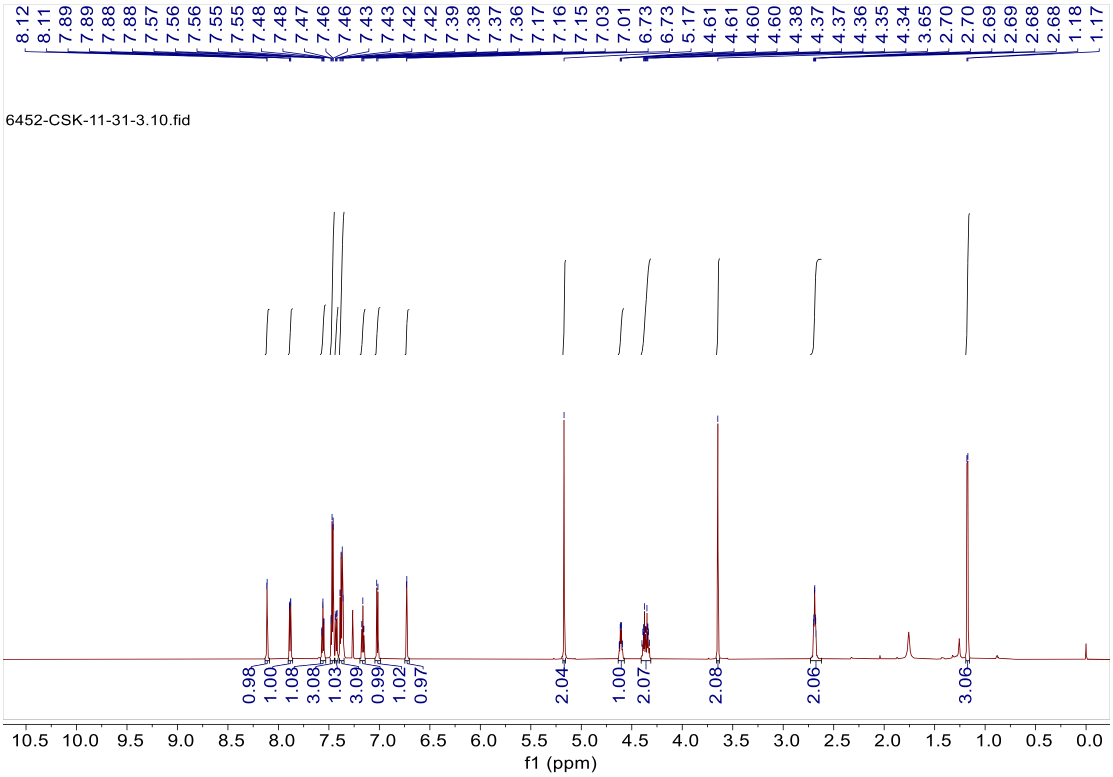

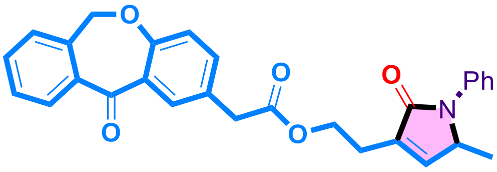

7ad

^{1}H NMR (700 MHz, $CDCl_3$)
^{13}C NMR (176 MHz, $CDCl_3$)

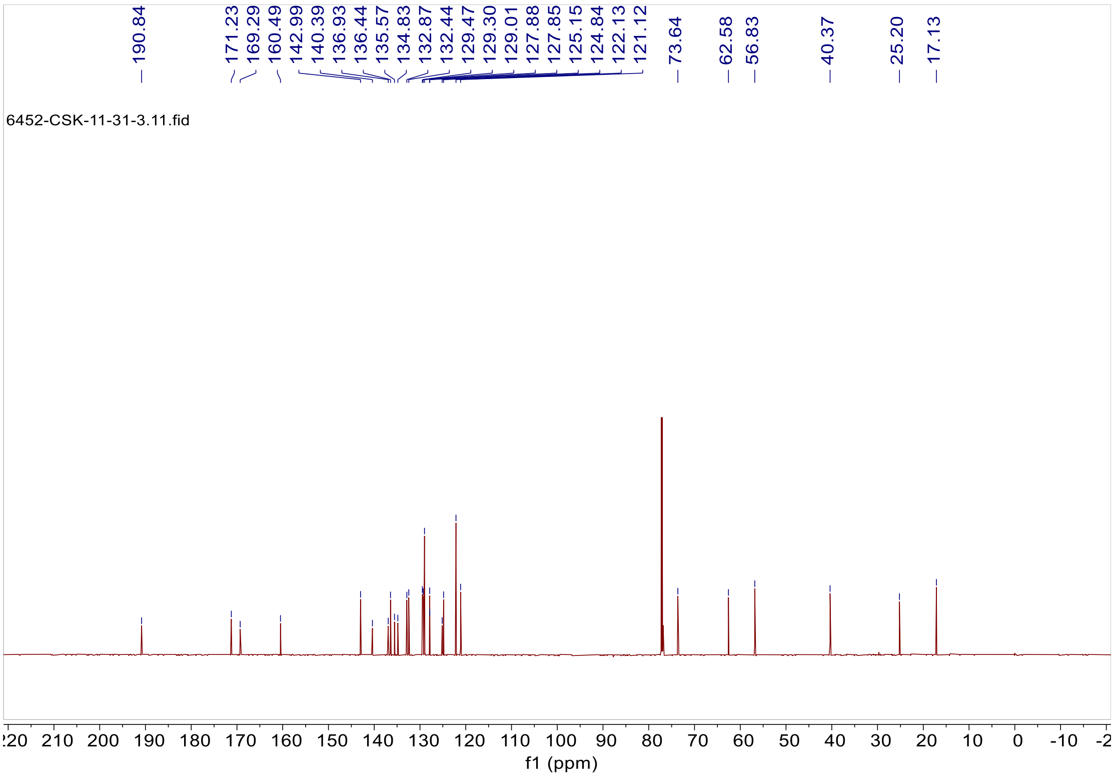

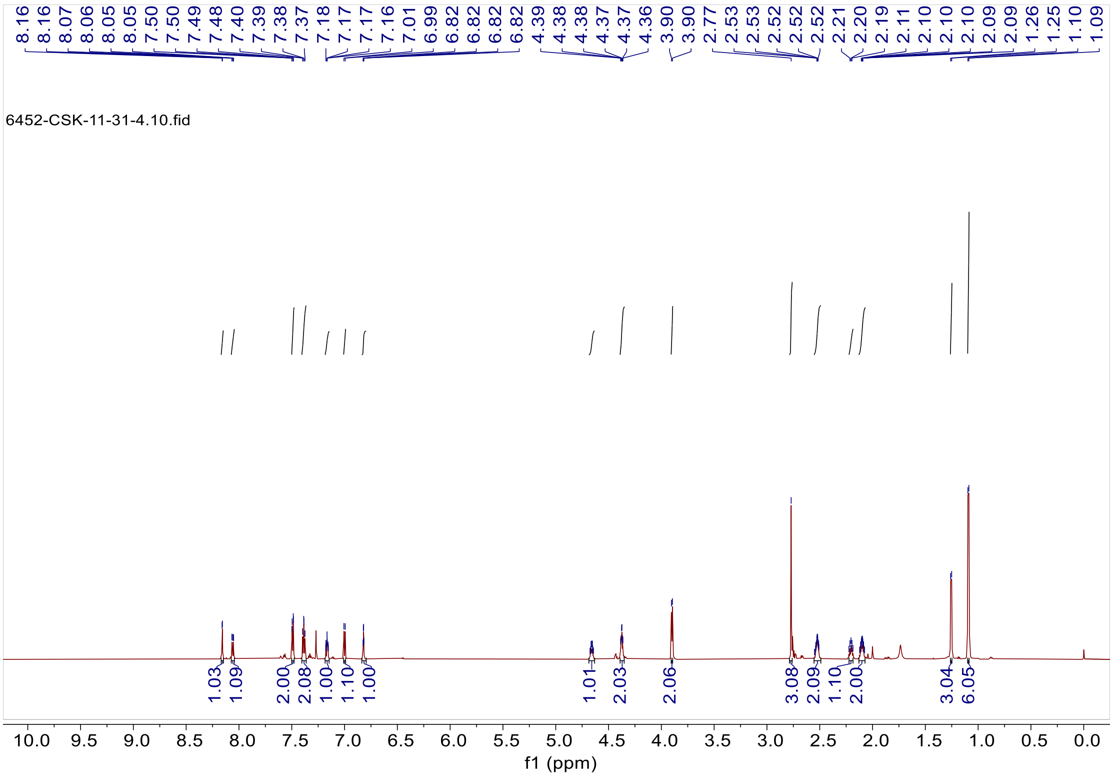

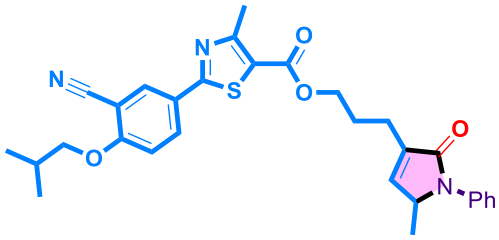

7ae

^{1}H NMR (700 MHz, $CDCl_3$)

^{13}C NMR (176 MHz, $CDCl_3$)

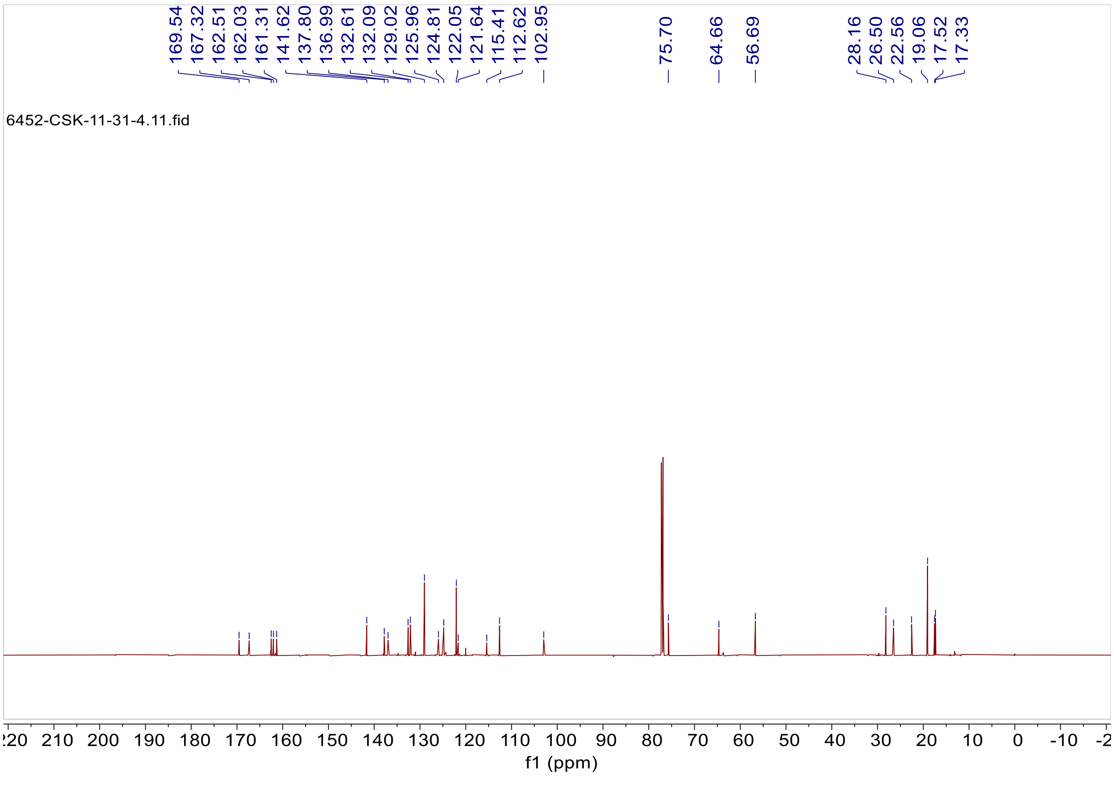

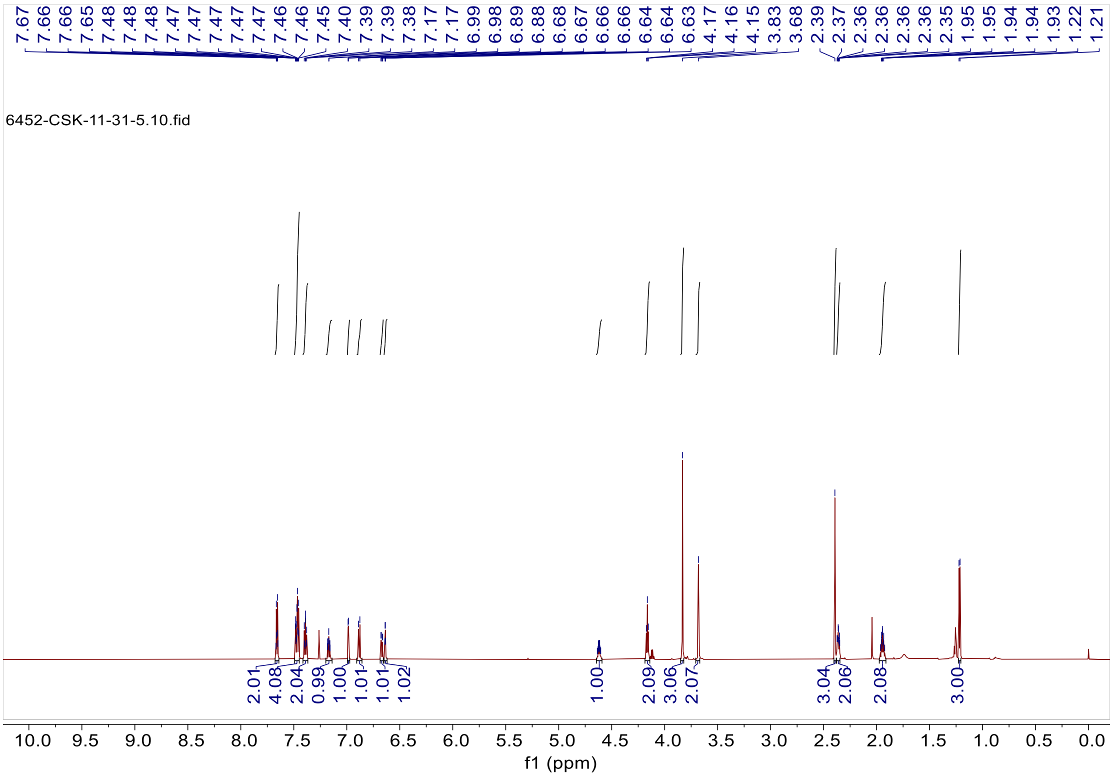

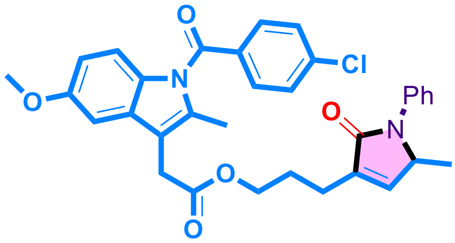

7af

^{1}H NMR (700 MHz, $CDCl_3$)

^{13}C NMR (176 MHz, $CDCl_3$)

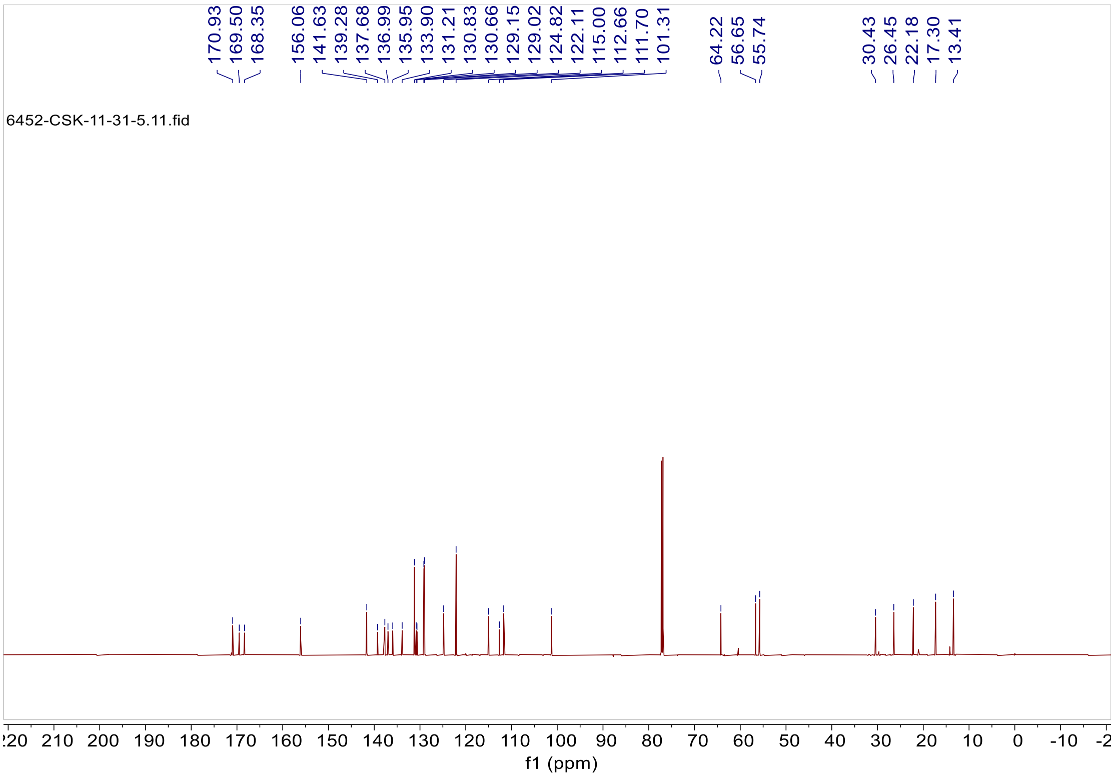

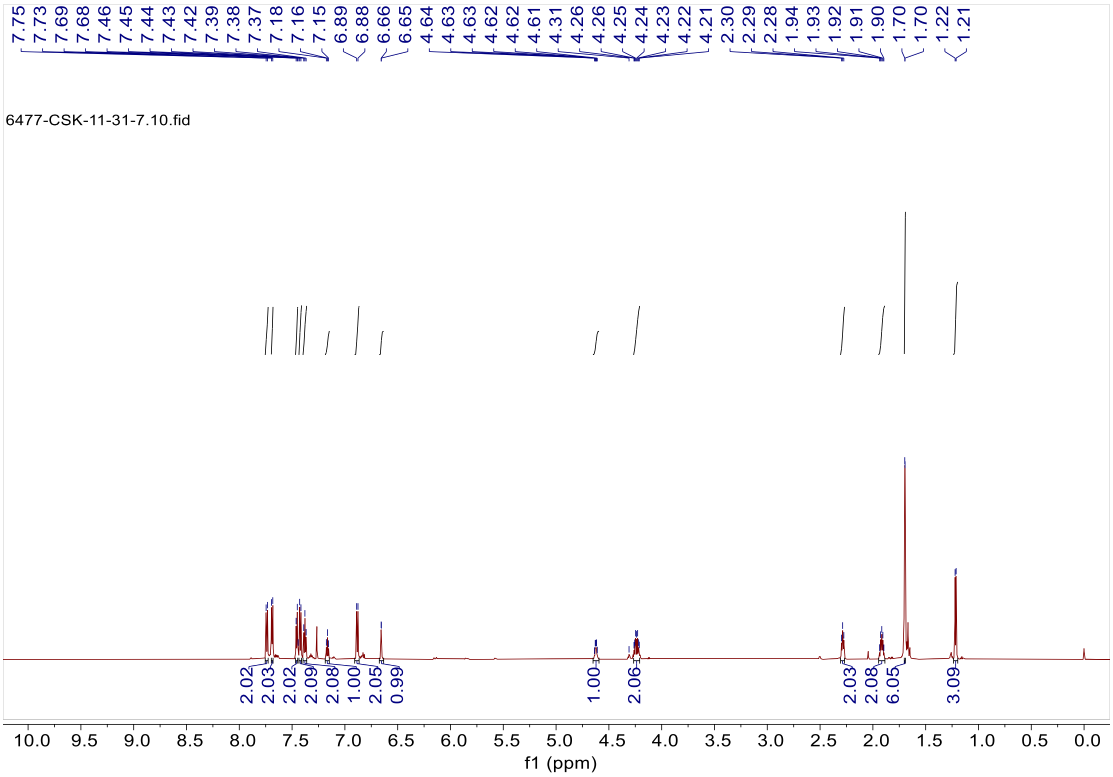

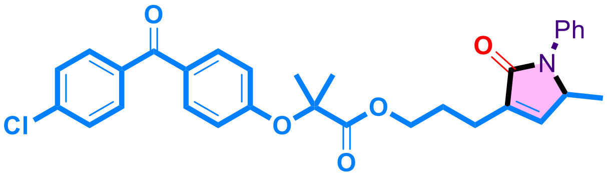

7ag

^{1}H NMR (700 MHz, $CDCl_3$)
^{13}C NMR (176 MHz, $CDCl_3$)

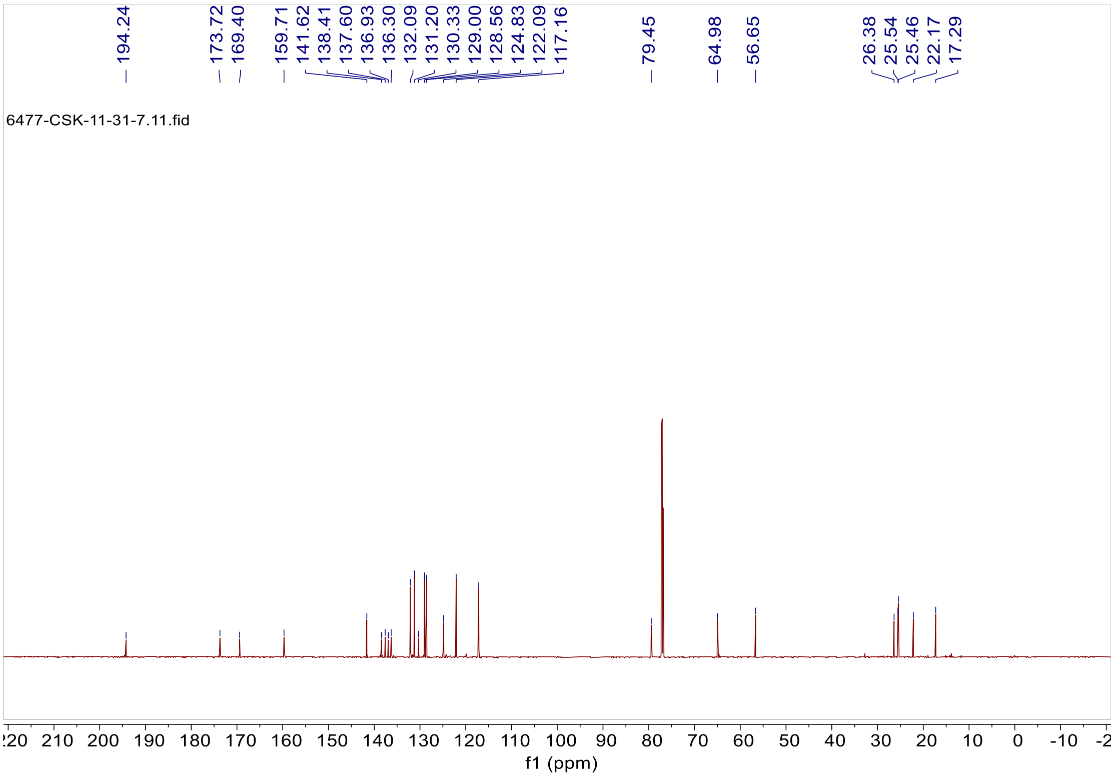

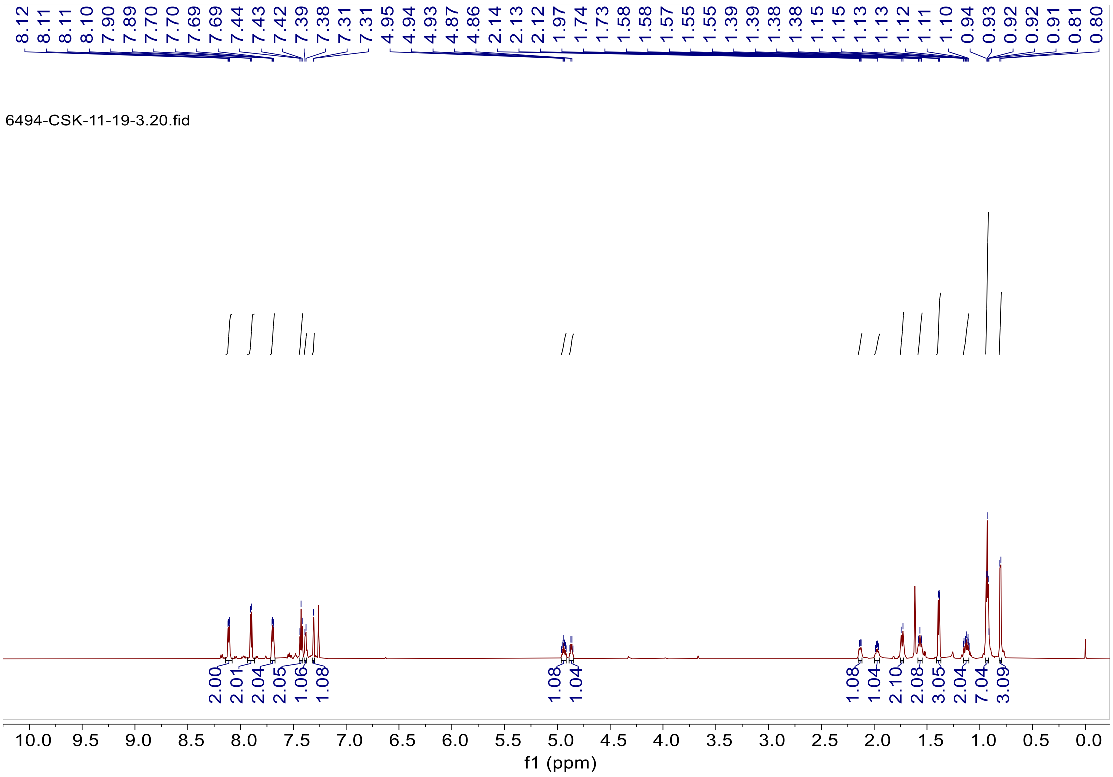

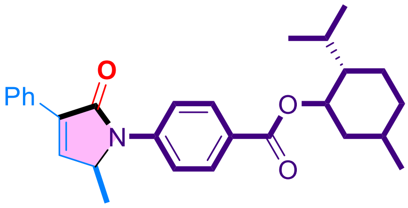

7aj

^{1}H NMR (700 MHz, $CDCl_3$)
^{13}C NMR (176 MHz, $CDCl_3$)

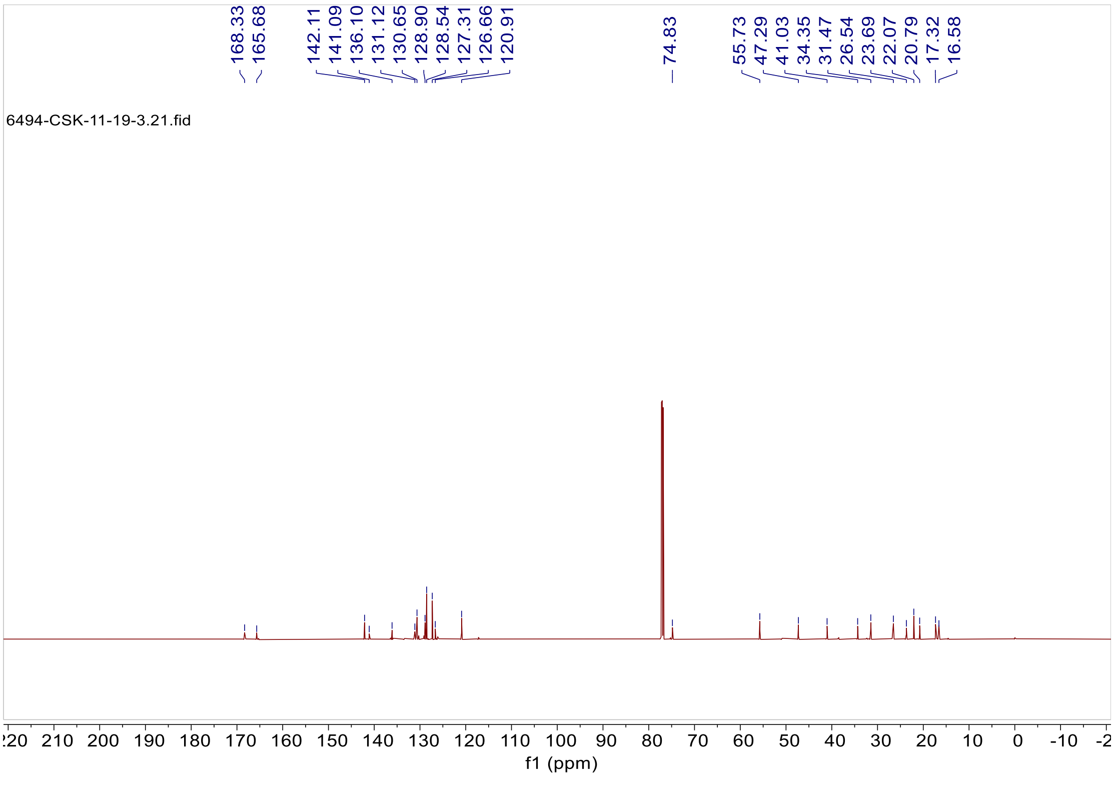

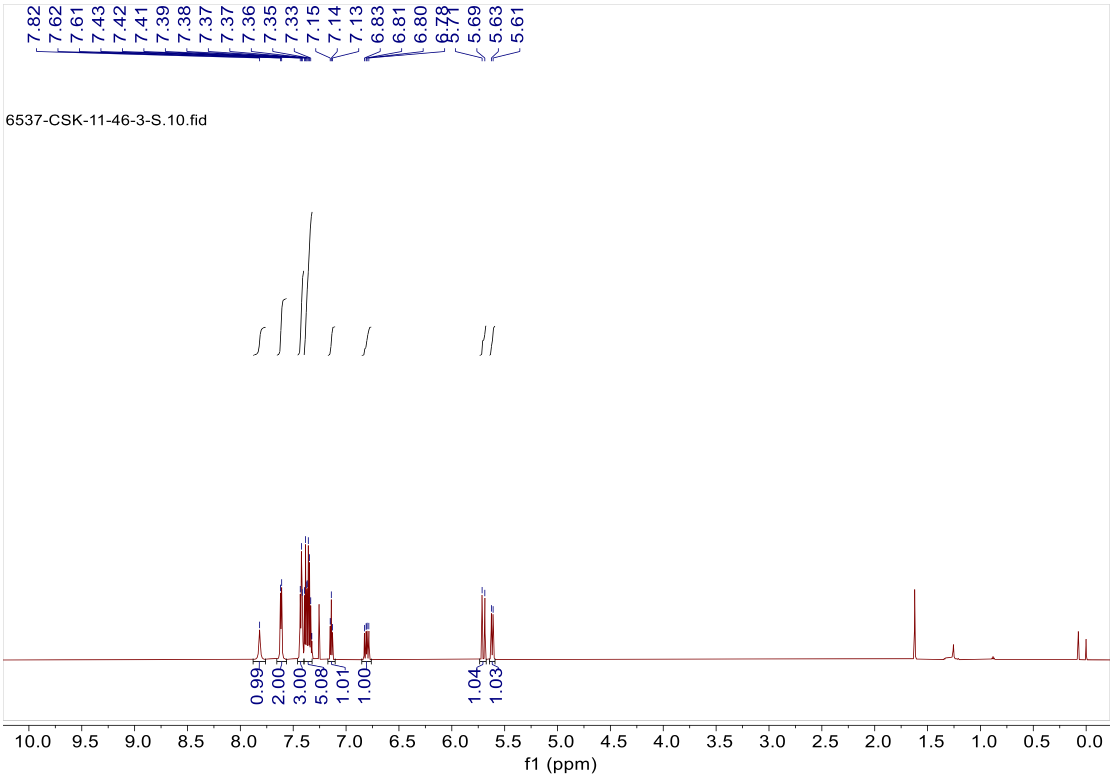

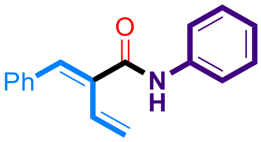

3a

^{1}H NMR (700 MHz, $CDCl_3$)

^{13}C NMR (176 MHz, $CDCl_3$)

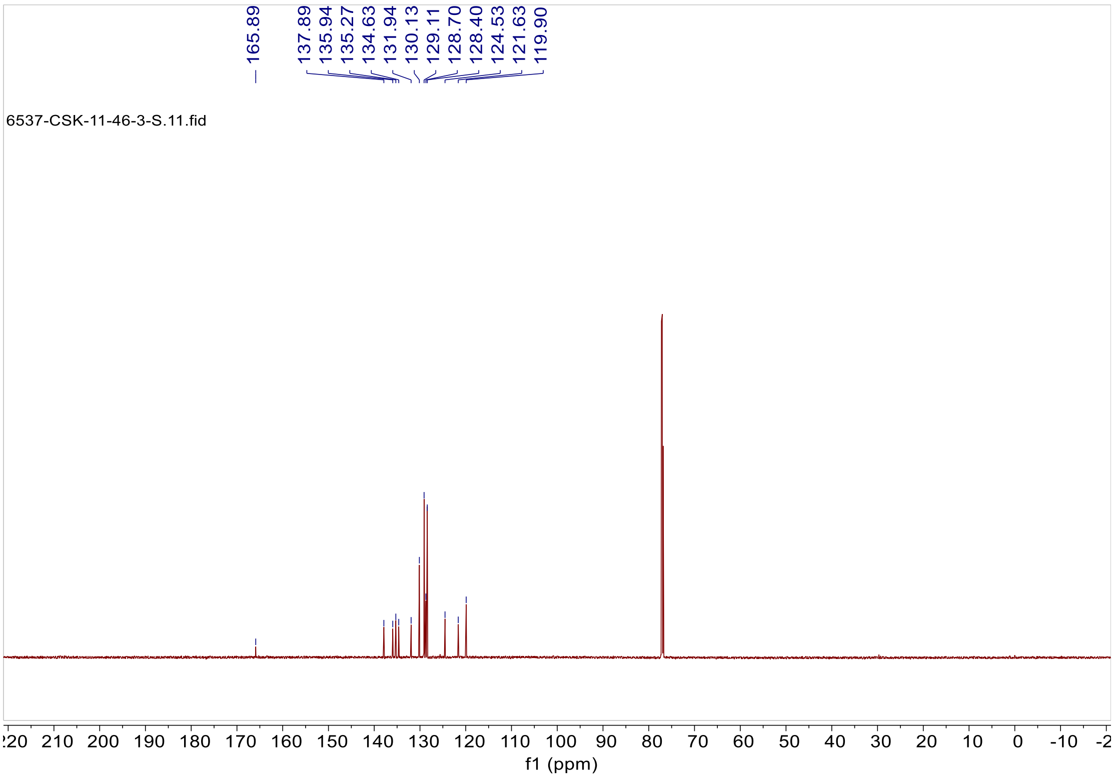

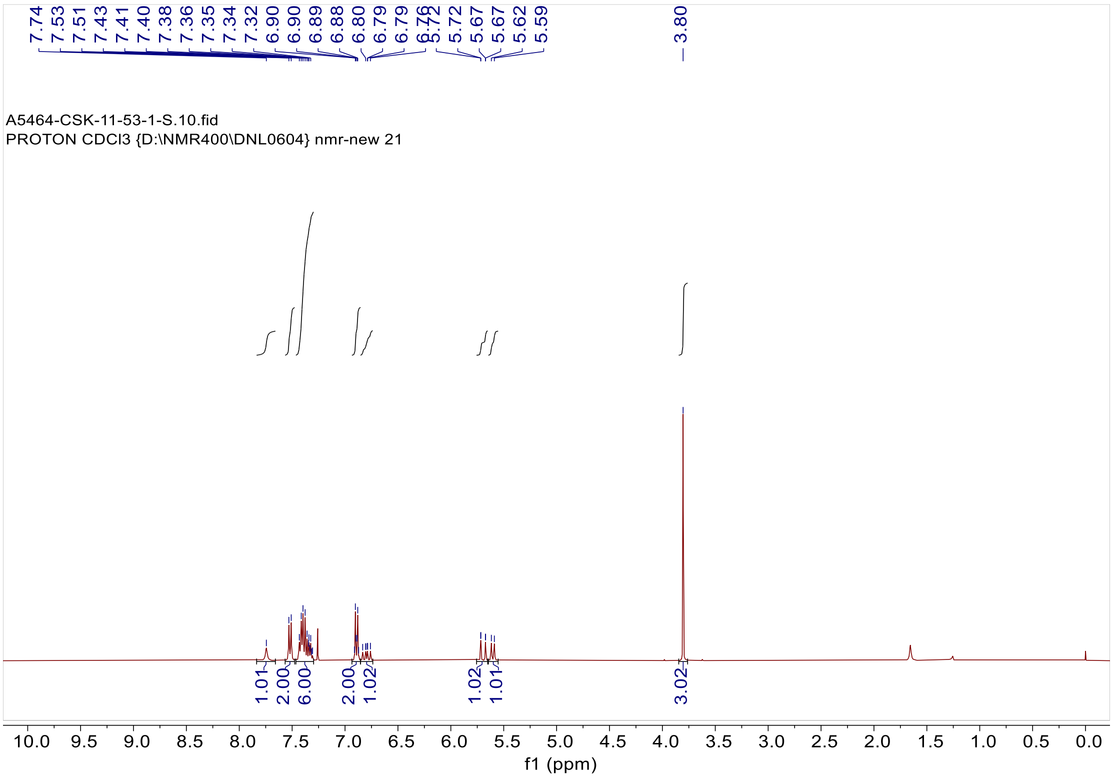

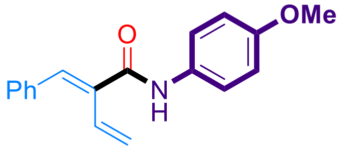

3b

^{1}H NMR (400 MHz, $CDCl_3$)
^{13}C NMR (100 MHz, $CDCl_3$)

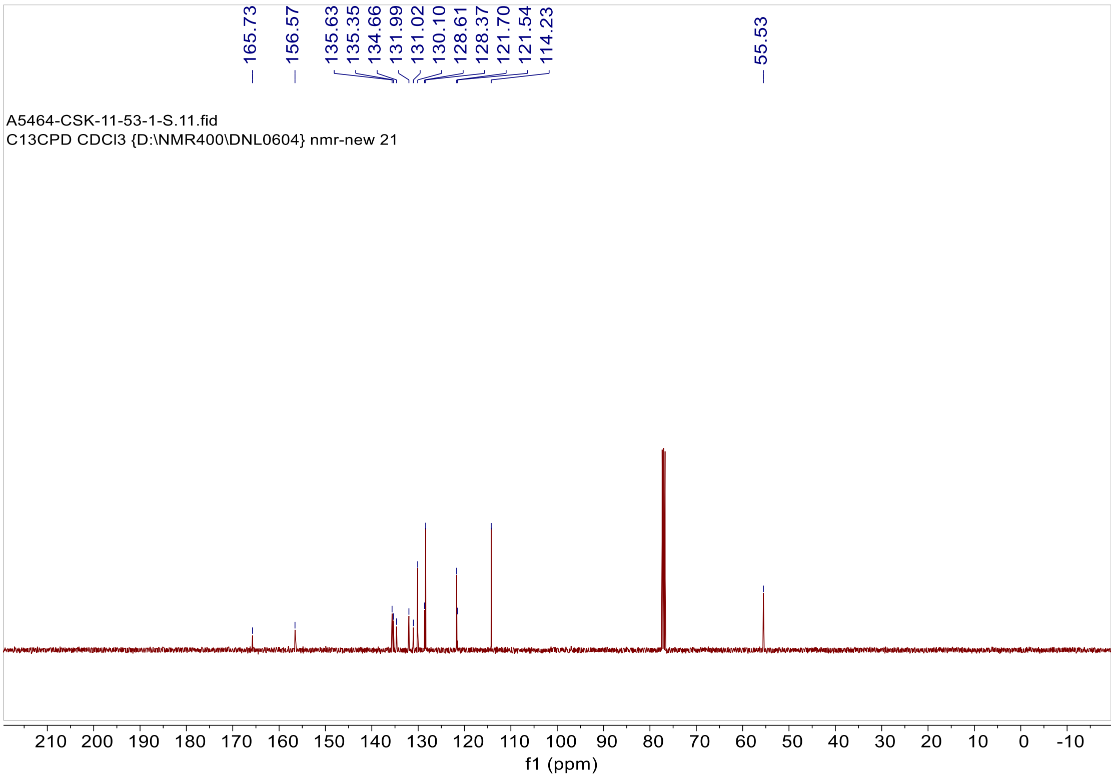

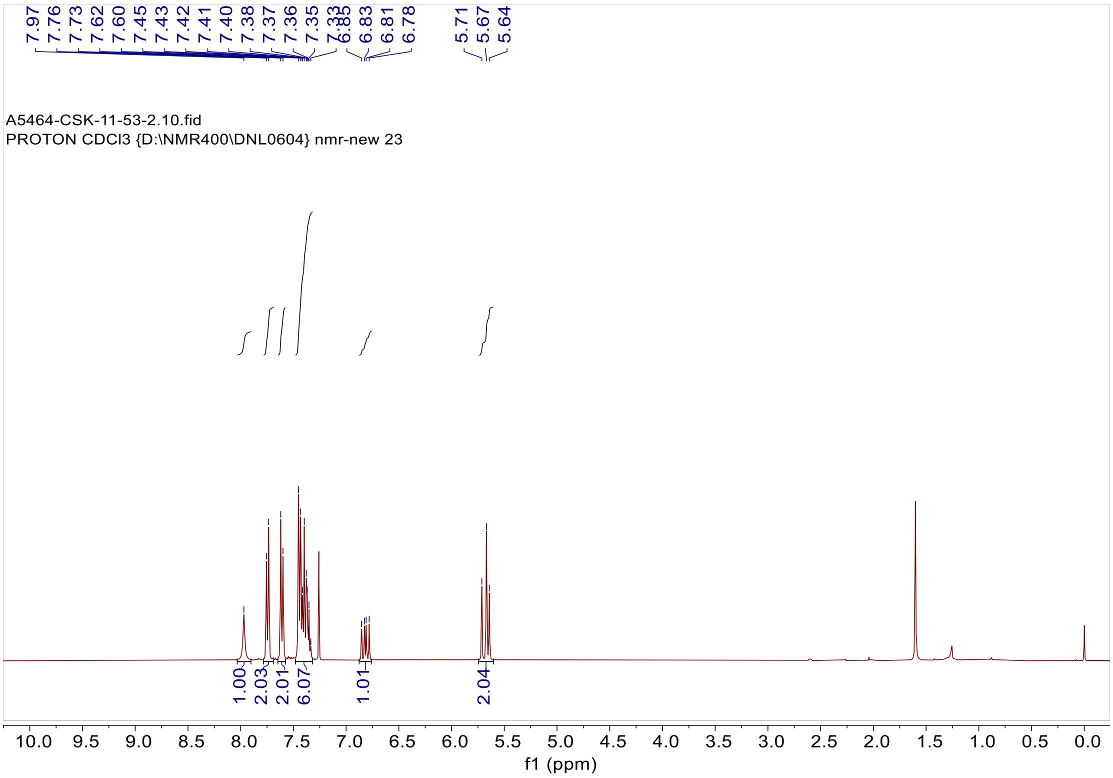

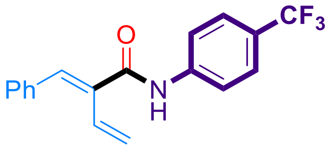

3c

^{1}H NMR (400 MHz, $CDCl_3$)
^{13}C NMR (100 MHz, $CDCl_3$)

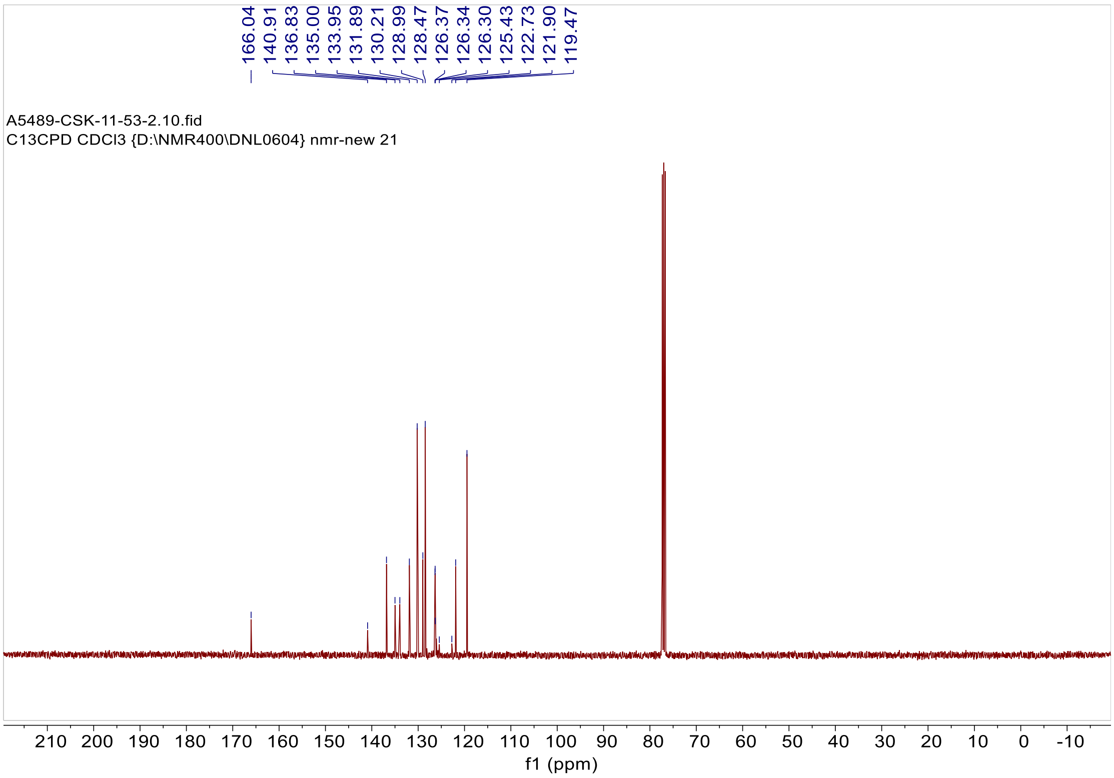

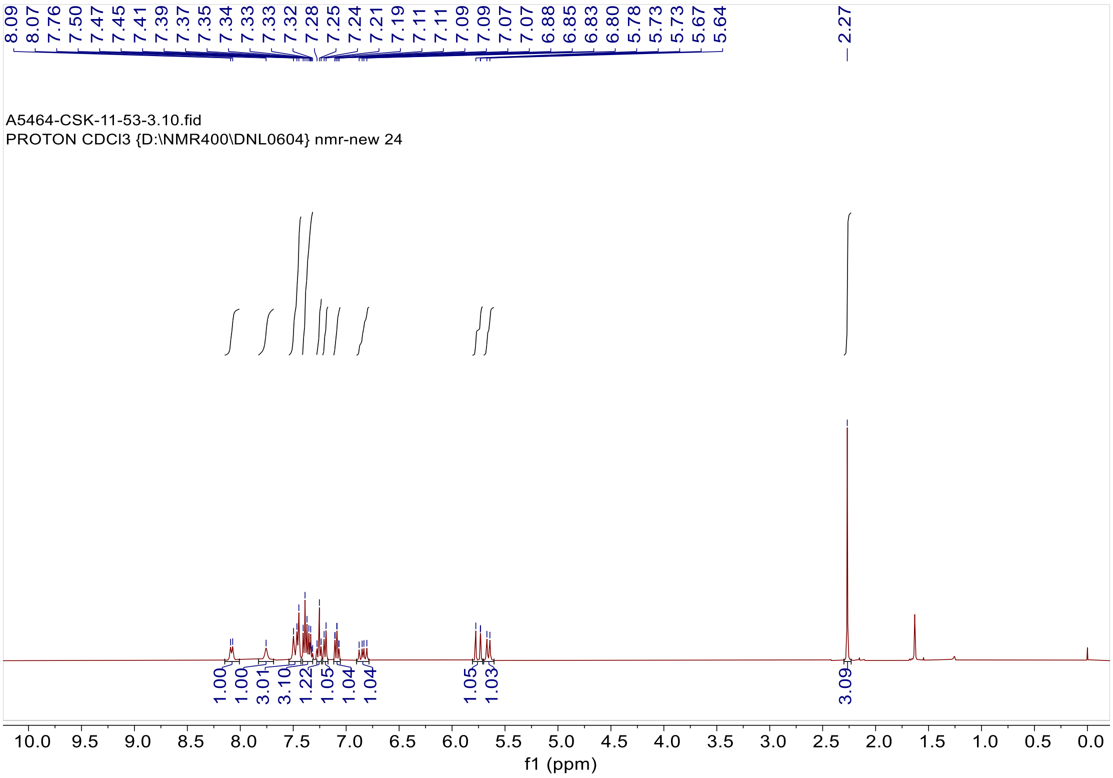

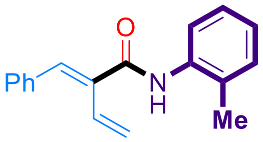

3d

^{1}H NMR (400 MHz, $CDCl_3$)
^{13}C NMR (100 MHz, $CDCl_3$)

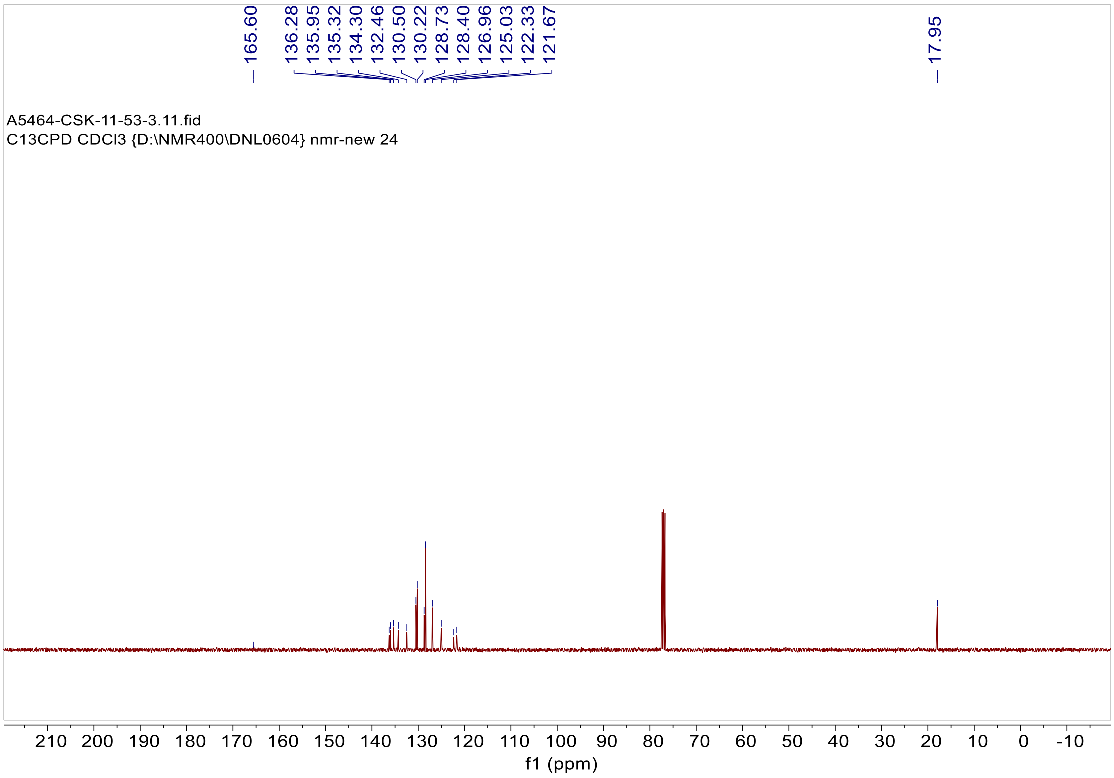

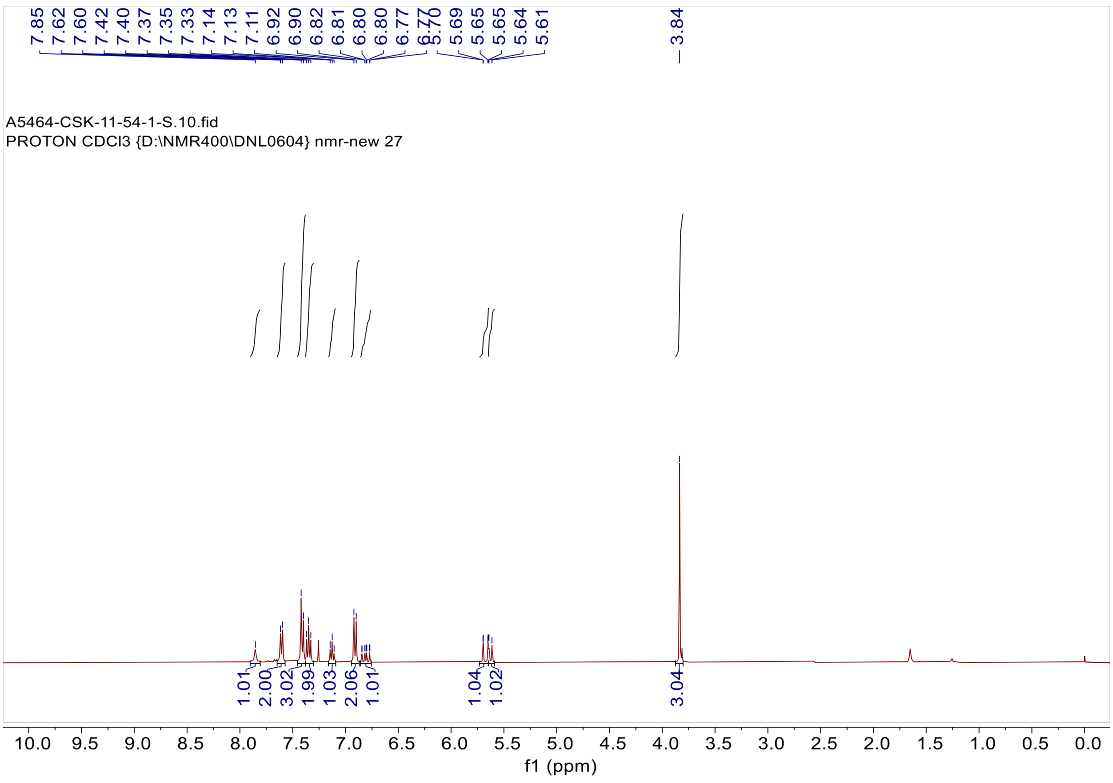

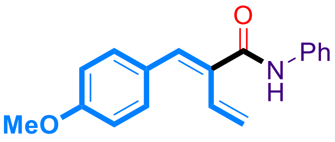

3e

^{1}H NMR (400 MHz, $CDCl_3$)
^{13}C NMR (100 MHz, $CDCl_3$)

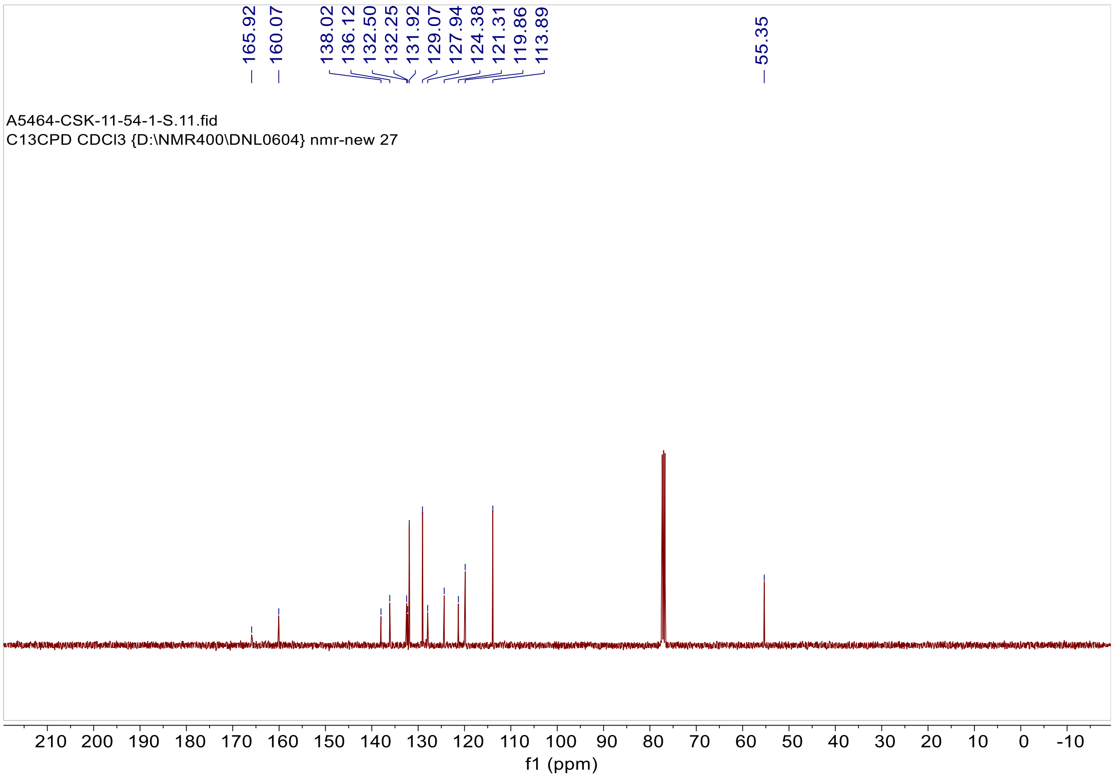

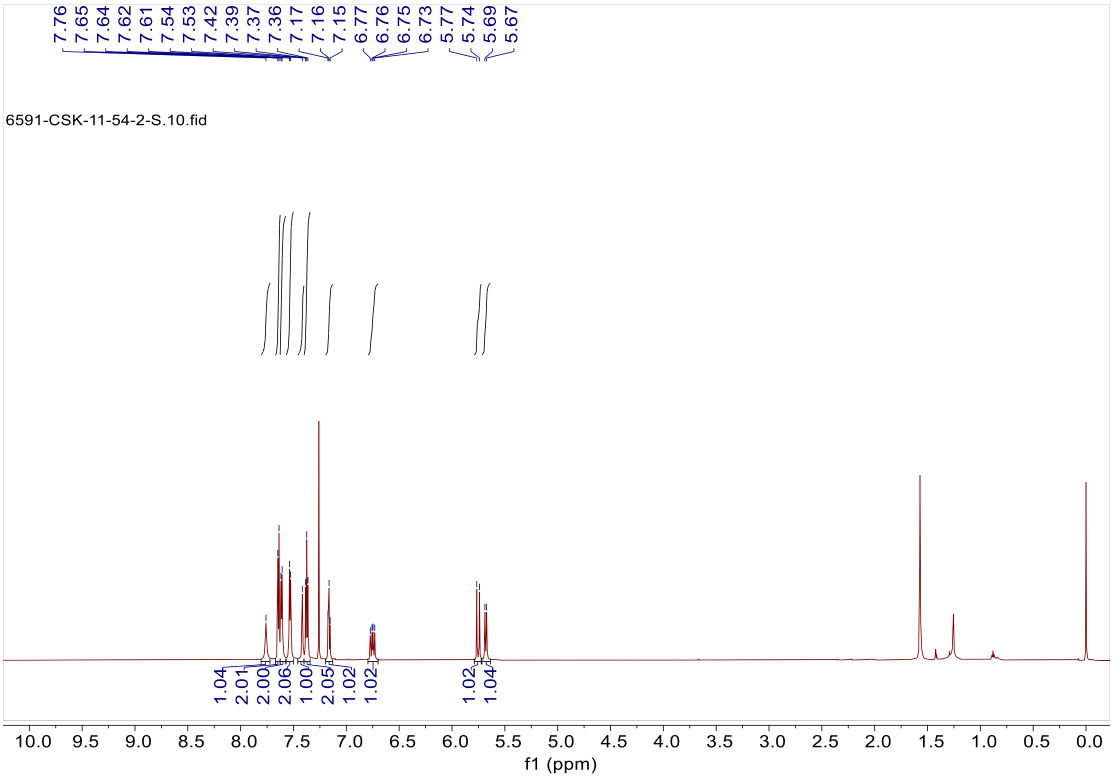

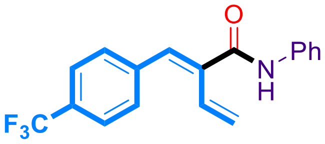

3f

^{1}H NMR (700 MHz, $CDCl_3$)

^{13}C NMR (176 MHz, $CDCl_3$)

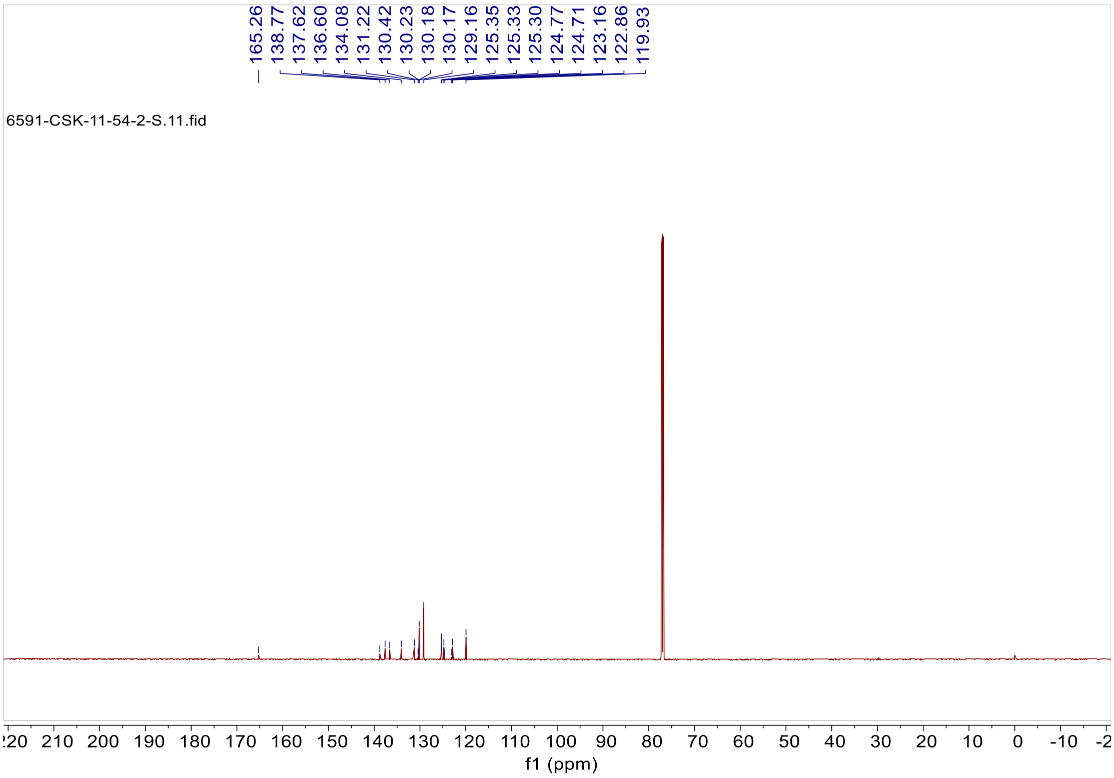

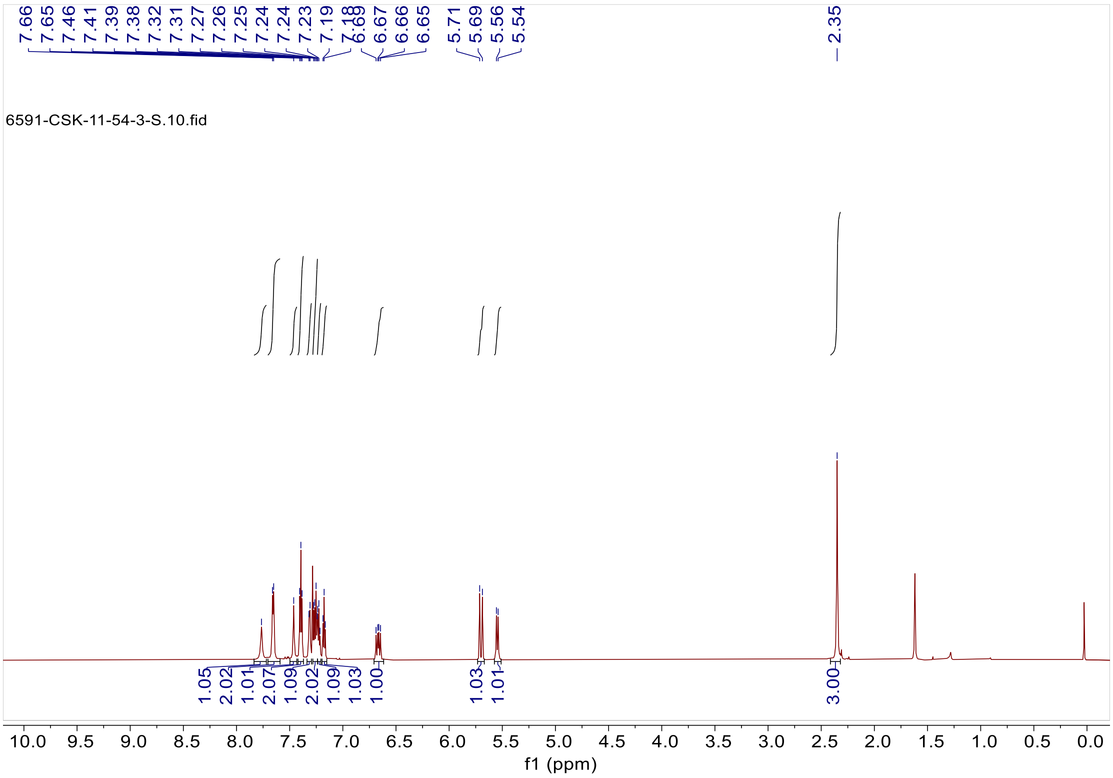

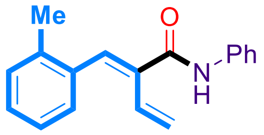

3g

^{1}H NMR (700 MHz, $CDCl_3$)
^{13}C NMR (176 MHz, $CDCl_3$)

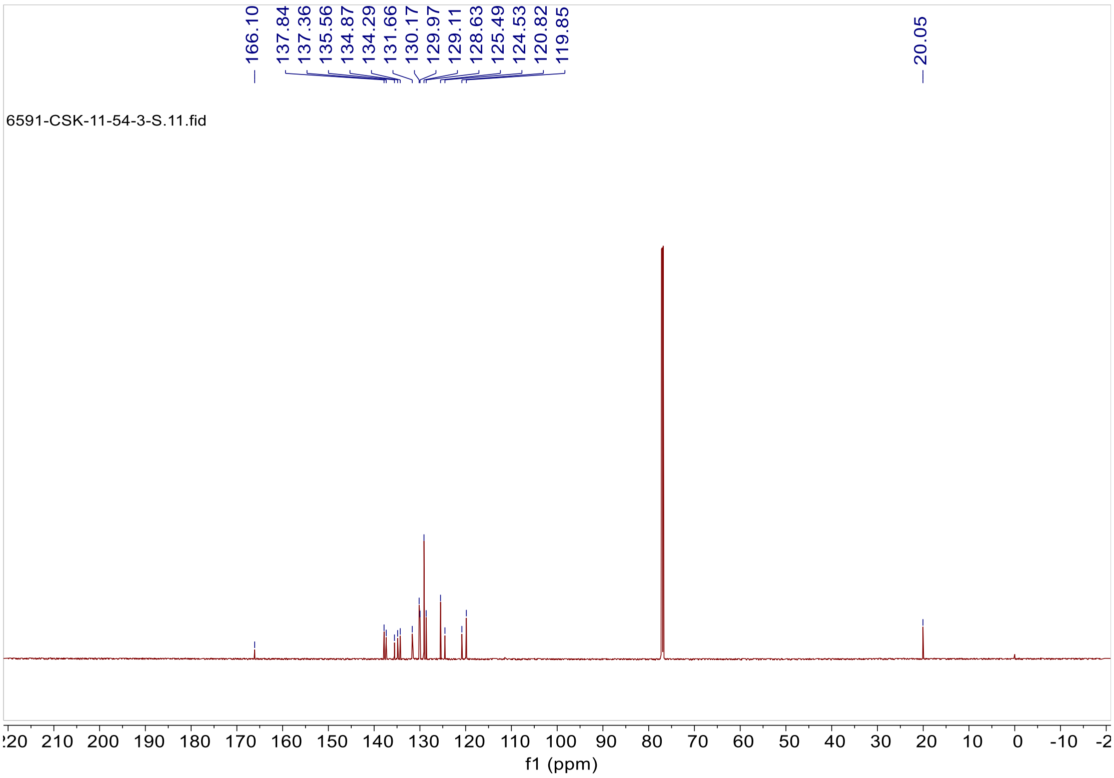

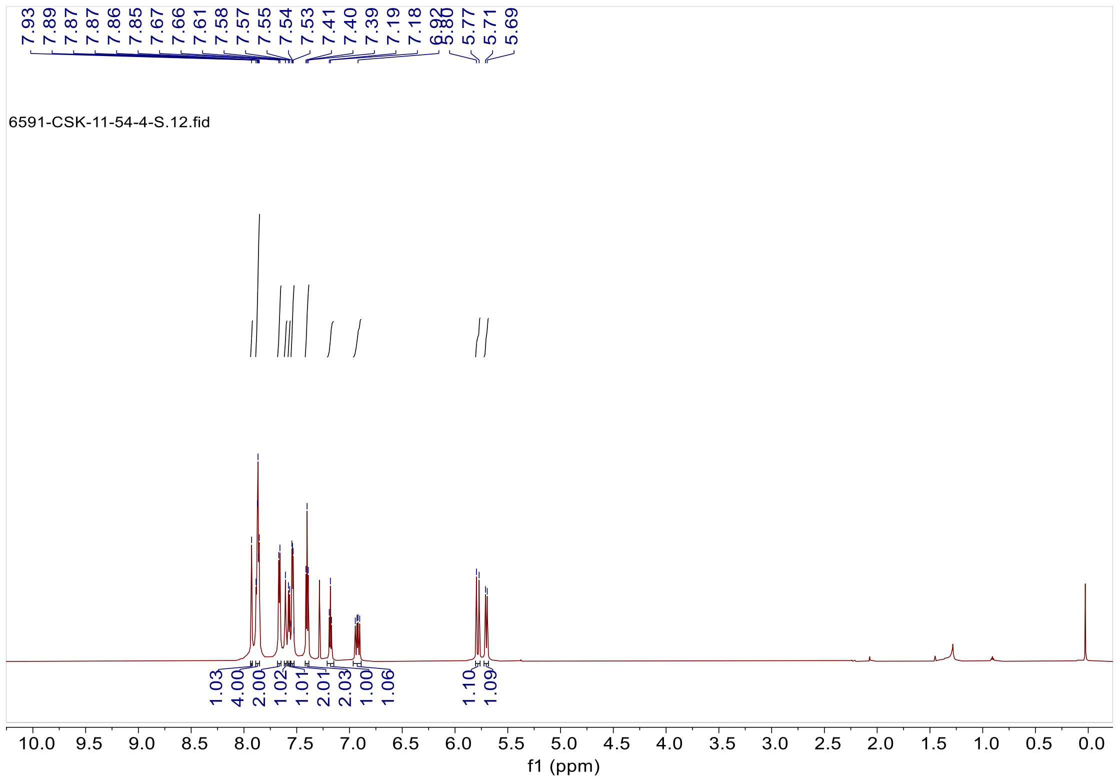

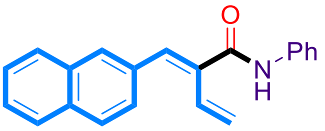

3h

^{1}H NMR (700 MHz, $CDCl_3$)
^{13}C NMR (176 MHz, $CDCl_3$)

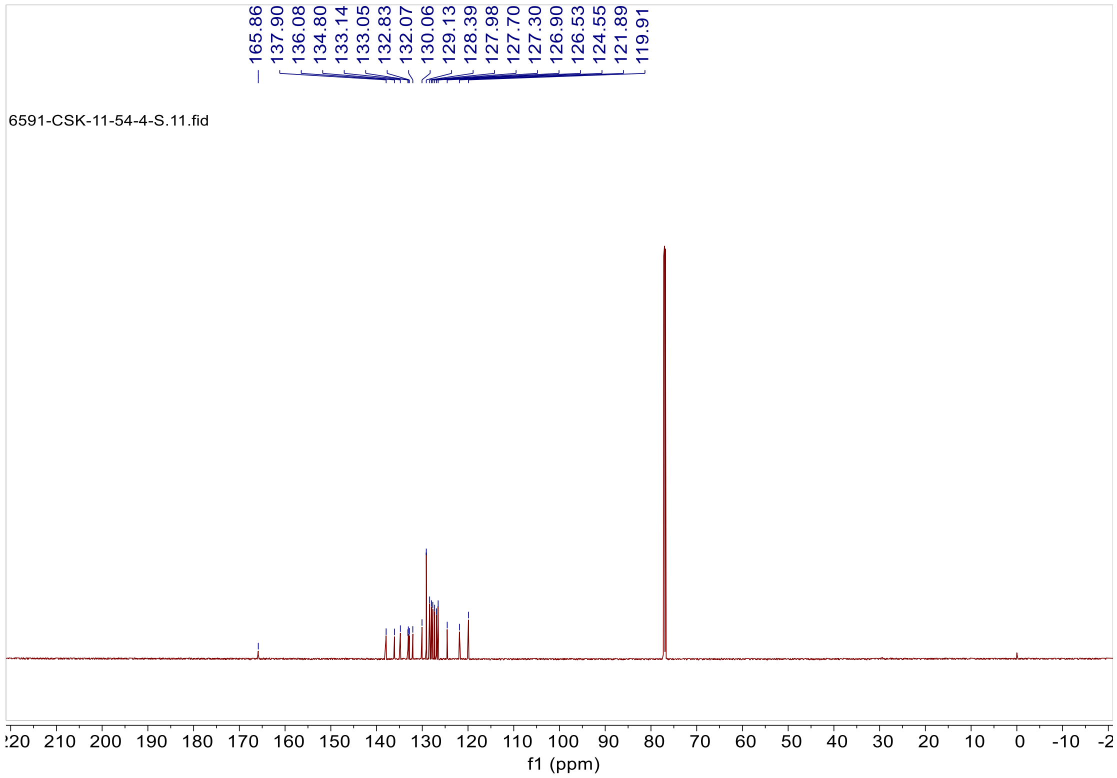

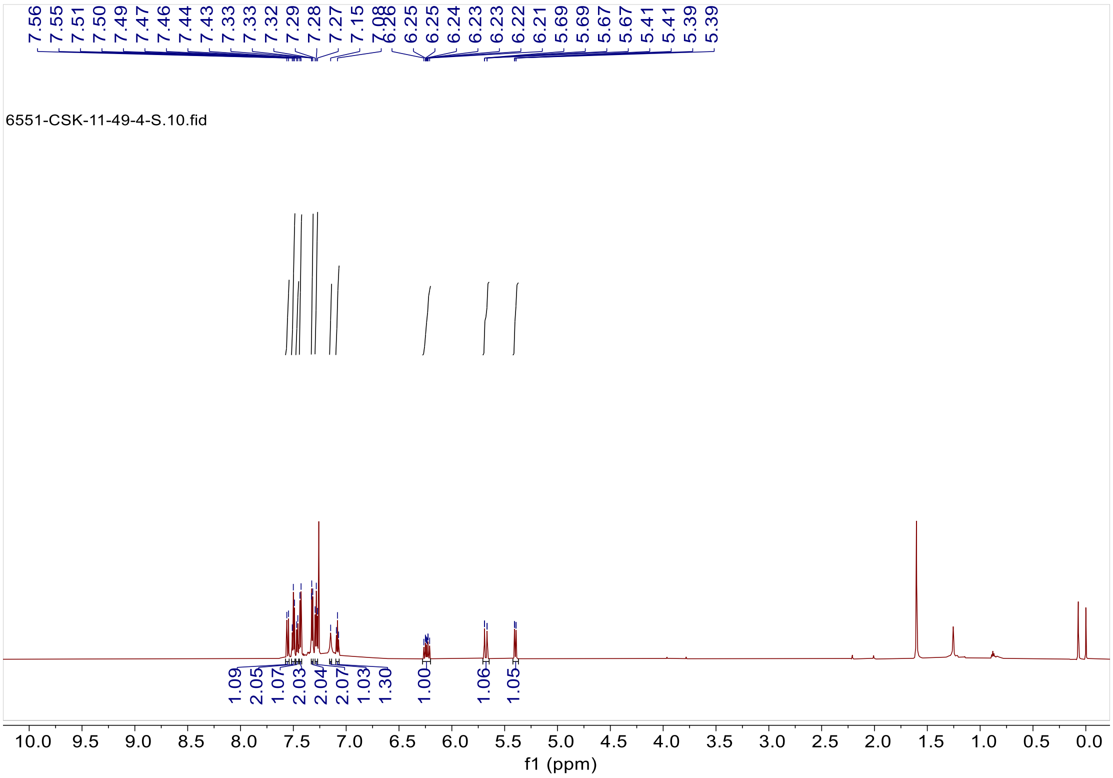

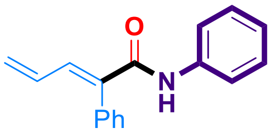

4a

^{1}H NMR (700 MHz, $CDCl_3$)
^{13}C NMR (176 MHz, $CDCl_3$)

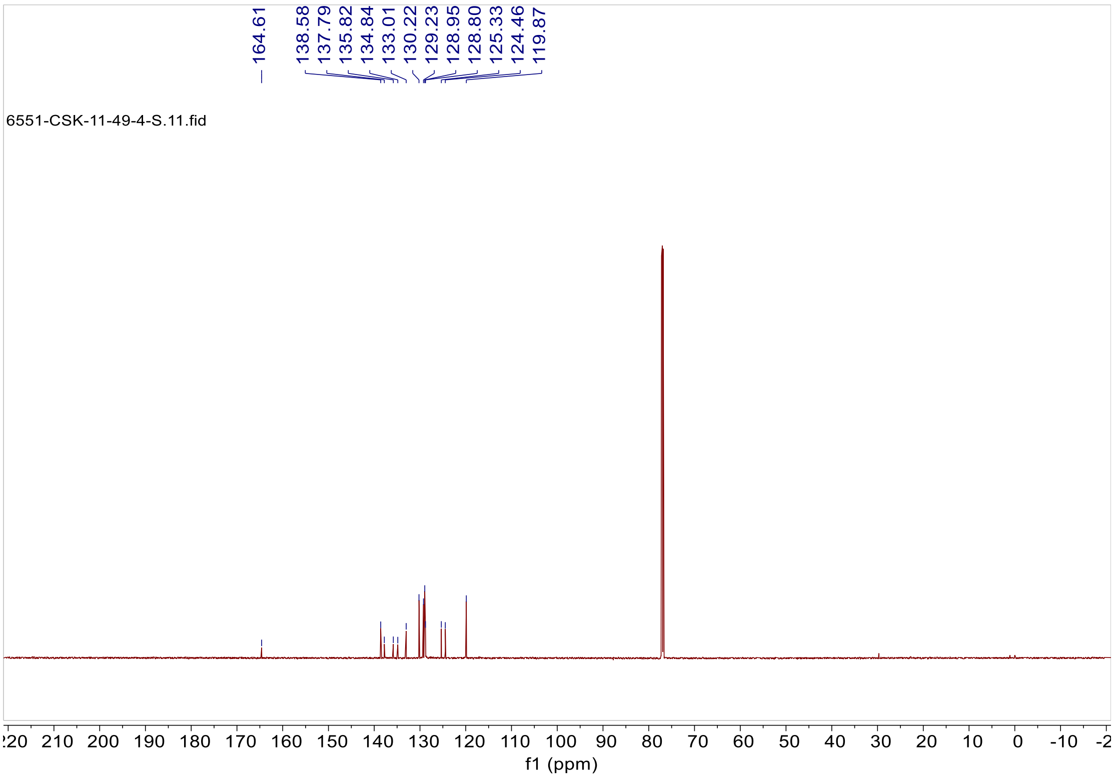

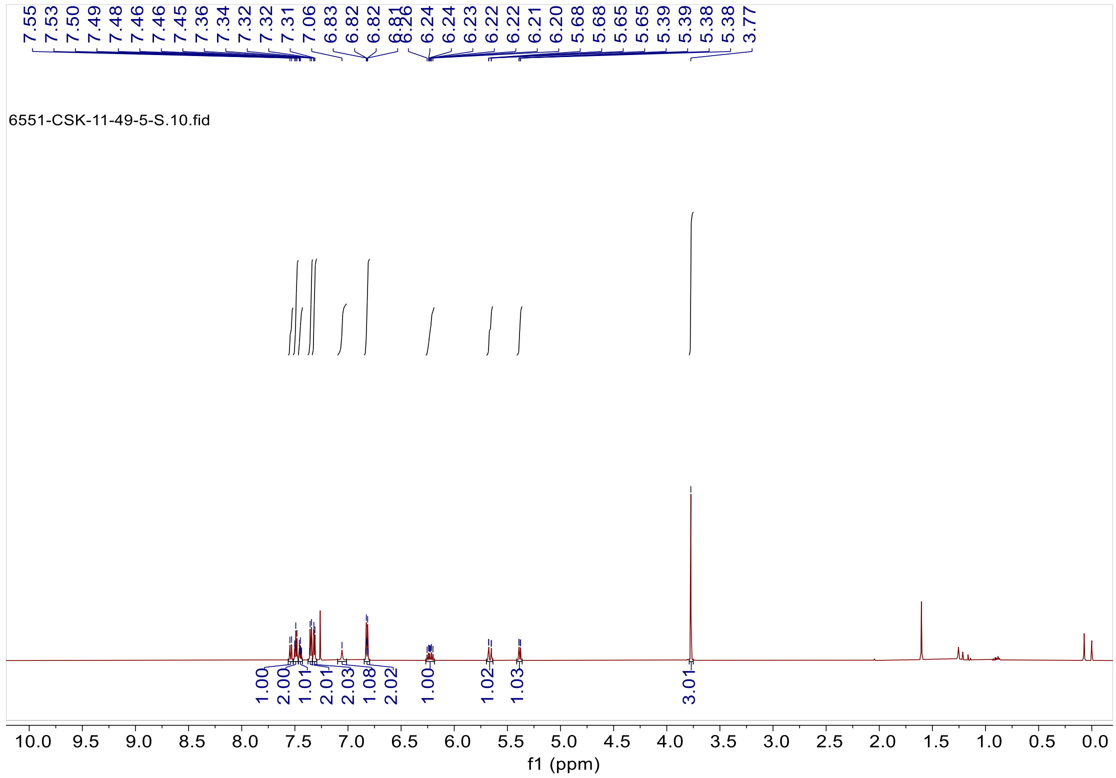

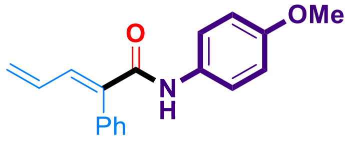

4b

^{1}H NMR (700 MHz, $CDCl_3$)

^{13}C NMR (176 MHz, $CDCl_3$)

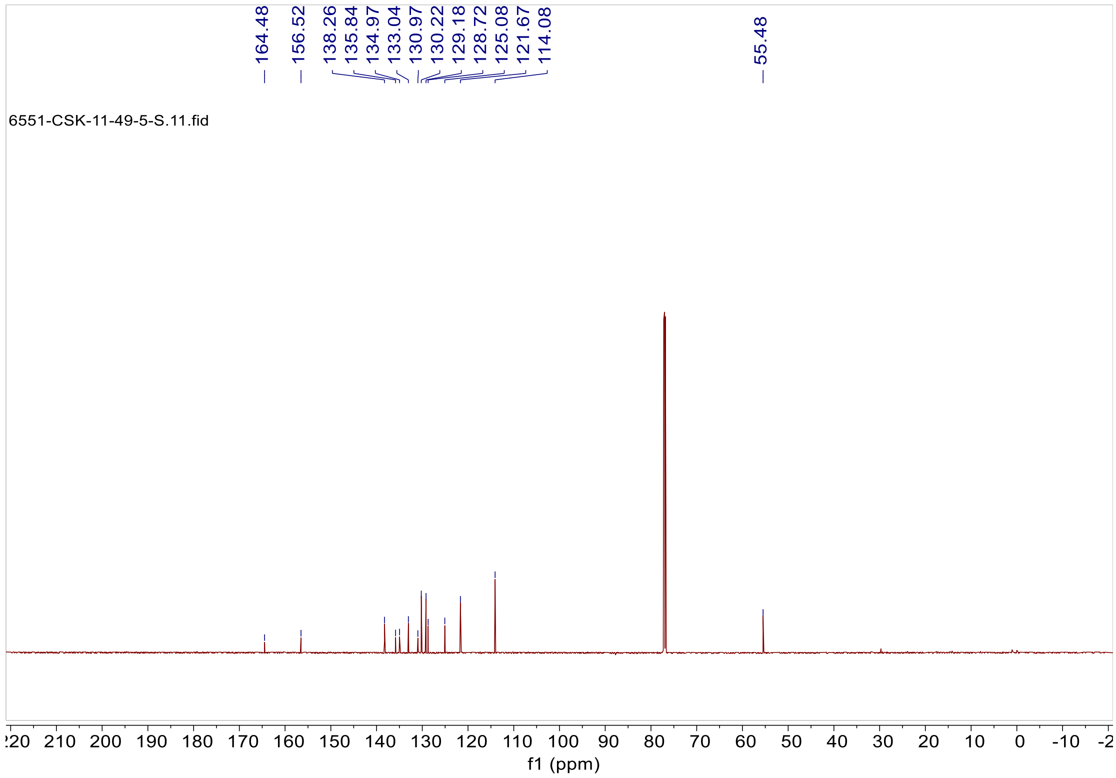

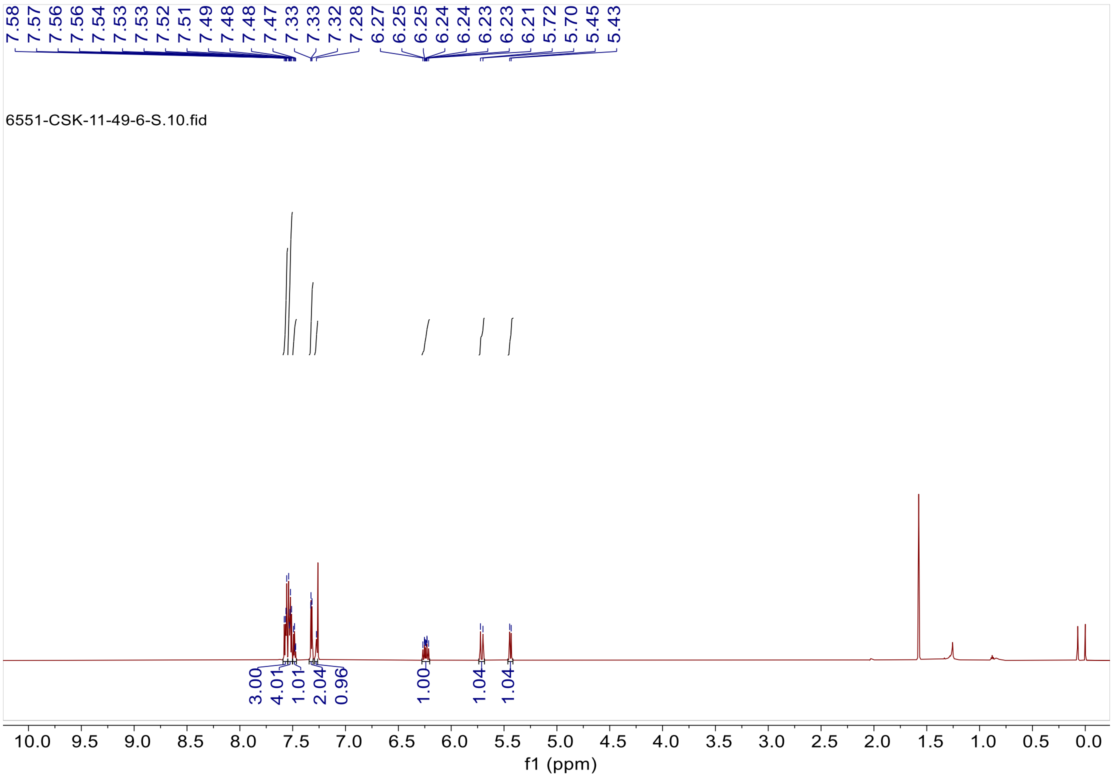

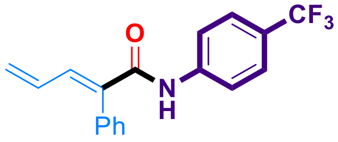

4c

^{1}H NMR (700 MHz, $CDCl_3$)
^{13}C NMR (176 MHz, $CDCl_3$)

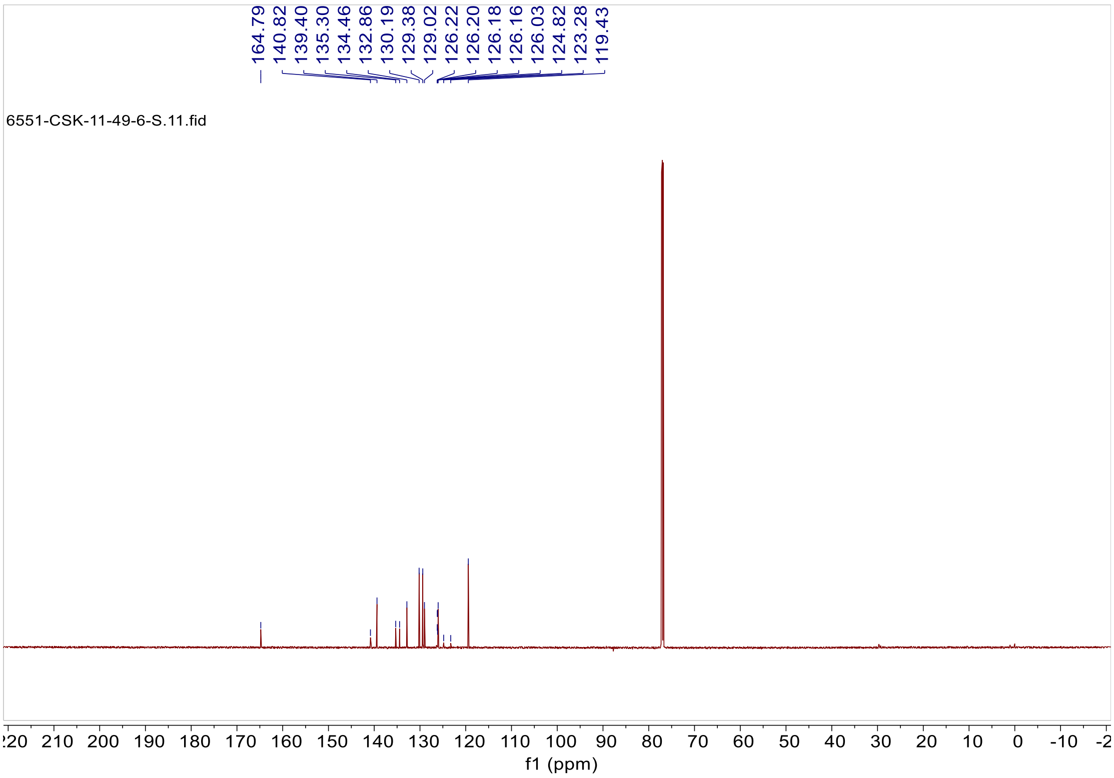

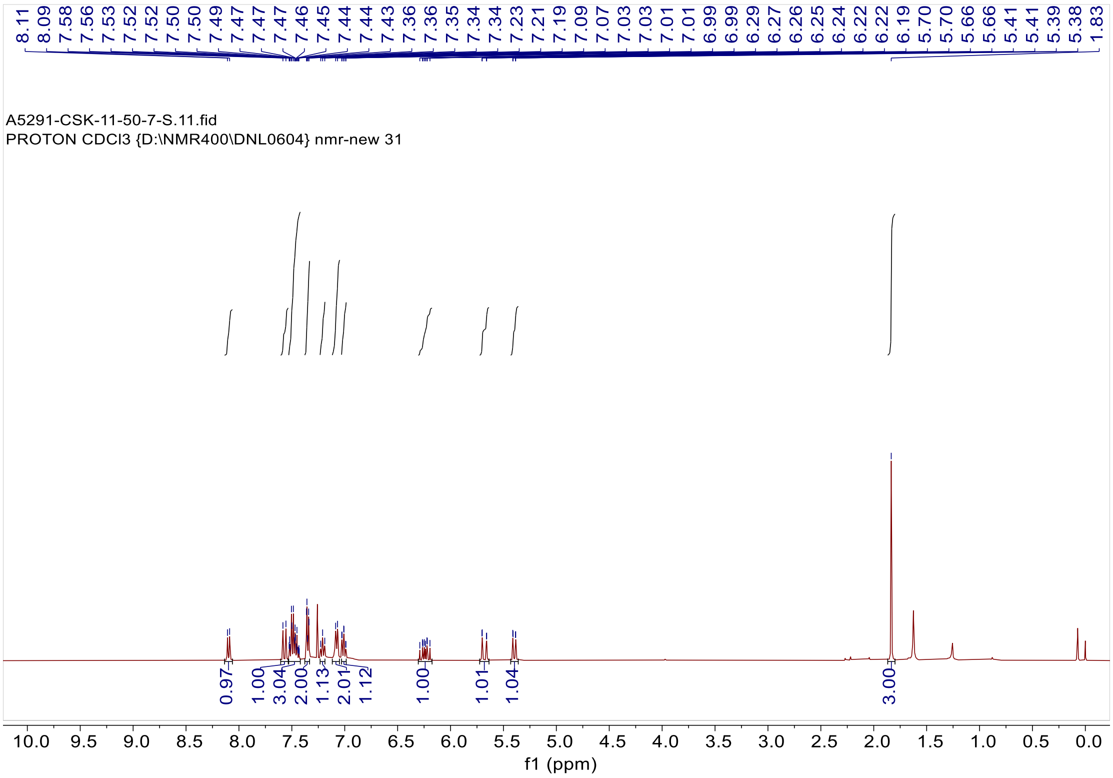

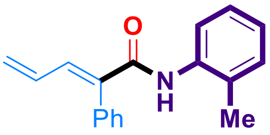

4d

^{1}H NMR (400 MHz, $CDCl_3$)
^{13}C NMR (100 MHz, $CDCl_3$)

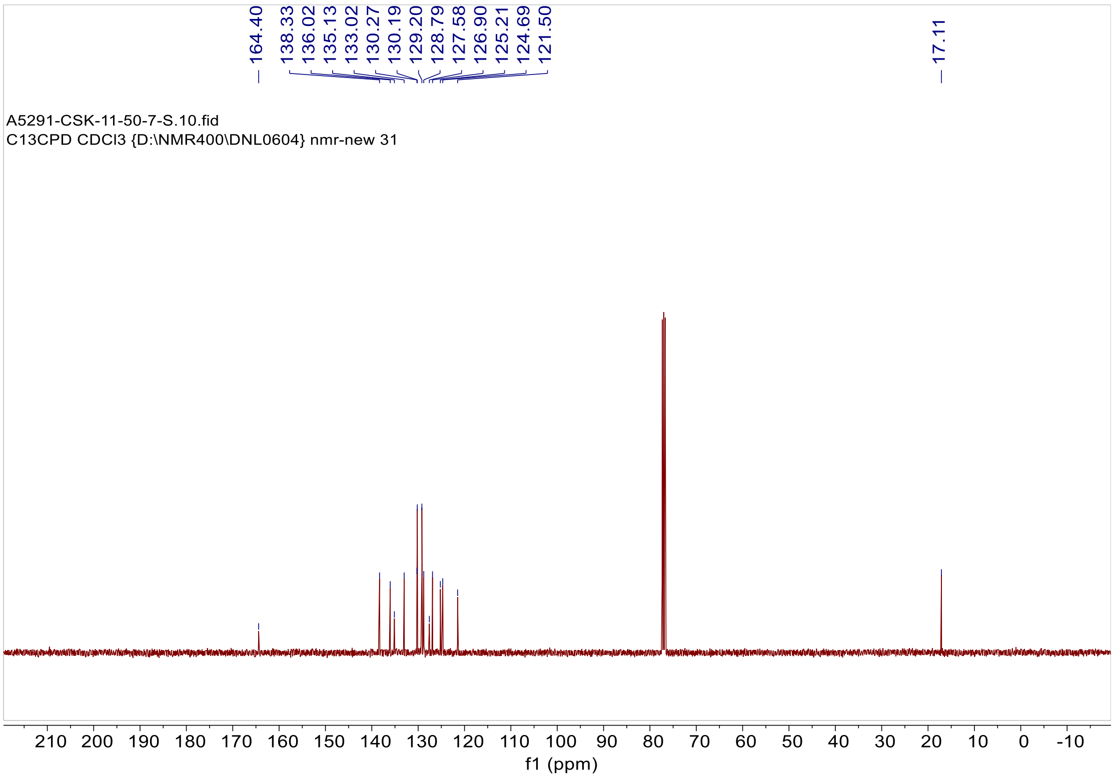

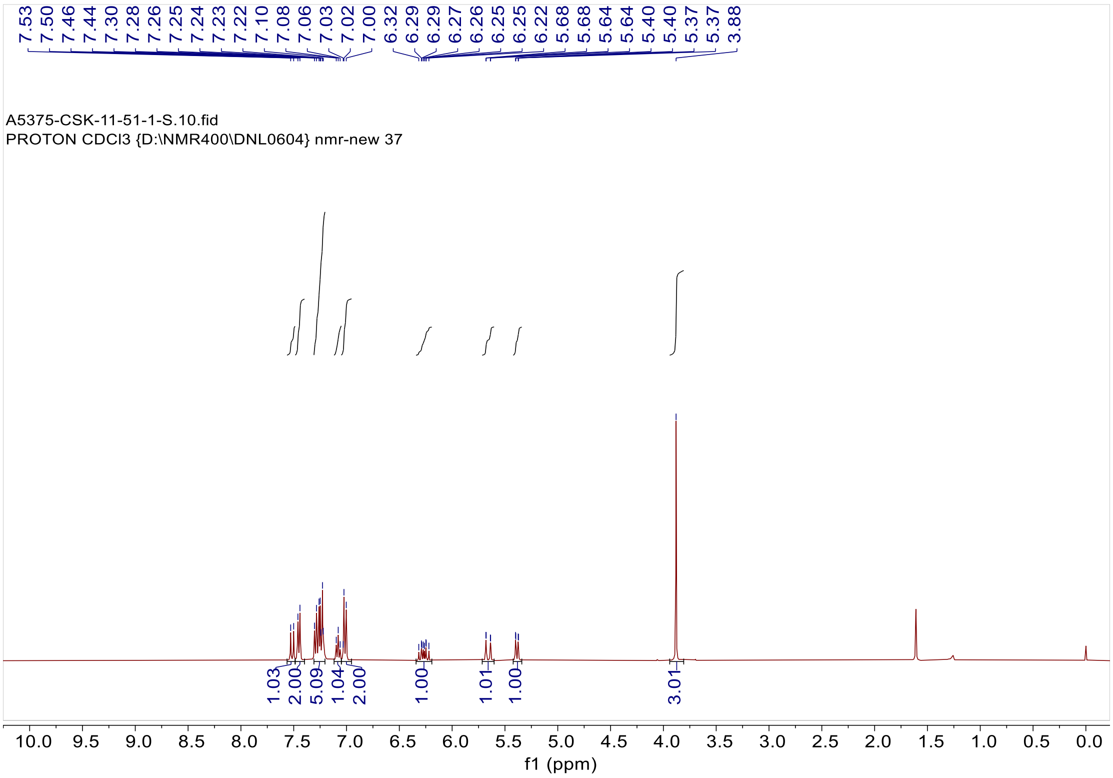

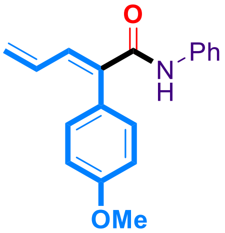

4e

^{1}H NMR (400 MHz, $CDCl_3$)
^{13}C NMR (100 MHz, $CDCl_3$)

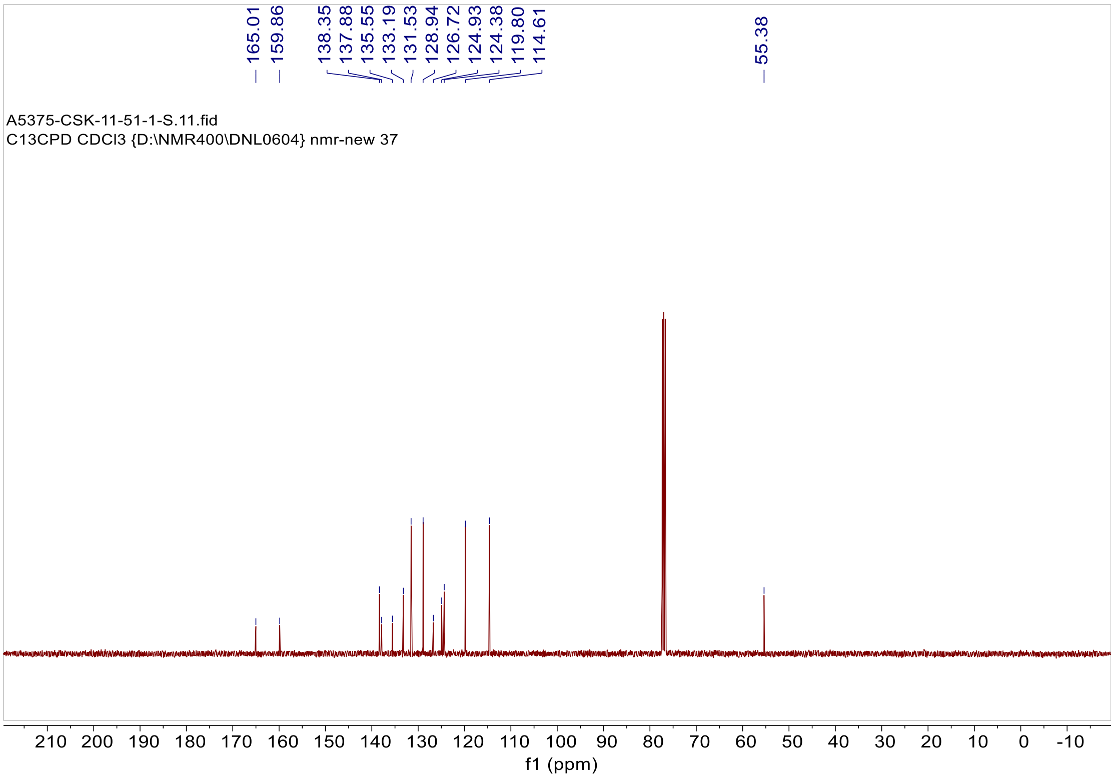

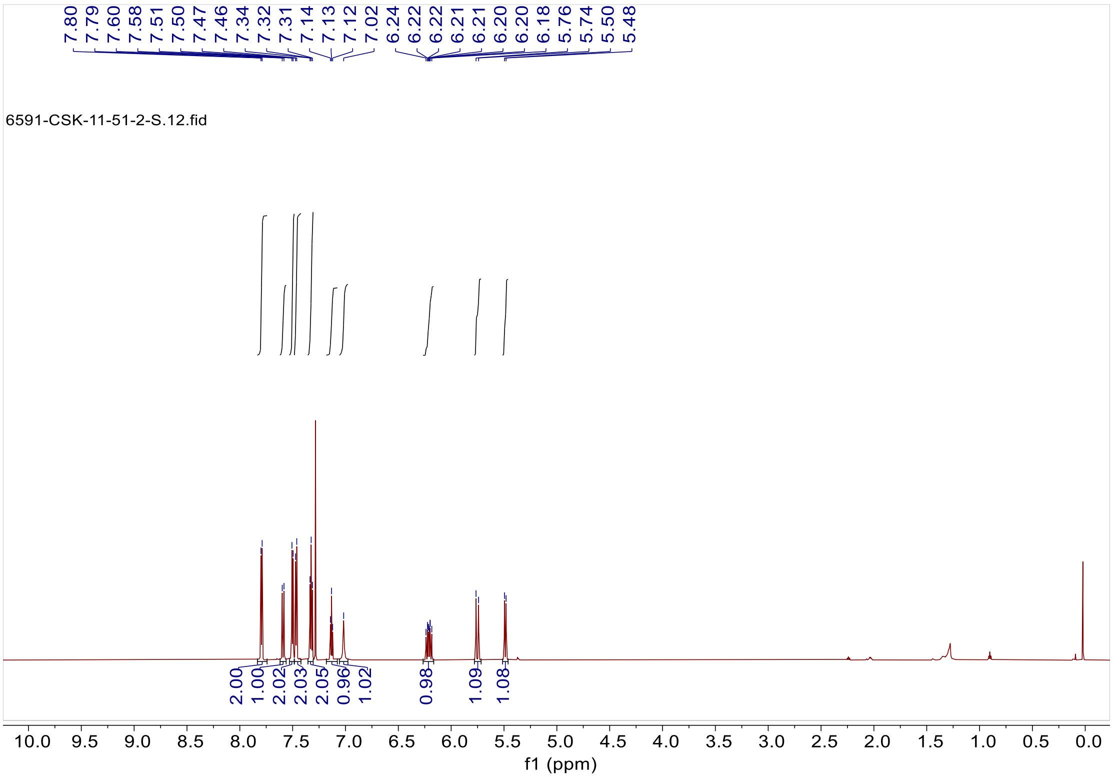

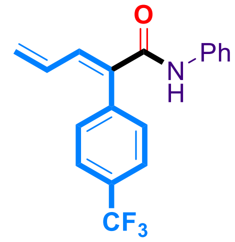

4f

^{1}H NMR (700 MHz, $CDCl_3$)
^{13}C NMR (176 MHz, $CDCl_3$)

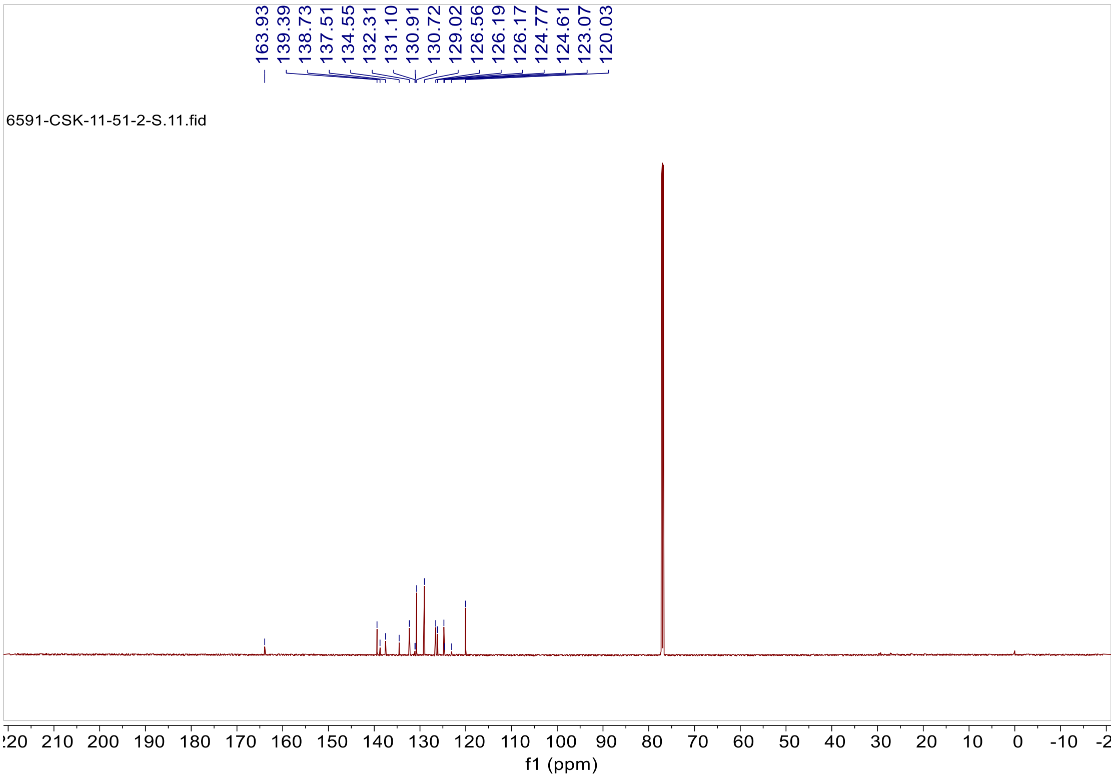

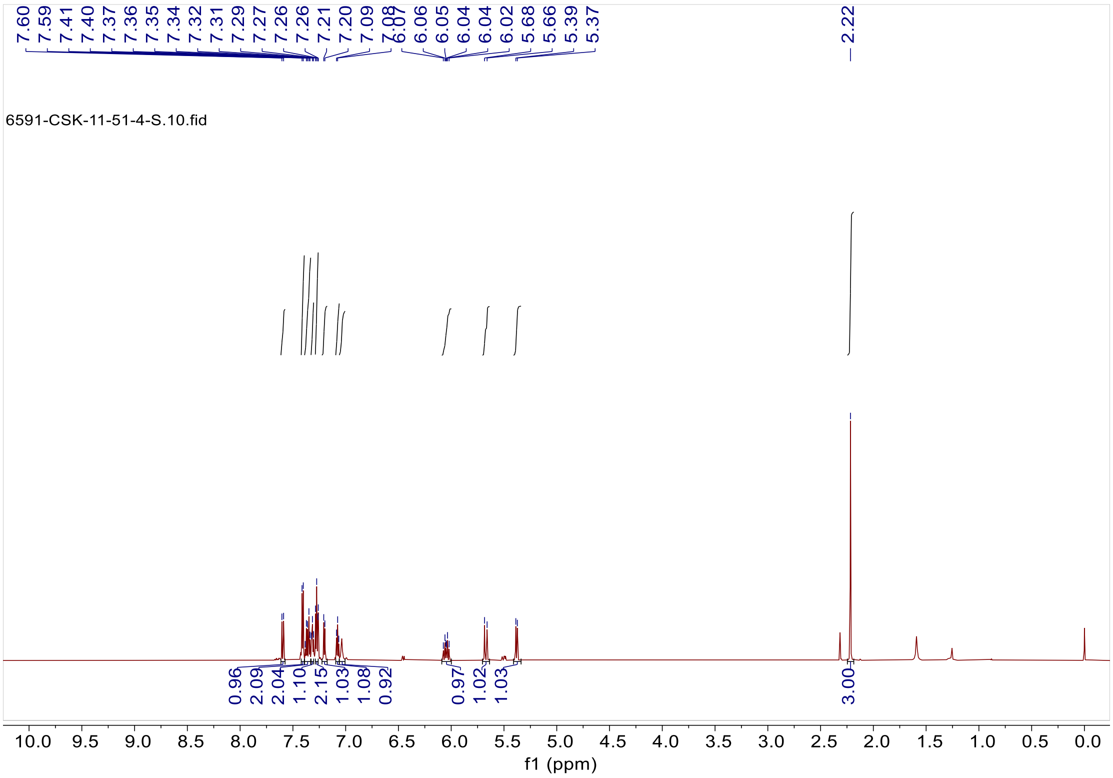

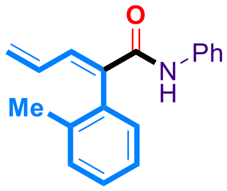

4g

^{1}H NMR (700 MHz, $CDCl_3$)
^{13}C NMR (176 MHz, $CDCl_3$)

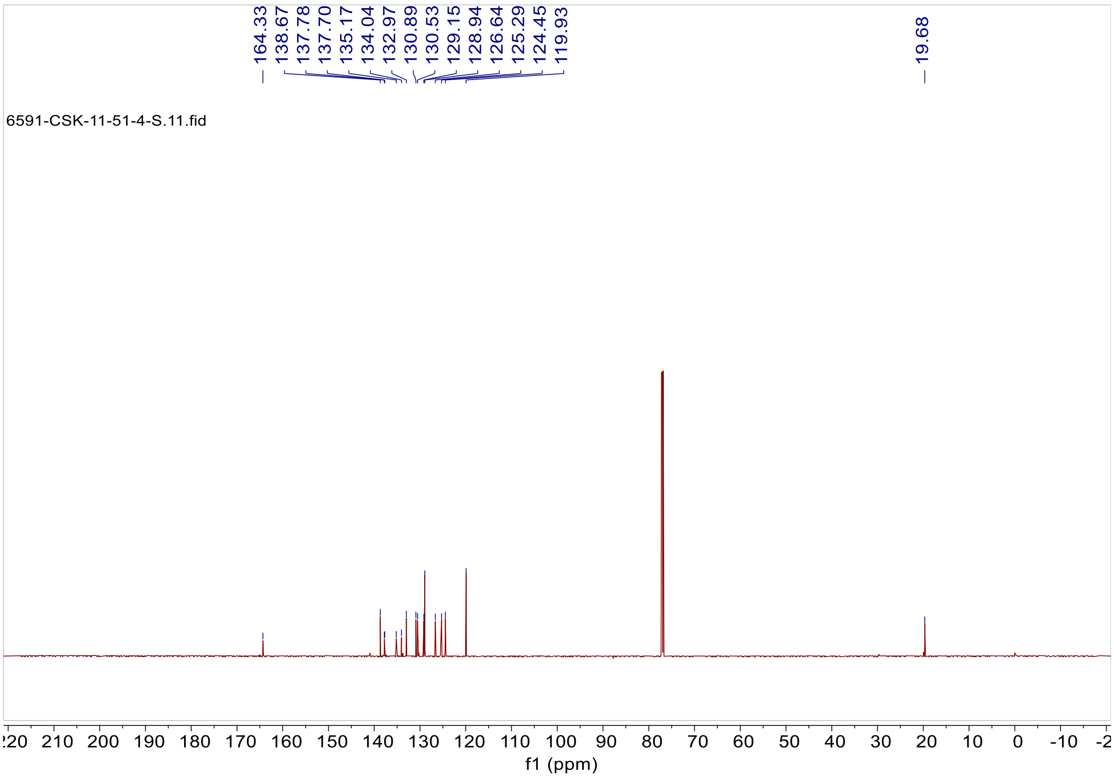

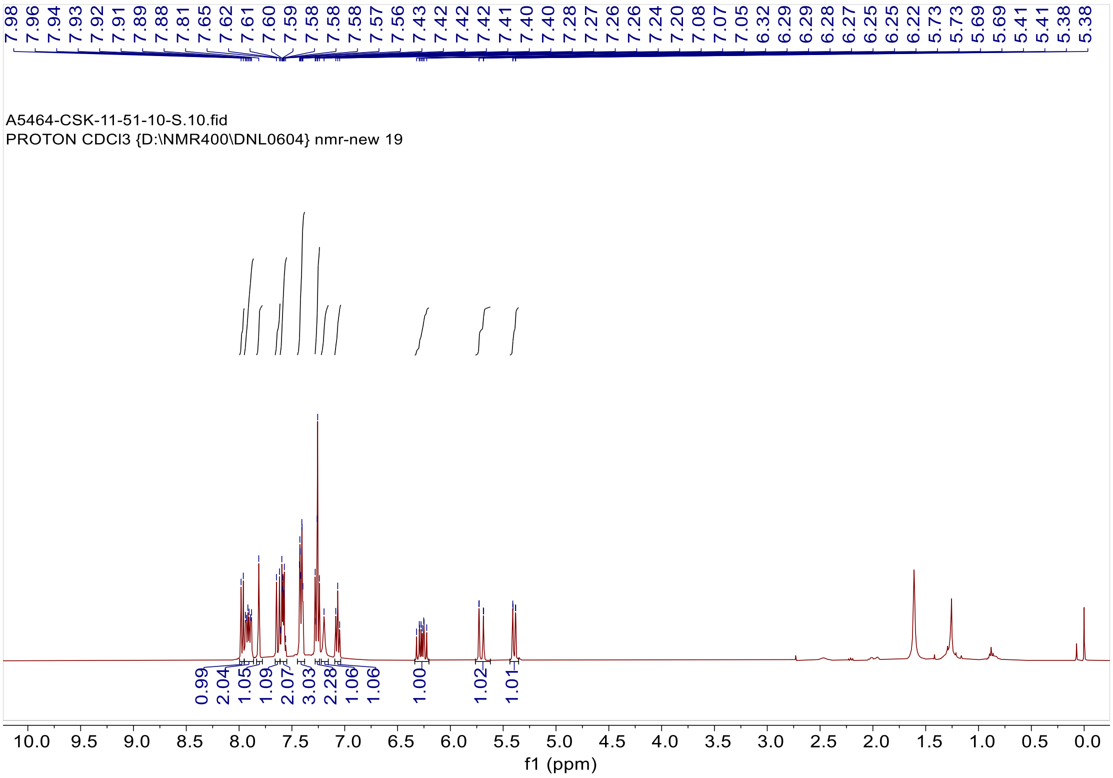

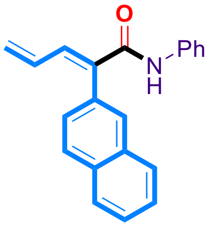

4h

^{1}H NMR (400 MHz, $CDCl_3$)
^{13}C NMR (100 MHz, $CDCl_3$)

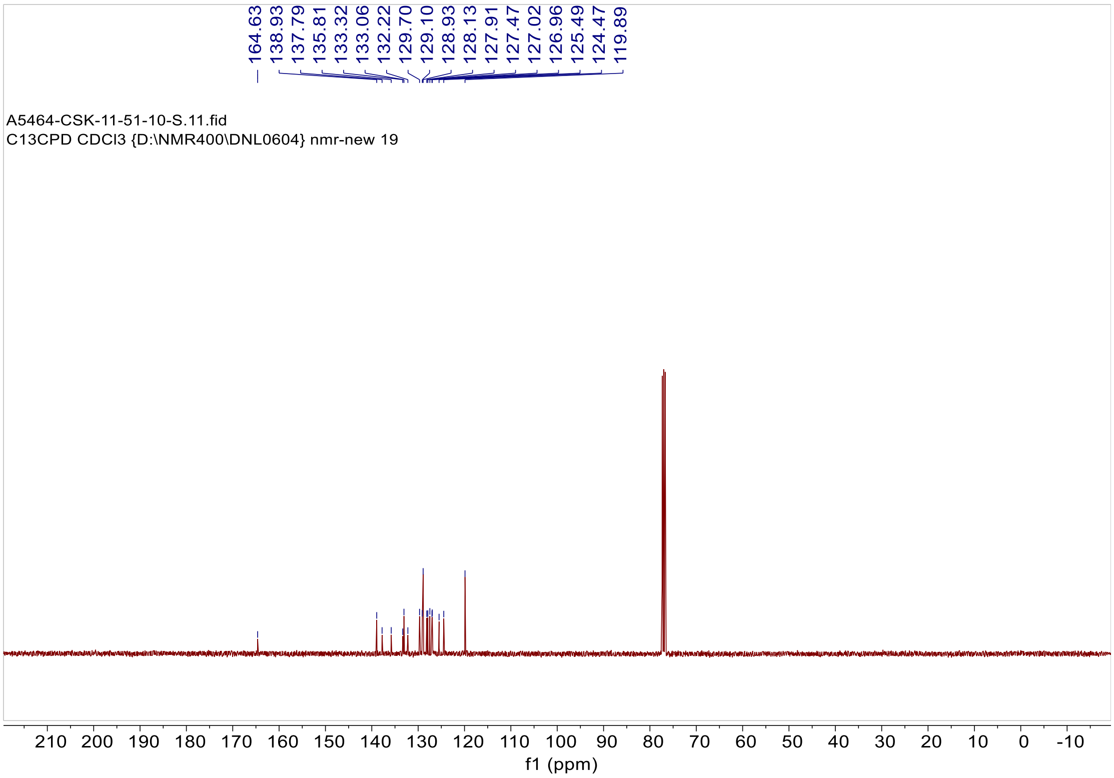

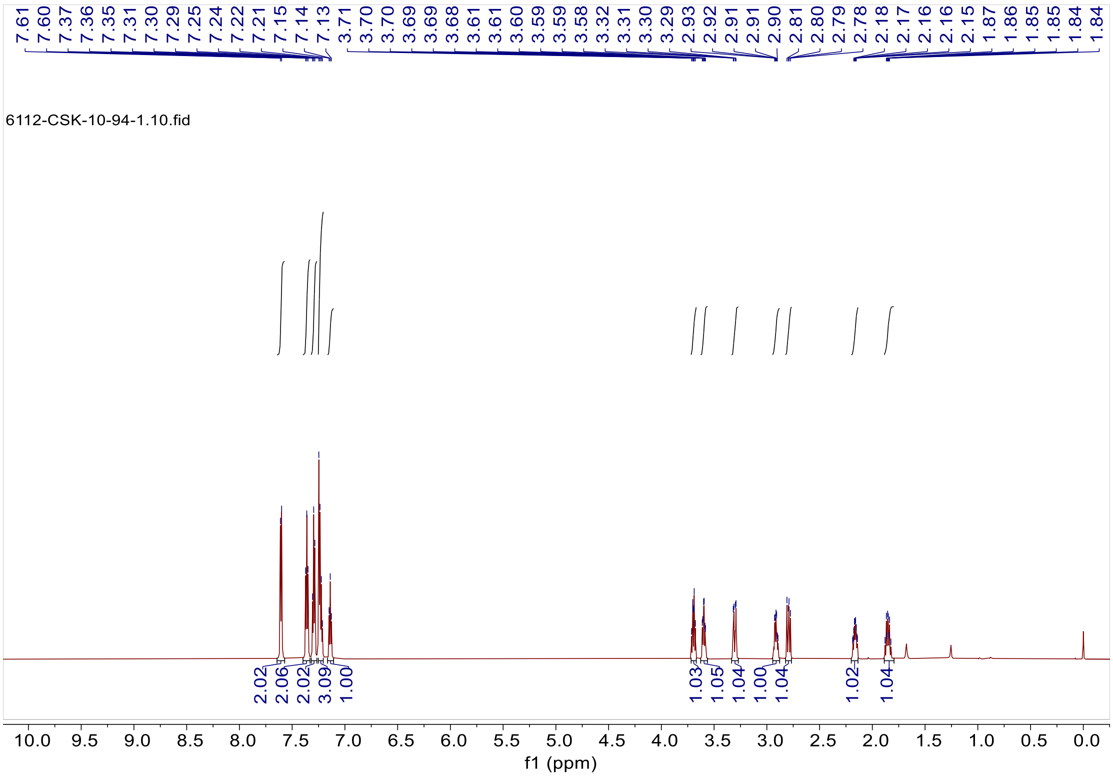

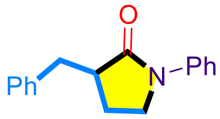

5a1

^{1}H NMR (400 MHz, $CDCl_3$)
^{13}C NMR (100 MHz, $CDCl_3$)

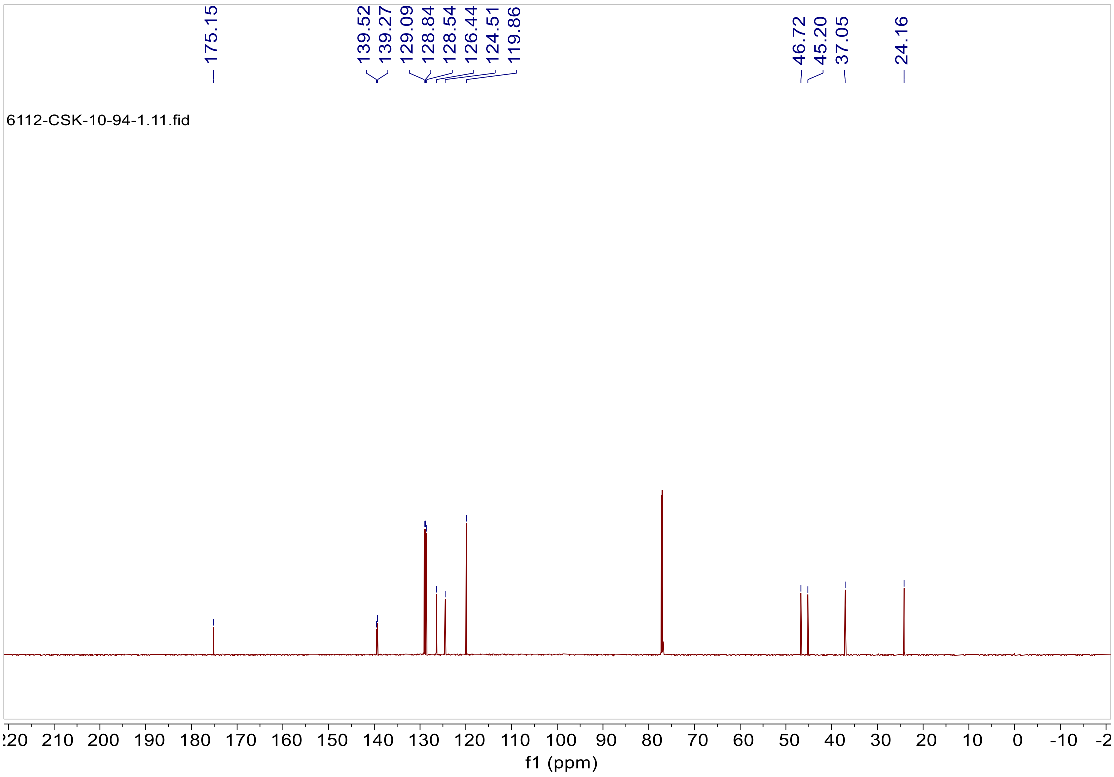

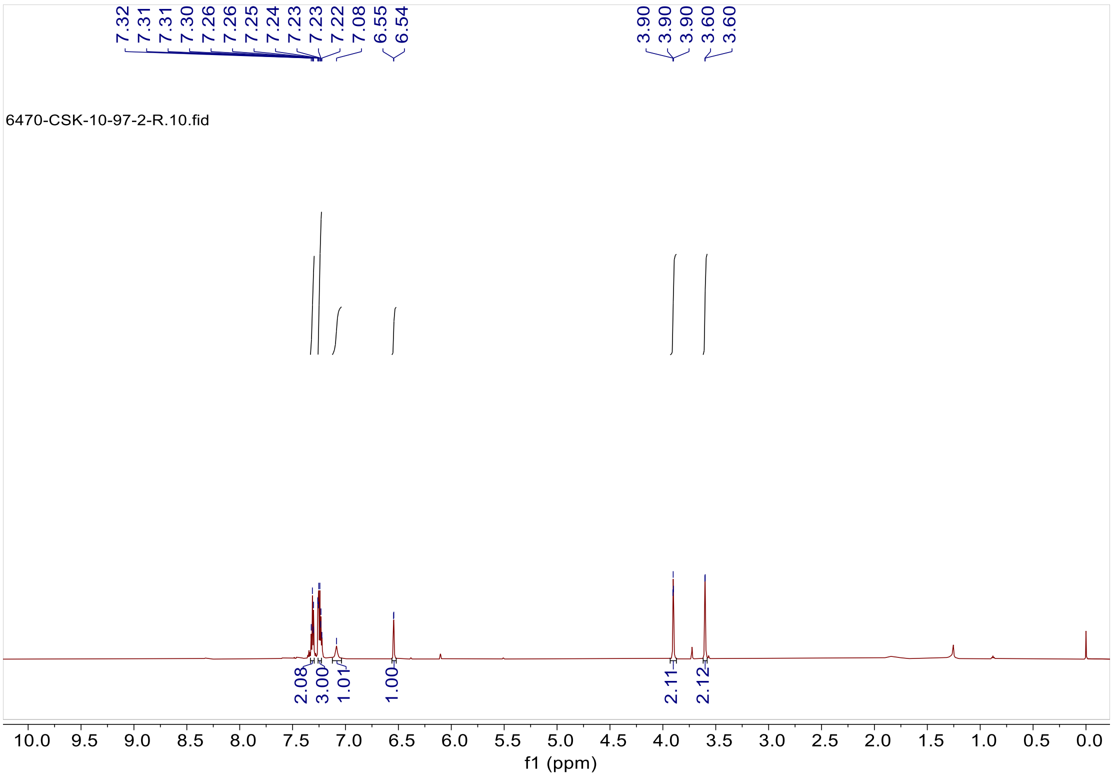

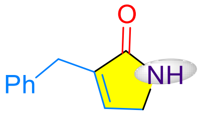

5d1

^{1}H NMR (700 MHz, $CDCl_3$)

^{13}C NMR (176 MHz, $CDCl_3$)

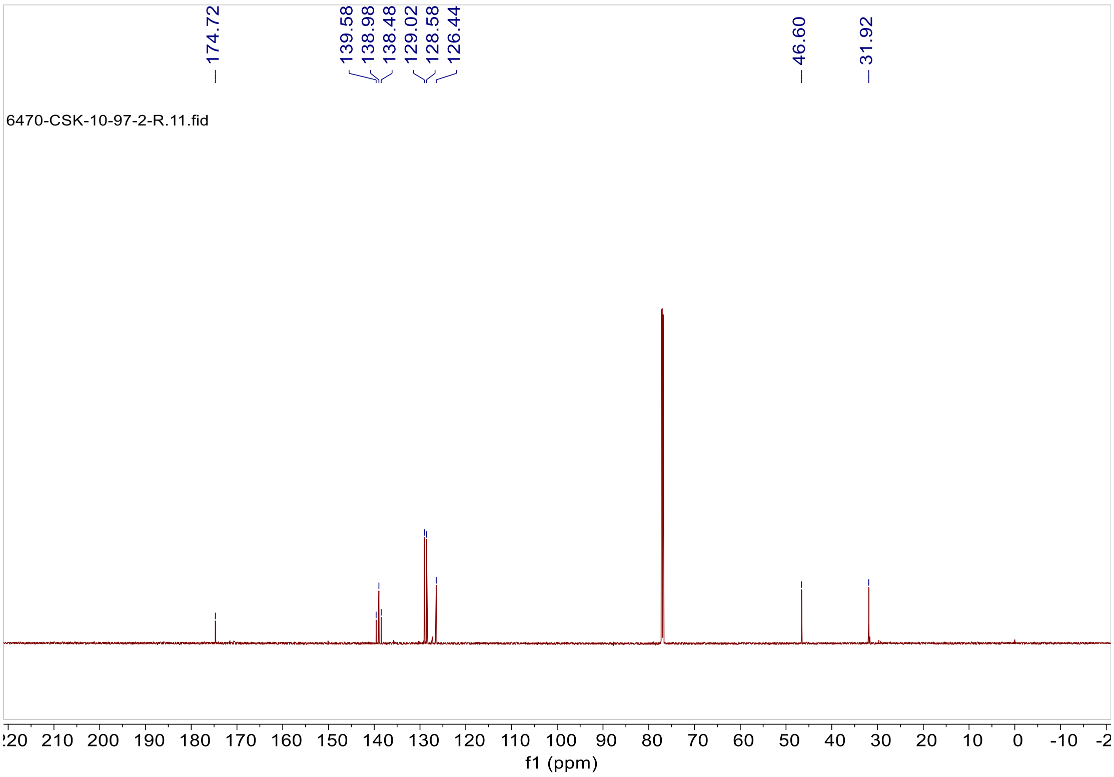

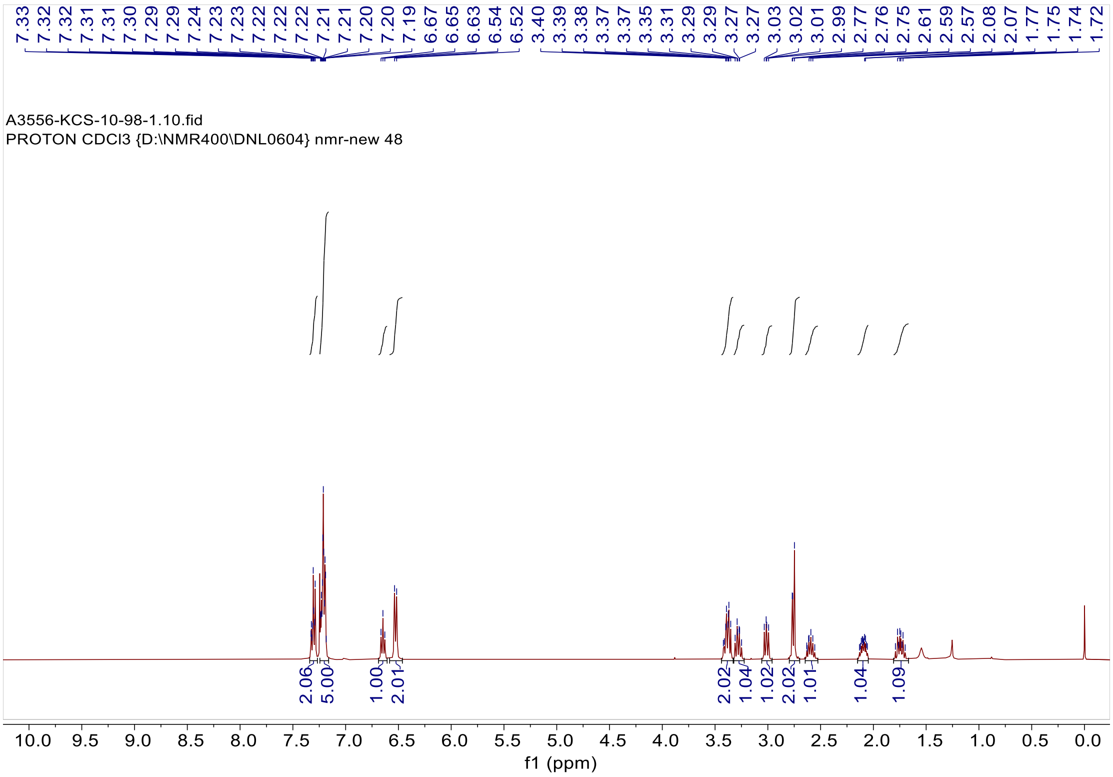

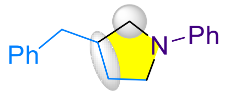

5a2

^{1}H NMR (400 MHz, $CDCl_3$)

^{13}C NMR (100 MHz, $CDCl_3$)

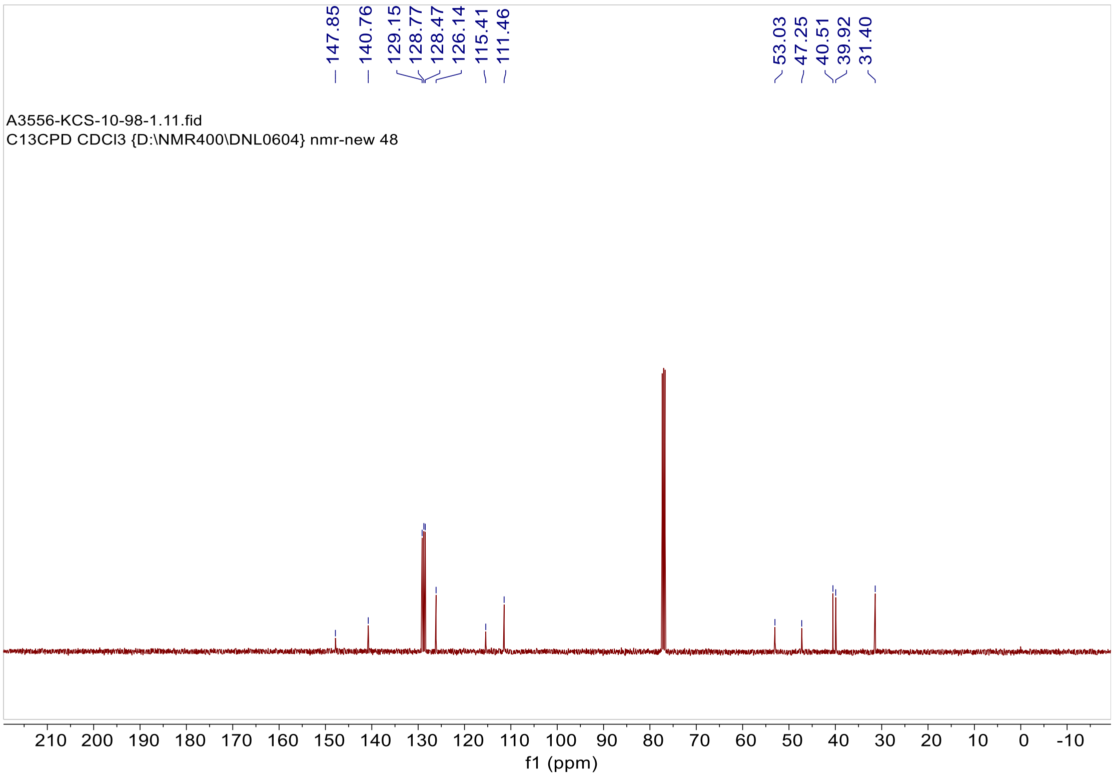

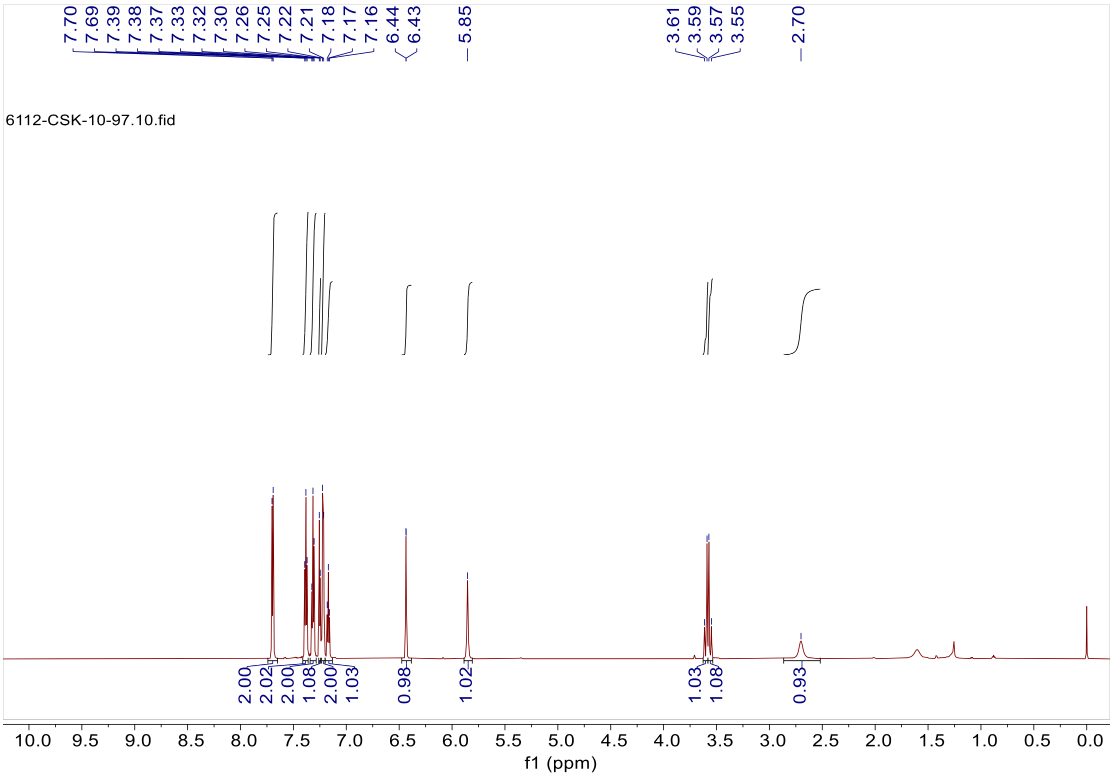

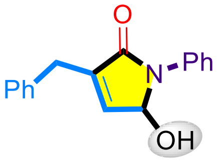

5a3

^{1}H NMR (700 MHz, $CDCl_3$)
^{13}C NMR (176 MHz, $CDCl_3$)

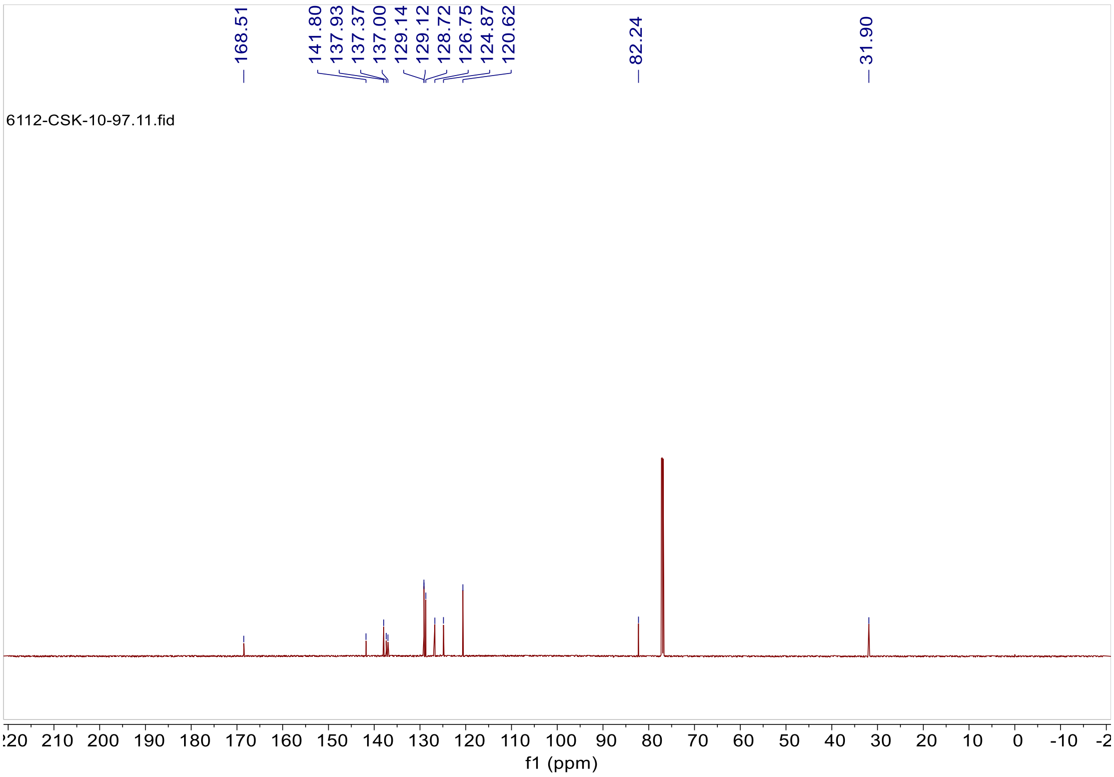

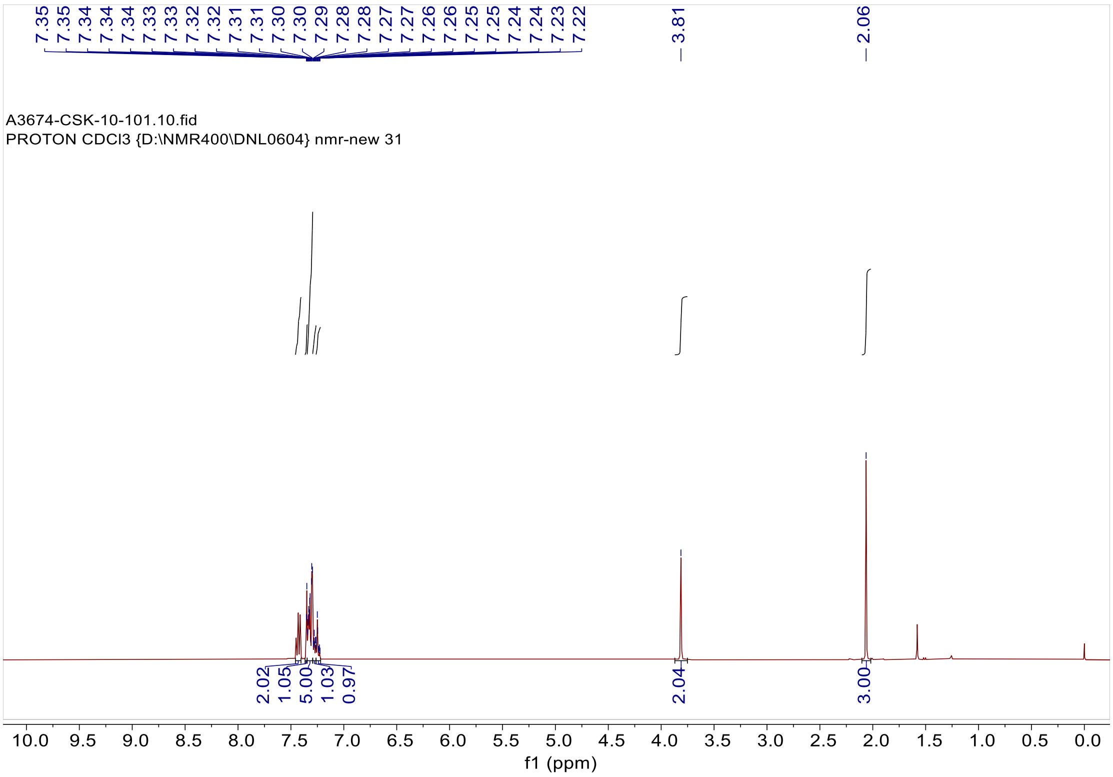

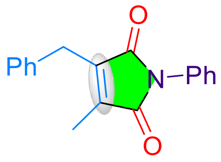

6a'

^{1}H NMR (400 MHz, $CDCl_3$)

^{13}C NMR (100 MHz, $CDCl_3$)

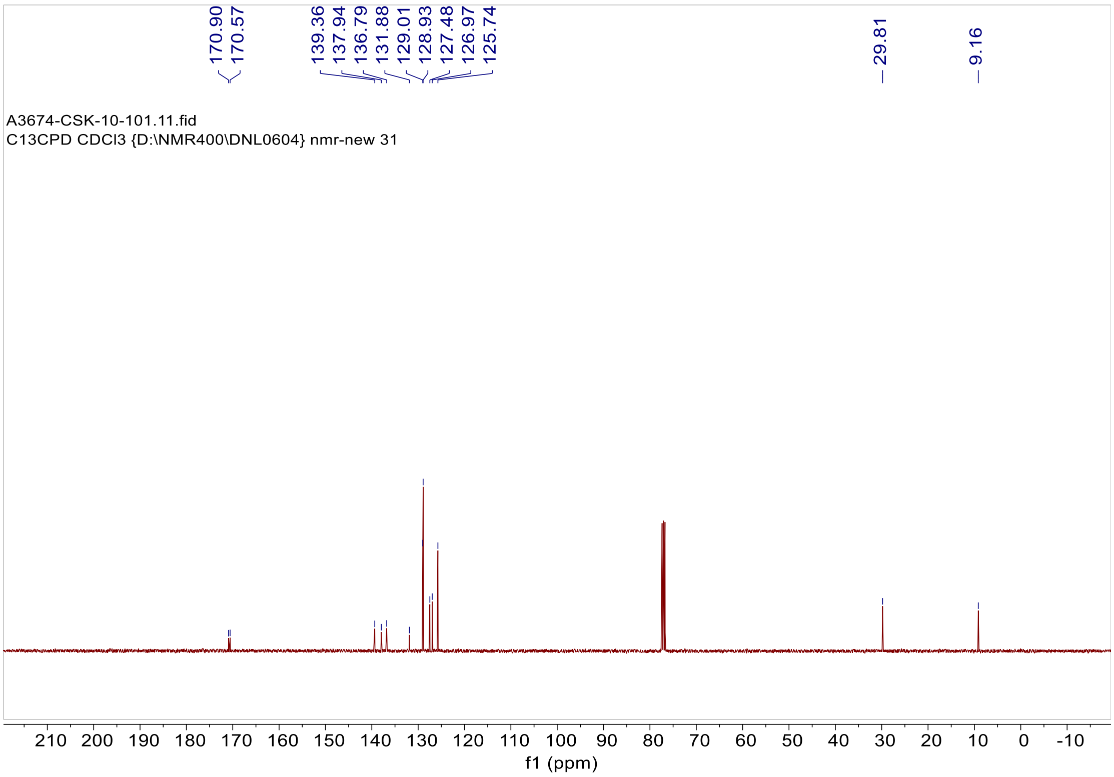

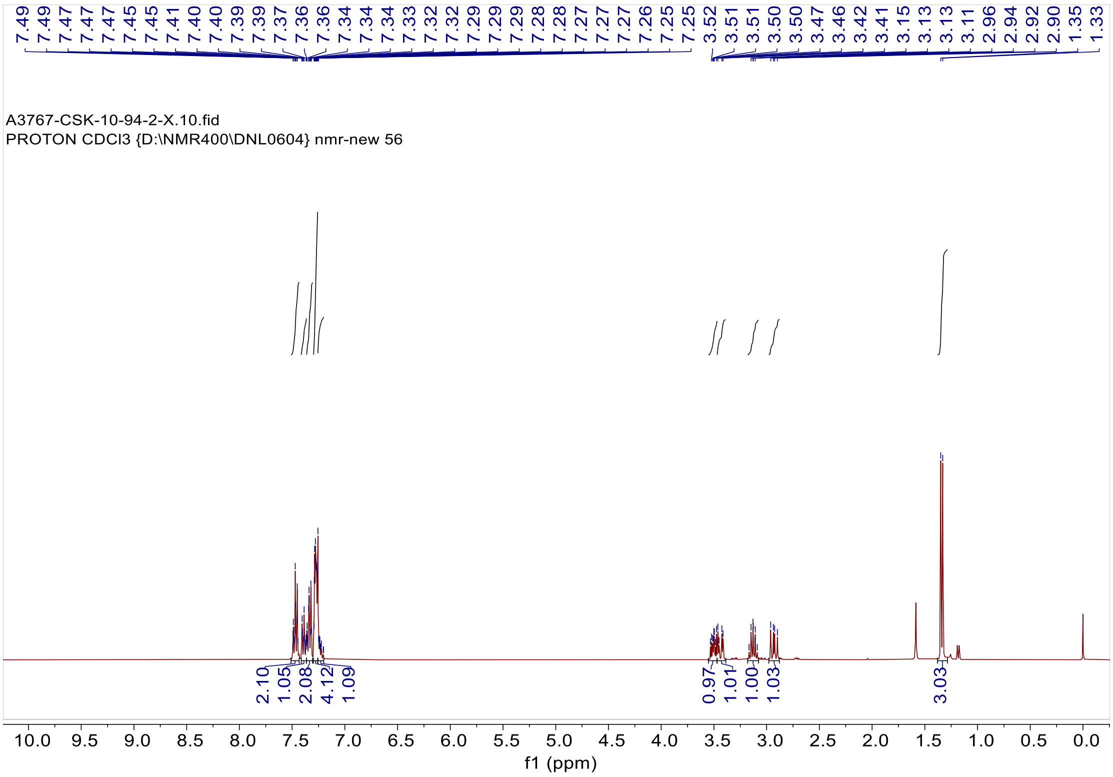

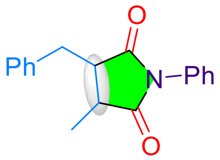

6a1

^{1}H NMR (400 MHz, $CDCl_3$)
^{13}C NMR (100 MHz, $CDCl_3$)

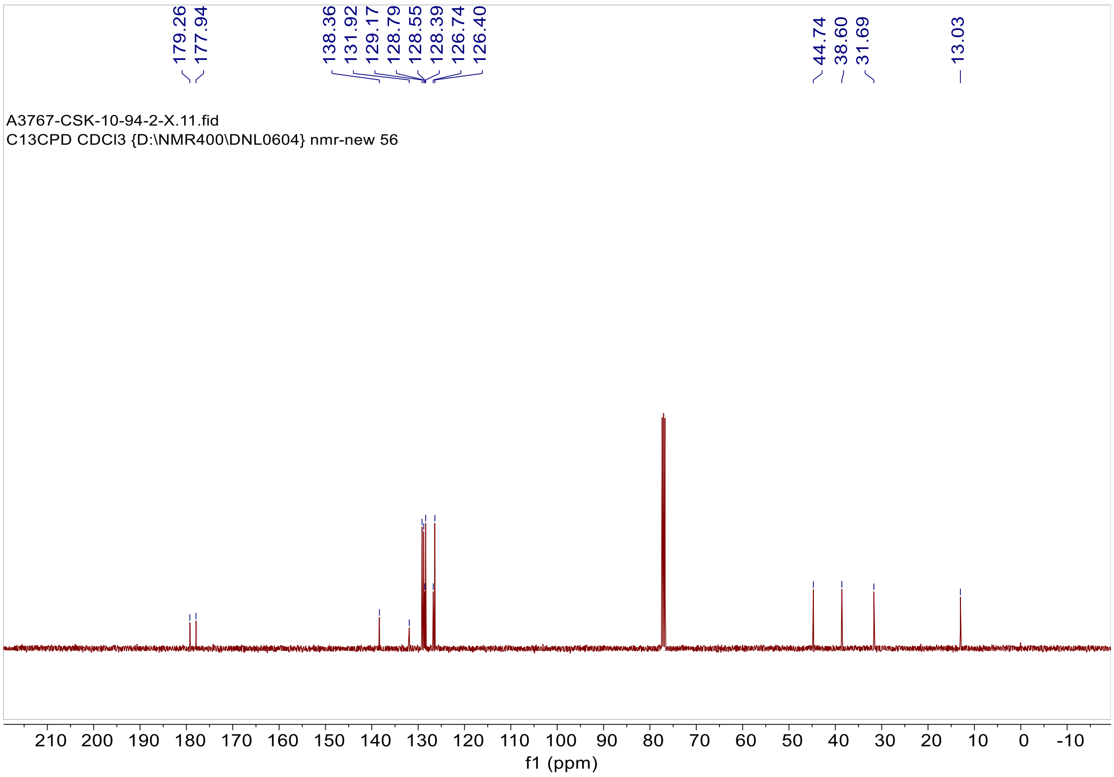

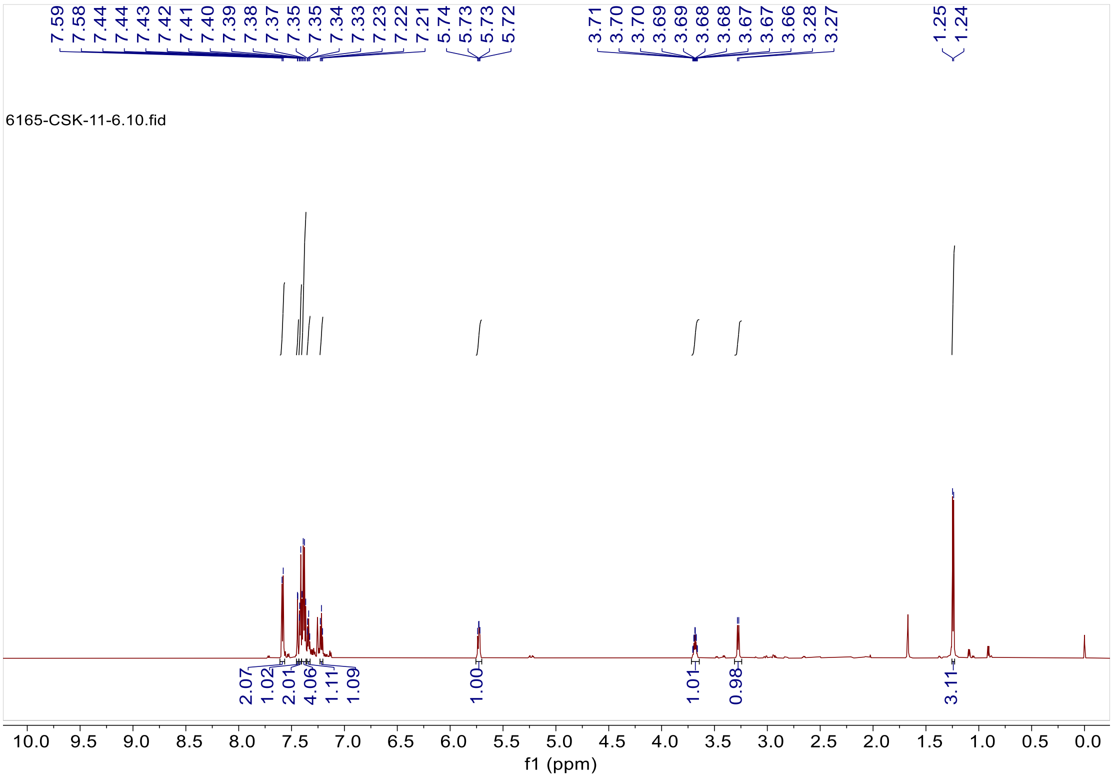

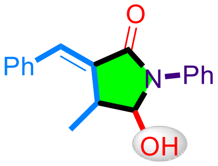

6a3

^{1}H NMR (700 MHz, $CDCl_3$)
^{13}C NMR (176 MHz, $CDCl_3$)

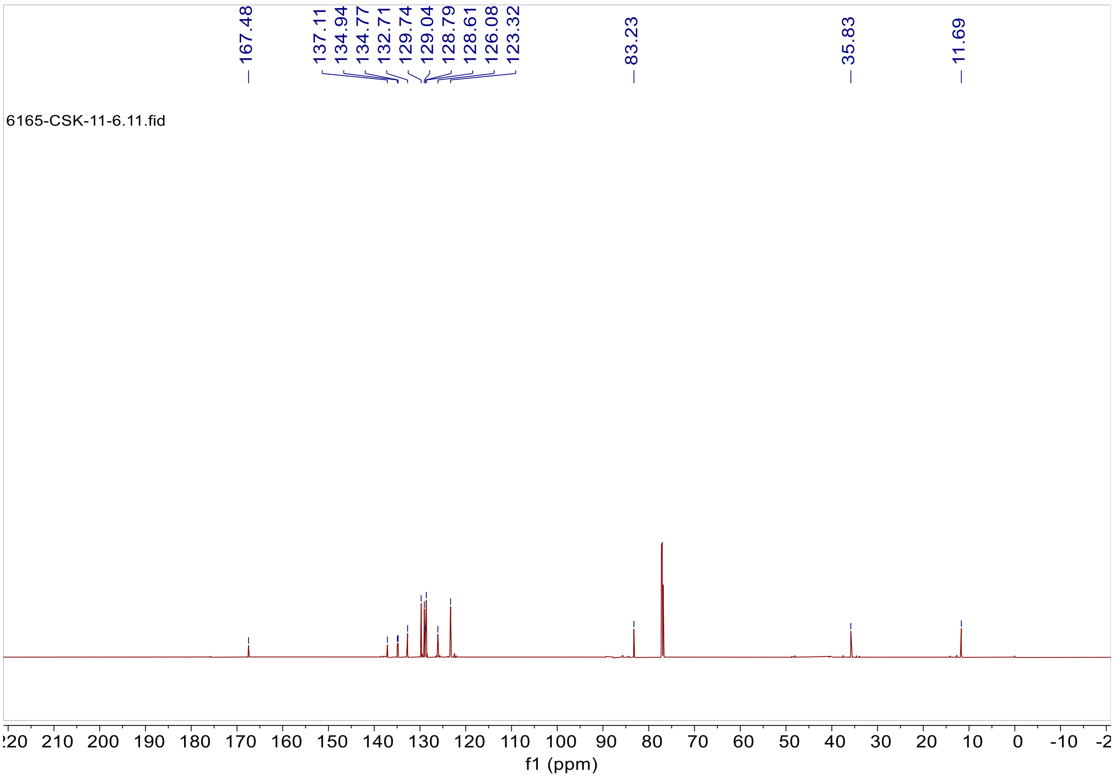

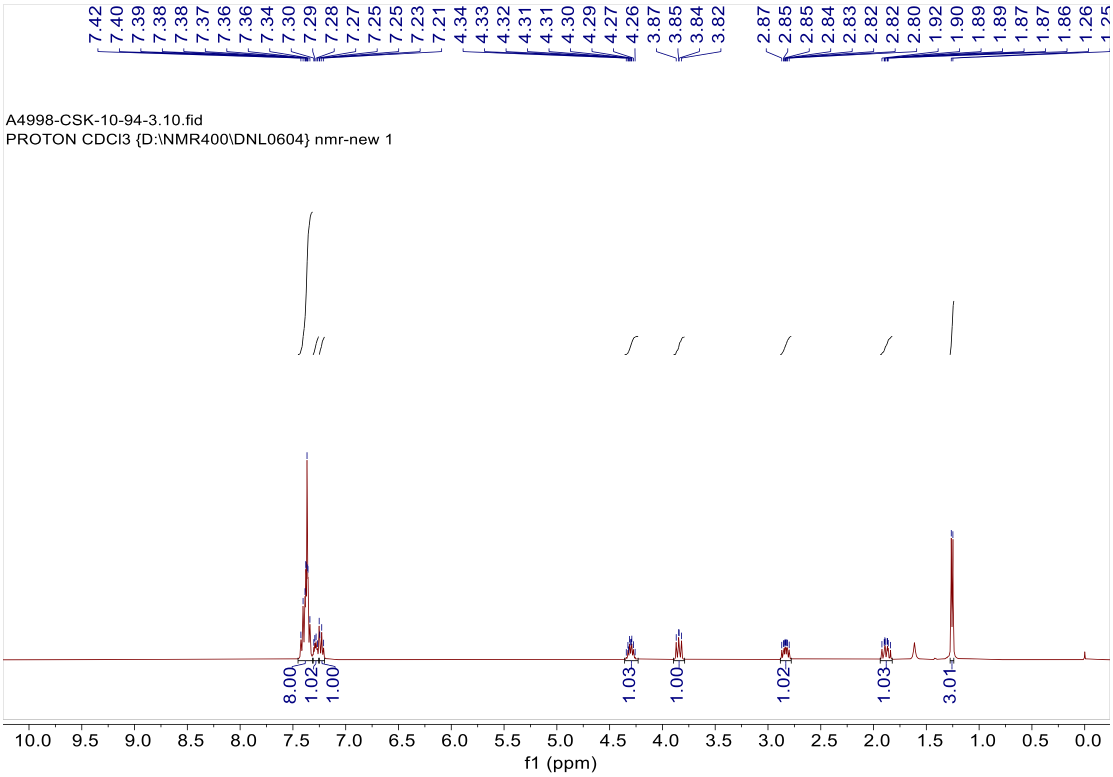

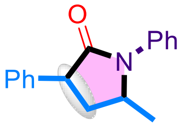

7a1

^{1}H NMR (400 MHz, $CDCl_3$)
^{13}C NMR (100 MHz, $CDCl_3$)

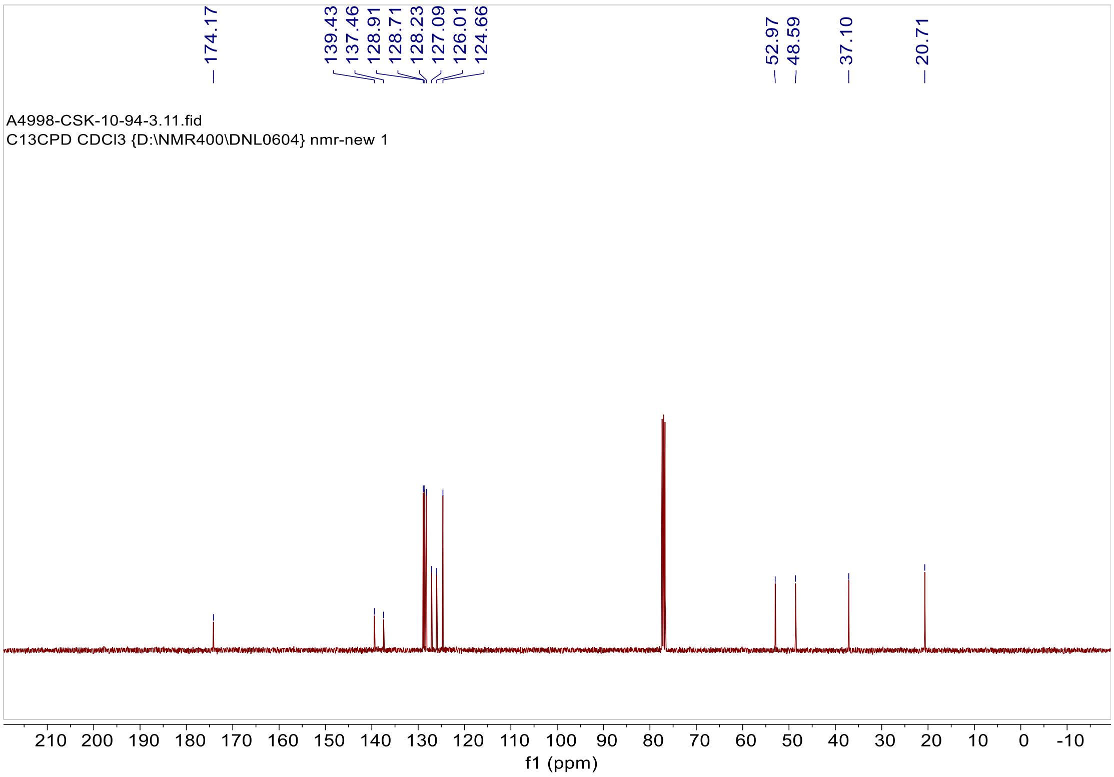

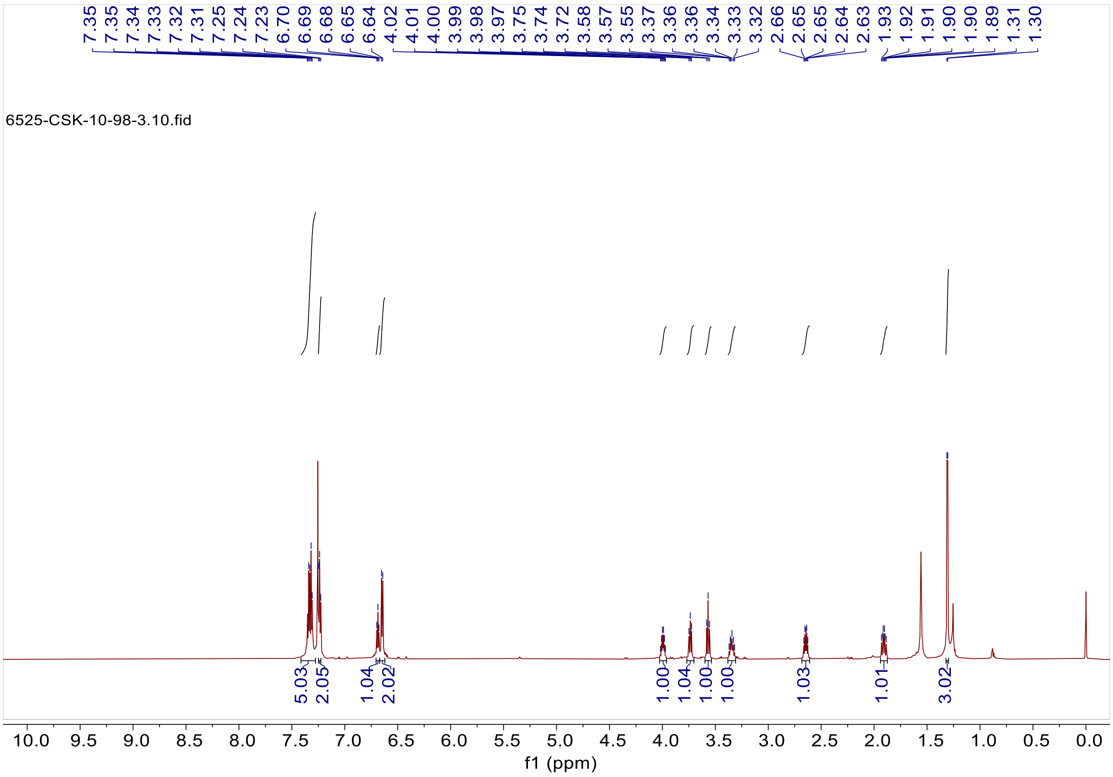

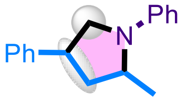

7a2

^{1}H NMR (700 MHz, $CDCl_3$)

^{13}C NMR (176 MHz, $CDCl_3$)

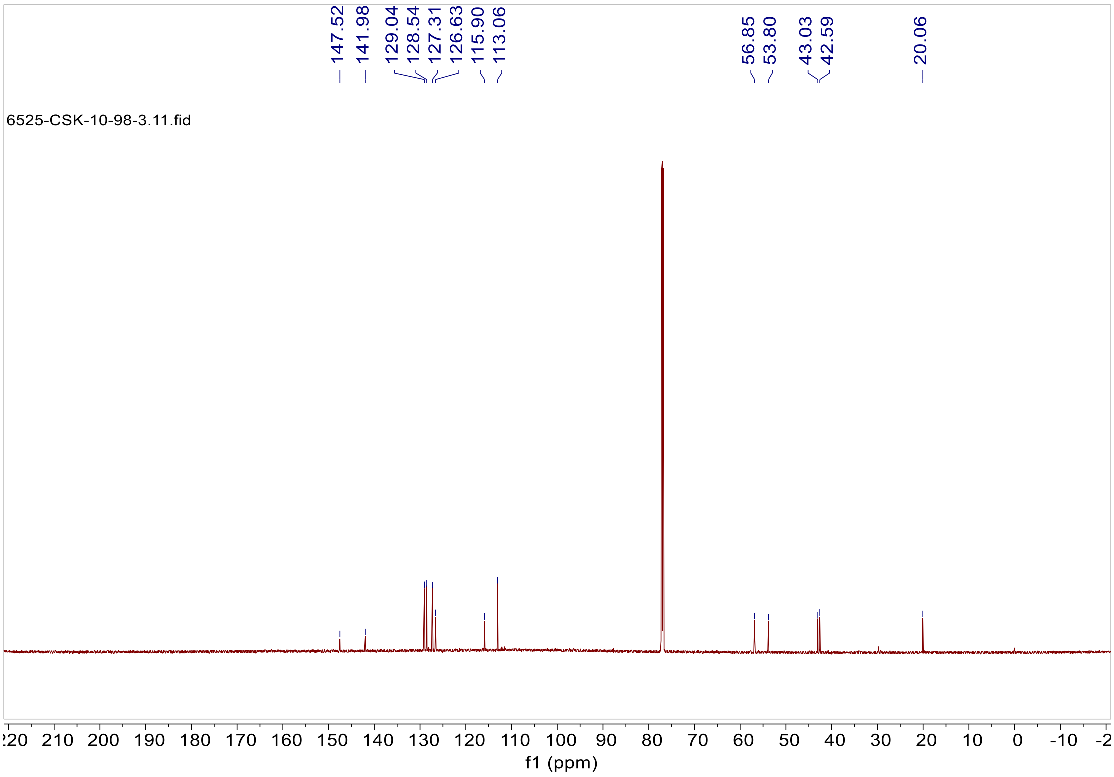

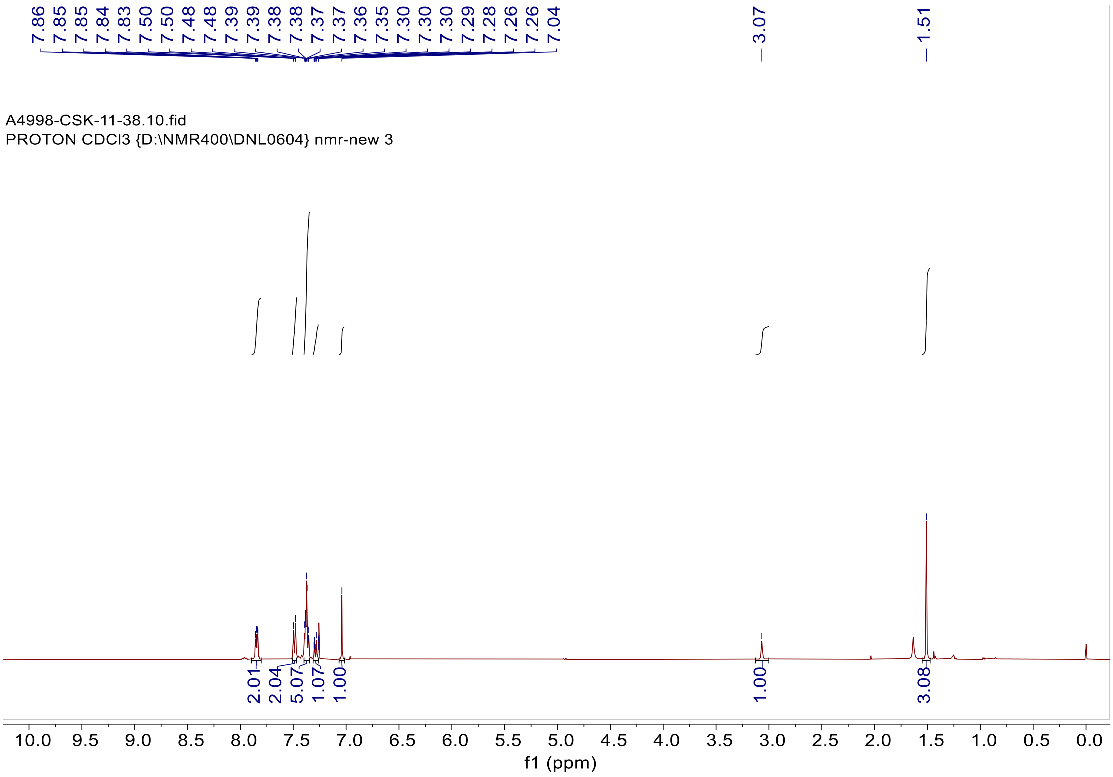

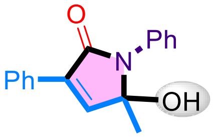

7a3

^{1}H NMR (400 MHz, $CDCl_3$)
^{13}C NMR (100 MHz, $CDCl_3$)

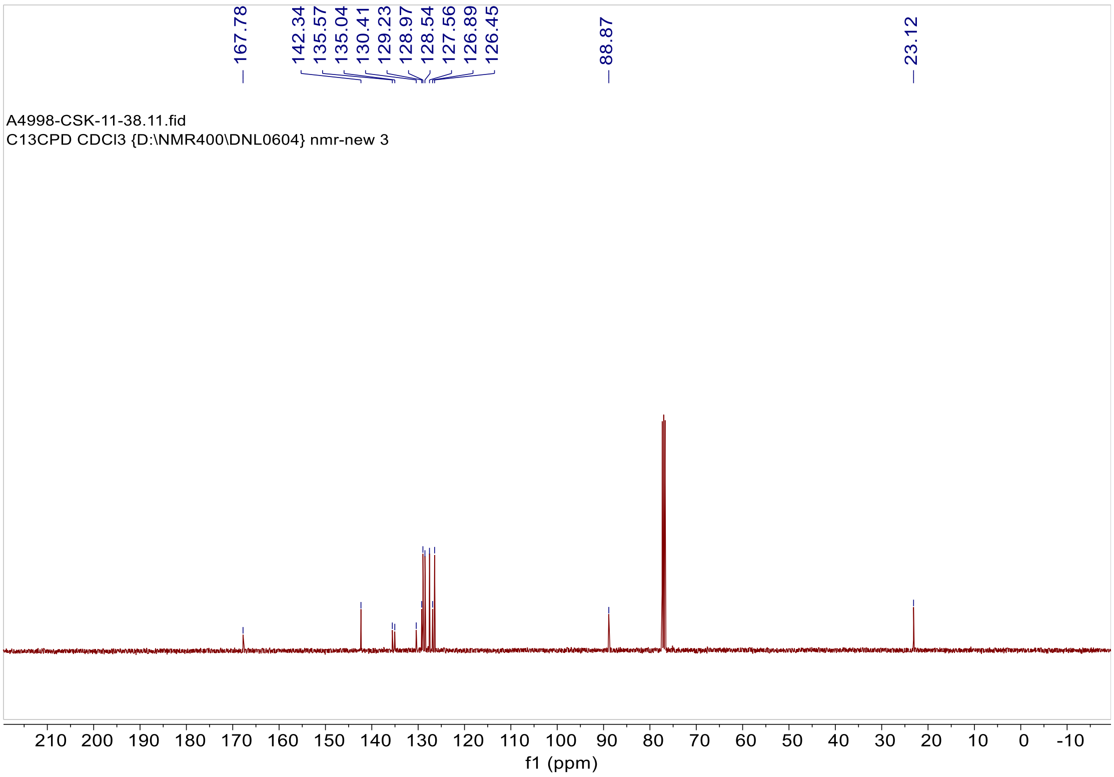

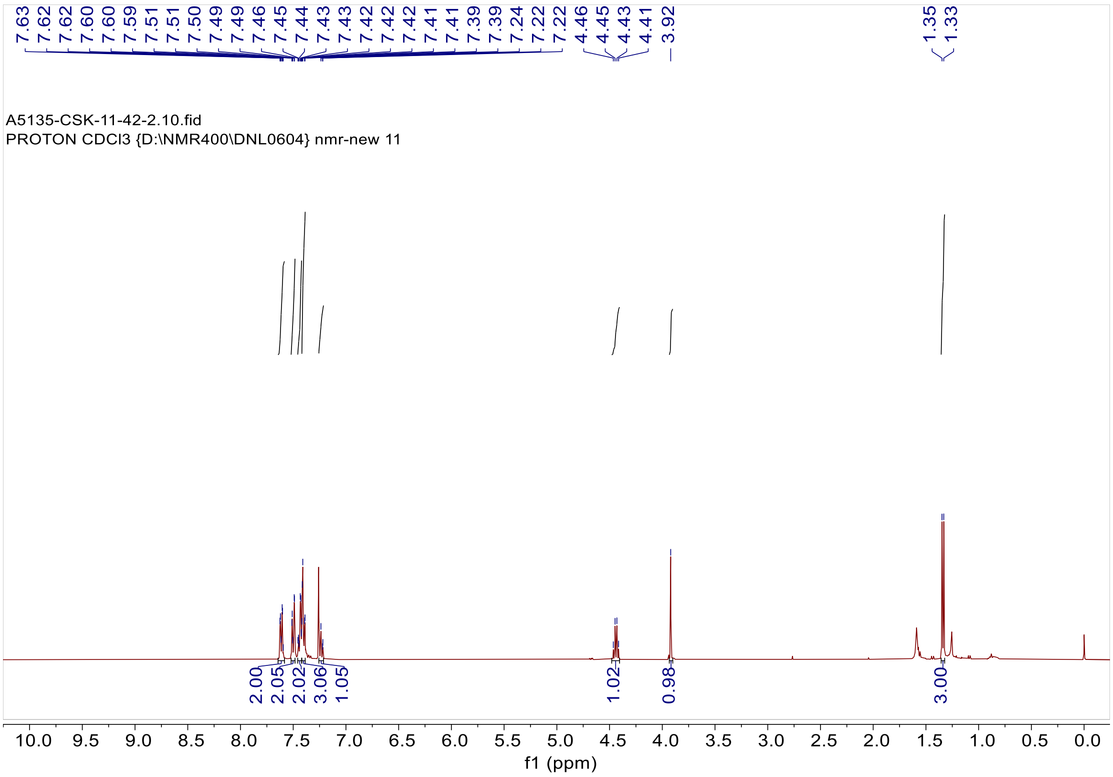

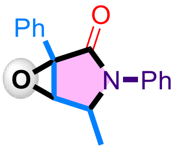

7a4

^{1}H NMR (400 MHz, $CDCl_3$)
^{13}C NMR (100 MHz, $CDCl_3$)

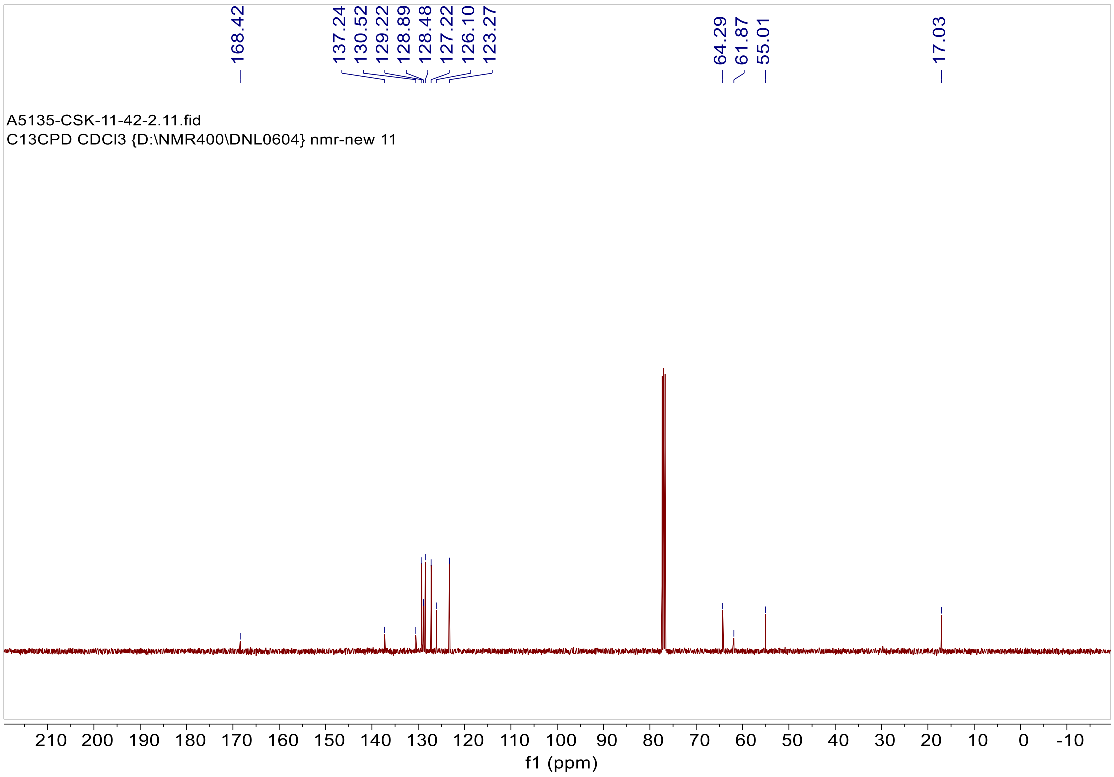

8. Mercury plot, crystal data and refinement results

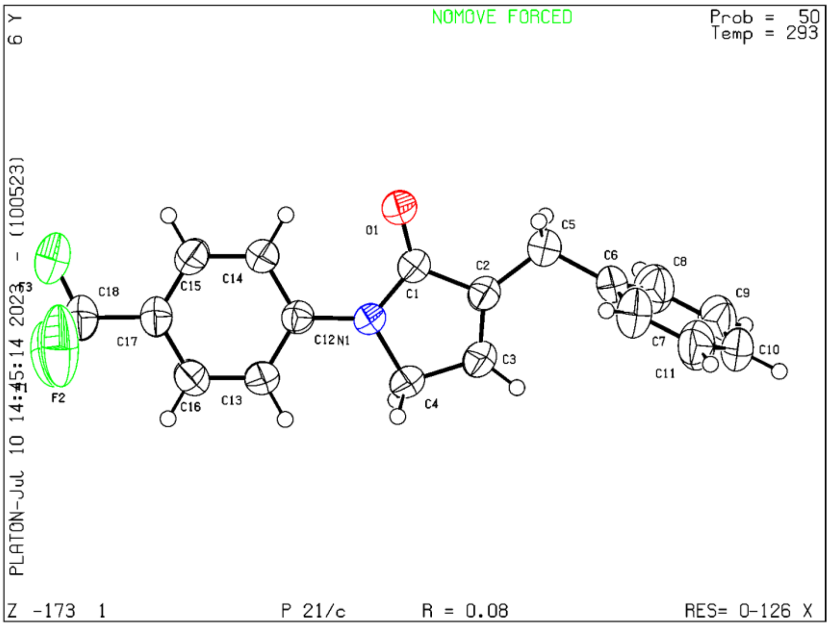

The Mercury plot of compound **5h**

Table S1. The crystal data and refinement results of compound **5h**.

Bond precision:	C-C = 0.0043 A		Wavelength=0.71073
Cell:	a=16.559(2)	b=8.2399(12)	c=11.1435(15)
	alpha=90	beta=93.120(12)	gamma=90
Temperature:	293 K		

	Calculated	Reported
Volume	1518.2(4)	1518.2(4)
Space group	P 21/c	P 21/c
Hall group	-P 2ybc	-P 2ybc
Moiety formula	C18 H14 F3 N O	?
Sum formula	C18 H14 F3 N O	C29 H29 F N29 O29
Mr	317.30	1266.81
Dx,g cm-3	1.388	1.386
Z	4	1
Mu (mm-1)	0.111	0.126
F000	656.0	647.0
F000'	656.42	
h,k,lmax	23,11,15	22,11,15
Nref	4311	3703
Tmin,Tmax	0.997,0.998	
Tmin'	0.996	

Correction method= Not given

Data completeness= 0.859 Theta(max)= 29.677

R(reflections)= 0.0762(2179) wR2(reflections)= 0.2434(3703)

S = 1.041 Npar= 208

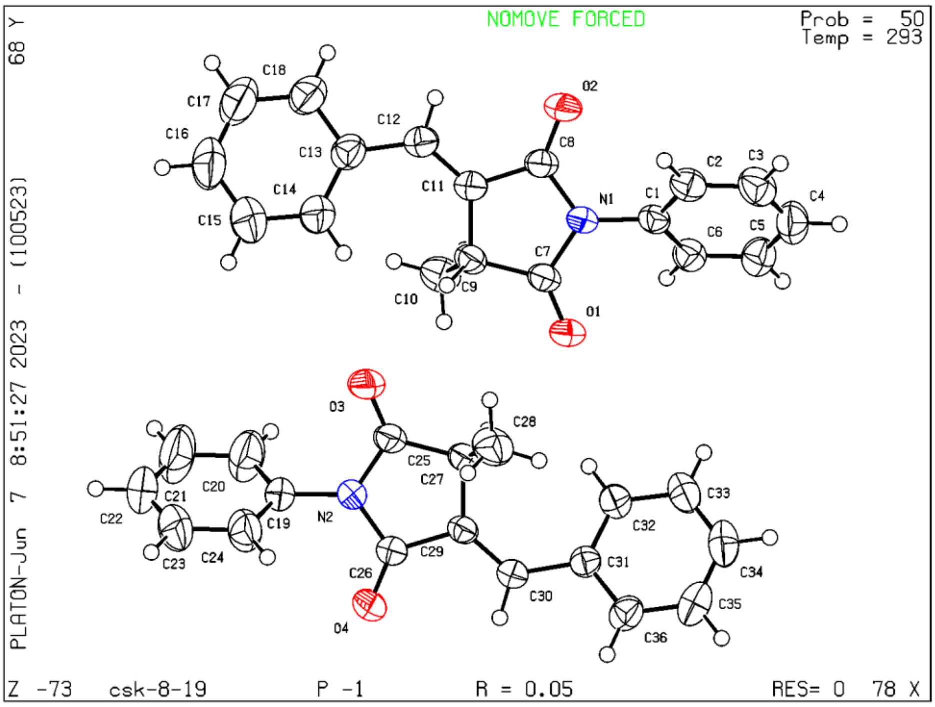

The Mercury plot of compound **6a**

Table S2. The crystal data and refinement results of compound **6a**.

Bond precision: C-C = 0.0029 A Wavelength=0.71073

Cell:	a=8.7076(8)	b=12.2837(14)	c=14.6303(11)
	alpha=96.454(8)	beta=98.136(7)	gamma=108.104(9)
Temperature:	293 K		

	Calculated	Reported
Volume	1452.0(3)	1452.0(3)
Space group	P -1	P -1
Hall group	-P 1	-P 1
Moiety formula	C18 H15 N O2	2(C18 H15 N O2)
Sum formula	C18 H15 N O2	C36 H30 N2 O4
Mr	277.31	554.62
Dx,g cm-3	1.269	1.269
Z	4	2
Mu (mm-1)	0.083	0.083
F000	584.0	584.0
F000′	584.26	
h,k,lmax	12,17,20	11,17,20
Nref	8299	7058
Tmin,Tmax	0.995,0.997	0.831,1.000
Tmin′	0.989	

Correction method= # Reported T Limits: Tmin=0.831 Tmax=1.000
AbsCorr = NONE

Data completeness= 0.850 Theta(max)= 29.781

R(reflections)= 0.0532(3974) wR2(reflections)= 0.1489(7058)

S = 1.015 Npar= 381

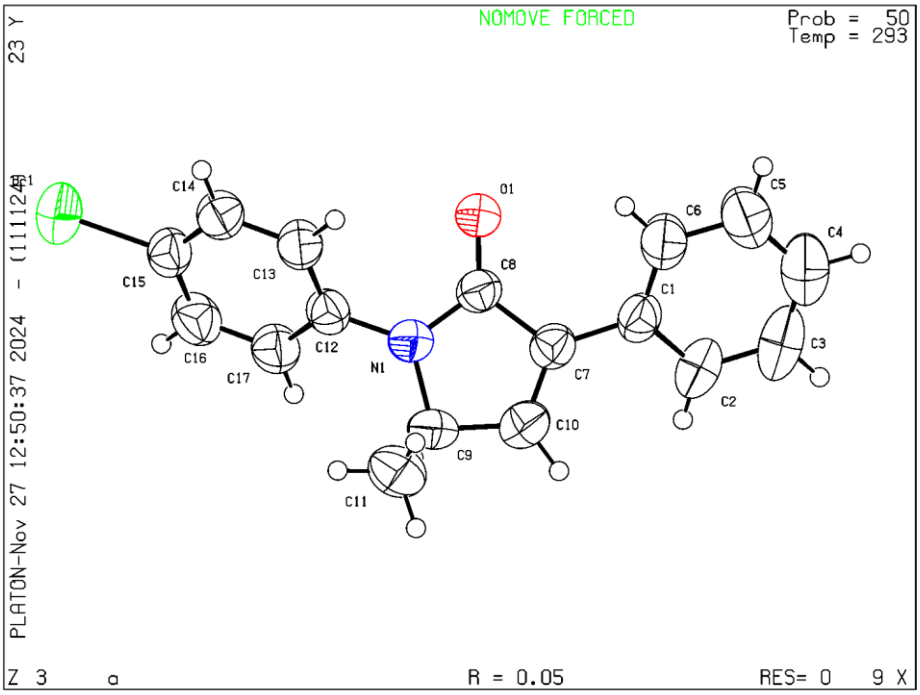

The Mercury plot of compound **7g**

Table S3. The crystal data and refinement results of compound **7g**.

Bond precision: C-C = 0.0072 A Wavelength=1.54178

Cell:	a=12.2200(15)	b=10.5406(13)	c=12.0723(16)
	alpha=90	beta=107.988(14)	gamma=90
Temperature:	293 K		

	Calculated	Reported
Volume	1478.980	1479.0(3)
Space group	P 21/c	?
Hall group	-P 2ybc	?
Moiety formula	C17 H14 Br N O	?
Sum formula	C17 H14 Br N O	C17 H14 Br N O
Mr	328.19	328.20
Dx,g cm-3	1.474	1.474
Z	4	4
Mu (mm-1)	3.736	3.736
F000	664.0	664.0
F000'	662.77	
h,k,lmax	14,12,14	14,12,14
Nref	2636	2612
Tmin,Tmax		
Tmin'		

Correction method= Not given

Data completeness= 0.991 Theta(max)= 66.970

R(reflections)= 0.0499(1827) wR2(reflections)= 0.1783(2612)

S = 1.097 Npar= 182

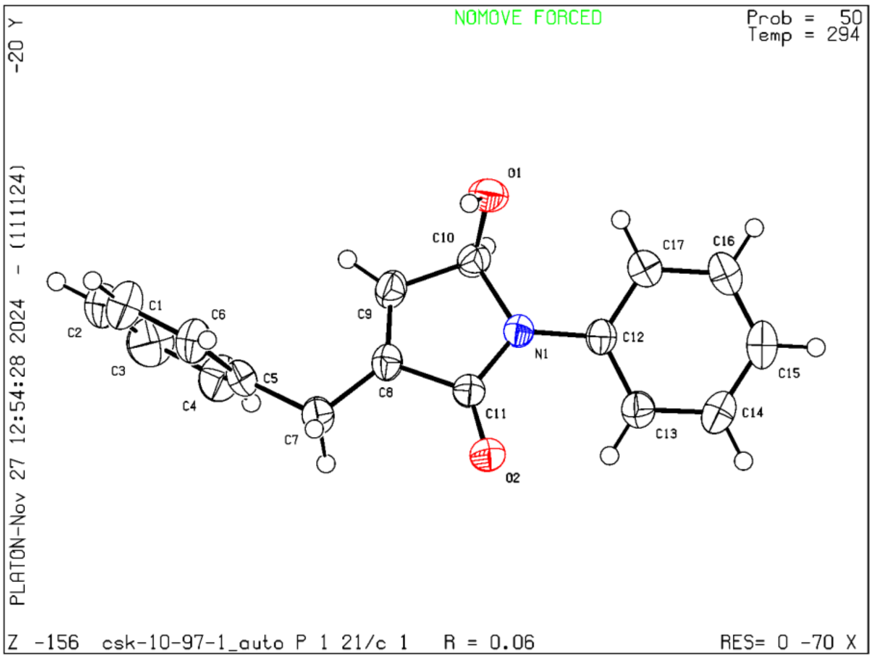

The Mercury plot of compound **5a3**

Table S4. The crystal data and refinement results of compound **5a3**.

Bond precision: C-C = 0.0027 A Wavelength=1.54178

Cell:	a=11.1240(7)	b=9.2213(6)	c=13.1263(9)
	alpha=90	beta=93.634(6)	gamma=90
Temperature:	294 K		

	Calculated	Reported
Volume	1343.76(15)	1343.76(15)
Space group	P 21/c	P 1 21/c 1
Hall group	-P 2ybc	-P 2ybc
Moiety formula	C17 H15 N O2	C17 H15 N O2
Sum formula	C17 H15 N O2	C17 H15 N O2
Mr	265.30	265.31
Dx,g cm-3	1.311	1.311
Z	4	4
Mu (mm-1)	0.690	0.690
F000	560.0	561.8
F000’	561.67	
h,k,lmax	13,10,15	13,10,15
Nref	2374	2355
Tmin,Tmax		0.285,1.000
Tmin’		

Correction method= # Reported T Limits: Tmin=0.285 Tmax=1.000
AbsCorr = MULTI-SCAN

Data completeness= 0.992 Theta(max)= 66.650

R(reflections)= 0.0600(2157) wR2(reflections)= 0.1546(2355)

S = 1.036 Npar= 182

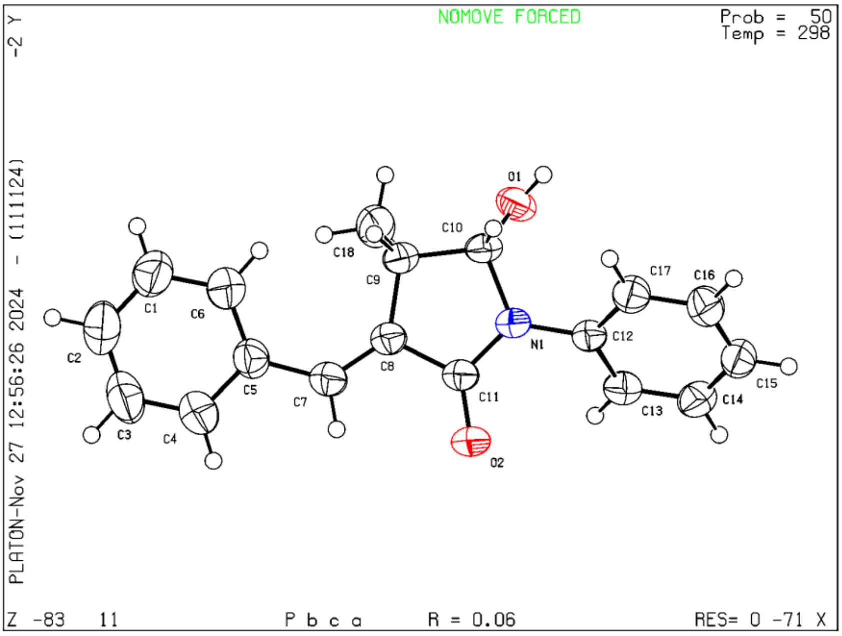

The Mercury plot of compound **6a3**

Table S5. The crystal data and refinement results of compound **6a3**.

```
Bond precision:   C-C = 0.0033 A          Wavelength=1.54178

Cell:              a=12.8038(5)      b=8.3396(4)      c=27.3329(11)
                   alpha=90          beta=90          gamma=90
Temperature:       298 K

                  Calculated                  Reported
Volume            2918.6(2)                   2918.6(2)
Space group       P b c a                     P b c a
Hall group        -P 2ac 2ab                  -P 2ac 2ab
Moiety formula    C18 H17 N O2                ?
Sum formula       C18 H17 N O2                C18 H17 N O2
Mr                279.33                      279.32
Dx,g cm-3         1.271                       1.271
Z                 8                           8
Mu (mm-1)         0.661                       0.661
F000              1184.0                      1184.0
F000'             1187.47
h,k,lmax          15,9,32                     15,9,32
Nref              2579                        2570
Tmin,Tmax         0.870,0.888                 0.359,1.000
Tmin'             0.870

Correction method= # Reported T Limits: Tmin=0.359 Tmax=1.000
AbsCorr = MULTI-SCAN

Data completeness= 0.997          Theta(max)= 66.812

                                                    wR2(reflections)=
R(reflections)= 0.0574( 2150)                       0.1578( 2570)

S = 1.077                   Npar= 192
```
